# A long-term perspective on Neanderthal environment and subsistence: Insights from the dental microwear texture analysis of hunted ungulates at Combe-Grenal (Dordogne, France)

**DOI:** 10.1371/journal.pone.0278395

**Published:** 2023-01-18

**Authors:** Emilie Berlioz, Eugénie Capdepon, Emmanuel Discamps

**Affiliations:** 1 UMR5608 TRACES, Team SMP3C, Toulouse, France; 2 Grupo I+D+i EVOADAPTA, Universidad de Cantabria, Santander, Spain; Griffith University, AUSTRALIA

## Abstract

Large bovids and cervids constituted major components of the European Middle Palaeolithic faunas and hence a key resource for Neanderthal populations. In paleoenvironmental reconstructions, red deer (*Cervus elaphus*) occurrence is classically considered as a tree-cover indicator while Bovinae (*Bison priscus* and *Bos primigenius*) and reindeer (*Rangifer tarandus*) occurrences are typically associated with open landscapes. However, insights into the ecology of extant ungulate populations show a more complex reality. Exploring the diet of past ungulates allows to better comprehend the hunting strategies of Palaeolithic populations and to reconstruct the modifications through time of past landscapes. By reflecting what animals have eaten during the last days or weeks of their life, dental microwear textures of herbivores link a population and its environment. Here we analyzed, via Dental Microwear Texture Analysis (DMTA), the diet of 50 *Bos/Bison*, 202 *R*. *tarandus* and 116 *C*. *elaphus* preyed upon by the Neanderthals that occupied Combe-Grenal rock-shelter, one of the most important Mousterian archaeo-sequences in southwestern France considering its long stratigraphy, abundance of faunal remains and the variations perceptible in Palaeolithic material culture. Grazers and mixed-feeders are the most represented dietary categories among Combe-Grenal’s guild of herbivores, highlighting the availability, along the sequence, of open landscapes. The absence of clear changes in the use of plant resources by hunted ungulates through time, even though palaeoenvironmental changes were well-documented by previous studies along the sequence, is interpreted as resulting from the hunting of non-randomly selected prey by Neanderthals, preferentially in open environments. Thus, these results provide further insight into the hunting strategies of Neanderthals and modify our perception of potential links between subsistence and material culture. Combe-Grenal hunters “stayed in the open” through millennia, and were not forced to switch to hunting tactics and material technology adapted to close encounters in forested environments.

## Introduction

When considering prehistoric societies whose subsistence relied heavily on the hunting of large mammals, knowledge of the animal communities they preyed upon is key to better contextualize human–environment interactions [1]. Reindeer (*Rangifer tarandus*), red deer (*Cervus elaphus*), steppe bison (*Bison priscus*) and aurochs (*Bos primigenius*) constituted key-resources for hunter-gatherer groups in Eurasia, and were often at the heart of Palaeolithic subsistence economies. Understanding the ethology and ecology of these preyed species (social, spatial, seasonal behavior, feeding ecology) and their long-term evolution is essential to better comprehend human behaviors during the Palaeolithic as these factors ultimately condition human acquisition, subsistence and mobility strategies. It also enlarges our understanding of how the global climatic oscillations have affected local paleoenvironments and human groups.

The impact of habitat anthropization in present days on most modern terrestrial ecosystems, resulting in the use of peri-optimal niches and refugee areas as a consequence of habitat loss and human activities notably for several large mammals [2–4], strongly skew our understanding of the ecological optimum and plasticity of these extant species. It is, however, this ecological knowledge acquired on extant communities that is so often used as habitat proxies to interpret past environmental conditions. This situation leads to undervaluing the potential ecological plasticity of ungulates, leading in turn to flawed paleoecological and paleoenvironmental inferences based on fossil ungulate occurrences [5] and limited understanding of the interactions of these key prey species with prehistoric societies. For example, European Bison (*Bison bonasus*) populations changed their ecology from a grazing diet in open landscapes toward a mixed-feeding behavior in more forested refugial habitats during the Holocene (11.7 ka cal. BP—present) as a response to human landscape transformations [6–9]. Such a change constitutes a perfect illustration of the complementarity between paleoecological and neoecological points of view when it comes to deciphering the ecological optimum and plasticity of a species [10, 11]. In that respect, studies relying only on the occurrence or frequency of macrofaunal species in fossil assemblages most likely miss a high proportion of paleoecological and paleoenvironmental information. To overcome these limitations, taxonomy-free analytical techniques taking the ecological plasticity into account are required. Among these, 3D Dental Microwear Texture Analyses (DMTA; [12–14]) constitute an efficient way to assess food type consumed and thus explore past ecologies, vegetal structure of local habitats and to infer paleoenvironments. This approach is based on the study of dental textures resulting from the ingested food items during the last days or weeks of the life of an animal [15–17].

To enhance zooarchaeological and paleoenvironnemental interpretations, we selected a key sequence from the European Middle Palaeolithic (Combe-Grenal, Dordogne, France) that recorded Neanderthal prey choices in the long term (over several dozens of millennia), and that has been at the center of research on past environments and subsistence strategies for decades [e. g. 18–26].

In Dordogne as in many other regions of Western Europe, this period is characterized by rapid and acute climatic oscillations [27, 28] that affected plant communities [29, 30] and, in turn, shaped micro-mammal and large mammal faunal communities [1, 31]. In the Combe-Grenal sequence (ca. 150,000 to 45,000 BP) as in other archeological sites, Neanderthals adapted their subsistence strategies to changes in animal populations. Reindeer (*Rangifer tarandus*), red deer (*Cervus elaphus*) and large bovids (steppe bison *Bison priscus* and/or aurochs *Bos primigenius*) constituted key resources for hunter-gatherer groups, providing both food (such as meat, marrow or grease) and raw materials (such as skin, bone, horn or antler). In this contribution, we present a long-term cross-species DMTA study of these three taxa (reindeer, red deer and Bovinae) that are present throughout the Combe-Grenal sequence. Exploring the evolution of the feeding behavior of these herbivores is a first-choice approach to enlarge our understanding of the impact of climate change on human-ungulate-environment relationships during the Middle Palaeolithic.

With this study, we aim at fulfilling three objectives: (i) explore the paleoecology (notably the feeding preferences) of bovids and cervids hunted by Neanderthals at Combe-Grenal, (ii) contribute to a better understanding of Combe-Grenal’s paleoenvironment and its evolution through time, and (iii) provide new insights on the subsistence strategies of Neanderthal populations, from the perspective of ungulate paleoecology. We test whether or not the evolution of the feeding ecology of preyed ungulate species through time reflect palaeoenvironmental changes throughout the sequence. This would allow us to better understand the impact of well-known global climate changes on the local paleoenvironments of Neanderthal hunting grounds.

## Material & methods

### Archeological site: Combe-Grenal

**Fig 1:**
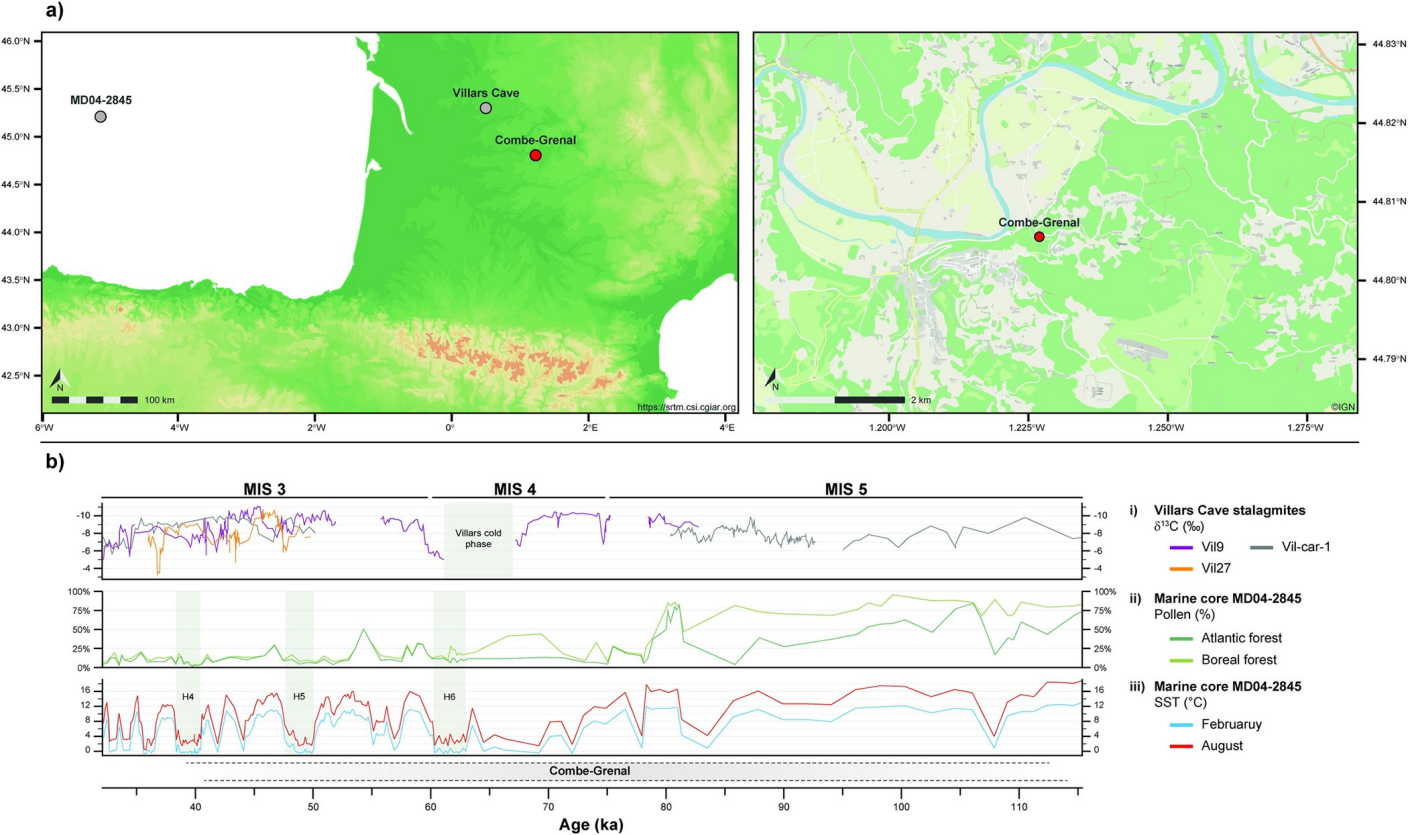
Combe-Grenal rock-shelter: Geographic, climatic and environmental context. a) Localisation: red dot: Combe-Grenal; grey dots: key regional environmental proxies. Sources of the maps: https://srtm.csi.cgiar.org/; IGN. b) Environmental and climatic context of Combe-Grenal, based on independent environmental proxies [27–30]: i) δ^13^C isotopic records from Villars Cave stalagmites; ii) Pollen percentages of the boreal and altantic forests iii) Sea Surface Temperatures records from the marine core MD04-2845. Heinrich events 4–6 are indicated by light grey intervals. It is worth noting that, as the part of Combe-Grenal sequence studied here has not yet been adequately dated by radiometric methods, correlations between climatic events, environmental changes and the site stratigraphy remain uncertain.

Combe-Grenal (Domme, southwestern France; Fig 1) is a rock-shelter situated in close proximity to the Dordogne River valley in the Périgord region. The 13m-long sequence was excavated during the 20^th^ century by, successively, D. Peyrony, E. Peyrony and F. Bordes, before being the focus of a new field project since 2014 led by J.-Ph. Faivre and E. Discamps [22, 24, 25]. F. Bordes’ excavations from 1953 to 1965 over 200 m^2^ were the most extensive, and contributed in placing Combe-Grenal as one of the most important Middle Palaeolithic sequences of Western Europe. F. Bordes defined 65 layers in three superimposed terraces (“paliers”) spanning MIS 6 to MIS 3 [22, 24, 25]. For this study, we focus on the uppermost terrace (layers 1 to 36) that yielded the most information on Middle Palaeolithic cultural and paleoenvironmental variability [26].

Bordes’ collections are characterized by abundant faunal remains [18, 21], (12,000 specimens, belonging to 27 different species and representing more than 550 individuals) witnessing several clear faunal turnovers [1, 22]. At Combe-Grenal, 29 Neanderthal remains, some bearing cutmarks, were also discovered [24, 32], alongside more than 144,000 lithic artefacts relating to all of the main technological flake production systems recognized in the Mousterian of southwestern Europe (notably Levallois, Quina and Discoid; cf. [26] for a recent review). These rich industries contributed to the definition of the different “Mousterian facies” as defined by F. Bordes. The succession of technological systems defines a clear stratigraphic succession and put Combe-Grenal at the heart of the debate on Mousterian variability in Western Europe [26].

**Fig 2:**
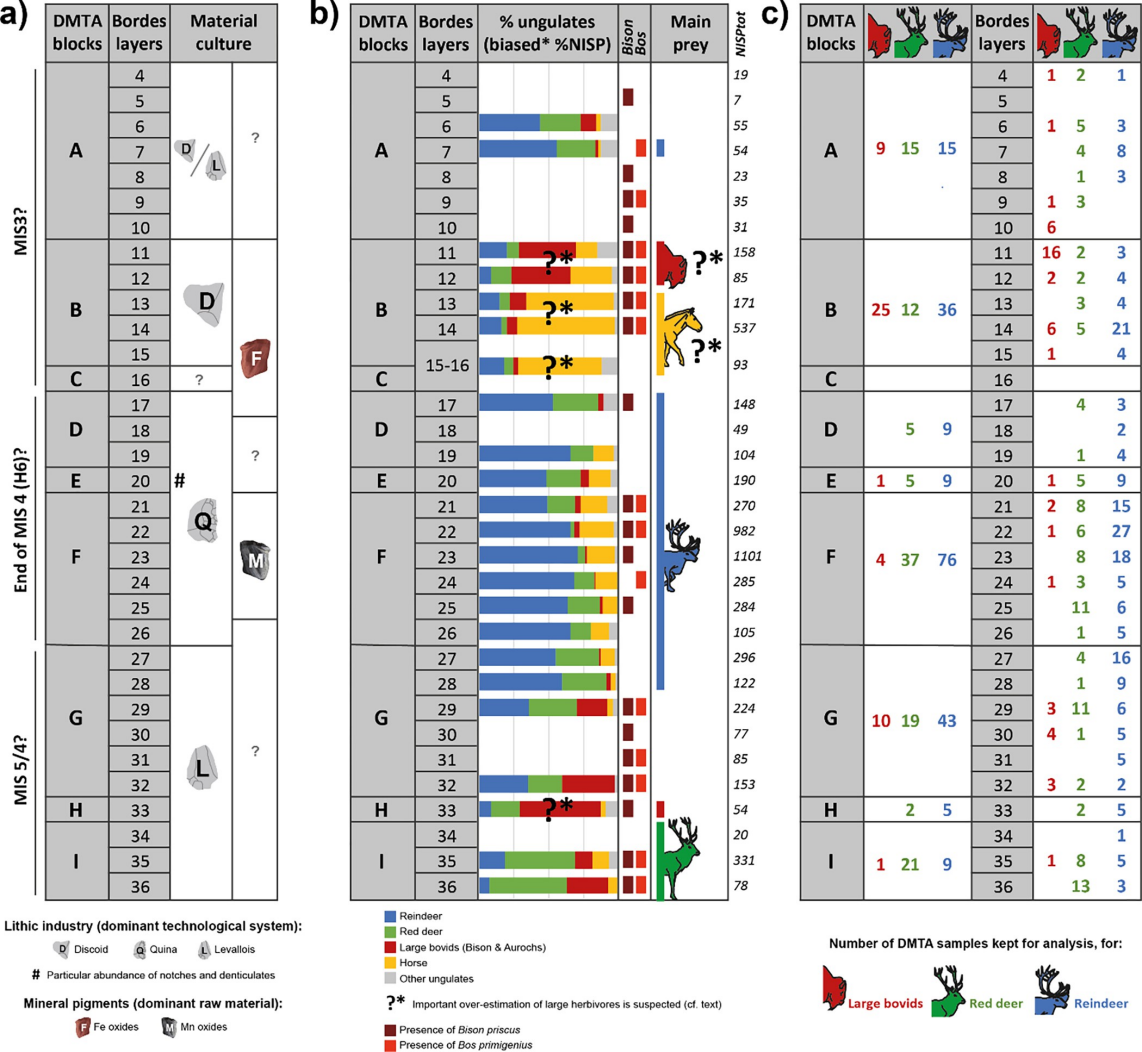
Contextual data and distribution of DTMA samples for layers 36 to 4 of Combe-Grenal. a) Summary of material culture evidence for lithic industries [26] and mineral pigments [33], b) ungulate proportions (only layers with a total ungulate NISP [number of identified specimens] of more than 50 are figured), presence/absence of Bison priscus and Bos primigenius, and total ungulate NISP in italics [18, 21], c) number of DMTA samples included in the present study, both by “blocks” as defined in this study (cf. text) and for each of Bordes’ layers.

Recent fieldwork has provided key comparative data highlighting important recovery bias in previous excavations; proportions of the largest herbivores are highly over-estimated in at least some of Bordes’ layers, as a consequence of preferential selection of teeth and larger bone fragments during excavation [22]. Bordes’ collections are however still invaluable by their size and stratigraphic extent. Provided that the number of exploitable specimens is sufficient, it is worth mentioning that, unlike other approaches that depend on the relative abundance of remains, DMTA is unaffected by such recovery bias, and thus allows us to reliably establish a correlation between the feeding behavior of a population and the type of environment it occupies. For this study, we sampled Bordes’ layers 4 to 36 (Fig 2).

That part of the sequence has not yet been adequately dated by radiometric methods. According to the most recent comparative chronological data [1, 26, 31] and pending the acquisition of new absolute dates, it has been tentatively correlated with MIS 5 or the beginning of MIS4 (layers 36 to 27, 115–65 ka cal. BP?), the end of MIS4 and Heinrich Stadial 6 (layers 26 to 17, 65–60 ka cal. BP?) and the first half of MIS3 (layers 16 to 4, 60–43 ka cal. BP?). As illustrated in Fig 1, MIS 4 and 3 are characterized by rapid cycles of forest cover expansion and contraction in response to the millenial-scale climatic changes of the Dansgaard-Oeschger and Heinrich events, with a progressive trend towards more open environments [27–30].

Fig 2 summarizes contextual data available for layers 36 to 4. Material culture shows the succession of lithic industries dominated by Levallois, Quina and Discoid production systems [see 26 for a synthesis], as well as a change in mineral pigment use, with layers dominated by manganese oxides or iron oxides [33] (Fig 2A). Paleontological analysis of these layers by J.-L. Guadelli [20, 21] (layers 4–35) and G. Laquay [18], (layer 36) provided detailed information on the herbivores hunted by Neanderthals. Generally speaking, Combe-Grenal upper terrace sees a succession of faunas dominated by red deer (layers 36 to 34), Bovinae (layer 33), followed by a transition (layers 32 to 29) towards reindeer-dominated faunas (layers 28 to 17; Fig 2B). In layers 16 to 11, horse and large bovids are the most abundant, but new fieldwork highlighted the over-representation of large ungulates in these layers as a consequence of recovery bias [22]. Large bovids cannot always be identified to species, but a few specimens of both *Bison priscus* (n = 77) and *Bos primigenius* (n = 24) have been formerly identified in almost all layers [18, 21], (Fig 2B). When both species are present in the same layer, *Bison priscus* is more abundant in 10 out of 12 cases, but sample sizes are low (N≤17).

### Archeological material

For this study, we analyzed the teeth of 116 red deer (*Cervus elaphus)*, 202 reindeer (*Rangifer tarandus)* and 50 Bovinae (Fig 2C). Both the steppe bison *Bison priscus* and the aurochs *Bos primigenius* are present at Combe-Grenal. The morphological proximity between the dental material of these two bovids makes specific determination difficult. Therefore, the majority of the bovid dental remains fall into the category "*Bos/Bison*" [21].

Specimens belonging to the four species were selected from the Middle Palaeolithic levels 4 to 36 of Combe-Grenal Bordes’ collections, on the basis of the quality of the preservation of their dental facets. These four ungulate species, present in most of Combe-Grenal sequence, are characterized by a great abundance associated with a large paleobiogeographical distribution, the combination of both characteristics suggesting the use of diversified habitats and an important ecological plasticity. Bordes’ collections are stored at the Musée National de la Préhistoire (MNP) of Les Eyzies, France. No permits were required for the described study, which complied with all relevant regulations.

### Extant species as comparative dataset

Seven extant ungulate populations with well-known diets were also included in the analyses to serve as a reference framework to interpret the diet of fossils (Table 1). The DMTA of all reference populations have been the subject of previous studies [5, 7, 34–36].

**Table 1:** Reference populations used in the present study, classified by their dietary category. N = sample size. In the figures, these populations are referred with a population number, given in the last column.

Dietary category	Species	Locality	N	References	n°
Grazer	*Bos taurus*	Camargue Natural Regional Park, France	44	[35]	2
	*Cervus elaphus*	Bauges Natural Regional Park, Bauges, France	23	[5, 36]	3
	*Rangifer tarandus*	Knutshø, Forollhogna National Park, Norway	52	[5, 34]	7
Mixed-Feeder	*Bison bonasus*	Białowieża primeval Forest, Poland	19	[7]	1
	*Cervus elaphus*	Domaniale forest of Chateauroux, France	29	[5]	5
	*Rangifer tarandus*	Hardangervidda National Park, Norway	68	[5, 34]	6
Browser	*Cervus elaphus*	Białowieża primeval Forest, Poland	23	[5, 7]	4

### DMTA implementation

Lower second molars were preferentially selected for the analysis. When this tooth was not available or too altered, the 1^st^ or 3^rd^ lower molar was selected. Details on the fossil sample are provided in S1 Appendix. Each tooth was carefully cleaned with a cotton swab soaked in ethanol. Once totally dry, the disto-lingual facet of the protoconid of lower molars was preferentially molded with a high-resolution polyvinylsiloxane elastomer (Regular Body President, ref. 6015–ISO 4823, medium consistency, polyvinylsiloxane addition type; Coltene Whaledent; see details on the procedures in the video here: http://anr-trident.prd.fr/v/; https://doi.org/10.5281/zenodo.7305567). The mold was then scanned directly with a Leica DCM8 white light confocal surface profilometer with a 100× objective (numerical aperture = 0.90, working distance = 0.9 mm) at the PAE2O “ArchéoScience” platform (UMR5608 TRACES, Toulouse, France). For each specimen we acquired a 251 × 333 μm surface (2584 × 1945 points, z-step: 0.2 μm) at the center of the dental facet. With LeicaMap 8.0.9, we then sampled a 200 × 200 μm sub-surface, leveled and mirrored in Z. The few missing points (<3%) were replaced by a smooth shape, using an algorithm calculated from the neighboring points (NMP ratio after cleaning: 0%). We applied the macro developed by Merceron et al. [37] to remove abnormal peaks. Remaining bigger artifacts and exogenous particles were removed manually and replaced by a smooth shape before proceeding to a final leveling of the surface. Photosimulations and false-color elevation maps of the processed data are available in S2 Appendix. All the data, both raw scans acquired with the Leica DCM8 (.plux files) and pre-treated data (.mnt files), are available on the NAKALA repository at the following URL: https://doi.org/10.34847/nkl.f4b0q9rm. Texture parameters were generated using a Scale Sensitive Fractal Analysis (SSFA) with the same software. In this study we considered area-scale fractal complexity (*Asfc*; Tables 2 and 3, S1 Appendix), exact proportion of the length-scale anisotropy of the relief (*epLsar*; Tables 2 and 3, S1 Appendix), Scale of maximum complexity (*Smc*; Tables 2 and 3, S1 Appendix) and heterogeneity of the area-scale fractal complexity at 9, 36 and 81 cells (*HAsfc 9*, *HAsfc 36*, *HAsfc 81*; Tables 2 and 3, S1 Appendix). These parameters are further described in Scott [13] and Scott et al. [12].

**Table 2:** Values for DMTA-SSFA parameters for the three ungulate taxa from Combe-Grenal and for extant reference populations. N = sample size; s.e.m. = standard error of mean. **Periods**: Ante-Quina: levels 27 to 36, Quina: levels 17 to 26, Post-Quina: levels 4 to 16. **DMTA-SSFA parameters**: Asfc: complexity; epLsar: anisotropy; Smc: Scale of maximum complexity; HAsfc 9, 36, 81: heterogeneities of the complexity (3x3, 6x6, 9x9 cells).

**Fossil populations**			**Asfc**		**epLsar (10** ^**−3**^ **)**		**Smc**		**HAsfc 9**		**HAsfc 36**		**HAsfc 81**	
	**Period**	**N**	**mean**	***s*.*e*.*m*.**	**mean**	***s*.*e*.*m*.**	**mean**	***s*.*e*.*m*.**	**mean**	***s*.*e*.*m*.**	**mean**	***s*.*e*.*m*.**	**mean**	***s*.*e*.*m*.**
*Bos/Bison* (N = 50)	**Ante-Quina**	11	2.99	*0*.*61*	4.35	*0*.*66*	1.40	*0*.*27*	0.280	*0*.*041*	0.450	*0*.*037*	0.559	*0*.*050*
	**Quina**	5	2.42	*1*.*08*	4.96	*1*.*39*	11.75	*9*.*50*	0.418	*0*.*135*	0.604	*0*.*138*	0.851	*0*.*202*
	**Post-Quina**	34	2.11	*0*.*14*	5.19	*0*.*43*	1.70	*0*.*18*	0.304	*0*.*027*	0.464	*0*.*025*	0.602	*0*.*032*
*Cervus elaphus* (N = 116)	**Ante-Quina**	42	1.66	*0*.*12*	5.29	*0*.*35*	12.22	*4*.*50*	0.365	*0*.*023*	0.563	*0*.*042*	0.683	*0*.*026*
	**Quina**	47	1.42	*0*.*12*	5.22	*0*.*32*	27.71	*7*.*48*	0.448	*0*.*047*	0.631	*0*.*062*	0.810	*0*.*072*
	**Post-Quina**	27	1.87	*0*.*19*	5.77	*0*.*45*	20.73	*11*.*37*	0.353	*0*.*023*	0.505	*0*.*024*	0.669	*0*.*031*
Rangifer tarandus (N = 202)	**Ante-Quina**	57	1.83	*0*.*11*	5.03	*0*.*23*	4.54	*1*.*47*	0.362	*0*.*023*	0.553	*0*.*037*	0.685	*0*.*045*
	**Quina**	94	2.09	*0*.*12*	4.21	*0*.*19*	6.31	*2*.*61*	0.373	*0*.*018*	0.538	*0*.*024*	0.684	*0*.*026*
	**Post-Quina**	51	2.30	*0*.*15*	4.52	*0*.*31*	15.29	*5*.*04*	0.312	*0*.*017*	0.501	*0*.*022*	0.641	*0*.*029*
**Extant populations**		**N**	**mean**	***s*.*e*.*m*.**	**mean**	***s*.*e*.*m*.**	**mean**	***s*.*e*.*m*.**	**mean**	***s*.*e*.*m*.**	**mean**	***s*.*e*.*m*.**	**mean**	***s*.*e*.*m*.**
***1*: *Bison bonasus* (Białowieża Forest, Poland)**		19	2.03	*0*.*25*	3.89	*0*.*48*	4.25	*1*.*31*	0.381	*0*.*052*	0.603	*0*.*086*	0.781	*0*.*104*
***2*: *Bos taurus* (Camargue, France)**		44	1.54	*0*.*14*	5.29	*0*.*31*	1.25	*0*.*10*	0.421	*0*.*046*	0.559	*0*.*041*	0.722	*0*.*062*
***3*: *Cervus elaphus* (Bauges, France)**		23	1.27	*0*.*12*	6.11	*0*.*52*	12.92	*5*.*32*	0.497	*0*.*106*	0.641	*0*.*098*	0.833	*0*.*120*
***4*: *Cervus elaphus* (Białowieża Forest, Poland)**		23	2.82	*0*.*36*	3.11	*0*.*26*	5.82	*1*.*82*	0.443	*0*.*084*	0.674	*0*.*062*	0.925	*0*.*081*
***5*: *Cervus elaphus* (Chateauroux, France)**		29	2.13	*0*.*15*	4.29	*0*.*37*	8.29	*2*.*59*	0.452	*0*.*045*	0.651	*0*.*061*	0.873	*0*.*082*
***6*: *Rangifer tarandus* (Hardangervidda, Norway)**		68	2.30	*1*.*08*	3.15	*1*.*54*	9.00	*21*.*23*	0.460	*0*.*294*	0.769	*0*.*342*	0.822	*0*.*398*
***7*: *Rangifer tarandus* (Knutshø, Norway)**		52	1.76	*0*.*64*	4.21	*2*.*03*	17.93	*35*.*38*	0.410	*0*.*206*	0.698	*0*.*300*	0.714	*0*.*311*

**Table 3:** Values for DMTA-SSFA parameters in each block for the three ungulate taxa from Combe-Grenal. N = sample size; s.e.m. = standard error of mean. Block A: levels 4 to 10; Block B: levels 11 to 15; Block C: level 16 (no DMTA sample), Block D: levels 17 to 19; Block E: level 20; Block F: levels 21 to 26; Block G: levels 27 to 32; Block H: level 33; Block I: levels 34 to 36. **DMTA-SSFA parameters**: Asfc: complexity; epLsar: anisotropy; Smc: Scale of maximum complexity; HAsfc 9, 36, 81: heterogeneities of the complexity (3x3, 6x6, 9x9 cells).

Fossils			Asfc		epLsar (10–3)		Smc		HAsfc 9		HAsfc 36		HAsfc 81	
	Block	N	mean	*s*.*e*.*m*.	mean	*s*.*e*.*m*.	mean	*s*.*e*.*m*.	mean	*s*.*e*.*m*.	mean	*s*.*e*.*m*.	mean	*s*.*e*.*m*.
*Bos/Bison*	**ALL**	**50**	**2.34**	***0*,*19***	**4,98**	***0*,*35***	**2,64**	***0*,*97***	**0,310**	***0*,*024***	**0,475**	***0*,*023***	**0,617**	***0*,*032***
	**A**	9	2.19	*0*.*34*	4.46	*0*.*93*	1.67	*0*.*16*	0.380	*0*.*066*	0.516	*0*.*061*	0.656	*0*.*077*
	**B**	25	2.08	*0*.*14*	5.45	*0*.*48*	1.71	*0*.*24*	0.277	*0*.*026*	0.445	*0*.*027*	0.582	*0*.*033*
	**C**			* *		* *		* *		* *		* *		* *
	**D**			* *		* *		* *		* *		* *		* *
	**E**	1	0.92	* *	8.43	* *	3.35	* *	0.162	* *	0.384	* *	0.508	* *
	**F**	4	2.80	*1*.*31*	4.09	*1*.*41*	13.85	*11*.*96*	0.482	*0*.*153*	0.659	*0*.*163*	0.936	*0*.*235*
	**G**	10	3.08	*0*.*67*	4.17	*0*.*69*	1.40	*0*.*30*	0.268	*0*.*043*	0.449	*0*.*041*	0.559	*0*.*055*
	**H**			* *		* *		* *		* *		* *		* *
	**I**	1	2.1183	* *	6.2216	* *	1.361	* *	0.3965	* *	0.4688	* *	0.5627	* *
*C*. *elaphus*	**ALL**	**116**	**1.61**	***0*.*08***	**5.37**	***0*.*21***	**20.48**	***4*.*35***	**0.396**	***0*.*022***	**0.577**	***0*.*030***	**0.731**	***0*.*032***
	**A**	15	1.72	*0*.*25*	6.29	*0*.*59*	36.18	*19*.*85*	0.347	*0*.*028*	0.486	*0*.*027*	0.680	*0*.*040*
	**B**	12	2.06	*0*.*28*	5.13	*0*.*69*	1.42	*0*.*17*	0.362	*0*.*039*	0.528	*0*.*044*	0.655	*0*.*051*
	**C**			* *		* *		* *		* *		* *		* *
	**D**	5	2.29	*0*.*49*	4.12	*0*.*82*	1.10	*0*.*25*	0.438	*0*.*074*	0.548	*0*.*058*	0.662	*0*.*063*
	**E**	5	1.84	*0*.*45*	4.64	*1*.*26*	11.00	*9*.*68*	0.294	*0*.*093*	0.449	*0*.*071*	0.559	*0*.*079*
	**F**	37	1.25	*0*.*11*	5.44	*0*.*36*	33.56	*9*.*20*	0.470	*0*.*057*	0.667	*0*.*077*	0.864	*0*.*089*
	**G**	19	1.73	*0*.*19*	4.65	*0*.*57*	16.88	*8*.*72*	0.393	*0*.*046*	0.622	*0*.*088*	0.711	*0*.*049*
	**H**	2	1.99	*0*.*74*	8.45	*0*.*46*	1.18	*0*.*18*	0.439	*0*.*021*	0.656	*0*.*026*	0.707	*0*.*002*
	**I**	21	1.56	*0*.*15*	5.57	*0*.*39*	9.06	*4*.*39*	0.333	*0*.*018*	0.501	*0*.*021*	0.655	*0*.*028*
*R*. *tarandus*	**ALL**	**202**	**2.07**	***0*.*07***	**4.52**	***0*.*14***	**8.08**	***1*.*82***	**0.354**	***0*.*012***	**0.533**	***0*.*016***	**0.673**	***0*.*019***
	**A**	15	2.45	*0*.*38*	5.31	*0*.*64*	32.61	*12*.*09*	0.316	*0*.*040*	0.522	*0*.*049*	0.679	*0*.*059*
	**B**	36	2.24	*0*.*15*	4.19	*0*.*33*	8.07	*4*.*68*	0.310	*0*.*018*	0.492	*0*.*024*	0.625	*0*.*032*
	**C**			* *		* *		* *		* *		* *		* *
	**D**	9	2.73	*0*.*61*	2.77	*0*.*38*	1.92	*0*.*53*	0.271	*0*.*038*	0.448	*0*.*040*	0.582	*0*.*044*
	**E**	9	2.43	*0*.*29*	3.82	*0*.*50*	3.02	*1*.*52*	0.435	*0*.*053*	0.585	*0*.*041*	0.736	*0*.*051*
	**F**	76	1.97	*0*.*12*	4.43	*0*.*21*	7.22	*3*.*22*	0.377	*0*.*021*	0.543	*0*.*029*	0.690	*0*.*031*
	**G**	43	1.83	*0*.*13*	4.87	*0*.*26*	5.36	*1*.*94*	0.377	*0*.*029*	0.560	*0*.*046*	0.692	*0*.*055*
	**H**	5	1.56	*0*.*29*	5.88	*0*.*65*	1.43	*0*.*33*	0.341	*0*.*069*	0.594	*0*.*109*	0.761	*0*.*137*
	**I**	9	2.00	*0*.*30*	5.35	*0*.*60*	2.37	*0*.*56*	0.303	*0*.*041*	0.497	*0*.*060*	0.611	*0*.*081*

### Statistics

We tested both inter- and intra-specific dietary variations through time. Low sample sizes for the three studied ungulates in most archeological layers of Combe-Grenal prevented us from statistically testing for texture variations between them (Fig 2C). In order to counterbalance this issue, the choice has been made to group together Bordes’ layers into archeological “Blocks” that are overall similar in terms of faunal associations and lithic industry prior to statistical analyses [1, 22, 26], (Fig 2). Therefore, block A corresponds to levels 4 to 10 of Bordes (Discoid/Levallois, mixed faunas), Block B to levels 11 to 15 (Discoid, dominated by large ungulates?), Block C to level 16 (no DMTA sample), Block D to levels 17 to 19 (Quina, mostly reindeer), Block E to level 20 (Quina rich in denticulates, mostly reindeer), Block F to levels 21 to 26 (Quina, mostly reindeer), Block G to 27 to 32 (Levallois, mixed faunas), Block H to level 33 (Levallois, large bovids?), Block I to levels 34 to 36 (Levallois, red deer). We performed an alternative analysis exploring statistical differences between three larger time periods defined in previous works [31, 38]: “Ante-Quina” [levels 27–36], Quina [levels 17–26] and “Post-Quina” [levels 4–16].

Statistical analyses were done following Smith & Warren [39] and Zuur et al. [40]. For each ungulate species, we tested the variation of its dental textures through time (Block or Period). Among each time frame considered (Block or Period), we also tested for inter-specific differences in dental textures. Data exploration and statistics were conducted with R v. 4.0.3 and the packages “outliers”[41], “ggplot2” [42], “lawstat” [43],“onewaytests” [44], “MASS” [45], “Hmisc” [46], “corrplot” [47], “regplot” [48], “AICcmodavg” [49], “emmeans” [50], “stats” [51], “lattice” [52], “FSA” [53]. The database and R markdown script are respectively available in S1 and S3 Appendices. After a visual data exploration (notably frequency plots and boxplots), [40] and Brown and Forsythe tests for homogeneity of variance [39] to test the applicability of parametric tests, we performed BoxCox data-transformations whenever needed (among reindeer, for inter-block differences in Smc; among red deer, for inter-block differences in HAsfc 81; for block H, inter-specific differences in epLsar; S3 Appendix). Generalized Linear Models (GLM) were then performed with the objective to select, based on the lower AIC, the model that best explains the distribution of data (“Block”, “Period” or “random”; “species” or “random”; AICs are available in S3 Appendix). Whenever best models showed statistical differences, post hoc Tukey HSD pairwise tests allowed us to identify inter-group significant differences. In the situation where the conditions for applying parametric tests were not satisfied (for Smc when testing for inter-specific differences during the Quina and inter-specific differences among the block F; and inter-period and inter-block differences in epLsar for reindeer, S3 Appendix), a Kruskal-Wallis test followed by a Dunn’s post-hoc test were used to explore the inter-group differences. We did not discuss significant differences between groups represented by less than 5 individuals.

## Results

### 1-Main represented dietary categories

**Fig 3:**
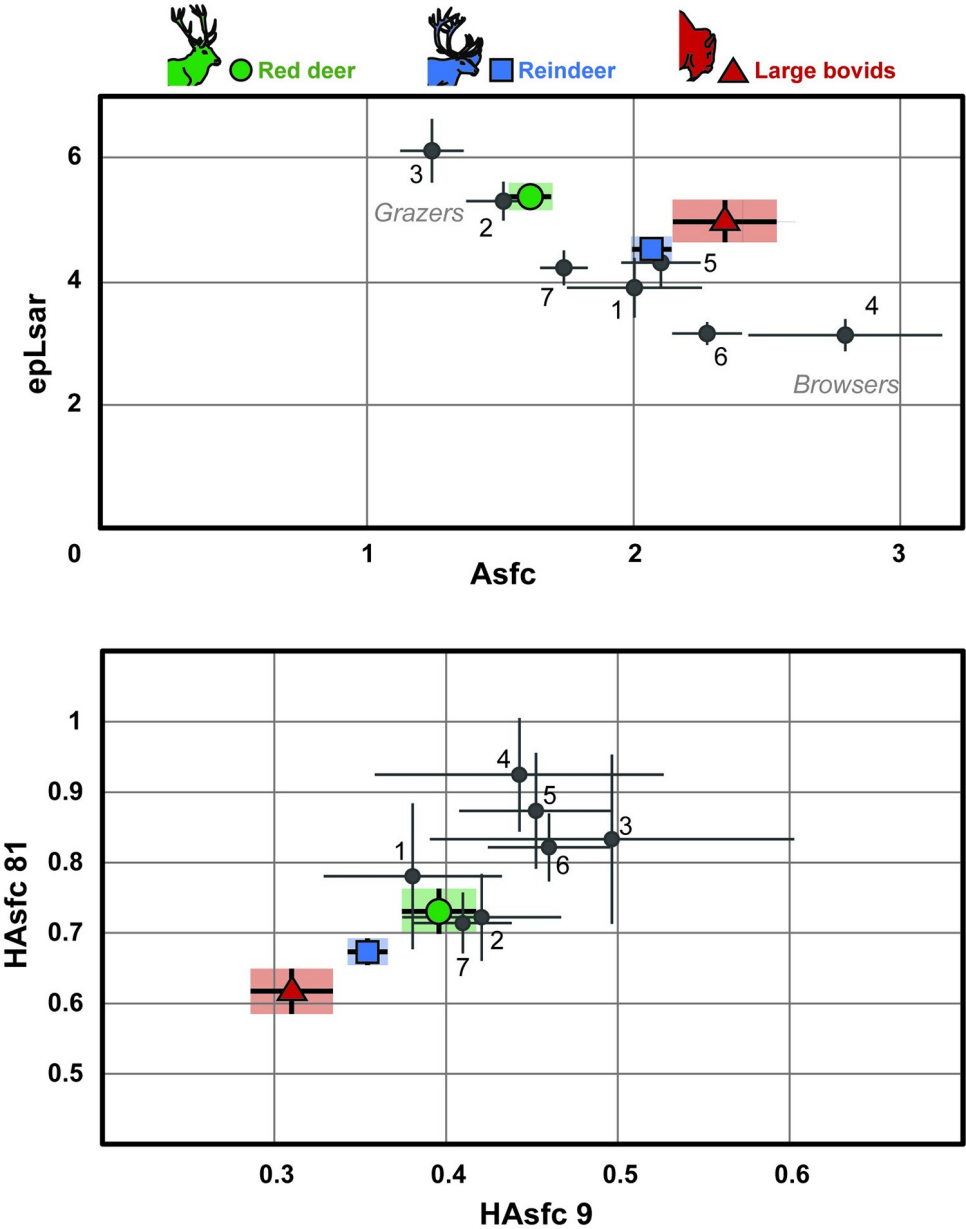
Distribution of the three ungulate taxa from Combe-Grenal (all layers grouped) based on the complexity (Asfc), anisotropy (epLsar) and heterogeneities of the complexity (HAsfc 9 and HAsfc 81) of the dental textures. Each taxon and reference population is represented by its mean and standard error of mean. Green circle: Cervus elaphus; Blue square: Rangifer tarandus; Red triangle: Bison priscus/Bos primigenius. **Reference populations with well-known diet**: 1: Bison bonasus, Białowieża, Poland; 2: Bos taurus, Camargue, France; 3: Cervus elaphus, Bauges, France; 4: Cervus elaphus, Białowieża, Poland; 5: Cervus elaphus, Chateauroux, France; 6: Rangifer tarandus, Hardangervidda, Norway; 7: Rangifer tarandus, Knutshø, Norway.

The three populations of extant ruminants assigned as grazers (lower part of Table 2, Fig 3; populations 2, 3, 7) are characterized by low complexities (*Asfc*) and heterogeneities of the complexity (*HAsfc 9*) coupled with high anisotropies (*epLsar*). Low to intermediate anisotropies (*epLsar*) coupled with middle to high complexities (*Asfc*) and intermediate to high heterogeneities of the complexity (*HAsfc 9*) are typical of the browsing dietary category, represented here by the red deer population from Białowieża primeval forest (Table 2; population 4). The mixed-feeder are represented here by three populations including the red deer sample from Chateauroux (population 5), the population of *Bison bonasus* from Białowieża (population 1) and the *Rangifer tarandus* population from Hardangervidda (population 6). They are characterized by intermediate values of their texture parameters (Fig 3; lower part of Table 2).

Results for fossils are discussed based on the herbivore dietary ecospace defined here on the basis of the seven extant European ungulate populations with well-known diet (Tables 1 and 2, Fig 3; see also [5]). *Bos/Bison* and *Rangifer tarandus* are characterized by medium to high mean *Asfc* and *epLsar* values. Heterogeneity (*HAsfc 9*) is generally low. For *Cervus elaphus*, the *Asfc* varies from low to intermediate values, coupled with medium to high *epLsar* and low *HAsfc 9* in most cases.

### 2-Inter-specific differences between contemporaneous ungulate populations

**Fig 4:**
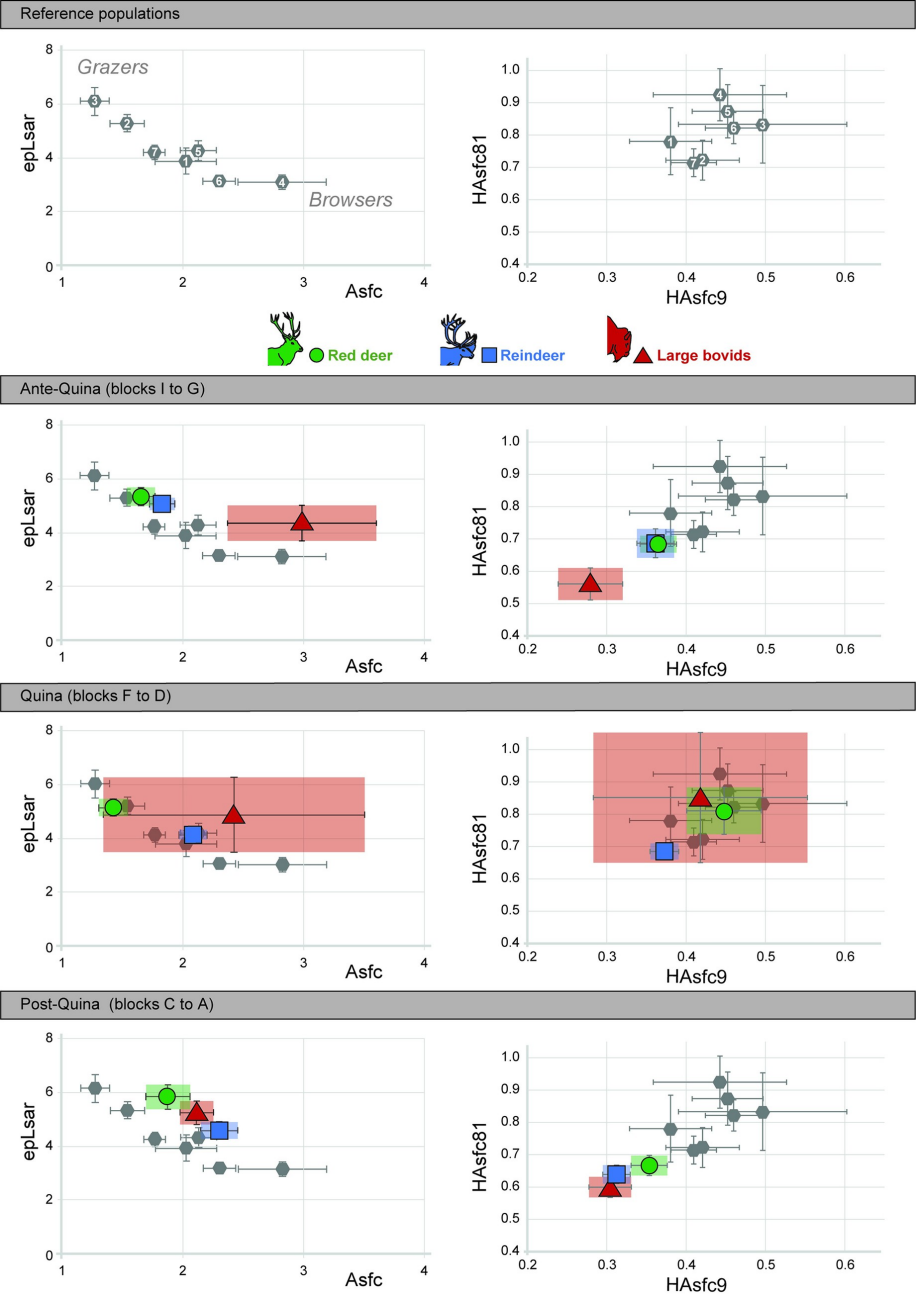
Distribution (mean and standard error of mean) for the three taxa for each period considered in this study, depending on complexity (Asfc) and anisotropy (epLsar (x10^-3^); left) and on heterogeneities of the complexity (HAsfc 9 and HAsfc 81; right). Reference populations are illustrated on the top of the figure and used as reference for the Ante-Quina, Quina and Post-Quina plots. **They are identified as follows:** 1: Bison bonasus, Białowieża, Poland; 2 : Bos taurus, Camargue, France; 3: Cervus elaphus, Bauges, France; 4: Cervus elaphus, Białowieża, Poland; 5: Cervus elaphus, Chateauroux, France; 6: Rangifer tarandus, Hardangervidda, Norway; 7: Rangifer tarandus, Knutshø, Norway. Bos/Bison is represented by red triangles, Cervus elaphus by green circles and Rangifer tarandus by blue squares. **Periods**: Ante-Quina: levels 27 to 36, Quina: levels 17 to 26, Post-Quina: levels 4 to 16.

During both Ante-Quina (*Asfc*, *Smc*), Quina (*Asfc*, *epLsar*, *Smc*, *HAsfc 81*) and Post-Quina (*epLsar*, *Smc*), the models based on the “species” variable explain better the distribution than the “random” models (lower AIC; S3 Appendix). Post-hoc tests allowed us to identify significant differences (p-value <0.05) between bovids and cervids during the Ante-Quina (bovids have higher *Asfc* during this period; Table 2; Fig 4, S1 and S3 Appendices) and between red deer and reindeer during the Quina (*Asfc* is lower while *epLsar* and *Smc* are higher for red deer during this period; Table 2; Fig 4, S1 and S3 Appendices).

**Fig 5:**
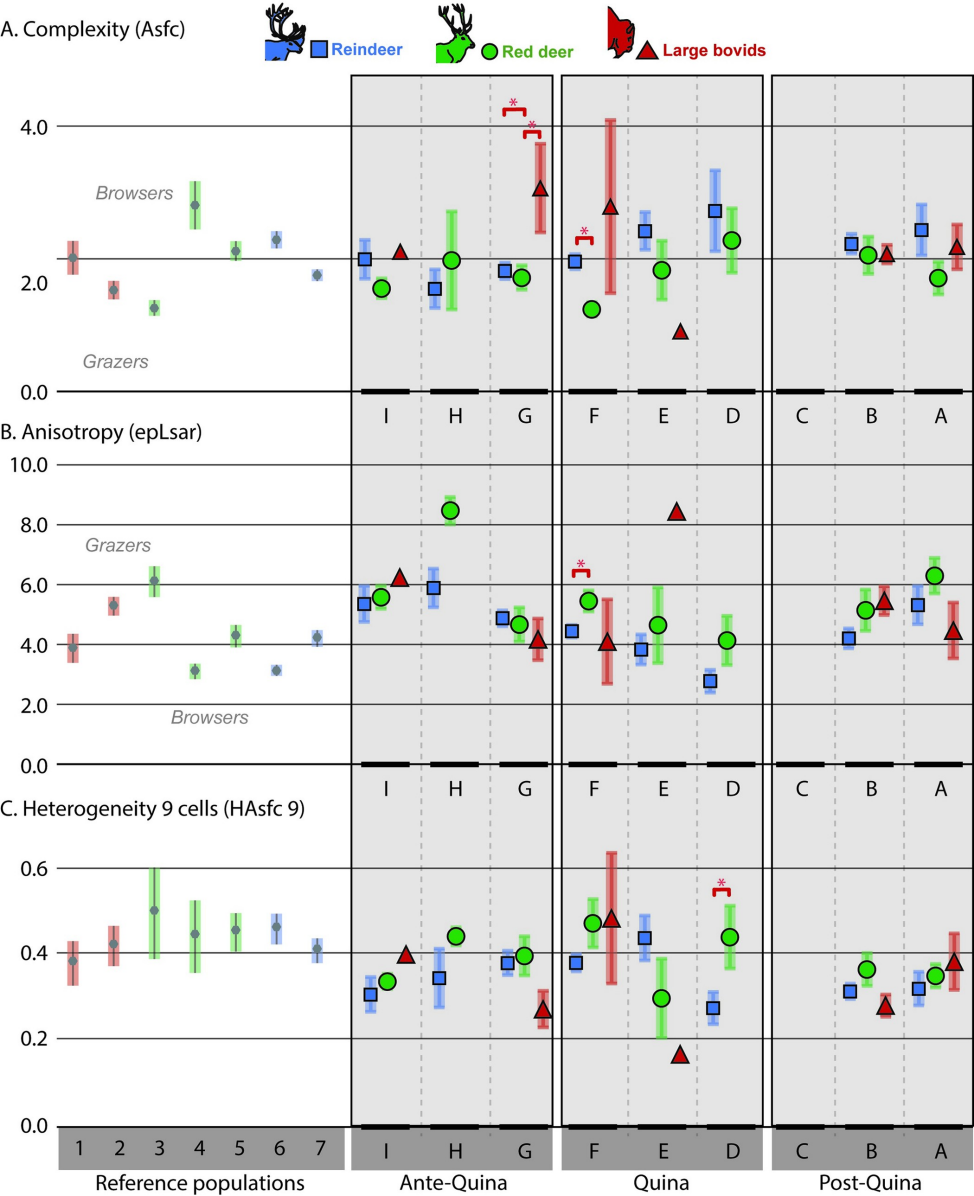
Barplots representing the mean and standard error of mean for Blocks A to I of Combe-Grenal and for extant European ungulate reference populations with well-known diet. **Reference populations**: 1: Bison bonasus, Białowieża, Poland; 2 : Bos taurus, Camargue, France; 3: Cervus elaphus, Bauges, France; 4: Cervus elaphus, Białowieża, Poland; 5: Cervus elaphus, Chateauroux, France; 6: Rangifer tarandus, Hardangervidda, Norway; 7: Rangifer tarandus, Knutshø, Norway. **Temporal Blocks:** Block A: levels 4 to 10, Block B: levels 11 to 15, Block C: level 16, Block D: levels 17 to 19, Block E: level 20, Block F: levels 21 to 26, Block G: 27 to 32, Block H: level 33, Block I: levels 34 to 36. **Periods**: Ante-Quina: levels 27 to 36, Quina: levels 17 to 26, Post-Quina: levels 4 to 16. Significant differences (see also S3 Appendix) are illustrated by a red “*”, for groups with N≥5 individuals.

In Blocks B (*epLsar*), D (*HAsfc 9*), E (HAsfc 36 and HAsfc 81), F (*Asfc*, *epLsar*, *Smc*, *HAsfc 81*) and G (*Asfc* and *Smc*) the variable “species” is more efficient than random to explain the distribution (smaller AIC, S3 Appendix). As identified with post-hoc tests, interspecific differences are located between red deer and reindeer in Block D (*HAsfc 9* is higher for red deer; Table 3) and Block F (epLsar and Smc are higher and Asfc lower for red deer; N<5 for Bovidae); Table 3; Fig 5, S1 and S3 Appendices). In Block G, *Cervus elaphus* differs in *Asfc* from the two other taxa (Table 3; Fig 5, S1 and S3 Appendices).

### 3-Intra-specific dietary variations through time

For almost all texture parameters and for the three taxa, as tested with the GLM models, data better fits a random distribution than a distribution explained by Blocks (S3 Appendix). Based on GLM (S3 Appendix), the periods better explain the distribution for *Asfc (Cervus elaphus*, *Rangifer tarandus)*, *epLsar (Rangifer tarandus)*, *Smc (Bos/Bison)*, *HAsfc 9 (Rangifer tarandus)*, *HAsfc 36 and HAsfc 81 (Bos/Bison)*. However, only few of these differences are supported by post-hoc tests. Bovids present higher HAsfc 81 values during the Quina (Table 2, Fig 4, S1 and S3 Appendices). The Kruskal-Wallis analysis followed by a Dunn post-hoc test testing for inter-Period differences for the *epLsar* of *Rangifer tarandus* support a significant difference between Ante-Quina and Quina (Table 2; Fig 4, S1 and S3 Appendices). Although not significant, we also observed a tendency toward an increase of *Asfc* and a decrease of *epLsar* through time for both cervids from Block H to Block D.

## Discussion

### Ungulates from Combe-Grenal: Paleoecology

When compared with the values for the seven reference populations categorizing browsing to grazing dietary categories (Table 1; Fig 3), the dental textures of the 116 *Cervus elaphus* from Combe-Grenal reflect a grazing diet, a feeding behavior that is well-known for extant relatives of this species living in open habitats [54, 55]. The extant red deer is indeed a polymorphic species. Its body mass variations range from one to five from one population to another, a particularity that has to be linked with the diversity of the habitats that are occupied by the species [56]. Through Eurasia, the red deer is indeed currently present from Southern Spain to Northern Norway and from the Atlantic coast of Western Europe to the Caspian sea in a large variety of habitats [57]. As shown by Gebert and Verheyden-Tixier [54] the main driver of its interpopulation dietary variations is the characteristics of its habitat, which makes of the feeding ecology of this cervid a very appropriate proxy for the paleoenvironmental reconstructions in archeological sites [5, 58].

The wild reindeer is nowadays probably the most dietary plastic of European deer, its ecology and body size being dependent on the characteristics of its habitat [56, 59, 60]. Its current distribution is circumpolar [56, 61]. It can tolerate extreme cold and its morphology facilitates its movements in snow [62, 63]. This deer can be found in both mountainous environments and oceanic coasts, in the tundra as in the taiga. The reindeer consumes what is available in its environment according to the seasonal plant phenology [56, 59, 64, 65]. As it is able to consume a resource until it is depleted, it also plays an important role in shaping the vegetation and landscapes [66–68]. When these resources are available, reindeer favors food with a high nutritional value. However, this species is very opportunistic, giving to its feeding behavior a potential interest for paleoenvironmental reconstructions. Knowing this, the mixed-feeding diet of the 202 *Rangifer tarandus* from Combe-Grenal witnesses the availability of a large amount of herbaceous monocotyledons, associated with a significant part of ligneous material, in the vicinity of Combe-Grenal.

The steppe bison *Bison priscus* is a large bovid which taxonomic relationships remain under debate [69–71 and references therein]. This species was one major element of the “mammoth steppe” mammalian assemblage [72, 73] and one of the key prey for European human populations in numerous fossil localities throughout Eurasia [74]. Its vast distribution, from the early middle Pleistocene to the early Holocene (that is to say between 0.781 and 0,0117 Ma), covered Eurasia and North America [72, 75]. During the last glaciation at the Pleistocene-Holocene transition, the species abundance drastically decreased to the point of its extinction. Climate and anthropogenic impacts are among the factors considered responsible for its extinction [75, 76]. The diversity of habitats (from cold and opened steppes to more forested habitats), of possible feeding behaviors [including both herbaceous monocotyledons and lichens: 77, 78] and of mobility behaviors of *Bison priscus*, capable of both migration and sedentarity [74], reflects the important ecological plasticity of the species. The aurochs (*Bos primigenius*) is generally considered as the wild ancestor of *Bos taurus*, the domestic cattle [79 and references therein]. The first occurrences of aurochs date back to the Pleistocene [between 2–1.5 Ma; 81]. Its geographical range included northern Africa and almost the whole Eurasia [80]. Overhunting and suitable habitat reduction have undoubtedly played a major role in their drop until their extinction, that most likely occurred in Poland during the 17^th^ century [80]. While several studies highlight the preference of *Bos primigenius* for forested habitats under mild and humid climatic conditions [80–82 and references therein], the feeding ecology of this bovid suggests a degree of dietary plasticity that allows it to adapt its diet over time in response to environmental changes [83]. The 50 Bovinae from Combe-Grenal are mixed feeders. Here again, such results support the occurrence of an abundant herbaceous monocotyledon layer, coupled with ligneous material.

Inside each of the temporal Blocks, significant inter-specific dietary differences (considered for N≥5 individuals only; Fig 5) are scarce, except during the Ante-Quina (Block G) between *Cervus elaphus* and the other taxa and the Quina (blocks D and F) when *Cervus elaphus* and *Rangifer tarandus* diets statistically differ. Before the Quina, both cervids are more engaged in grazing while bovids present dental textures reflecting the ingestion of a large amount of ligneous material, similar to the reference population of *Cervus elaphus* from Białowieża primeval forest (Fig 5). Block F reflects the difference between the grazing diet of *Cervus elaphus* and the mixed-feeding diet of *Rangifer tarandus*. The heterogeneity of the complexity (*HAsfc 9*) is generally interpreted as reflecting the diversity of the ingested food items [12, 13]. In Block D, *HAsfc 9* is lower for *Rangifer tarandus*.

Similar dental textures in most cases for the four contemporaneous and sympatric ungulate populations indicate no obvious niche-partitioning between these large herbivores.

### Comparison with previous paleoenvironmental interpretations

Considering the well-known, previously discussed, ecological plasticity of the four ungulates investigated, able to adapt their feeding ecology to changes in resource availability in their habitat, we could expect DMTA analysis of these taxa to track environmental changes known for the region from the MIS5 to the MIS3 (Fig 1). However, in contradiction to this hypothetical proposition, the dental textures do not reflect clear variations in the feeding ecology of the four ungulates from Combe-Grenal through time. This archeological site has however frequently been cited as an example of a sequence that document marked environmental changes during the Middle Palaeolithic. Previously acquired paleoenvironmental data can be summarized as follows:

- Clear changes in the climate and vegetation have been documented in Western France from MIS 5 to MIS 3 [27–30], (Fig 1), but the environmental conditions in the immediate vicinity of Combe-Grenal are largely unknown.
- Changes in the proportions of hunted ungulates at Combe-Grenal [20, 21] have however been shown to track regional vegetation changes known for the period [1, 31].
- Data on rodent species are scarce, but highlight a slight diachronic pattern, notably the presence of the woody *Microtus (Terricola) subterraneus* during the “Ante-Quina” (including in layer 31) but not in the following Quina and “Post-Quina” periods [1, 84].
- Isotopic analyses of *Equus* teeth from layers 30 to 4, that are contemporaneous to the ungulates analysed in the present study, were interpreted by the authors as evidencing marked changes in temperature and aridity throughout the sequence [23]. In particular, they highlighted values in dentine δ^13^C during our period of interest that are in favour of an open environment, and ample variations in both dentine δ^15^N and enamel phosphate δ^18^O through time [23]. For several layers of interest to our study [4, 9, 16, 20, 21, 32, 33, 35] isotope results were however unavailable in the study of Richards et al. [23].

We observe for the two deer a global tendency toward a decrease in the ingestion of abrasive herbaceous monocotyledons from blocks H to D (layers 33 to 17). This decrease is supported by both the progressive increase of complexity (*Asfc*; Fig 5A; Table 3) and a decrease of anisotropy (*epLsar*; Fig 5B; Table 3). For reindeer, this decrease in anisotropy is supported at a larger time scale by the significant difference between Ante-Quina and Quina (p-value <0.05; Fig 4). The fact that this trend occurs for both cervids reinforces its relevance, despite being only “partially significant” (not significant at the thinner scale of blocks, but significant between Ante-Quina and Quina for reindeer). Despite previously mentioned paleoclimatic variations, paleoenvironmental changes and faunal turnovers (Figs 1 and 2), our DMTA results imply that the same types of resources were available and consumed by the four preyed ungulates throughout the sequence, at least during the last days or weeks prior to their death.

These seemingly contradictory findings are the result of a combination of the following two factors: (i) from a technical perspective, the apparent discrepancy between paleoenvironmental data acquired by DMTA analysis and from other proxies including isotopic analyses reflect the different periods of life and time scales that these approaches document [85]. While most other analyses document the feeding ecology, climatic and environmental conditions experienced by animal populations during few months to few years, DMTA on the other hand is an open window over the feeding preferences and resource availability for the animals shortly before their death ([15], the last few days or weeks, also referred as the « last supper effect »; [16, 17]). (ii) from an archaeological perspective, DMTA results might be explained, for a large part, by the impact of past hunting practices by Neanderthal groups on the sampled ungulate individuals. This latter aspect is further developed in the next section.

### Neanderthal subsistence strategies and habitat uses

The DMTA of ecologically plastic herbivores constitutes an efficient proxy for paleoenvironmental reconstructions [5, 58]. Based on this assumption and on Combe-Grenal ungulate textures (Fig 3), it is possible to infer that *Cervus elaphus*, *Rangifer tarandus* and *Bos/Bison* from Combe-Grenal were, at the time when they were killed, occupying an open, tundra-like habitat, characterized by the predominance of an herbaceous layer associated with shrubs that explain the grazing diet of *Cervus elaphus* and the mixed-feeding diet of both bovids and reindeer. No major change in the resource availability in the habitat(s) where these animals have been hunted, as witnessed by the absence of significant dental texture variations through time for the three ungulate taxa (Fig 5), is in favor of a certain habitat and dietary homogeneity throughout millennia. As these dental textures belong to animals that were hunted by successive Neanderthal groups that occupied Combe-Grenal rock-shelter, they likely represent a non-random sample of the wild population(s) from which they were selected, potentially biased in the direction of particular hunting seasons or preferential hunting territories. By selectively hunting in particular landscapes of their territory, hunters can thus be seen as a “buffering” agent of the paleoenvironmental signal. Tracking and killing ungulates in open settings was probably easier for Neanderthal hunters, and this preference for open settings potentially made environmental changes invisible in a DMTA analysis of archaeofaunas. Under these conditions, DMTA provide us with a very important information: the continuous occurrence, throughout the sequence of Combe-Grenal, of grassland areas that were sufficiently extensive to constitute a hunting ground for Neanderthal hunters and a major part of the diet of the hunted animals shortly before their death. However, these results do not document changes, expected as a consequence of climatic changes, in the location and/or variation in the extent of these grassland areas in the hunting territories exploited by Neanderthal groups over time. This potential “buffering” effect is undoubtedly an element to be taken into account when aiming to infer the palaeoenvironmental changes occurring throughout an archeological sequence using DMTA.

The regional climatic changes through time, notably the progressive habitat opening and rapid climatic changes of MIS 4–3 induced rapid cycles of forest cover expansion and contraction, that impacted the food resources that were available for large ungulates [27–30], (Fig 1). The modification towards peri-optimal habitats might have forced ecological adaptations for several ungulate species, then followed by modifications of their range of distribution, that are reflected in the archeological record by changes in faunal associations. In this context, Neanderthal hunting strategies seem to involve the use of open landscapes over the entire sequence, despite clear paleo-environmental changes in the region. This is how we interpret the homogeneous DMTA results that we obtain, despite varying proportions of large ungulates across the sequence. It is the combination of ungulate ecological plasticity and hunting selection by Neanderthal groups that produces such an apparent “discrepancy” between proxies (e.g. proportions of hunted ungulates on one hand, DMTA data on the other). MIS 5 to 3 paleoenvironmental changes (such as decreasing tree cover and temperature, cf. Fig 1) might have induced demographic changes in herbivore populations, yet some of these ungulates persisted in open grasslands and were preferentially targeted by Neanderthal hunters.

Alternatively, the possibility of a specific hunting of ungulates at a given season during which a large proportion of herbaceous monocotyledons was consumed also remains to be explored. Data on season of death for cervids and bovids in layers 4–36 of Combe-Grenal is extremely scarce: an analysis of tooth eruption sequences and wear allowed Guadelli [21; p. 453–457] to propose reindeer hunting throughout the year in layers 22 and 23. Steele [86] analysis of red deer teeth did not provide detailed information on layers 1 to 36 due to small sample size. Finally, Binford [19] data is not available by layer and insufficiently detailed, making its integration difficult. Acquiring further seasonal data would contribute to our understanding of Neanderthal hunting strategies by understanding whether these animals were culled by Neanderthal populations during seasonal hunts or over several seasons. This aspect needs supplementary investigation and will benefit from the comparison of our DMTA results with those of cementum analyses [see for example 87], currently underway in the framework of the ANR DeerPal project. Acquiring DMTA data from lower parts of the sequence (below layer 36) would also be of interest to test if hunted *Cervus elaphus* always presented a grazing diet at Combe-Grenal.

## Conclusion

In this study, DMTA analysis of preyed *Cervus elaphus*, *Rangifer tarandus* and *Bos/Bison* from Combe-Grenal, that reflects ungulate dietary preferences shortly before their death, support the fact that these animals were, at the time when they were killed, occupying an open, tundra-like habitat. Despite evidence for climatic and environmental changes in the vicinity of the site from MIS 5 to MIS 3, dental textures show relatively little variation through time. This apparent discrepancy between environmental changes and the feeding ecology of these dietary plastic ungulates highlights the continuous preference of Combe-Grenal Neanderthals for hunting in open landscapes, and the persistence of grassland areas in Combe-Grenal vicinity that were sufficiently extensive to constitute the principal hunting ground for Neanderthal hunters all along the sequence. Exploring the feeding ecology of preyed ungulates from other contemporaneous archaeological sites will allow us to decipher if these findings can be extrapolated at the more regional scale of southwestern France or if they reflect specific hunting strategies in the territories used by Neanderthal populations in the vicinity of Combe-Grenal.

DMTA permits to go further in our understanding of human-environment interactions: even when there are ample evidences for paleoenvironmental changes, zooarchaeologists still have to test whether or not these changes drastically altered hunting ground use or not. In the case of Combe-Grenal, it might be the case that they did not. The three taxa studied here, in addition to horses, formed most of Neanderthal subsistence for all of Combe-Grenal layers 36 to 4. DMTA analysis identifies only “open-living” individuals. This means that Combe-Grenal hunters “stayed in the open”, and were never forced to switch to hunting tactics adapted to close encounters in forested environments. As such, they never had, for example, to alter their lithic implements or mobility strategies for such use. Any discussion on the influence of paleoenvironmental change on material culture or human history should include such a precise understanding of local environmental conditions if misinterpretations are to be avoided.

## Supporting information

S1 Appendix Individual information and values for texture parameters for all Combe-Grenal specimens. Ref: reference for each specimen; c: layer; lm: lower molar; dex: right; sin: left; bd: bucco-distal facet; bm: bucco-mesial facet.
(XLSX)

S2 Appendix Photosimulations and false color elevation maps of molar facets of Combe-Grenal Ungulates, scanned by E. Berlioz and E. Capdepon with the white light confocal microscope Leica DCM8 of TRACES Lab, Toulouse. (PDF)

S3 Appendix R markdown script including results, used to explore and analyze the dataset of the present study. (PDF)
