# Supplementary material for: A long-term perspective on Neanderthal environment and subsistence: Insights from the dental microwear texture analysis of hunted ungulates at Combe-Grenal (Dordogne, France)
Source: PLoS One. 2023 Jan 18;18(1):e0278395. doi: 10.1371/journal.pone.0278395 (PMC9847971; doi:10.1371/journal.pone.0278395)
Supplement: S2 Appendix — (PDF) [file pone.0278395.s002.pdf]

"A long-term perspective on Neandertal environment and subsistence: insights from the dental microwear texture analysis of hunted ungulates at Combe-Grenal (Dordogne, France)"

authors: Berlioz, E.; Capdepon, E.; Discamps, E.

Appendice 2:
surfaces scanned by E. Berlioz and E. Capdepon, pre-treatement by E. Berlioz and E. Capdepon, validation by E. Berlioz (2019)

***Rangifer tarandus* - Block A**

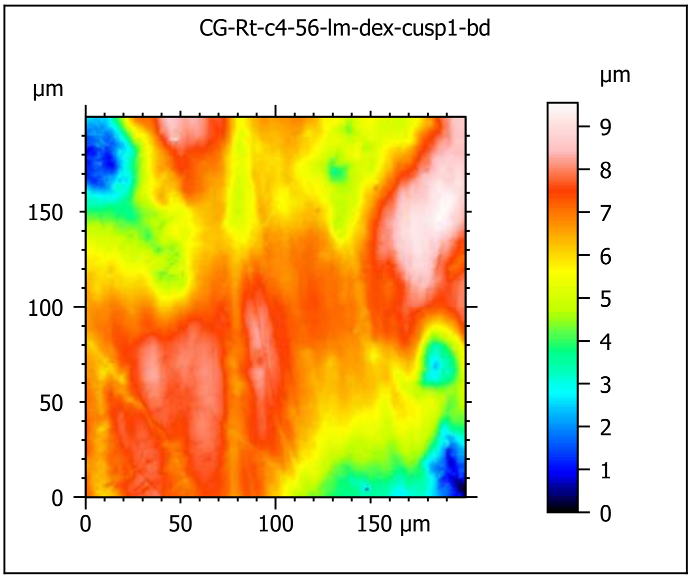

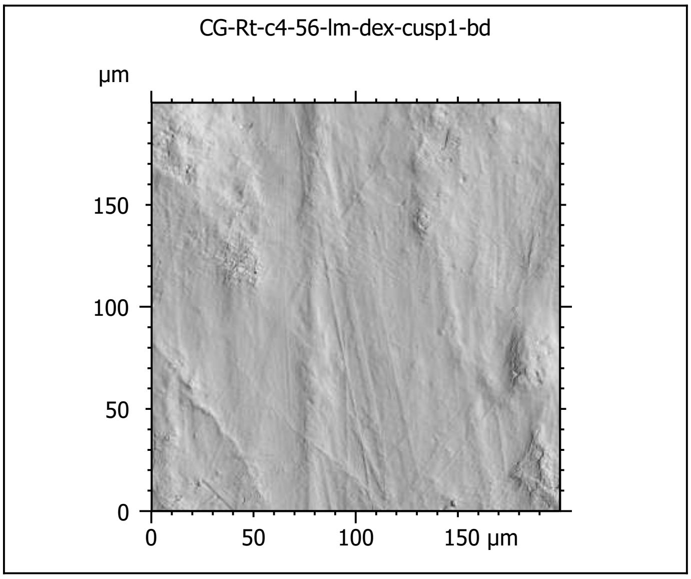

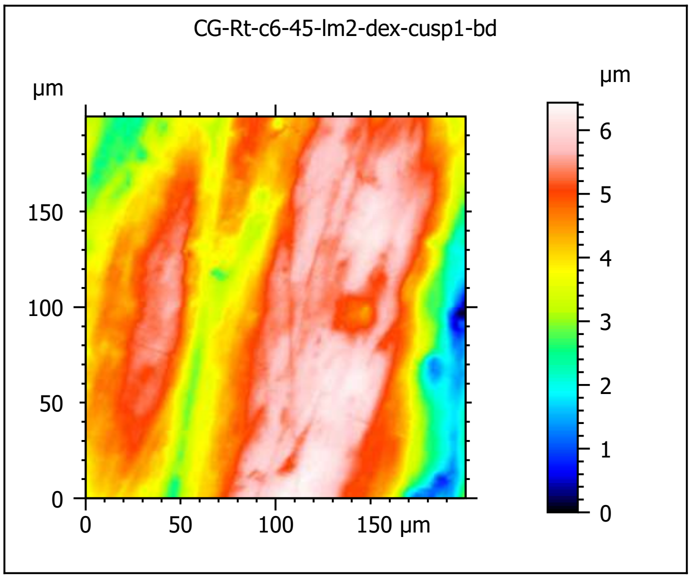

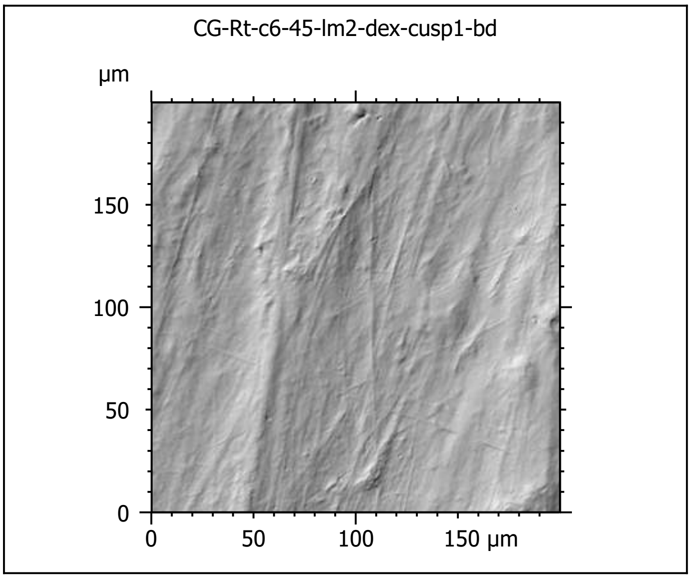

Leica Map Premium 8.2.9843

CG-Rt-c6-46-lm3-sin-cusp1-bd

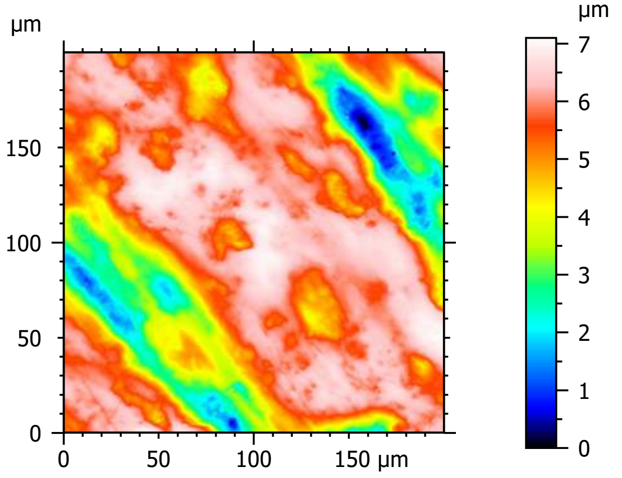

CG-Rt-c6-46-lm3-sin-cusp1-bd

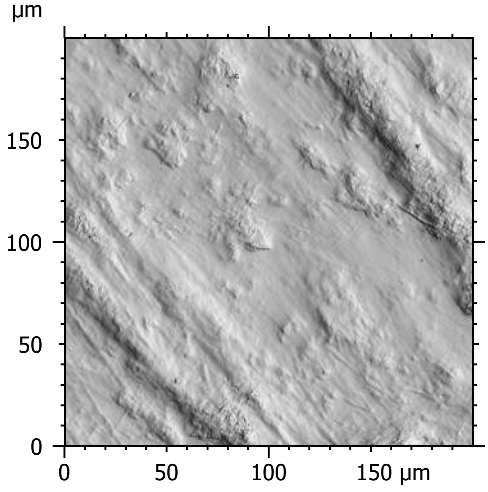

CG-Rt-c6-47-lm3-dex-cusp1-bd

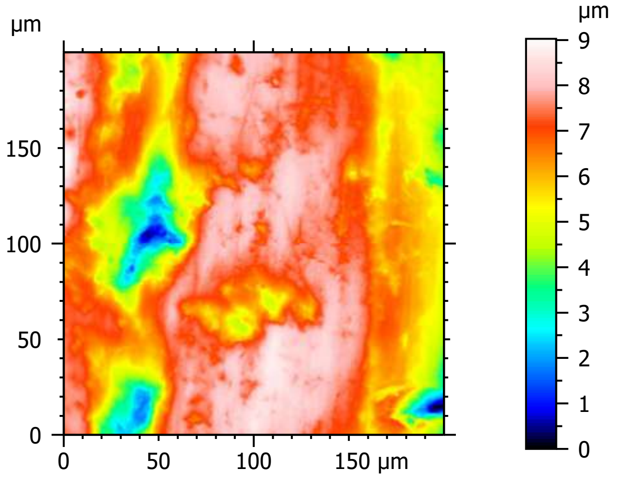

CG-Rt-c6-47-lm3-dex-cusp1-bd

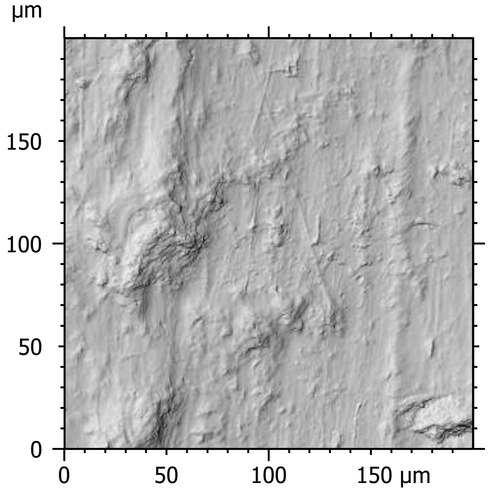

CG-Rt-c7-32-lm2-sin-cusp1-bd

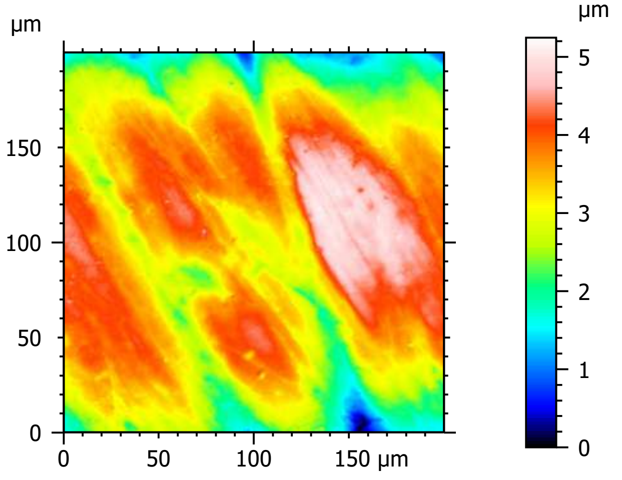

CG-Rt-c7-32-lm2-sin-cusp1-bd

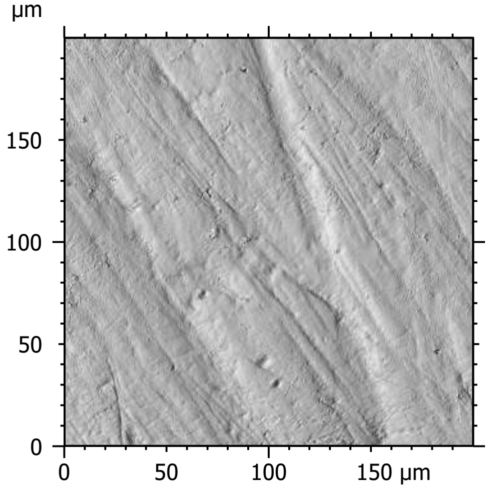

Leica Map Premium 8.2.9843

CG-Rt-c7-33-lm3-sin-cusp1-bd

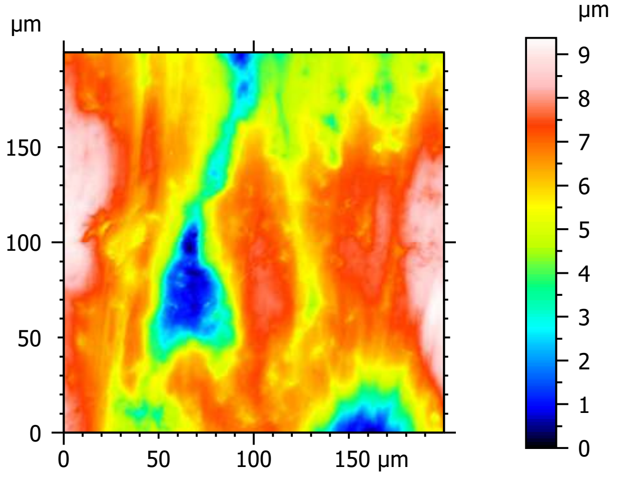

CG-Rt-c7-33-lm3-sin-cusp1-bd

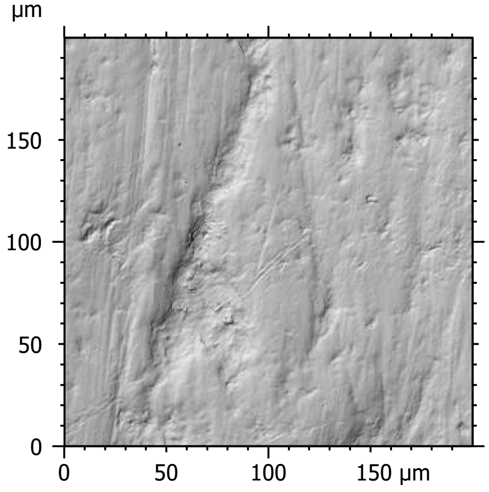

CG-Rt-c7-34-lm2-dex-cusp1-bd

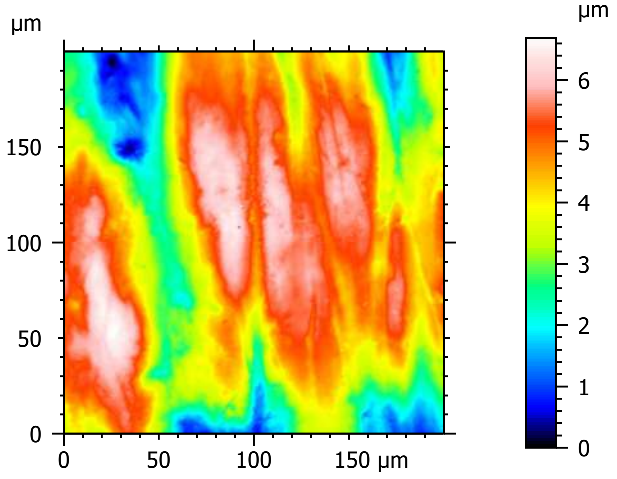

CG-Rt-c7-34-lm2-dex-cusp1-bd

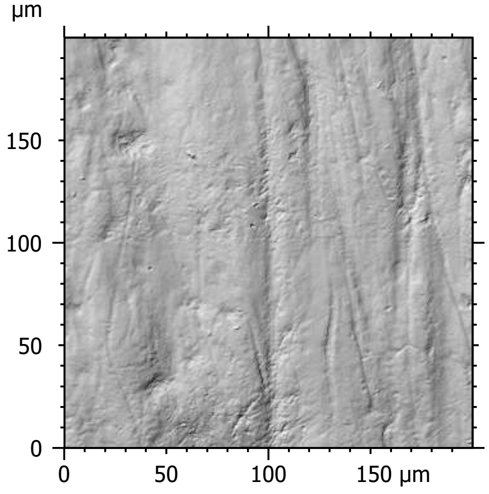

CG-Rt-c7-36-lm2-sin-cusp1-bd

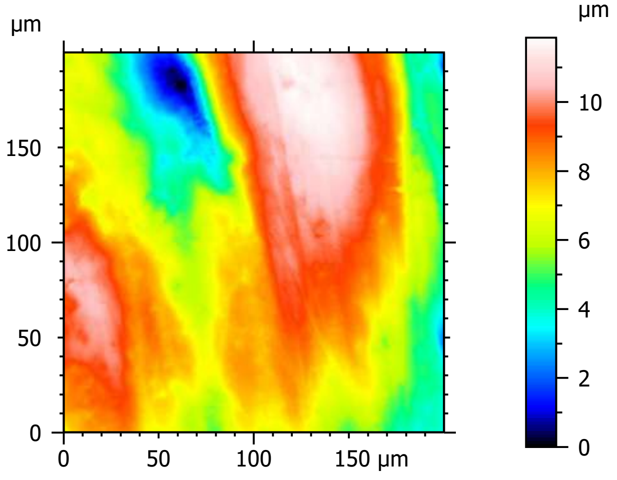

CG-Rt-c7-36-lm2-sin-cusp1-bd

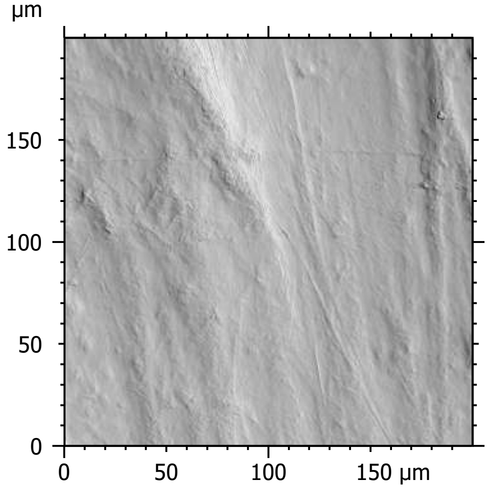

Leica Map Premium 8.2.9843

CG-Rt-c7-37-lm2-dex-cusp1-bd

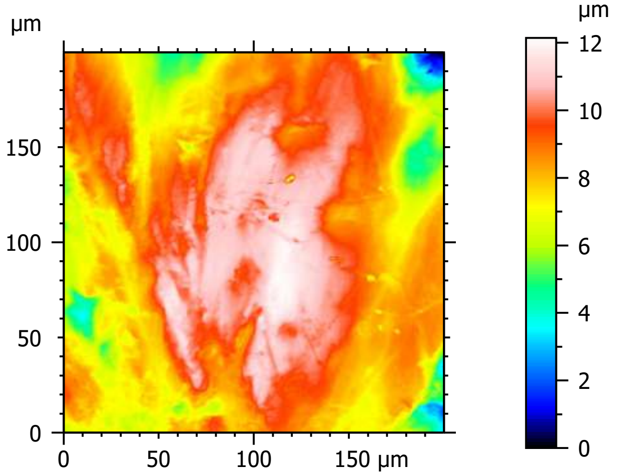

CG-Rt-c7-37-lm2-dex-cusp1-bd

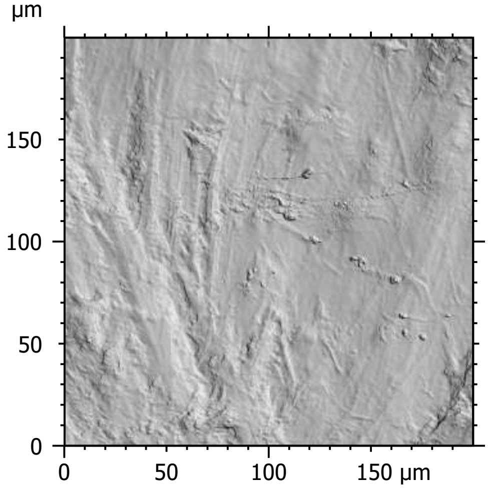

CG-Rt-c7-38-lm2-dex-cusp1-bd

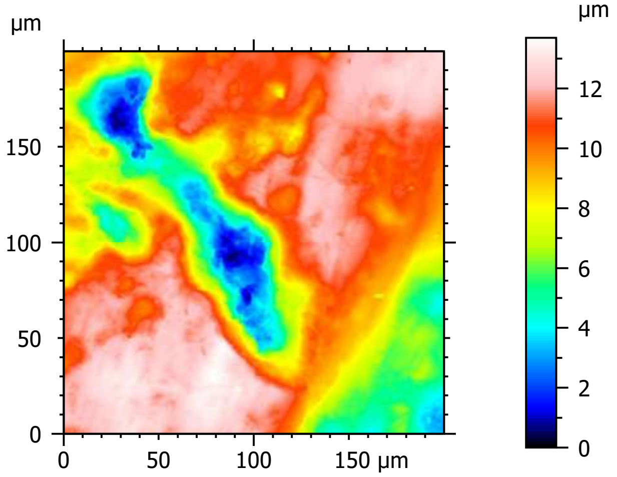

CG-Rt-c7-38-lm2-dex-cusp1-bd

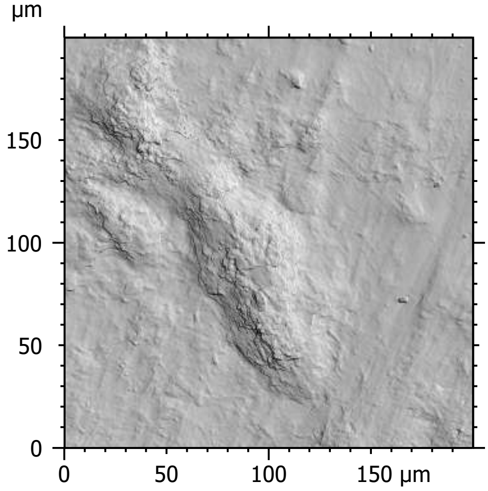

CG-Rt-c7-39-lm2-dex-cusp1-bd

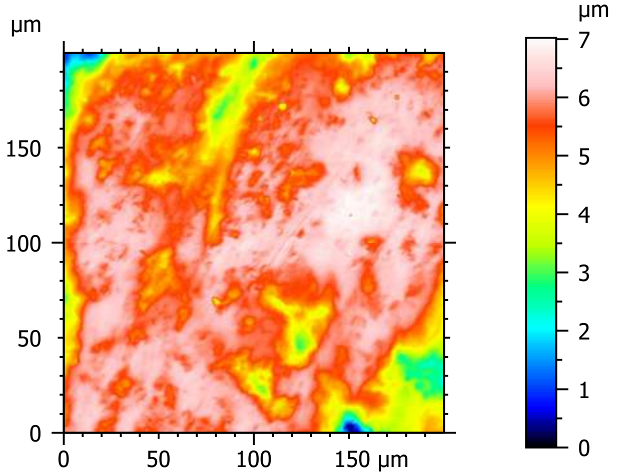

CG-Rt-c7-39-lm2-dex-cusp1-bd

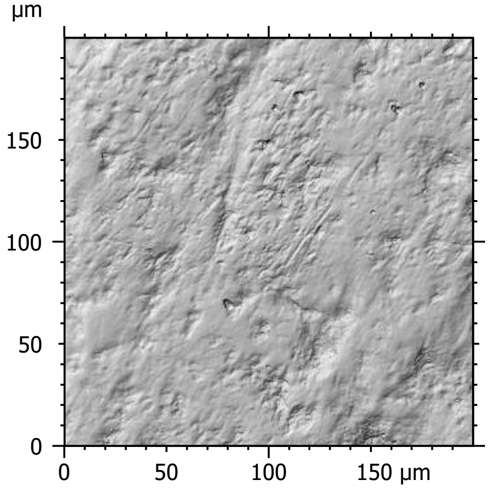

Leica Map Premium 8.2.9843

CG-Rt-c7-no_nb-lm2-sin-cusp1-bd

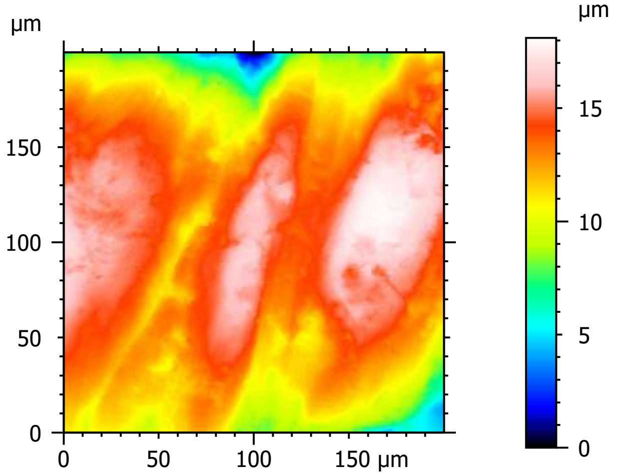

CG-Rt-c7-no_nb-lm2-sin-cusp1-bd

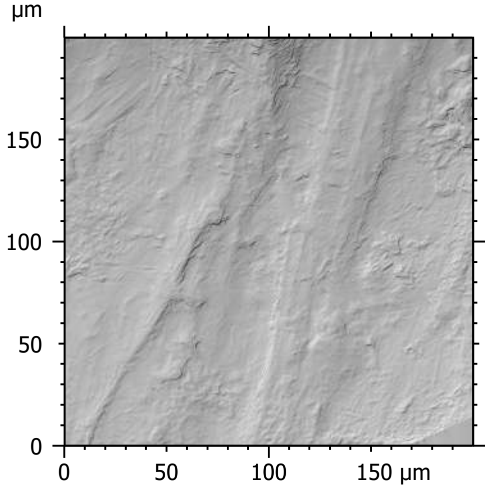

CG-Rt-c8-29-lm2-dex-cusp1-bd

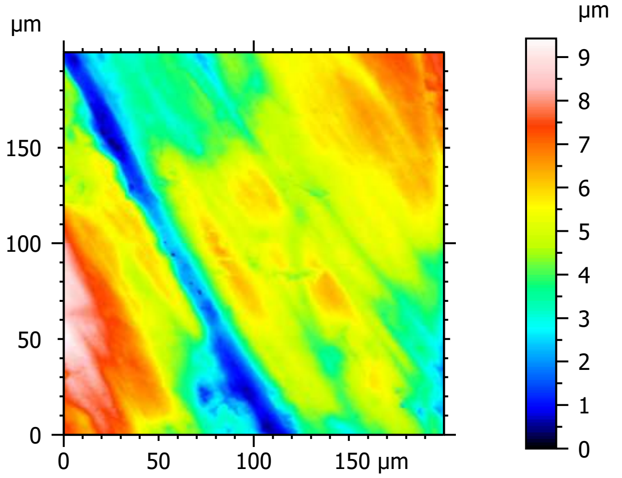

CG-Rt-c8-29-lm2-dex-cusp1-bd

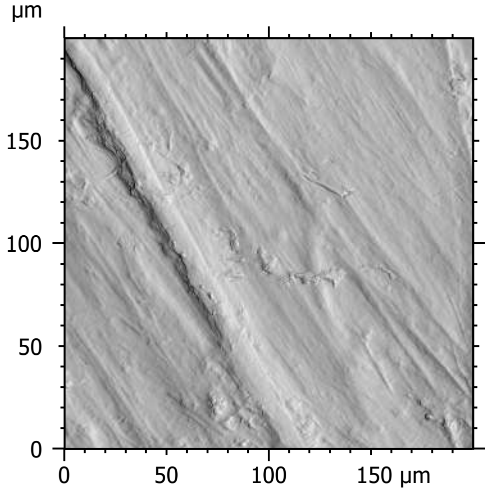

CG-Rt-c8-30-lm3-sin-cusp2-bd

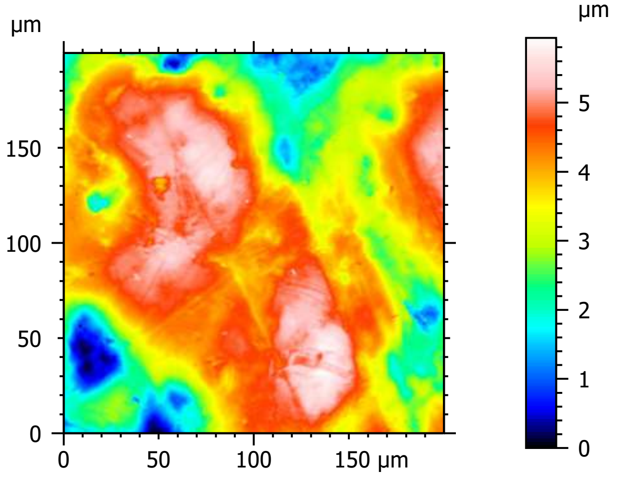

CG-Rt-c8-30-lm3-sin-cusp2-bd

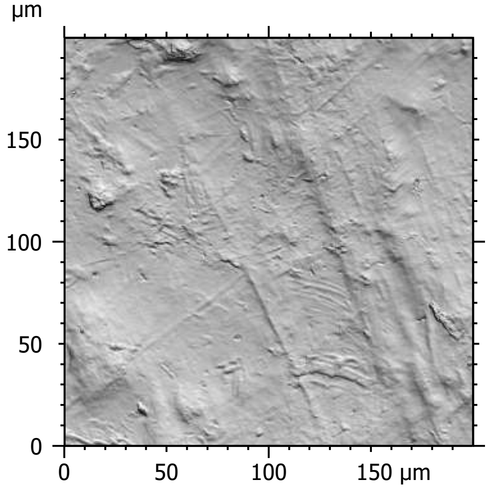

Leica Map Premium 8.2.9843

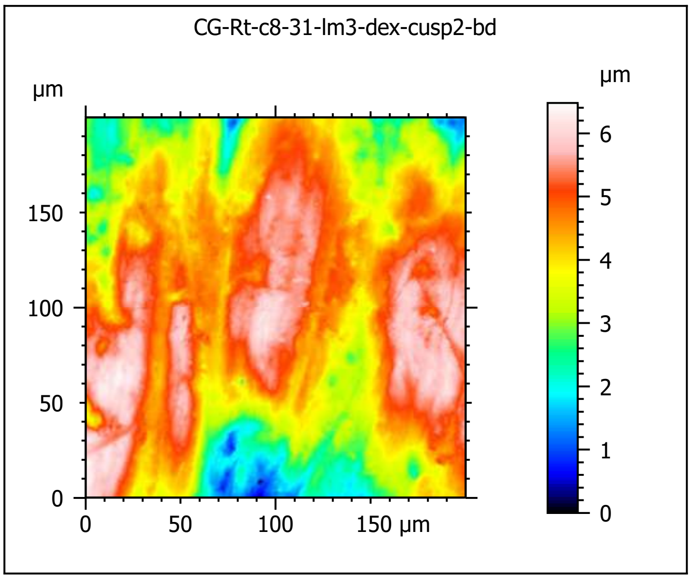

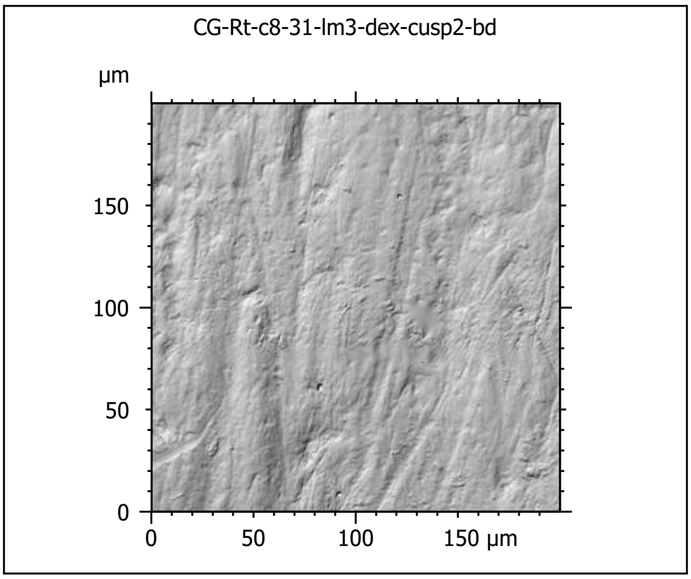

Leica Map Premium 8.2.9843

"A long-term perspective on Neandertal environment and subsistence: insights from the dental micro-texture analysis of hunted ungulates at Combe-Grenal (Dordogne, France)"

authors: Berlioz, E.; Capdepon, E.; Discamps, E.

Appendice 2:
surfaces scanned by E. Berlioz and E. Capdepon, pre-treatement by E. Berlioz and E. Capdepon, validation by E. Berlioz (2019)

***Rangifer tarandus* - Block B**

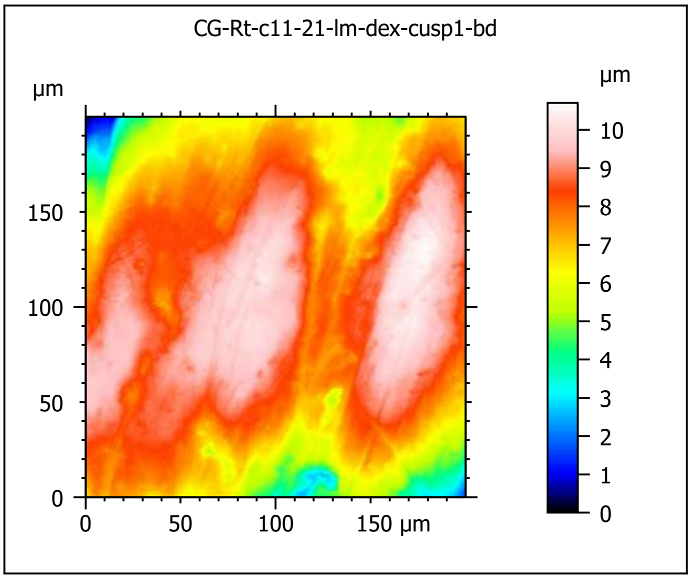

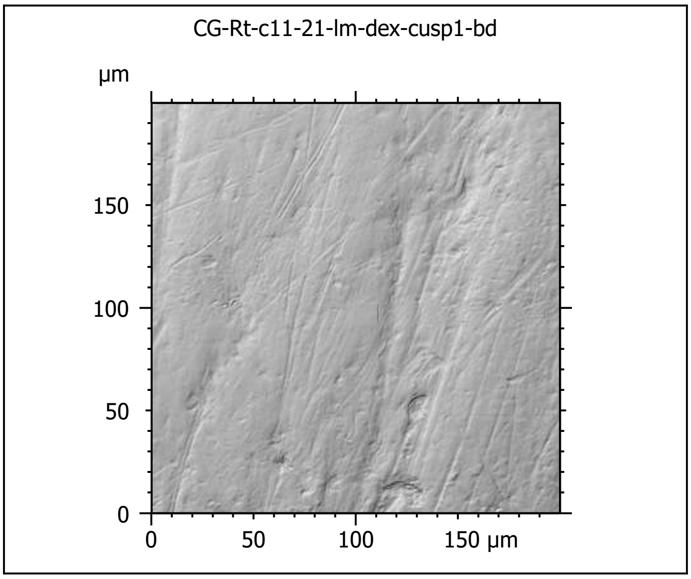

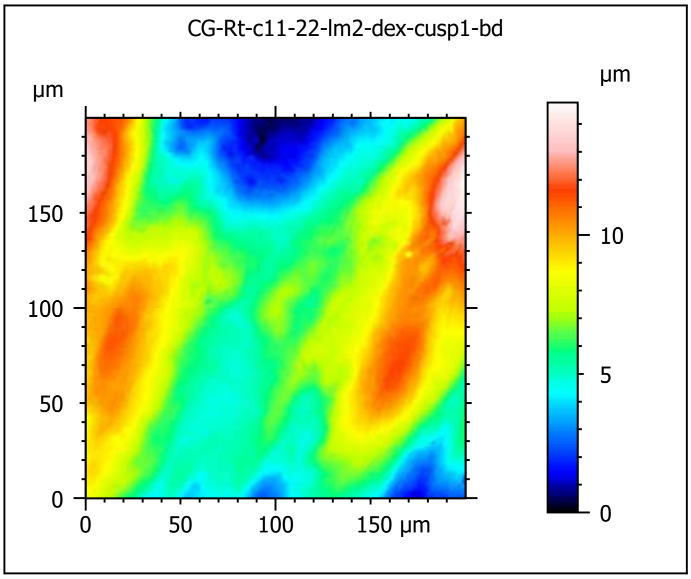

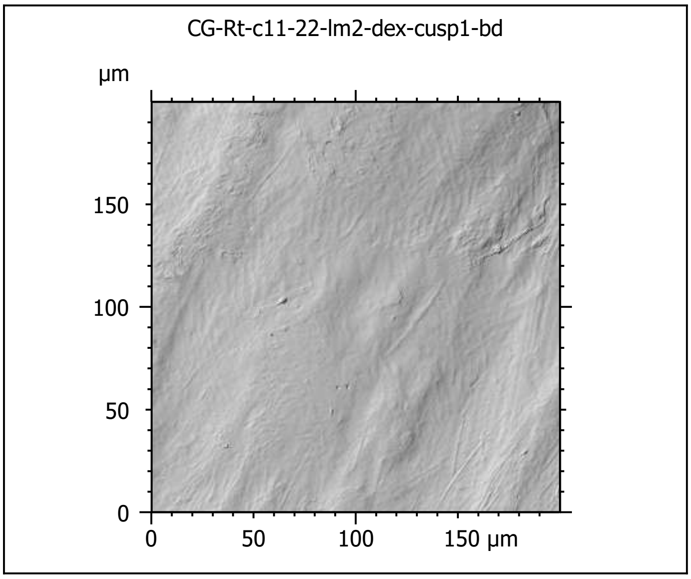

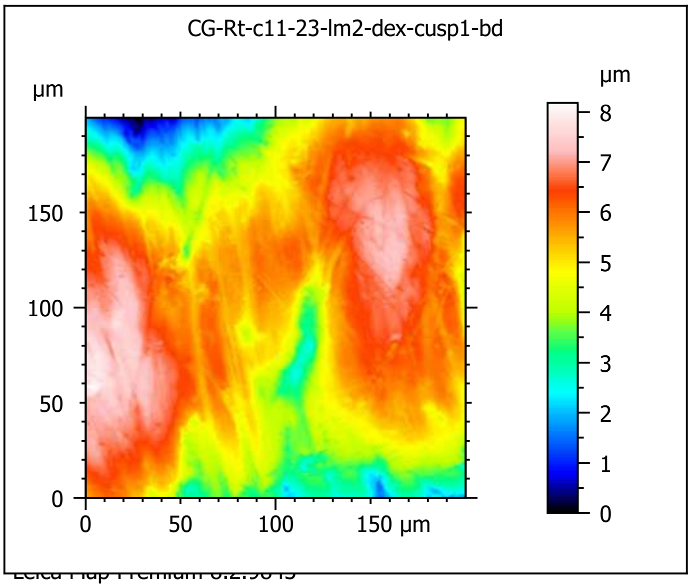

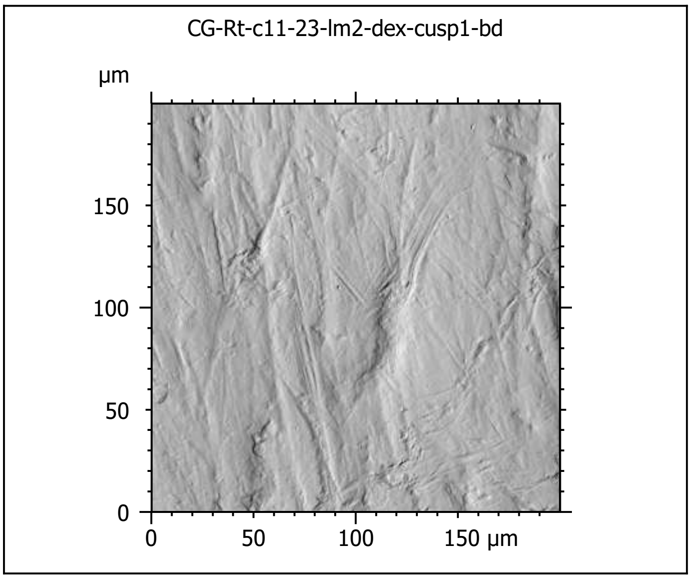

CG-Rt-c12-11-lm3-sin-cusp1-bd

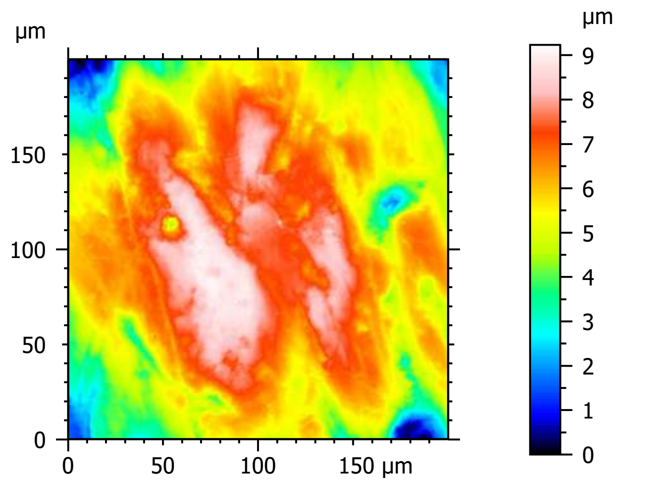

CG-Rt-c12-11-lm3-sin-cusp1-bd

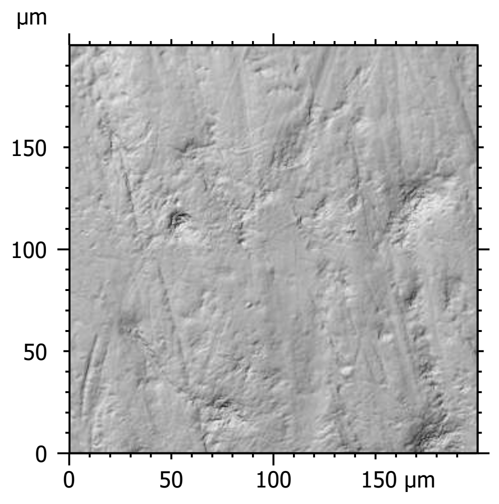

CG-Rt-c12-12-lm3-dex-cusp1-bd

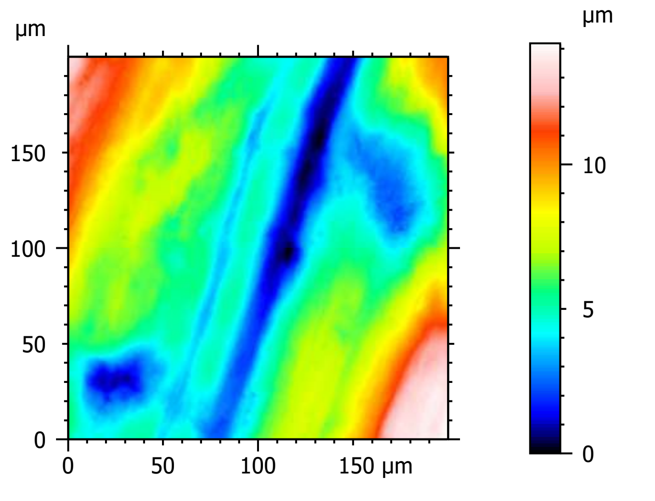

CG-Rt-c12-12-lm3-dex-cusp1-bd

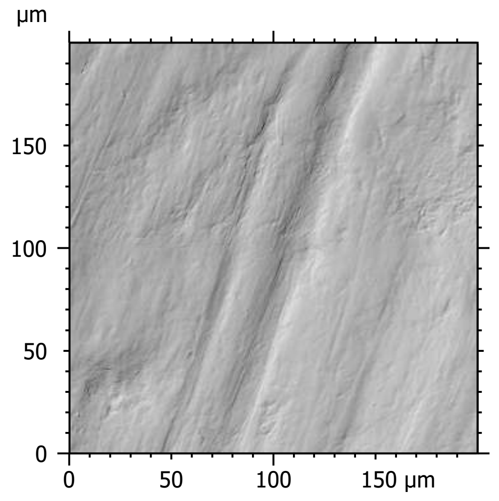

CG-Rt-c12-13-lm1-dex-cusp1-bd

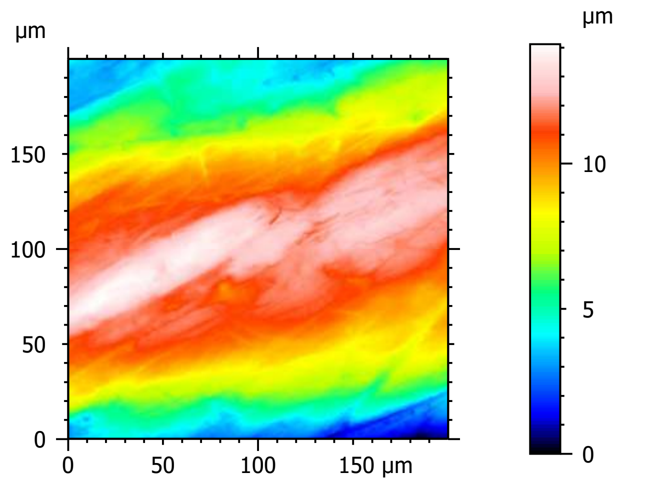

CG-Rt-c12-13-lm1-dex-cusp1-bd

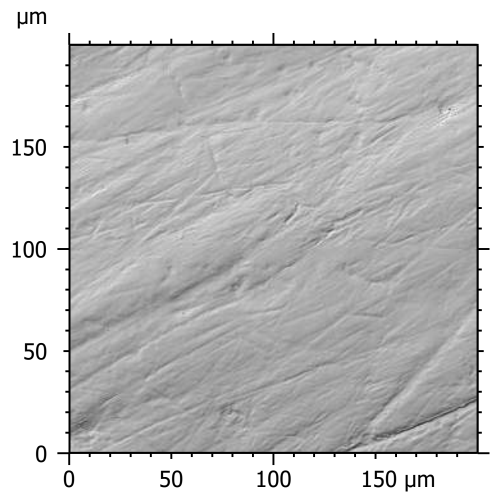

Leica Map Premium 8.2.9843

CG-Rt-c12-14-lm-sin-cusp1-bd

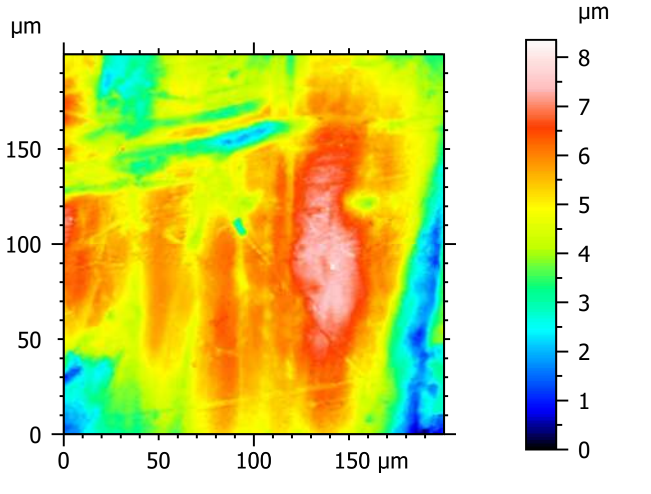

CG-Rt-c12-14-lm-sin-cusp1-bd

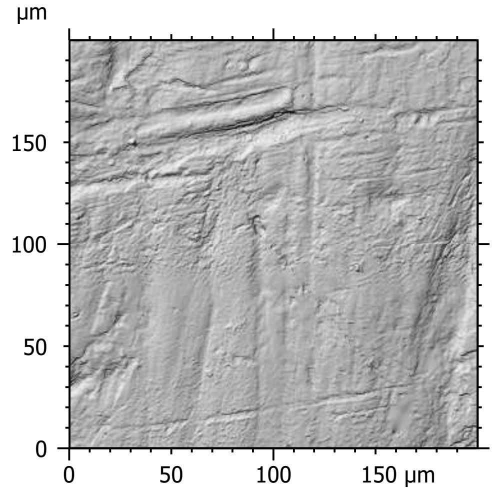

CG-Rt-c13-1-lm2-sin-cusp1-bd

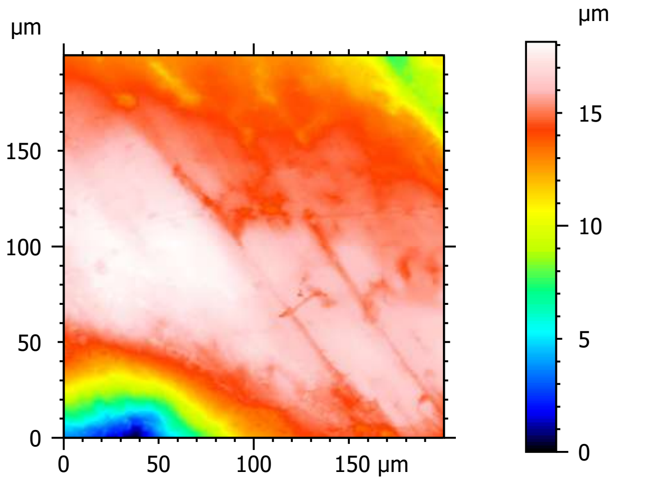

CG-Rt-c13-1-lm2-sin-cusp1-bd

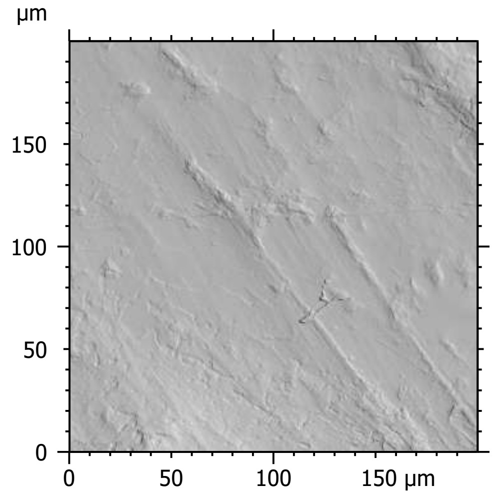

CG-Rt-c13-2-lm2-sin-cusp1-bd

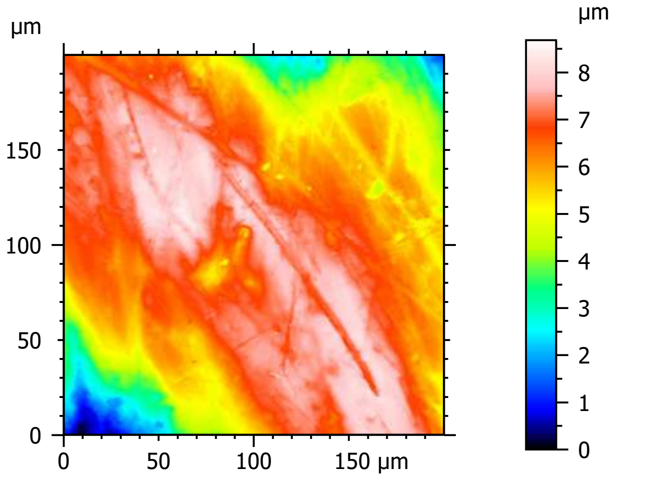

CG-Rt-c13-2-lm2-sin-cusp1-bd

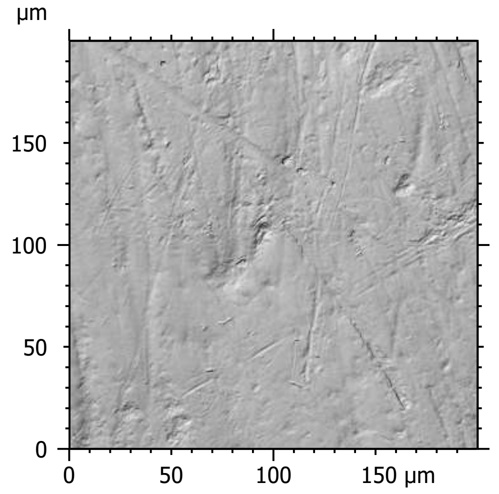

Leica Map Premium 8.2.9843

CG-Rt-c13-3-lm2-sin-cusp1-bd

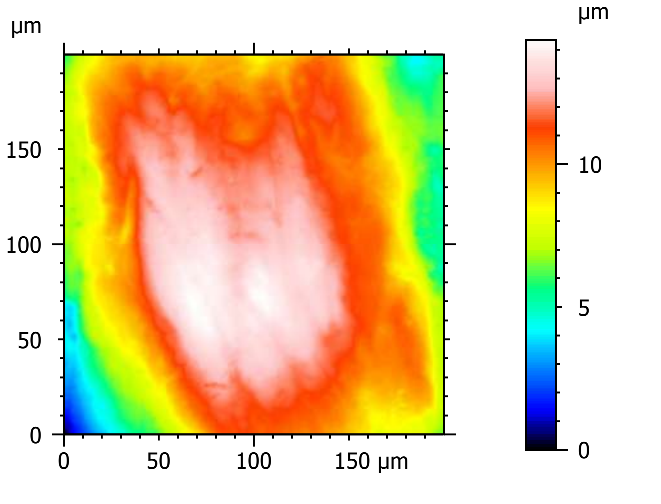

CG-Rt-c13-3-lm2-sin-cusp1-bd

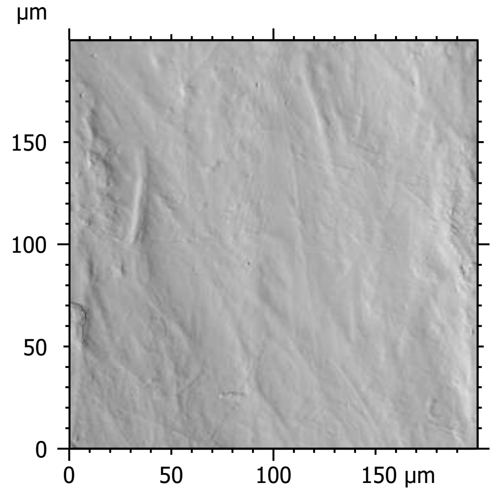

CG-Rt-c13-4-lm2-dex-cusp1-bd

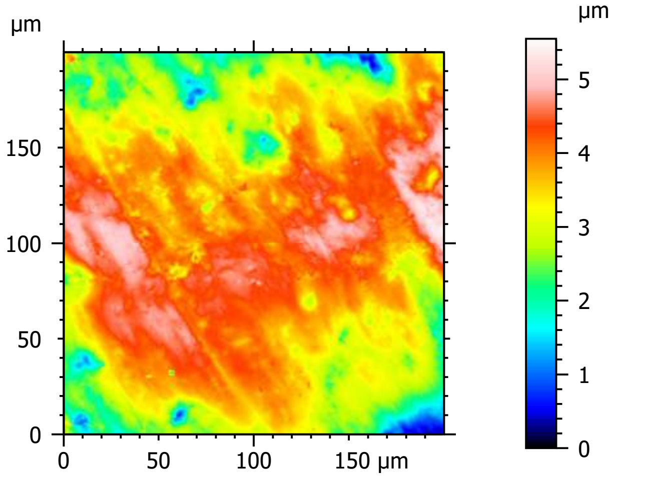

CG-Rt-c13-4-lm2-dex-cusp1-bd

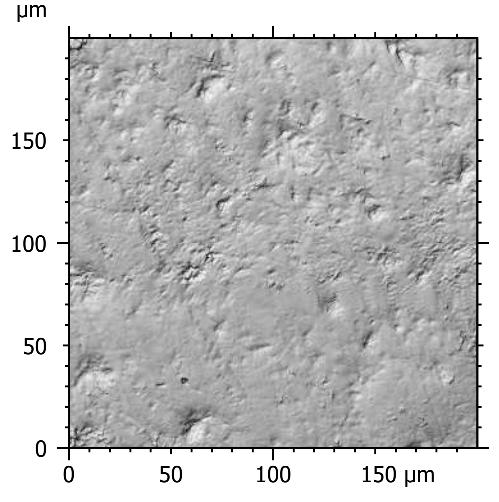

CG-Rt-c14-132-lm3-SIN-cusp1-bd

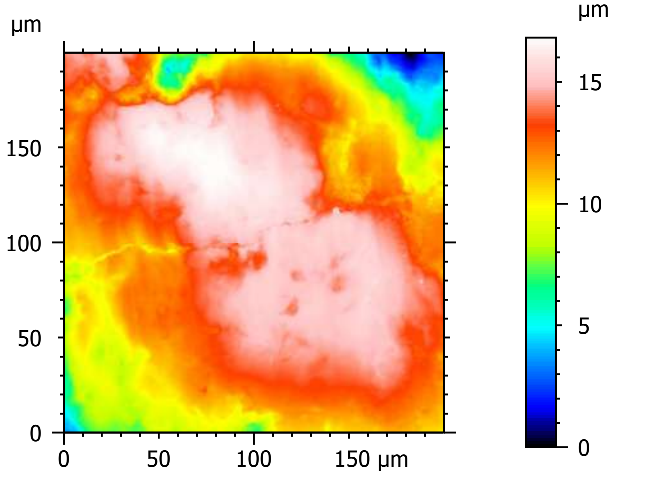

CG-Rt-c14-132-lm3-SIN-cusp1-bd

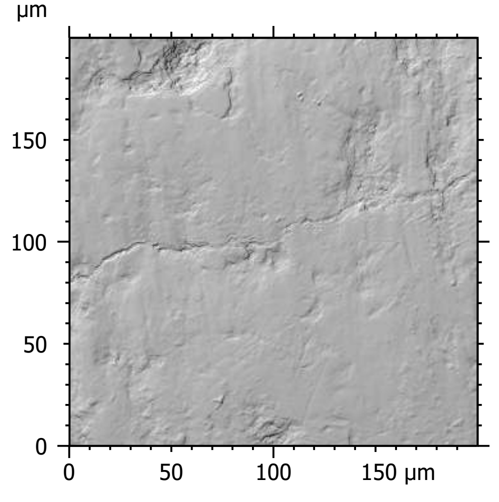

Leica Map Premium 8.2.9843

CG-Rt-c14-133-lm2-sin-cusp1-bd

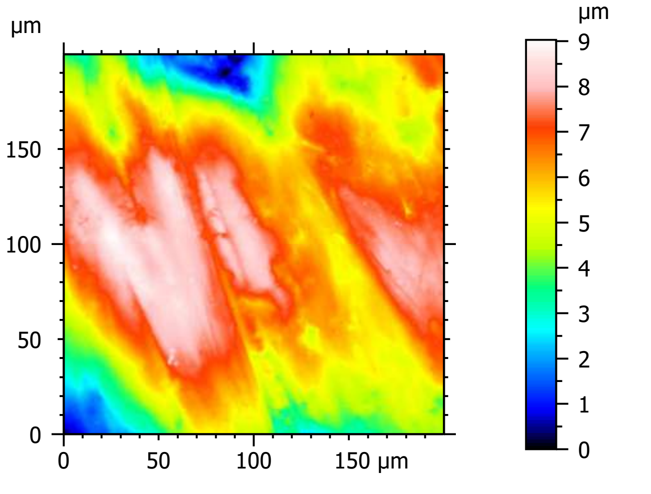

CG-Rt-c14-133-lm2-sin-cusp1-bd

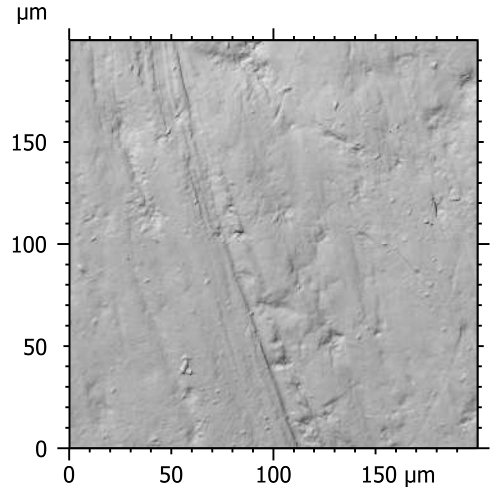

CG-Rt-c14-134-lm-sin-cusp1-bd

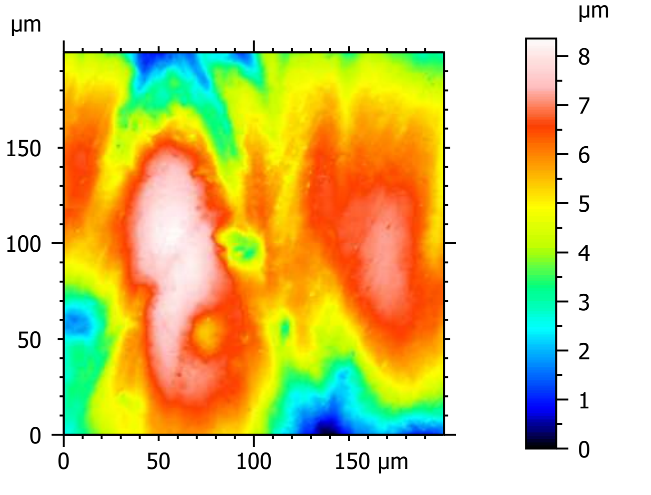

CG-Rt-c14-134-lm-sin-cusp1-bd

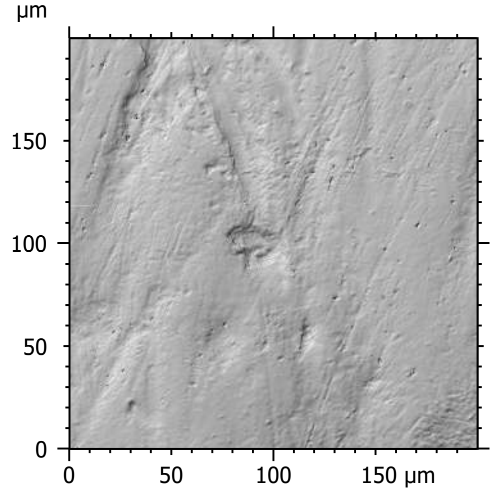

CG-Rt-c14-135-lm3-sin-cusp1-bd

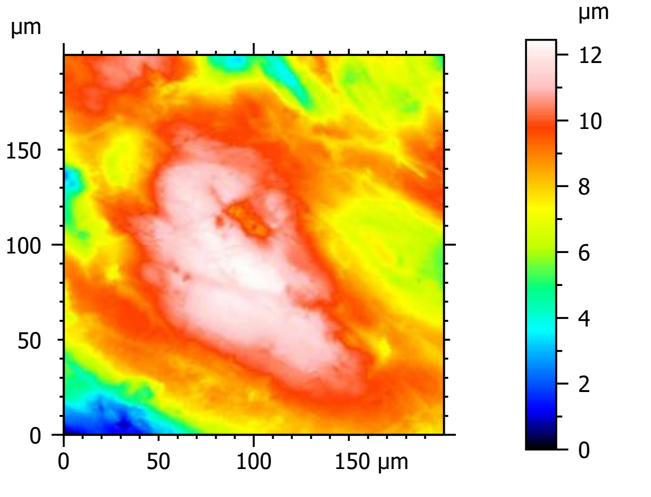

CG-Rt-c14-135-lm3-sin-cusp1-bd

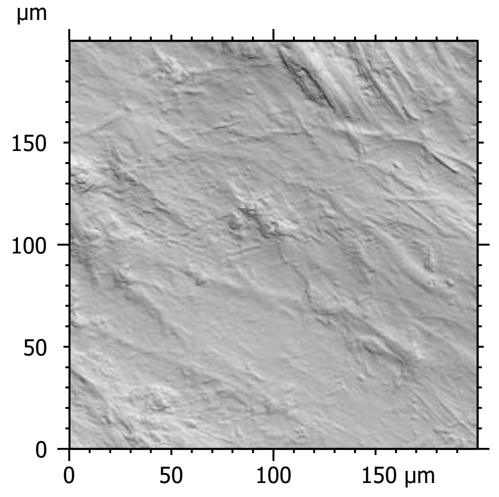

Leica Map Premium 8.2.9843

CG-Rt-c14-136-lm1-sin-cusp1-bd

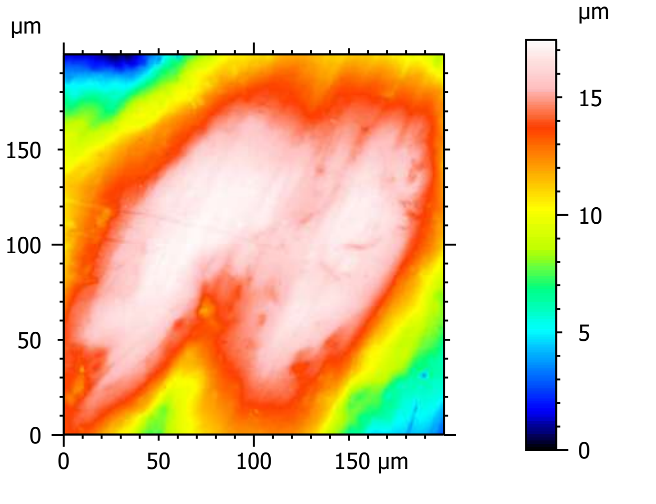

CG-Rt-c14-136-lm1-sin-cusp1-bd

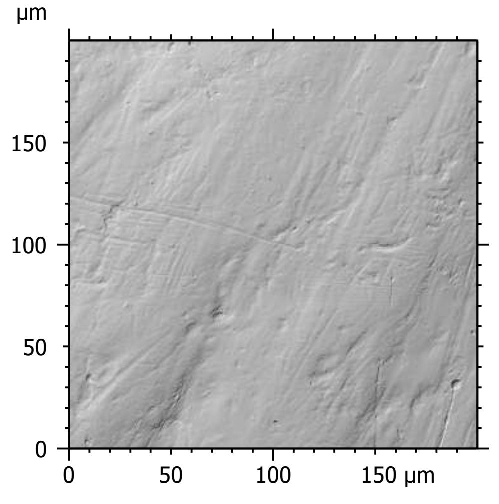

CG-Rt-c14-137-lm-sin-cusp1-bd

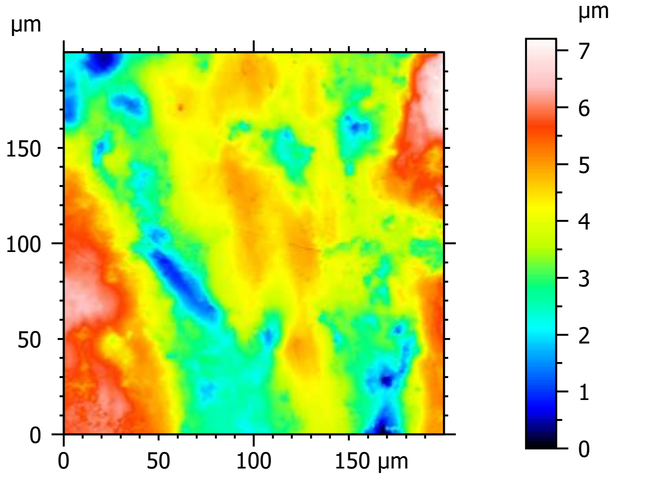

CG-Rt-c14-137-lm-sin-cusp1-bd

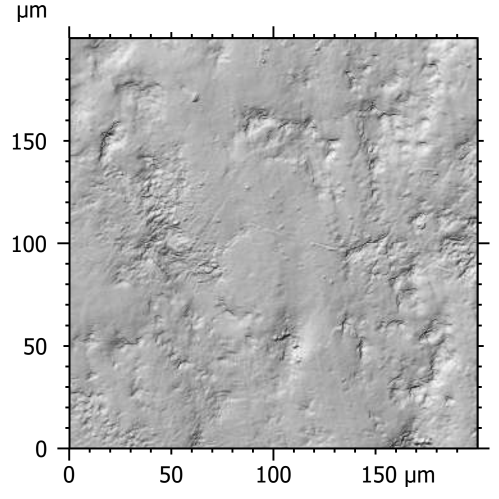

CG-Rt-c14-138-lm-sin-cusp1-bd

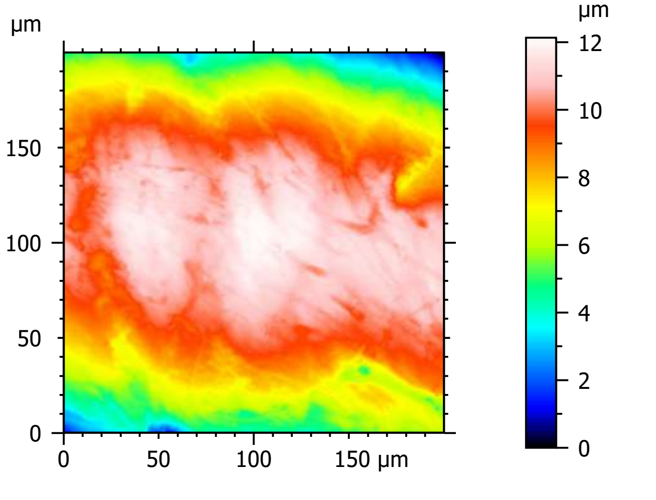

CG-Rt-c14-138-lm-sin-cusp1-bd

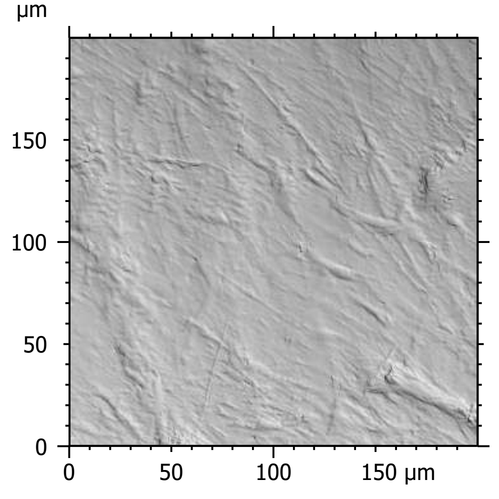

Leica Map Premium 8.2.9843

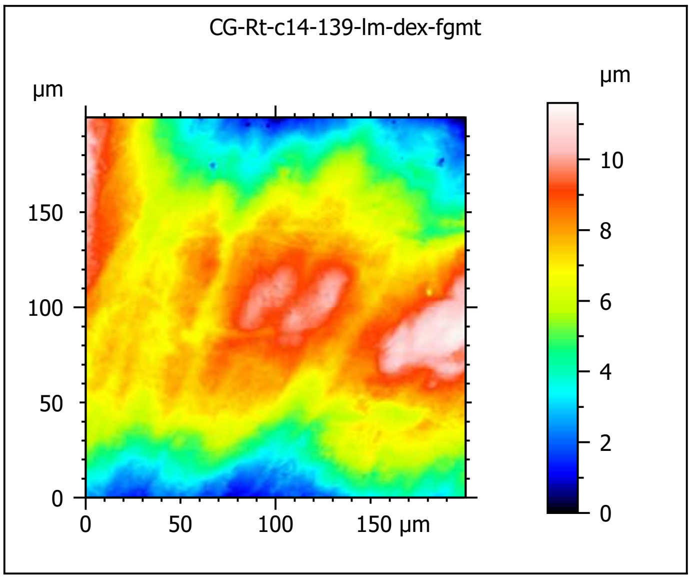

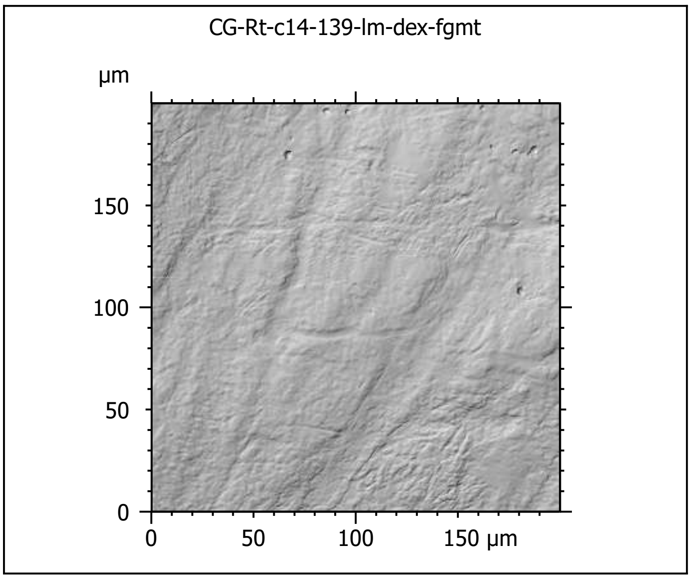

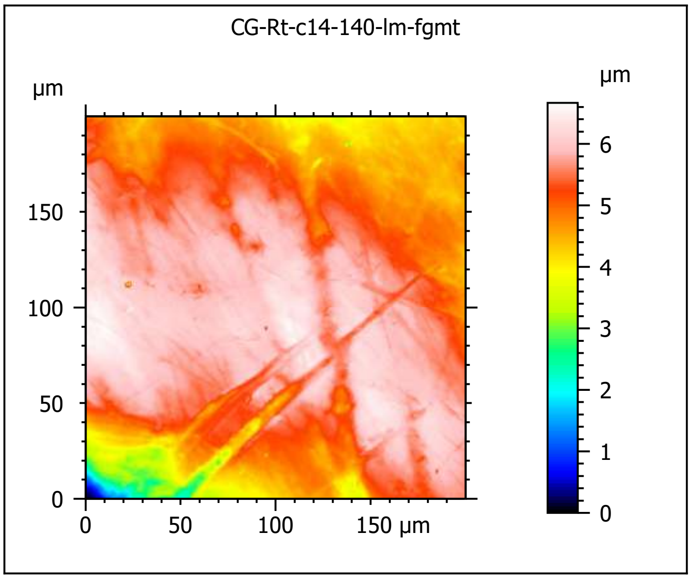

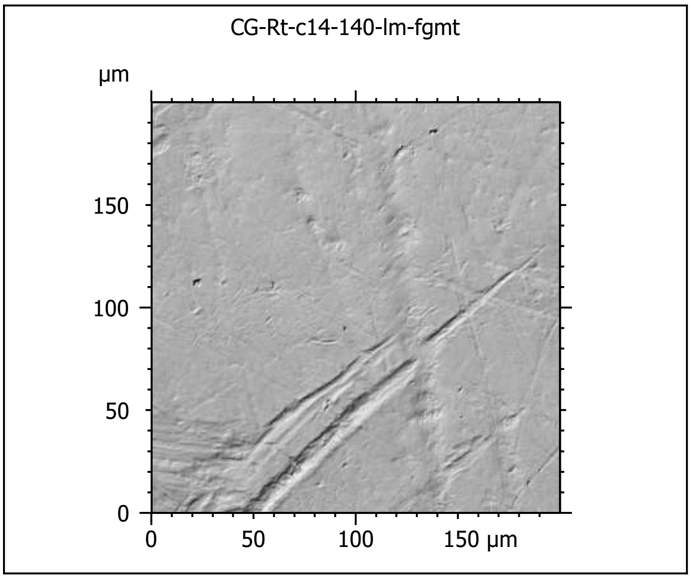

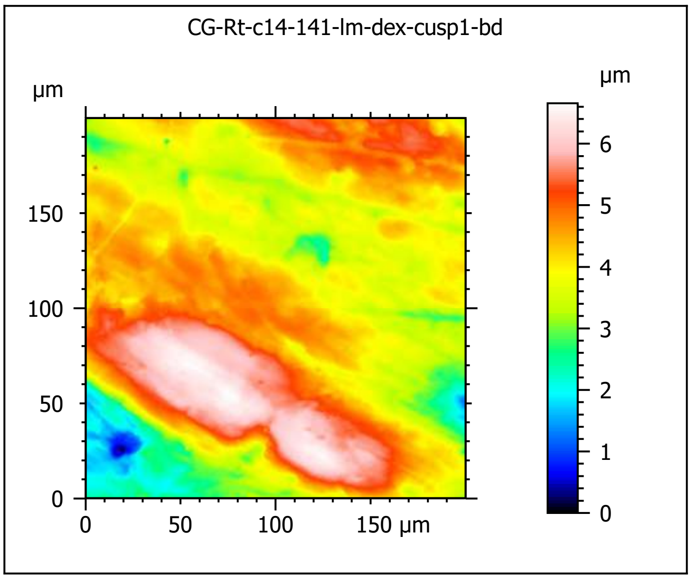

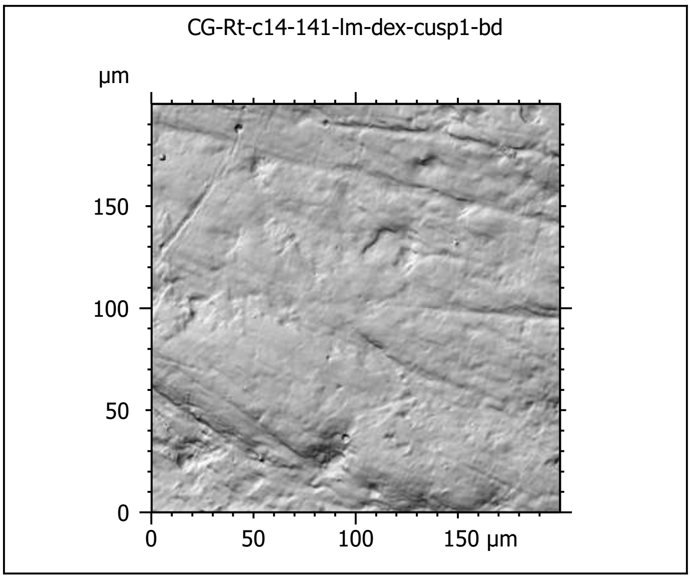

Leica Map Premium 8.2.9843

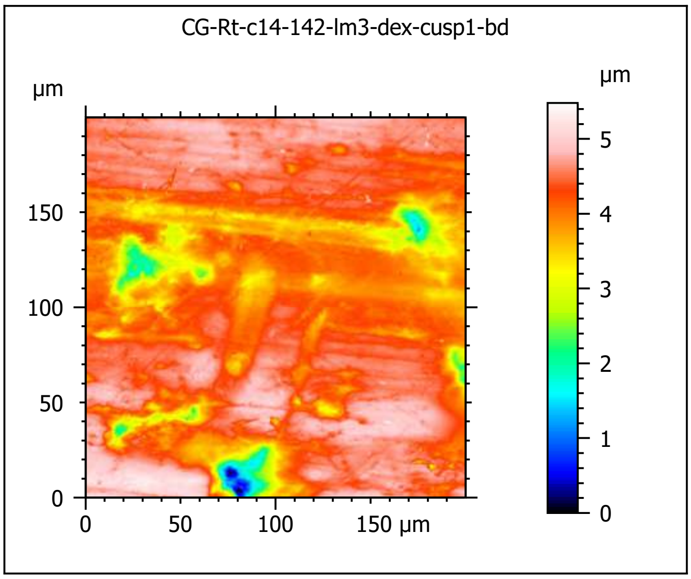

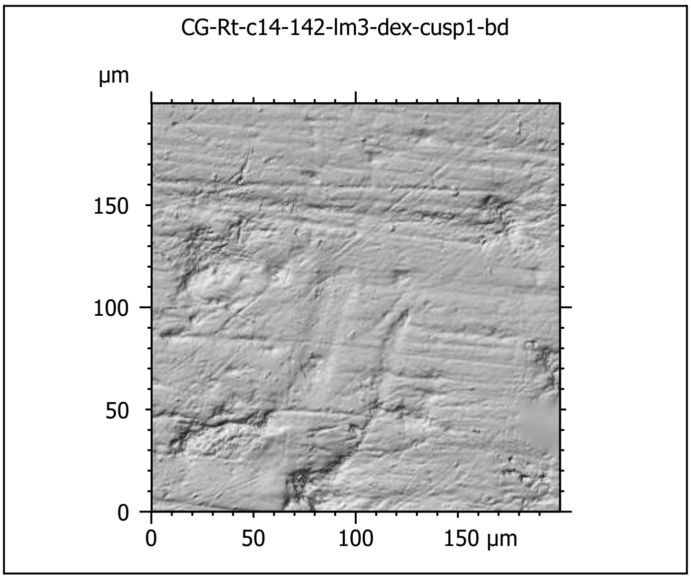

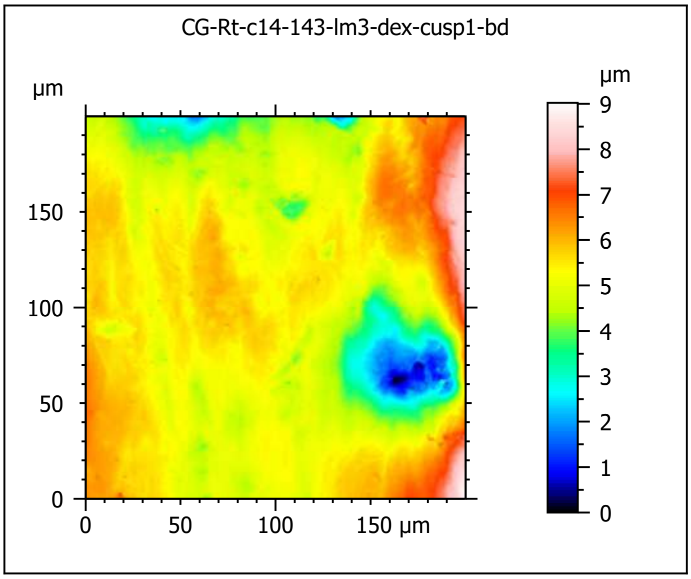

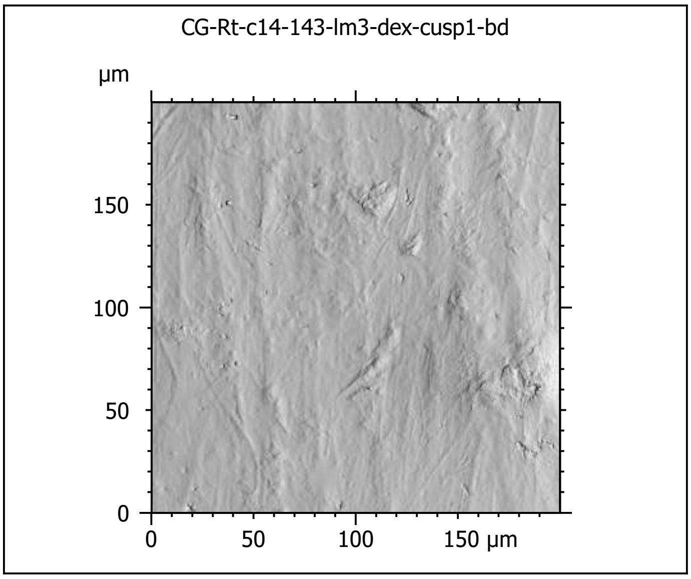

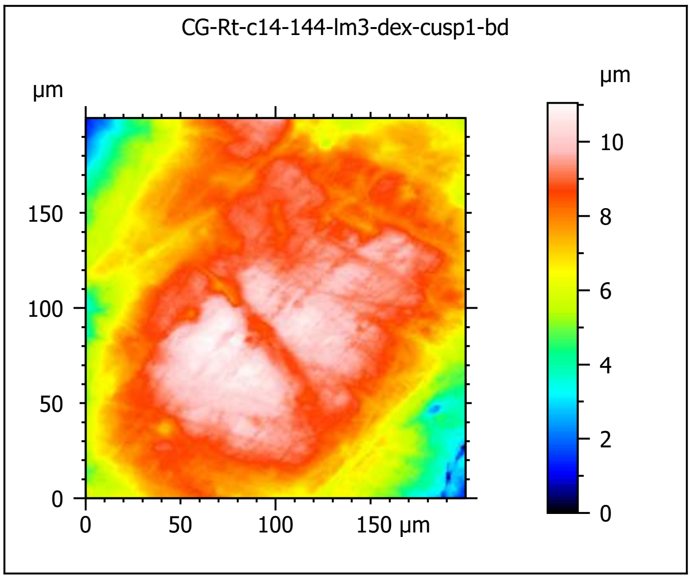

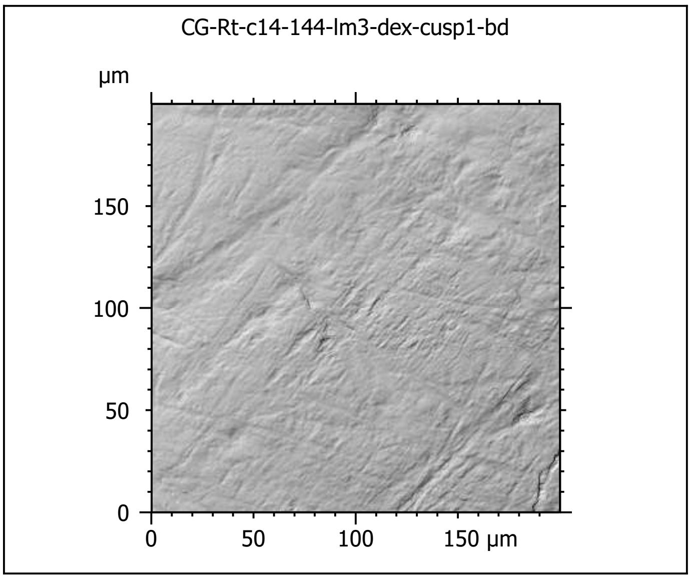

Leica Map Premium 8.2.9843

CG-Rt-c14-145-lm3-dex-cusp1-bd

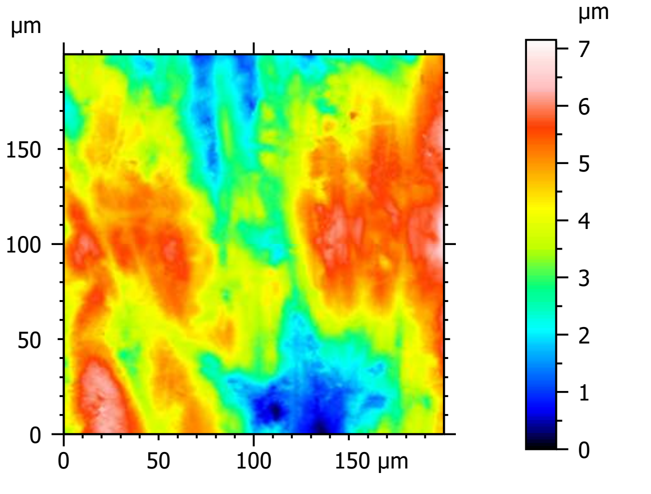

CG-Rt-c14-145-lm3-dex-cusp1-bd

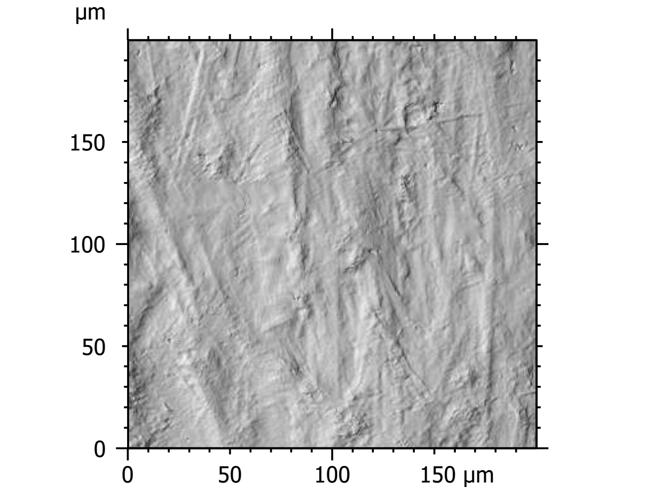

CG-Rt-c14-146-lm2-dex-cusp1-bd

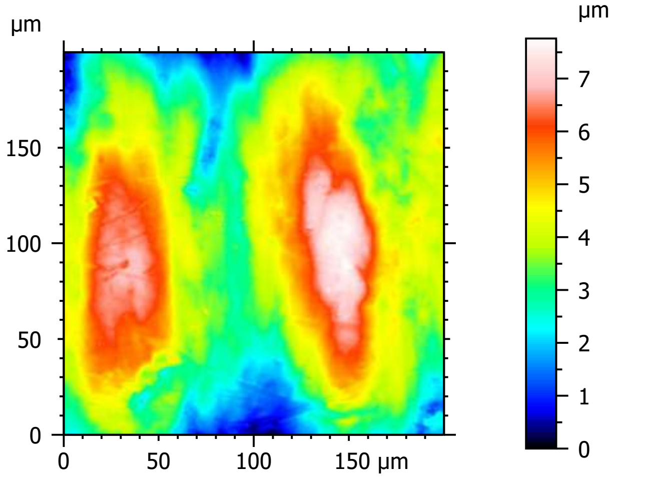

CG-Rt-c14-146-lm2-dex-cusp1-bd

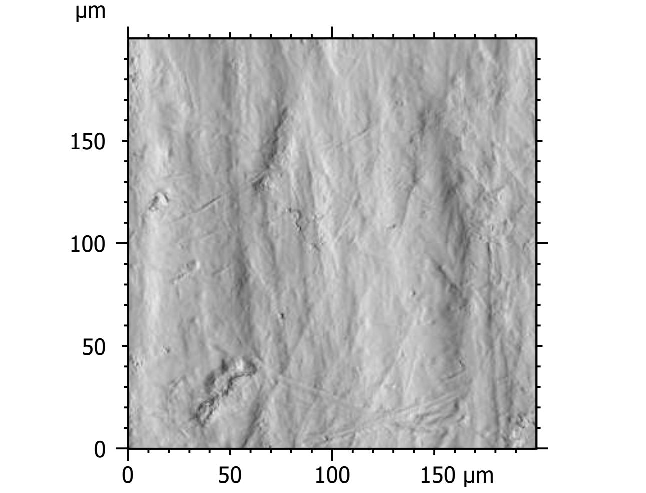

CG-Rt-c14-147-lm2-dex-cusp1-bd

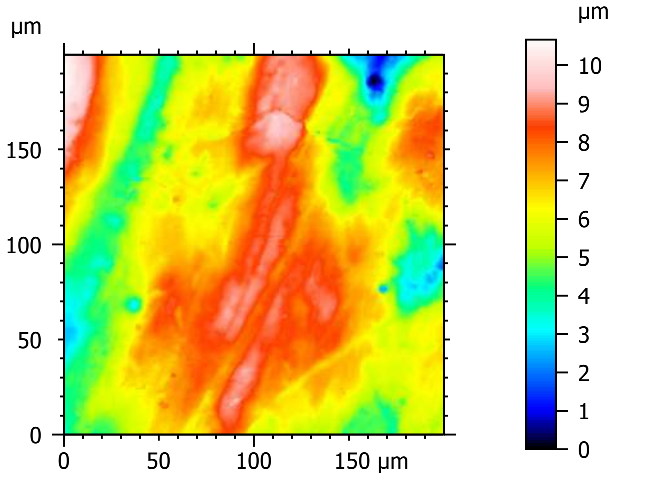

CG-Rt-c14-147-lm2-dex-cusp1-bd

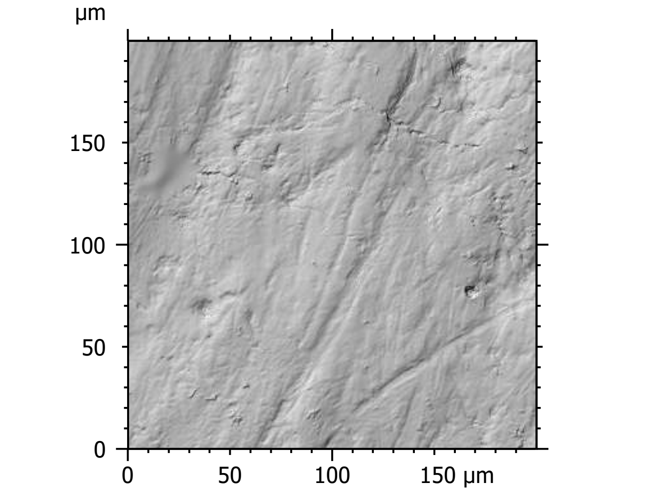

Leica Map Premium 8.2.9843

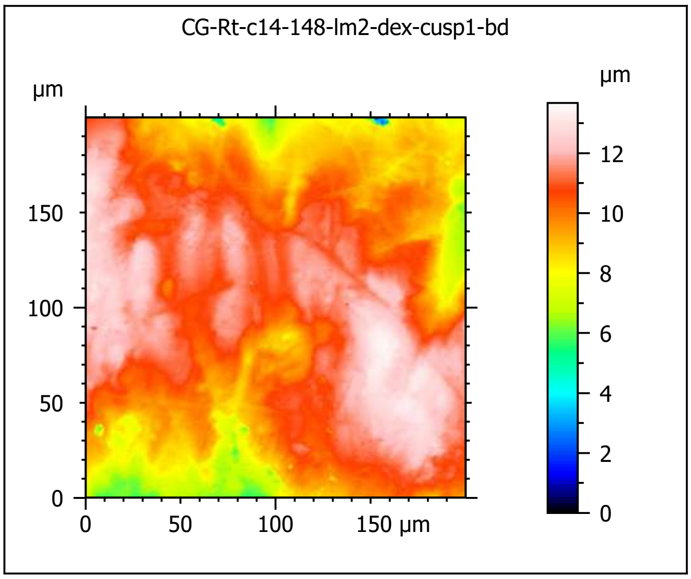

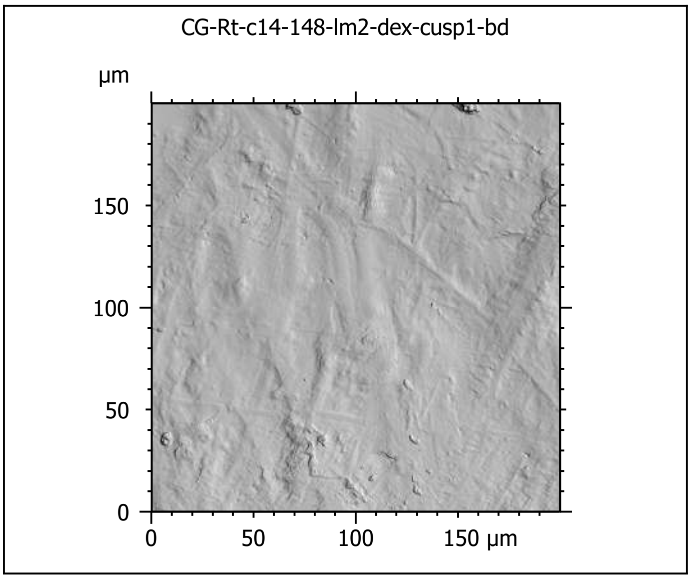

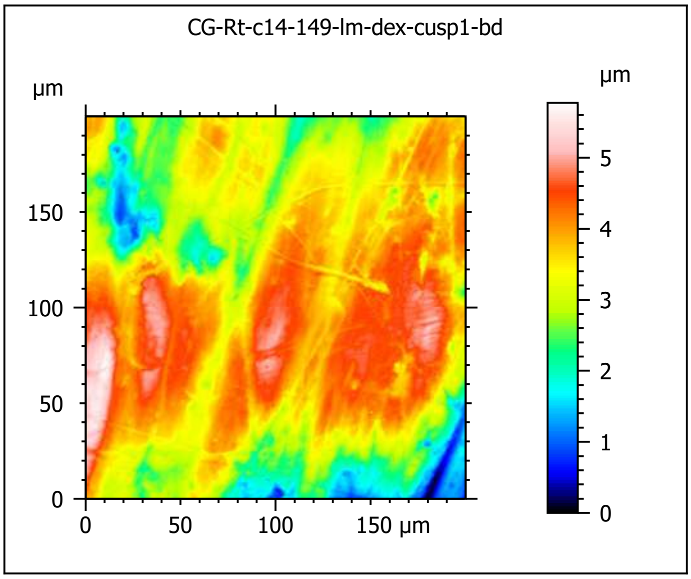

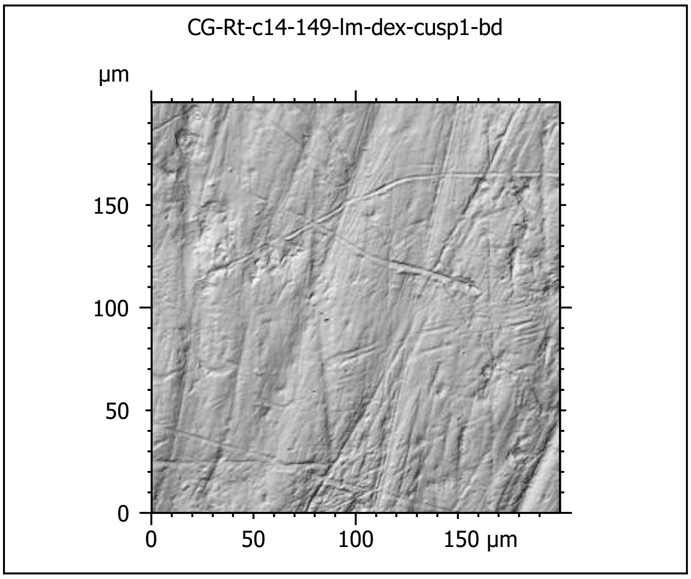

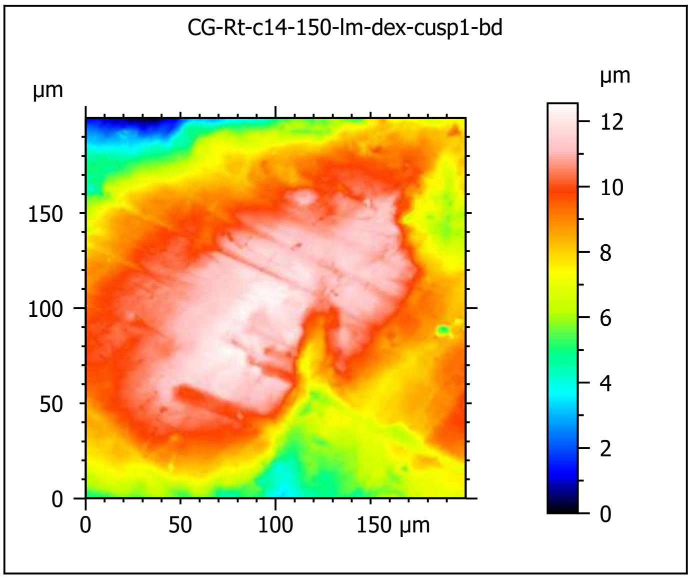

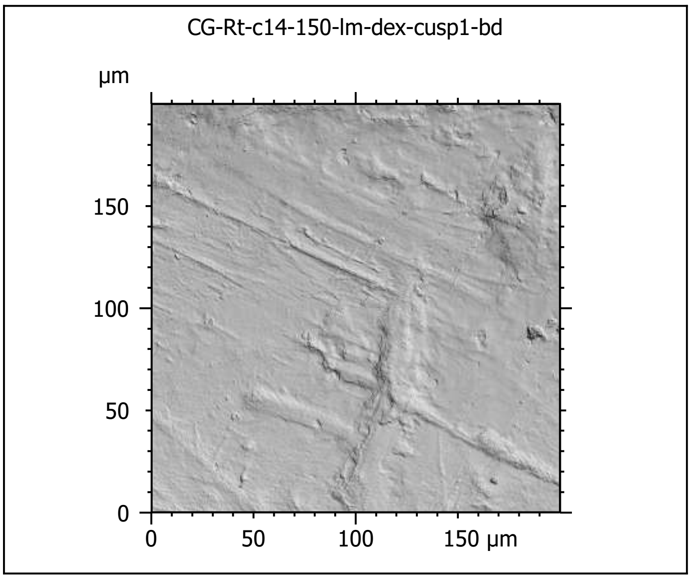

Leica Map Premium 8.2.9843

CG-Rt-c14-151-lm-dex-cusp1-bd

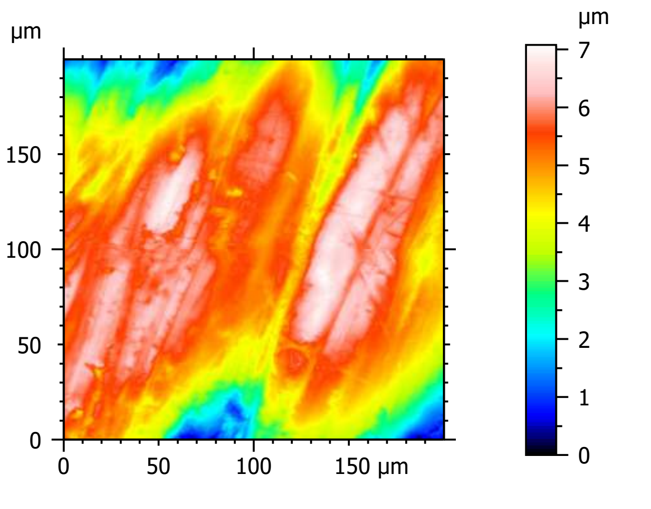

CG-Rt-c14-151-lm-dex-cusp1-bd

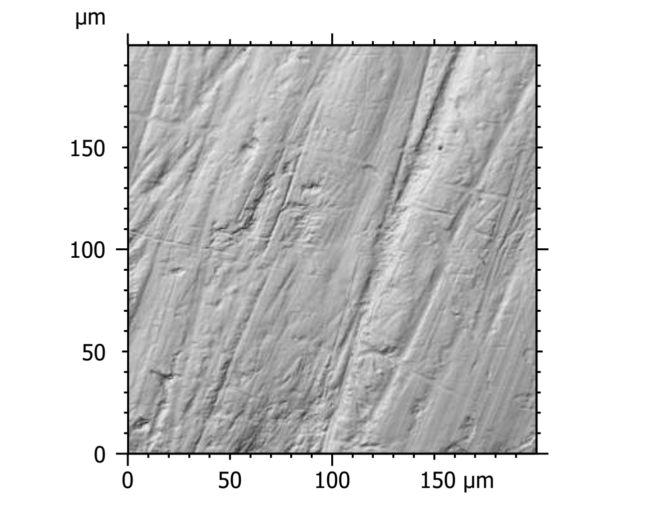

CG-Rt-c14-152-lm2-dex-cusp1-bd

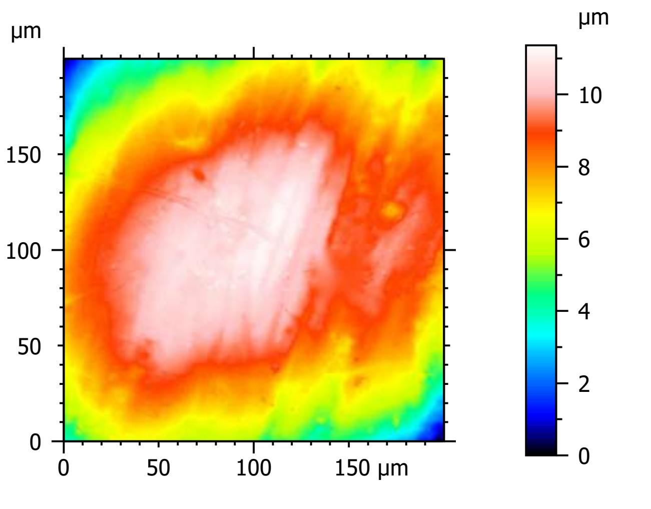

CG-Rt-c14-152-lm2-dex-cusp1-bd

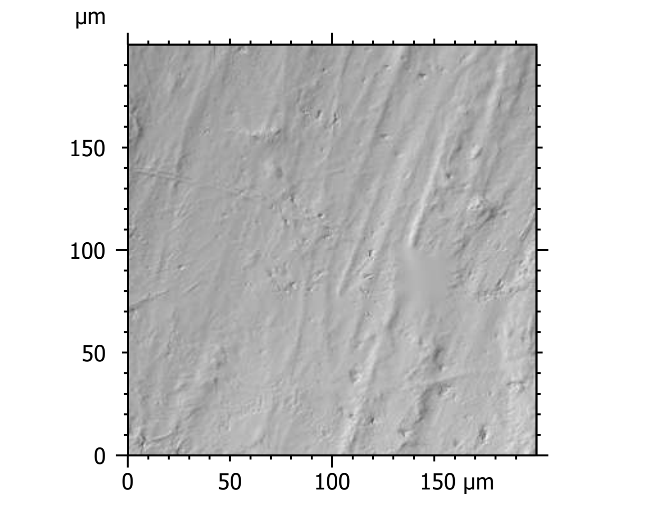

CG-Rt-c15-128-lm-sin-cusp1-bd

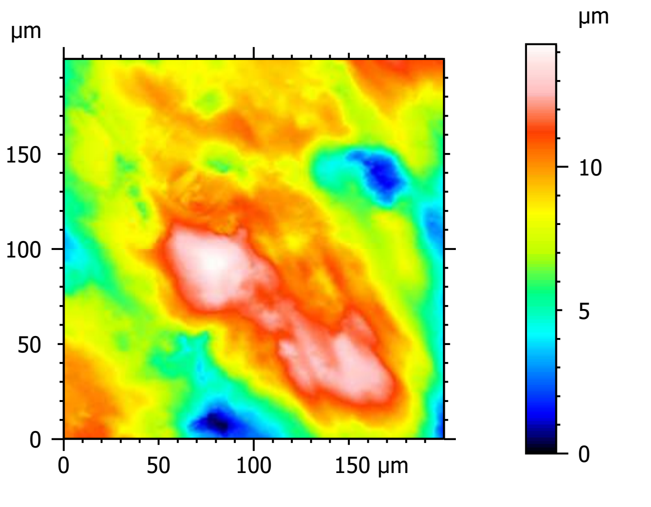

CG-Rt-c15-128-lm-sin-cusp1-bd

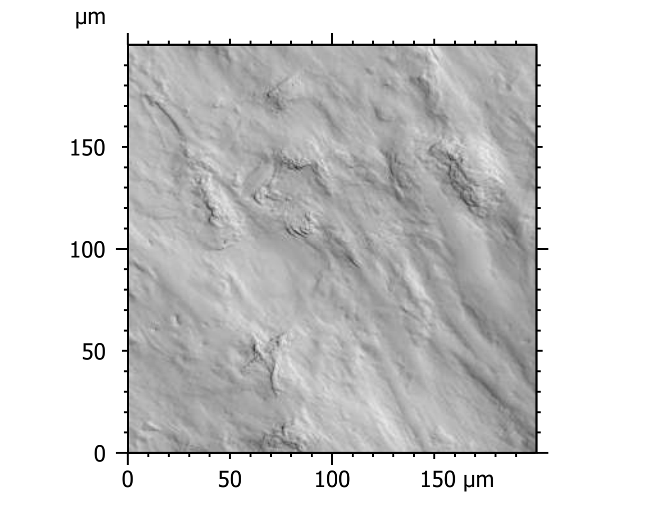

Leica Map Premium 8.2.9843

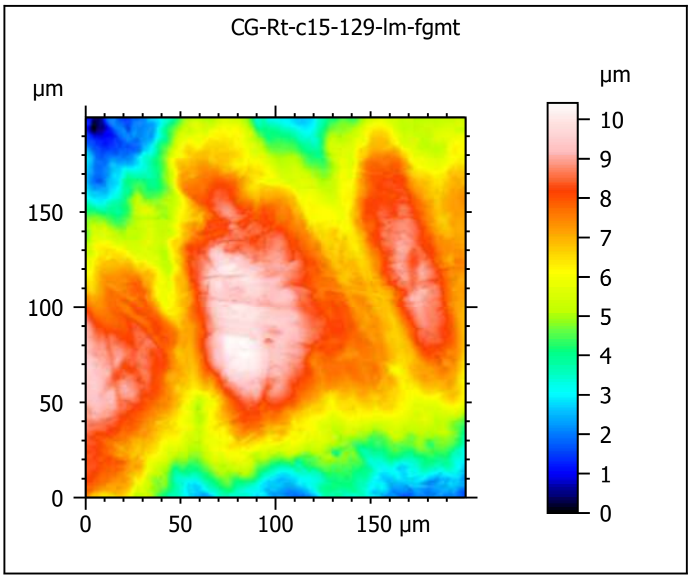

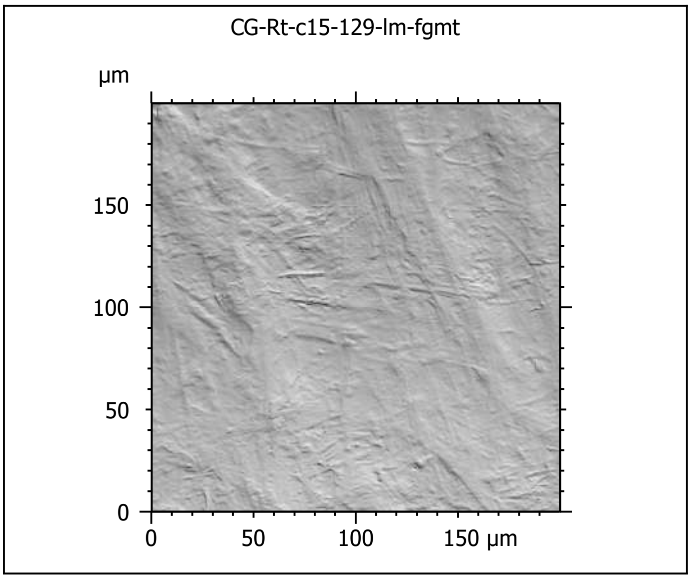

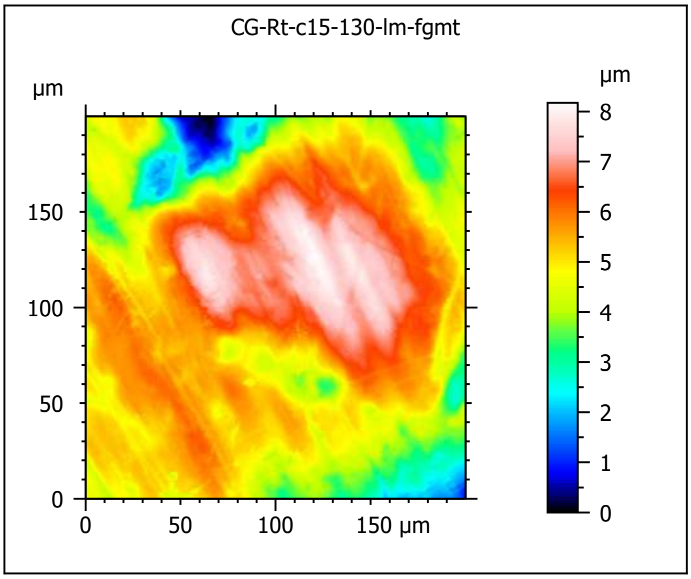

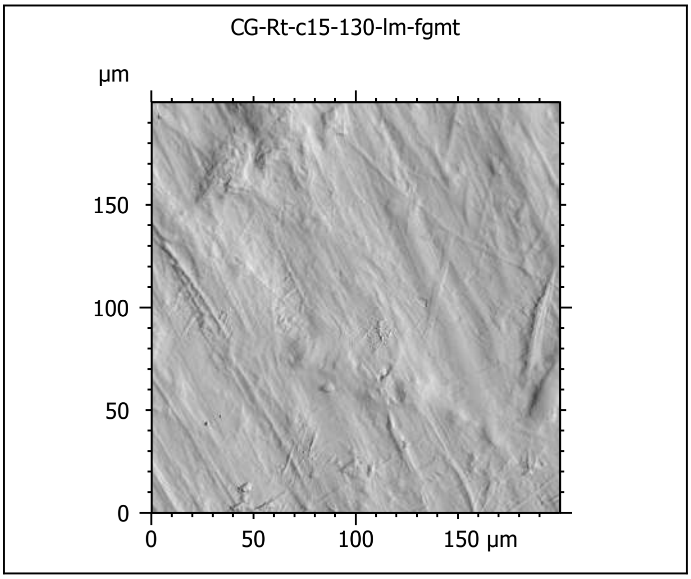

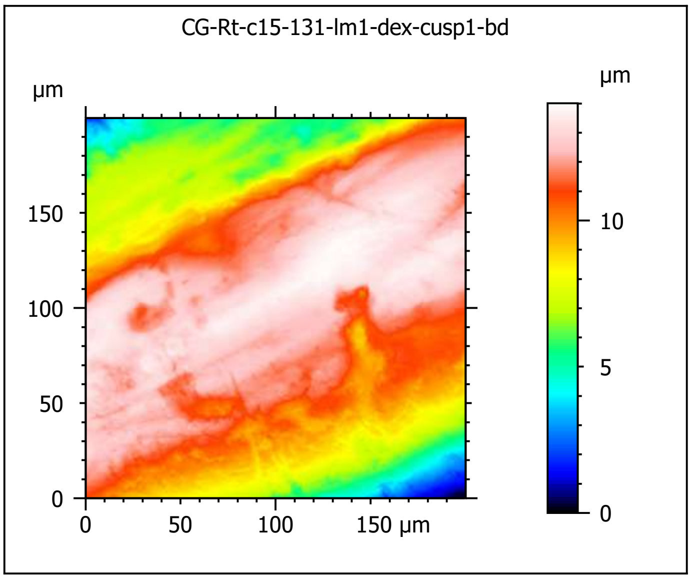

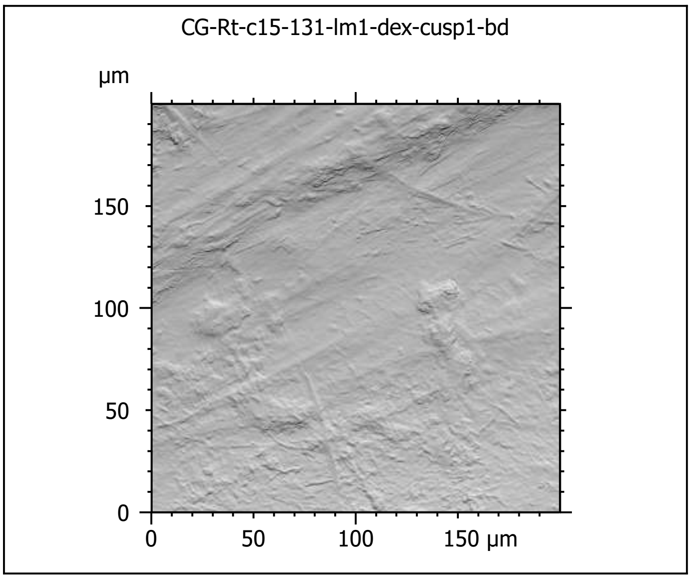

Leica Map Premium 8.2.9843

"A long-term perspective on Neandertal environment and subsistence: insights from the dental micro-texture analysis of hunted ungulates at Combe-Grenal (Dordogne, France)"

authors: Berlioz, E.; Capdepon, E.; Discamps, E.

Appendice 2:
surfaces scanned by E. Berlioz and E. Capdepon, pre-treatement by E. Berlioz and E. Capdepon, validation by E. Berlioz (2019)

***Rangifer tarandus* - Block D**

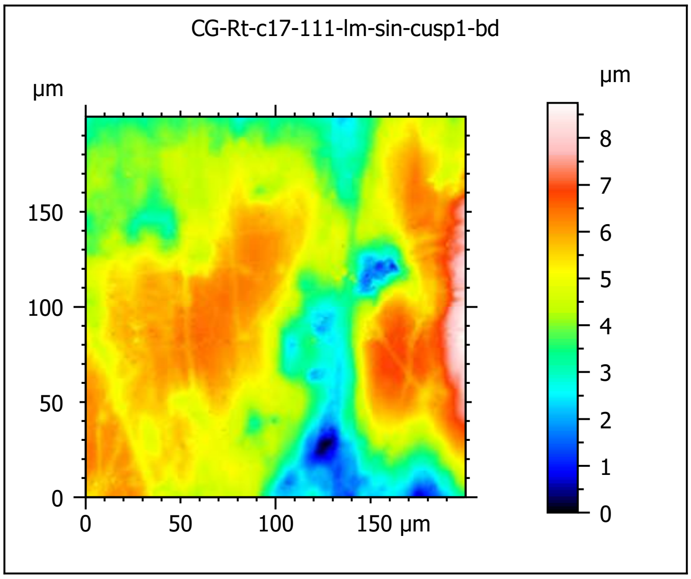

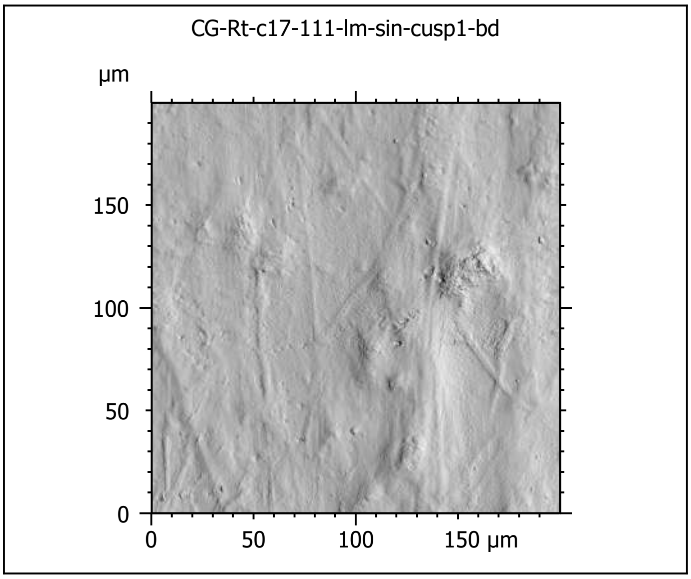

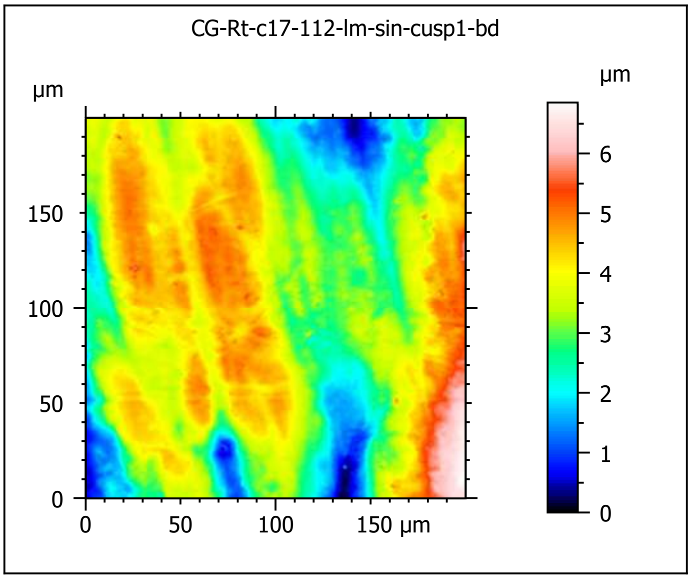

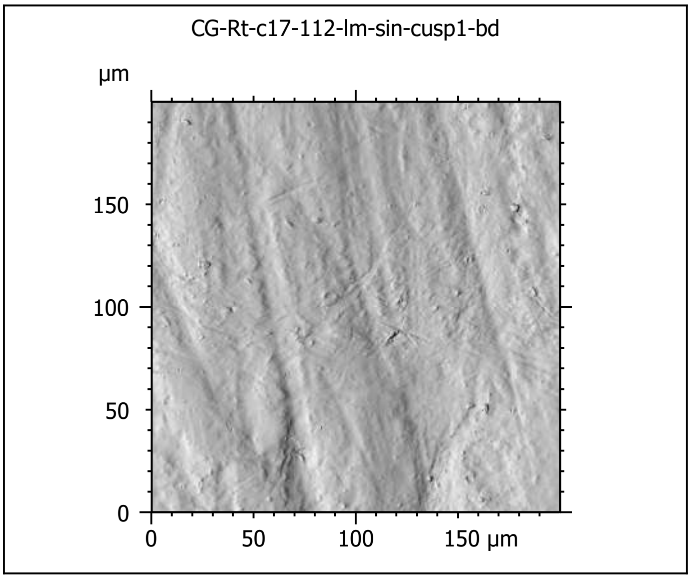

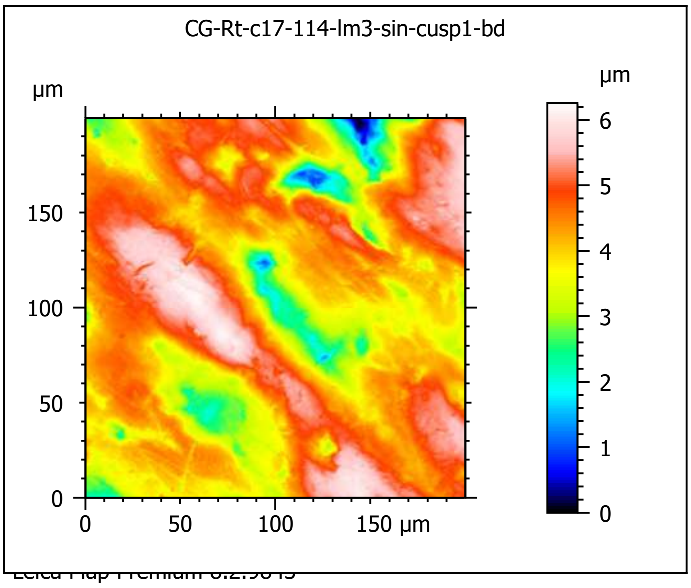

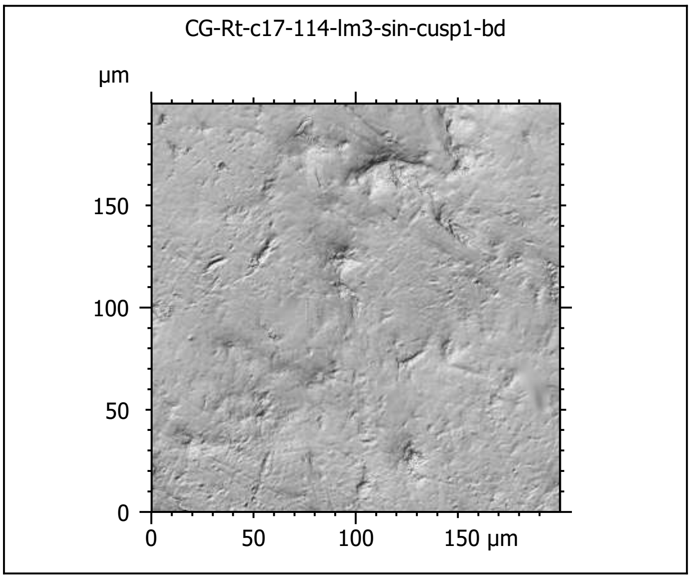

CG-Rt-c18-122-lm-sin-cusp1-bd

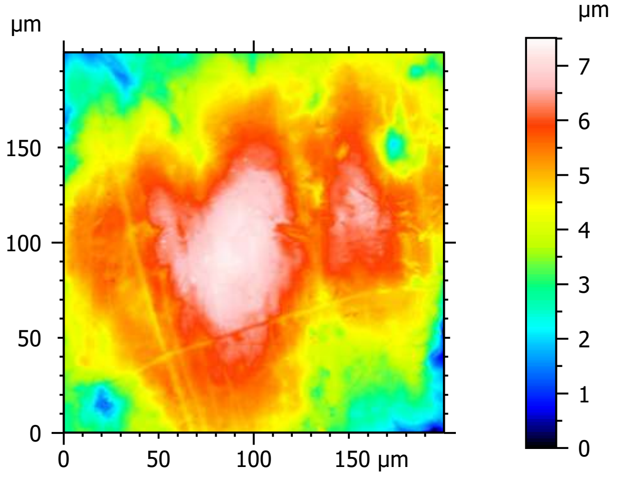

CG-Rt-c18-122-lm-sin-cusp1-bd

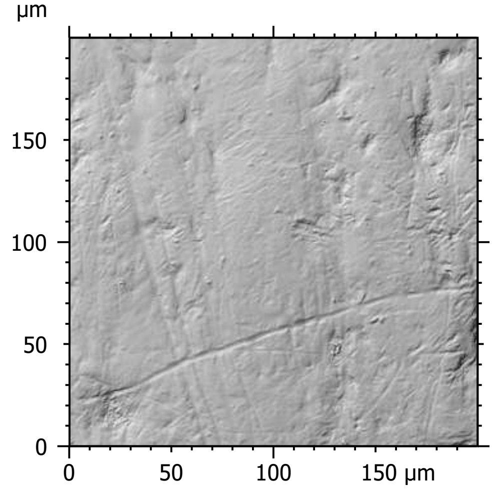

CG-Rt-c18-123-lm3-dex-cusp2-bd

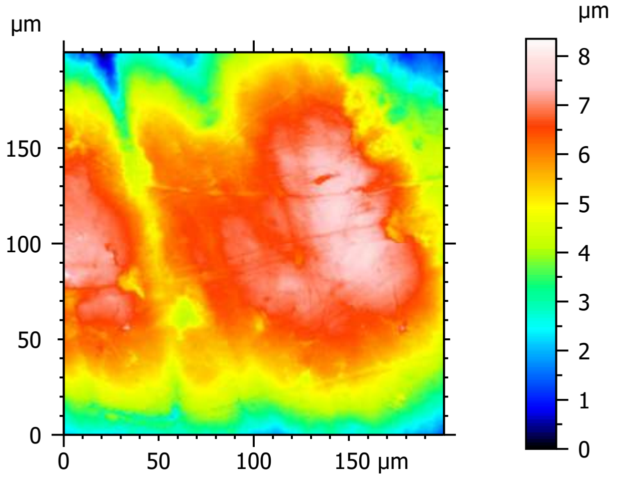

CG-Rt-c18-123-lm3-dex-cusp2-bd

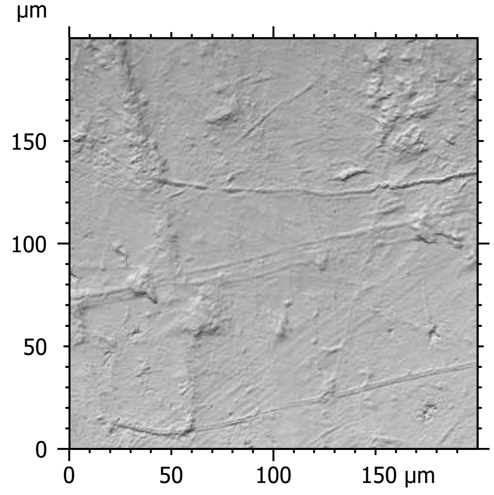

CG-Rt-c19-86-lm-sin-cusp1-bd

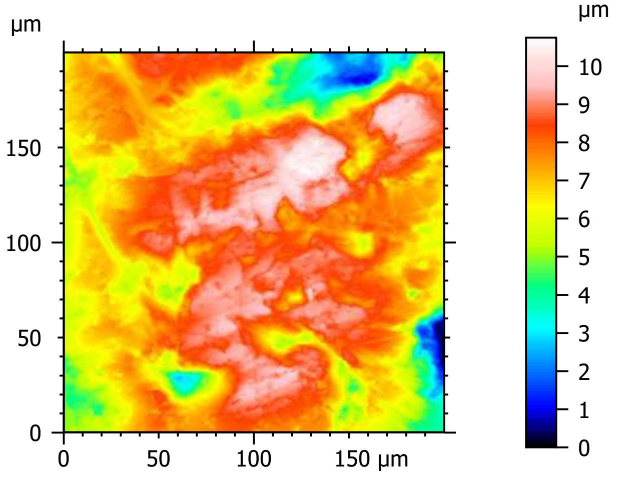

CG-Rt-c19-86-lm-sin-cusp1-bd

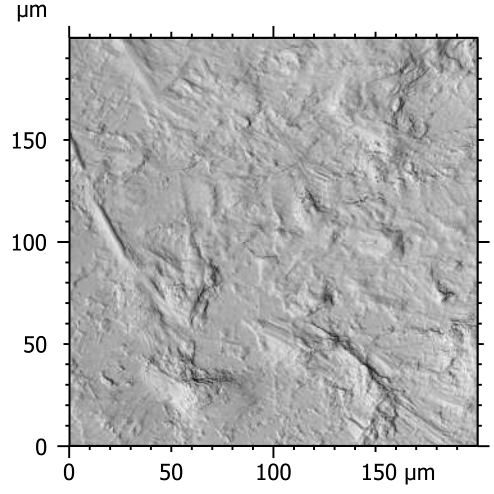

Leica Map Premium 8.2.9843

CG-Rt-c19-87-lm3-dex-cusp1-bd

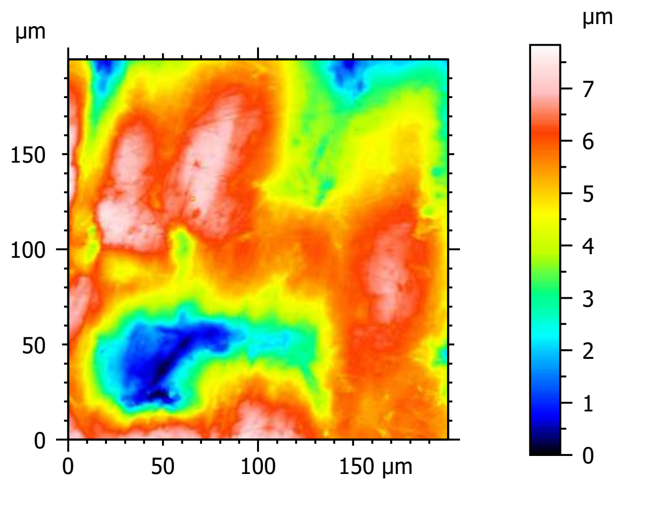

CG-Rt-c19-87-lm3-dex-cusp1-bd

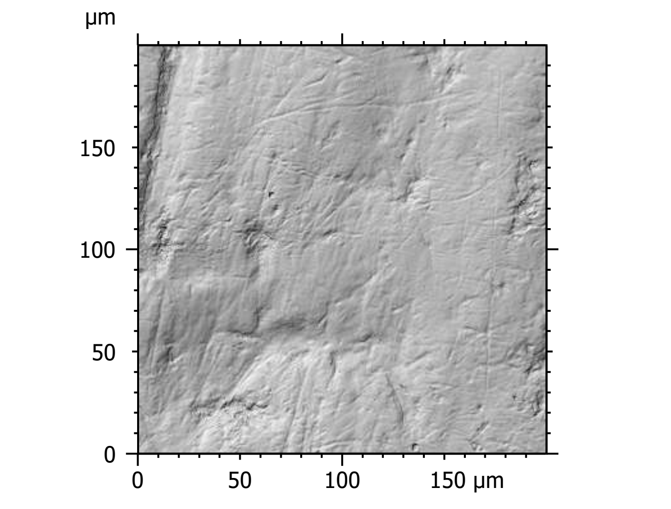

CG-Rt-c19-88-lm-sin-cusp1-bd

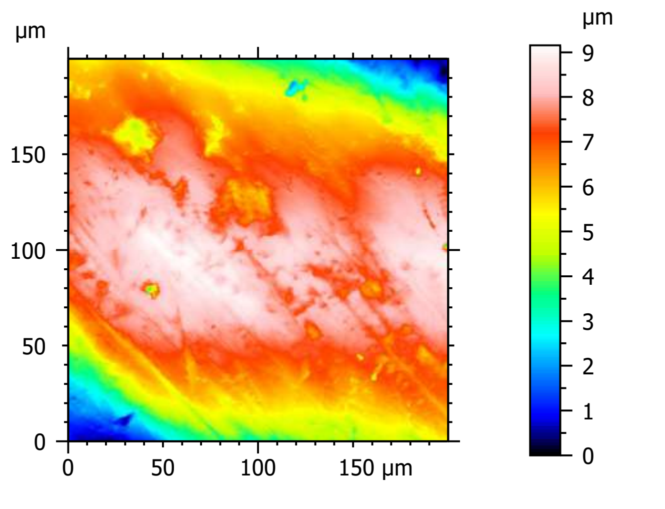

CG-Rt-c19-88-lm-sin-cusp1-bd

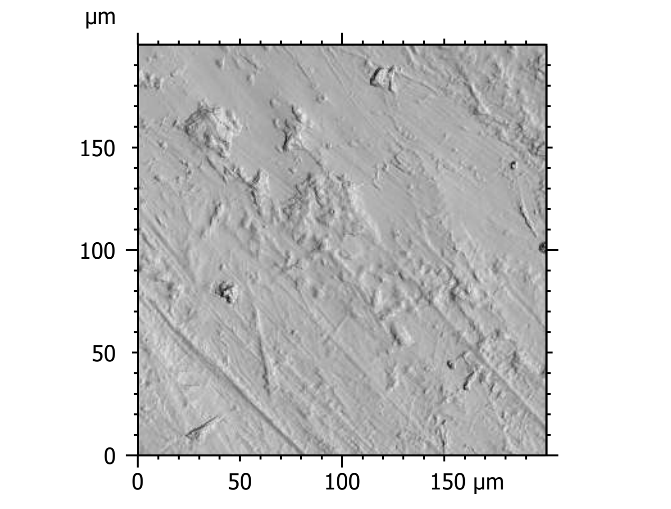

CG-Rt-c19-89-lm-sin-cusp1-bd

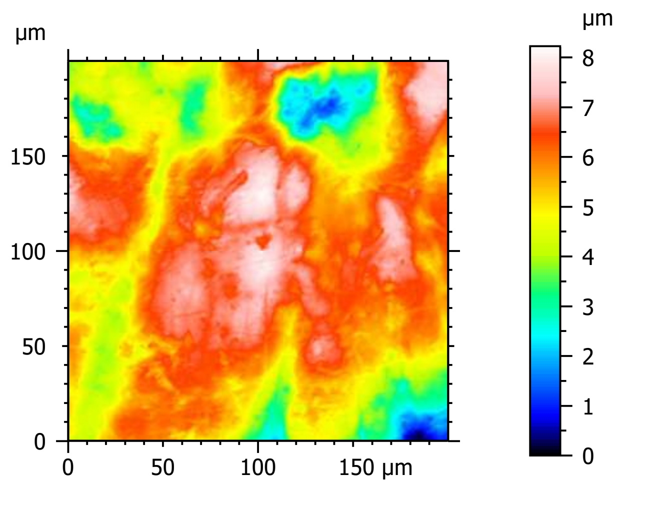

CG-Rt-c19-89-lm-sin-cusp1-bd

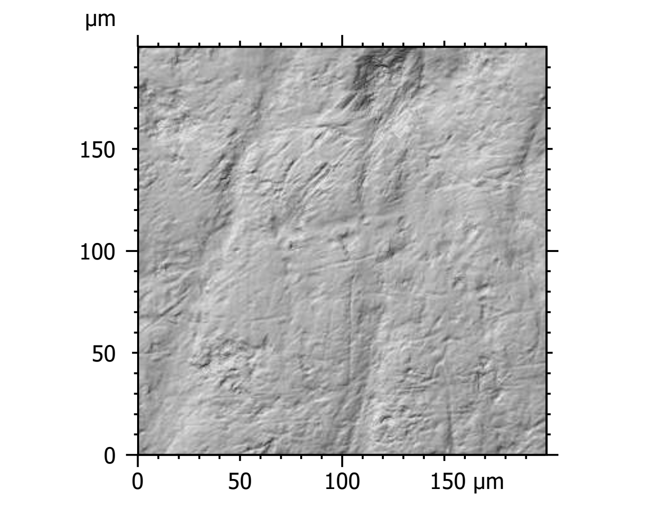

Leica Map Premium 8.2.9843

"A long-term perspective on Neandertal environment and subsistence: insights from the dental micro-texture analysis of hunted ungulates at Combe-Grenal (Dordogne, France)"

authors: Berlioz, E.; Capdepon, E.; Discamps, E.

Appendice 2:
surfaces scanned by E. Berlioz and E. Capdepon, pre-treatement by E. Berlioz and E. Capdepon, validation by E. Berlioz (2019)

Rangifer tarandus **- Block E**

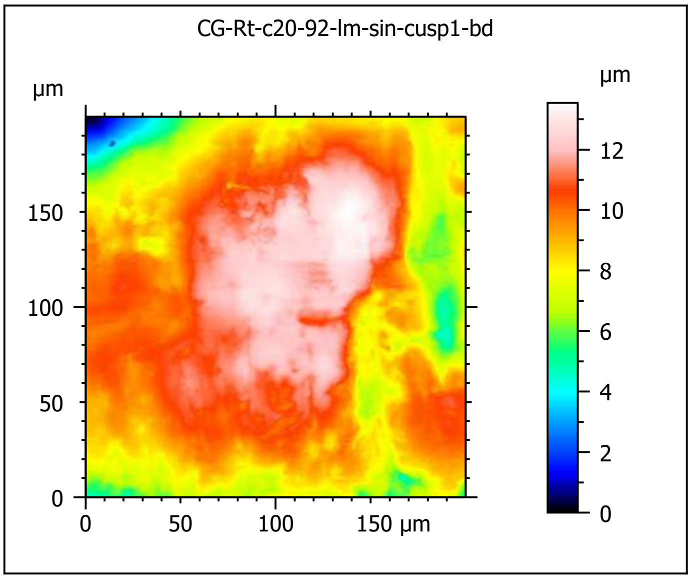

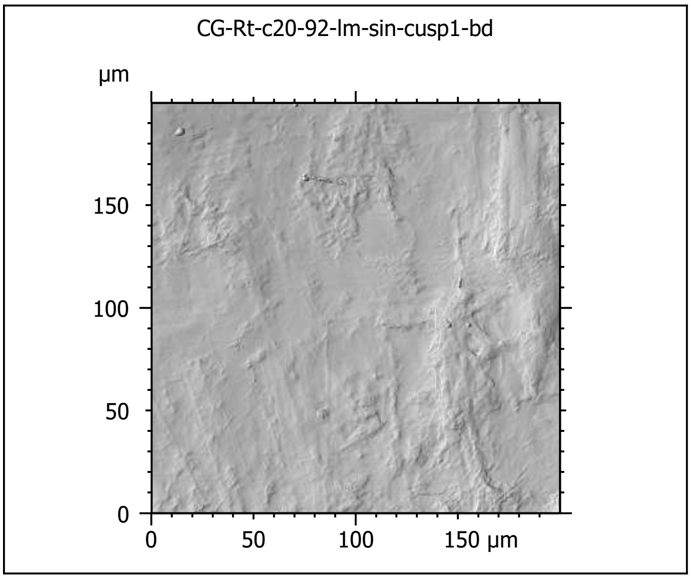

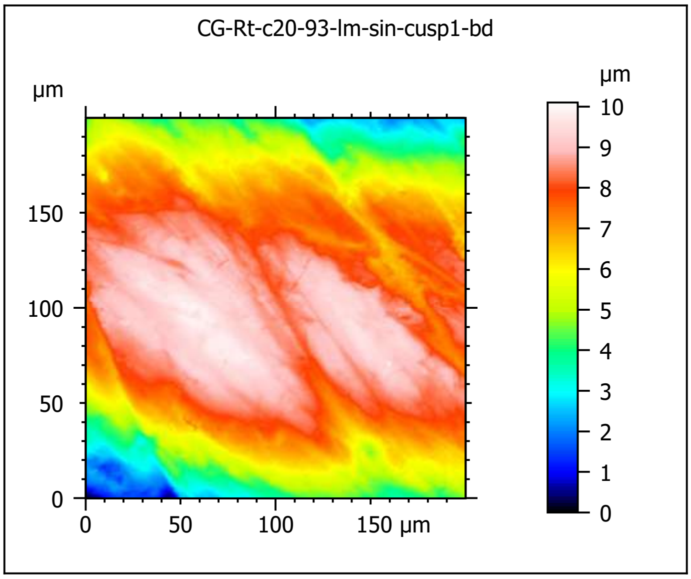

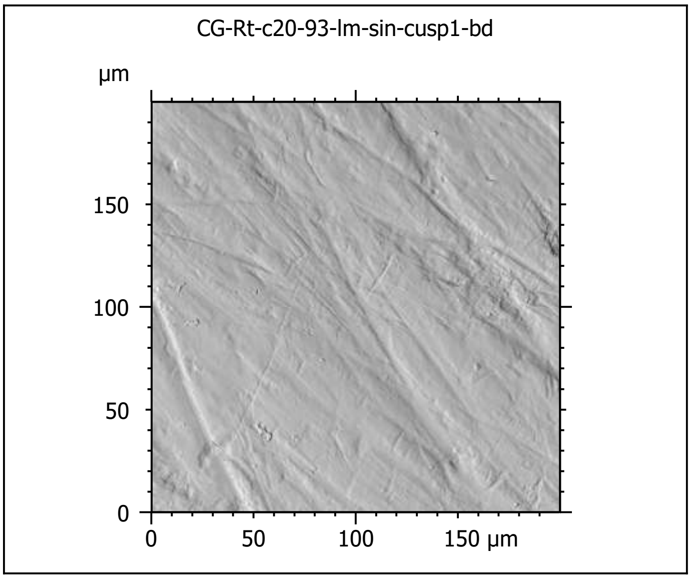

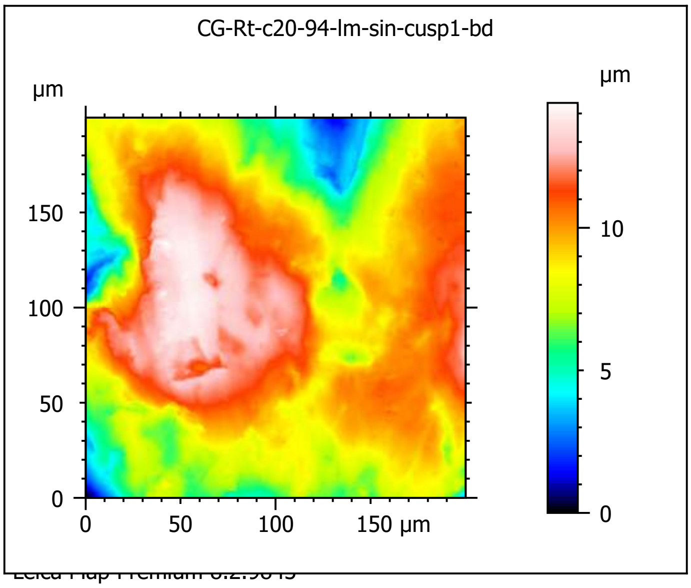

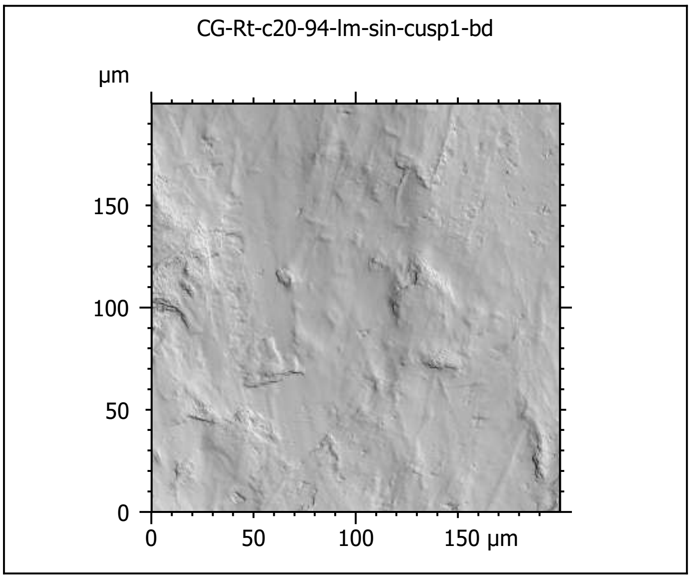

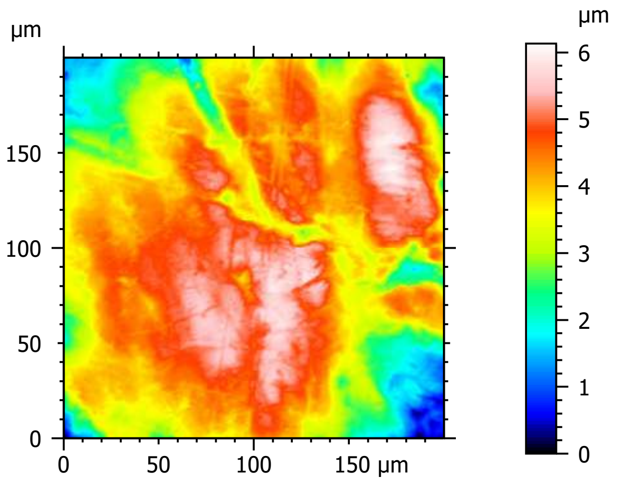

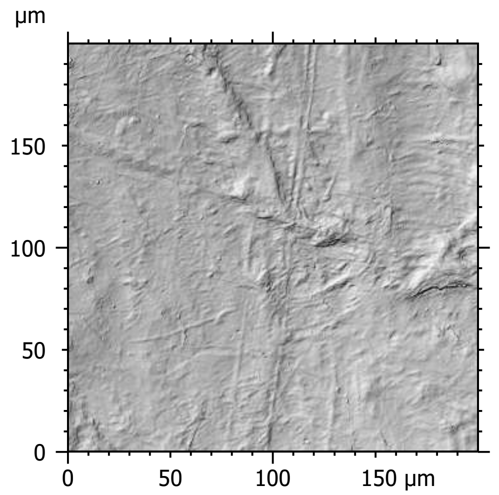

CG-Rt-c20-97-lm3-dex-cusp1-bd

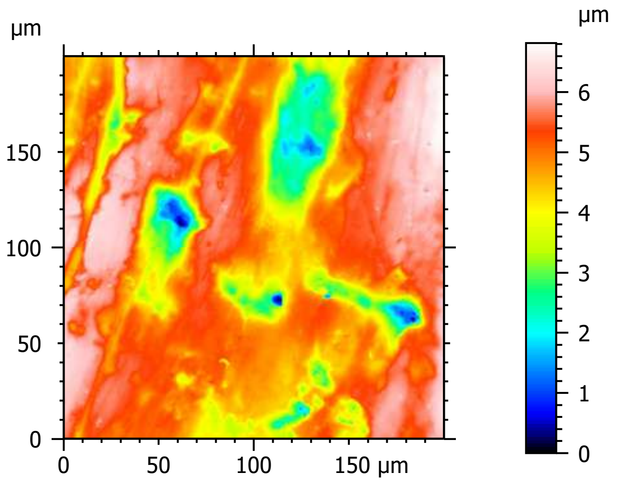

CG-Rt-c20-97-lm3-dex-cusp1-bd

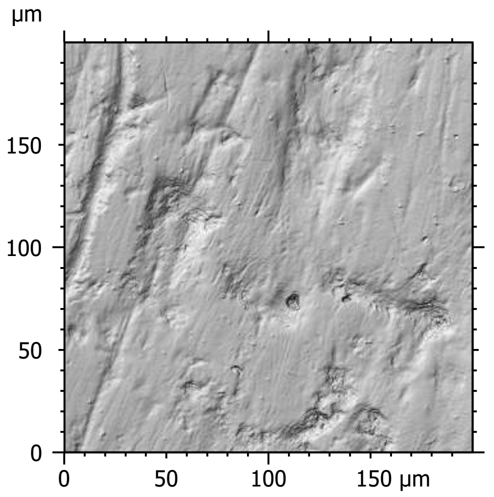

CG-Rt-c20-99-lm3-dex-cusp1-bd

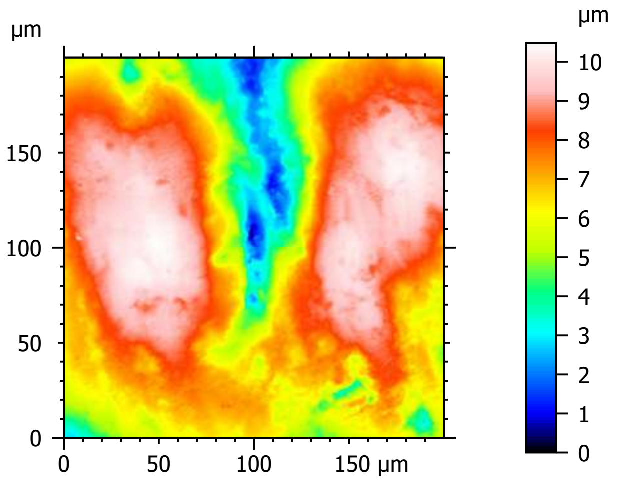

CG-Rt-c20-99-lm3-dex-cusp1-bd

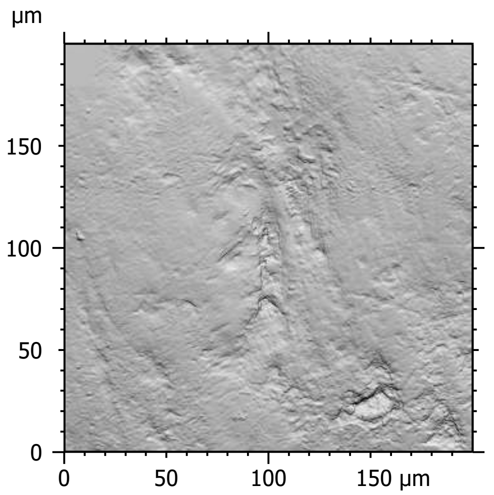

Leica Map Premium 8.2.9843

"A long-term perspective on Neandertal environment and subsistence: insights from the dental micro-texture analysis of hunted ungulates at Combe-Grenal (Dordogne, France)"

authors: Berlioz, E.; Capdepon, E.; Discamps, E.

Appendice 2:
surfaces scanned by E. Berlioz and E. Capdepon, pre-treatement by E. Berlioz and E. Capdepon, validation by E. Berlioz (2019)

***Rangifer tarandus* - Block F**

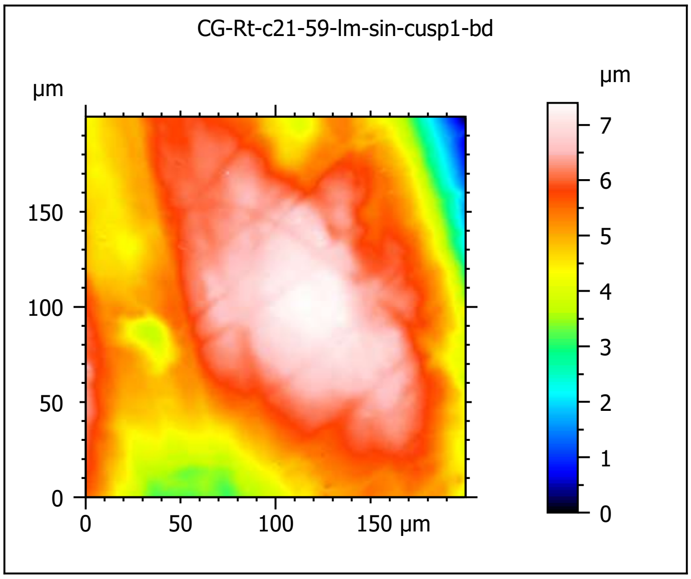

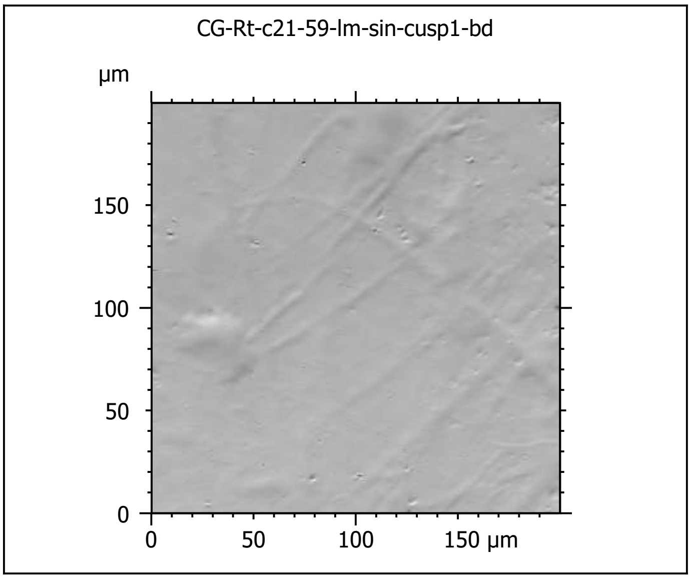

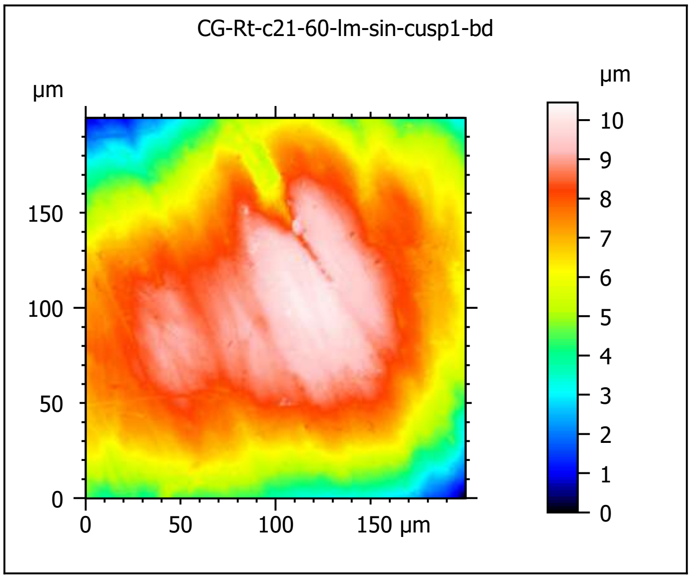

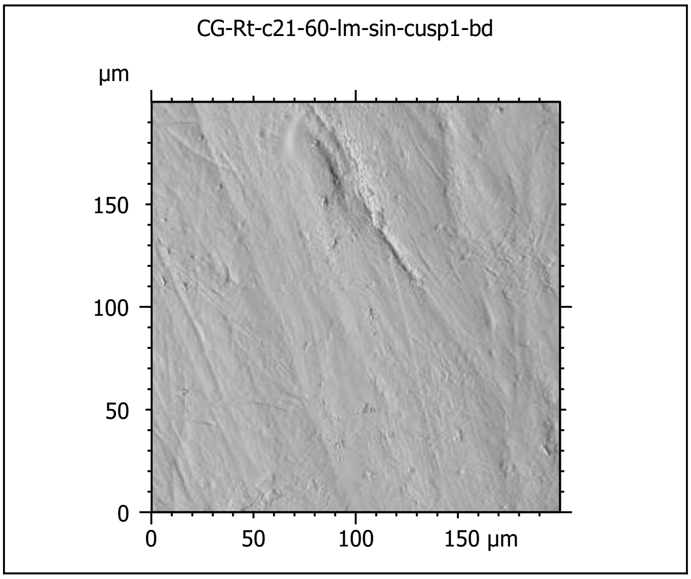

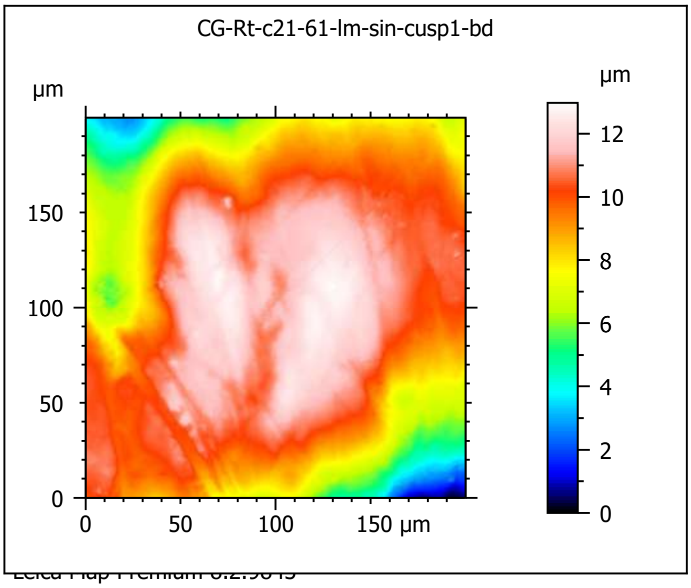

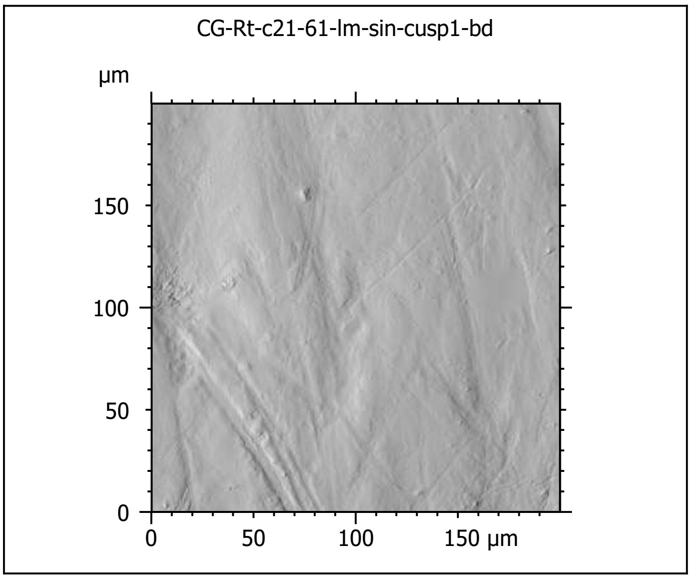

CG-Rt-c21-62-lm3-sin-cusp1-bd

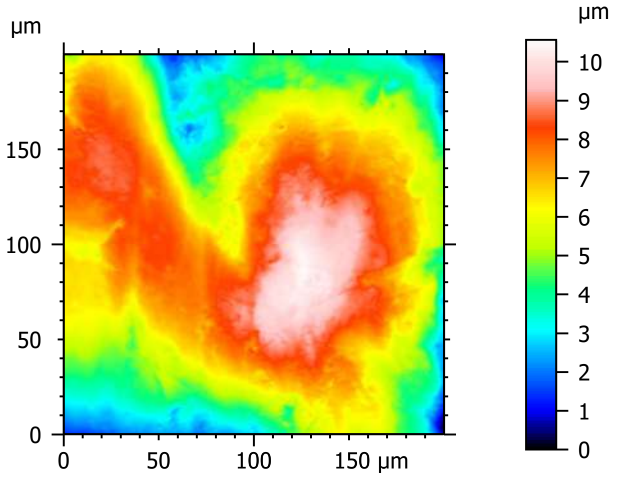

CG-Rt-c21-62-lm3-sin-cusp1-bd

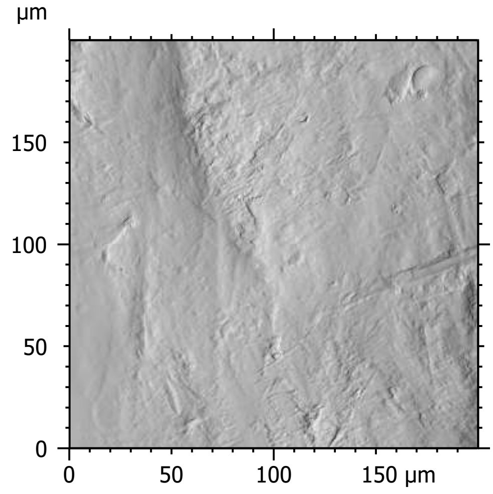

CG-Rt-c21-63-lm2-sin-cusp1-bd

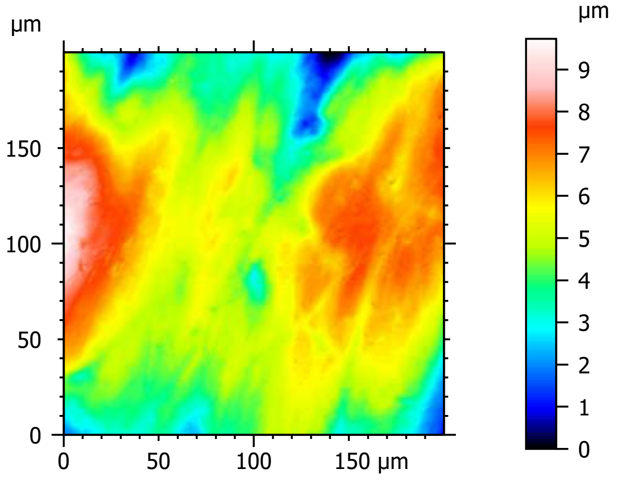

CG-Rt-c21-63-lm2-sin-cusp1-bd

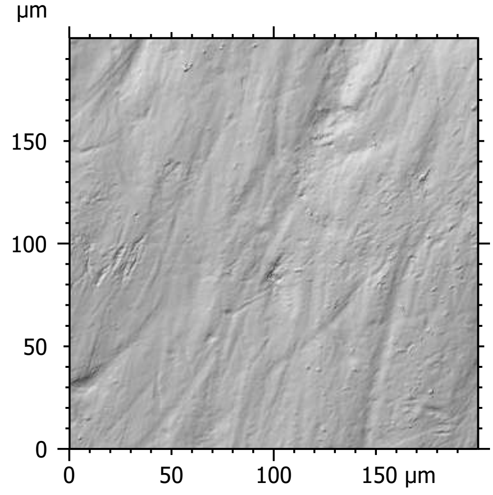

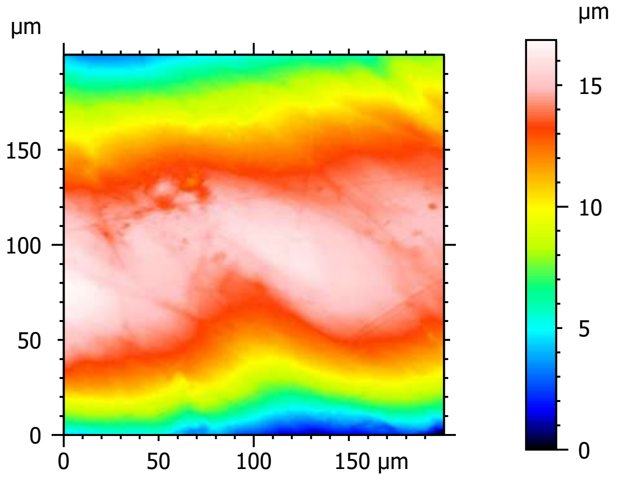

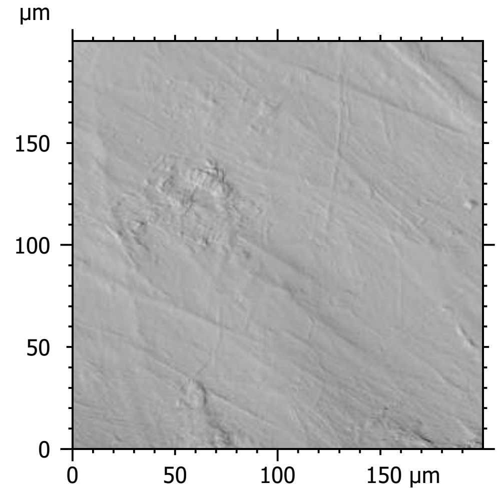

CG-Rt-c21-65-lm2-dex-cusp1-bd

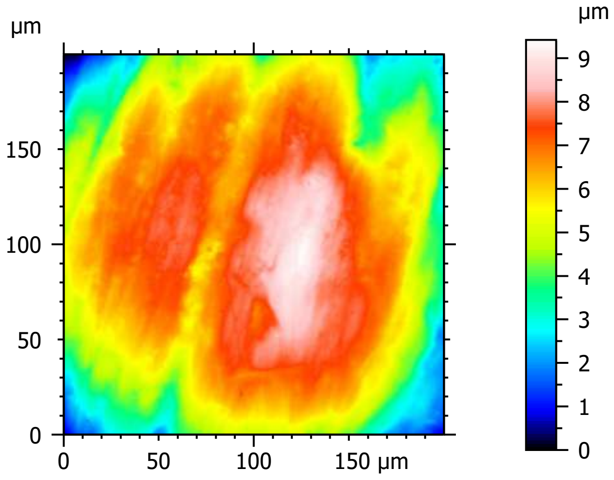

CG-Rt-c21-65-lm2-dex-cusp1-bd

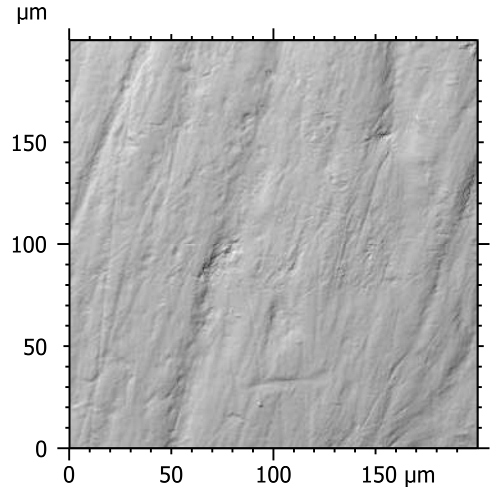

CG-Rt-c21-66-lm2-dex-cusp1-bd

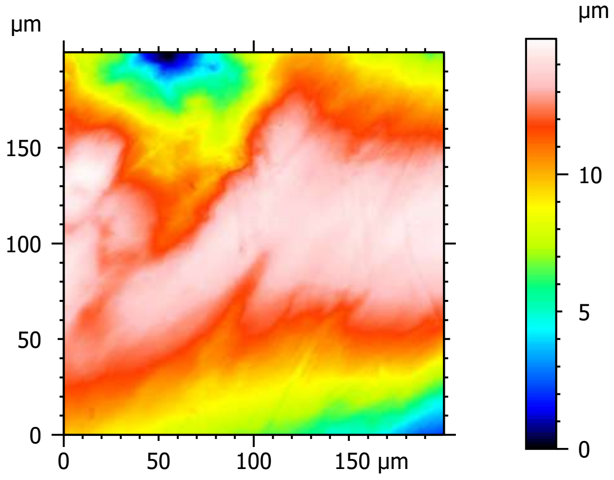

CG-Rt-c21-66-lm2-dex-cusp1-bd

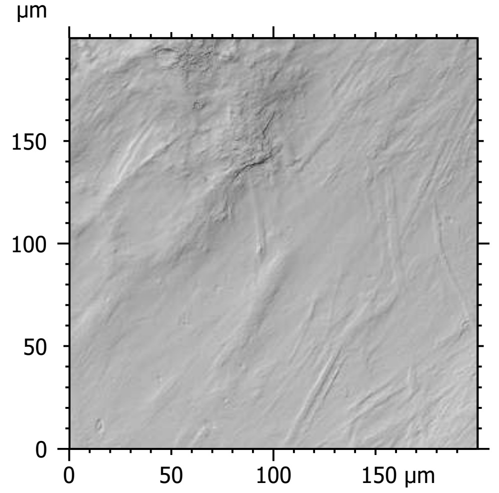

CG-Rt-c21-67-lm-sin-cusp1-bd

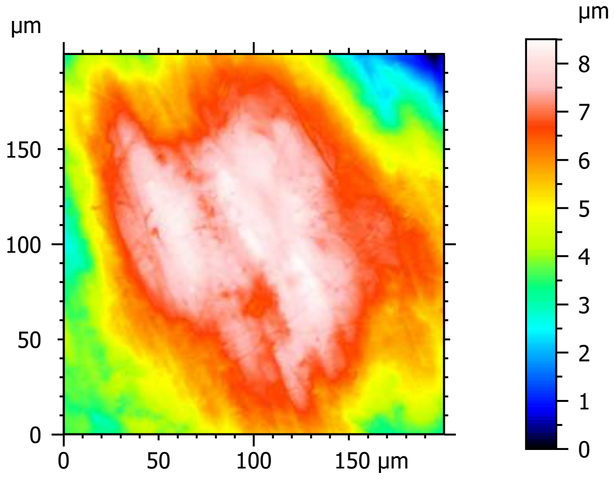

CG-Rt-c21-67-lm-sin-cusp1-bd

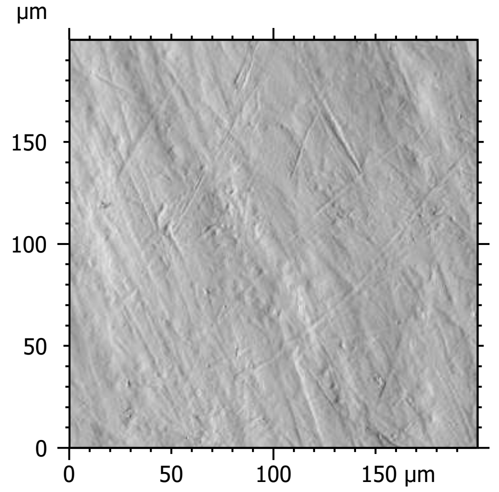

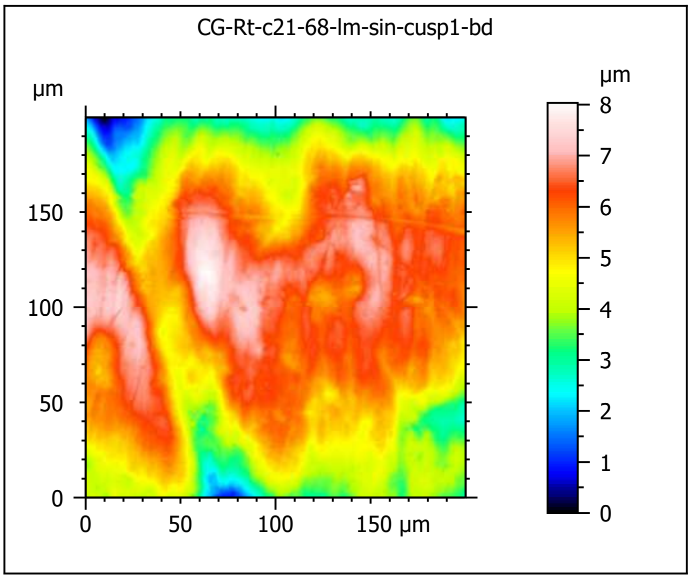

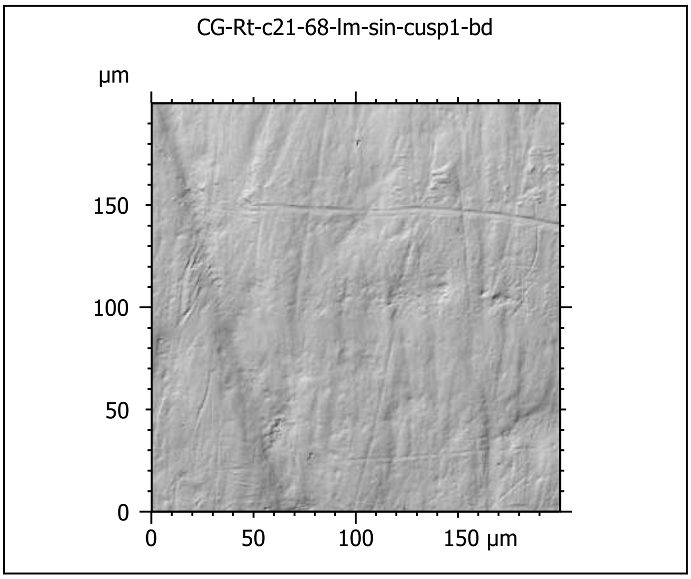

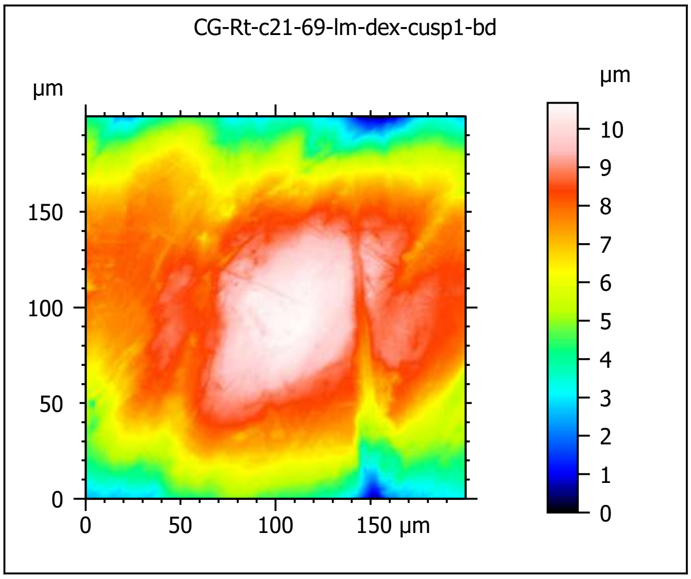

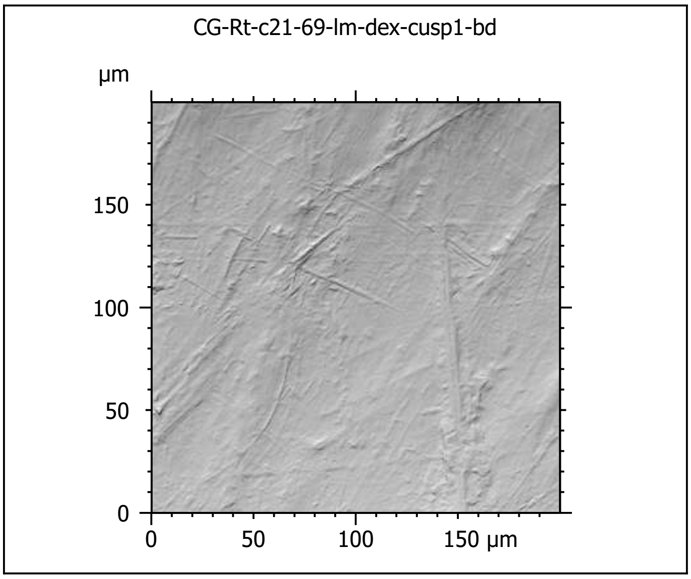

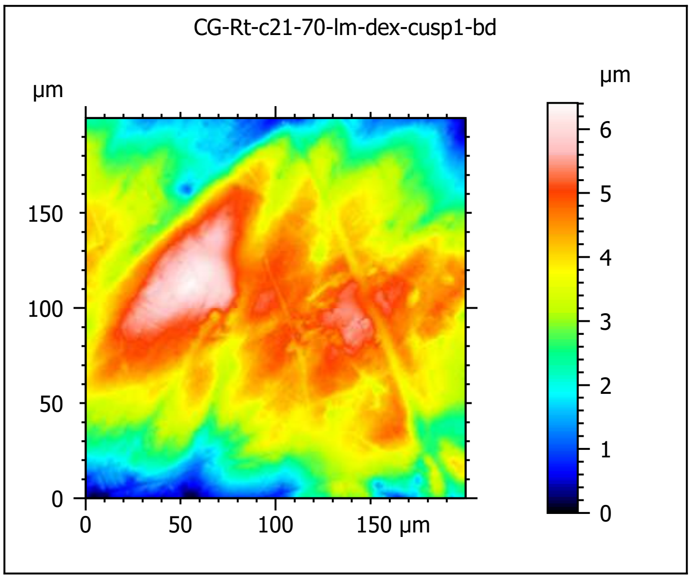

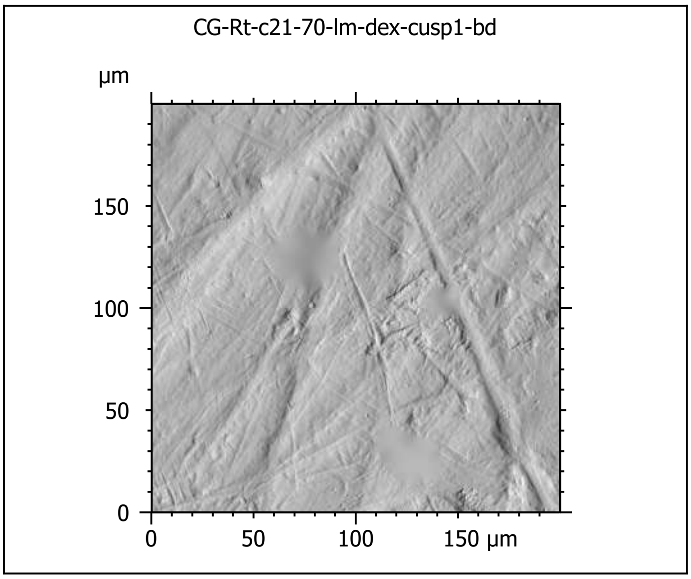

Leica Map Premium 8.2.9843

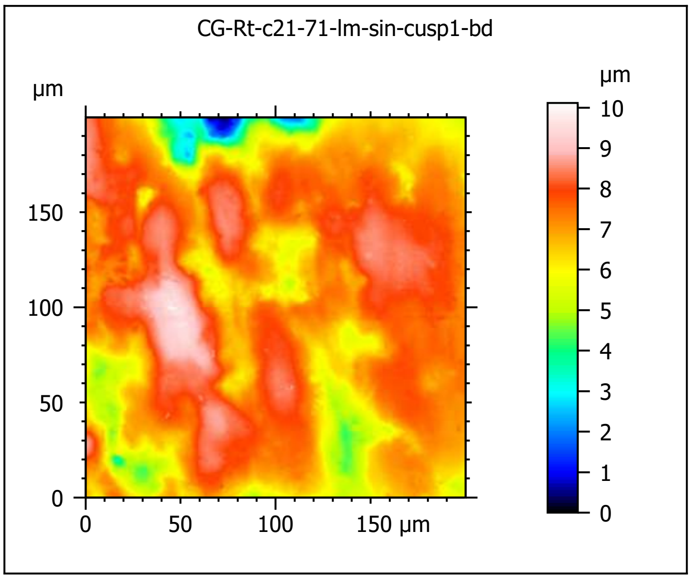

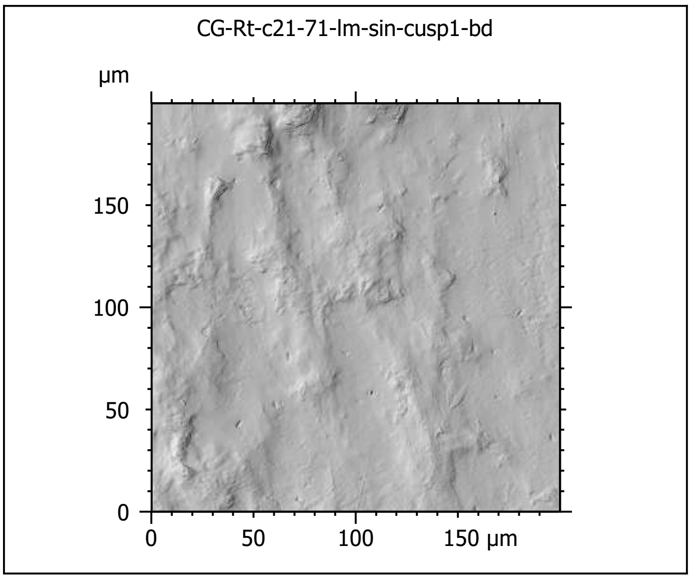

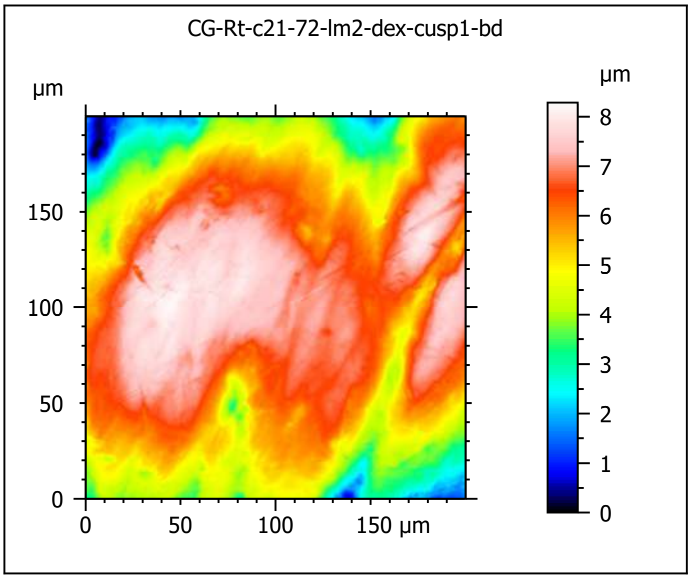

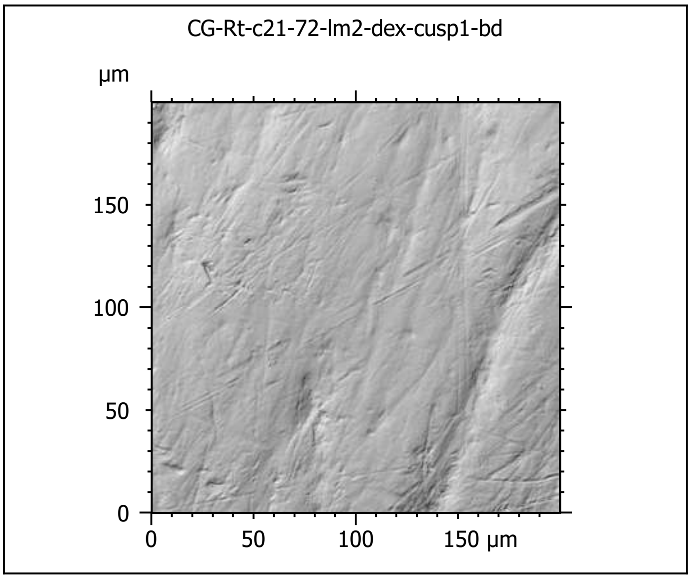

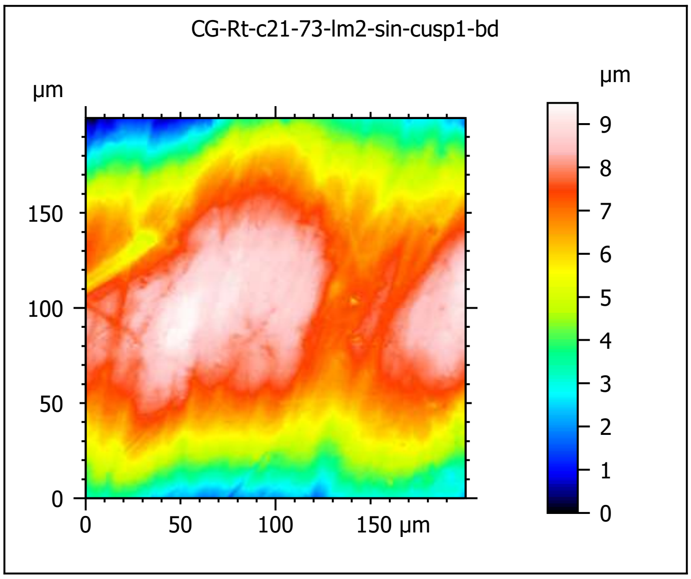

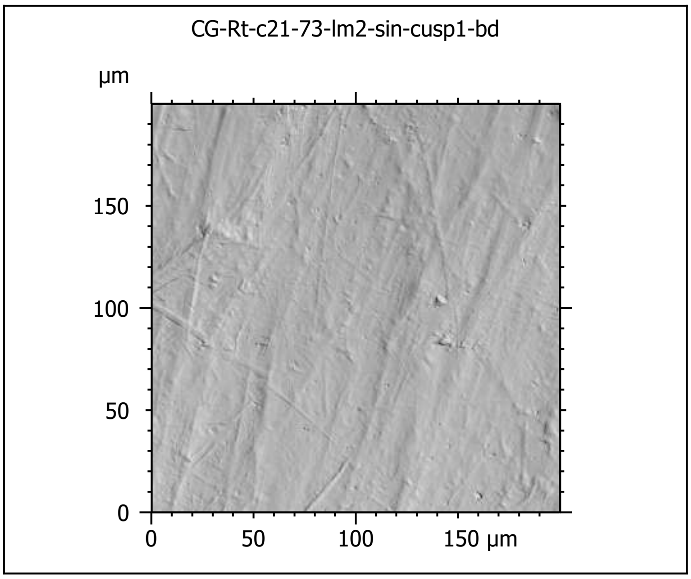

Leica Map Premium 8.2.9843

CG-Rt-c22-392-lm2-sin-cusp1-bd

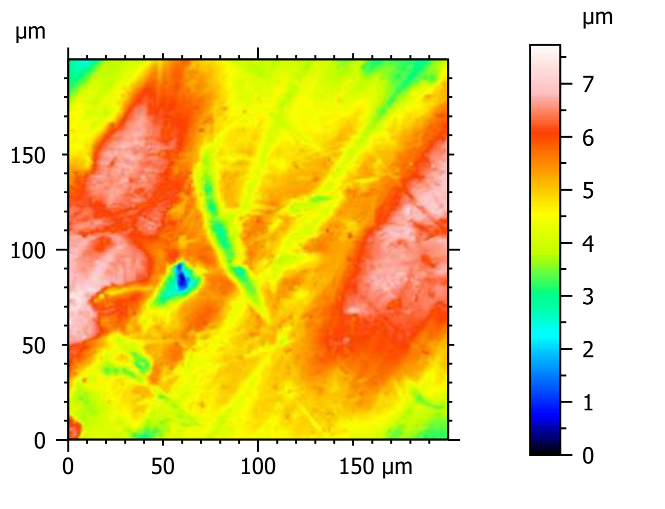

CG-Rt-c22-392-lm2-sin-cusp1-bd

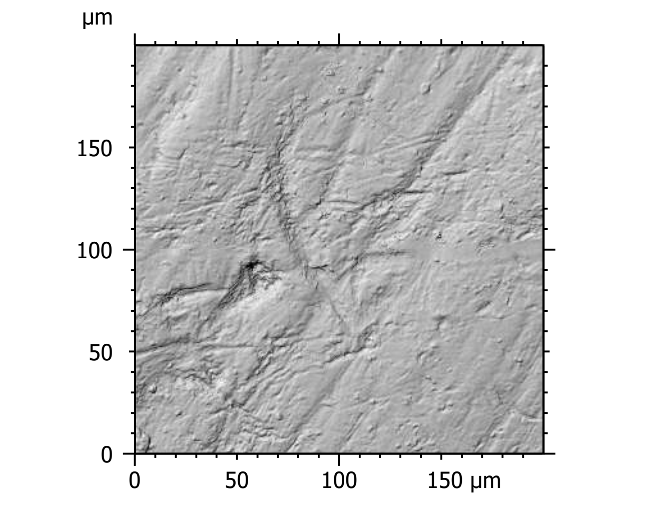

CG-Rt-c22-394-lm-sin-cusp1-bd

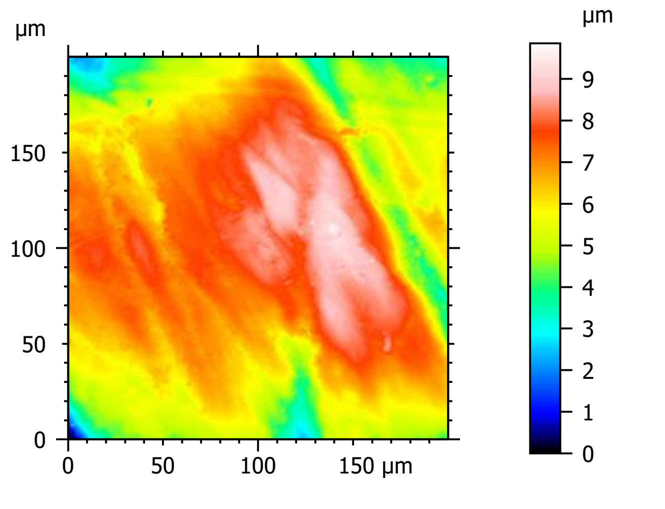

CG-Rt-c22-394-lm-sin-cusp1-bd

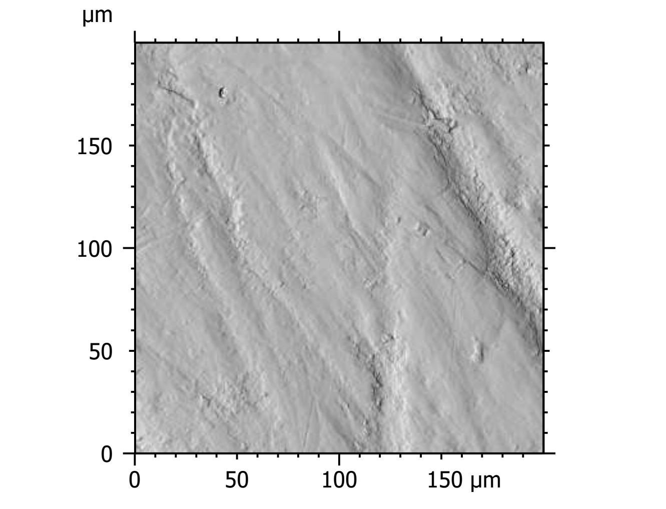

CG-Rt-c22-395-lm2-dex-cusp1-bd

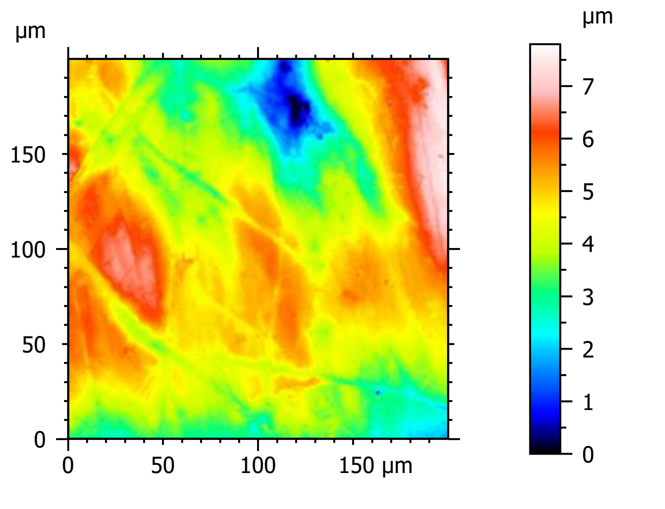

CG-Rt-c22-395-lm2-dex-cusp1-bd

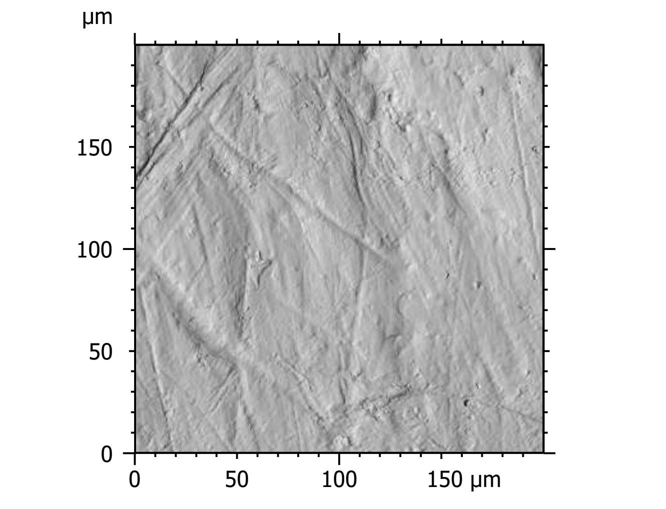

Leica Map Premium 8.2.9843

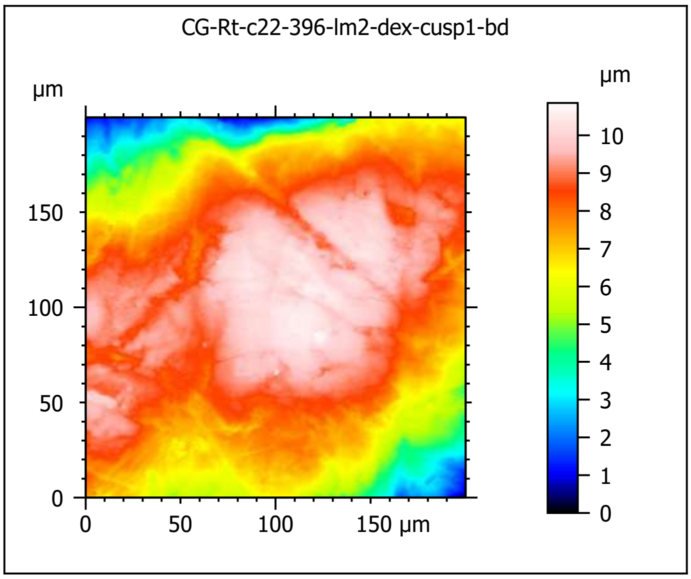

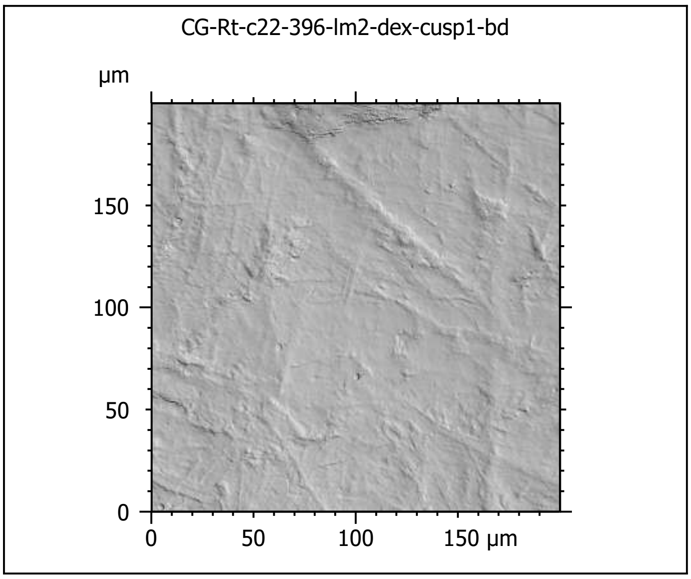

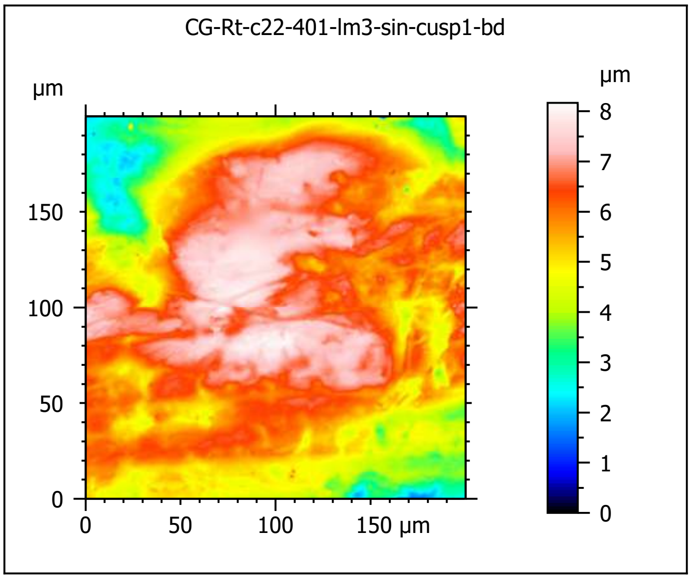

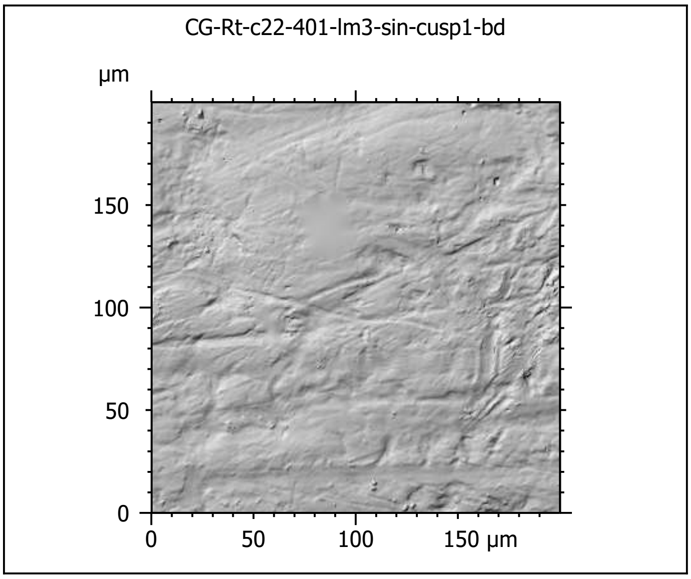

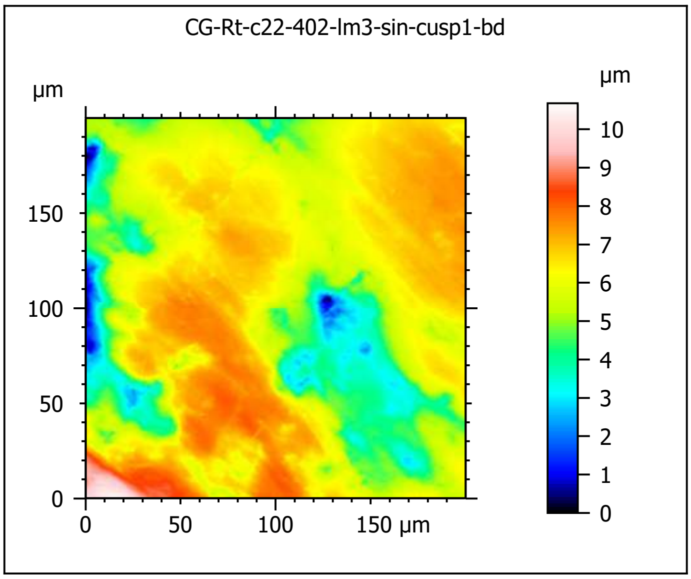

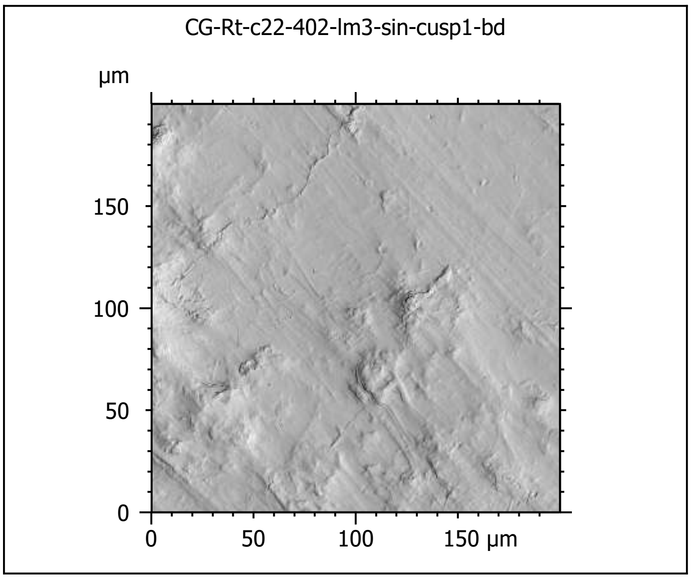

Leica Map Premium 8.2.9843

CG-Rt-c22-404-lm3-sin-cusp1-bd

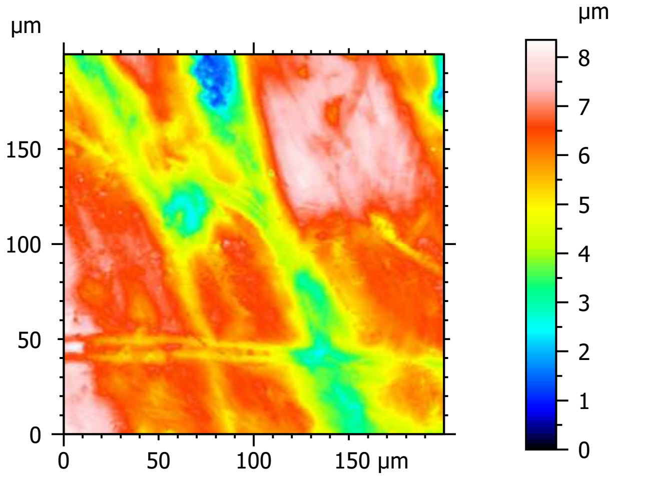

CG-Rt-c22-404-lm3-sin-cusp1-bd

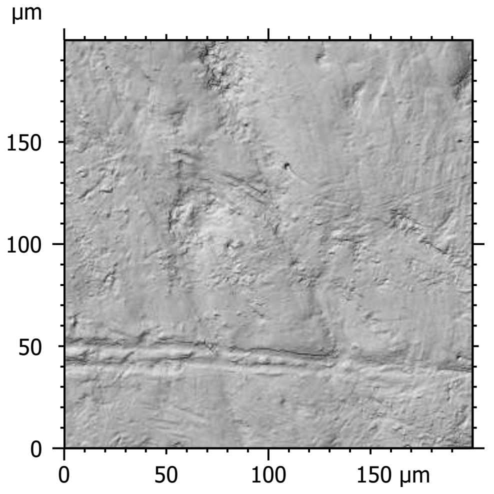

CG-Rt-c22-405-lm2-sin-cusp1-bd

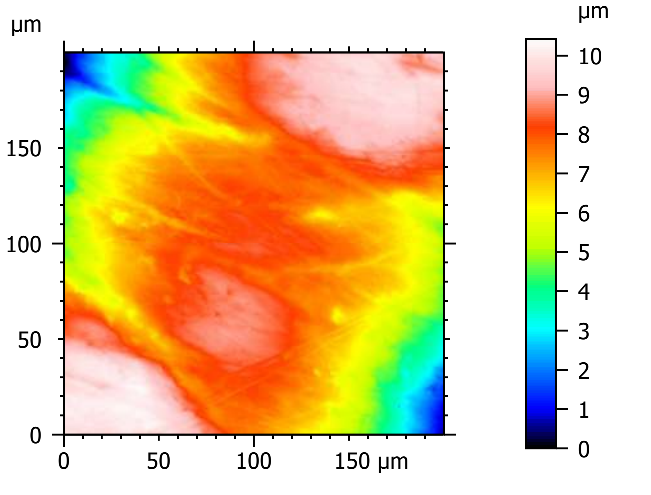

CG-Rt-c22-405-lm2-sin-cusp1-bd

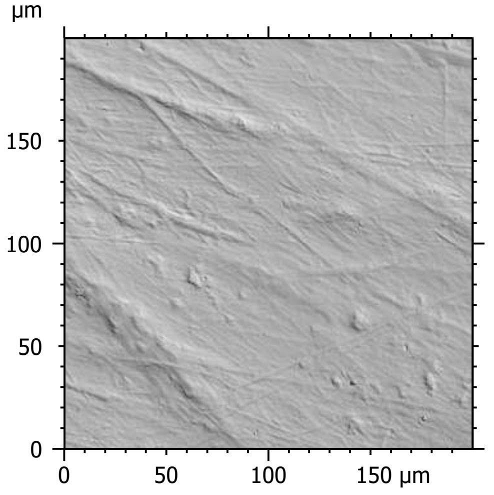

CG-Rt-c22-406-lm3-sin-cusp1-bd

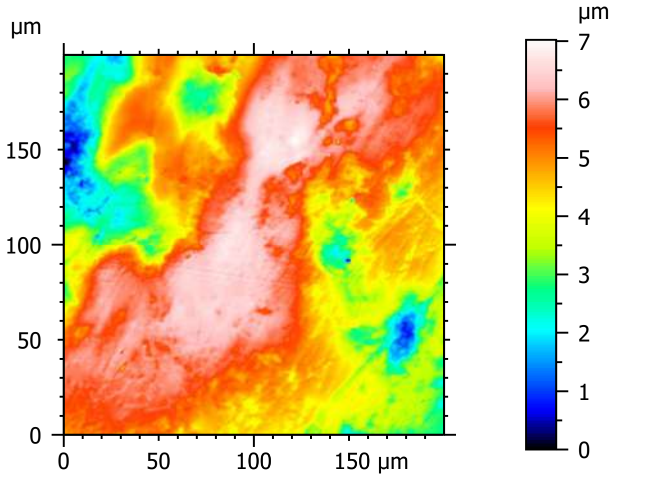

CG-Rt-c22-406-lm3-sin-cusp1-bd

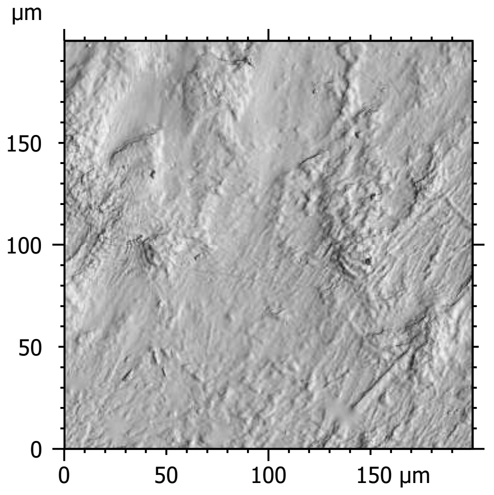

Leica Map Premium 8.2.9843

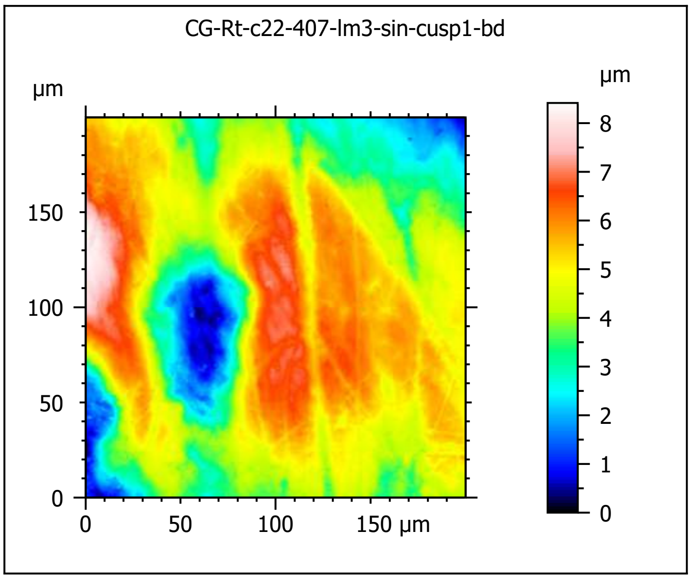

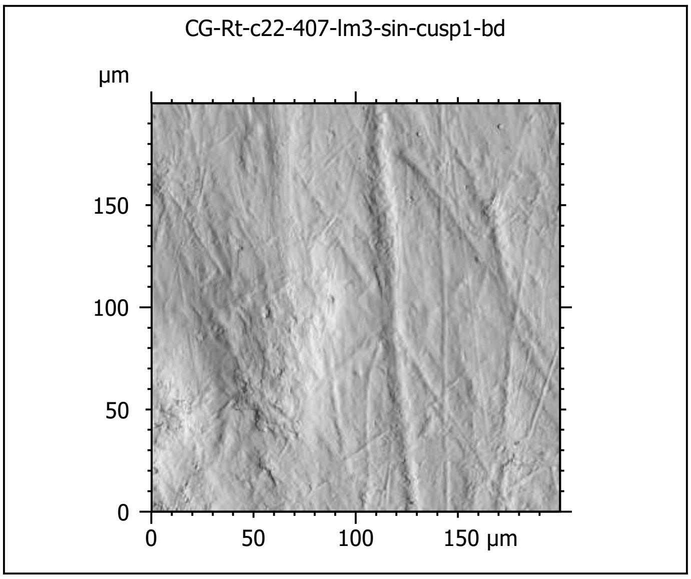

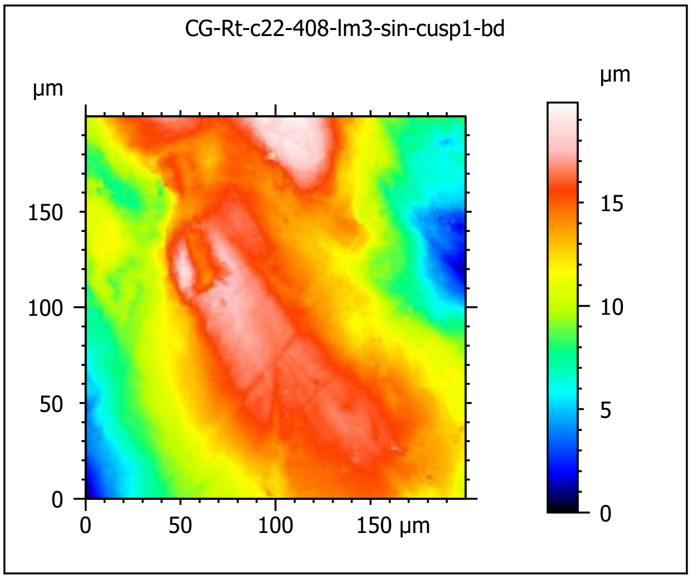

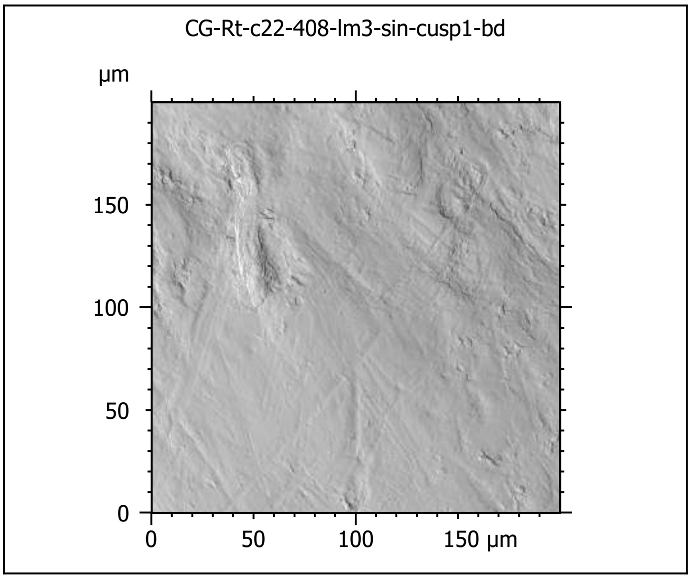

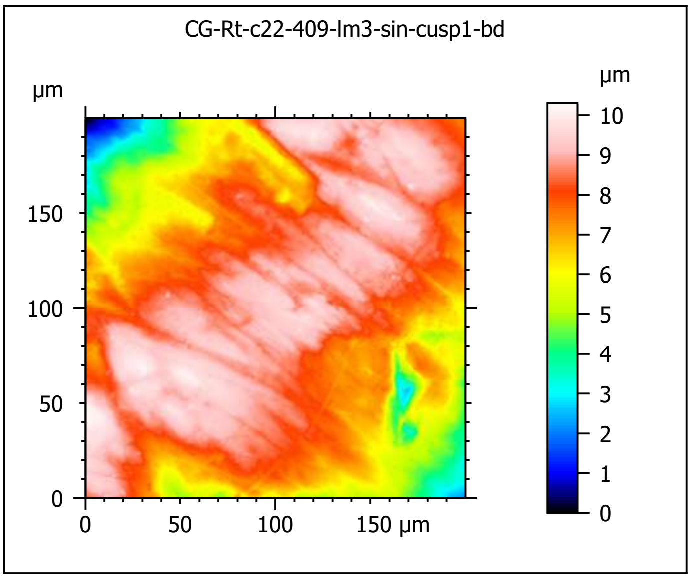

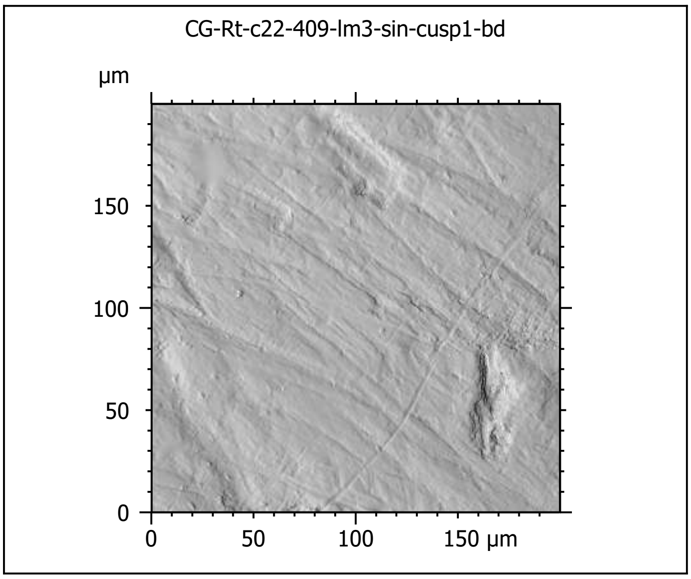

Leica Map Premium 8.2.9843

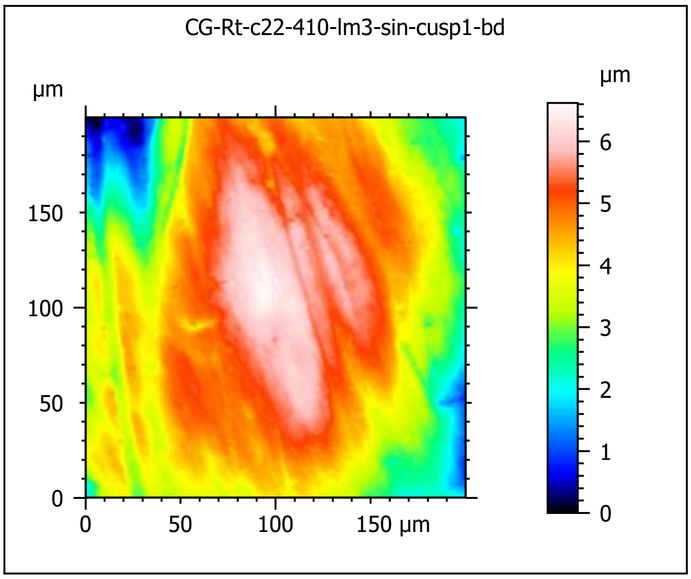

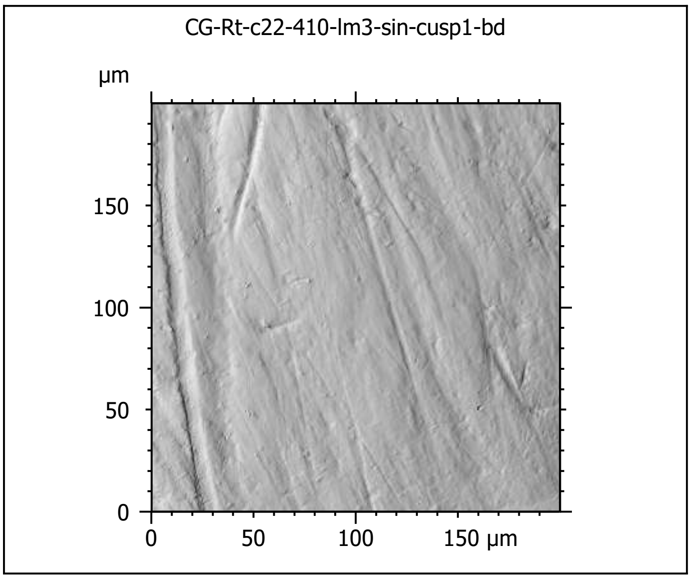

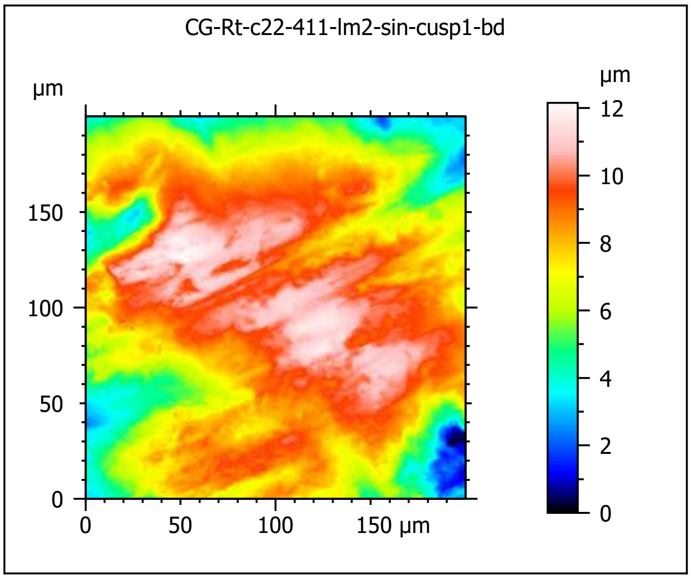

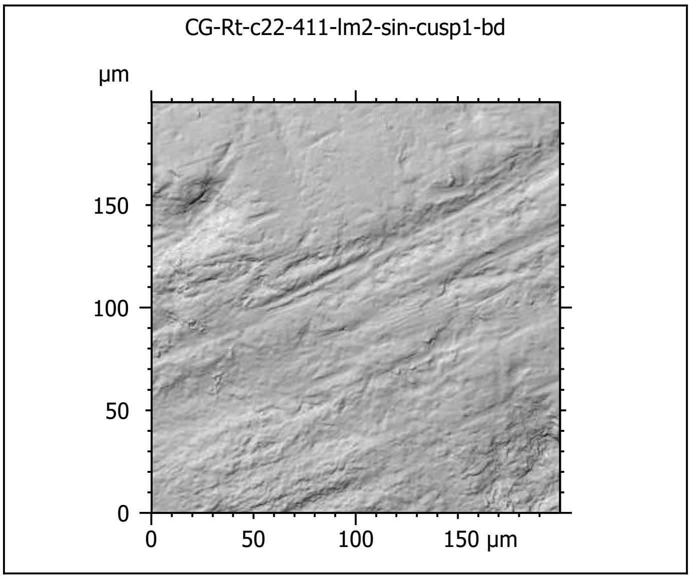

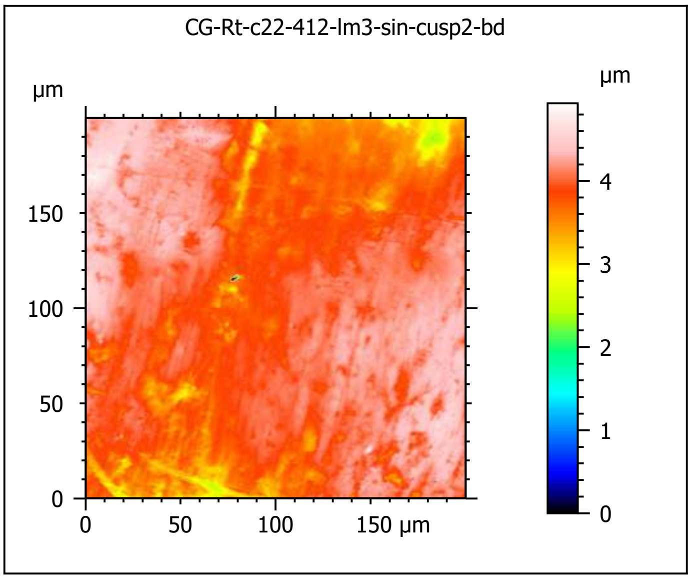

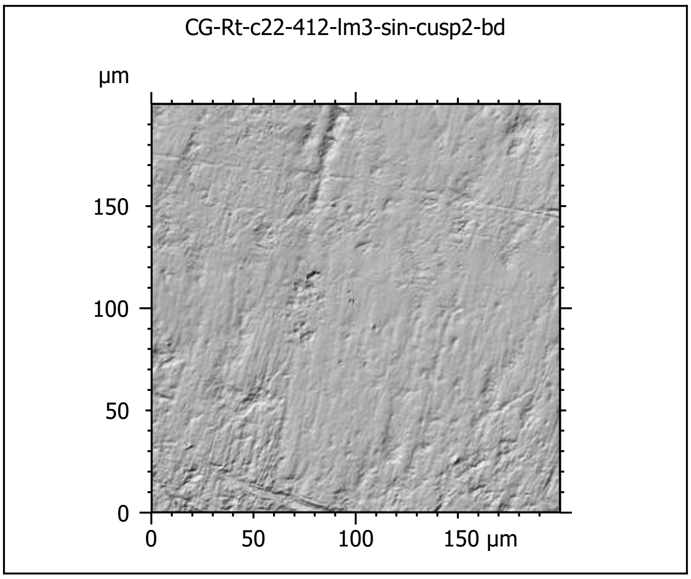

Leica Map Premium 8.2.9843

CG-Rt-c22-415-lm3-sin-cusp2-bd

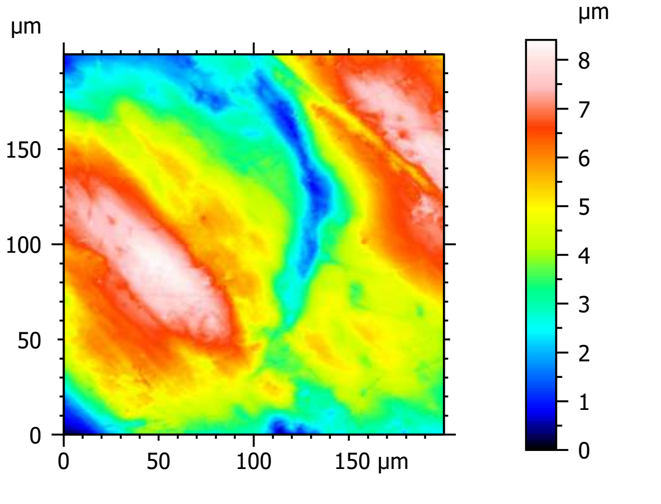

CG-Rt-c22-415-lm3-sin-cusp2-bd

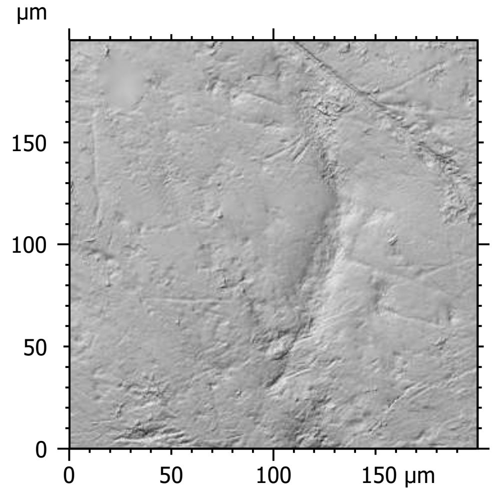

CG-Rt-c22-414-lm3-sin-cusp1-bd

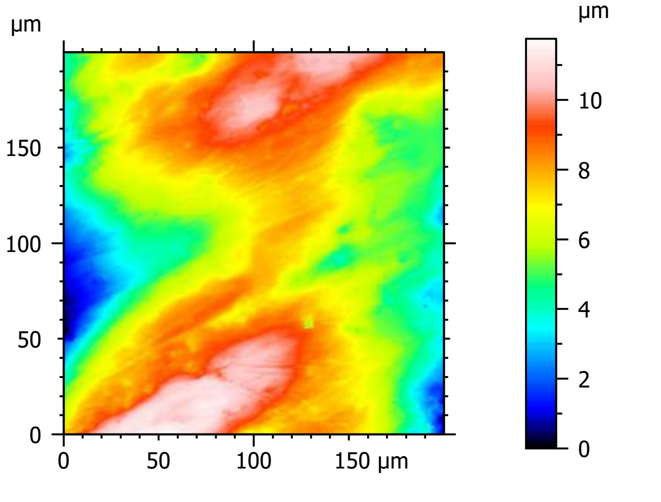

CG-Rt-c22-414-lm3-sin-cusp1-bd

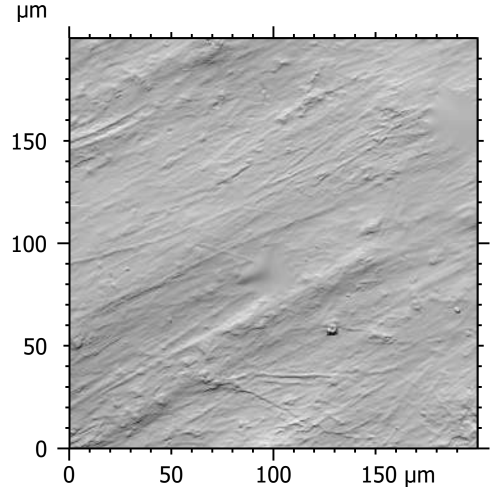

CG-Rt-c22-416-lm3-sin-cusp1-bd

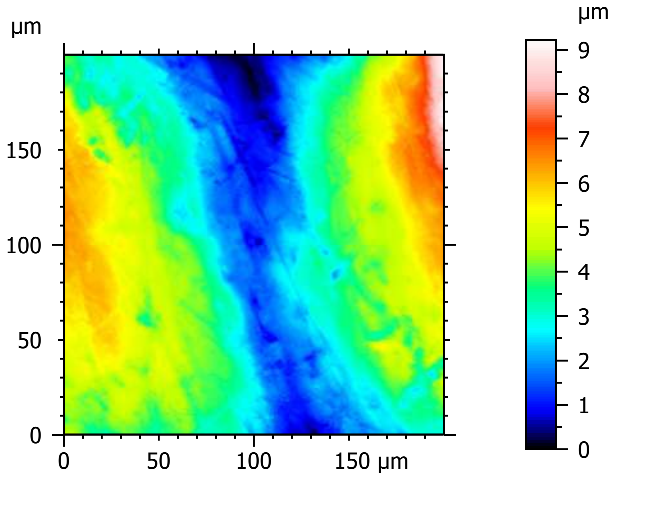

CG-Rt-c22-416-lm3-sin-cusp1-bd

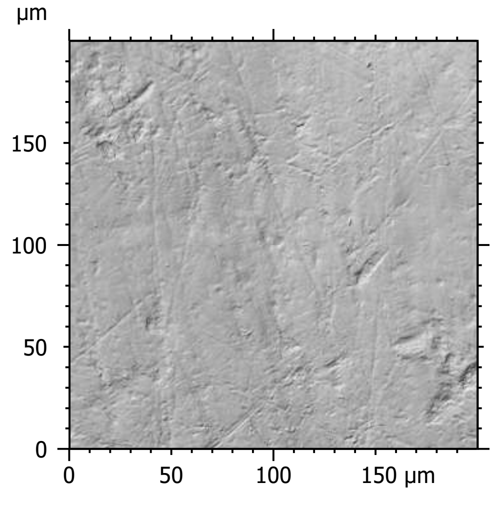

Leica Map Premium 8.2.9843

CG-Rt-c22-417-lm3-sin-cusp1-bd

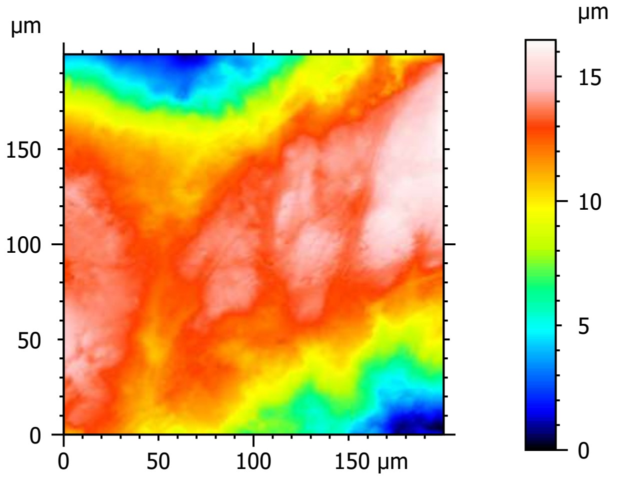

CG-Rt-c22-417-lm3-sin-cusp1-bd

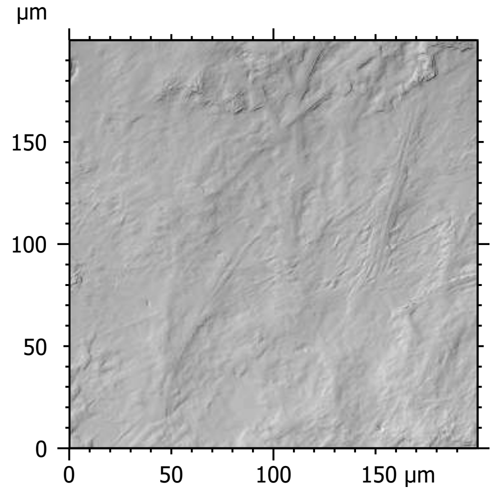

CG-Rt-c22-419-lm3-sin-cusp1-bd

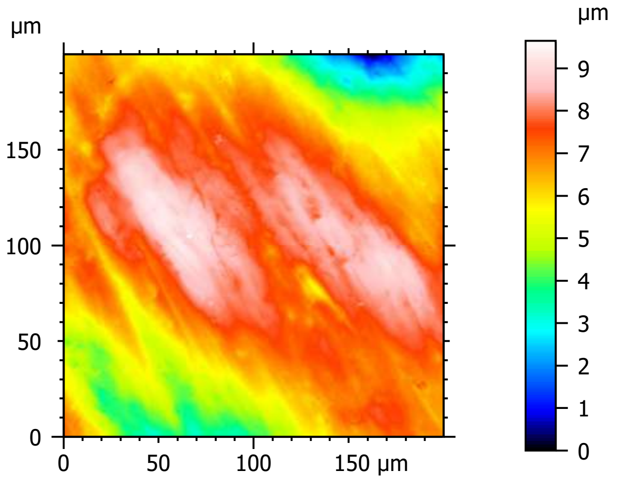

CG-Rt-c22-419-lm3-sin-cusp1-bd

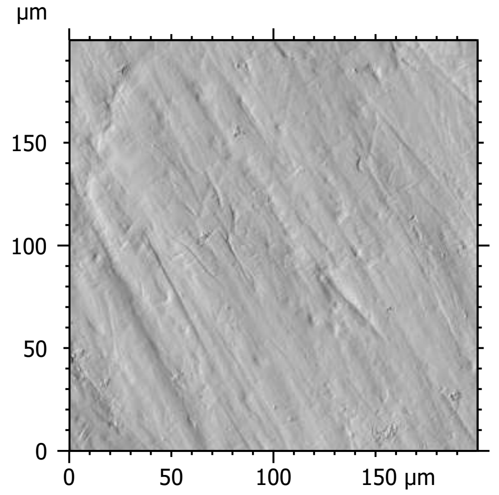

CG-Rt-c22-420-lm3-sin-cusp2-bd

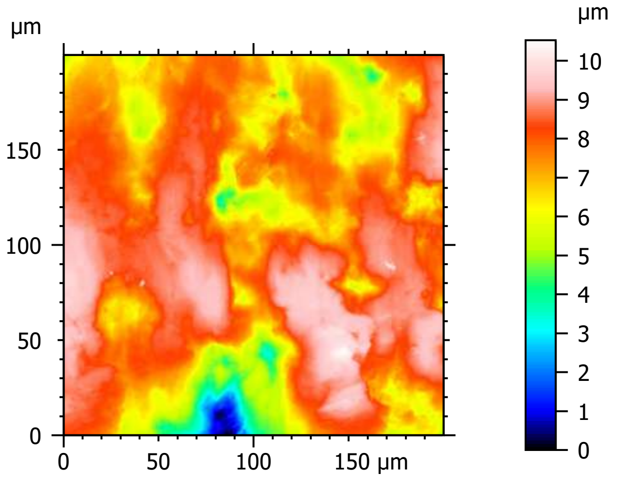

CG-Rt-c22-420-lm3-sin-cusp2-bd

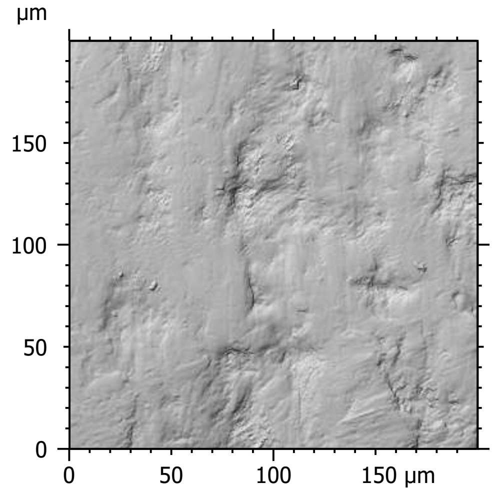

Leica Map Premium 8.2.9843

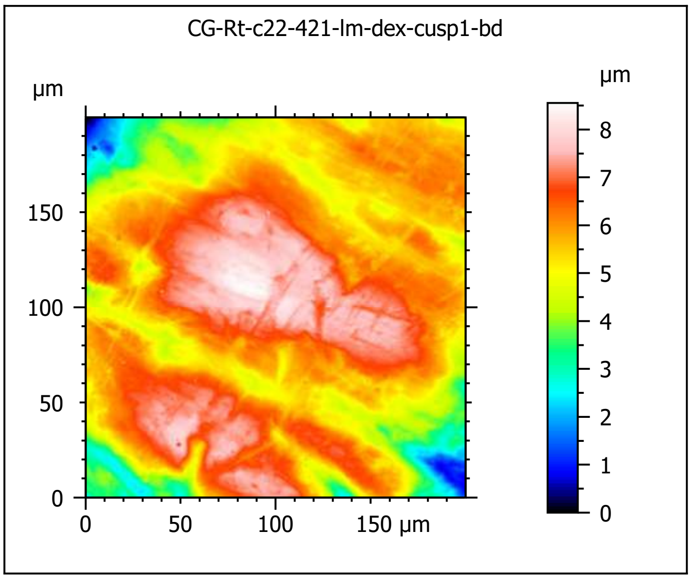

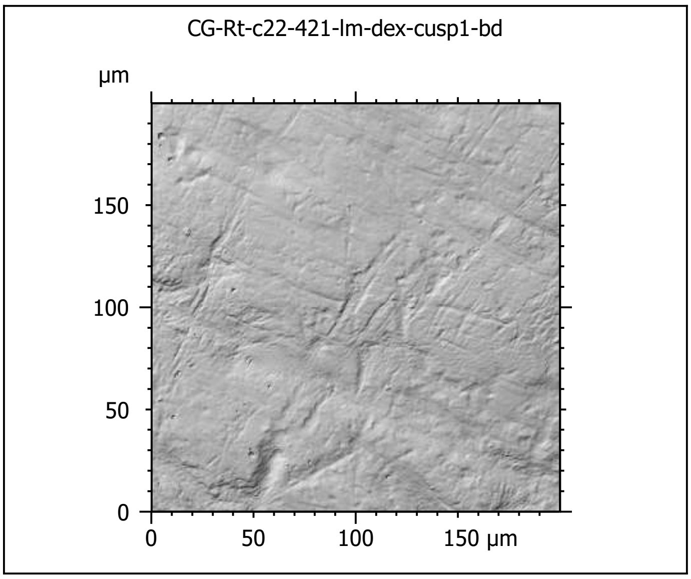

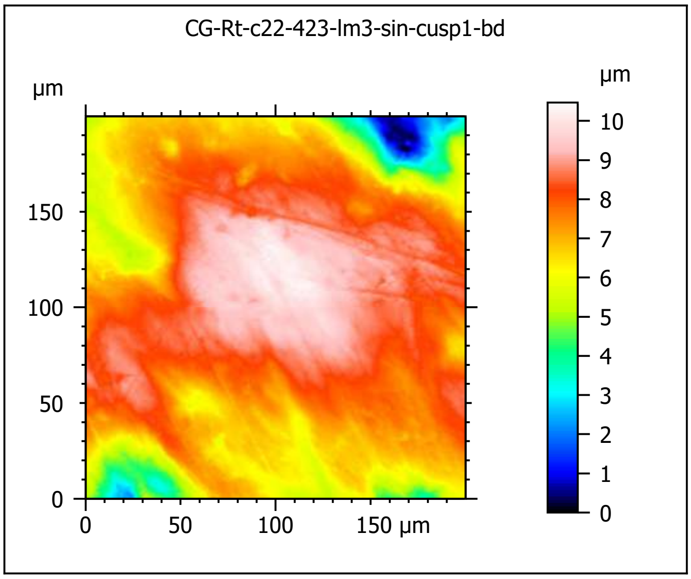

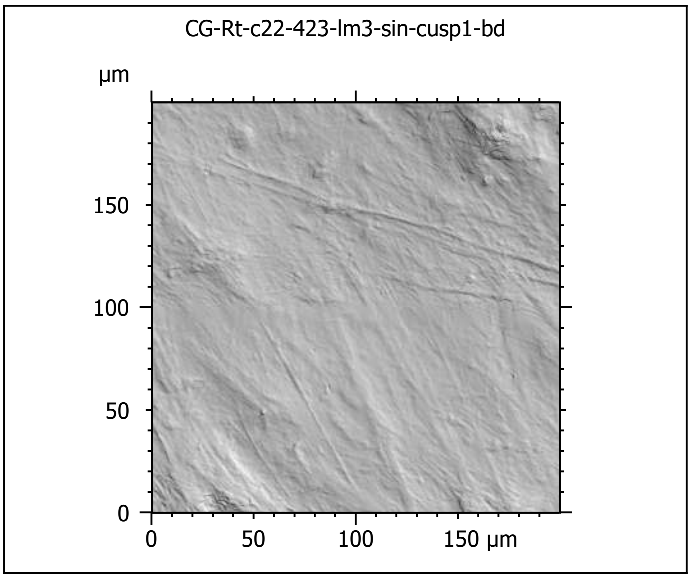

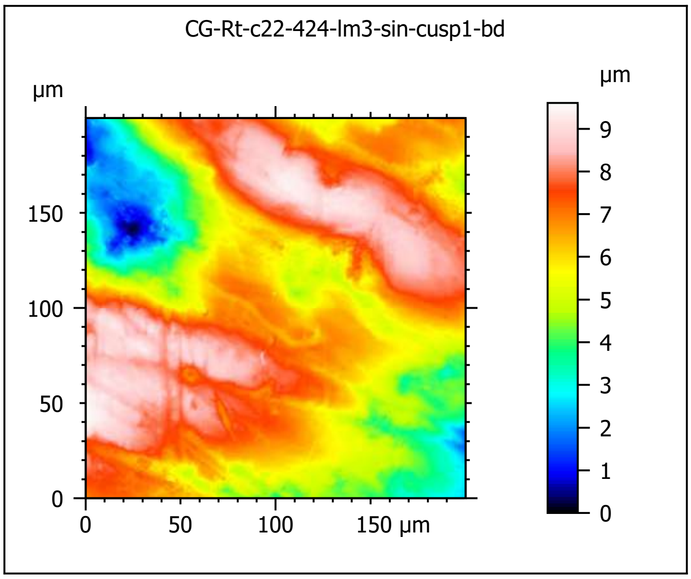

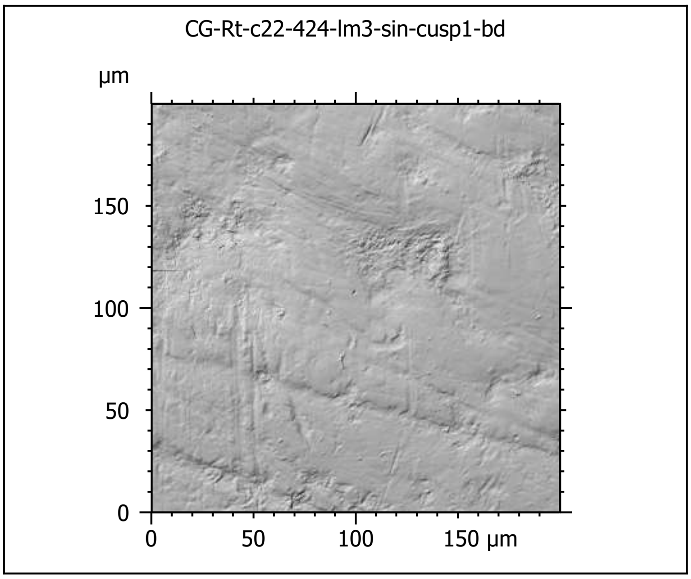

Leica Map Premium 8.2.9843

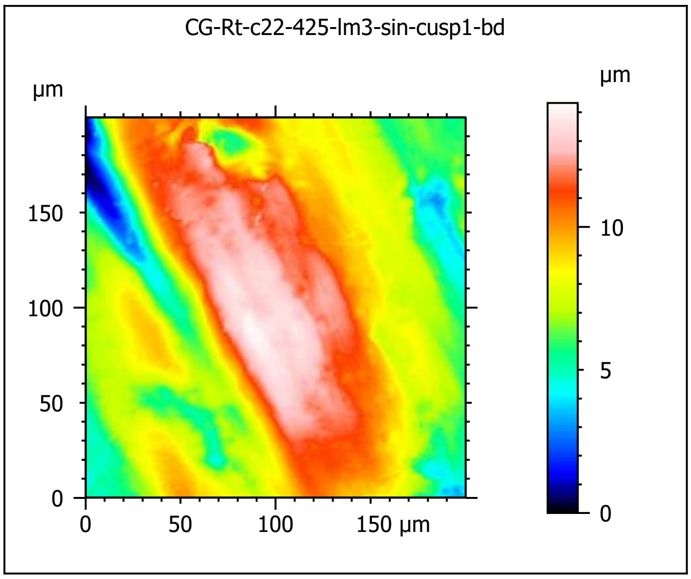

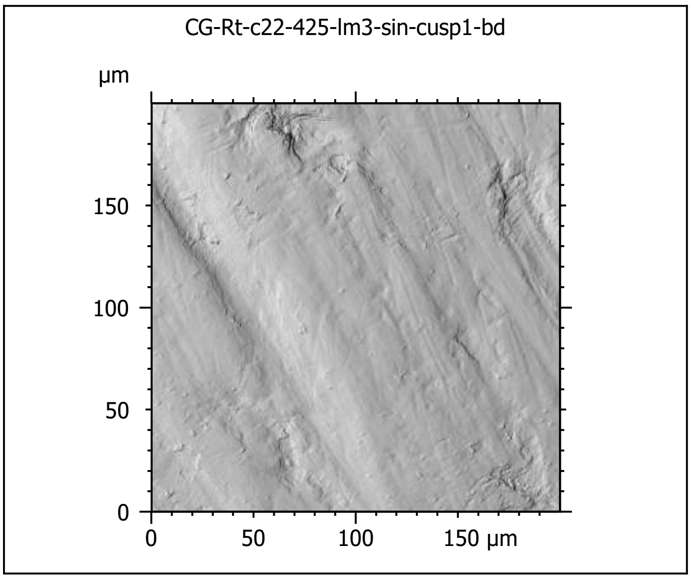

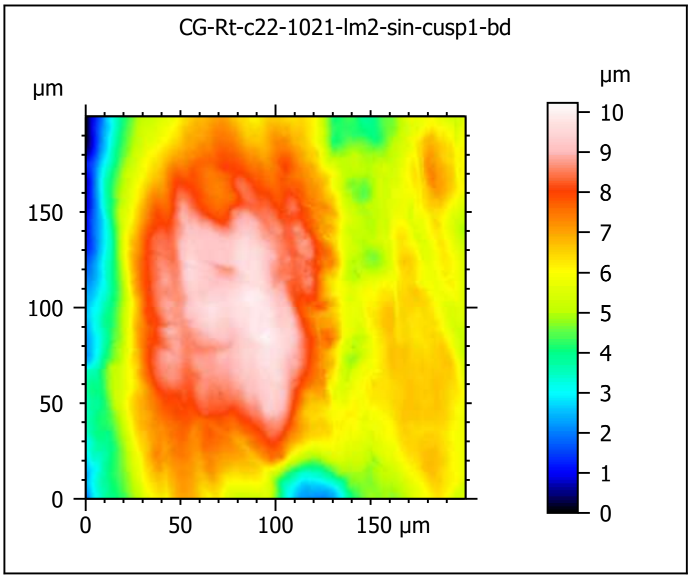

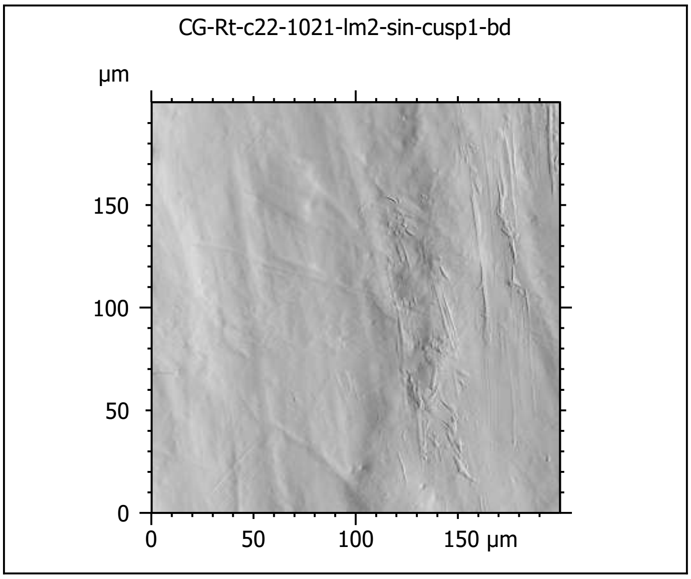

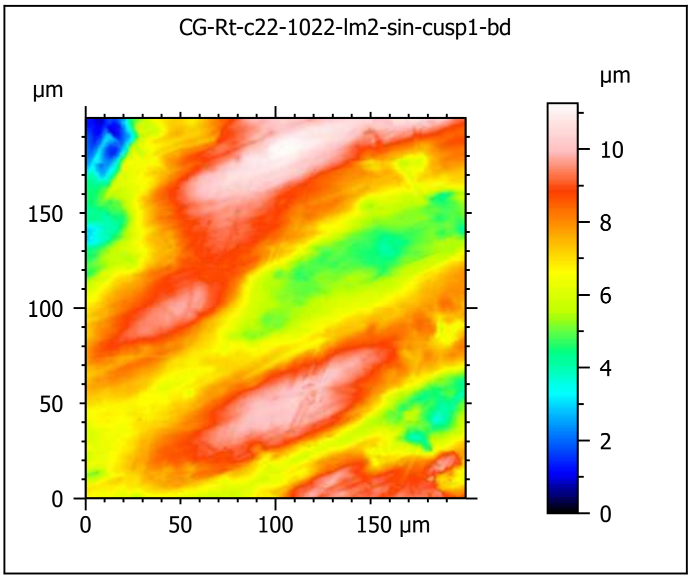

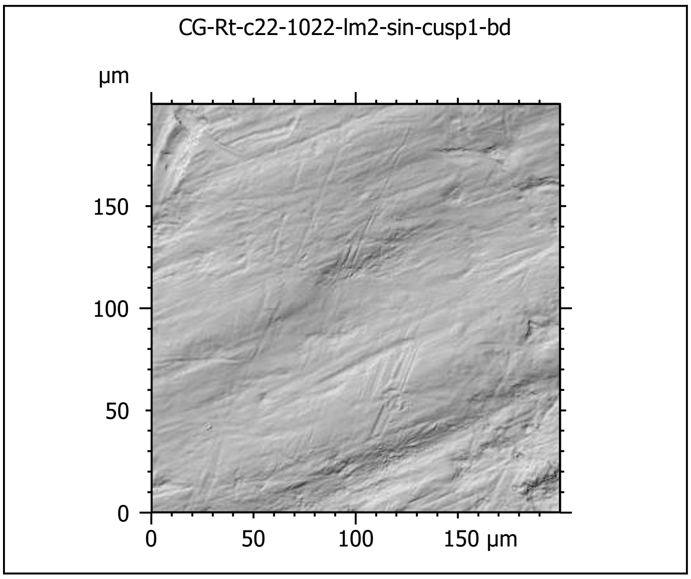

Leica Map Premium 8.2.9843

CG-Rt-c23-301-lm3-dex-cusp1-bd

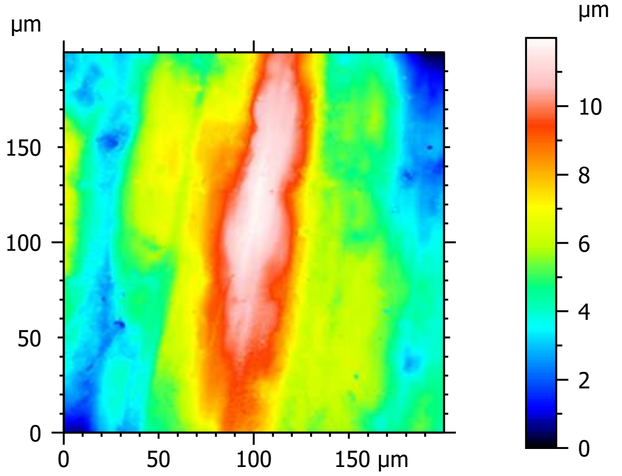

CG-Rt-c23-301-lm3-dex-cusp1-bd

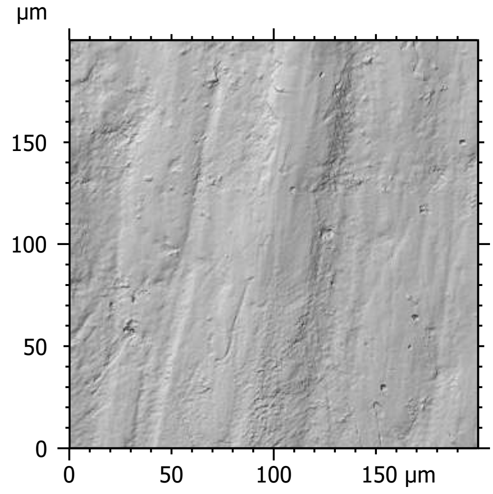

CG-Rt-c23-302-lm3-dex-cusp1-bd

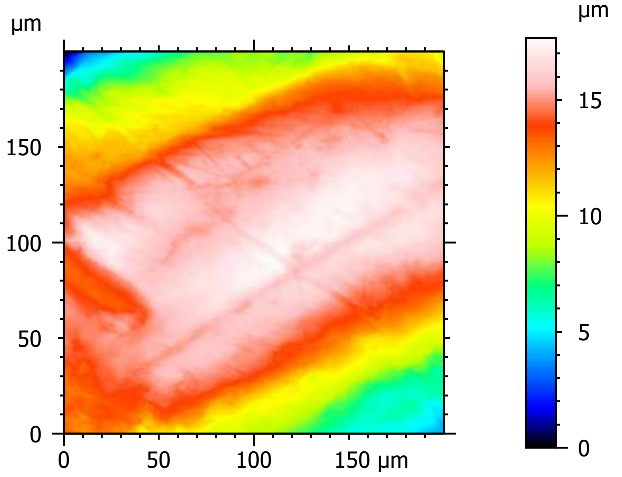

CG-Rt-c23-302-lm3-dex-cusp1-bd

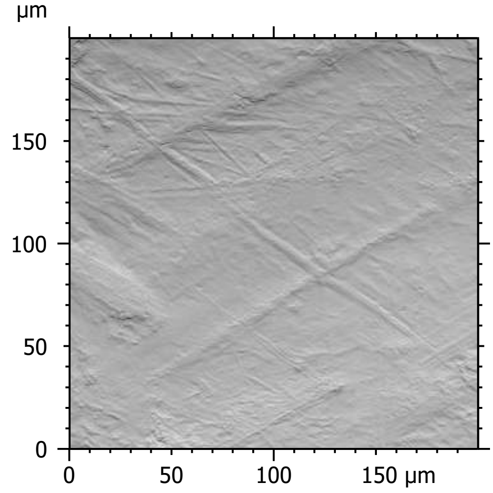

CG-Rt-c23-303-lm3-dex-cusp1-bd

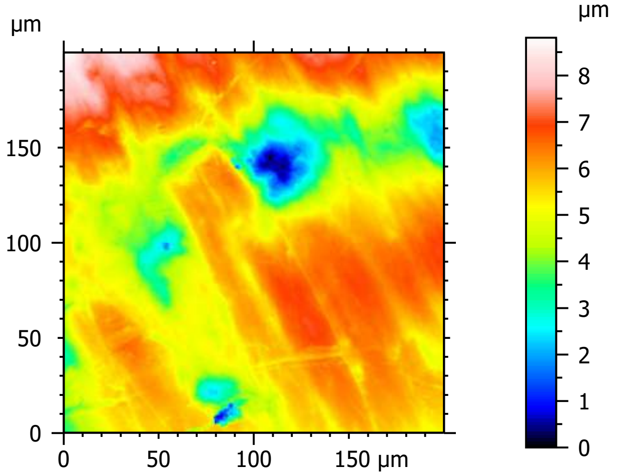

CG-Rt-c23-303-lm3-dex-cusp1-bd

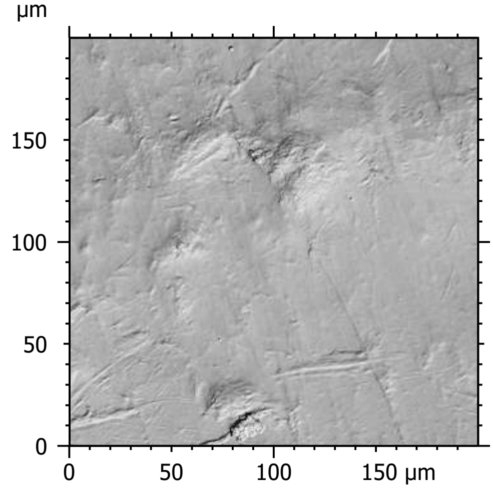

Leica Map Premium 8.2.9843

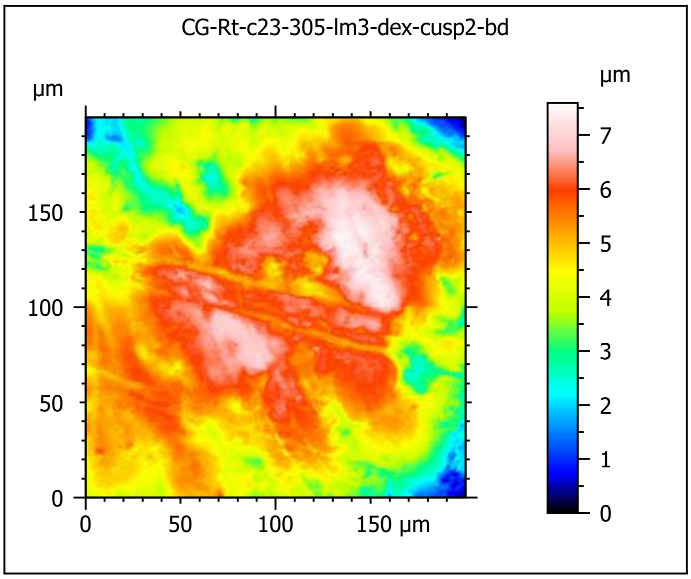

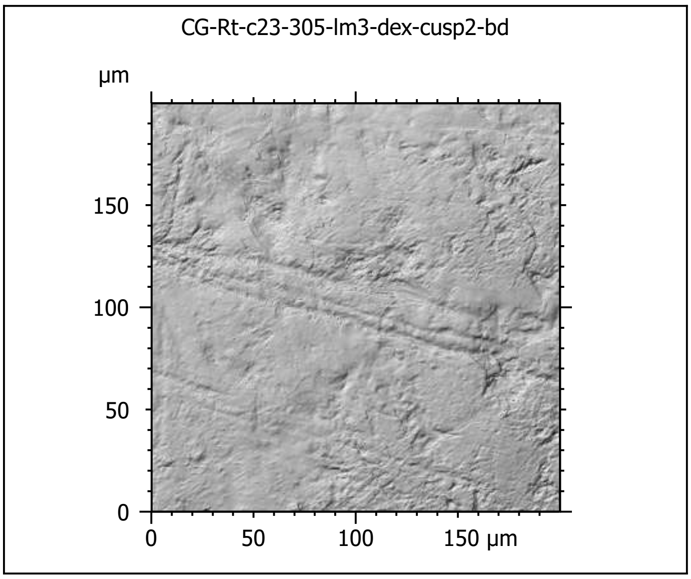

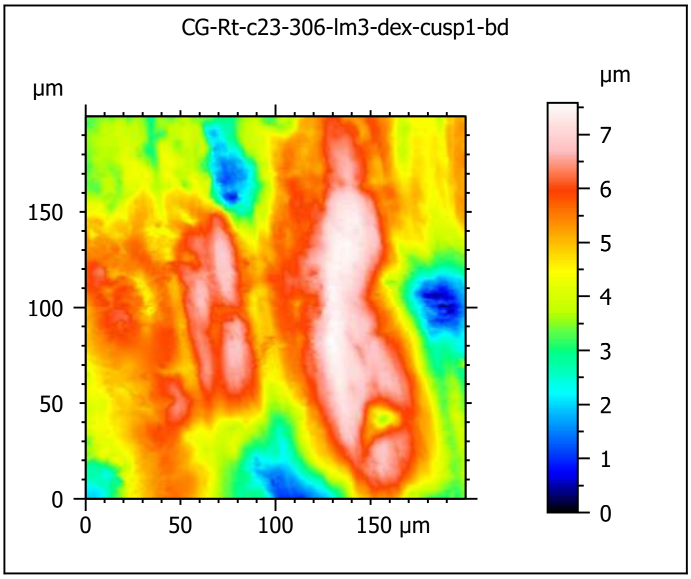

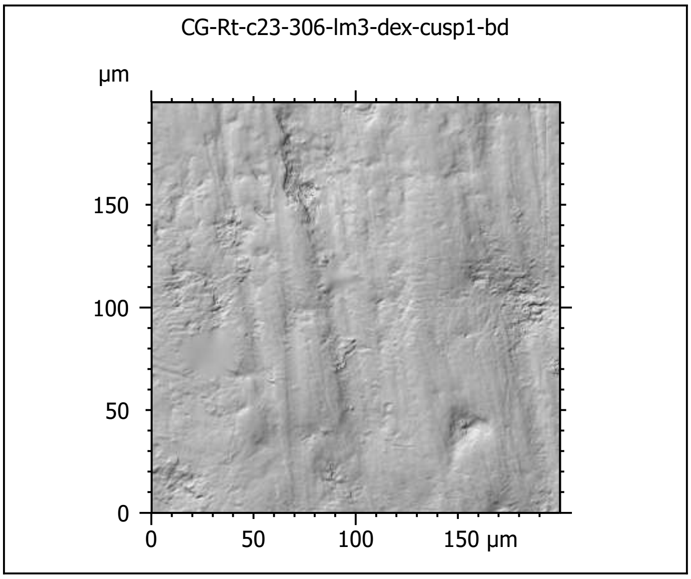

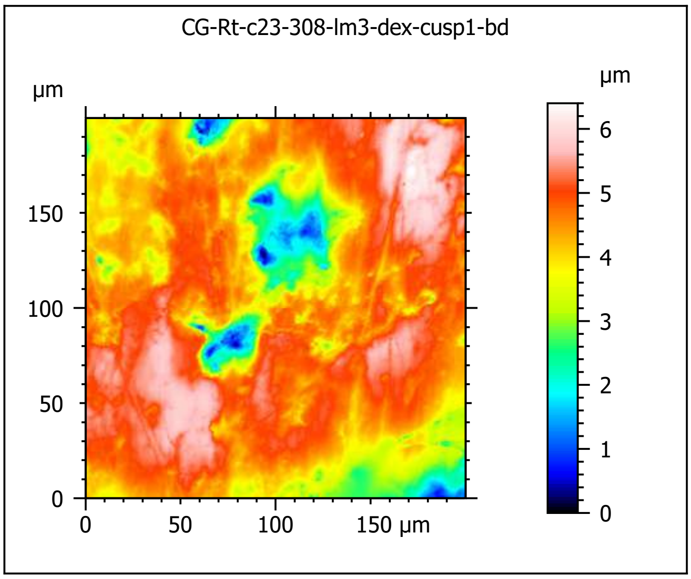

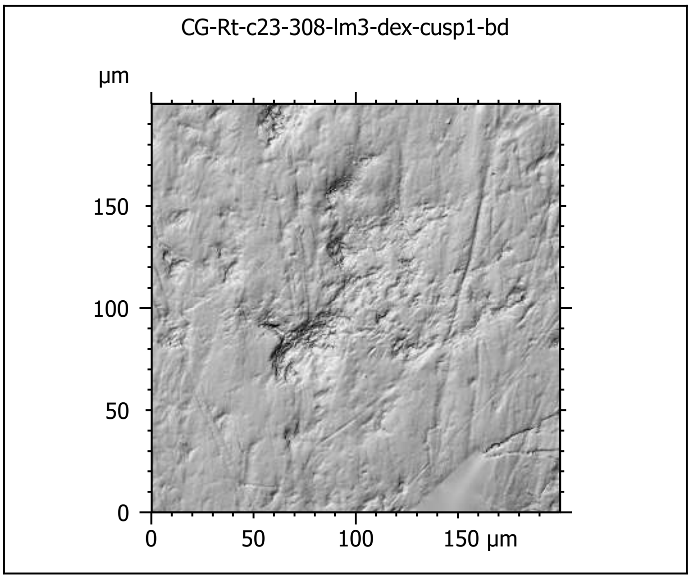

Leica Map Premium 8.2.9843

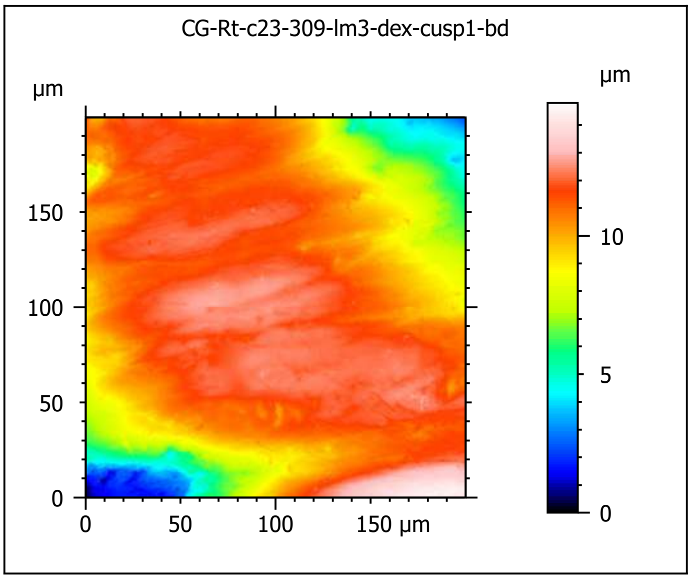

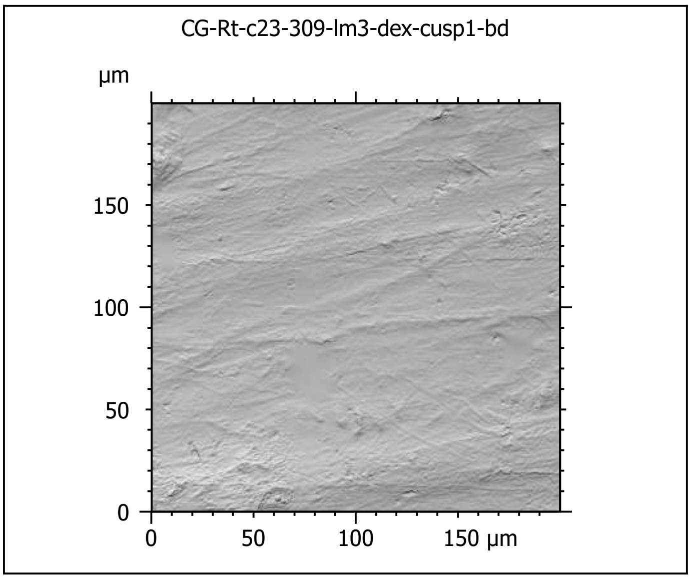

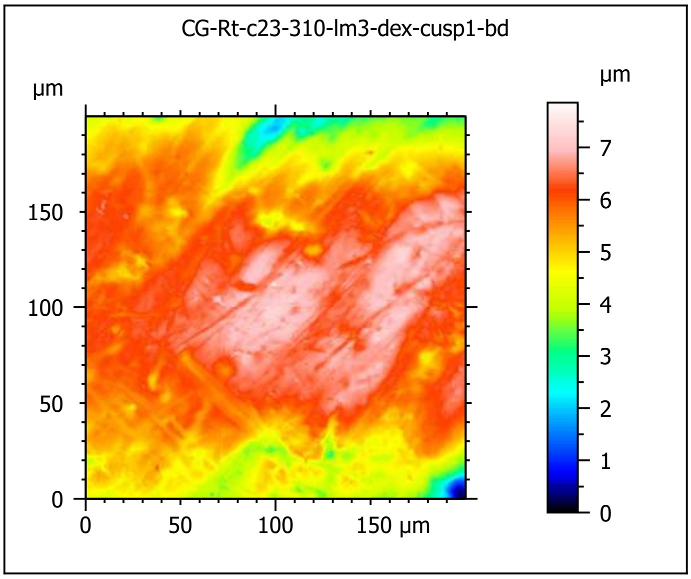

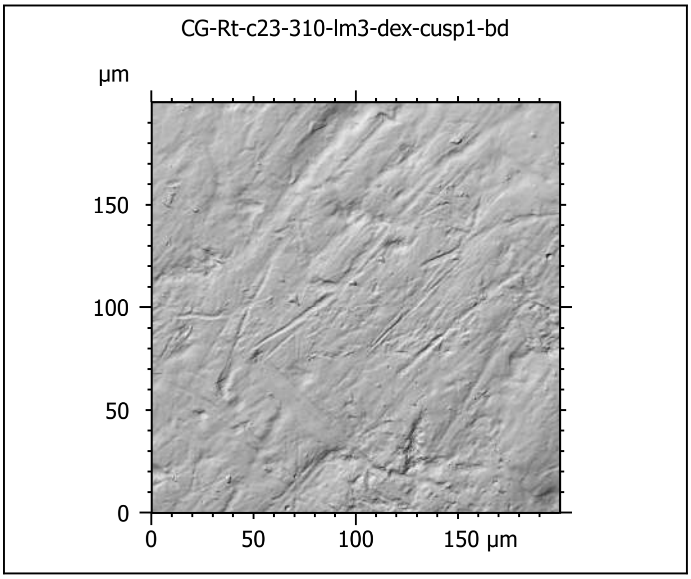

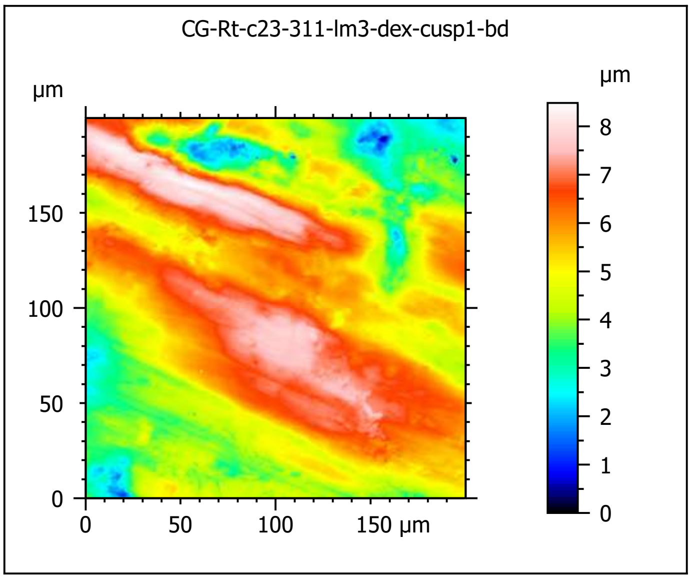

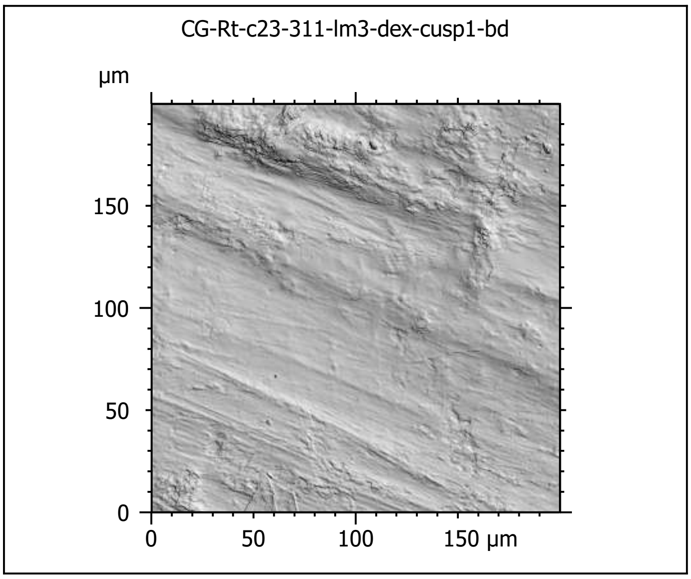

Leica Map Premium 8.2.9843

CG-Rt-c23-312-lm3-dex-cusp1-bd

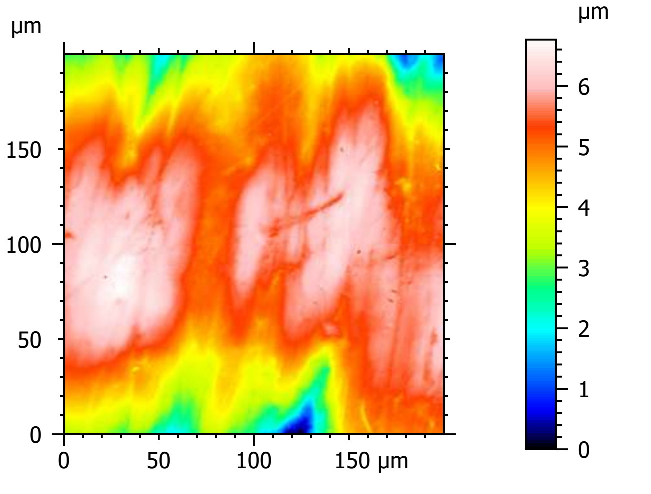

CG-Rt-c23-312-lm3-dex-cusp1-bd

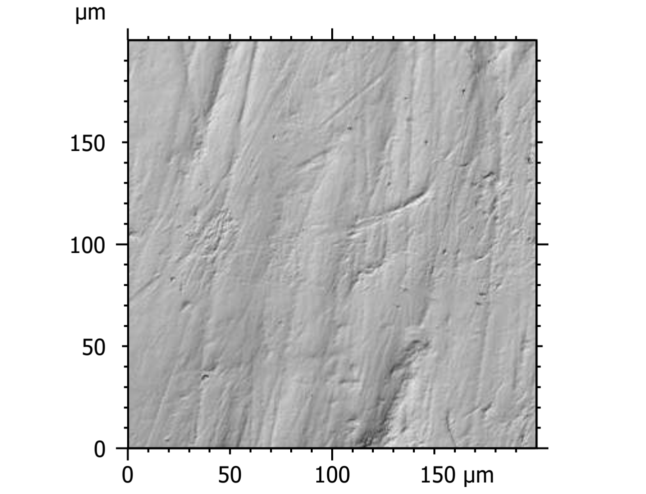

CG-Rt-c23-314-lm2-dex-cusp1-bd

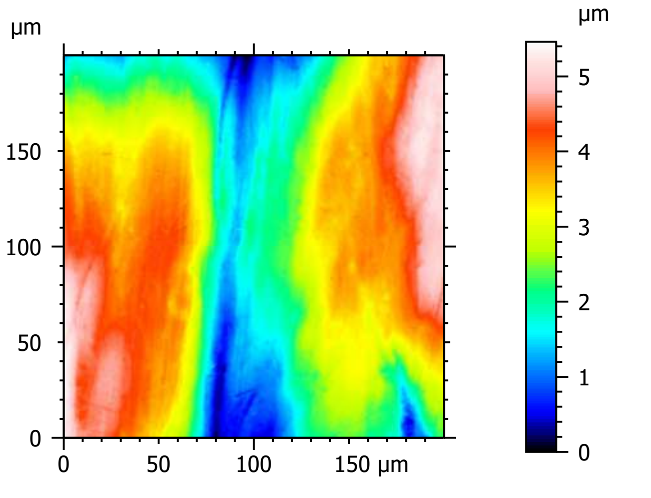

CG-Rt-c23-314-lm2-dex-cusp1-bd

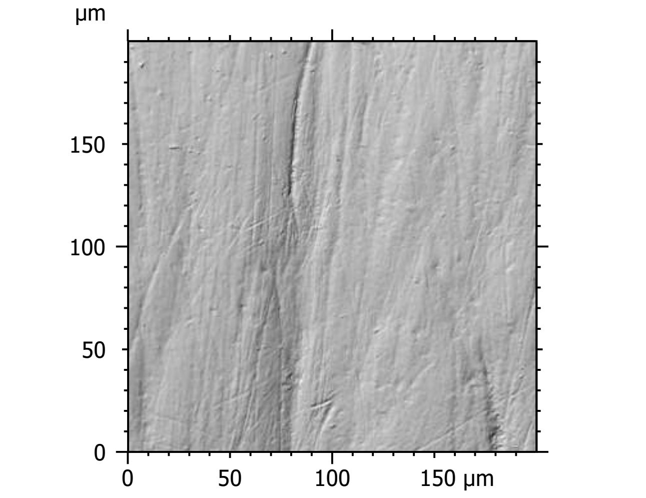

CG-Rt-c23-315-lm3-dex-cusp1-bd

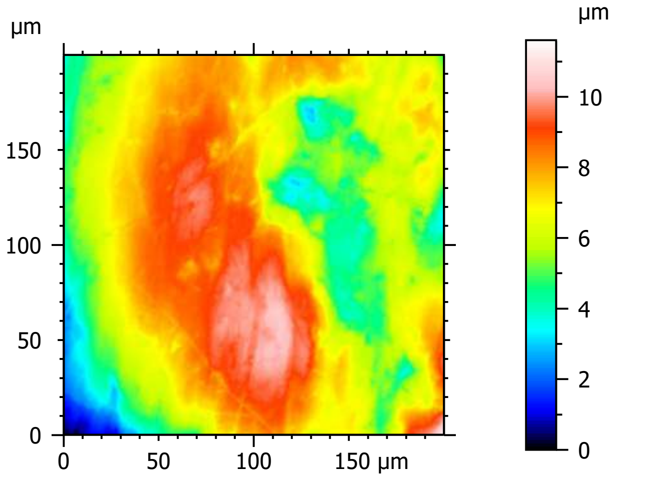

CG-Rt-c23-315-lm3-dex-cusp1-bd

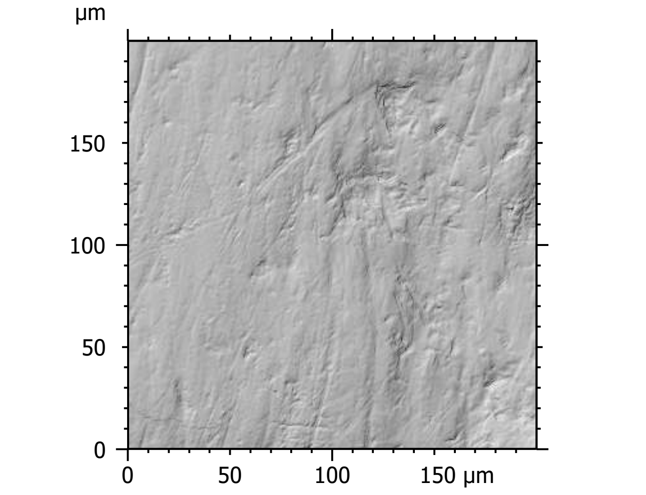

Leica Map Premium 8.2.9843

CG-Rt-c23-316-lm2-dex-cusp1-bd

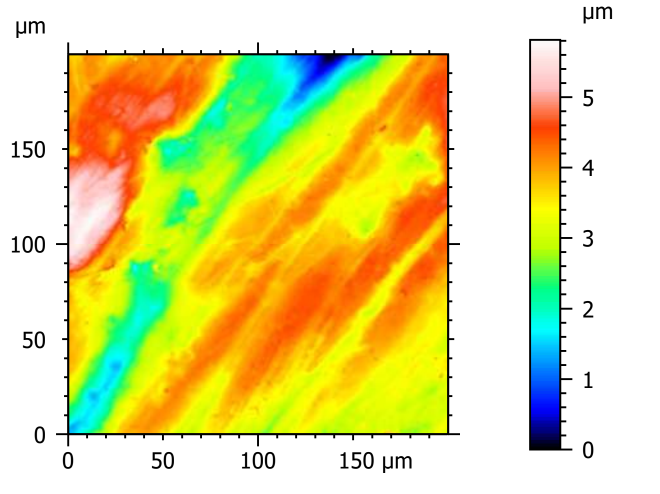

CG-Rt-c23-316-lm2-dex-cusp1-bd

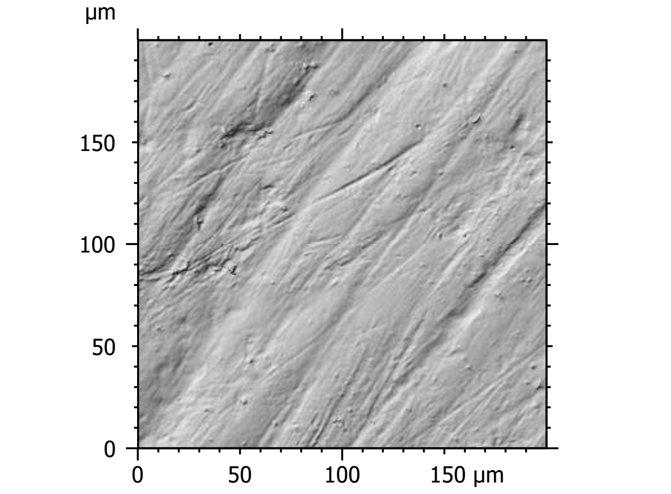

CG-Rt-c23-317-lm3-dex-cusp1-bd

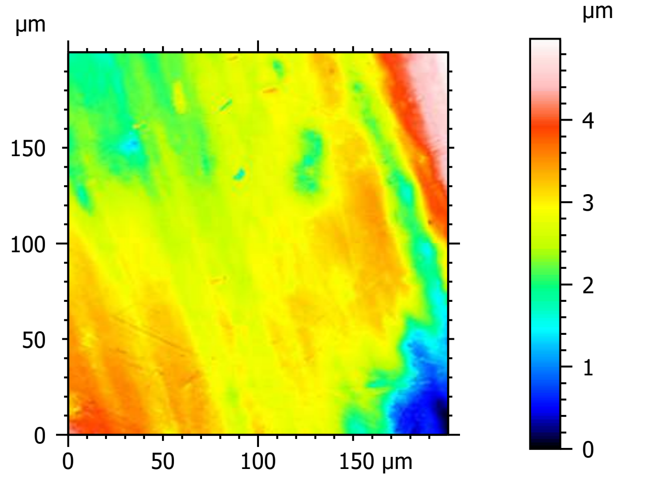

CG-Rt-c23-317-lm3-dex-cusp1-bd

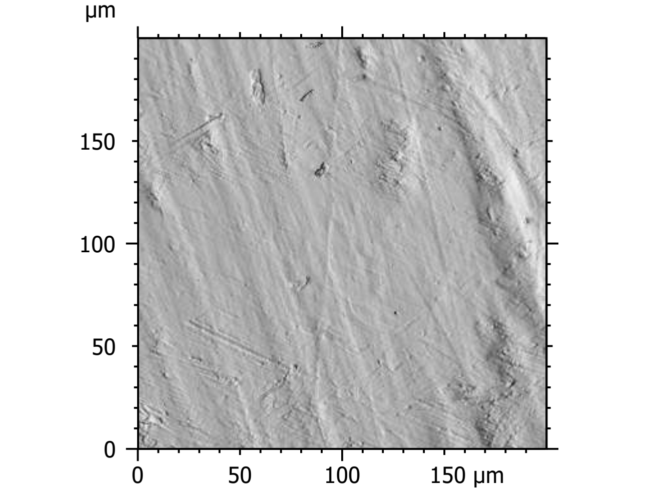

CG-Rt-c23-318-lm2-dex-cusp1-bd

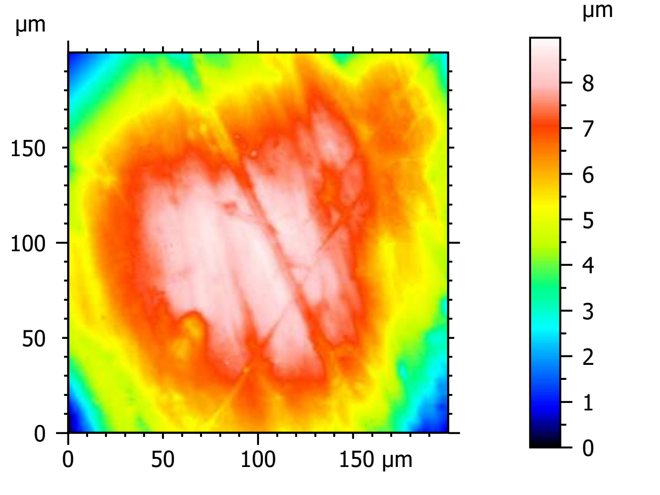

CG-Rt-c23-318-lm2-dex-cusp1-bd

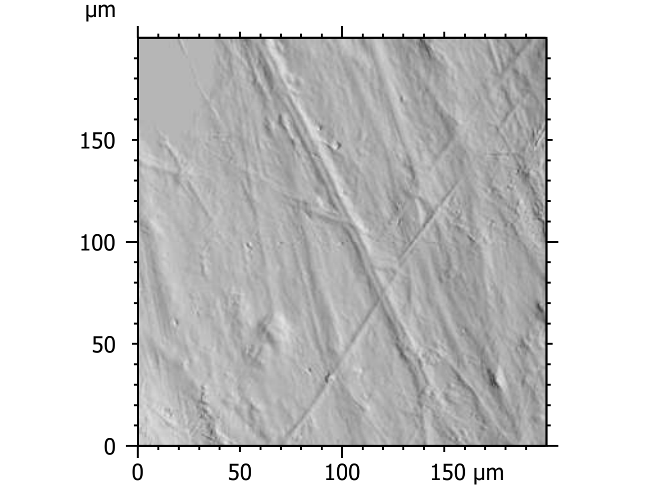

Leica Map Premium 8.2.9843

CG-Rt-c23-319-lm2-dex-cusp1-bd

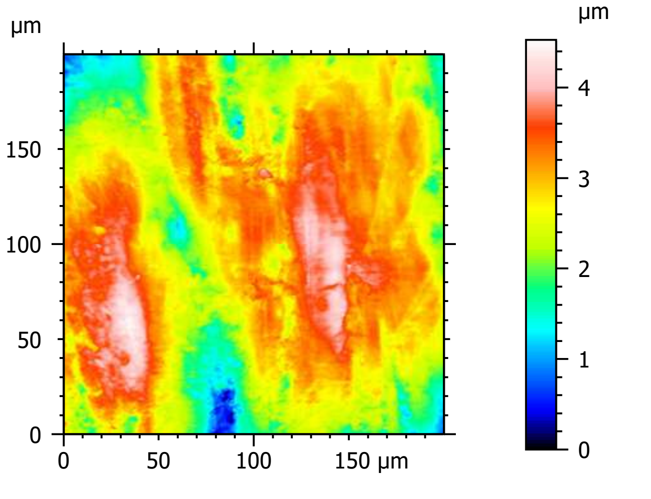

CG-Rt-c23-319-lm2-dex-cusp1-bd

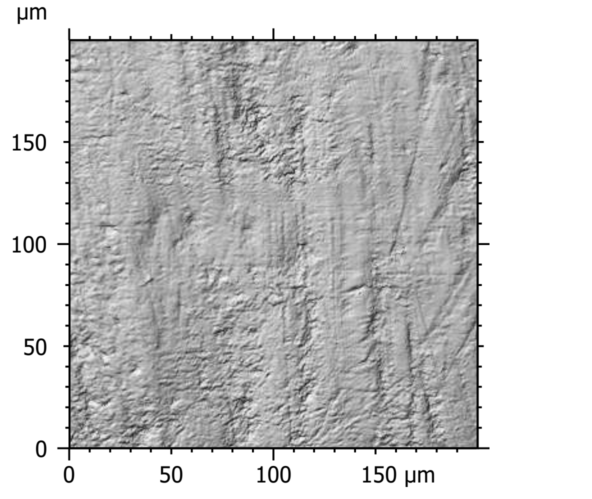

CG-Rt-c23-1023-lm2-sin-cusp1-bd

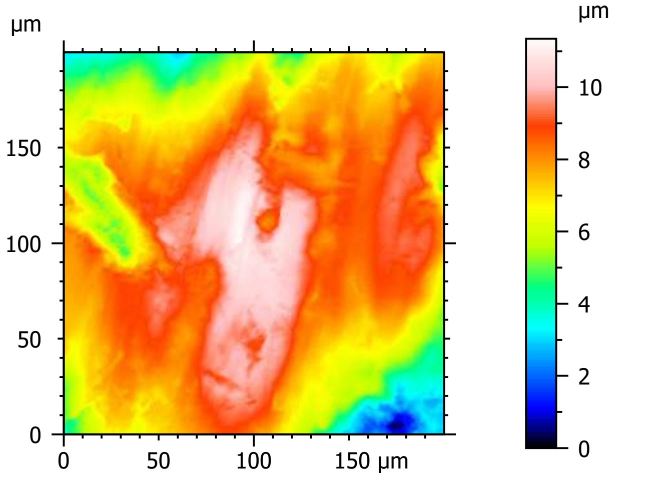

CG-Rt-c23-1023-lm2-sin-cusp1-bd

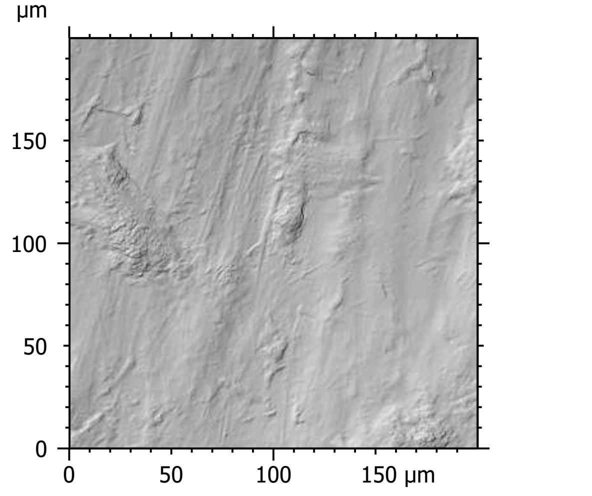

CG-Rt-c23-1024-lm2-sin-cusp1-bd

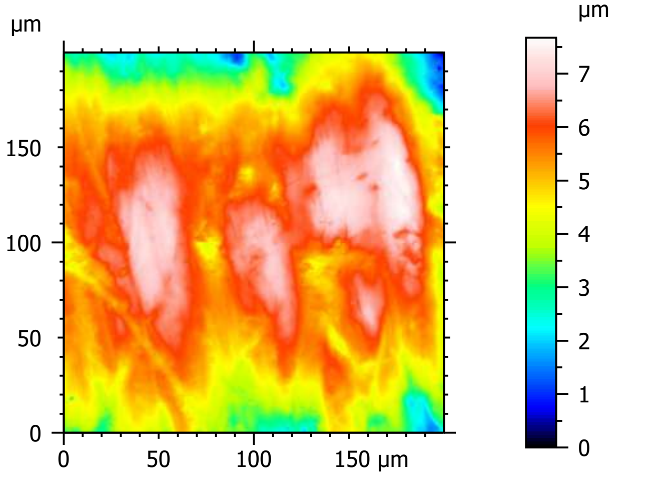

CG-Rt-c23-1024-lm2-sin-cusp1-bd

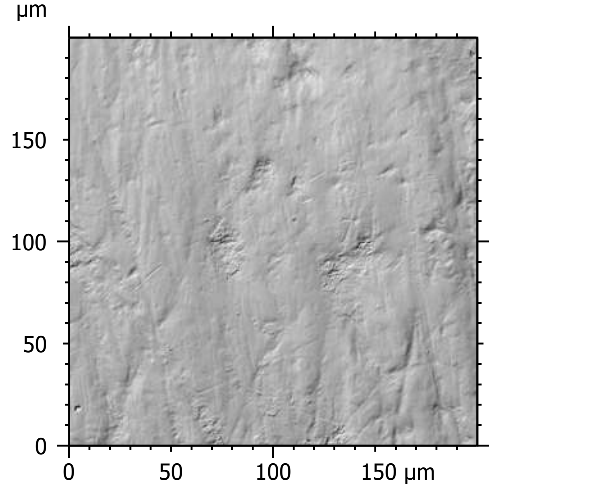

Leica Map Premium 8.2.9843

CG-Rt-c24-463-lm2-sin-cusp1-bd

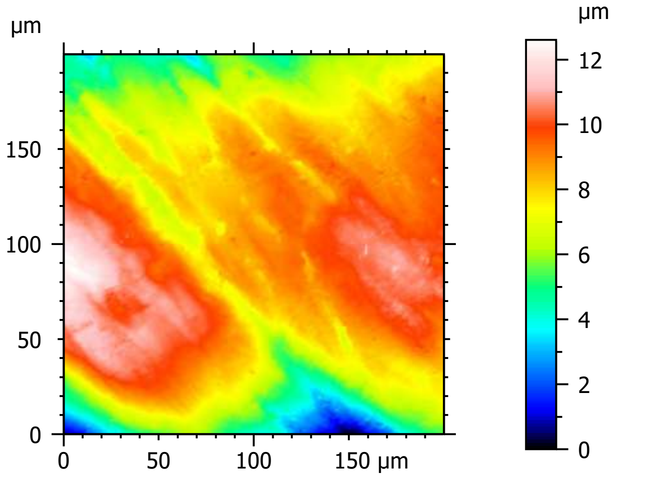

CG-Rt-c24-463-lm2-sin-cusp1-bd

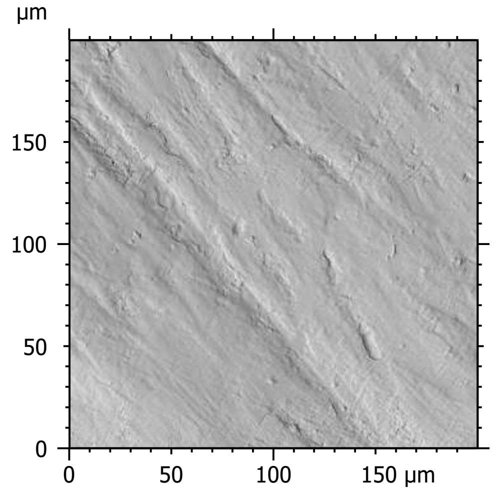

CG-Rt-c24-465-lm1-sin-cusp1-bd

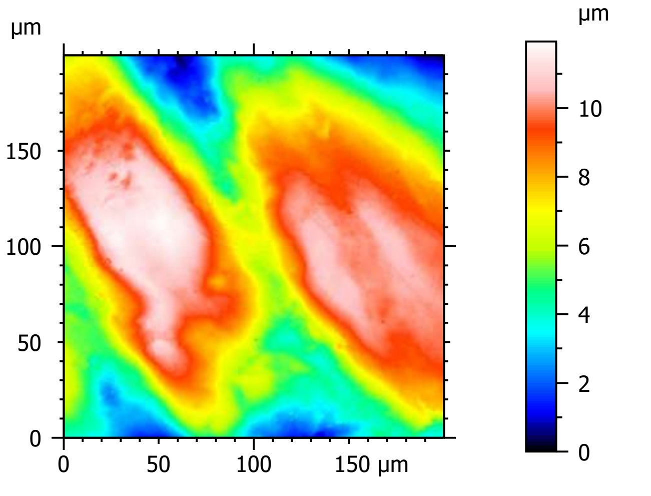

CG-Rt-c24-465-lm1-sin-cusp1-bd

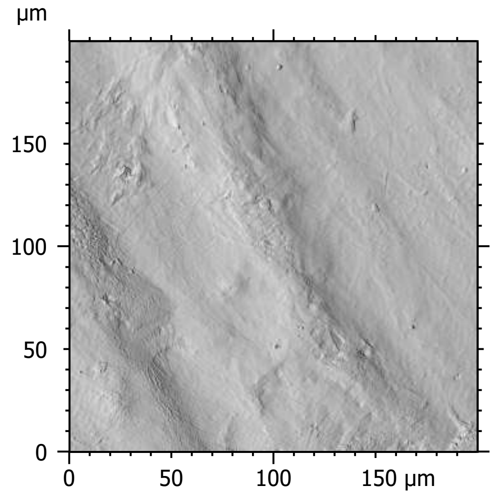

CG-Rt-c24-467-lm2-sin-cusp1-bd

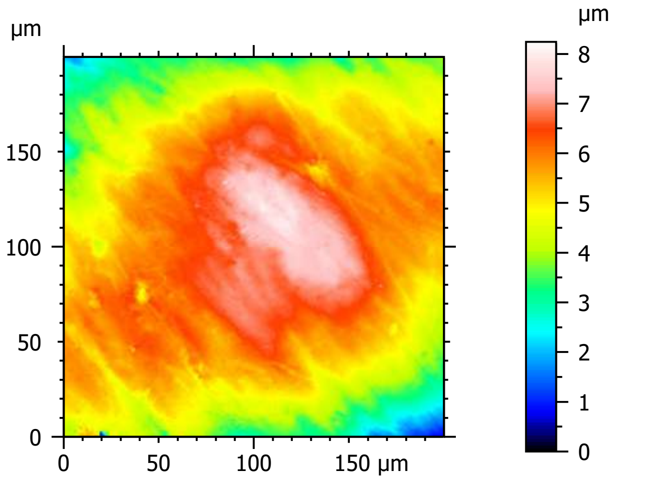

CG-Rt-c24-467-lm2-sin-cusp1-bd

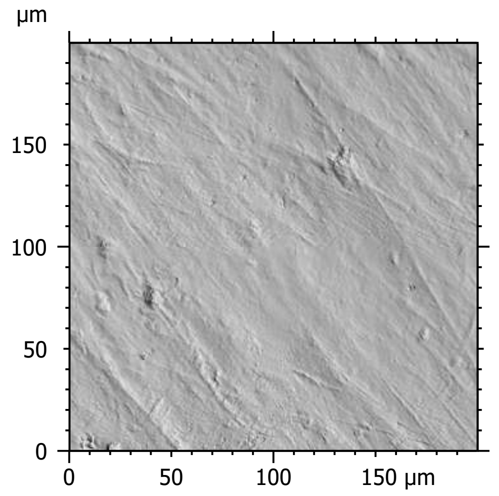

Leica Map Premium 8.2.9843

CG-Rt-c24-468-lm3-sin-cusp2-bd

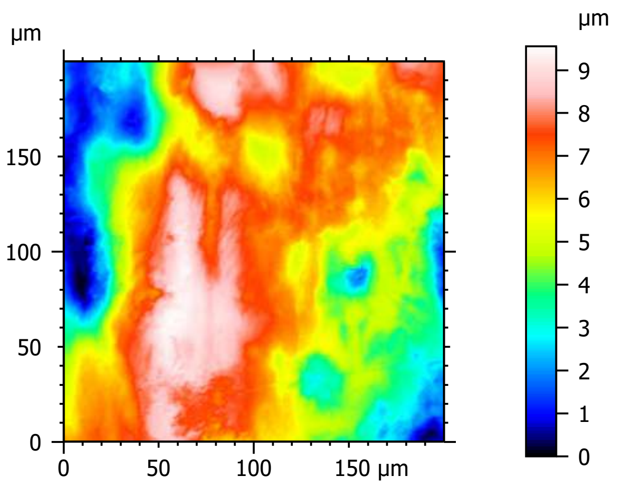

CG-Rt-c24-468-lm3-sin-cusp2-bd

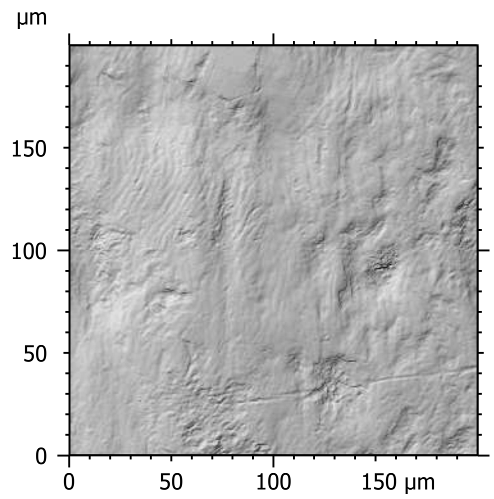

CG-Rt-c24-469-lm3-sin-cusp1-bd

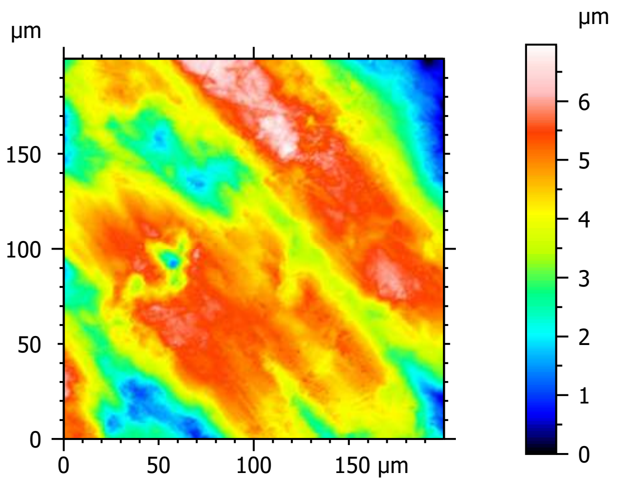

CG-Rt-c24-469-lm3-sin-cusp1-bd

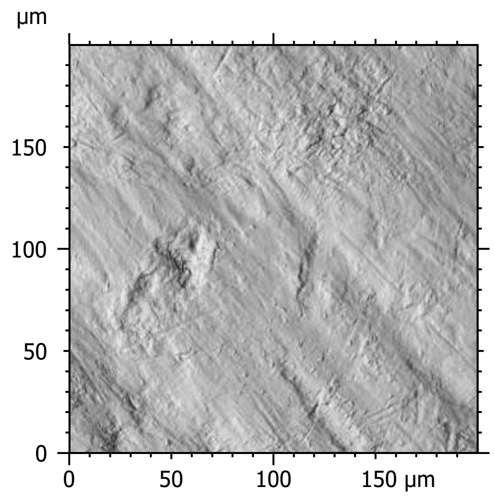

Leica Map Premium 8.2.9843

CG-Rt-c25-444-lm1-sin-cusp1-bd

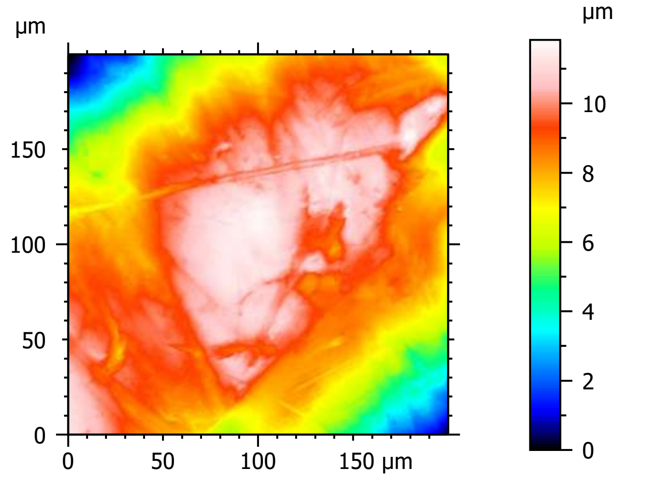

CG-Rt-c25-444-lm1-sin-cusp1-bd

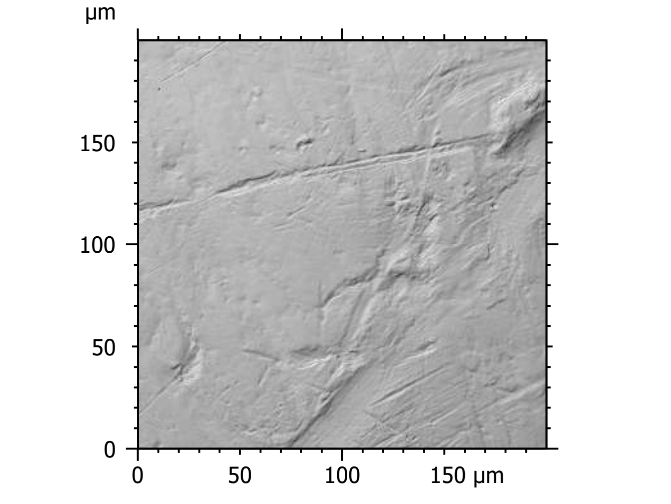

CG-Rt-c25-446-lm2-sin-cusp1-bd

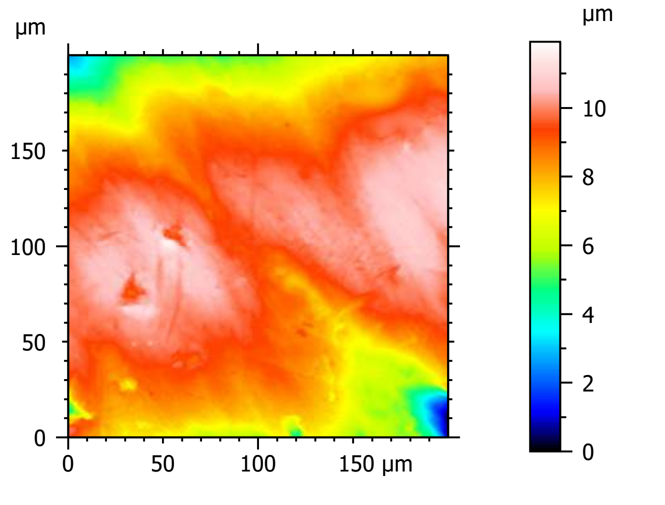

CG-Rt-c25-446-lm2-sin-cusp1-bd

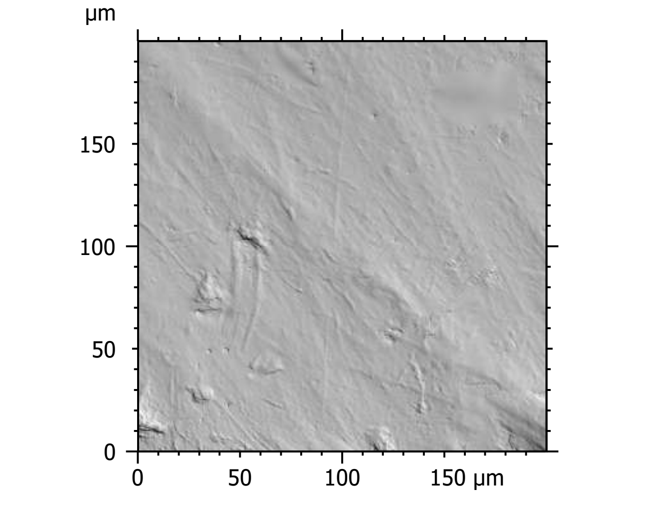

CG-Rt-c25-447-lm3-dex-cusp1-bd

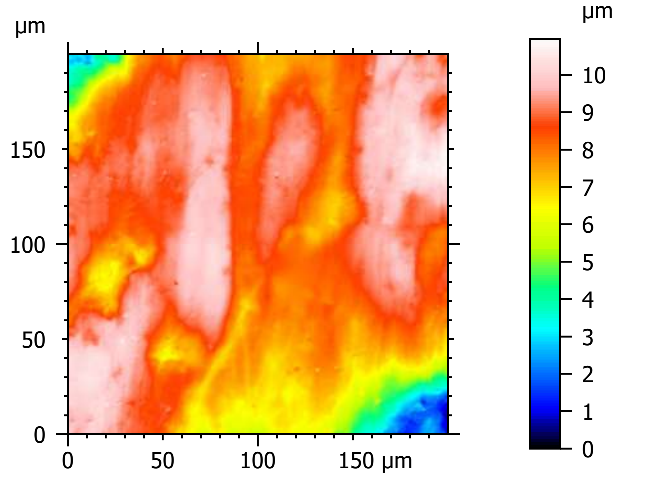

CG-Rt-c25-447-lm3-dex-cusp1-bd

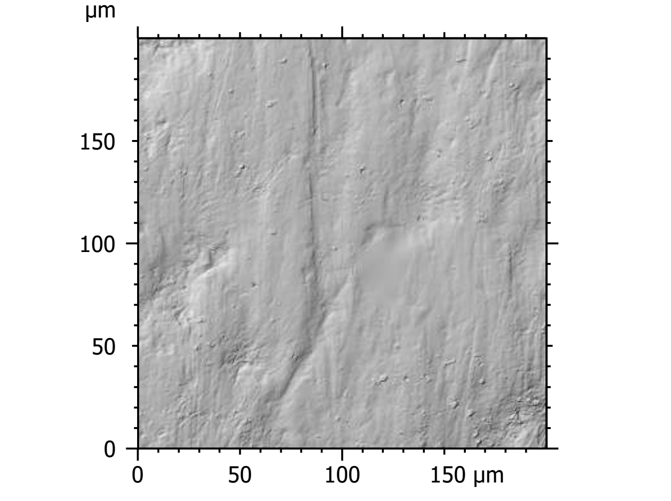

CG-Rt-c25-449-lm3-sin-cusp1-bd

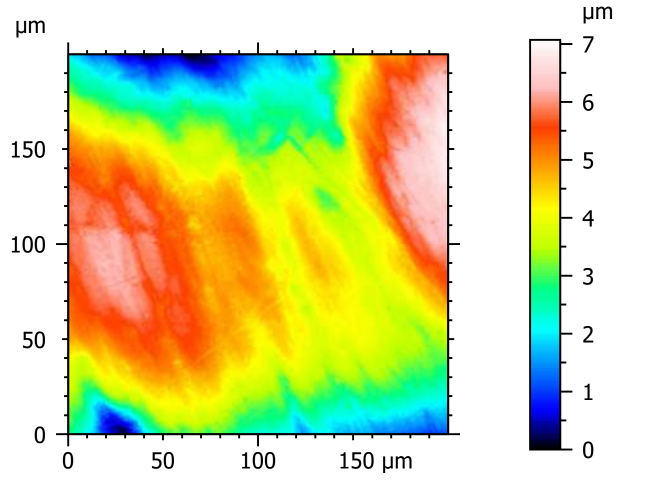

CG-Rt-c25-449-lm3-sin-cusp1-bd

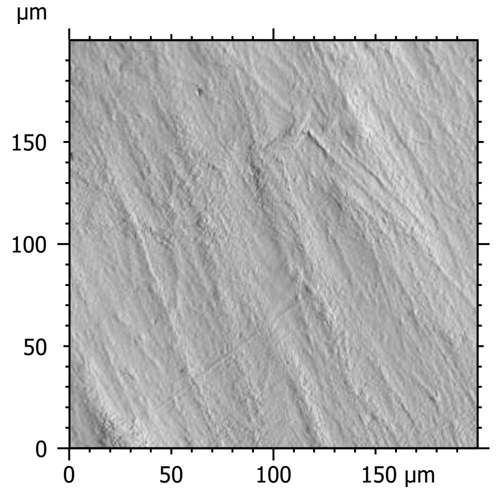

CG-Rt-c25-451-lm3-sin-cusp1-bd

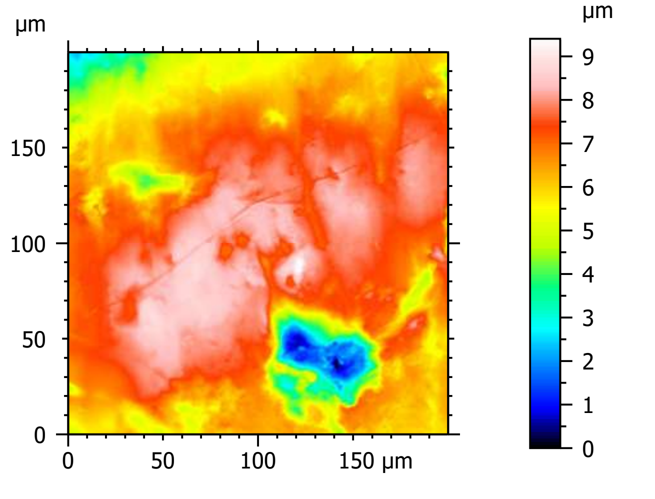

CG-Rt-c25-451-lm3-sin-cusp1-bd

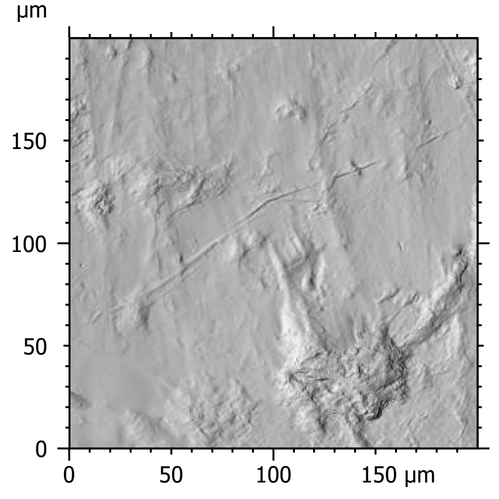

CG-Rt-c25-452-lm3-sin-cusp1-bd

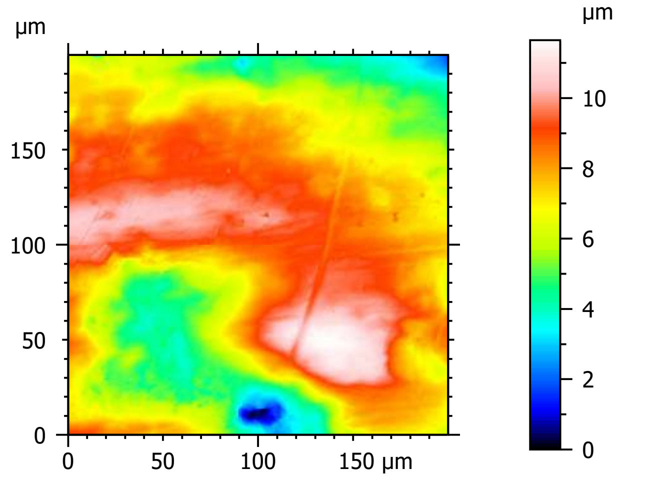

CG-Rt-c25-452-lm3-sin-cusp1-bd

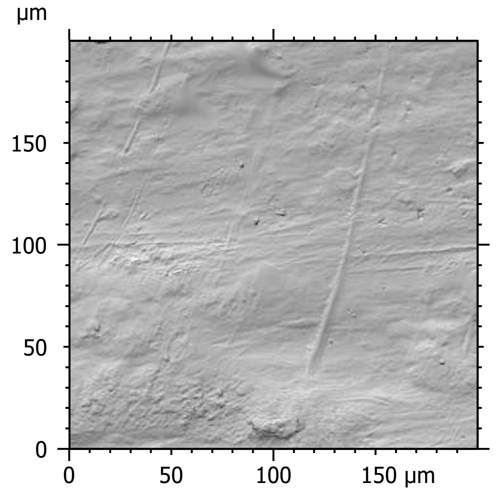

Leica Map Premium 8.2.9843

CG-Rt-c26-453-lm3-sin-cusp1-bd

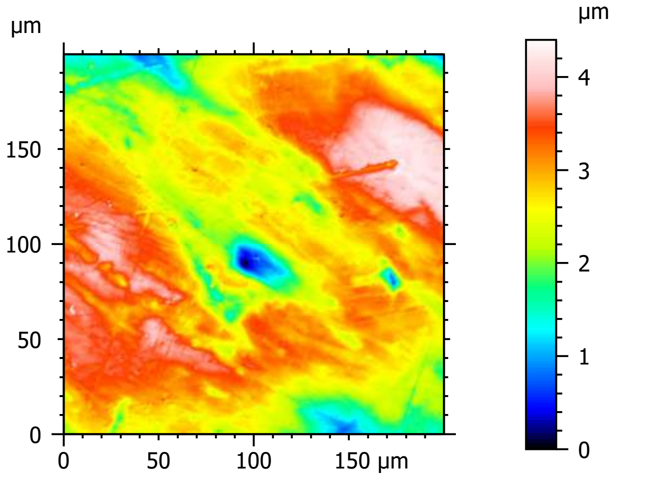

CG-Rt-c26-453-lm3-sin-cusp1-bd

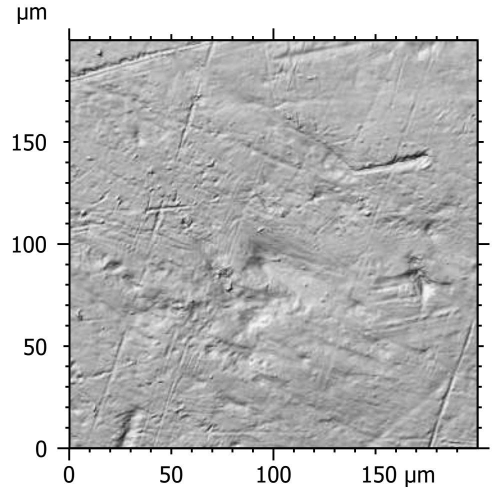

CG-Rt-c26-455-lm3-sin-cusp1-bd

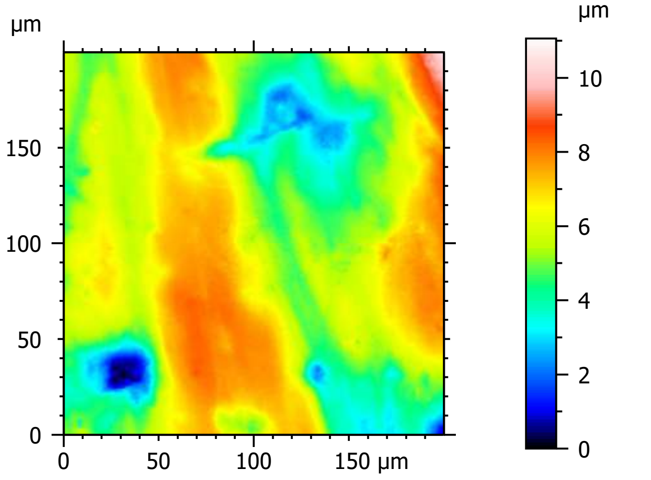

CG-Rt-c26-455-lm3-sin-cusp1-bd

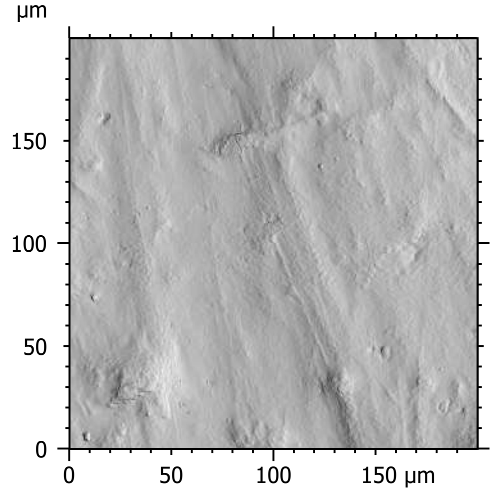

CG-Rt-c26-454-lm-sin-cusp1-bd

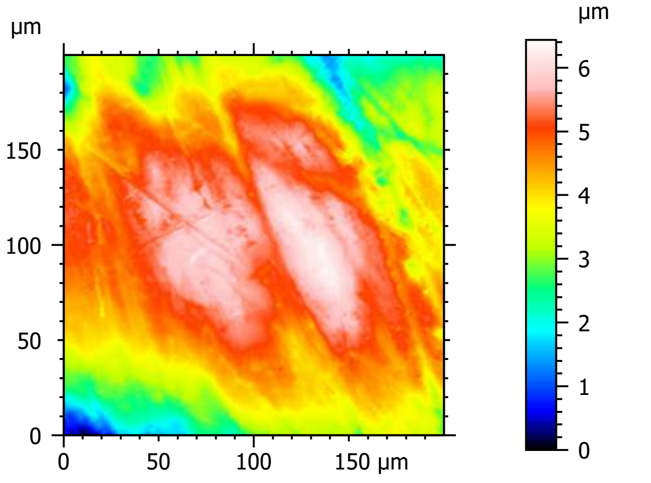

CG-Rt-c26-454-lm-sin-cusp1-bd

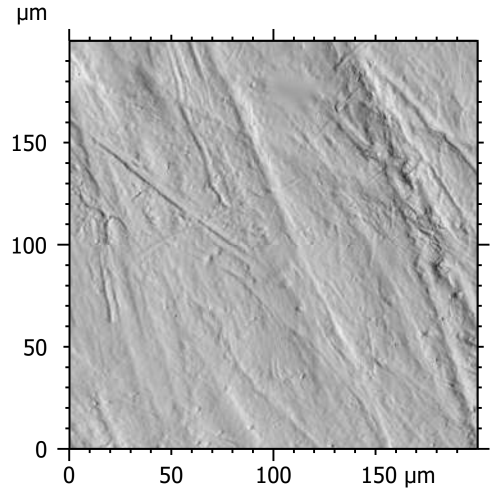

Leica Map Premium 8.2.9843

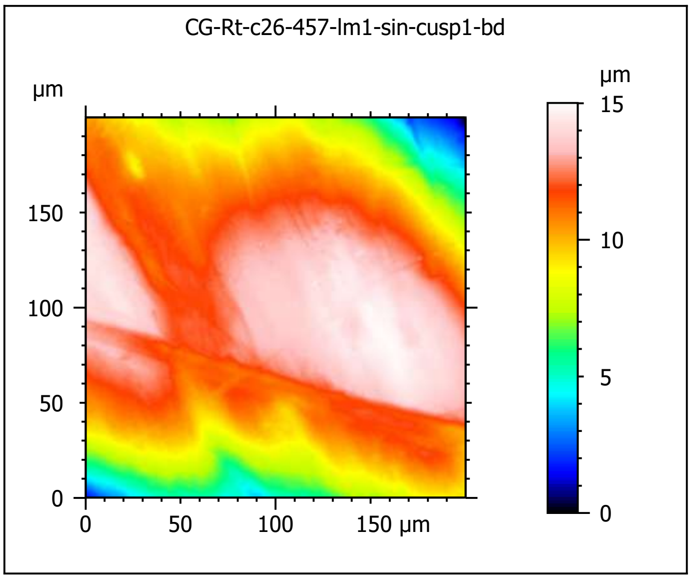
CG-Rt-c26-457-lm1-sin-cusp1-bd
µm
150
100
50
0
0
50
100
150 µm
µm
15
10
5
0

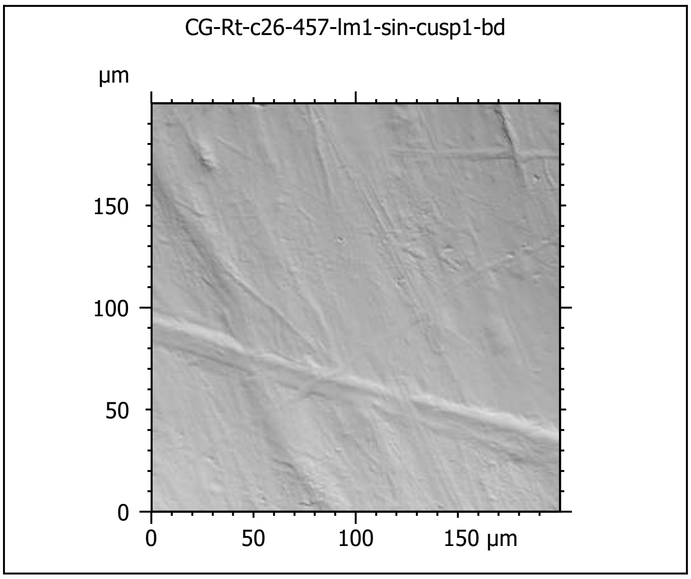
CG-Rt-c26-457-lm1-sin-cusp1-bd
µm
150
100
50
0
0
50
100
150 µm

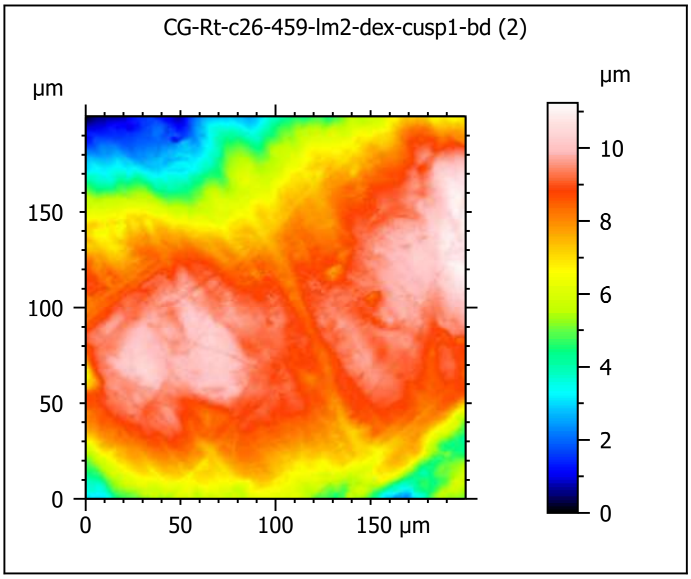
CG-Rt-c26-459-lm2-dex-cusp1-bd (2)
µm
150
100
50
0
0
50
100
150 µm
µm
10
8
6
4
2
0

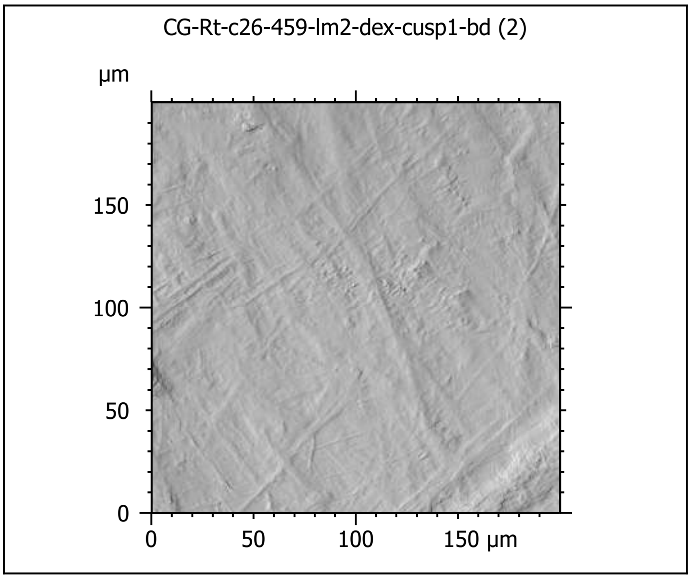
CG-Rt-c26-459-lm2-dex-cusp1-bd (2)
µm
150
100
50
0
0
50
100
150 µm

"A long-term perspective on Neandertal environment and subsistence: insights from the dental micro-texture analysis of hunted ungulates at Combe-Grenal (Dordogne, France)"

authors: Berlioz, E.; Capdepon, E.; Discamps, E.

Appendice 2:
surfaces scanned by E. Berlioz and E. Capdepon, pre-treatement by E. Berlioz and E. Capdepon, validation by E. Berlioz (2019)

***Rangifer tarandus* - Block G**

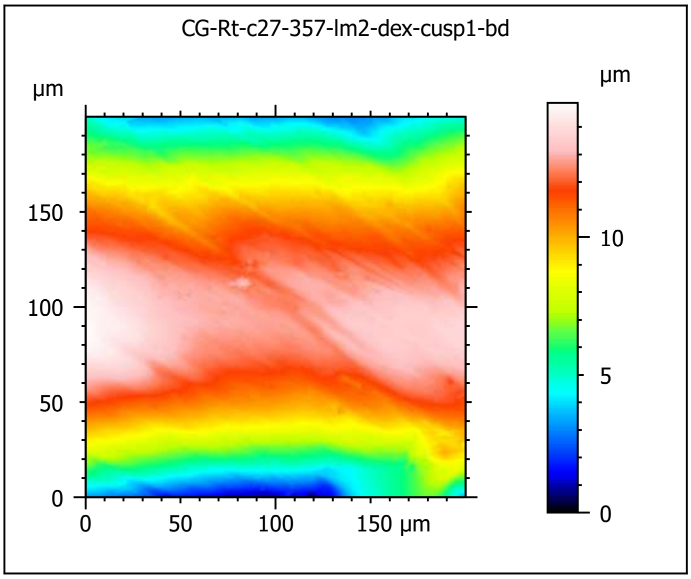

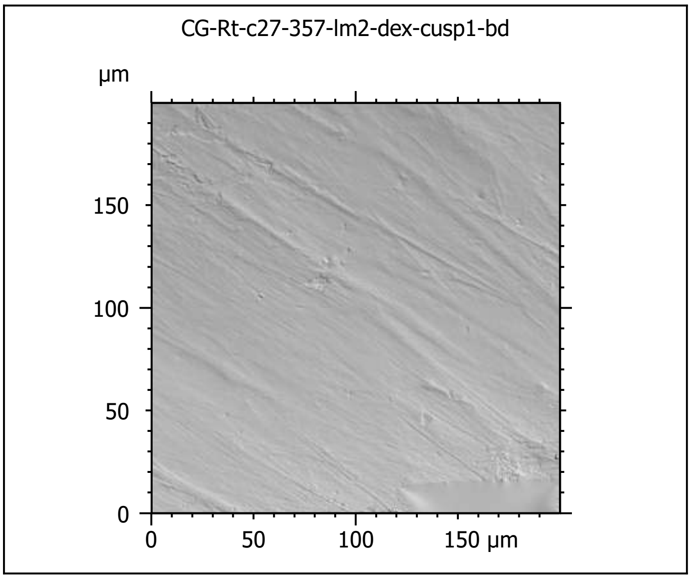

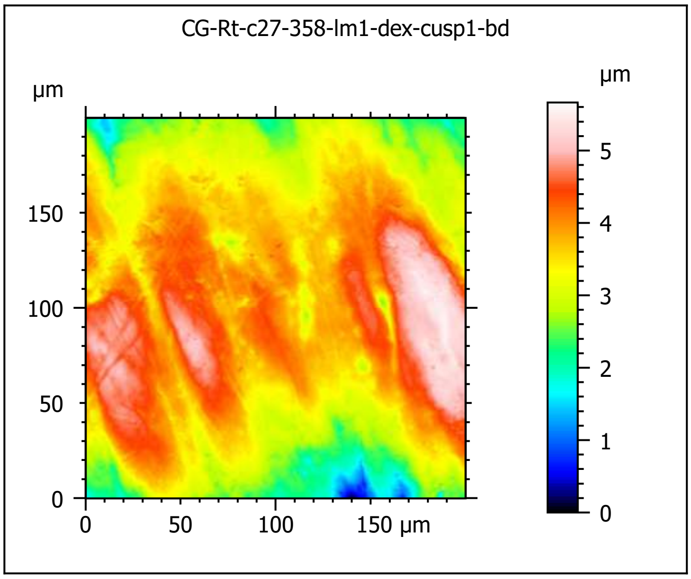

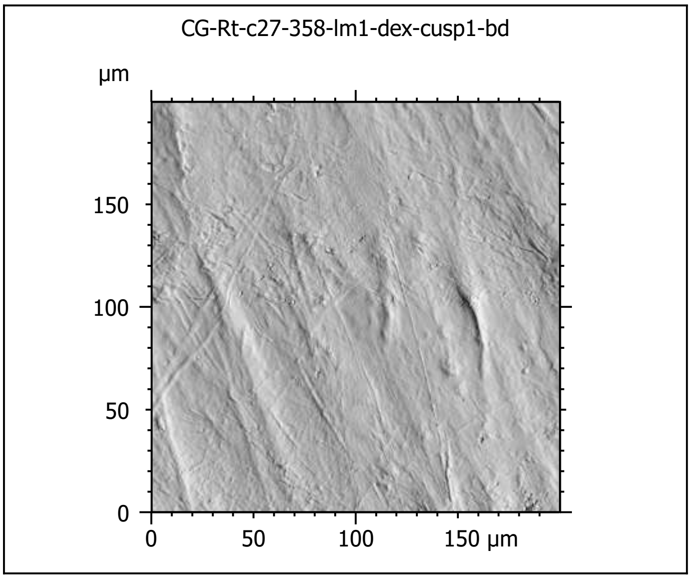

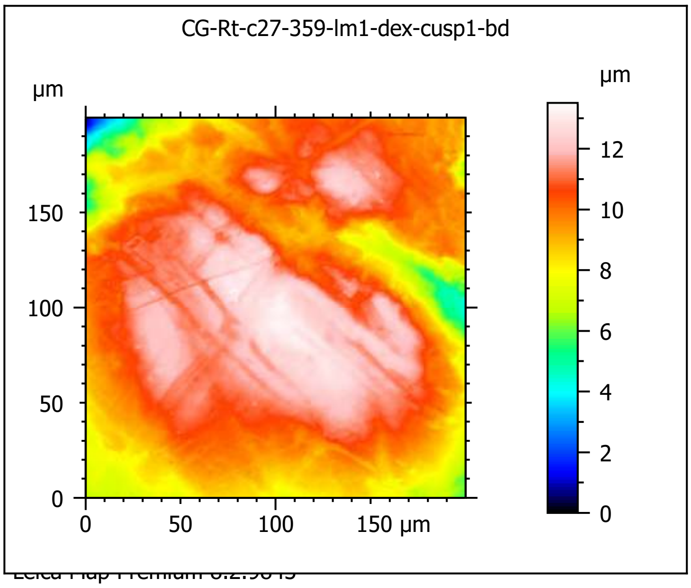

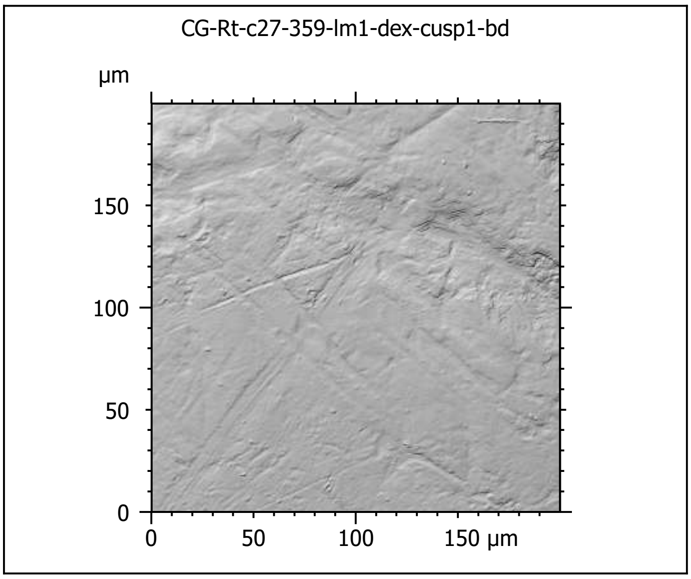

CG-Rt-c27-360-lm3-dex-cusp1-bd

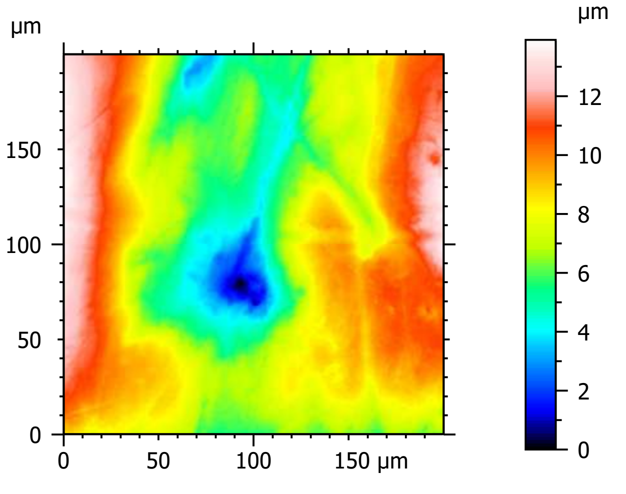

CG-Rt-c27-360-lm3-dex-cusp1-bd

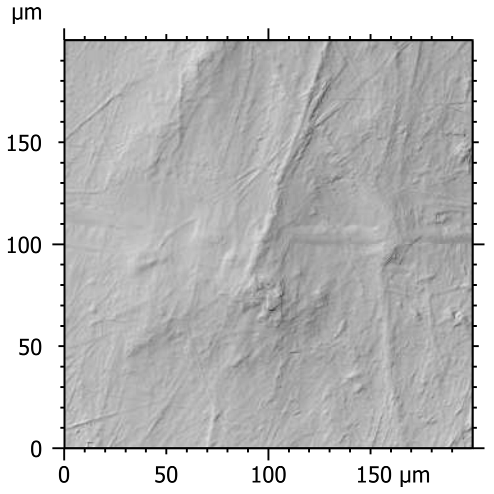

CG-Rt-c27-361-lm2-dex-cusp1-bd

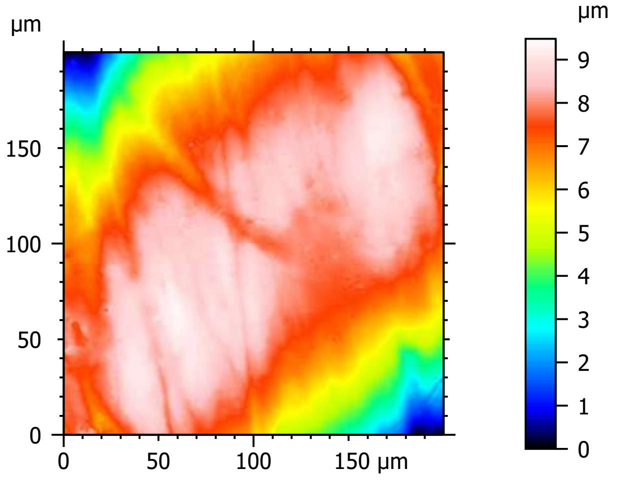

CG-Rt-c27-361-lm2-dex-cusp1-bd

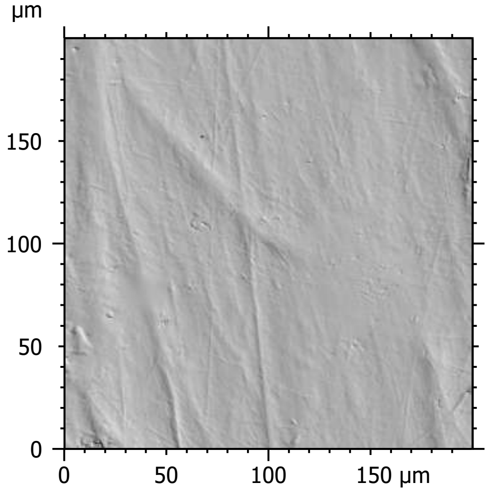

CG-Rt-c27-363-lm-dex-cusp2-bd

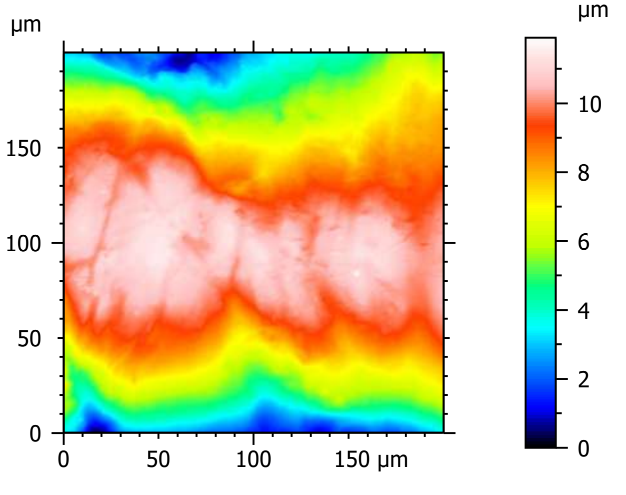

CG-Rt-c27-363-lm-dex-cusp2-bd

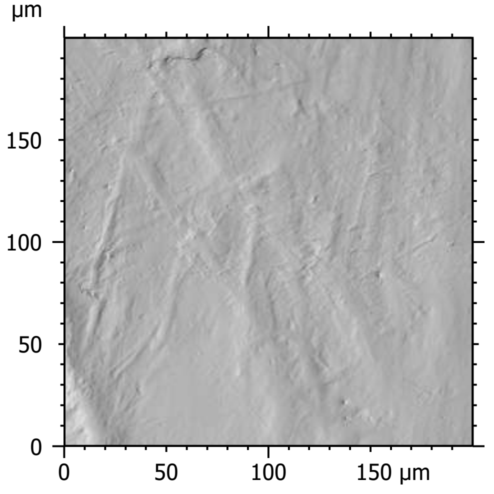

Leica Map Premium 8.2.9843

CG-Rt-c27-364-lm3-dex-cusp2-bd

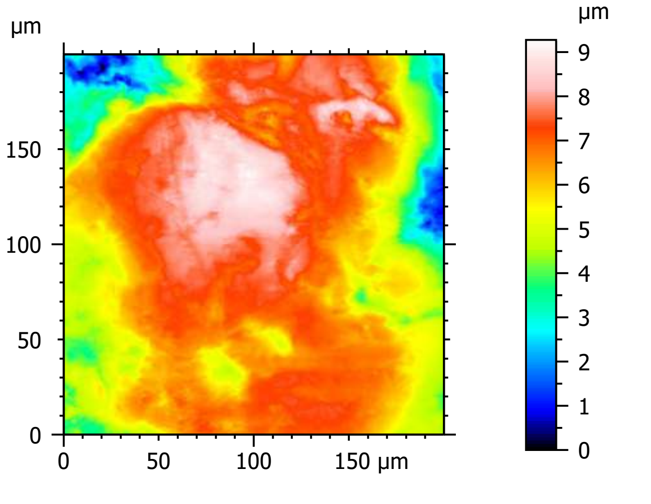

CG-Rt-c27-364-lm3-dex-cusp2-bd

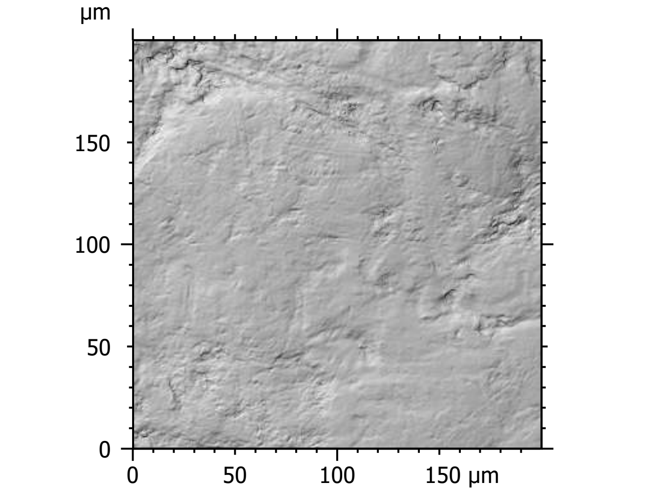

CG-Rt-c27-365-lm2-dex-cusp1-bd

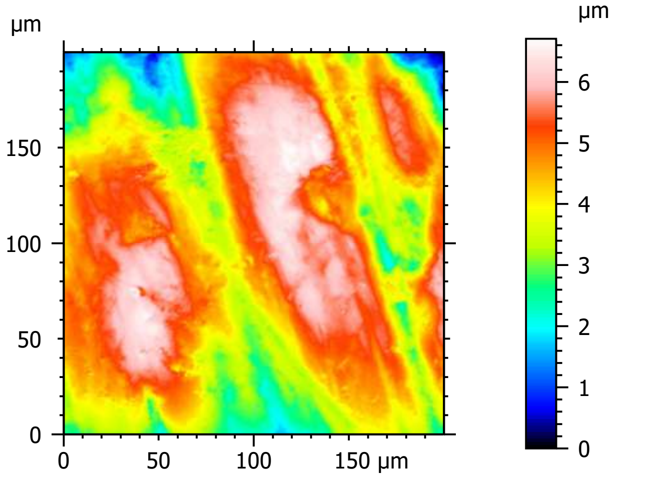

CG-Rt-c27-365-lm2-dex-cusp1-bd

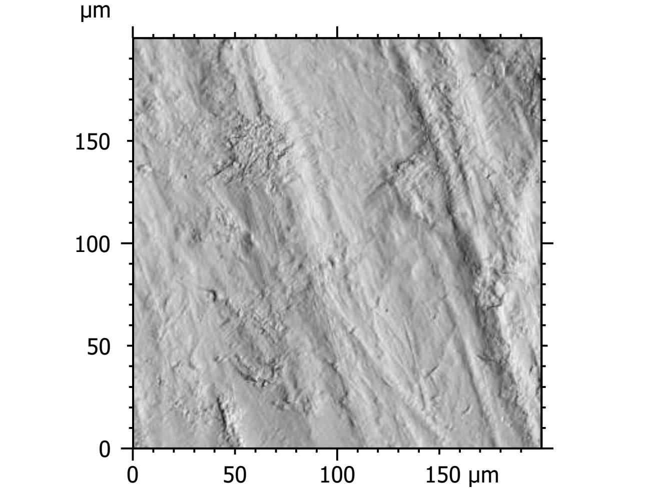

CG-Rt-c27-367-lm3-dex-cusp2-bd

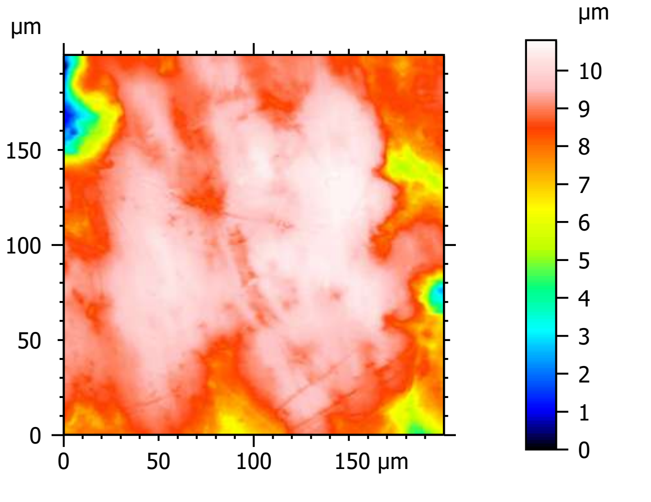

CG-Rt-c27-367-lm3-dex-cusp2-bd

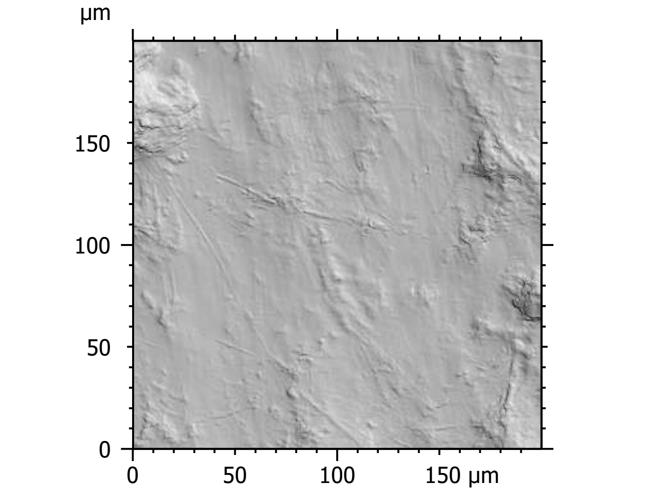

Leica Map Premium 8.2.9843

CG-Rt-c27-369-lm-dex-cusp2-bd

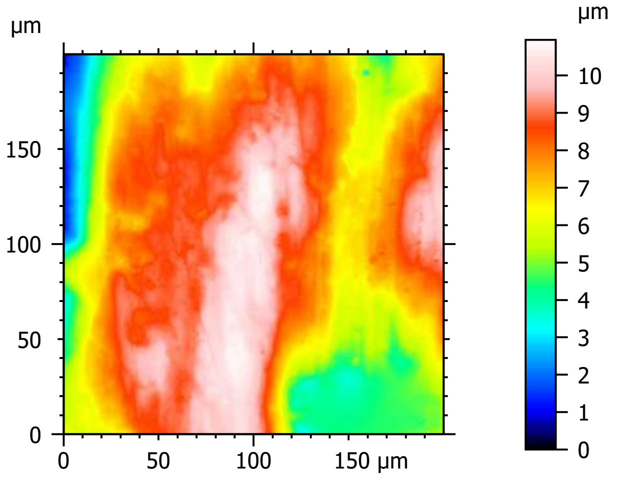

CG-Rt-c27-369-lm-dex-cusp2-bd

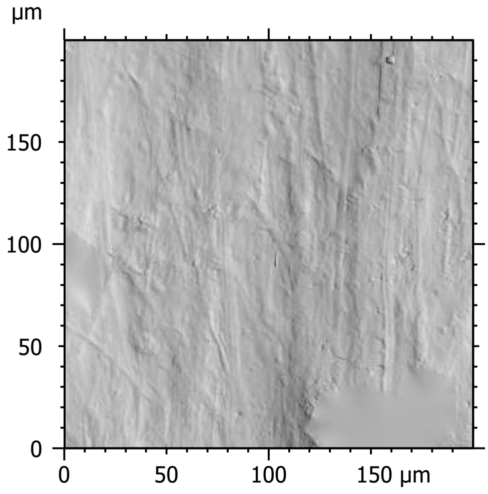

CG-Rt-c27-370-lm-dex-cusp1-bd

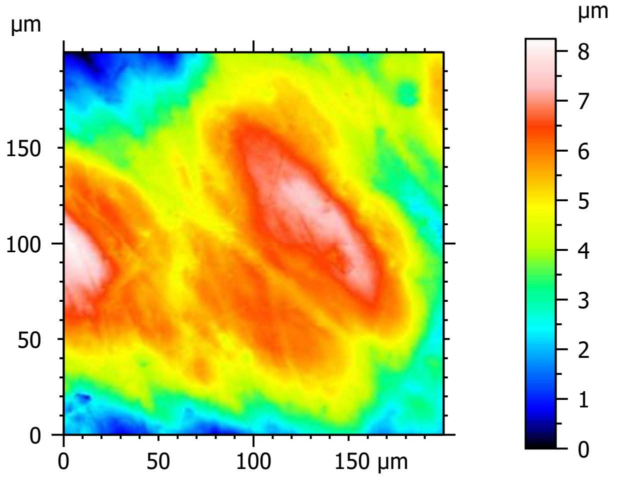

CG-Rt-c27-370-lm-dex-cusp1-bd

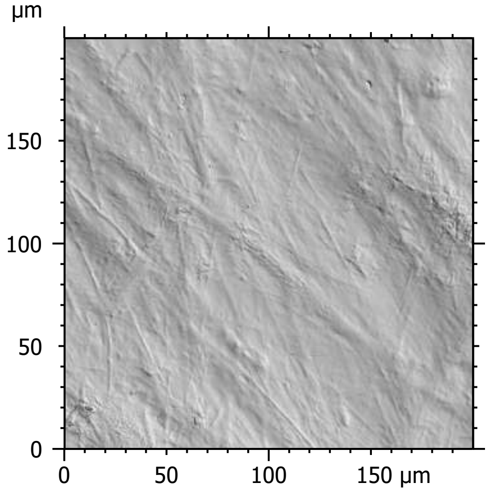

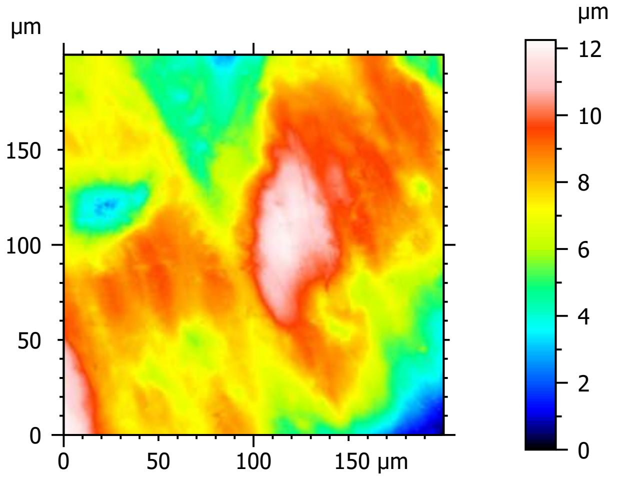

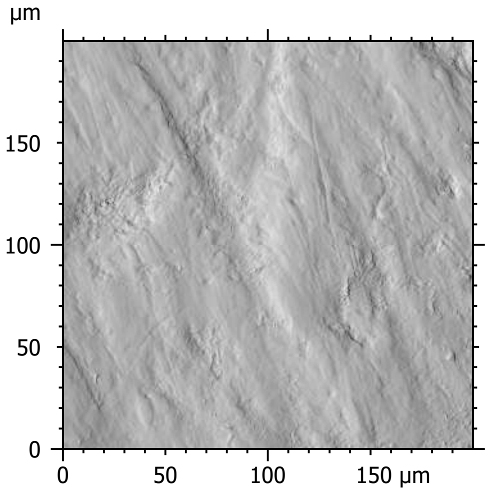

Leica Map Premium 8.2.9843

CG-Rt-c27-372-lm-dex-cusp1-bd

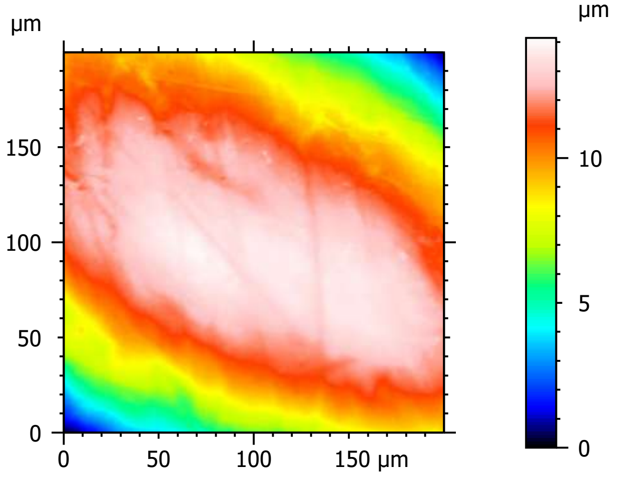

CG-Rt-c27-372-lm-dex-cusp1-bd

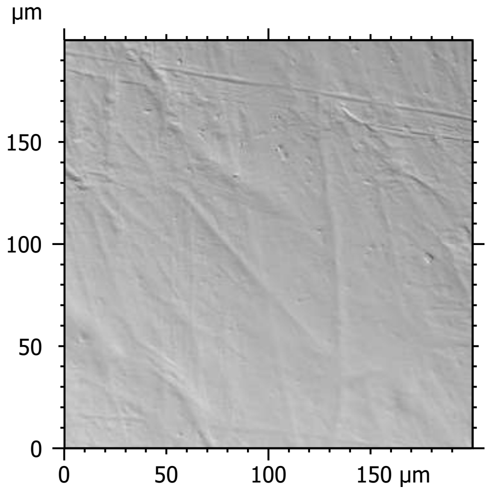

CG-Rt-c27-1018-lm2-sin-cusp1-bd

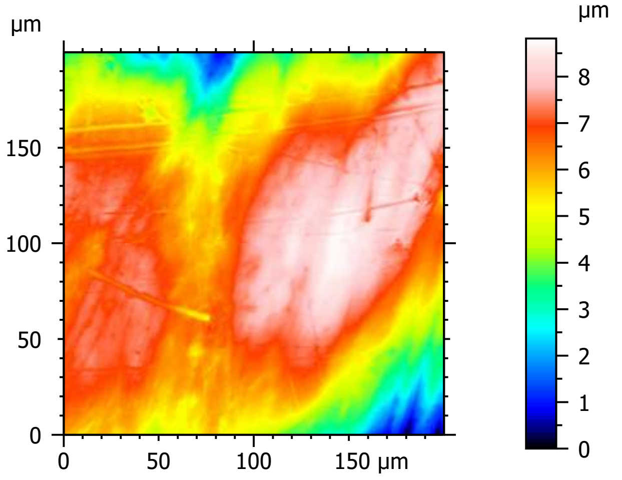

CG-Rt-c27-1018-lm2-sin-cusp1-bd

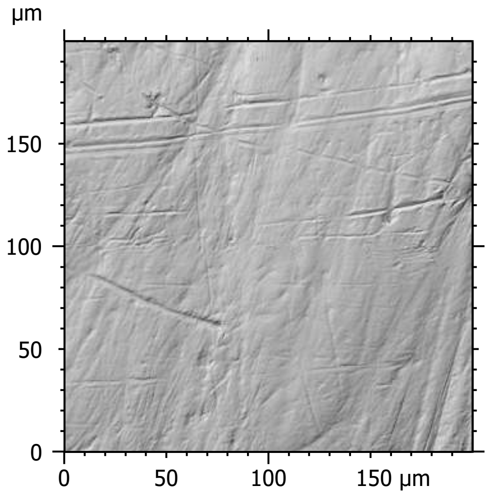

CG-Rt-c27-1019-lm2-dex-cusp2-bd

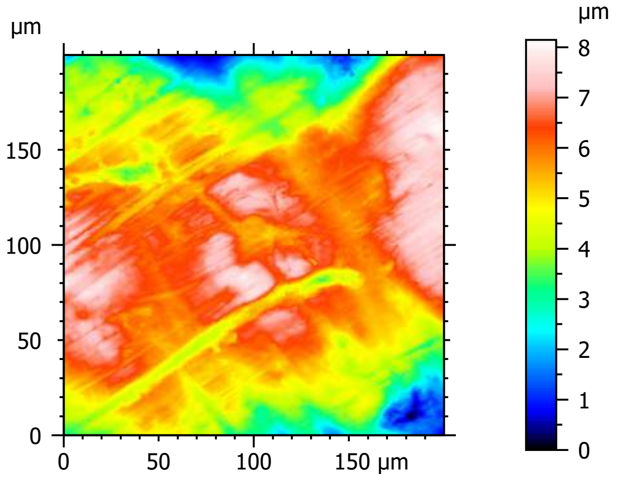

CG-Rt-c27-1019-lm2-dex-cusp2-bd

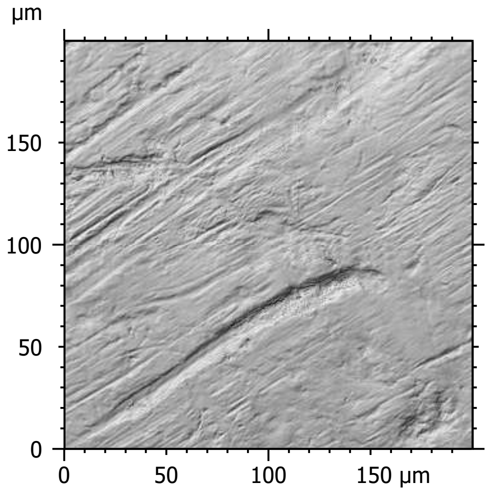

Leica Map Premium 8.2.9843

CG-Rt-c27-1020-lm3-sin-cusp1-bd

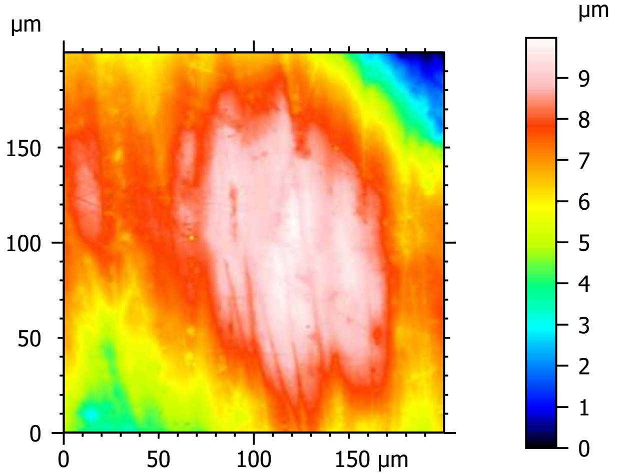

CG-Rt-c27-1020-lm3-sin-cusp1-bd

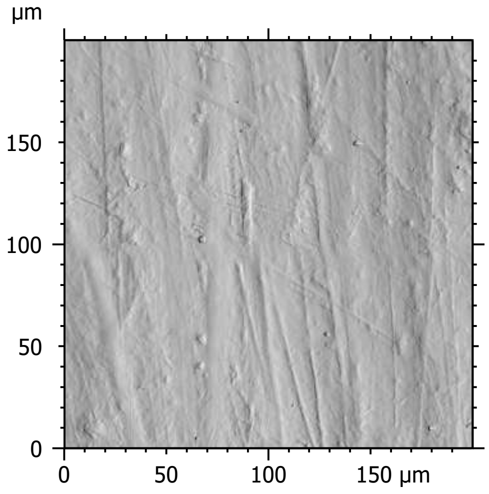

CG-Rt-c28-328-lm2-sin-cusp1-bd

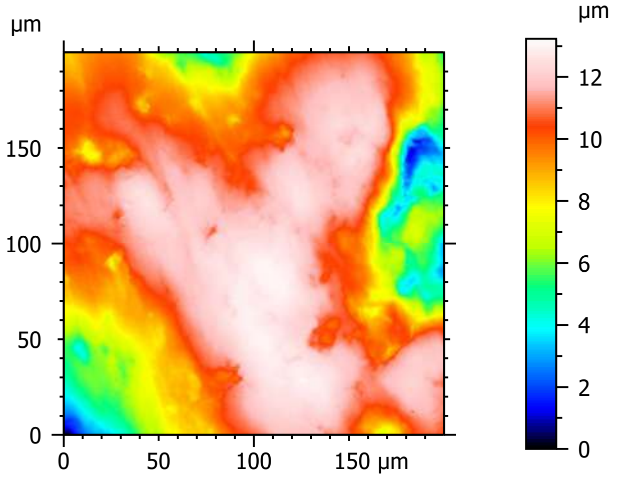

CG-Rt-c28-328-lm2-sin-cusp1-bd

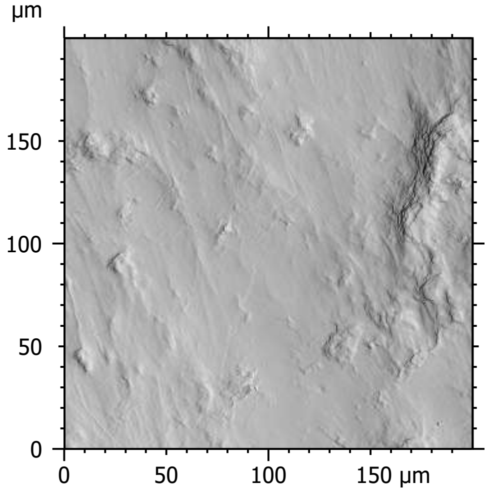

CG-Rt-c28-329-lm2-sin-cusp1-bd

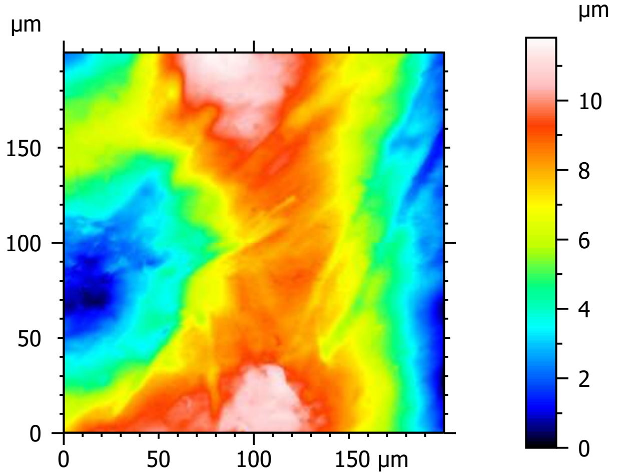

CG-Rt-c28-329-lm2-sin-cusp1-bd

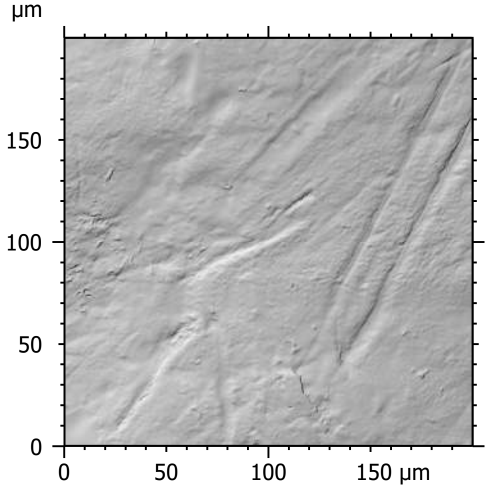

Leica Map Premium 8.2.9843

CG-Rt-c28-330-lm2-sin-cusp1-bd

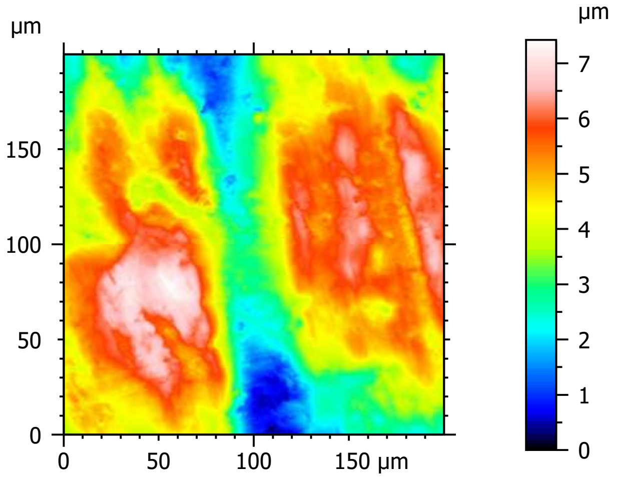

CG-Rt-c28-330-lm2-sin-cusp1-bd

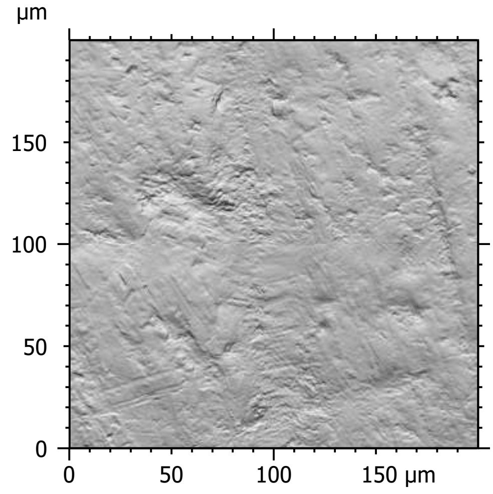

CG-Rt-c28-332-lm2-sin-cusp1-bd

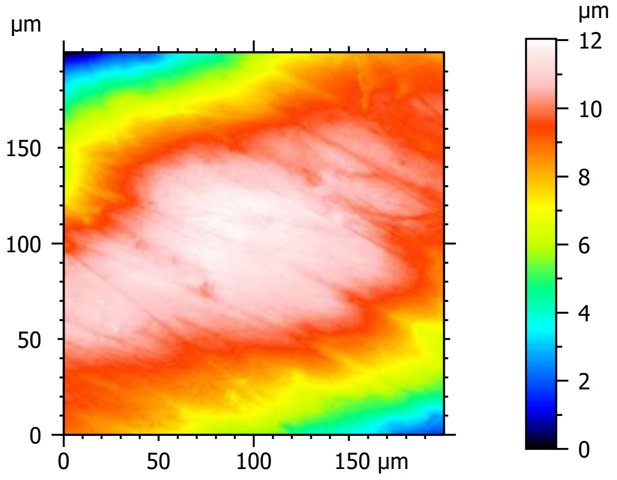

CG-Rt-c28-332-lm2-sin-cusp1-bd

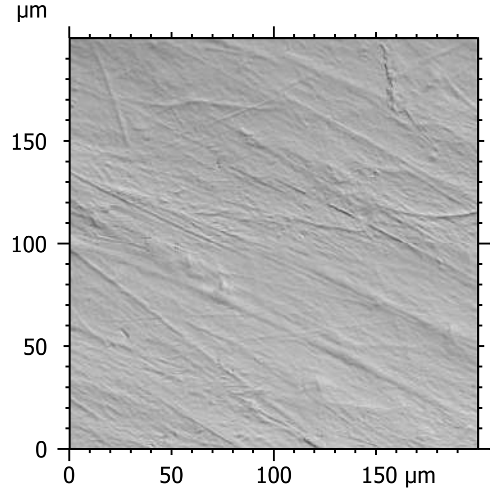

CG-Rt-c28-333-lm2-sin-cusp1-bd

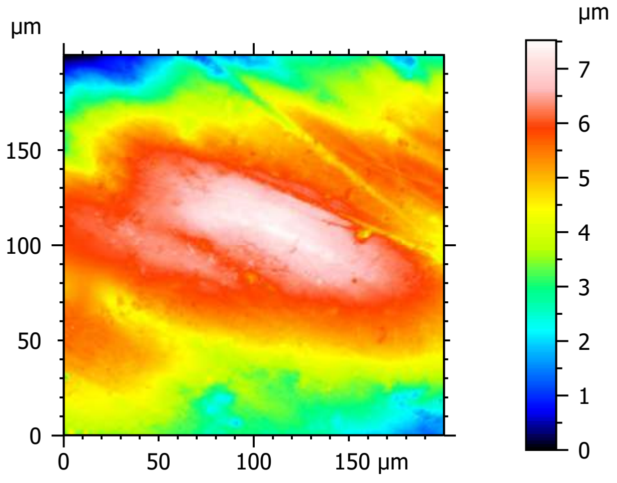

CG-Rt-c28-333-lm2-sin-cusp1-bd

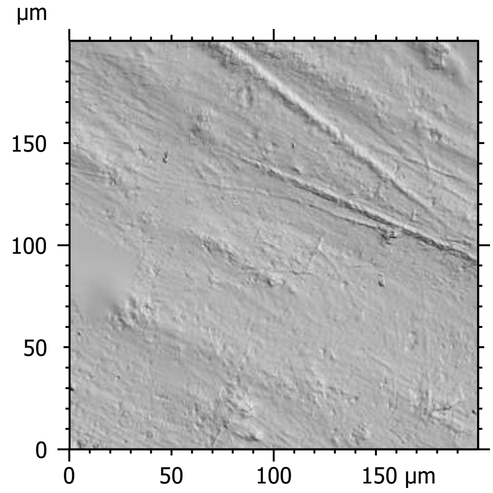

Leica Map Premium 8.2.9843

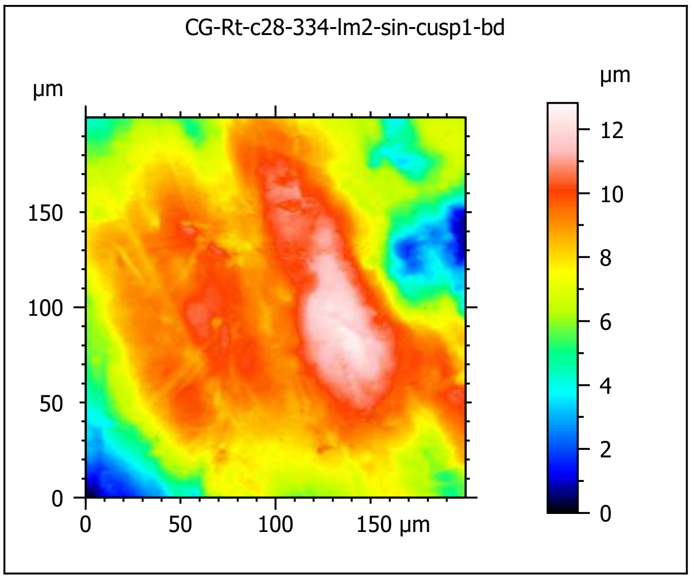

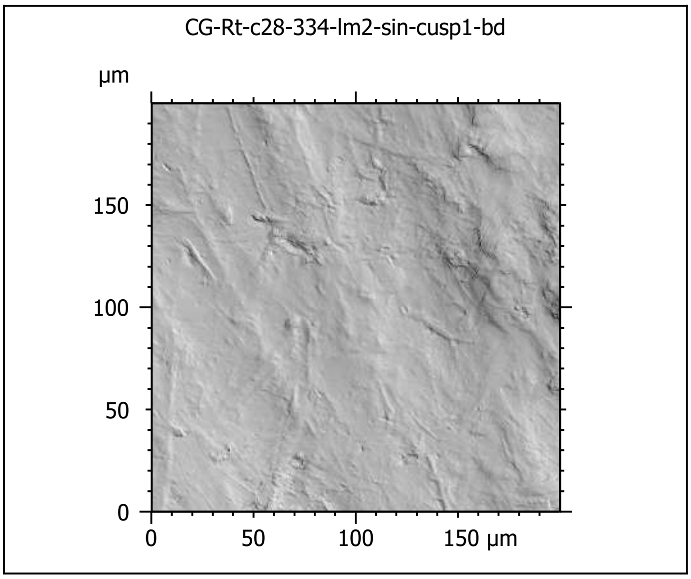

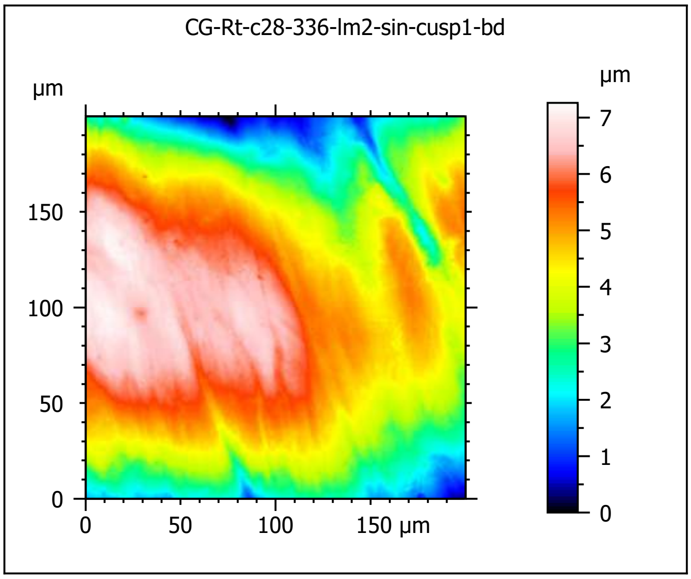

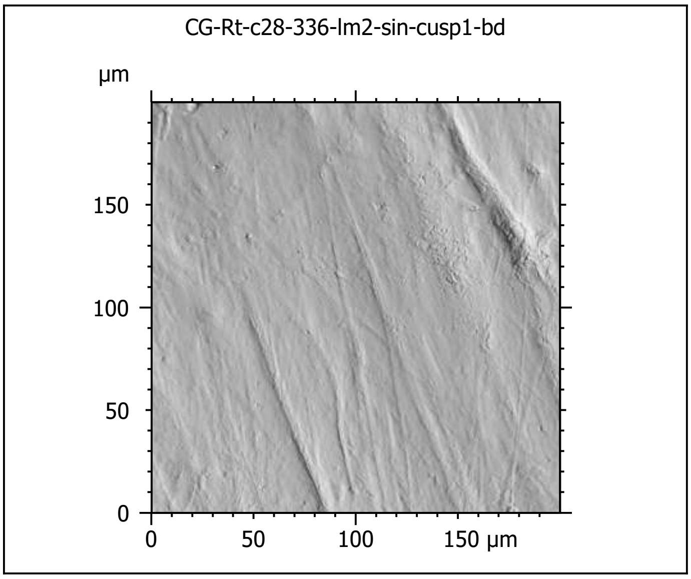

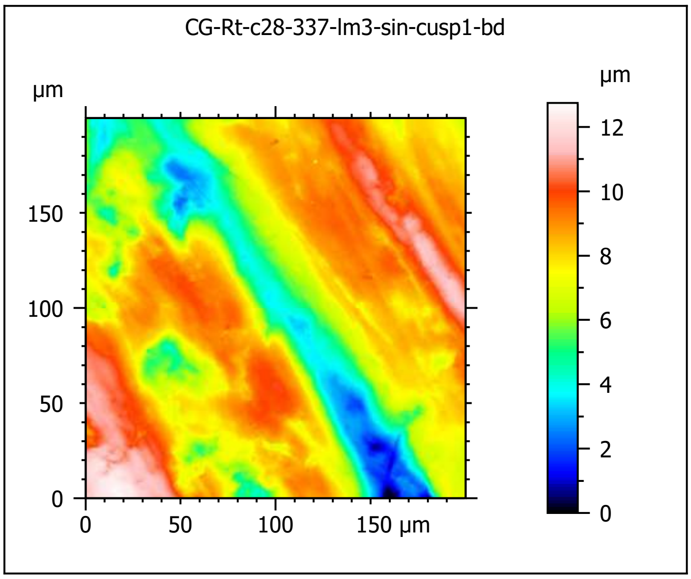

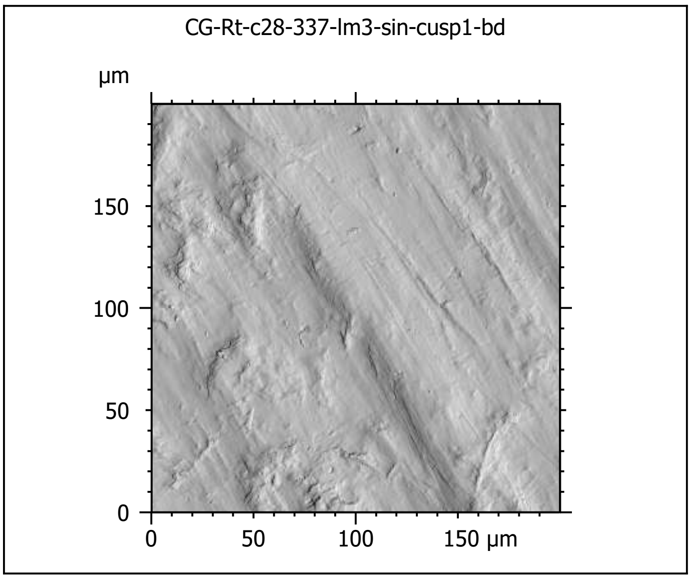

Leica Map Premium 8.2.9843

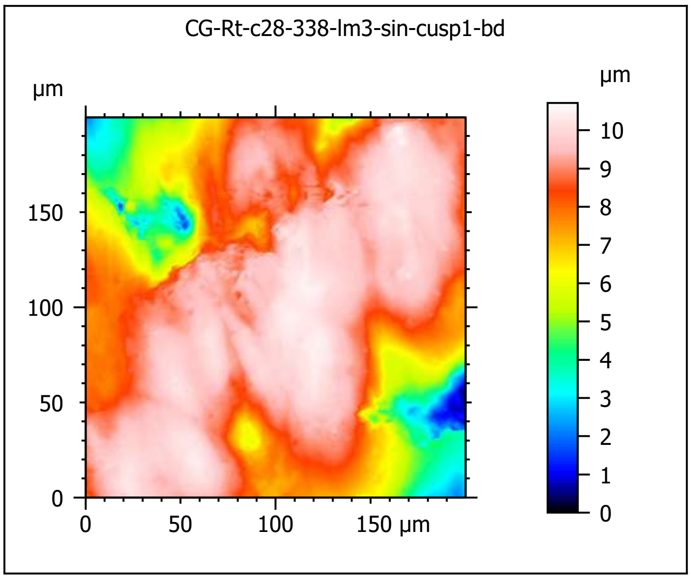

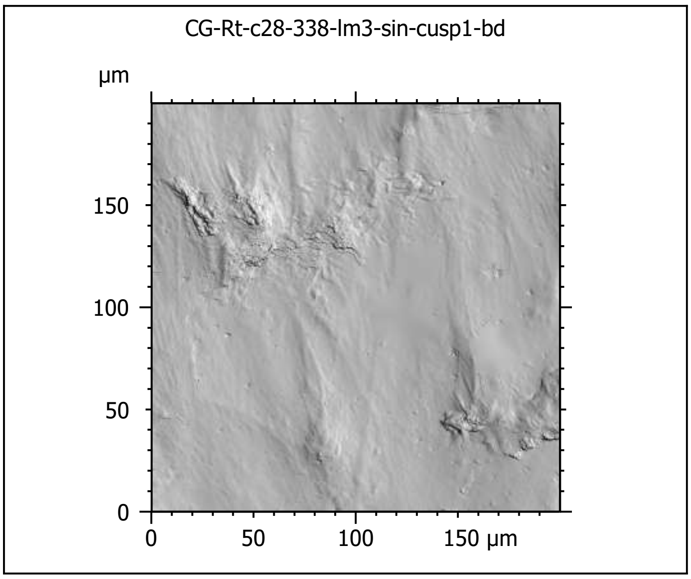

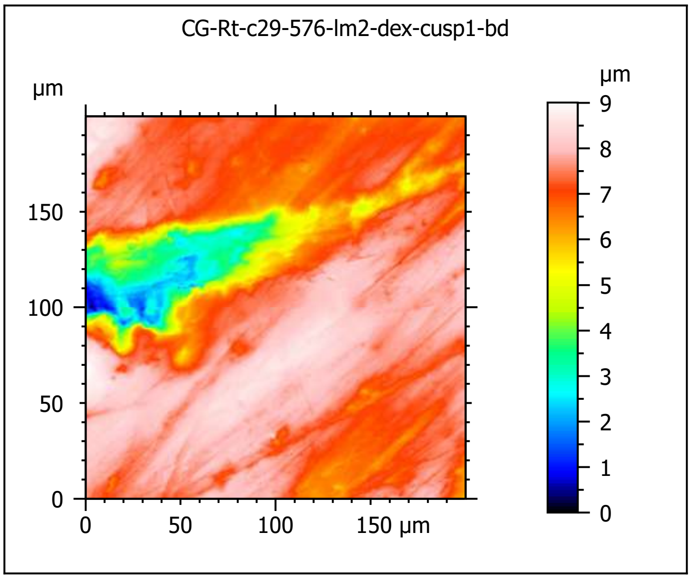

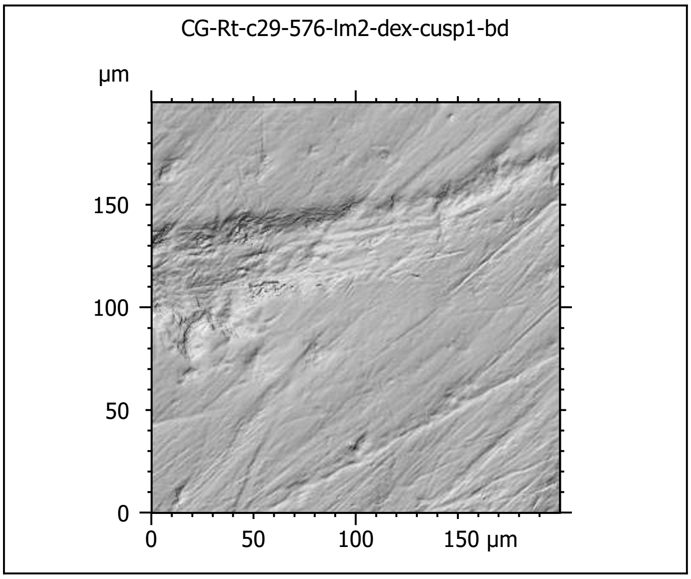

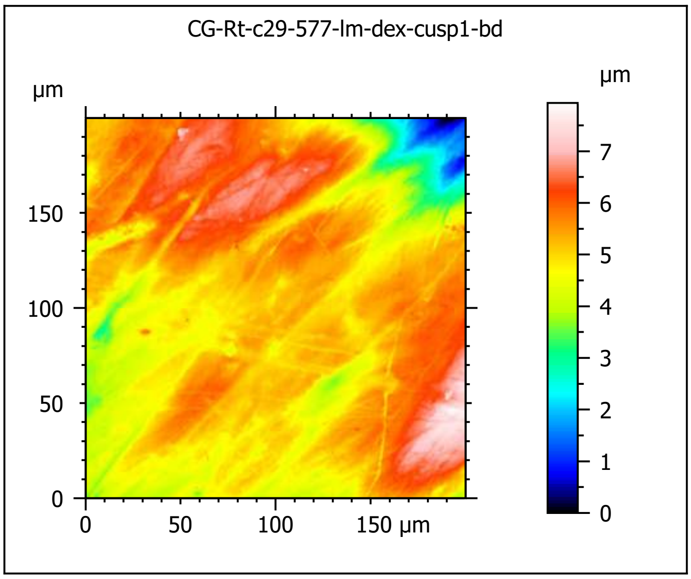

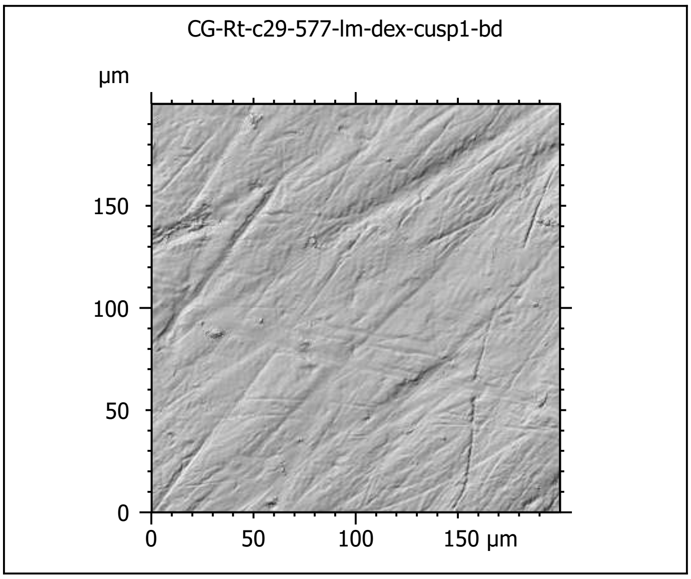

Leica Map Premium 8.2.9843

CG-Rt-c29-578-lm2-dex-cusp1-bd

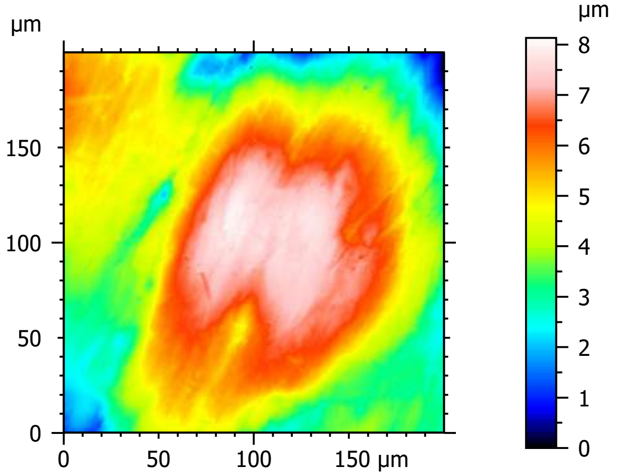

CG-Rt-c29-578-lm2-dex-cusp1-bd

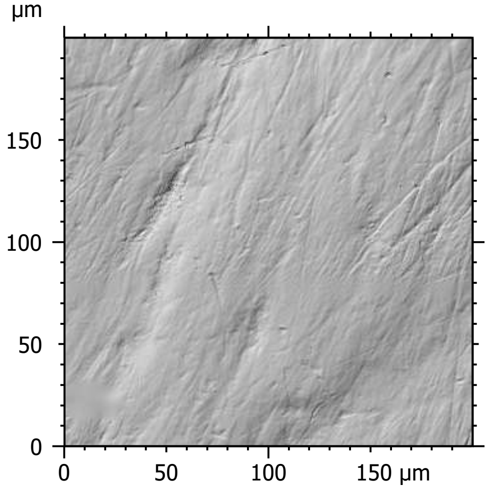

CG-Rt-c29-579-lm-dex-cusp1-bd

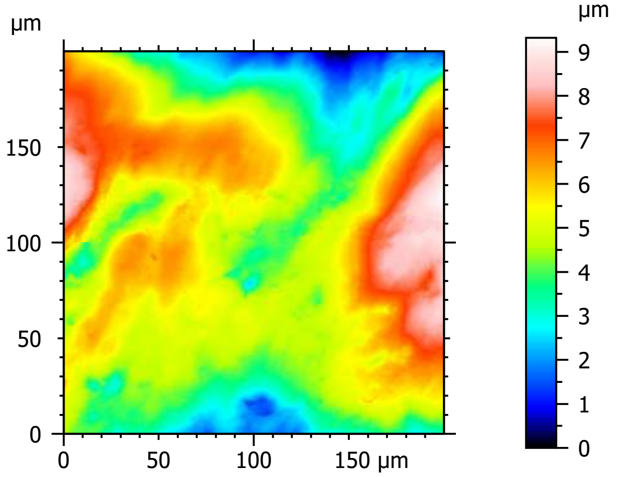

CG-Rt-c29-579-lm-dex-cusp1-bd

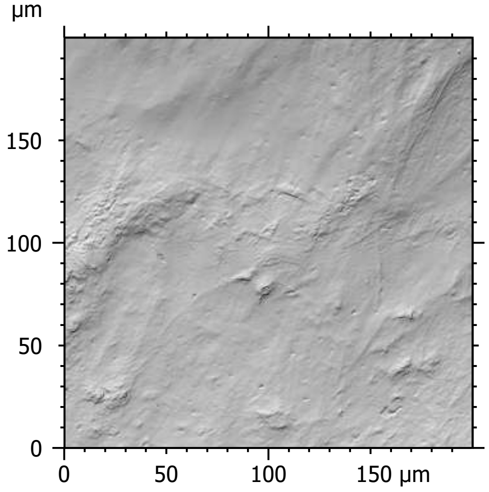

CG-Rt-c29-580-lm3-dex-cusp1-bd

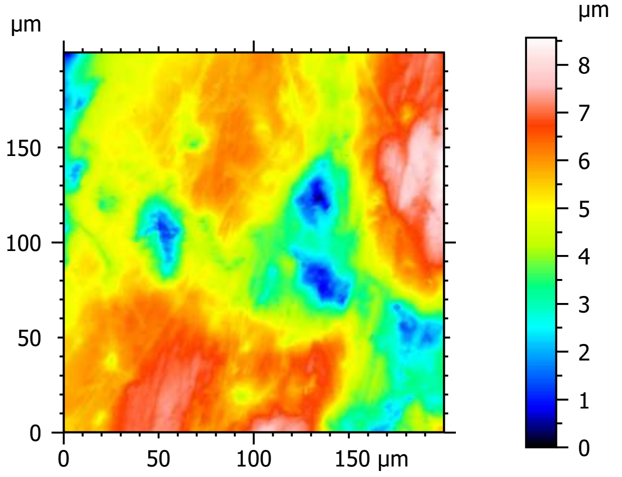

CG-Rt-c29-580-lm3-dex-cusp1-bd

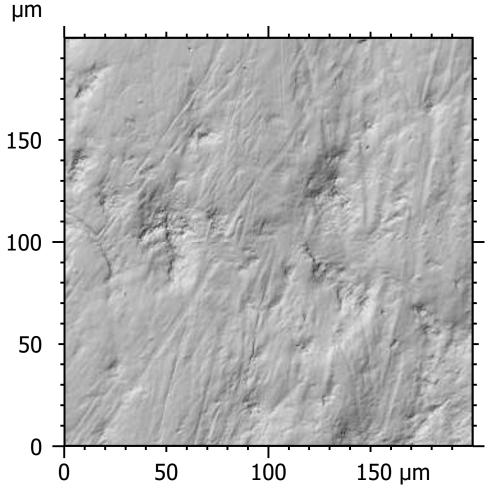

Leica Map Premium 8.2.9843

CG-Rt-c29-584-lm2-dex-cusp1-bd

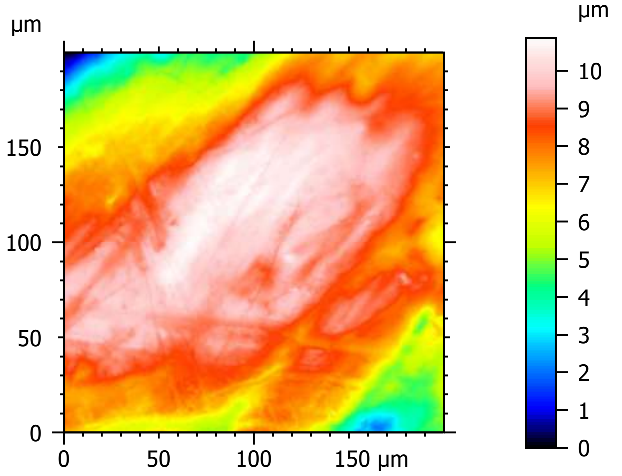

CG-Rt-c29-584-lm2-dex-cusp1-bd

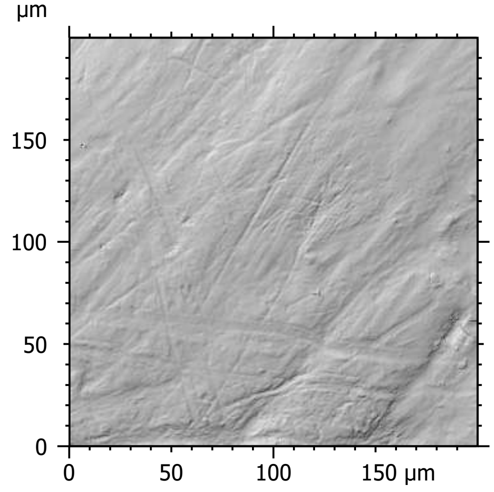

CG-Rt-c30-586-lm1-dex-cusp1-bd

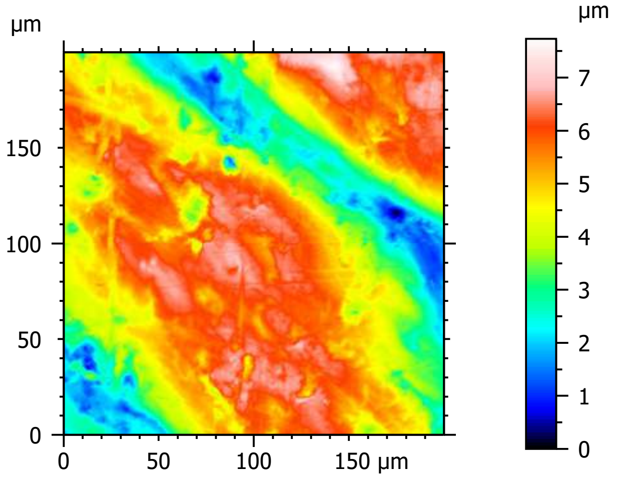

CG-Rt-c30-586-lm1-dex-cusp1-bd

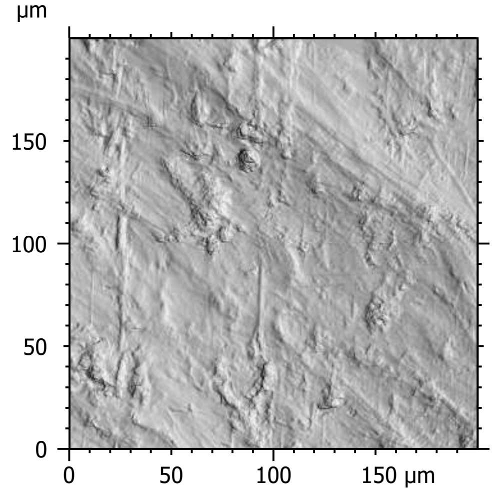

CG-Rt-c30-587-lm-sin-cusp1-bd

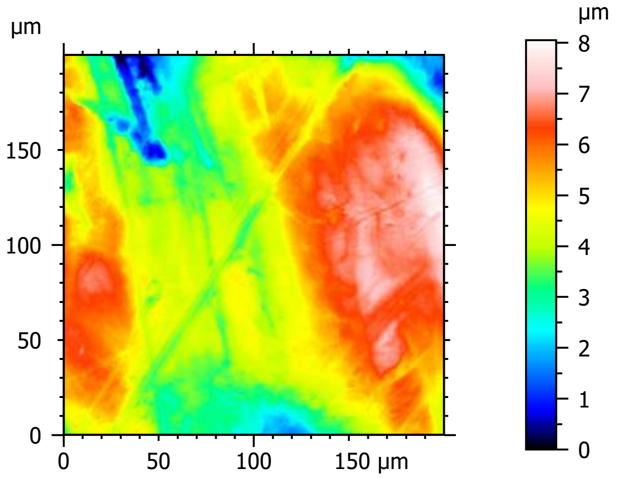

CG-Rt-c30-587-lm-sin-cusp1-bd

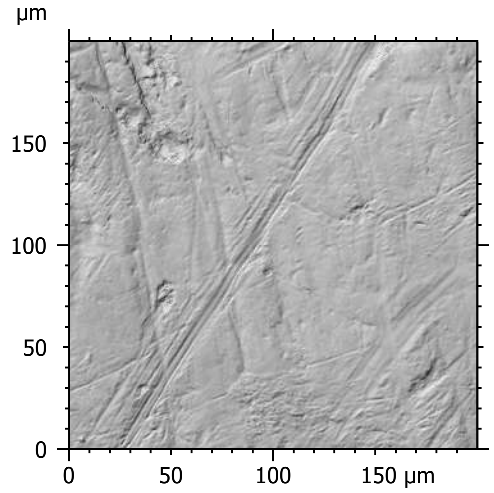

Leica Map Premium 8.2.9843

CG-Rt-c30-589-lm-dex-cusp1-bd

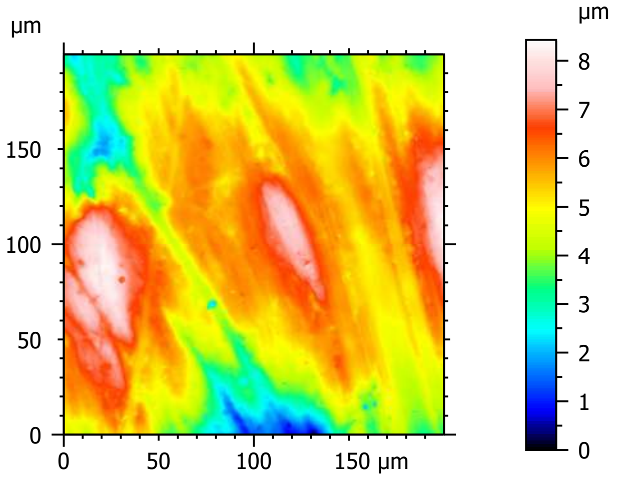

CG-Rt-c30-589-lm-dex-cusp1-bd

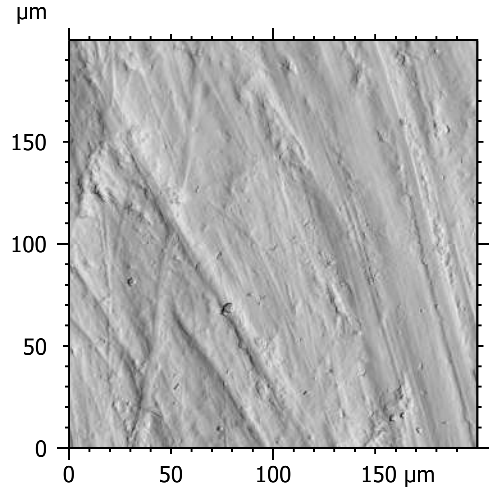

CG-Rt-c30-590-lm3-sin-cusp1-bd

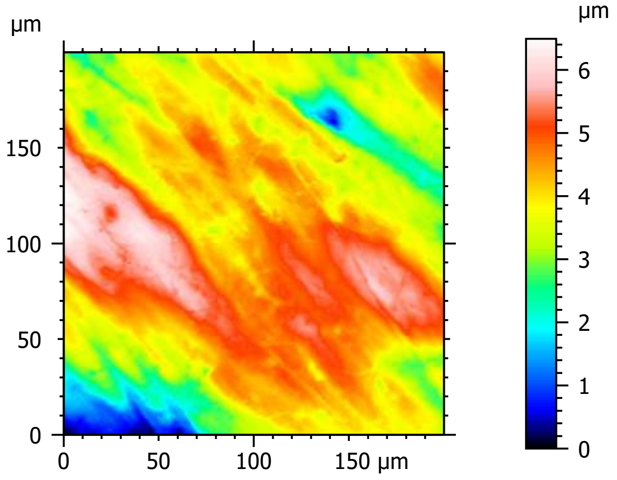

CG-Rt-c30-590-lm3-sin-cusp1-bd

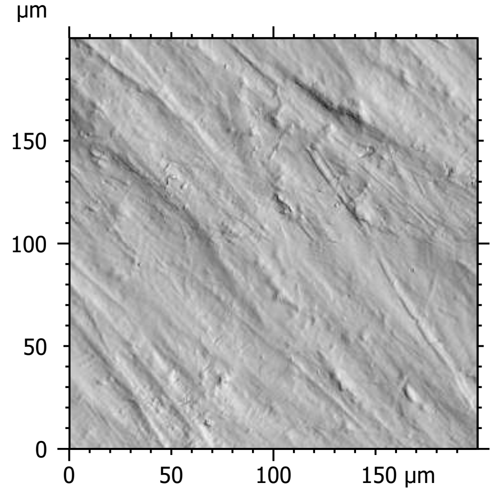

CG-Rt-c30-591-lm3-sin-cusp1-bd

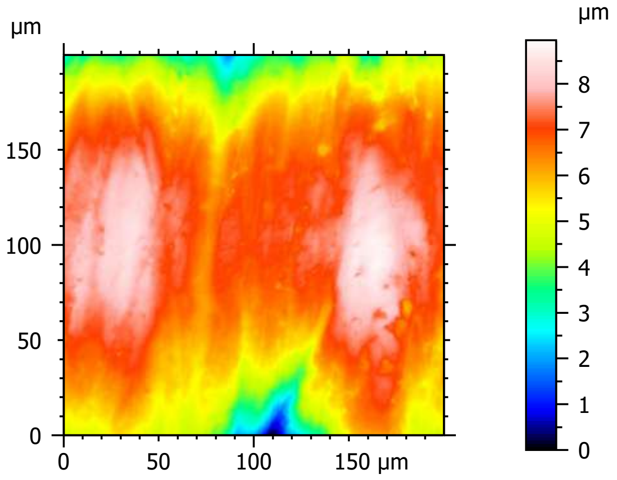

CG-Rt-c30-591-lm3-sin-cusp1-bd

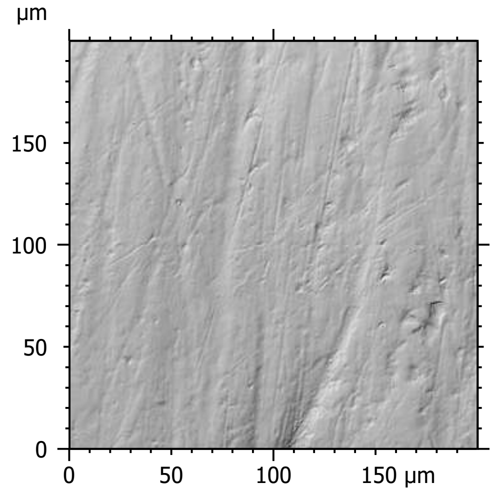

Leica Map Premium 8.2.9843

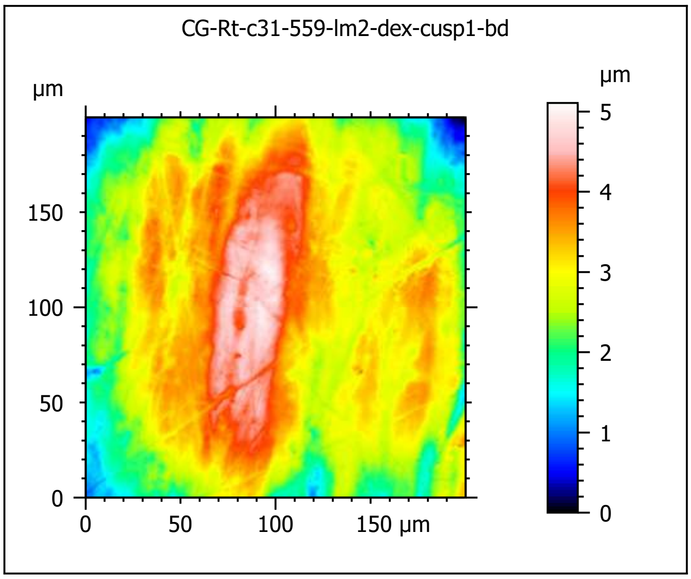

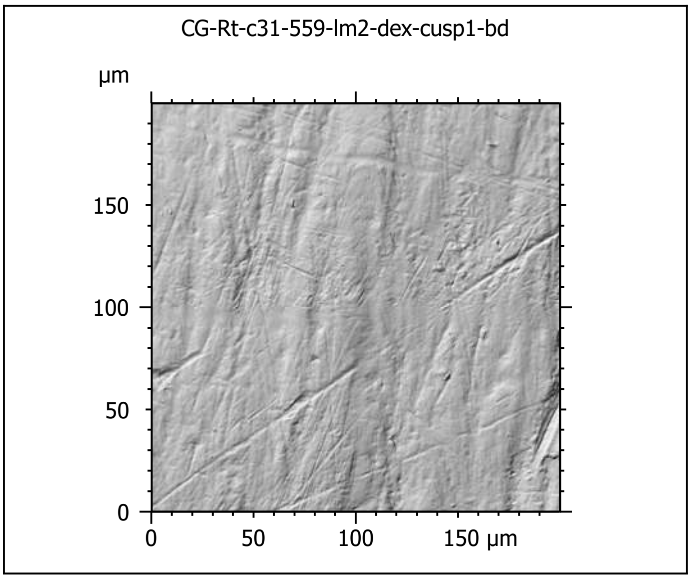

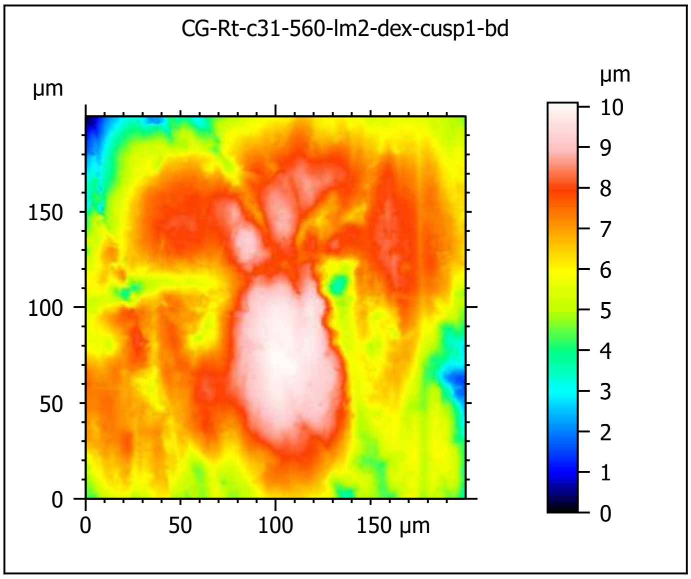

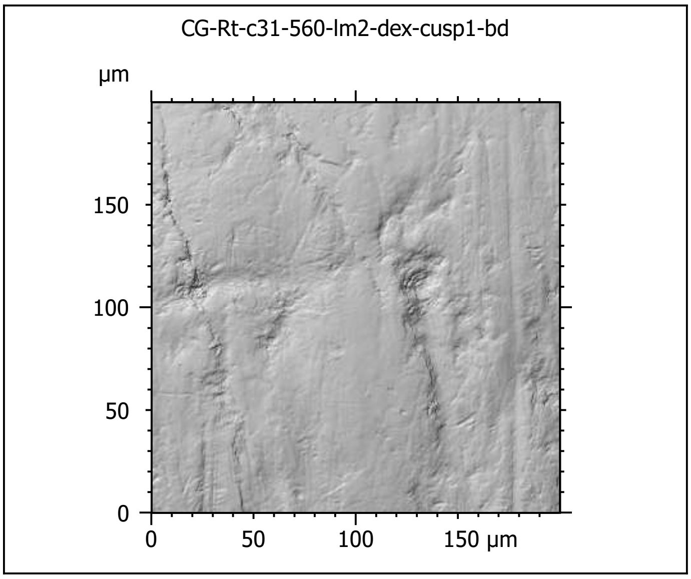

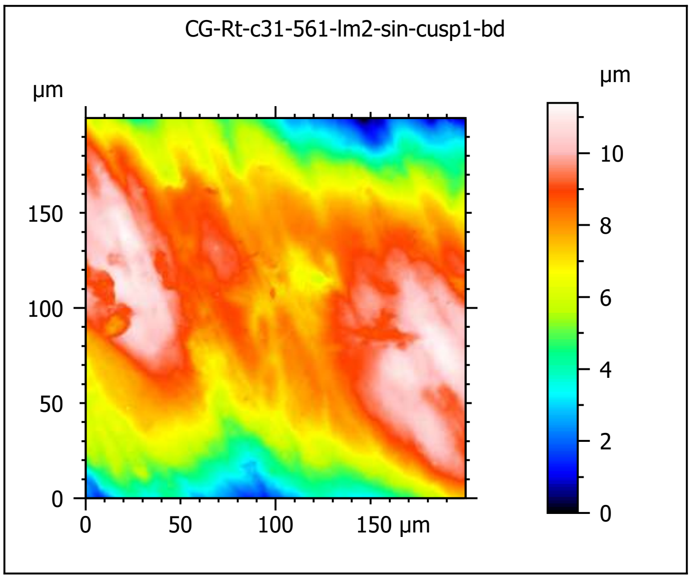

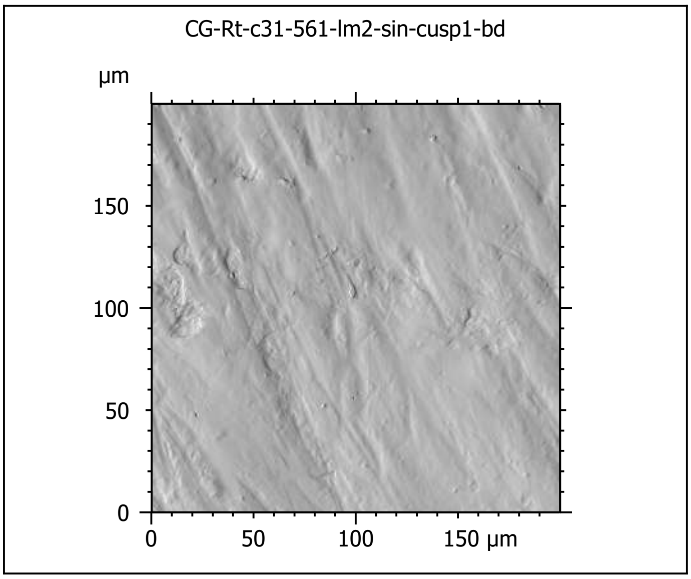

Leica Map Premium 8.2.9843

CG-Rt-c31-562-lm-sin-cusp1-bd

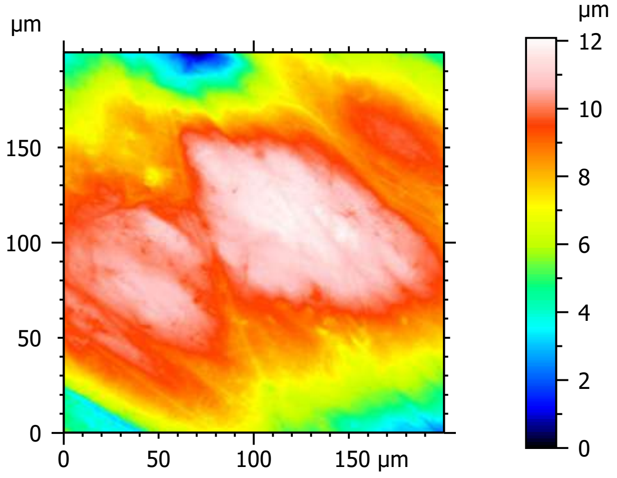

CG-Rt-c31-562-lm-sin-cusp1-bd

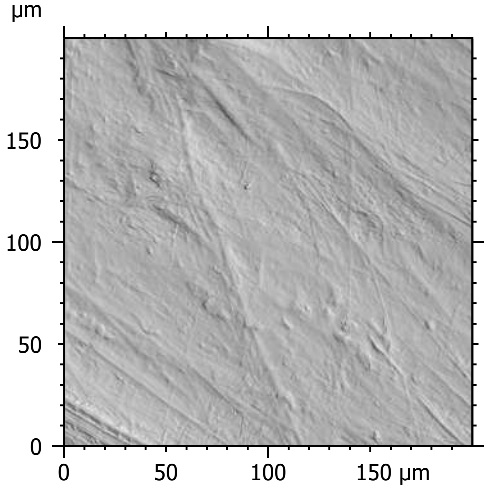

CG-Rt-c31-563-lm3-sin-cusp1-bd

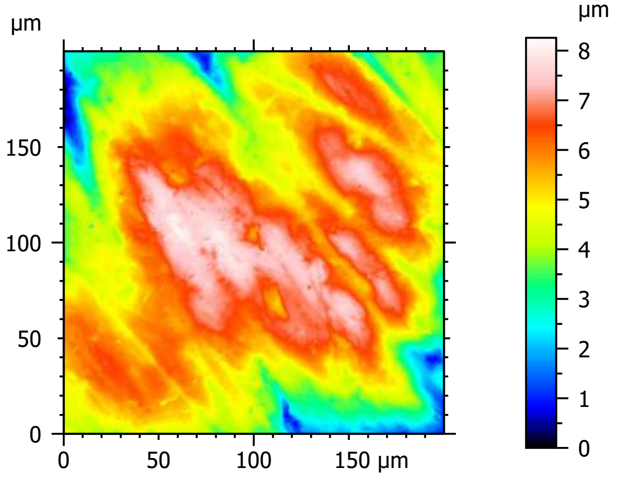

CG-Rt-c31-563-lm3-sin-cusp1-bd

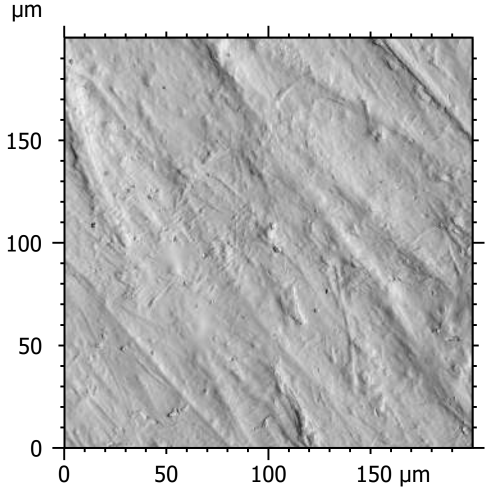

CG-Rt-c32-326-lm2-sin-cusp1-bd

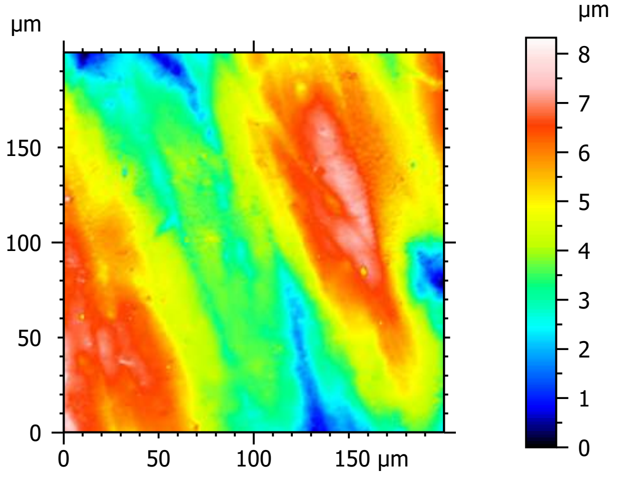

CG-Rt-c32-326-lm2-sin-cusp1-bd

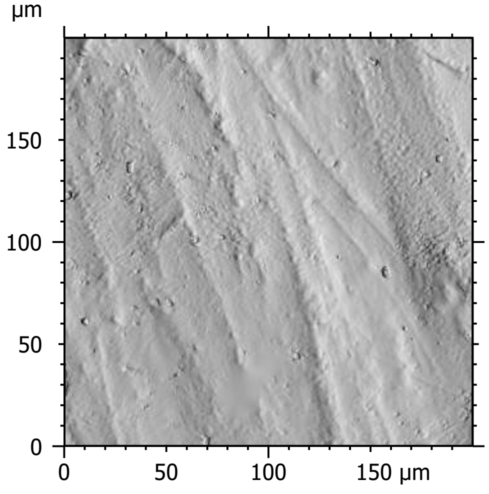

Leica Map Premium 8.2.9843

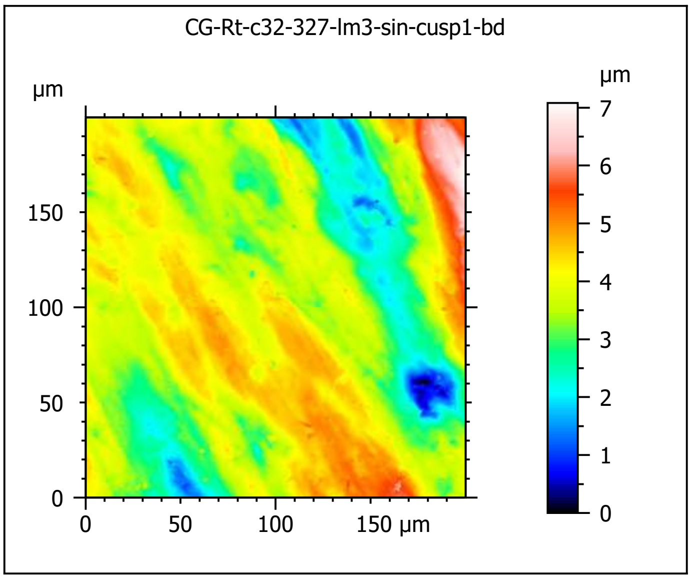

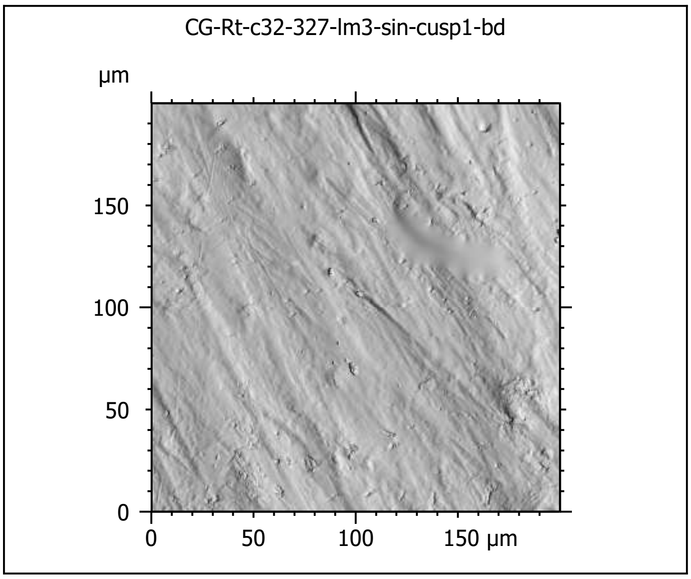

Leica Map Premium 8.2.9843

"A long-term perspective on Neandertal environment and subsistence: insights from the dental micro-texture analysis of hunted ungulates at Combe-Grenal (Dordogne, France)"

authors: Berlioz, E.; Capdepon, E.; Discamps, E.

Appendice 2:
surfaces scanned by E. Berlioz and E. Capdepon, pre-treatement by E. Berlioz and E. Capdepon, validation by E. Berlioz (2019)

***Rangifer tarandus* - Block H**

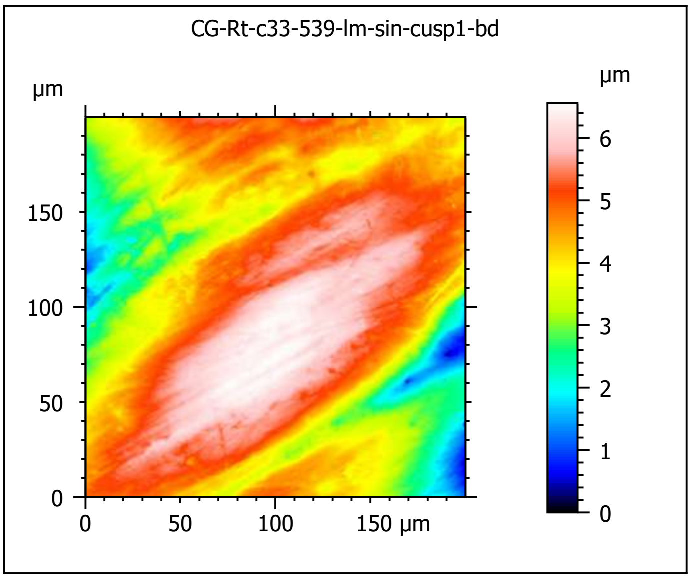

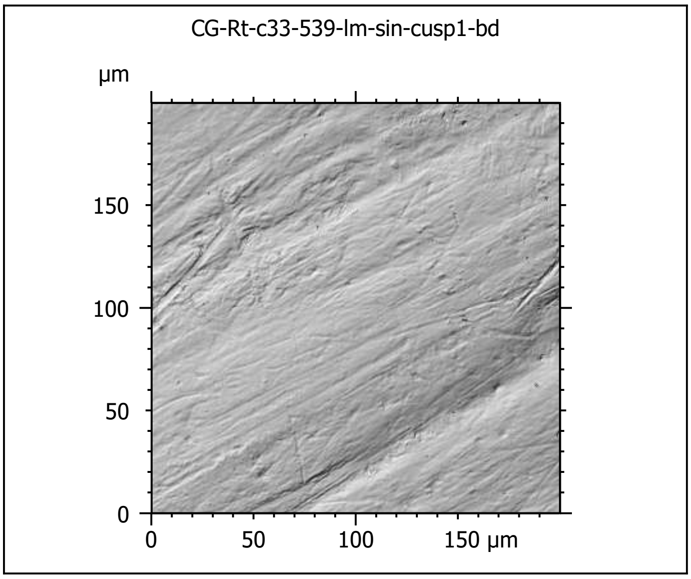

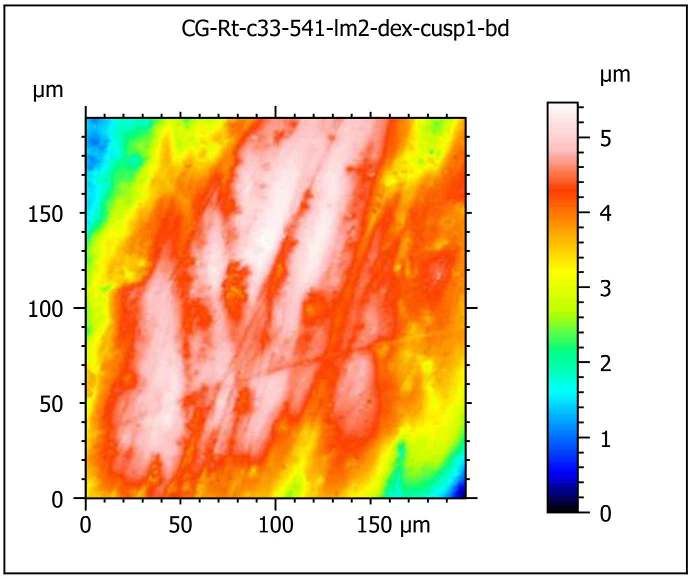

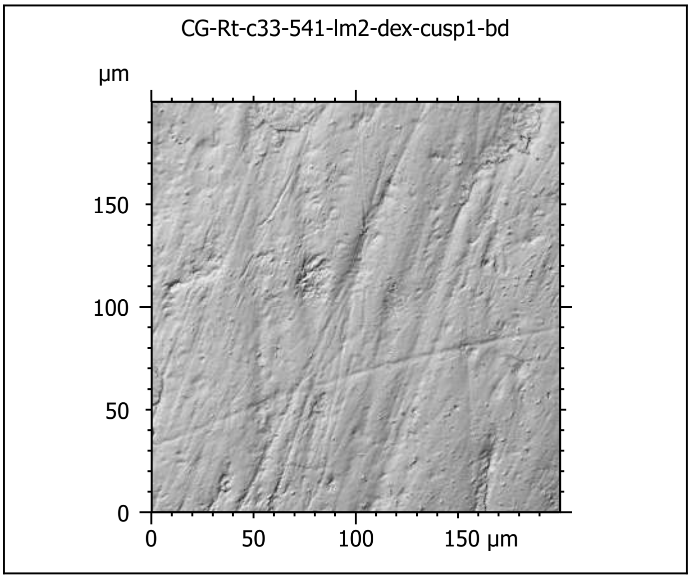

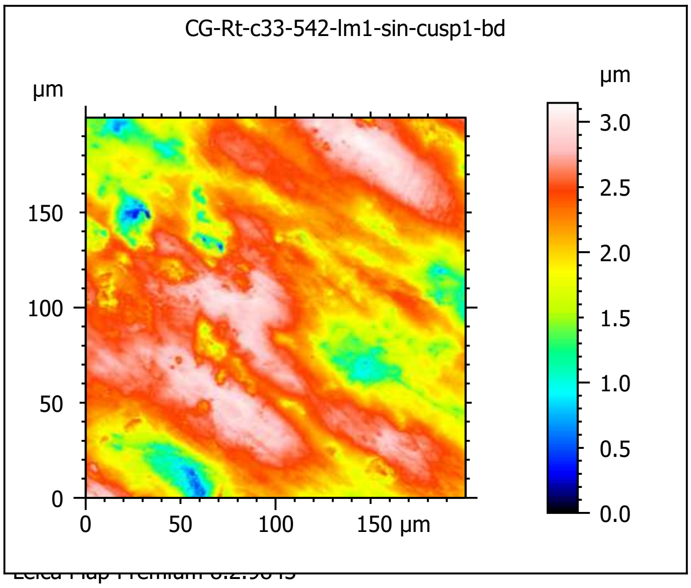

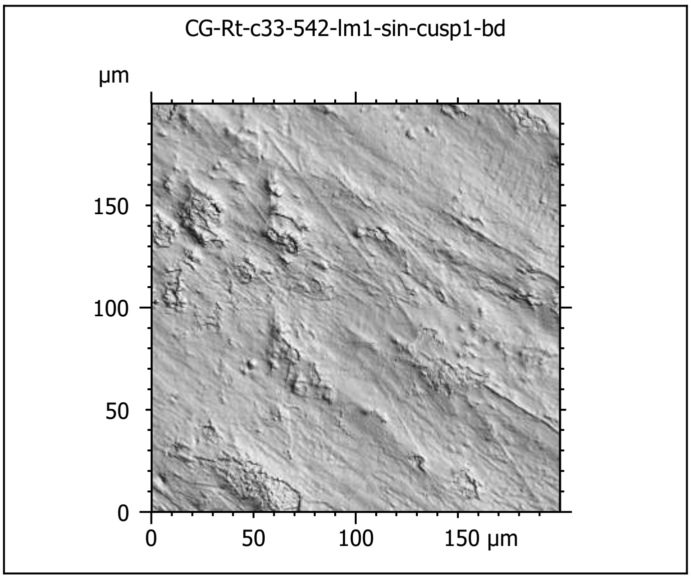

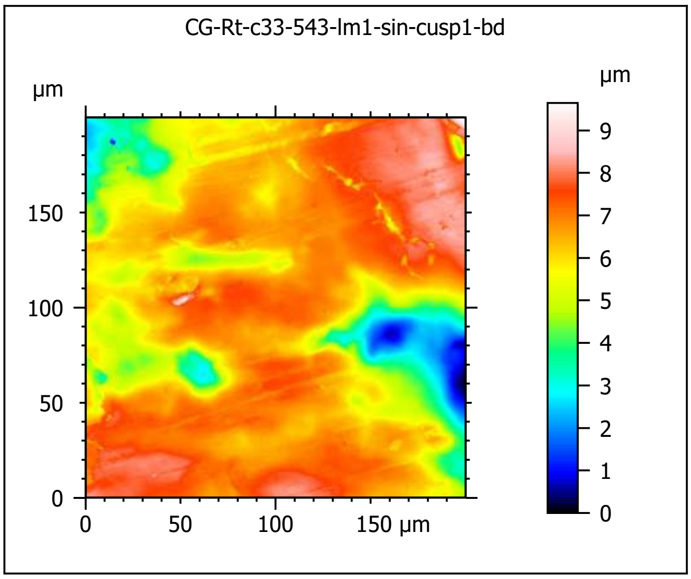

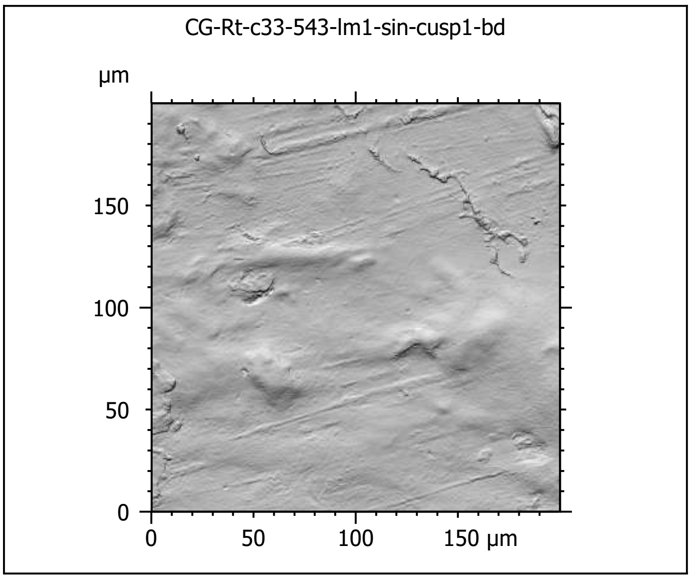

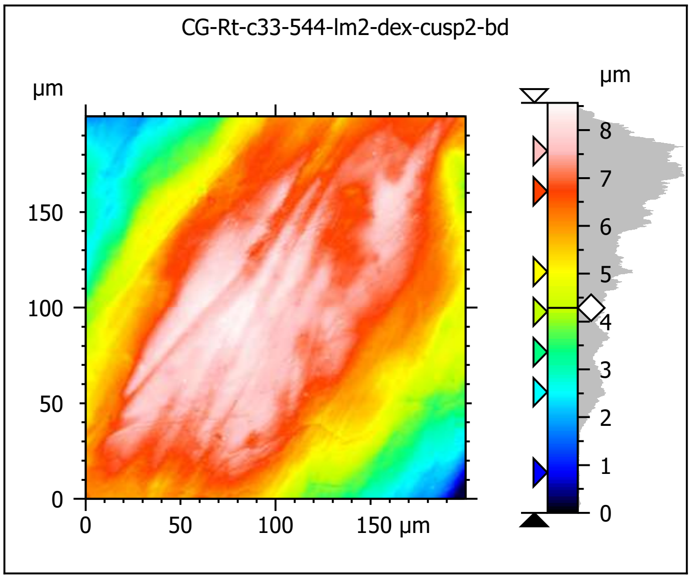

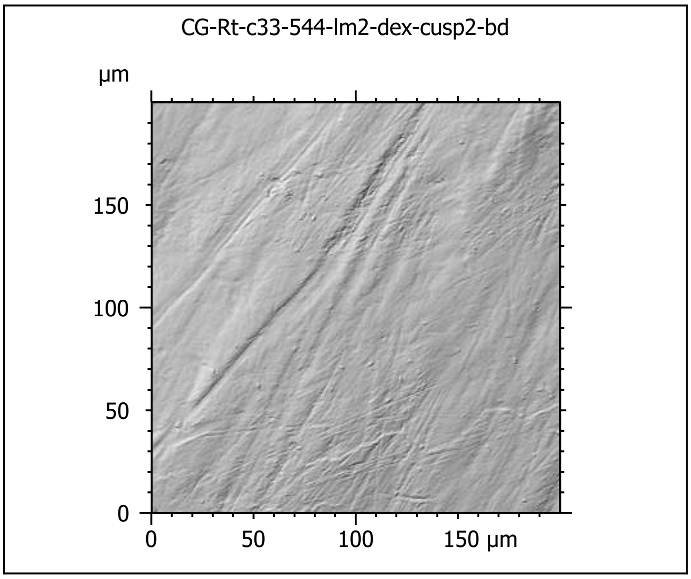

Leica Map Premium 8.2.9843

"A long-term perspective on Neandertal environment and subsistence: insights from the dental micro-texture analysis of hunted ungulates at Combe-Grenal (Dordogne, France)"

authors: Berlioz, E.; Capdepon, E.; Discamps, E.

Appendice 2:
surfaces scanned by E. Berlioz and E. Capdepon, pre-treatement by E. Berlioz and E. Capdepon, validation by E. Berlioz (2019)

***Rangifer tarandus* - Block I**

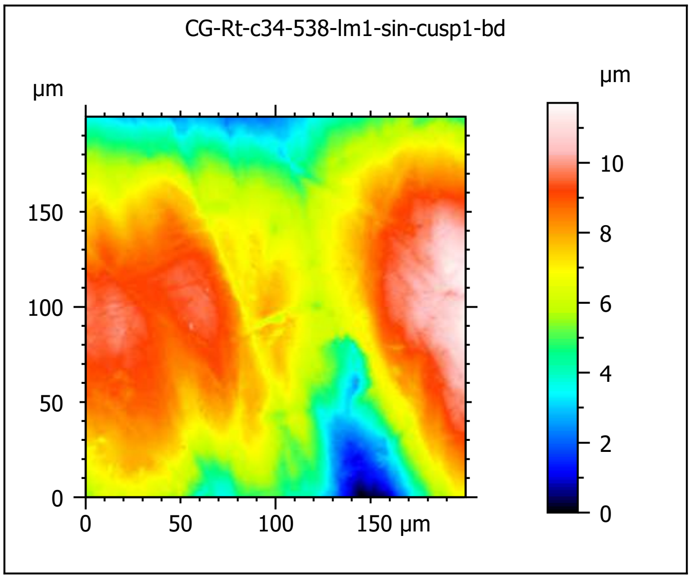

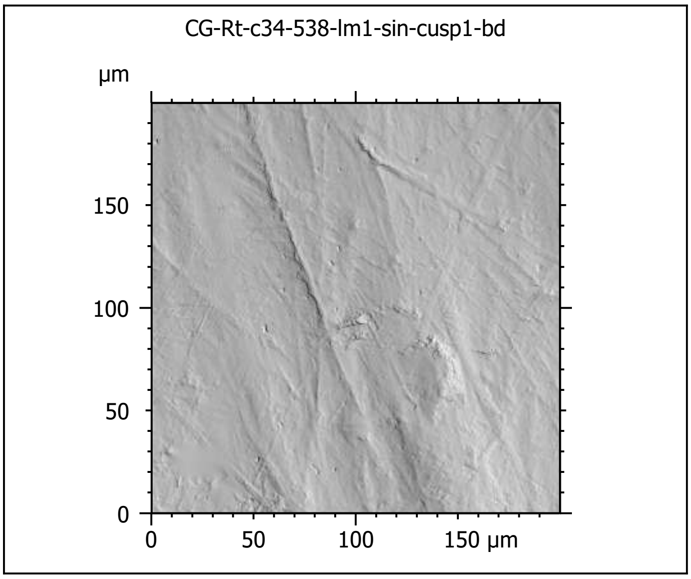

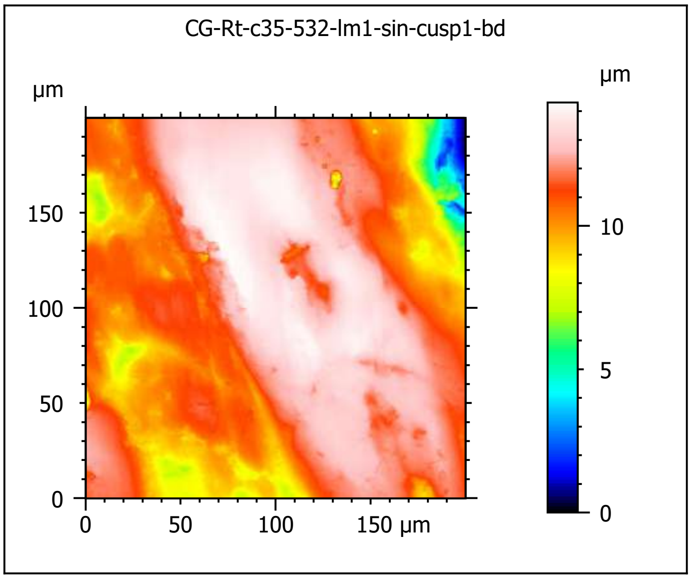

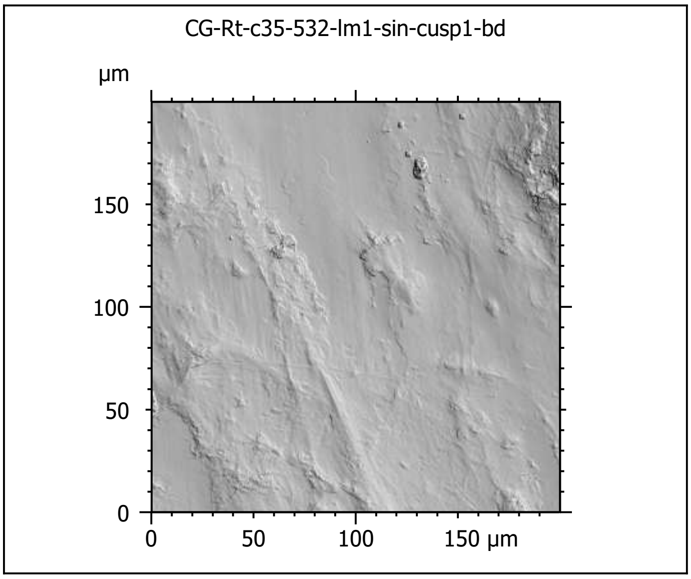

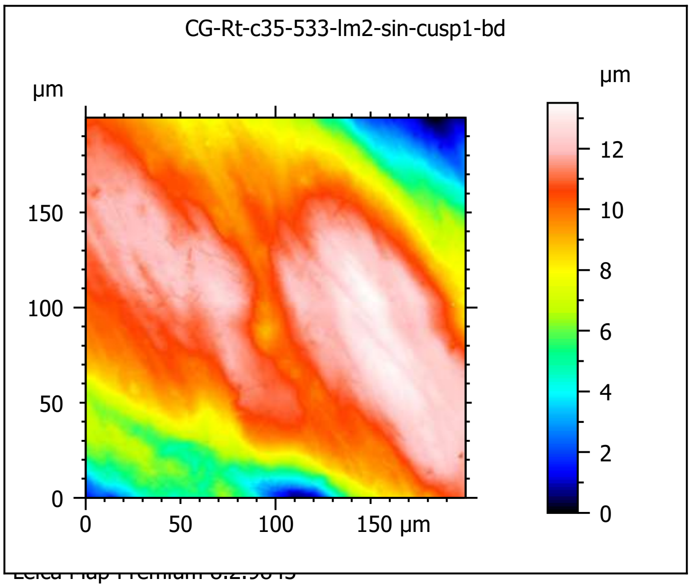

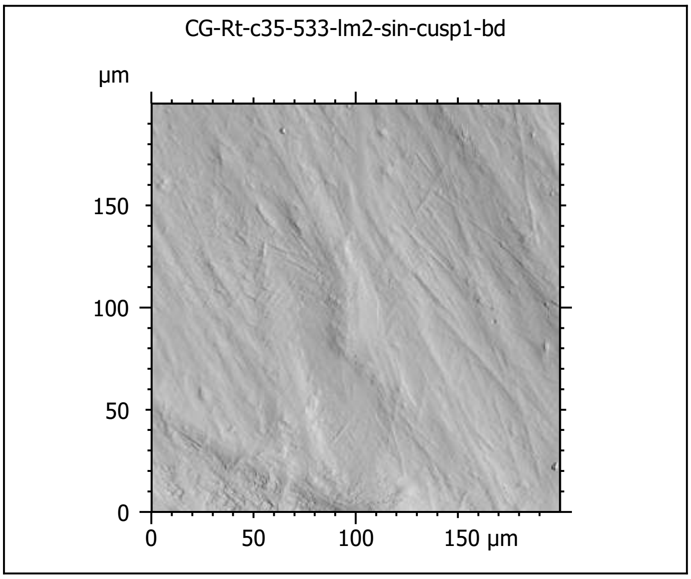

CG-Rt-c35-535-lm2-dex-cusp1-bd

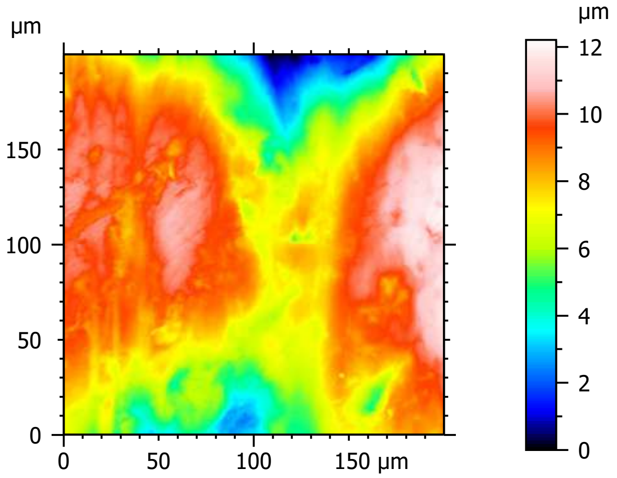

CG-Rt-c35-535-lm2-dex-cusp1-bd

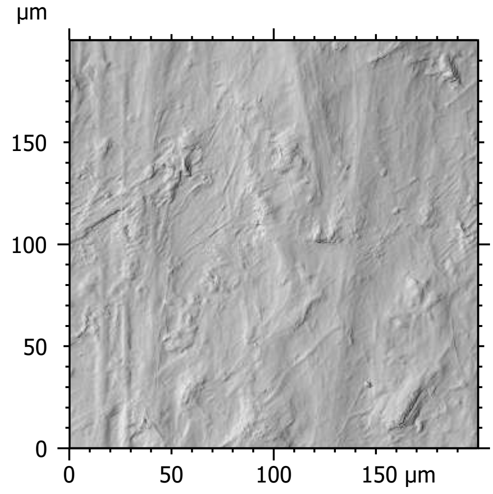

CG-Rt-c35-536-lm2-dex-cusp1-bd

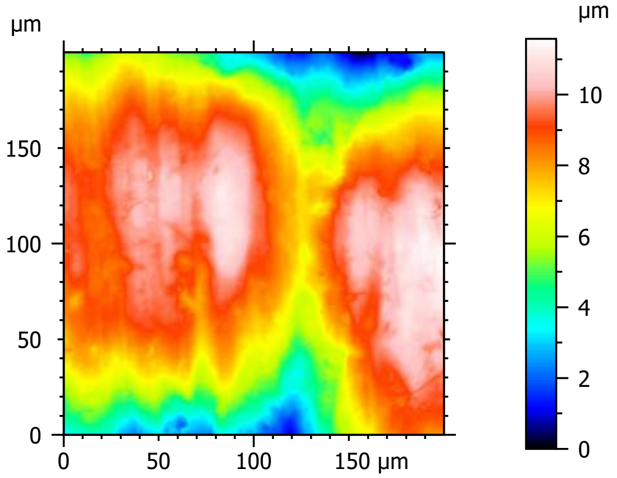

CG-Rt-c35-536-lm2-dex-cusp1-bd

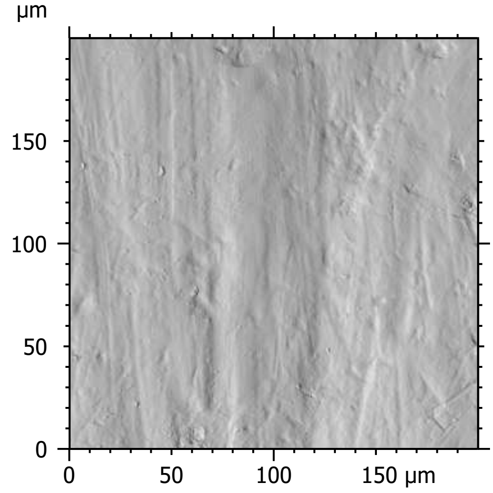

CG-Rt-c35-1026-lm2-sin-cusp1-bd

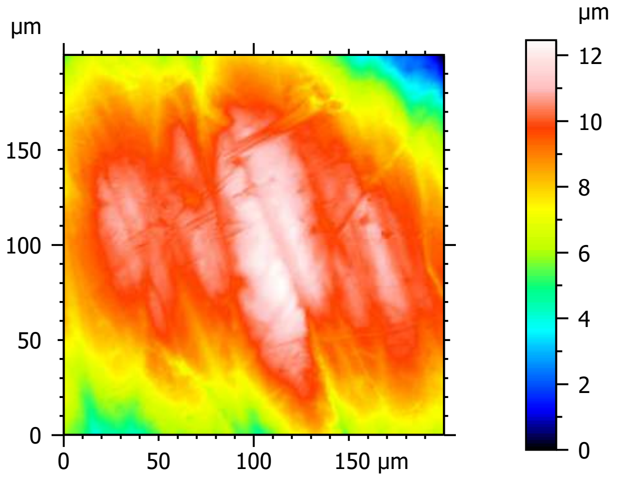

CG-Rt-c35-1026-lm2-sin-cusp1-bd

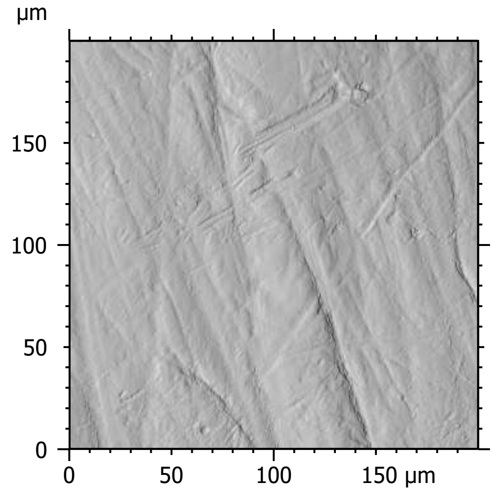

Leica Map Premium 8.2.9843

CG-Rt-c36-599-lm2-sin-cusp1-bd

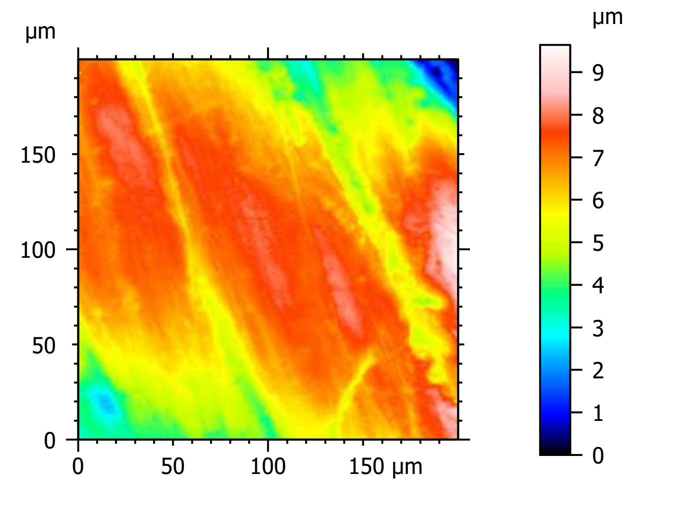

CG-Rt-c36-599-lm2-sin-cusp1-bd

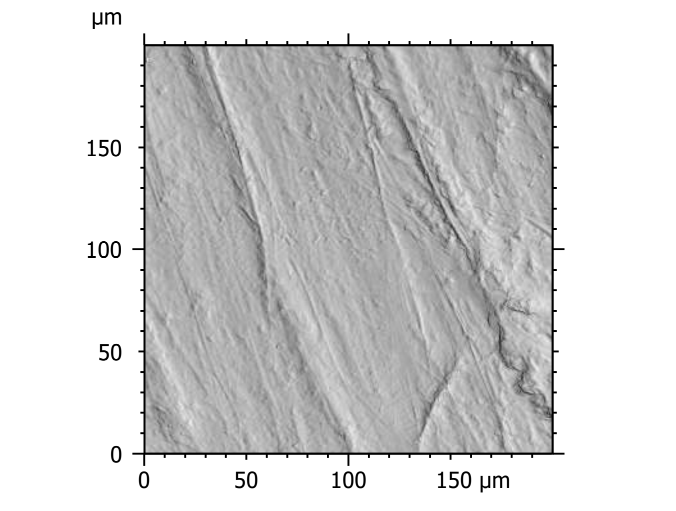

CG-Rt-c36-1027-lm2-dex-cusp2-bm

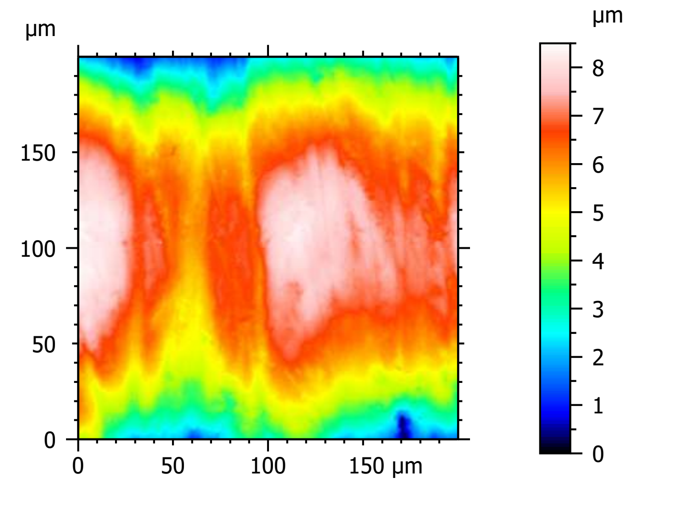

CG-Rt-c36-1027-lm2-dex-cusp2-bm

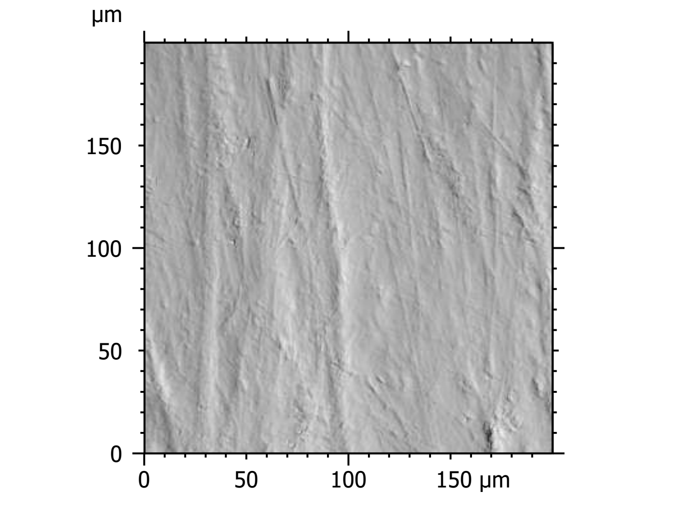

CG-Rt-c36-1028-lm2-sin-cusp1-bm

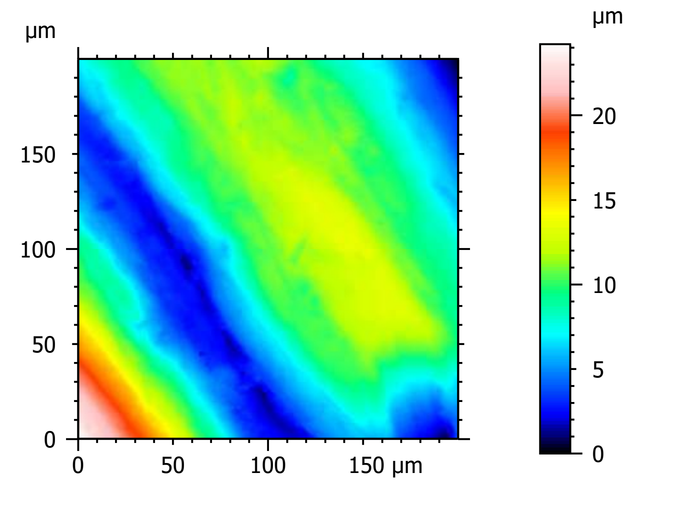

CG-Rt-c36-1028-lm2-sin-cusp1-bm

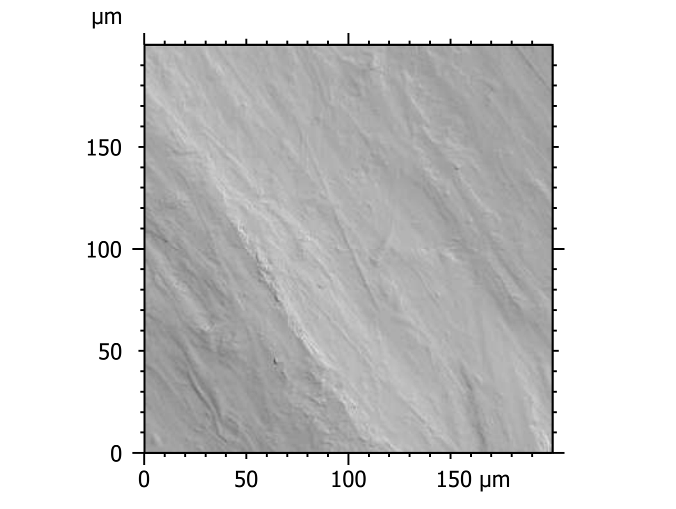

Leica Map Premium 8.2.9843

"A long-term perspective on Neandertal environment and subsistence: insights from the dental micro-texture analysis of hunted ungulates at Combe-Grenal (Dordogne, France)"

authors: Berlioz, E.; Capdepon, E.; Discamps, E.

Appendice 2:
surfaces scanned by E. Berlioz and E. Capdepon, pre-treatement by E. Berlioz and E. Capdepon, validation by E. Berlioz (2019)

***Cervus elaphus*- Block A**

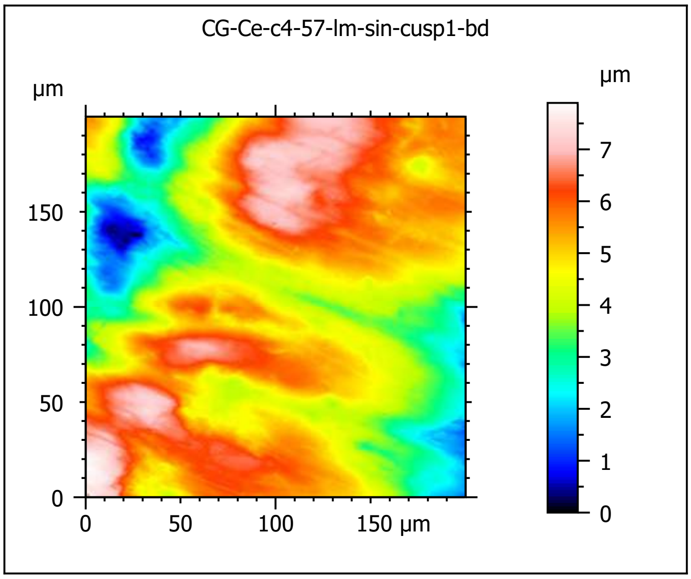

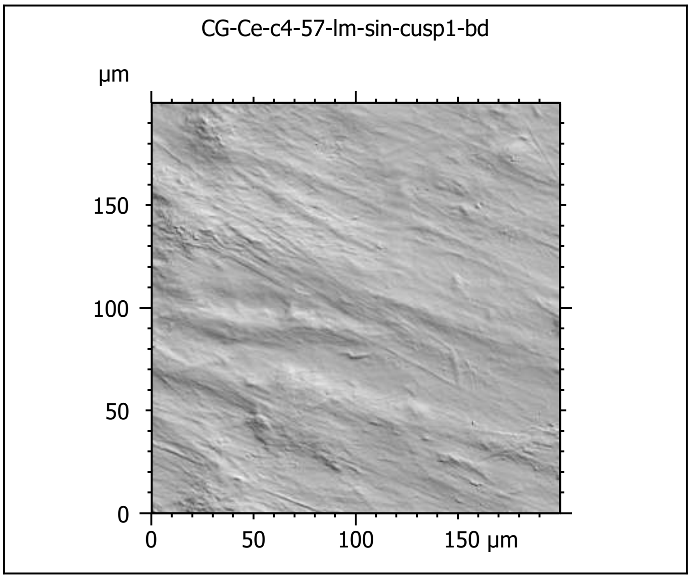

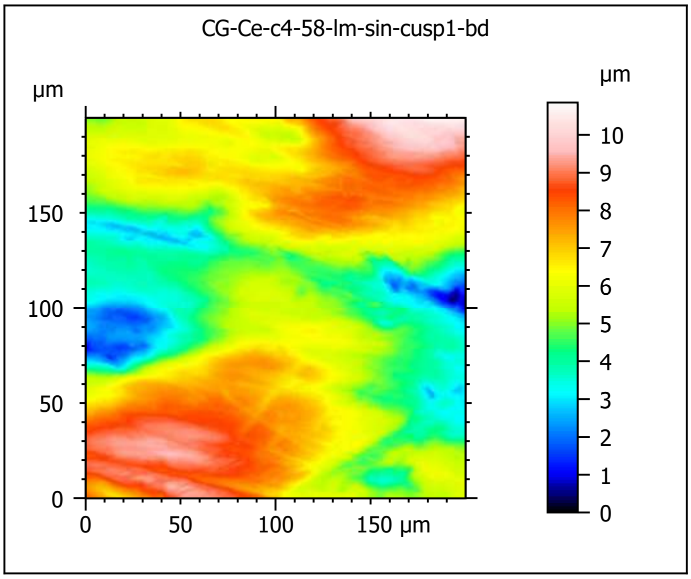

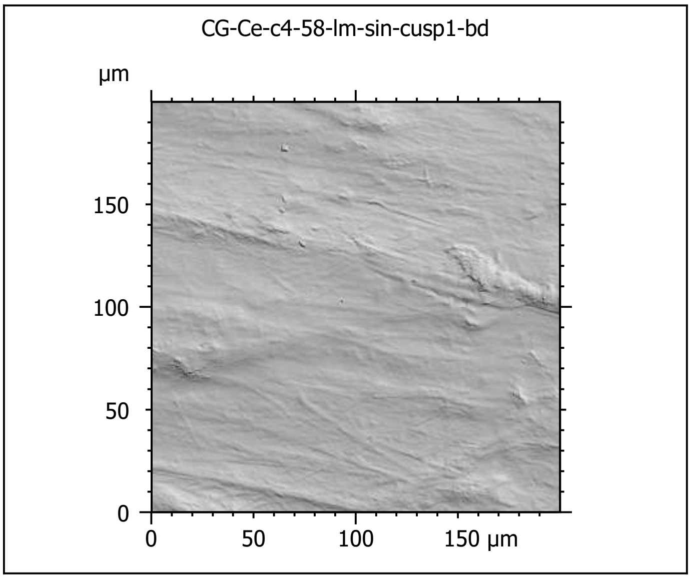

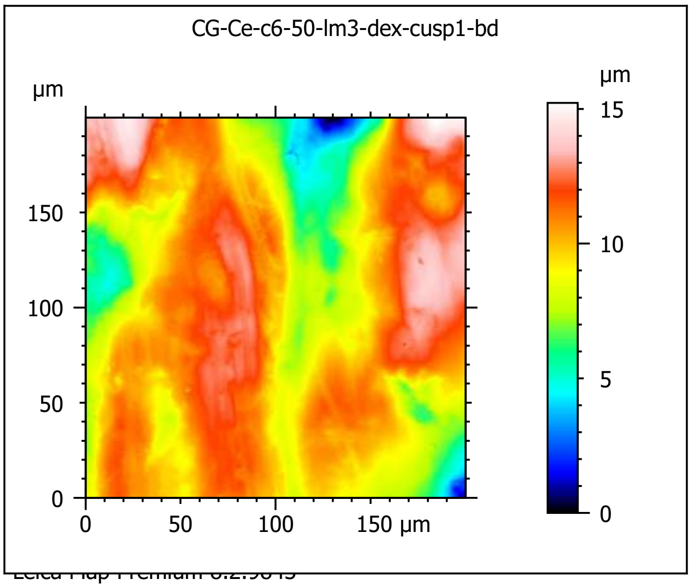

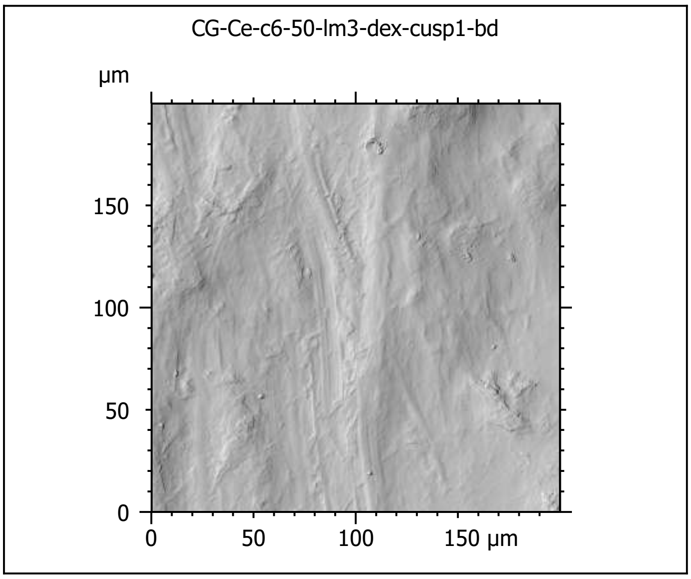

CG-Ce-c6-51-lm-sin-cusp1-bd

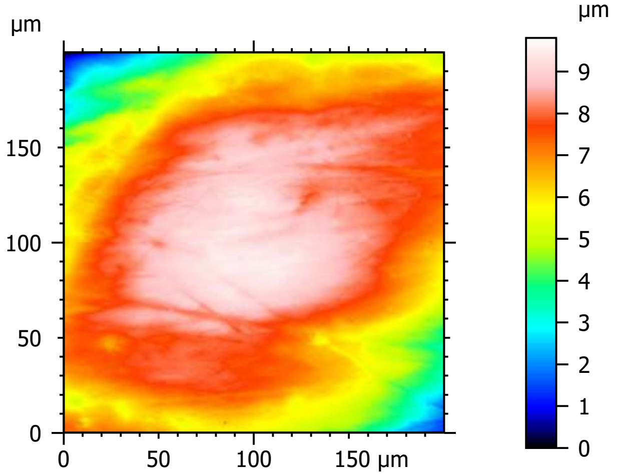

CG-Ce-c6-51-lm-sin-cusp1-bd

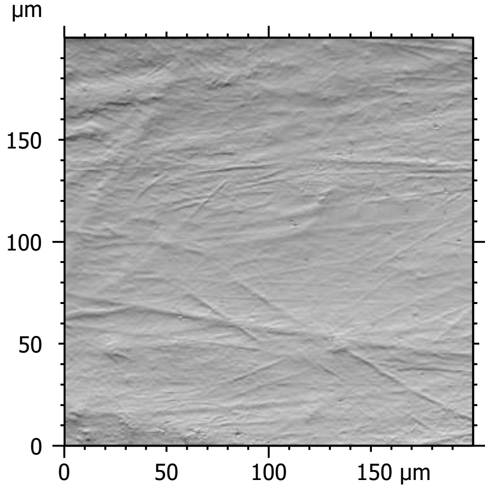

CG-Ce-c6-52-lm-dex-cusp1-bd

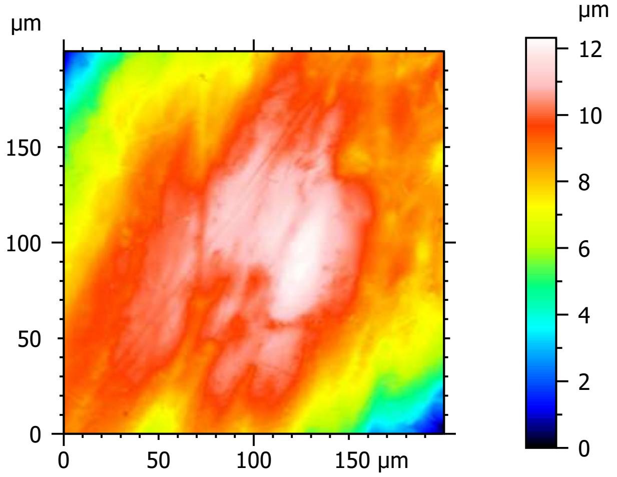

CG-Ce-c6-52-lm-dex-cusp1-bd

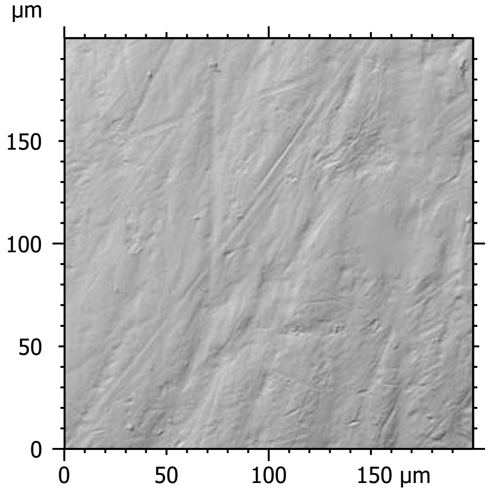

CG-Ce-c6-53-lm-dex-cusp1-bd

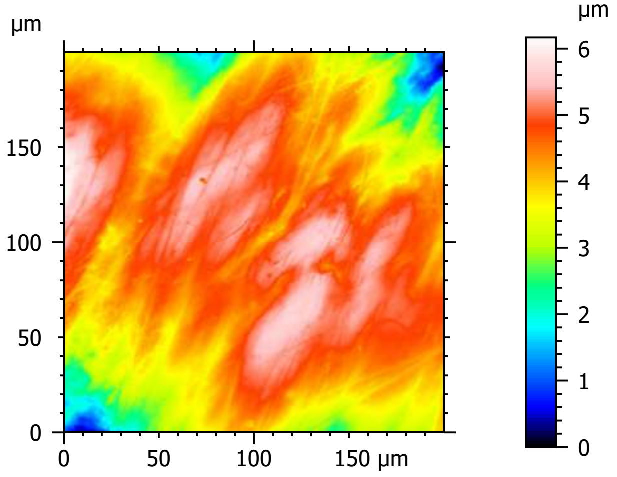

CG-Ce-c6-53-lm-dex-cusp1-bd

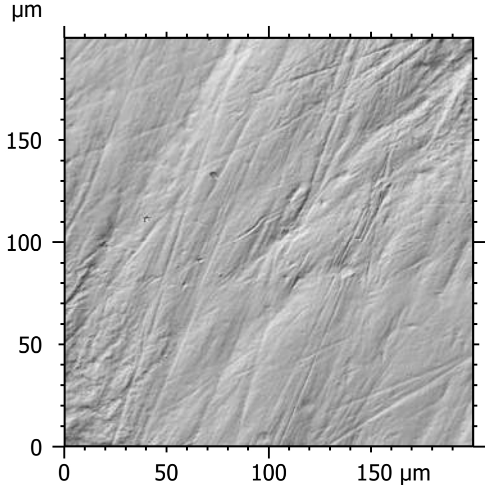

Leica Map Premium 8.2.9843

CG-Ce-c6-54-lm-dex-cusp1-bd

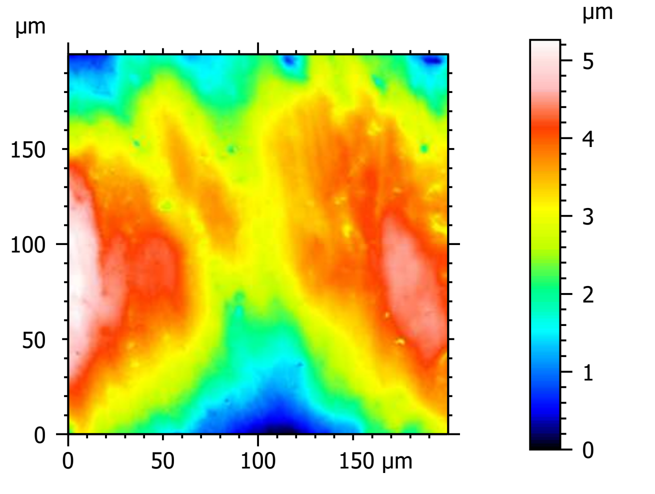

CG-Ce-c6-54-lm-dex-cusp1-bd

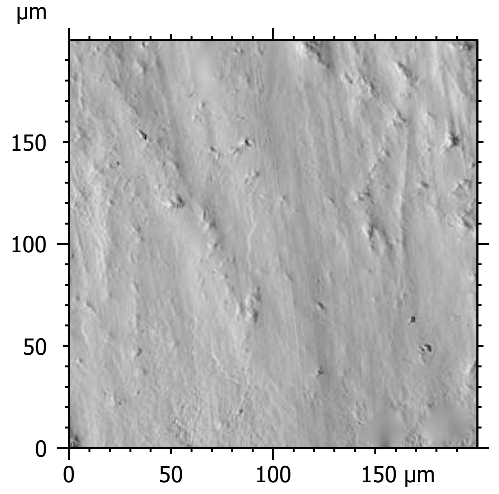

CG-Ce-c7-40-lm2-dex-cusp1-bd

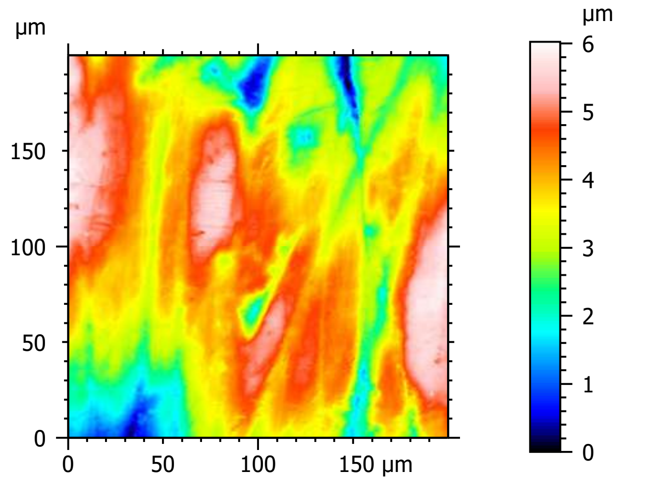

CG-Ce-c7-40-lm2-dex-cusp1-bd

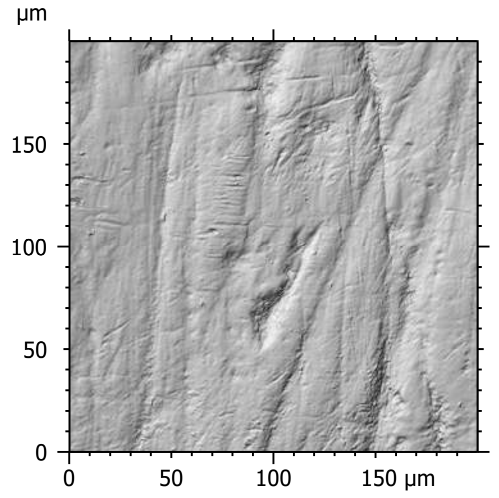

CG-Ce-c7-41-lm3-dex-cusp1-bd

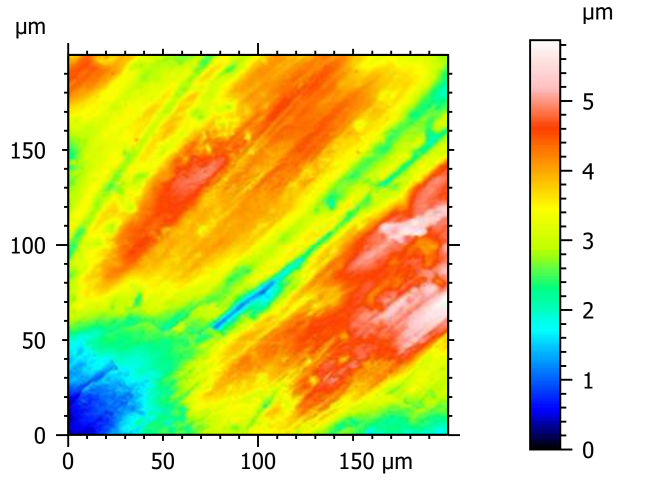

CG-Ce-c7-41-lm3-dex-cusp1-bd

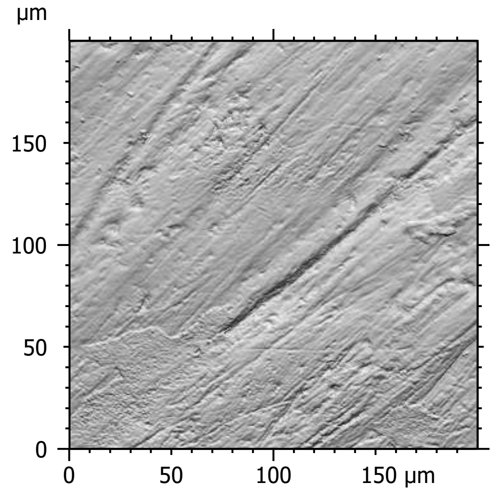

Leica Map Premium 8.2.9843

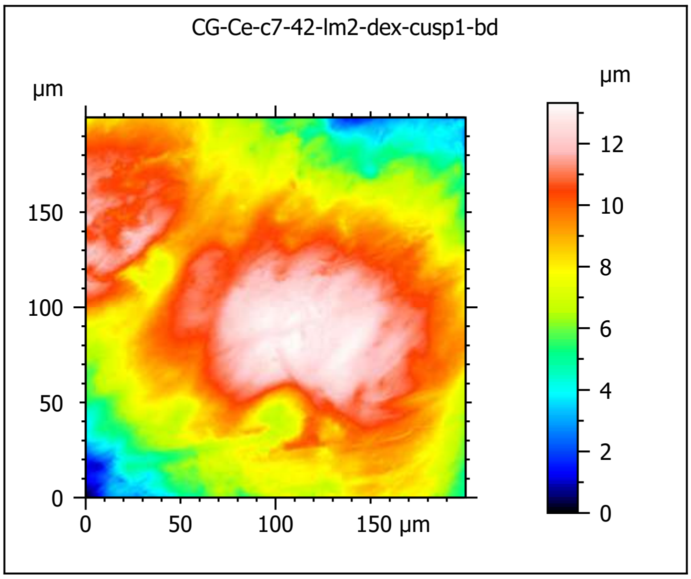

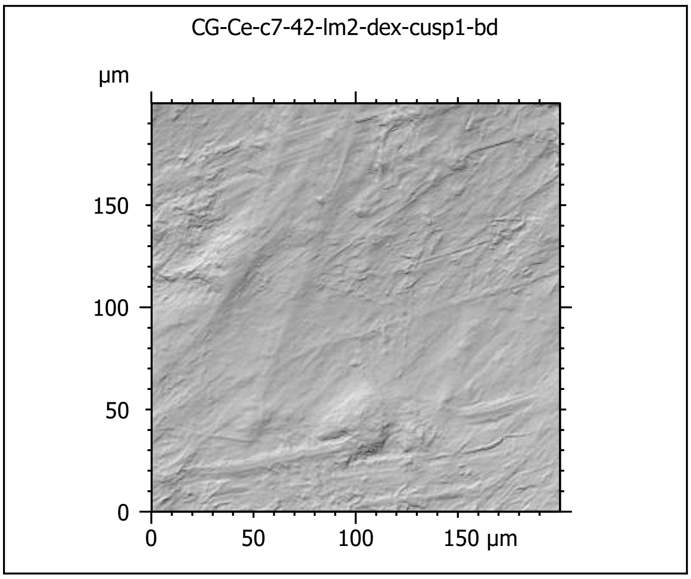

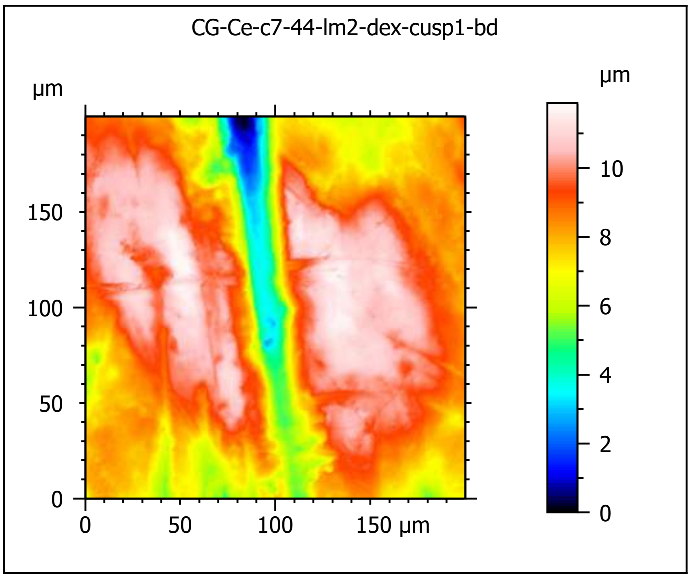

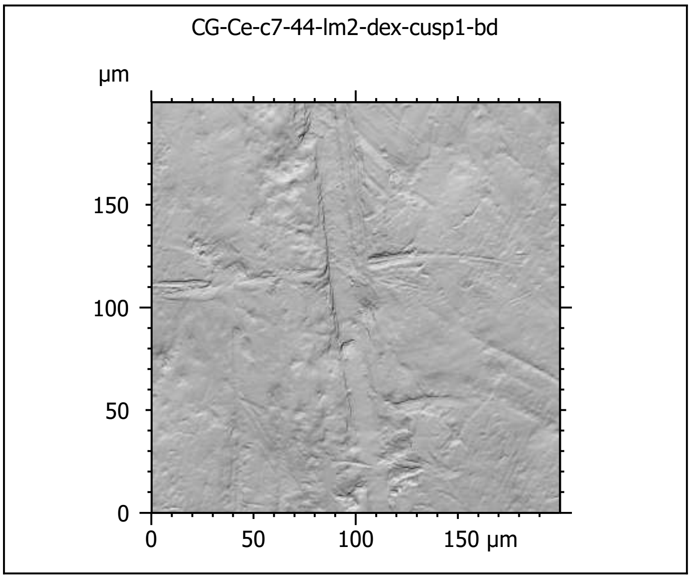

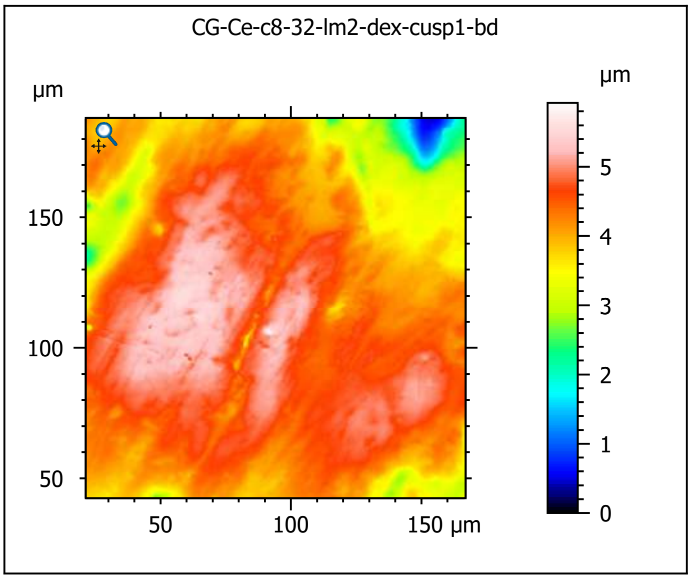

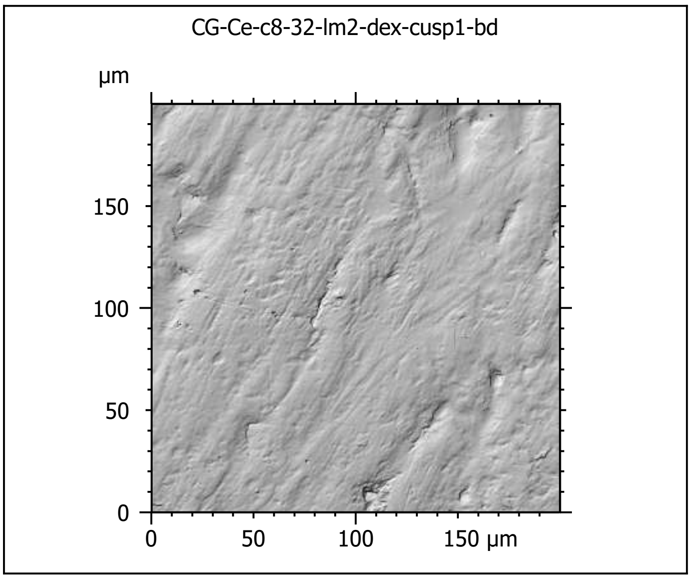

Leica Map Premium 8.2.9843

CG-Ce-c9-24-lm3-dex-cusp1-bd

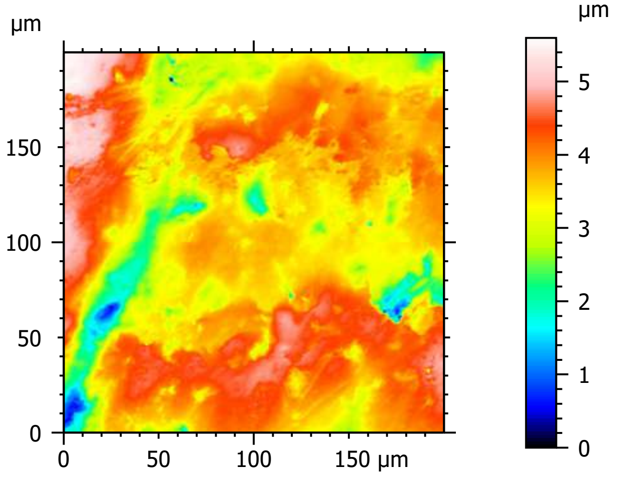

CG-Ce-c9-24-lm3-dex-cusp1-bd

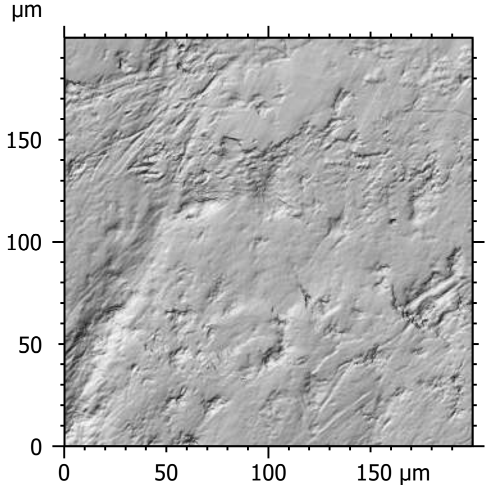

CG-Ce-c9-25-lm-dex-cusp1-bd

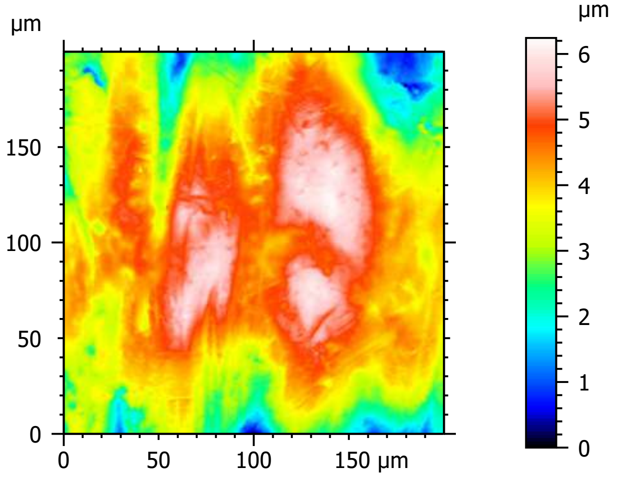

CG-Ce-c9-25-lm-dex-cusp1-bd

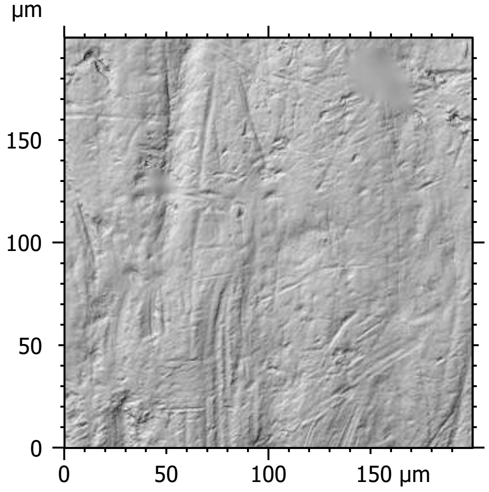

CG-Ce-c9-26-lm2-dex-cusp1-bd

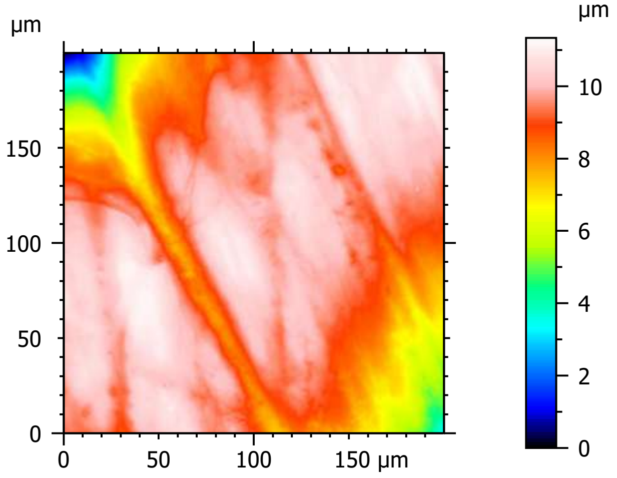

CG-Ce-c9-26-lm2-dex-cusp1-bd

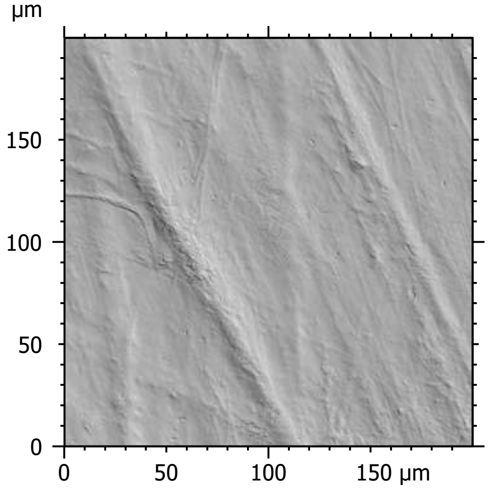

Leica Map Premium 8.2.9843

"A long-term perspective on Neandertal environment and subsistence: insights from the dental micro-texture analysis of hunted ungulates at Combe-Grenal (Dordogne, France)"

authors: Berlioz, E.; Capdepon, E.; Discamps, E.

Appendice 2:
surfaces scanned by E. Berlioz and E. Capdepon, pre-treatement by E. Berlioz and E. Capdepon, validation by E. Berlioz (2019)

***Cervus elaphus*- Bock B**

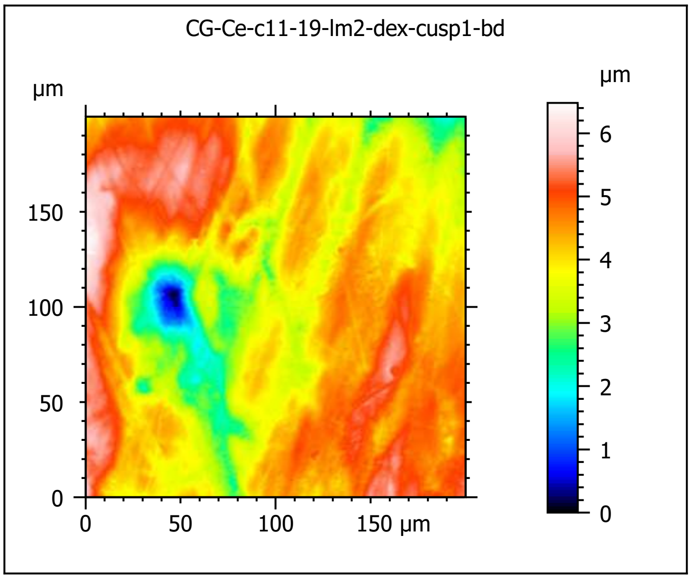

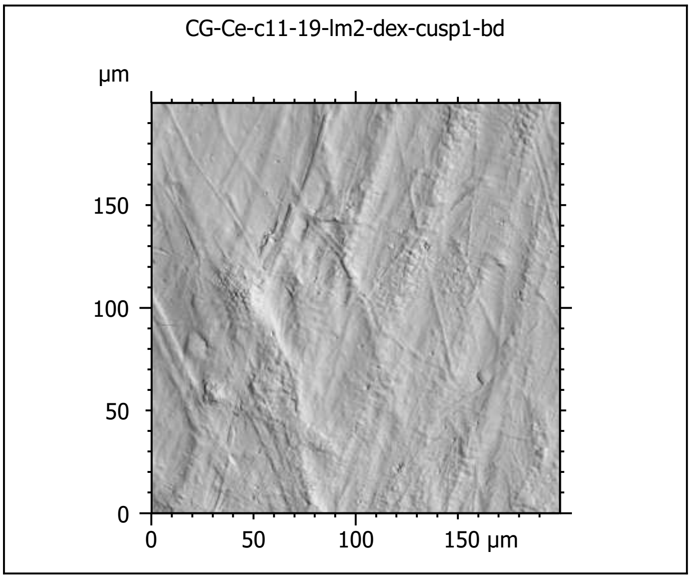

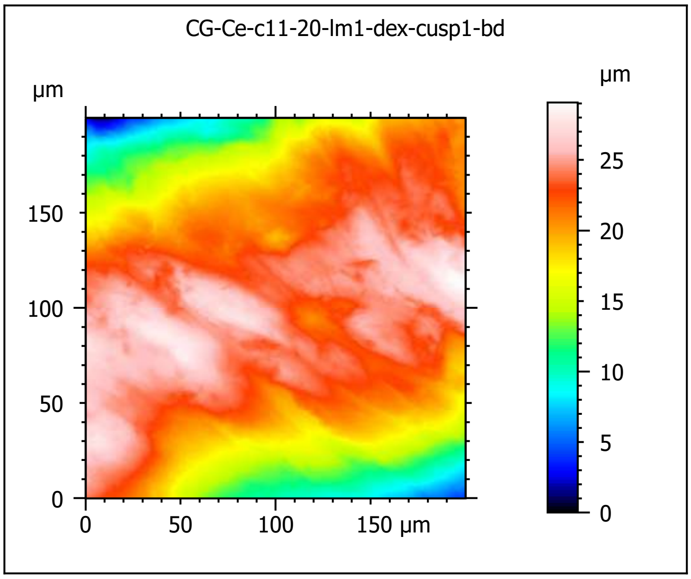

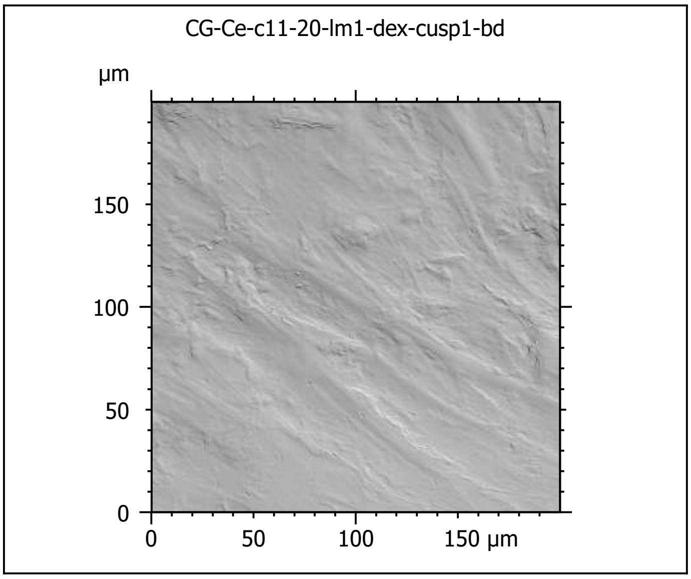

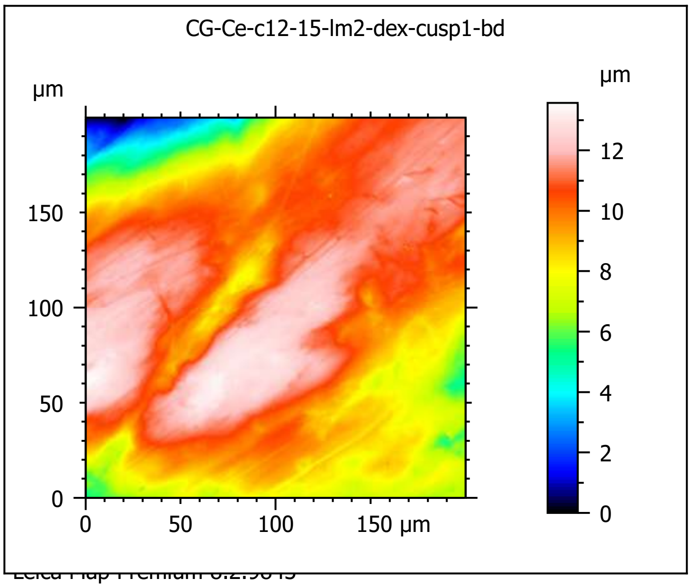

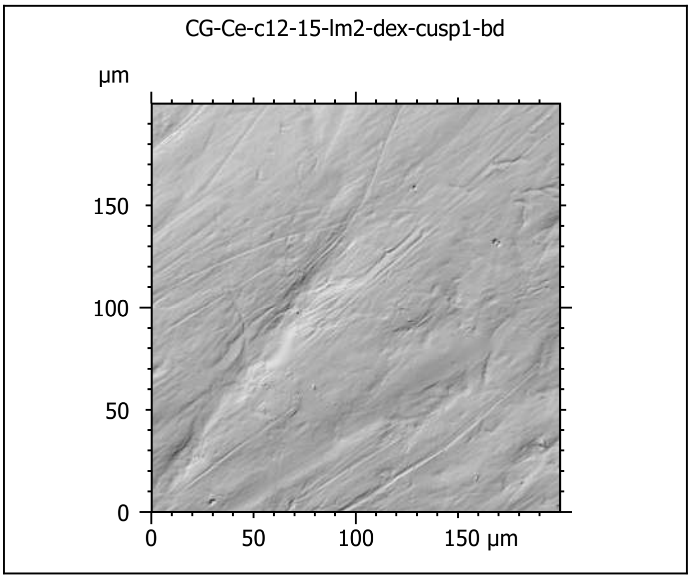

CG-Ce-c12-17-lm1-dex-cusp1-bd

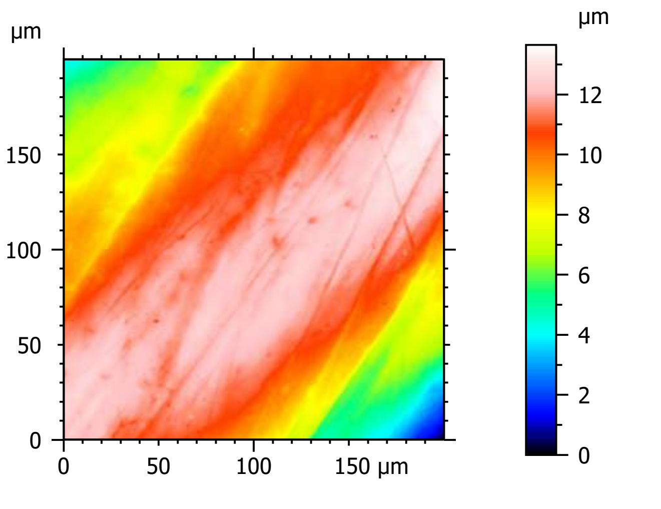

CG-Ce-c12-17-lm1-dex-cusp1-bd

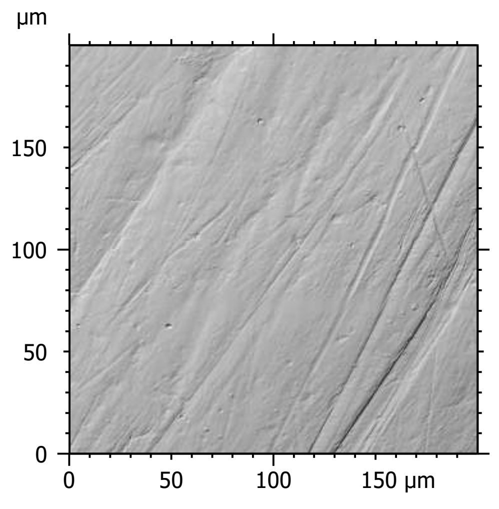

CG-Ce-c13-7-lm2-sin-cusp1-bd

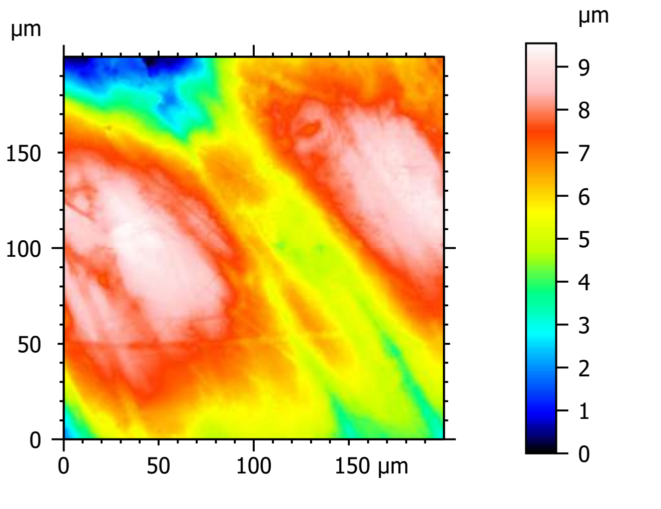

CG-Ce-c13-7-lm2-sin-cusp1-bd

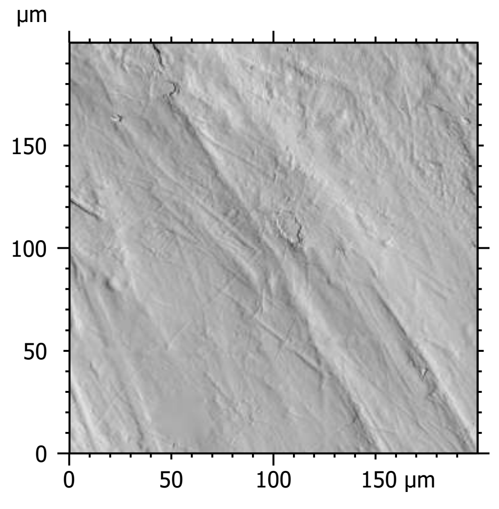

CG-Ce-c13-9-lm-sin-cusp1-bd

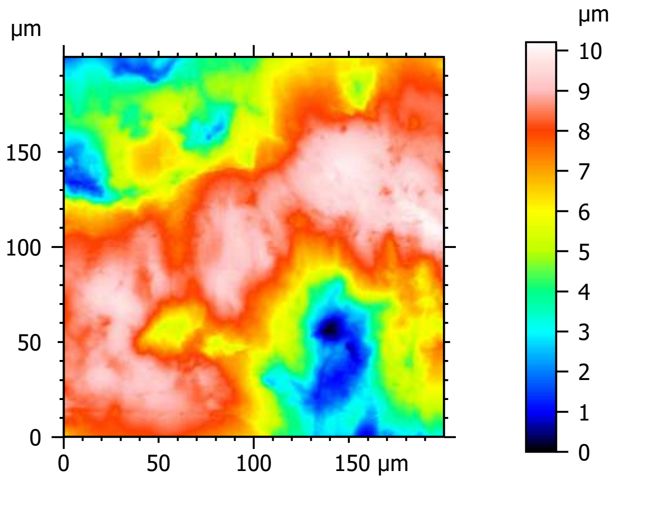

CG-Ce-c13-9-lm-sin-cusp1-bd

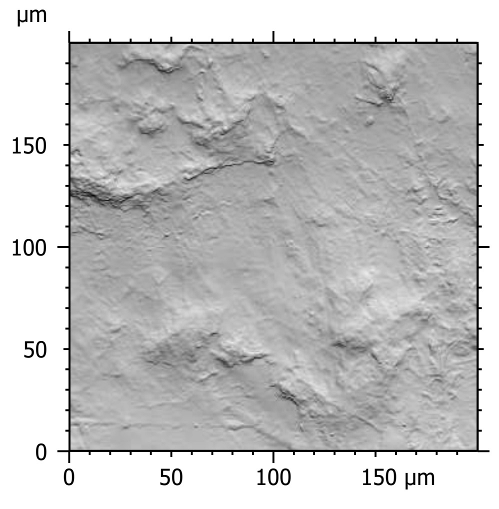

Leica Map Premium 8.2.9843

CG-Ce-c13-10-lm-sin-cusp1-bd

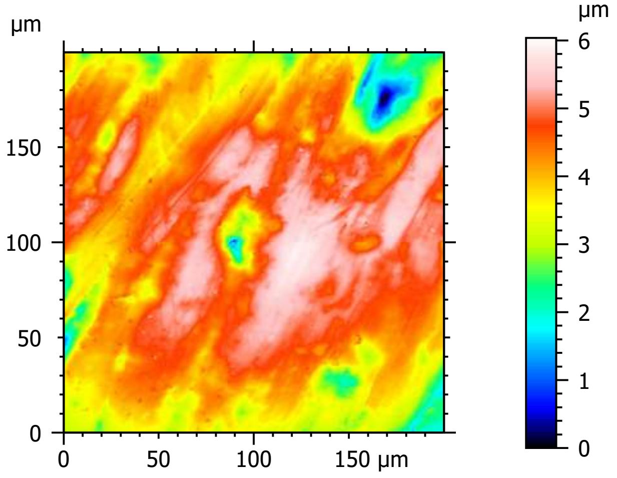

CG-Ce-c13-10-lm-sin-cusp1-bd

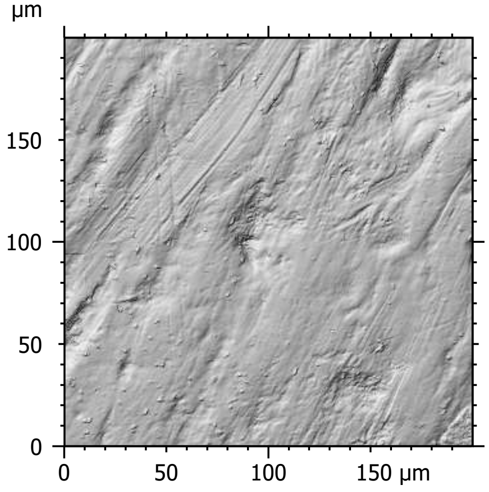

CG-Ce-c14-153-lm2-sin-cusp1-bd

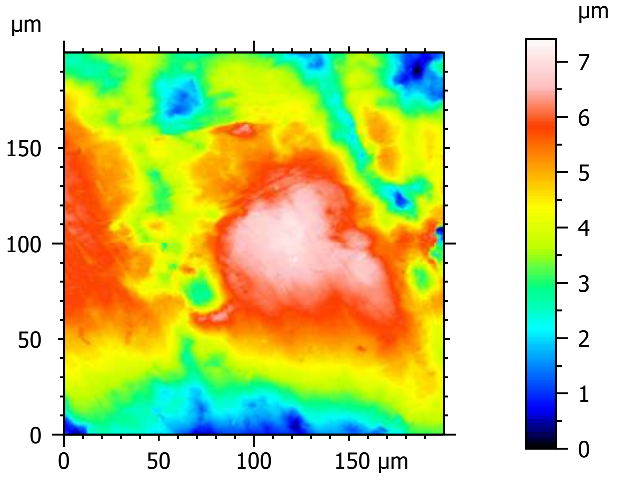

CG-Ce-c14-153-lm2-sin-cusp1-bd

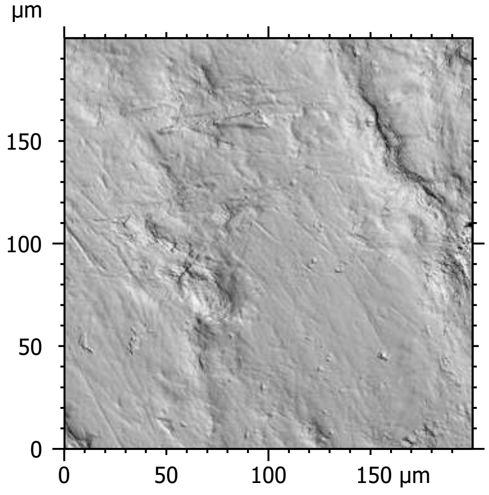

CG-Ce-c14-154-lm-sin-fgmt

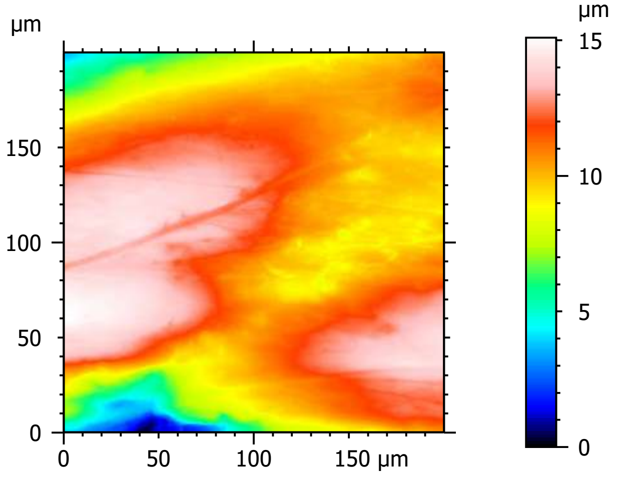

CG-Ce-c14-154-lm-sin-fgmt

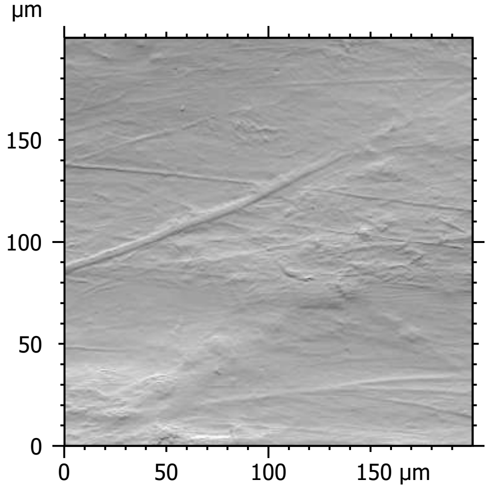

Leica Map Premium 8.2.9843

CG-Ce-c14-155-lm-sin-cusp1-bd

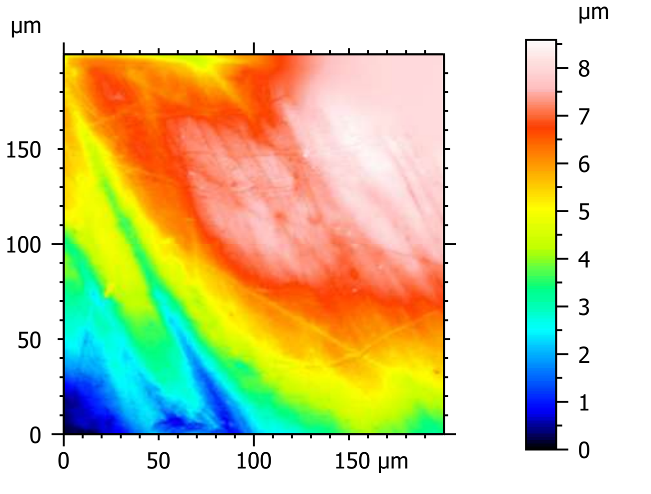

CG-Ce-c14-155-lm-sin-cusp1-bd

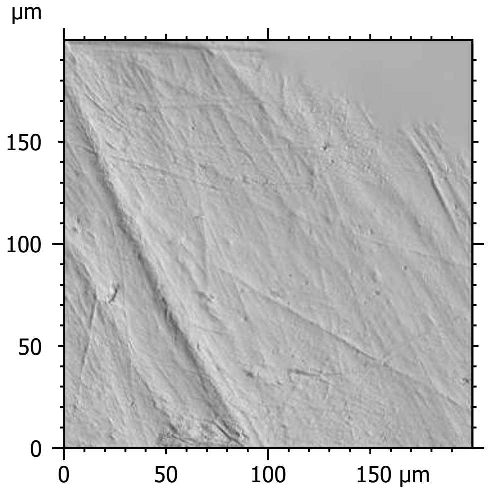

CG-Ce-c14-156-lm-dex-cusp1-bd

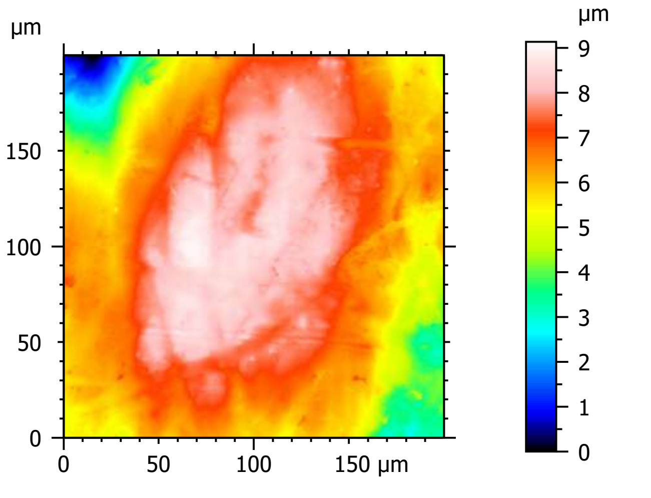

CG-Ce-c14-156-lm-dex-cusp1-bd

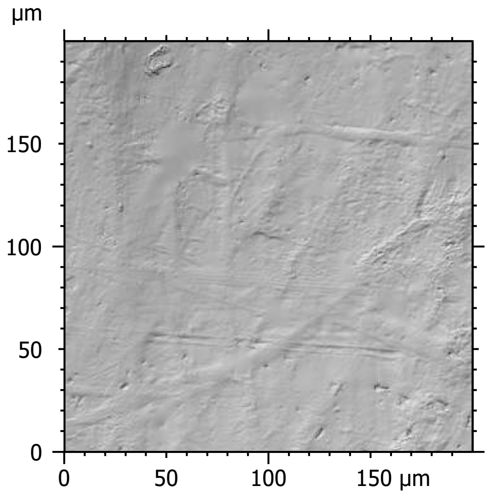

CG-Ce-c14-157-lm2-sin-cusp1-bd

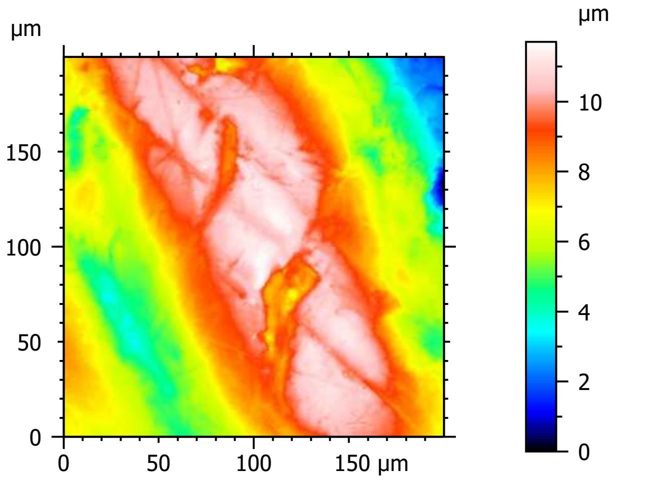

CG-Ce-c14-157-lm2-sin-cusp1-bd

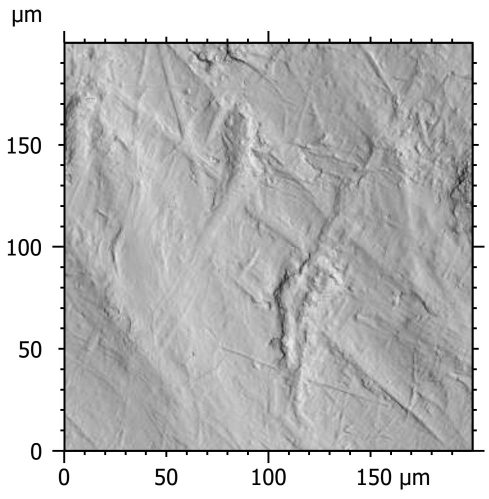

Leica Map Premium 8.2.9843

"A long-term perspective on Neandertal environment and subsistence: insights from the dental micro-texture analysis of hunted ungulates at Combe-Grenal (Dordogne, France)"

authors: Berlioz, E.; Capdepon, E.; Discamps, E.

Appendice 2:
surfaces scanned by E. Berlioz and E. Capdepon, pre-treatement by E. Berlioz and E. Capdepon, validation by E. Berlioz (2019)

***Cervus elaphus*- Block D**

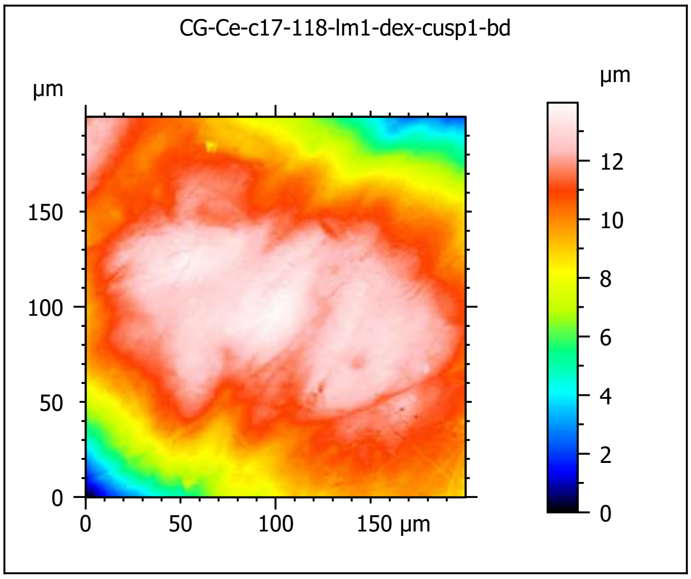

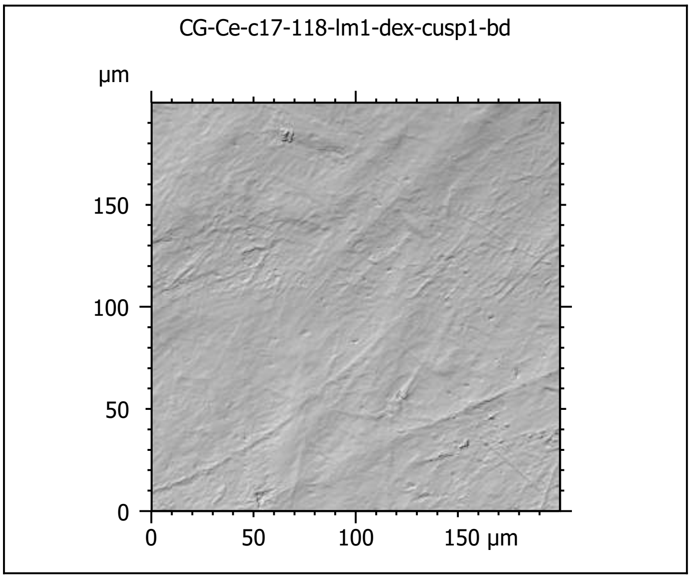

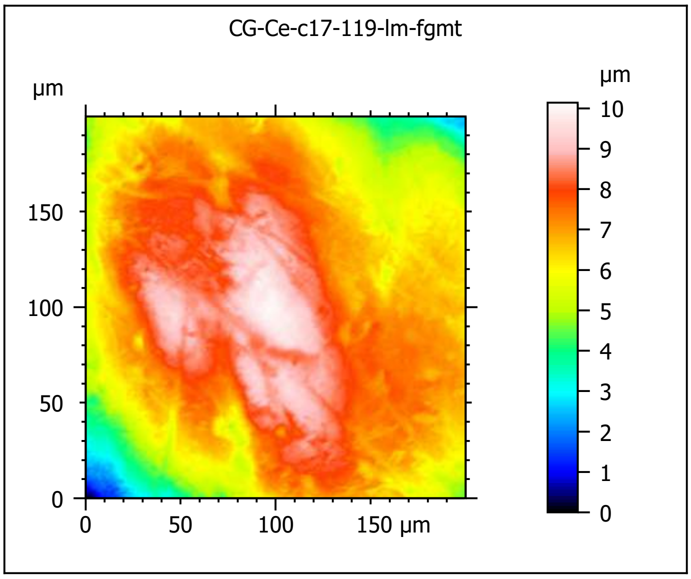

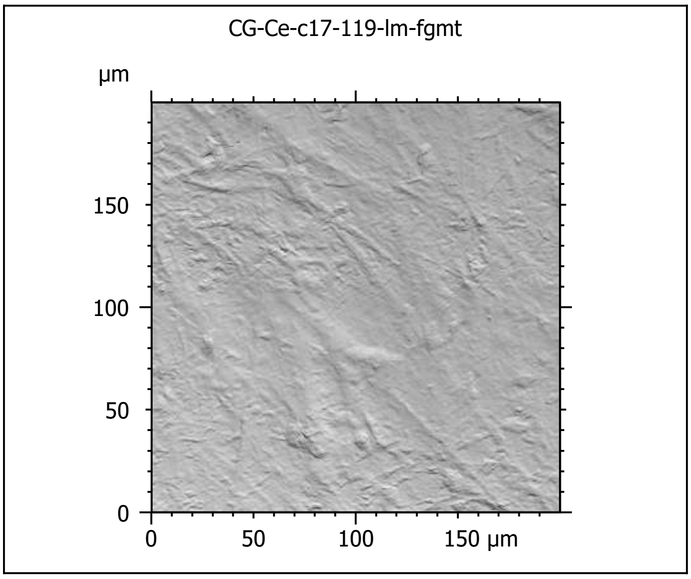

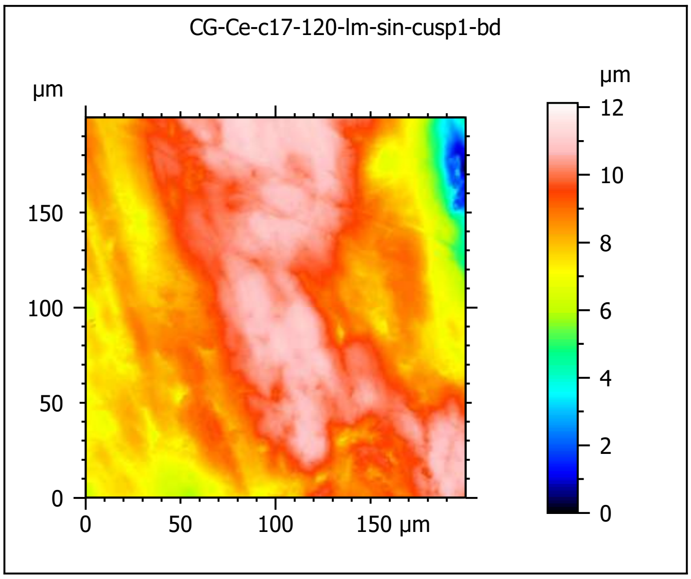

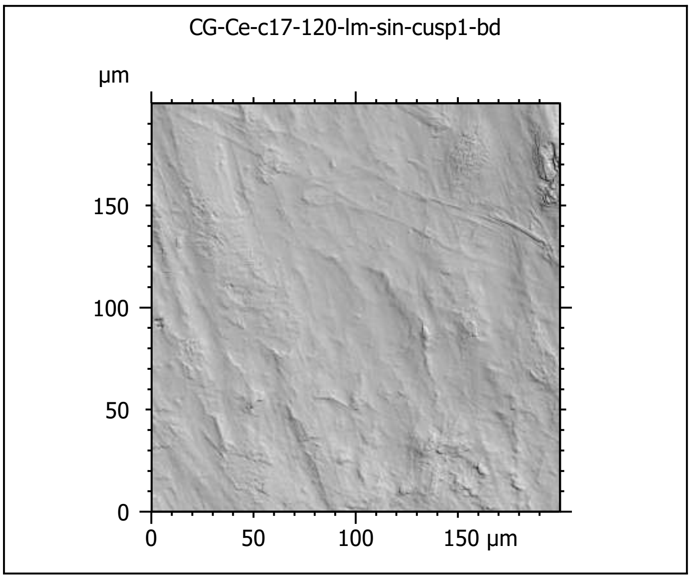

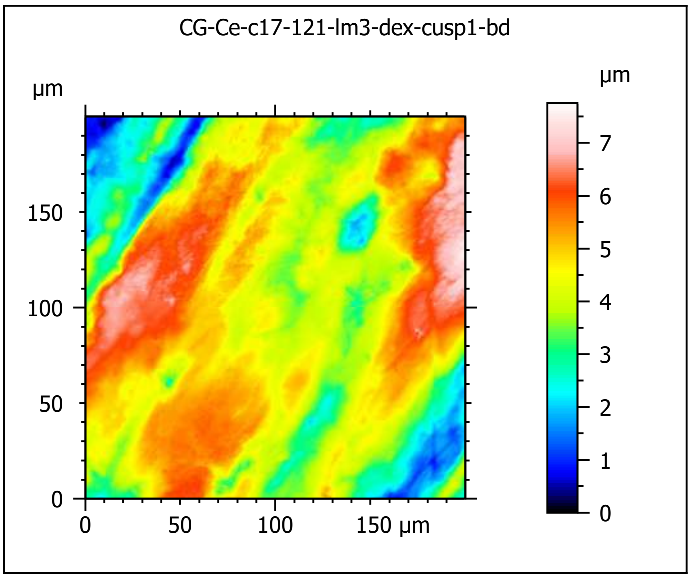

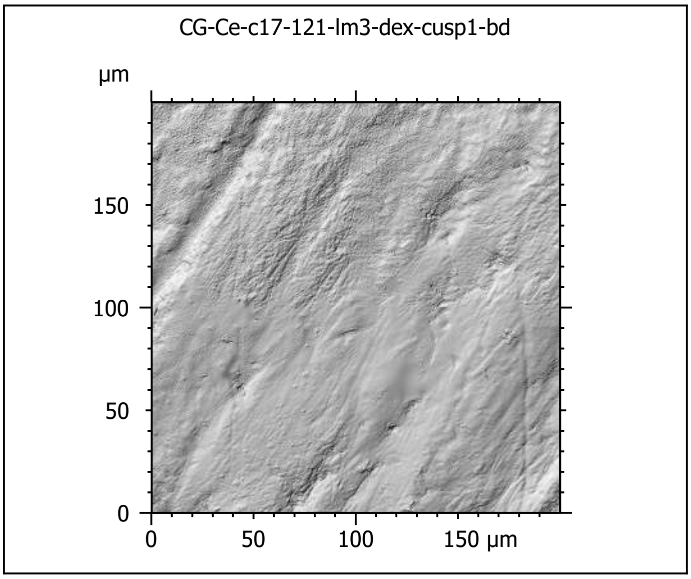

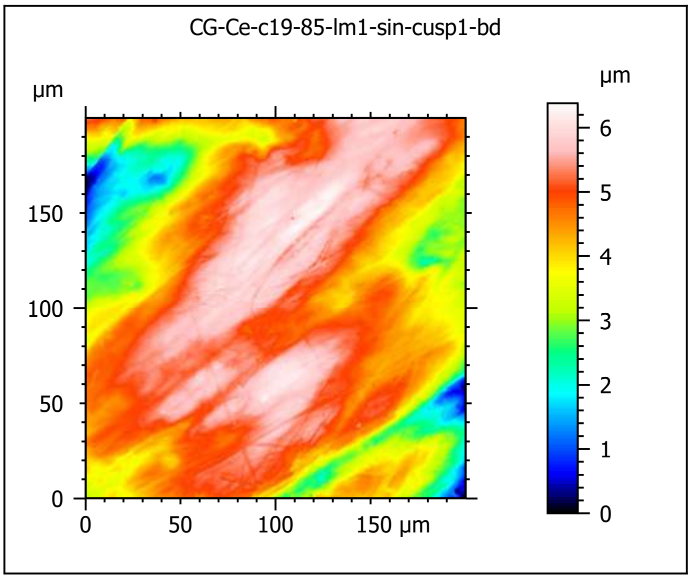

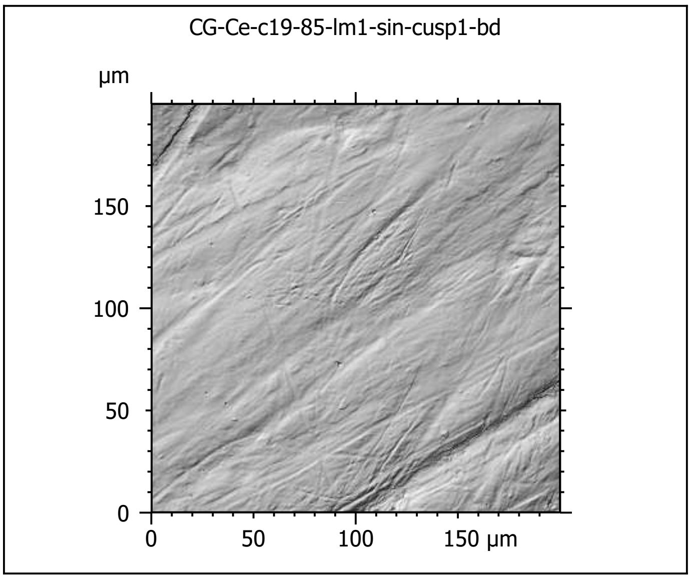

Leica Map Premium 8.2.9843

"A long-term perspective on Neandertal environment and subsistence: insights from the dental micro-texture analysis of hunted ungulates at Combe-Grenal (Dordogne, France)"

authors: Berlioz, E.; Capdepon, E.; Discamps, E.

Appendice 2:
surfaces scanned by E. Berlioz and E. Capdepon, pre-treatement by E. Berlioz and E. Capdepon, validation by E. Berlioz (2019)

***Cervus elaphus*- Block E**

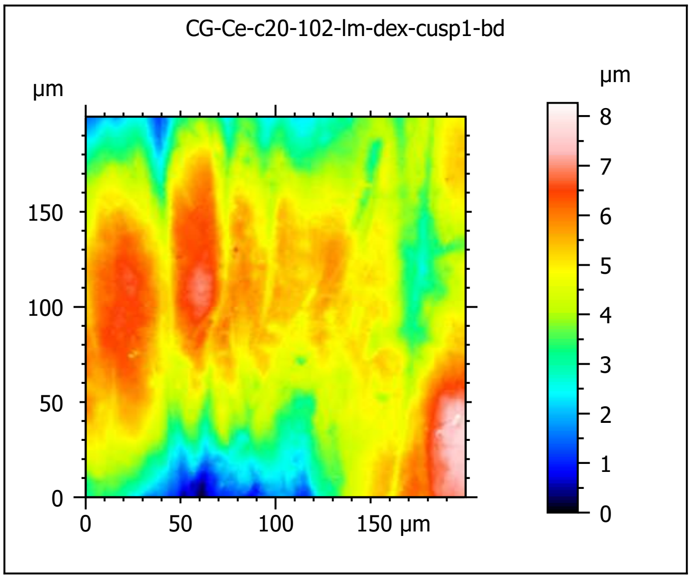

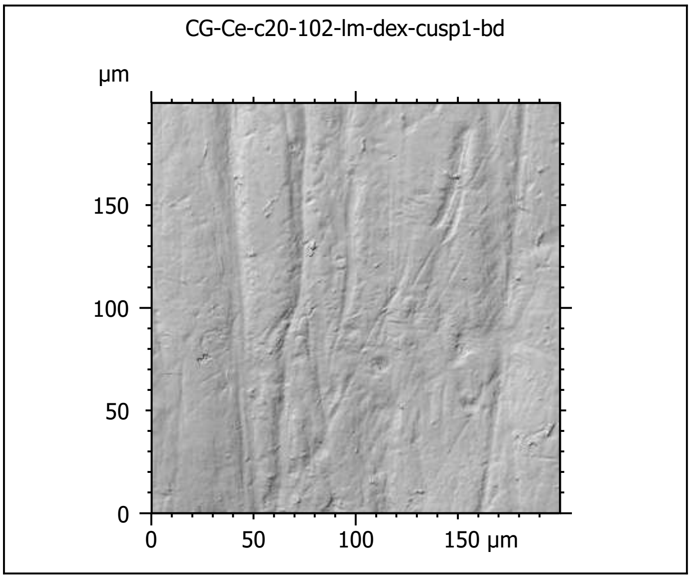

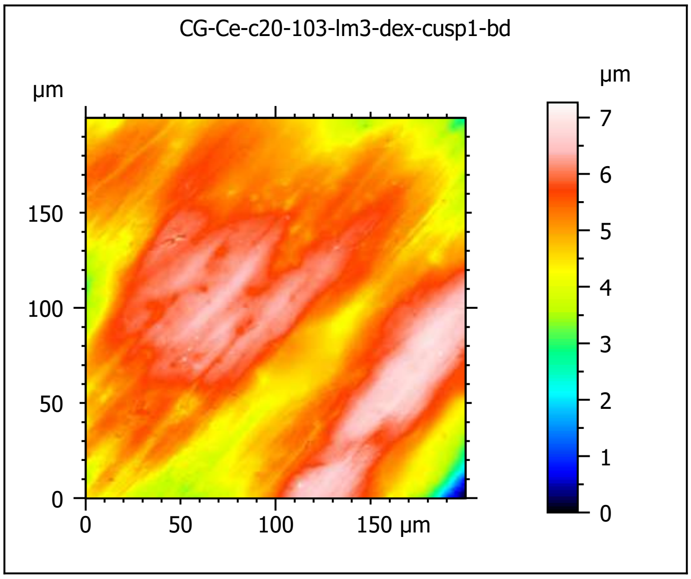

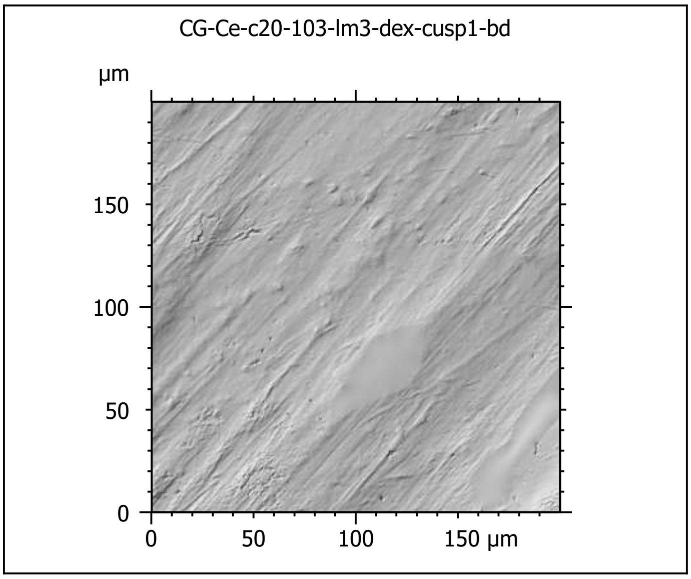

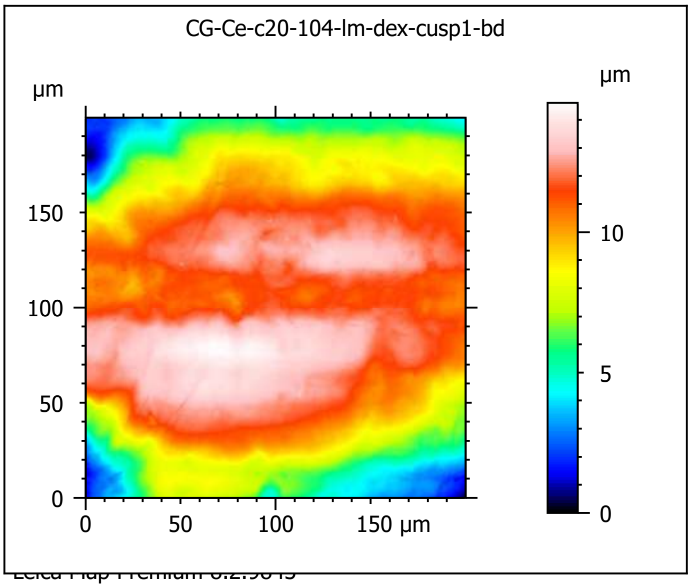

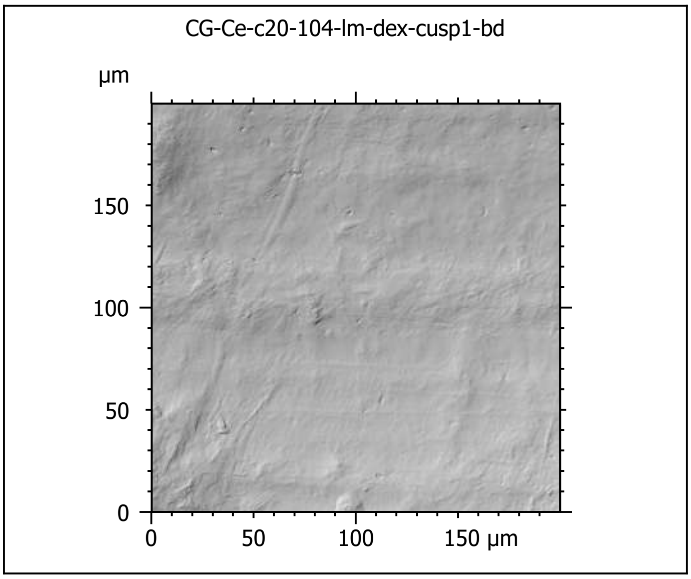

CG-Ce-c20-105-lm3-dex-cusp1-bd

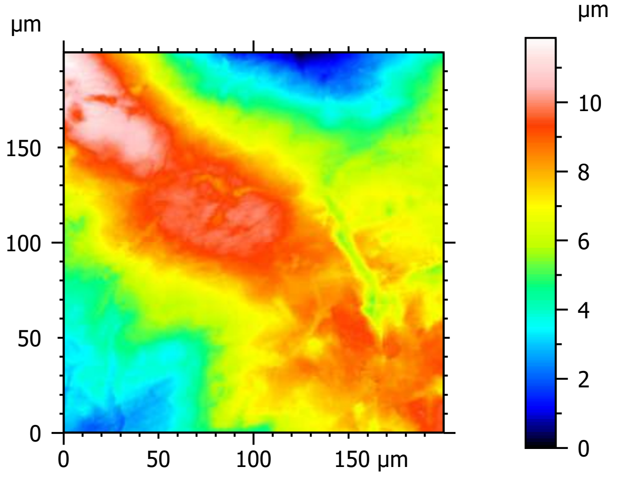

CG-Ce-c20-105-lm3-dex-cusp1-bd

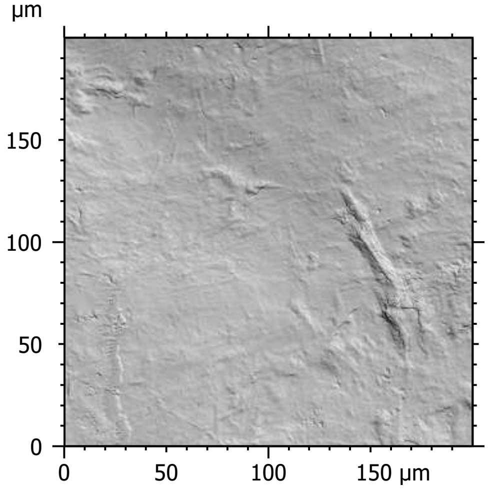

CG-Ce-c20-106-lm-sin-cusp1-bd

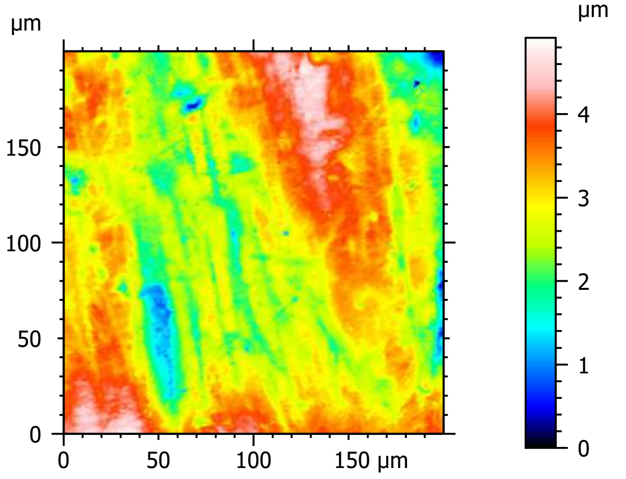

CG-Ce-c20-106-lm-sin-cusp1-bd

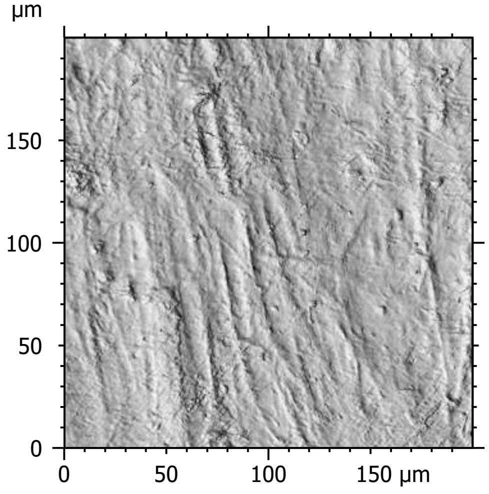

Leica Map Premium 8.2.9843

"A long-term perspective on Neandertal environment and subsistence: insights from the dental micro-texture analysis of hunted ungulates at Combe-Grenal (Dordogne, France)"

authors: Berlioz, E.; Capdepon, E.; Discamps, E.

Appendice 2:
surfaces scanned by E. Berlioz and E. Capdepon, pre-treatement by E. Berlioz and E. Capdepon, validation by E. Berlioz (2019)

***Cervus elaphus*- Block F**

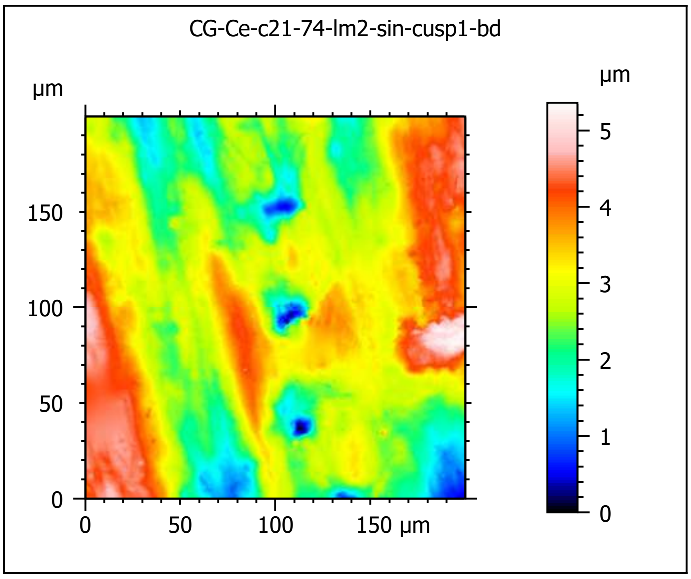

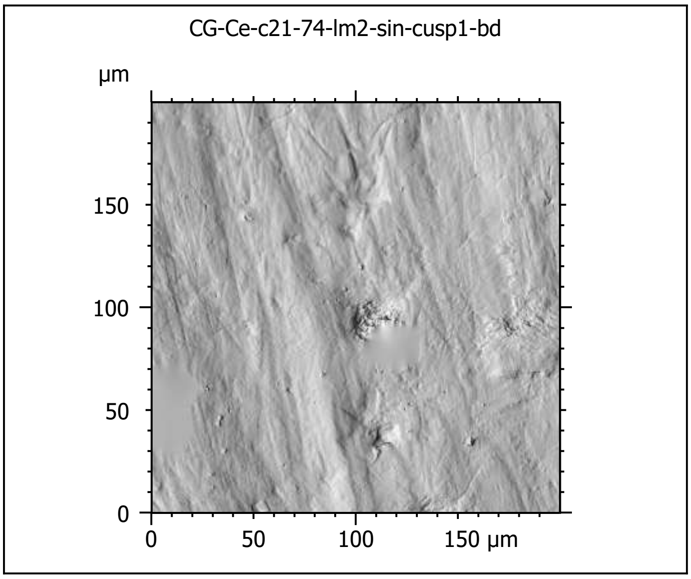

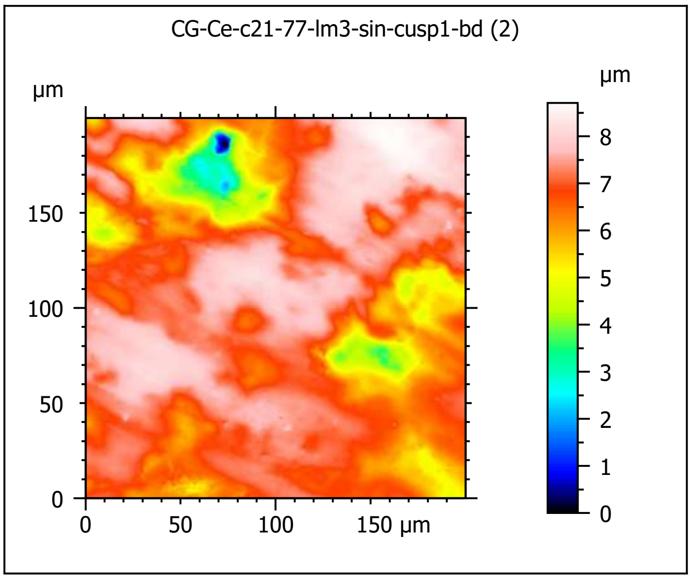

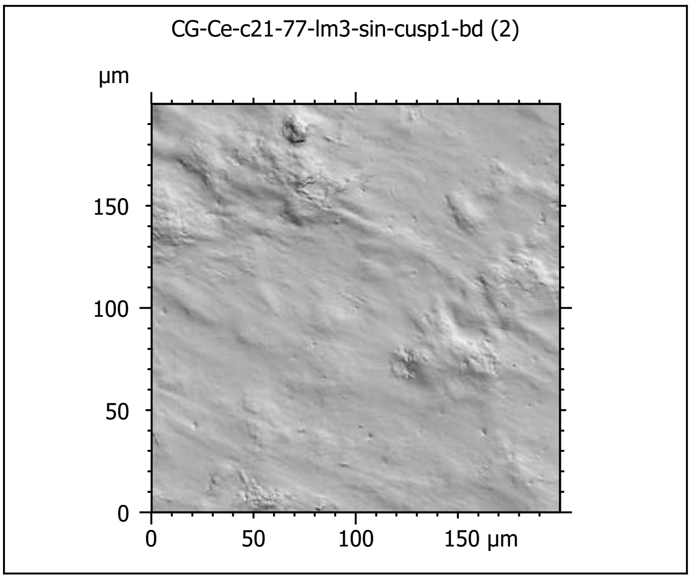

Leica Map Premium 8.2.9843

CG-Ce-c21-78-lm-dex-cusp1-bd

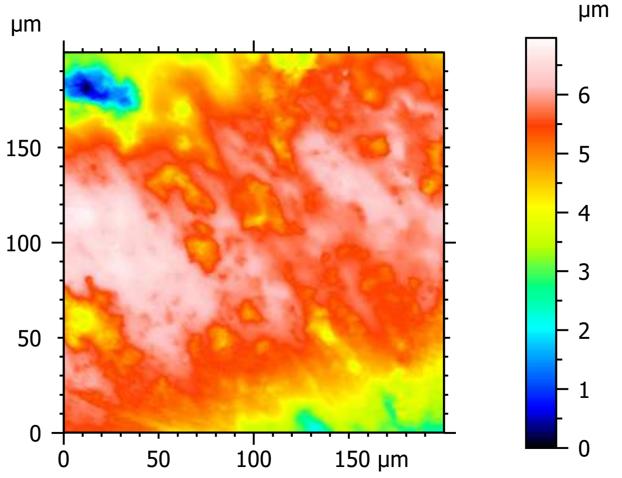

CG-Ce-c21-78-lm-dex-cusp1-bd

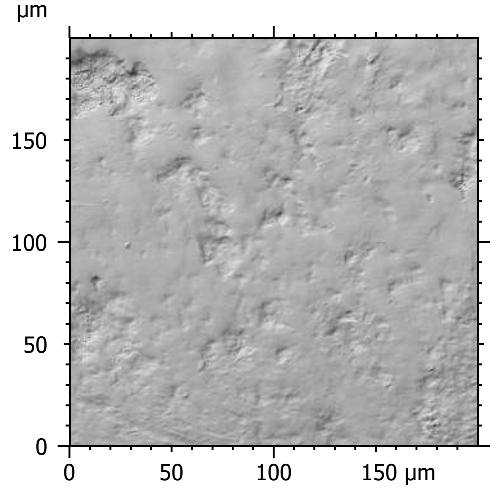

CG-Ce-c21-79-lm-sin-cusp1-bd

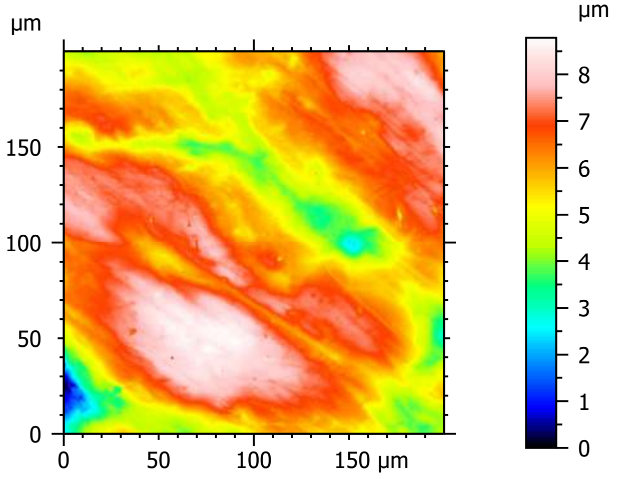

CG-Ce-c21-79-lm-sin-cusp1-bd

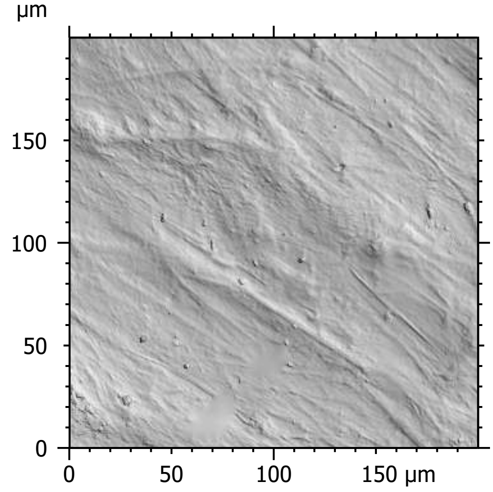

CG-Ce-c21-80-lm-dex-cusp1-bd

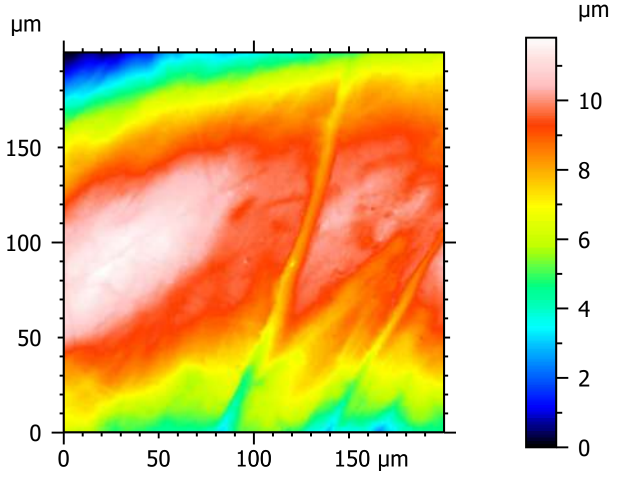

CG-Ce-c21-80-lm-dex-cusp1-bd

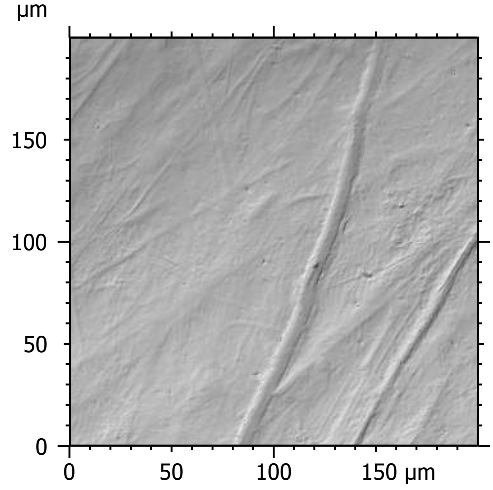

Leica Map Premium 8.2.9843

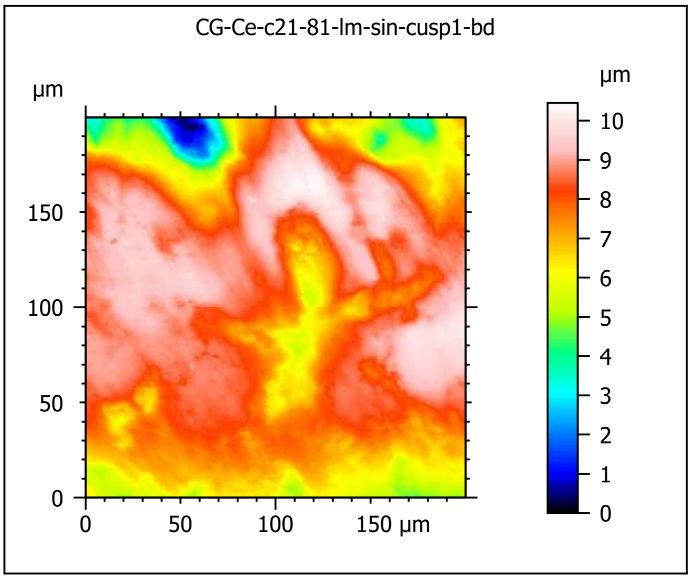

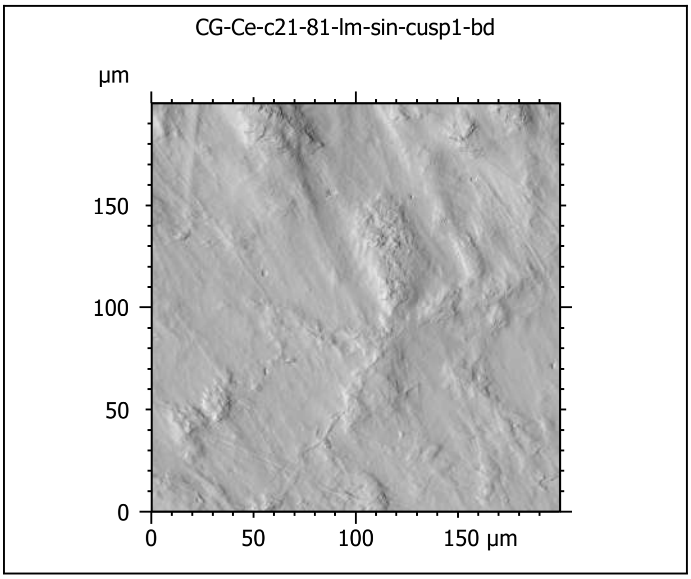

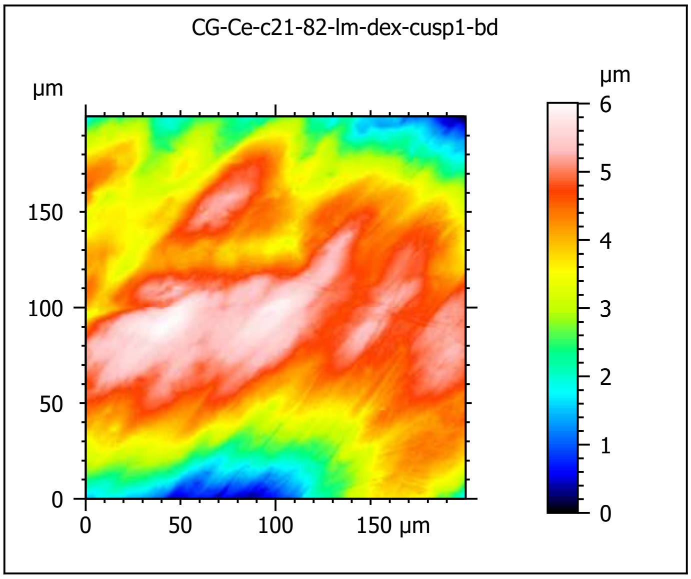

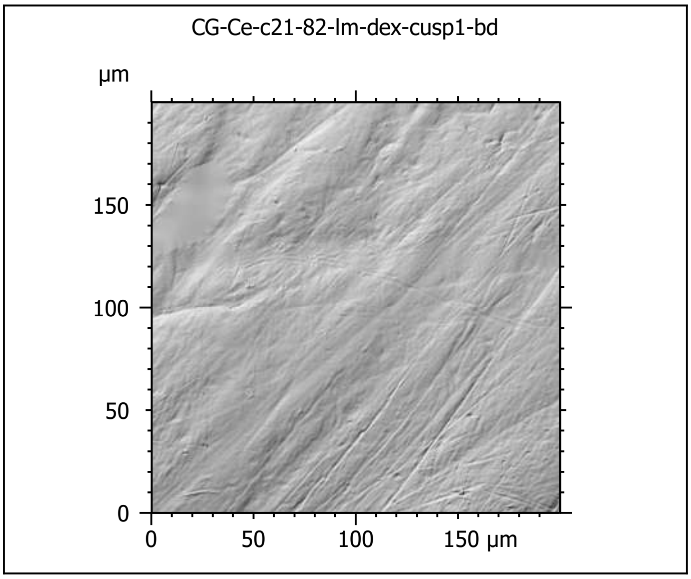

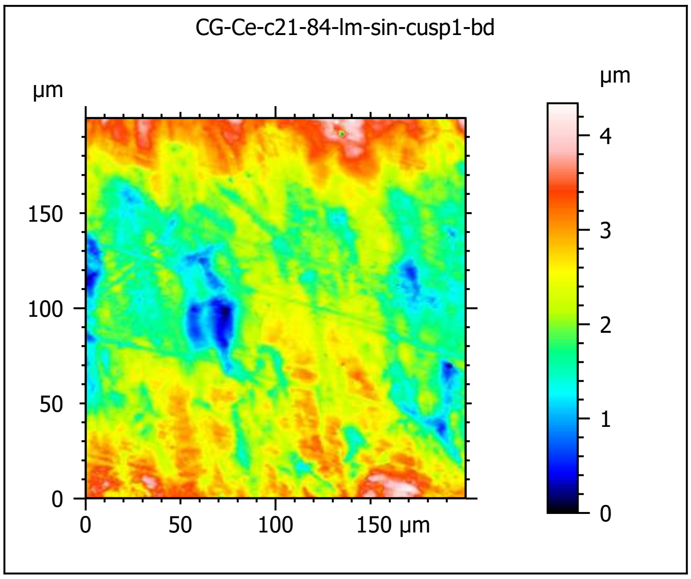

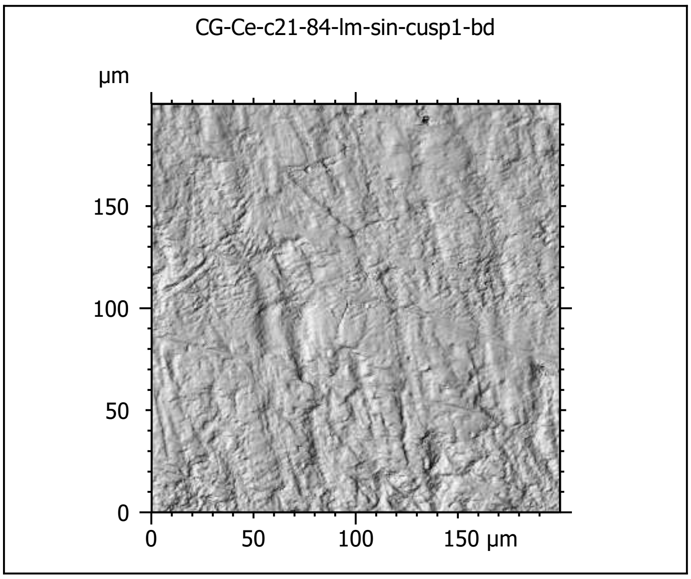

Leica Map Premium 8.2.9843

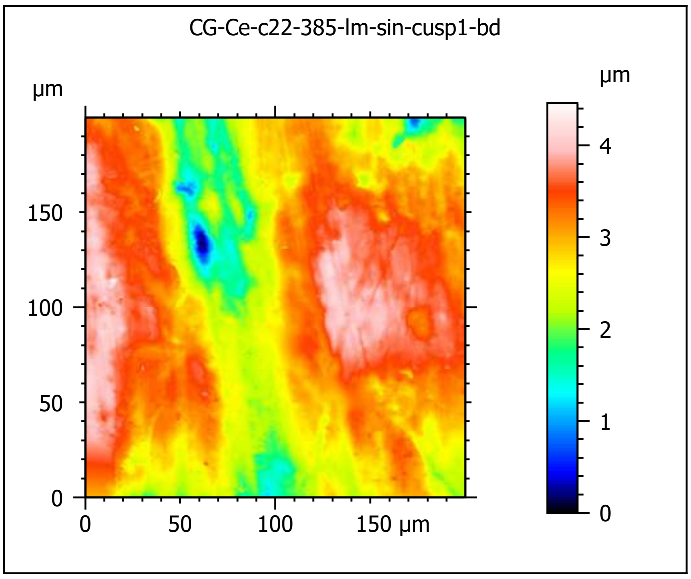

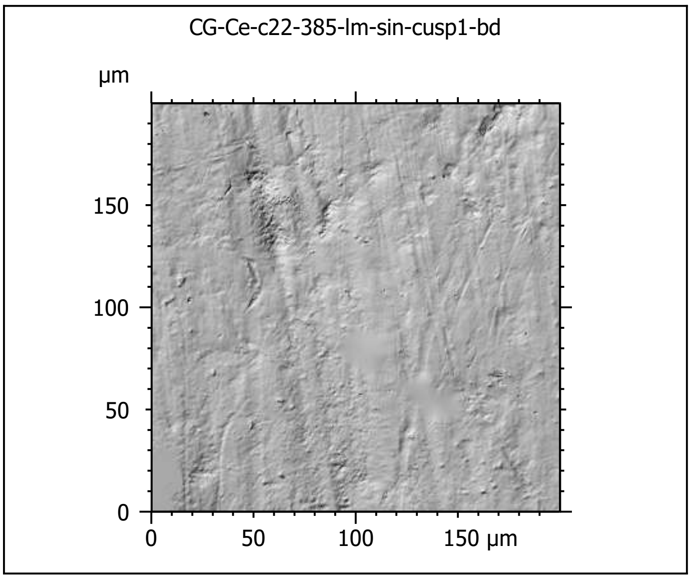

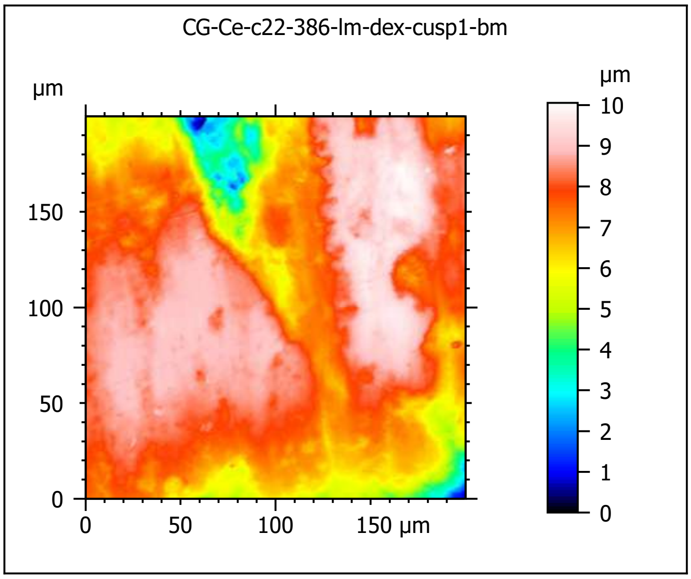

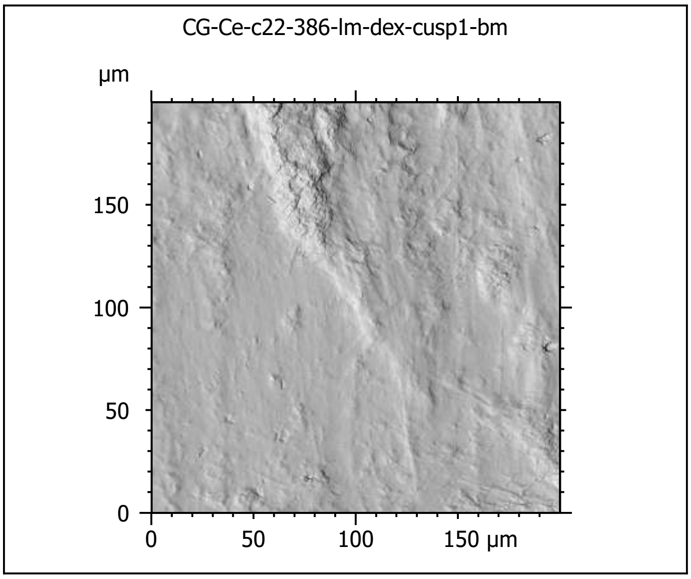

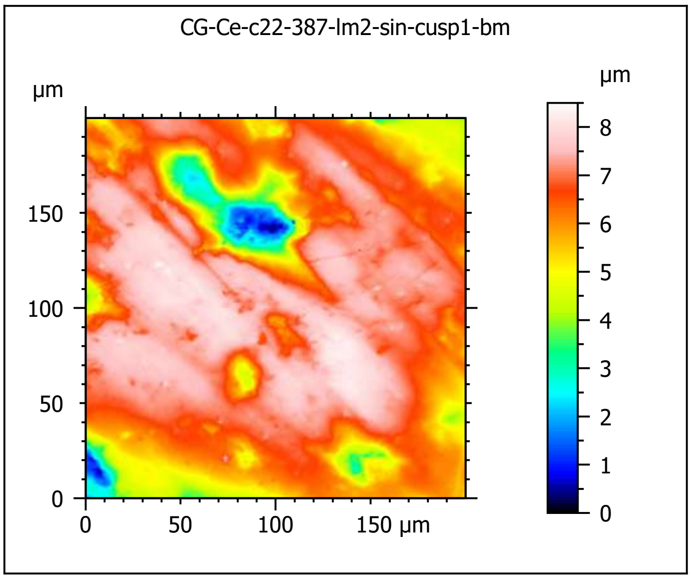

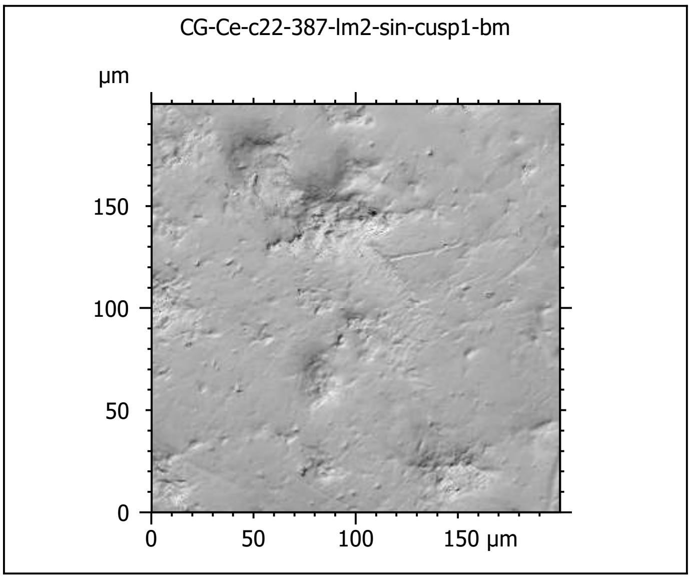

Leica Map Premium 8.2.9843

CG-Ce-c22-388-lm2-sin-cusp1-bd

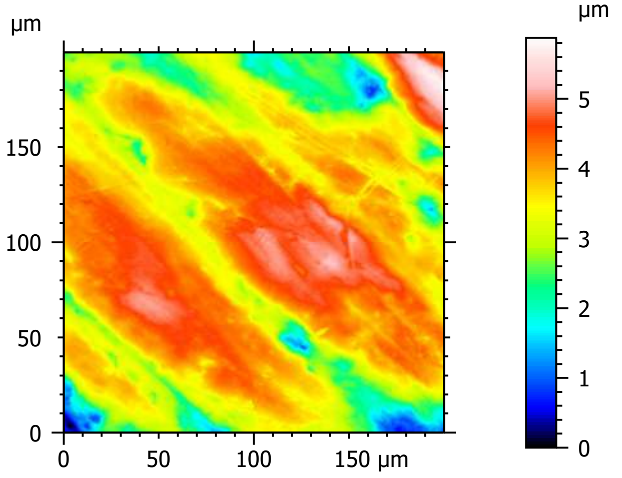

CG-Ce-c22-388-lm2-sin-cusp1-bd

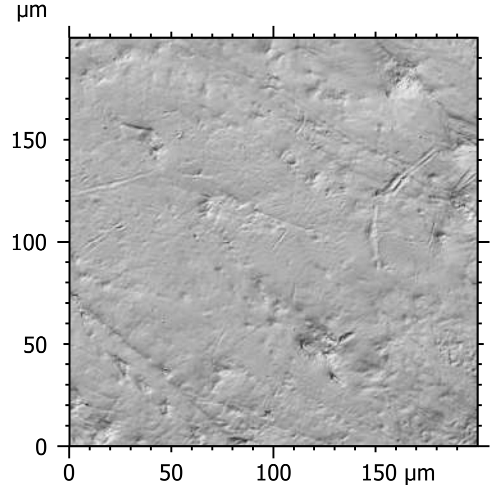

CG-Ce-c22-389-lm1-sin-cusp1-bm

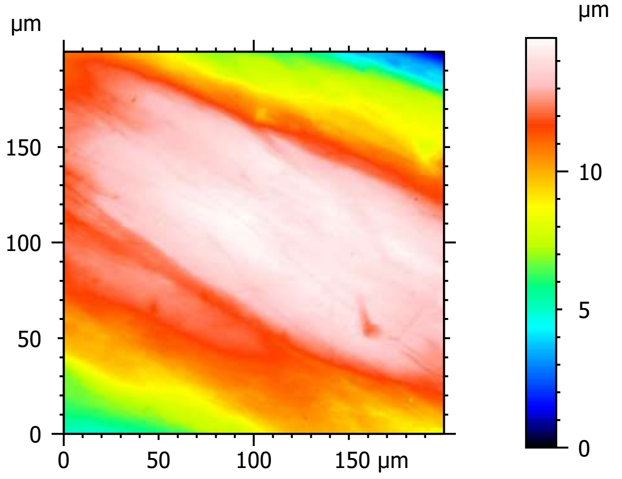

CG-Ce-c22-389-lm1-sin-cusp1-bm

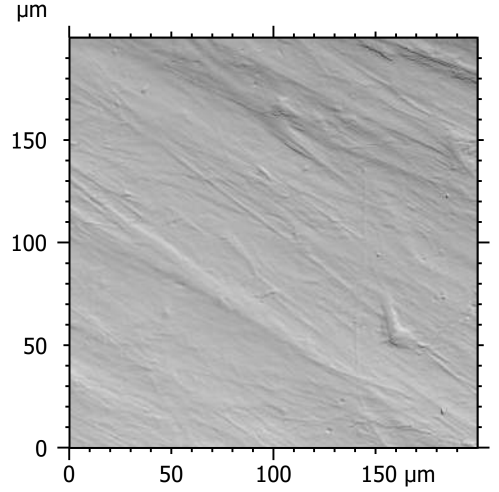

CG-Ce-c22-1025-lm2-dex-cusp1-bd

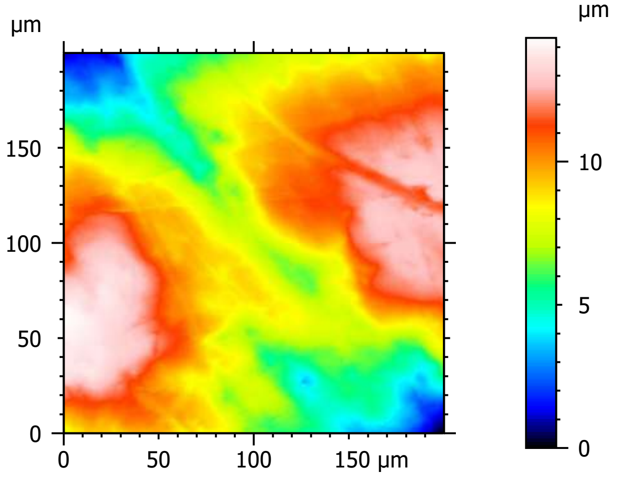

CG-Ce-c22-1025-lm2-dex-cusp1-bd

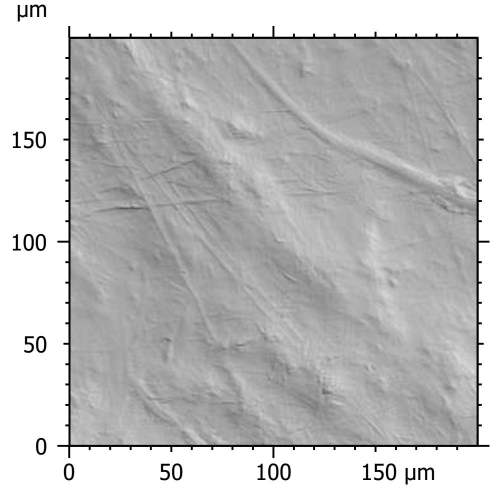

Leica Map Premium 8.2.9843

CG-Ce-c23-374-lm2-sin-cusp1-bd

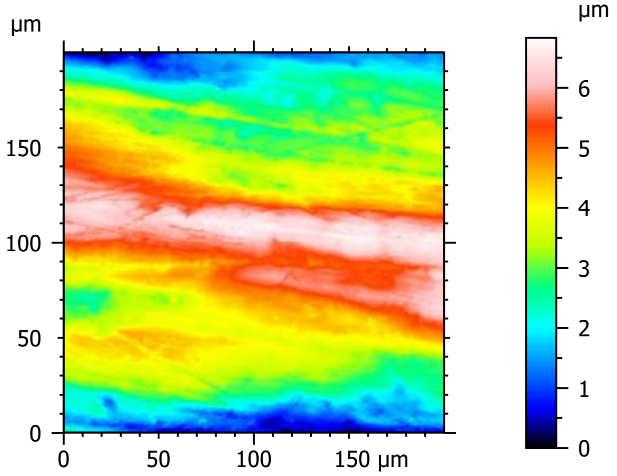

CG-Ce-c23-374-lm2-sin-cusp1-bd

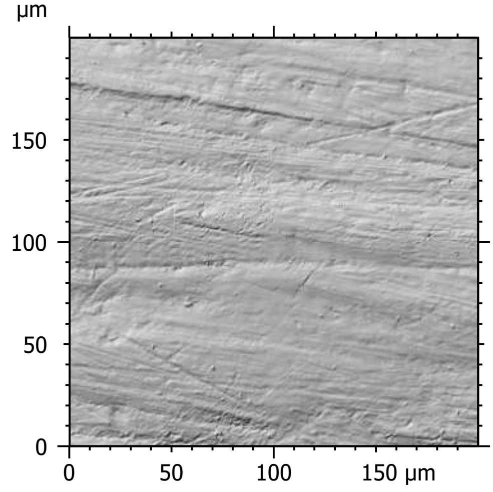

CG-Ce-c23-376-lm-sin-cusp1-bd

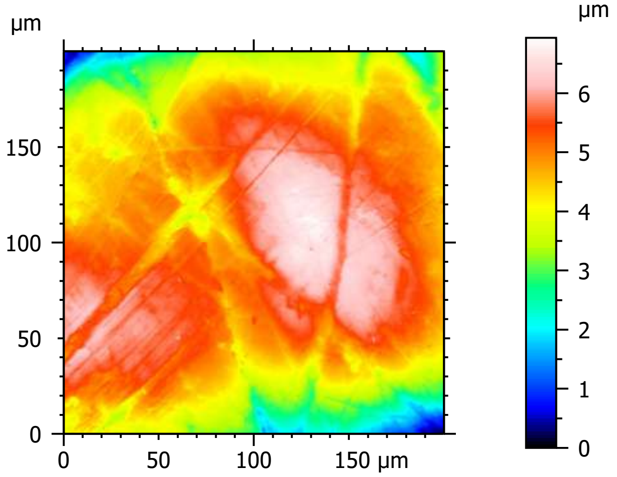

CG-Ce-c23-376-lm-sin-cusp1-bd

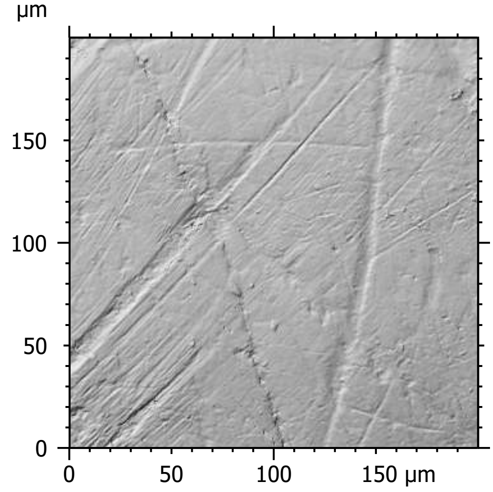

CG-Ce-c23-377-lm-sin-cusp1-bd

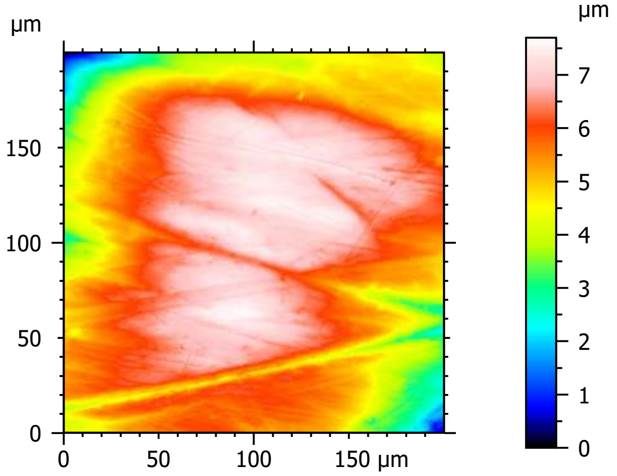

CG-Ce-c23-377-lm-sin-cusp1-bd

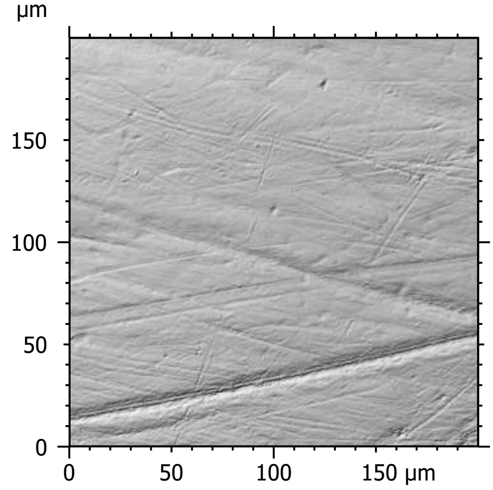

Leica Map Premium 8.2.9843

CG-Ce-c23-378-lm-sin-cusp1-bd

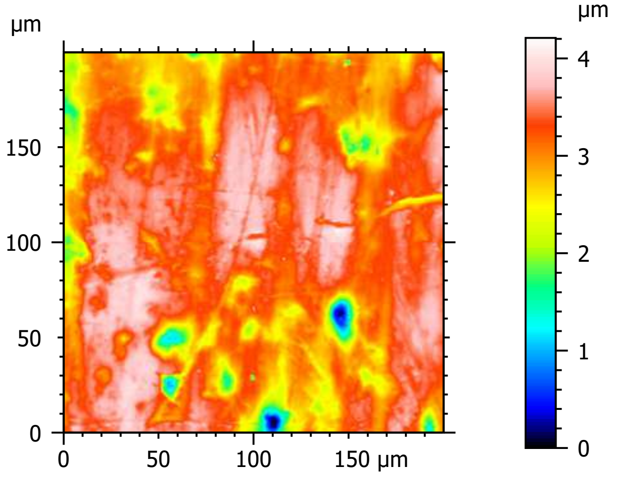

CG-Ce-c23-378-lm-sin-cusp1-bd

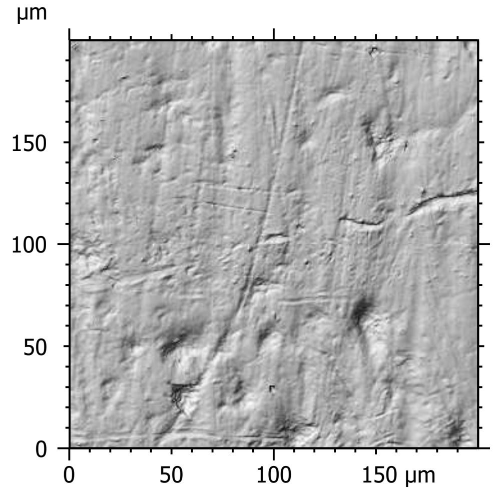

CG-Ce-c23-380-lm-dex-cusp1-bd

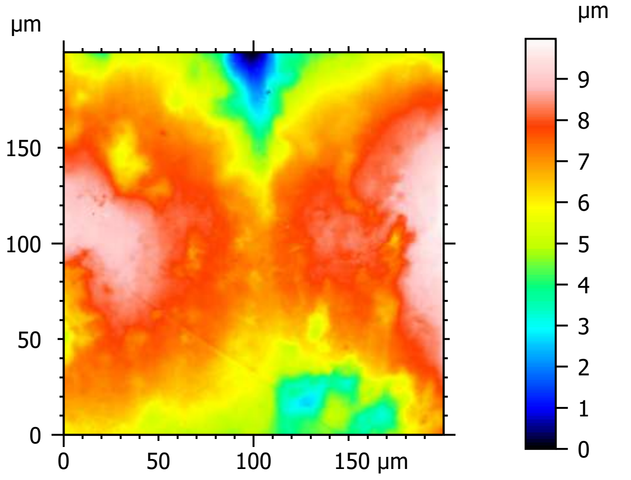

CG-Ce-c23-380-lm-dex-cusp1-bd

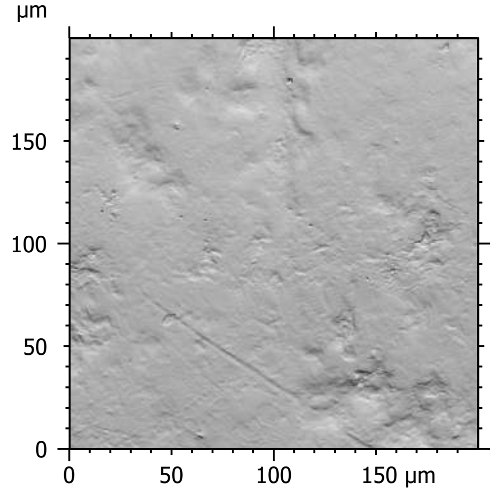

CG-Ce-c23-381-lm-sin-cusp1-bd

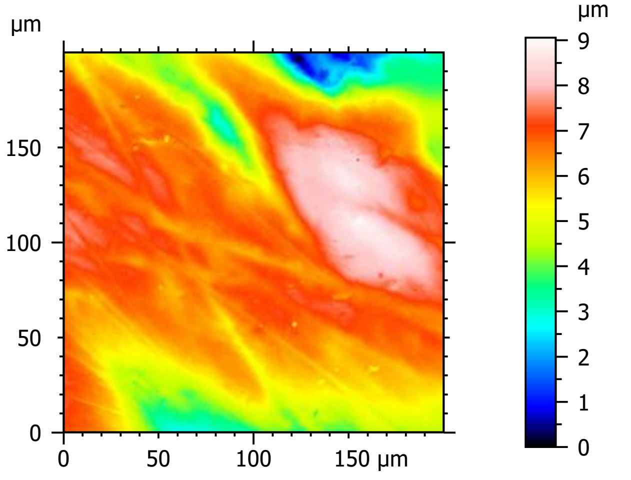

CG-Ce-c23-381-lm-sin-cusp1-bd

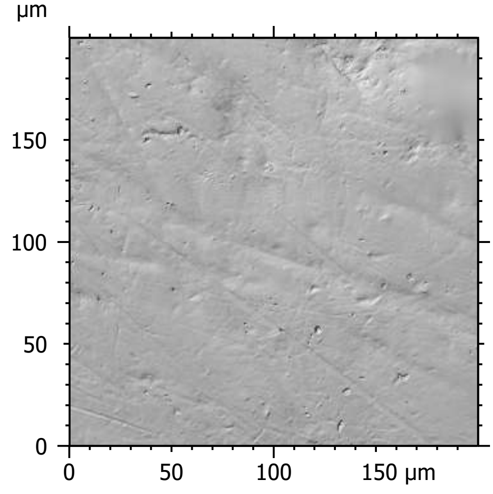

Leica Map Premium 8.2.9843

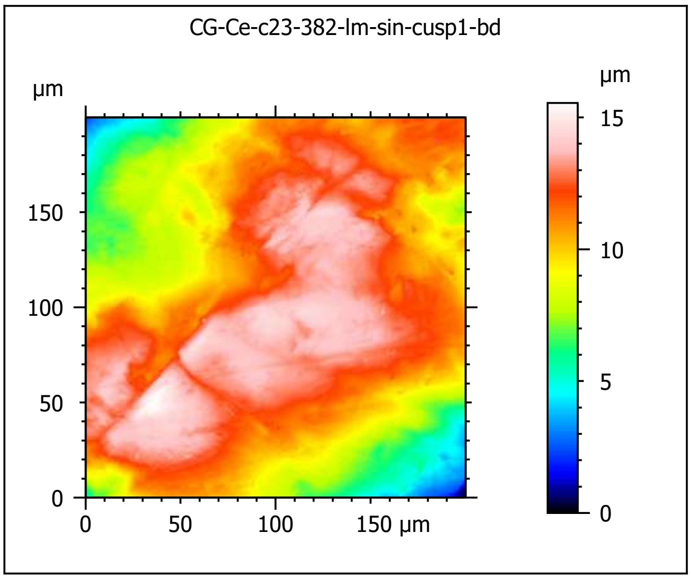

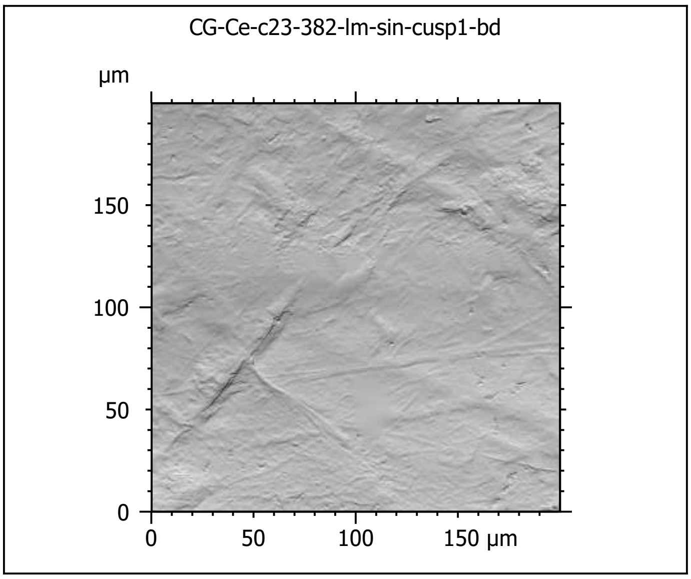

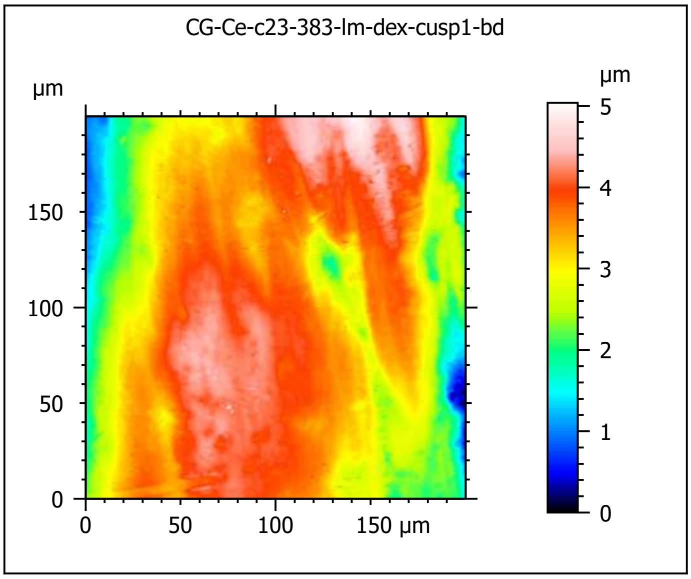

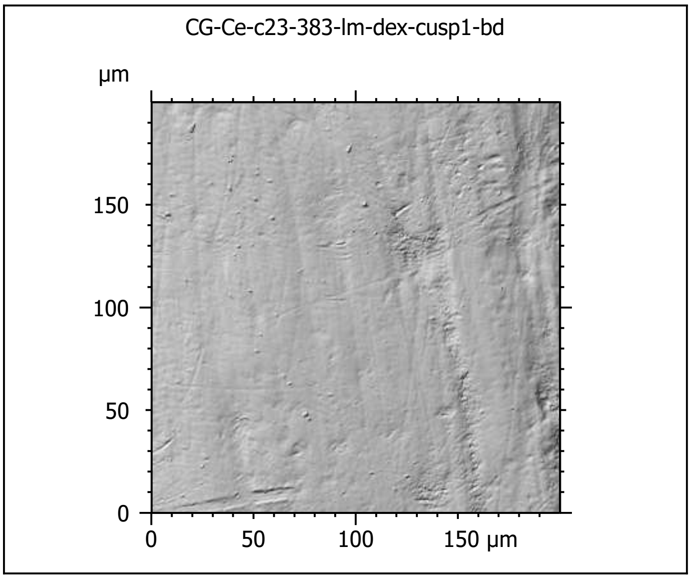

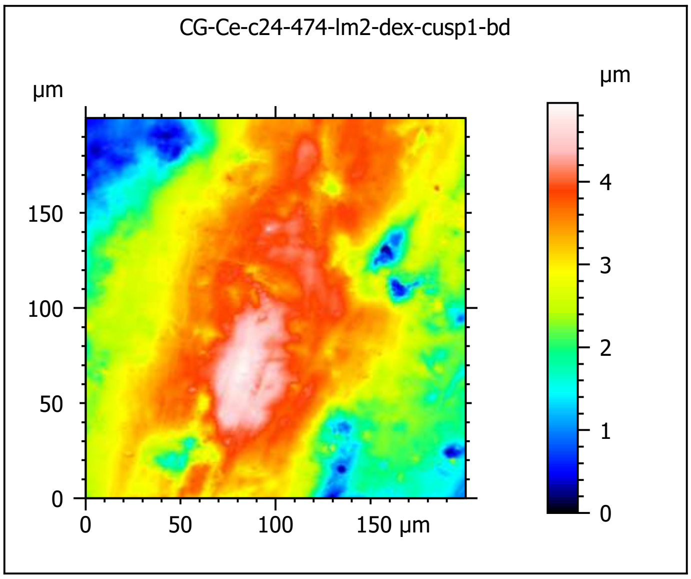

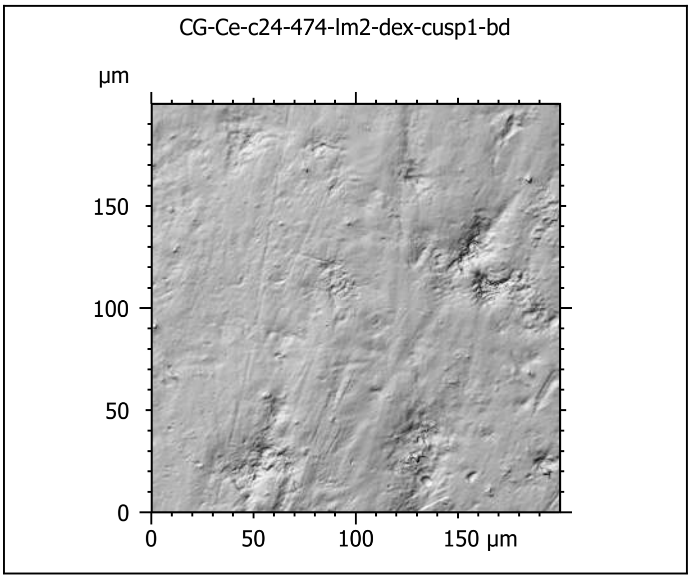

Leica Map Premium 8.2.9843

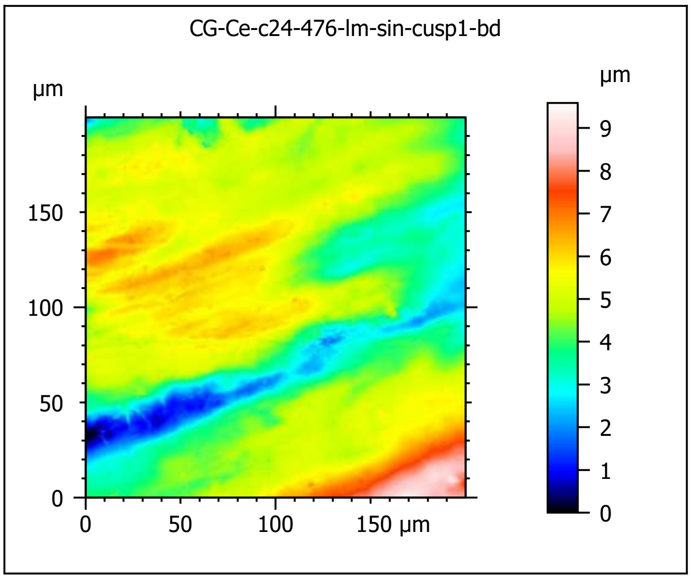

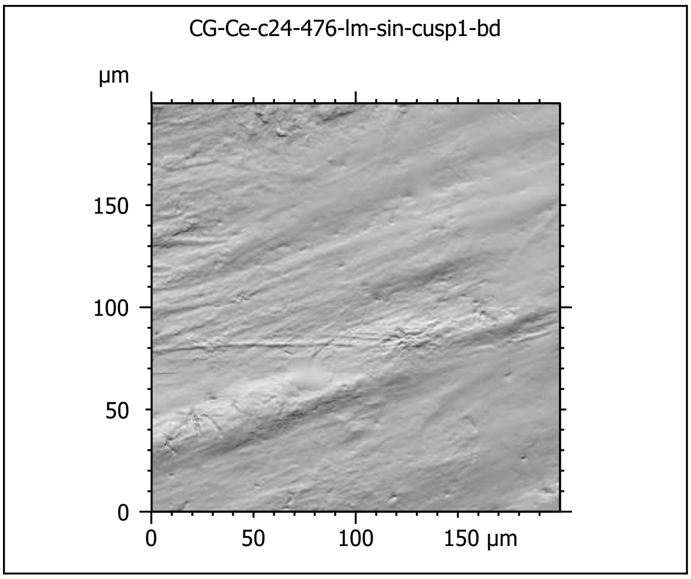

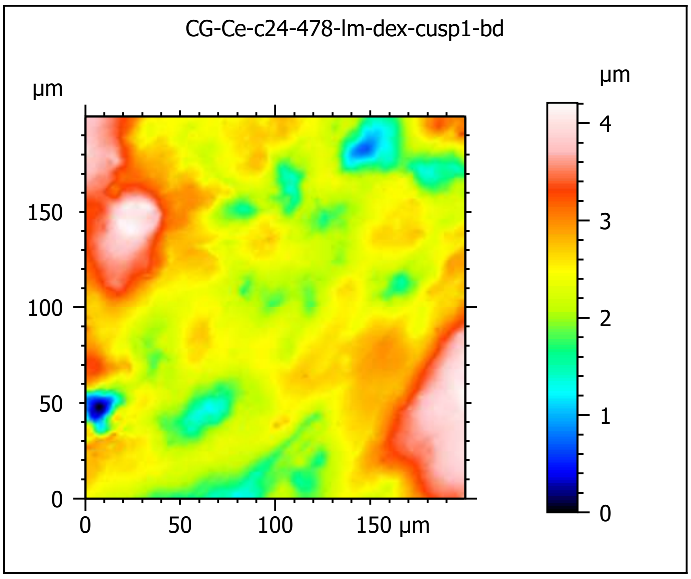

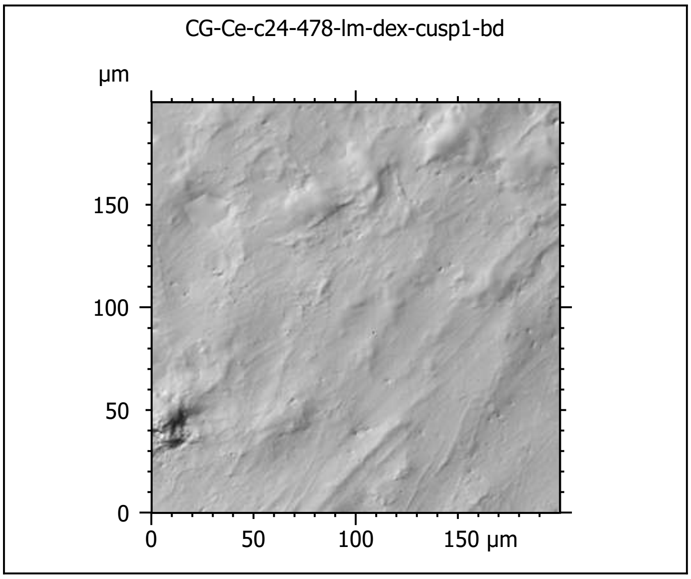

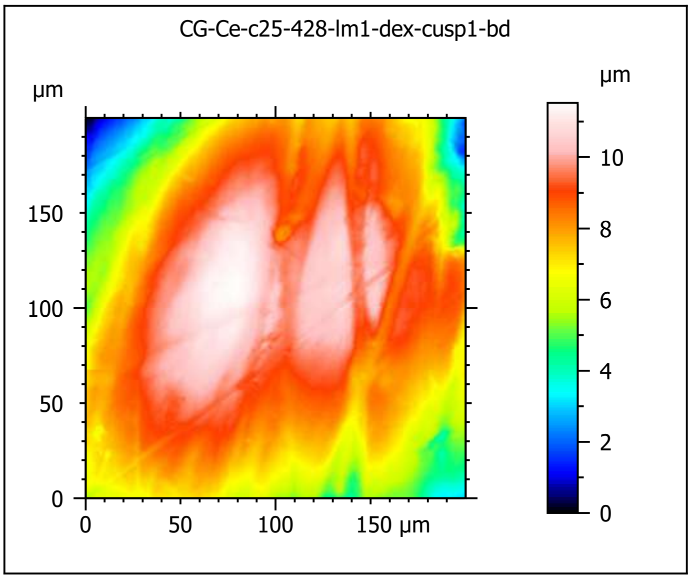

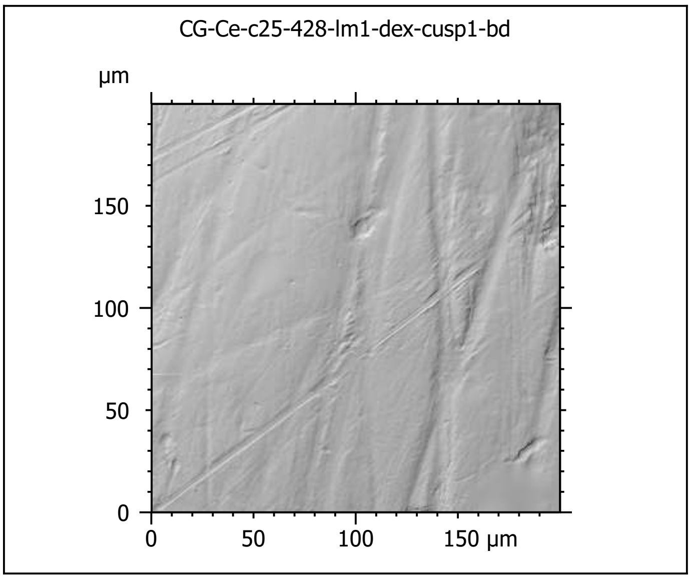

Leica Map Premium 8.2.9843

CG-Ce-c25-429-lm2-dex-cusp1-bd

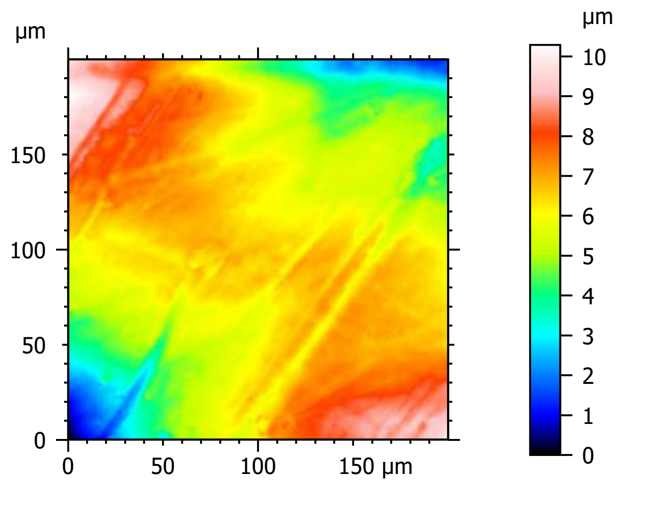

CG-Ce-c25-429-lm2-dex-cusp1-bd

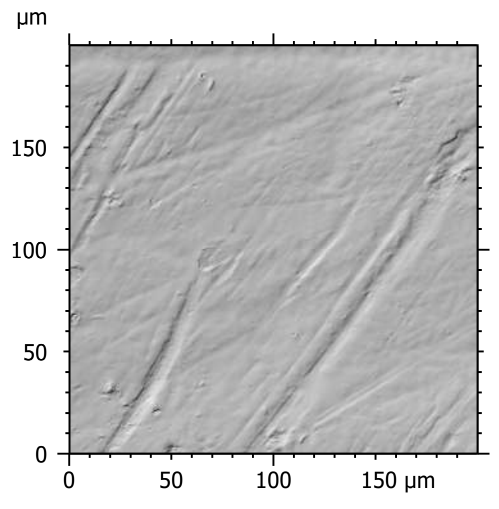

CG-Ce-c25-431-lm2-dex-cusp1-bm

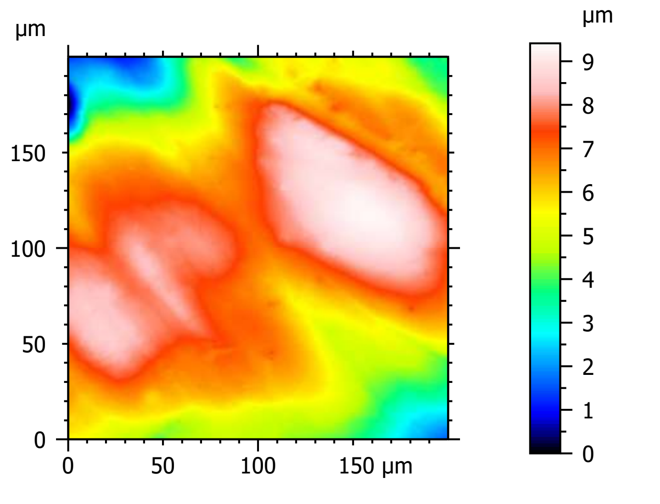

CG-Ce-c25-431-lm2-dex-cusp1-bm

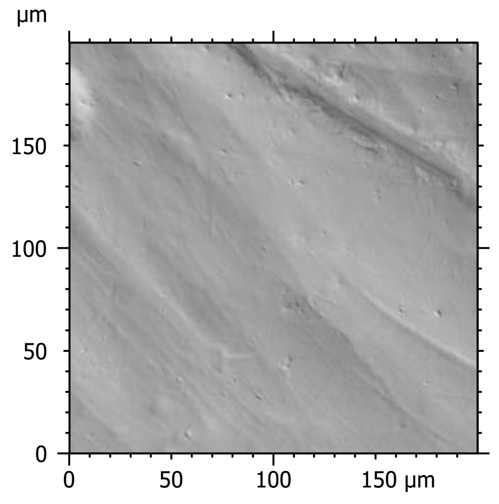

CG-Ce-c25-432-lm2-dex-cusp1-bd

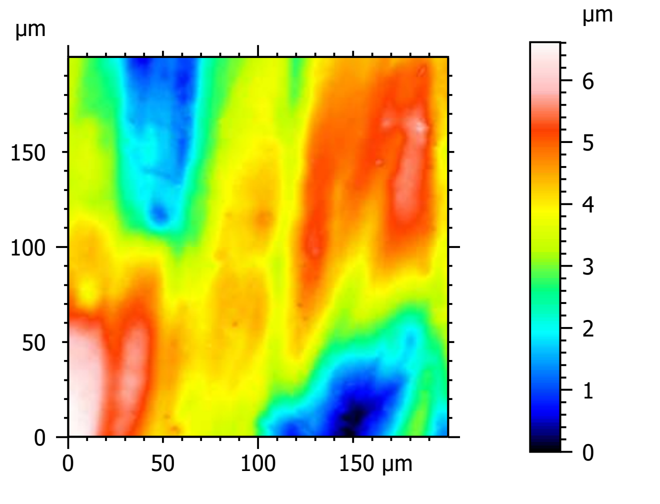

CG-Ce-c25-432-lm2-dex-cusp1-bd

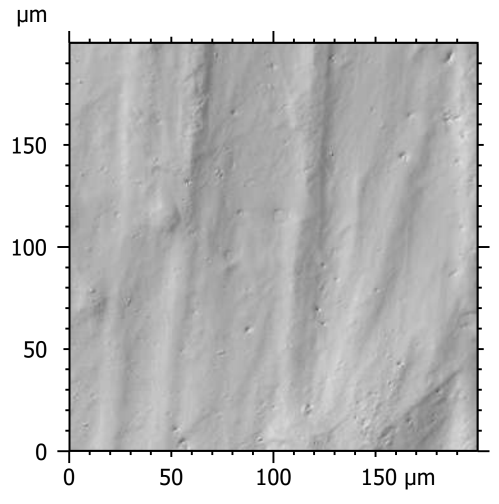

Leica Map Premium 8.2.9843

CG-Ce-c25-433-lm-dex-cusp1-bd

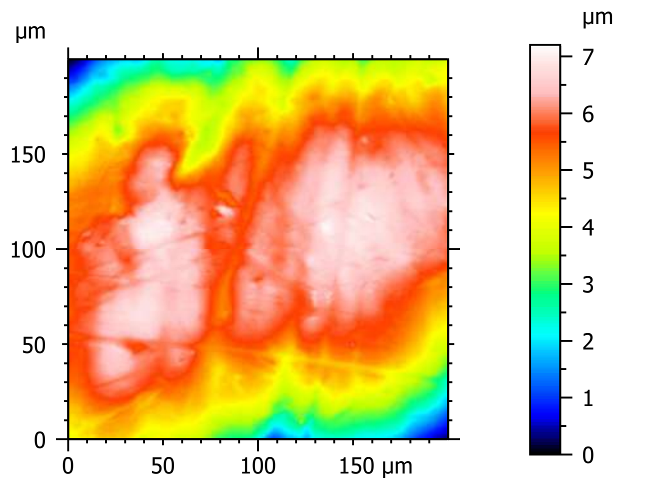

CG-Ce-c25-433-lm-dex-cusp1-bd

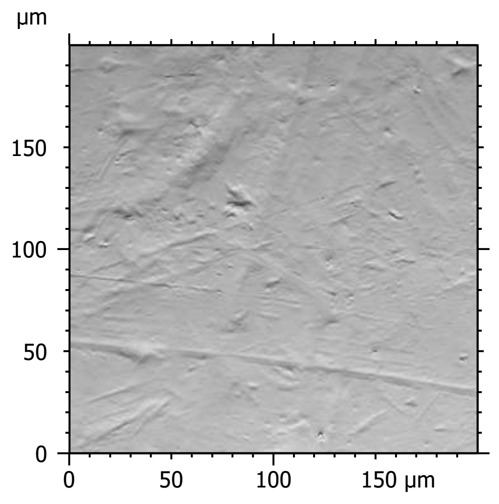

CG-Ce-c25-434-lm2-dex-cusp1-bd

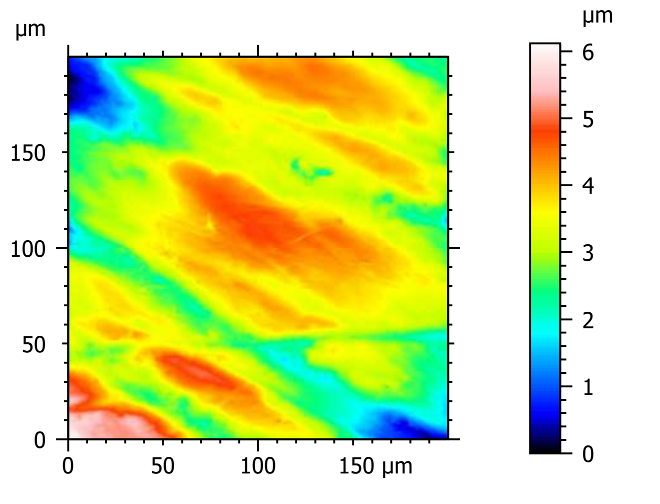

CG-Ce-c25-434-lm2-dex-cusp1-bd

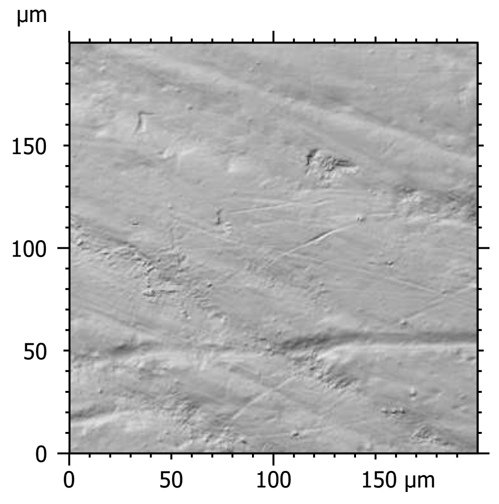

CG-Ce-c25-435-lm3-dex-cusp1-bd

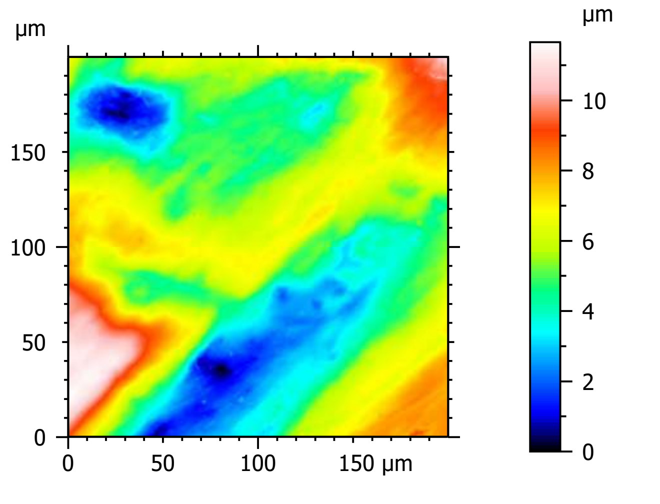

CG-Ce-c25-435-lm3-dex-cusp1-bd

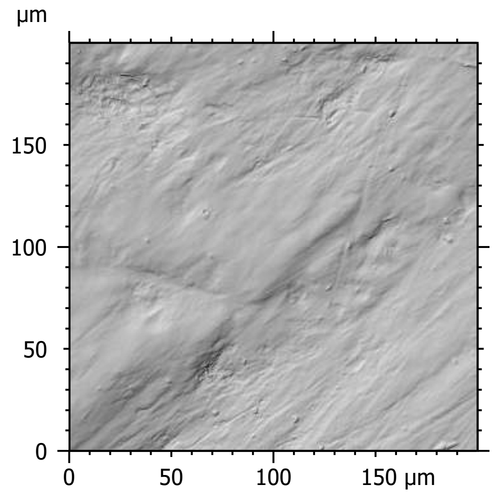

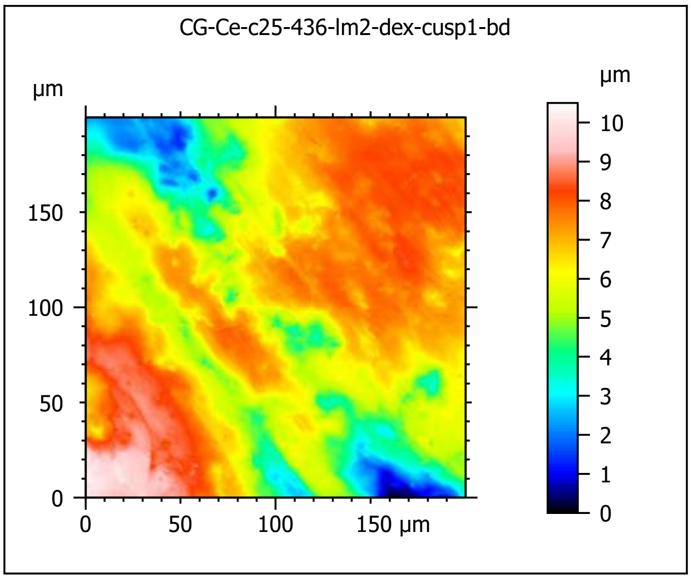

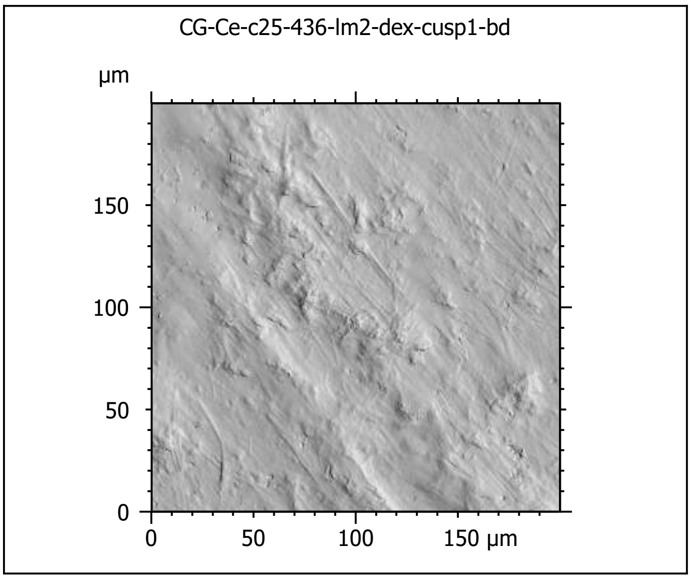

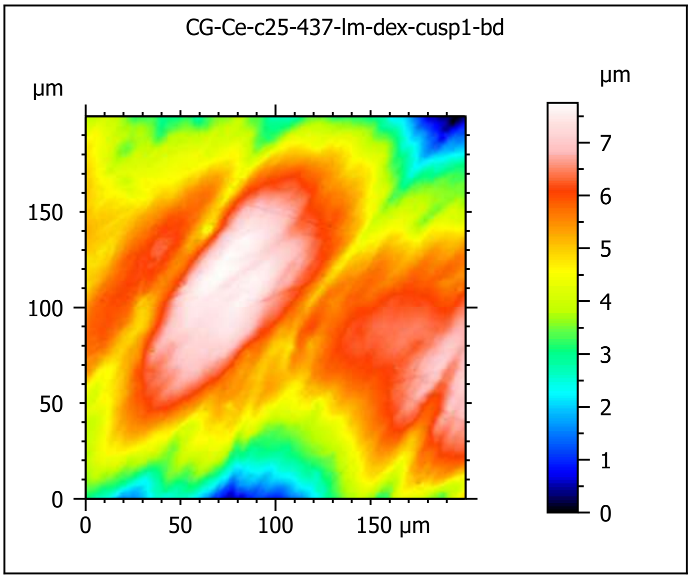

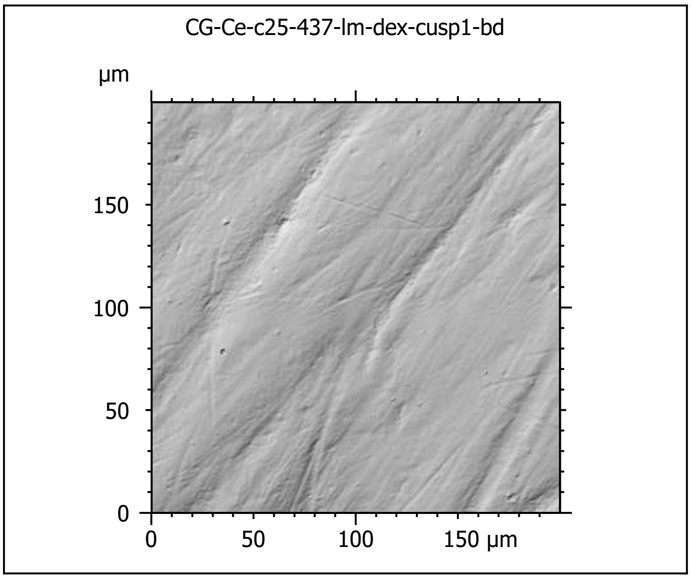

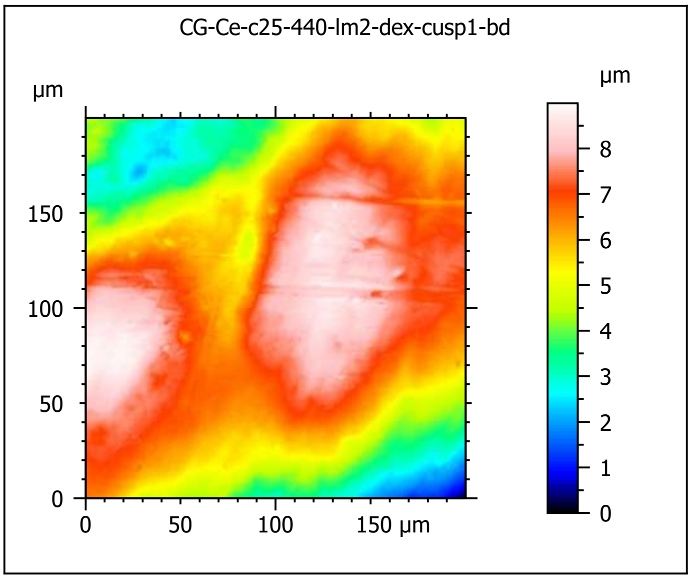

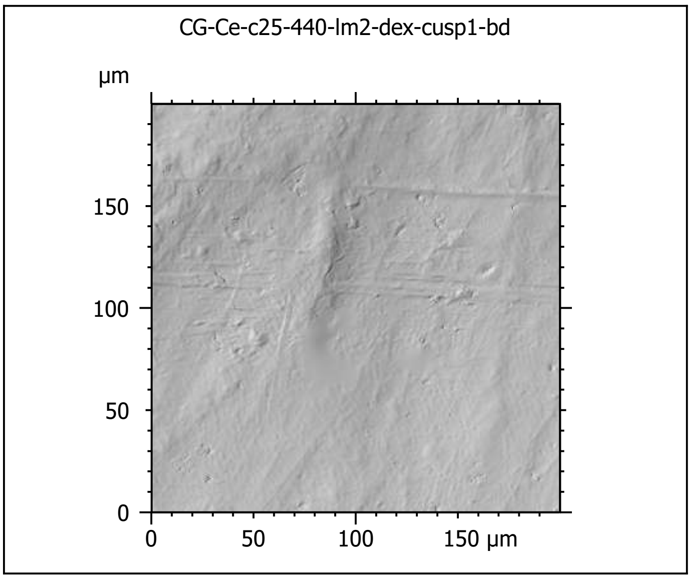

Leica Map Premium 8.2.9843

CG-Ce-c25-441-lm-dex-cusp1-bd

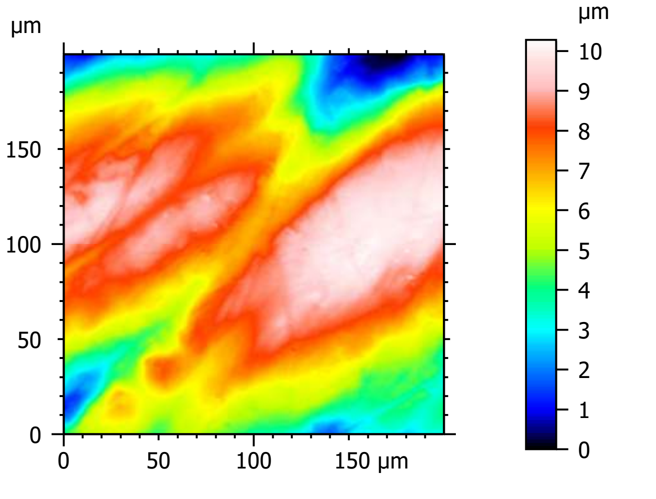

CG-Ce-c25-441-lm-dex-cusp1-bd

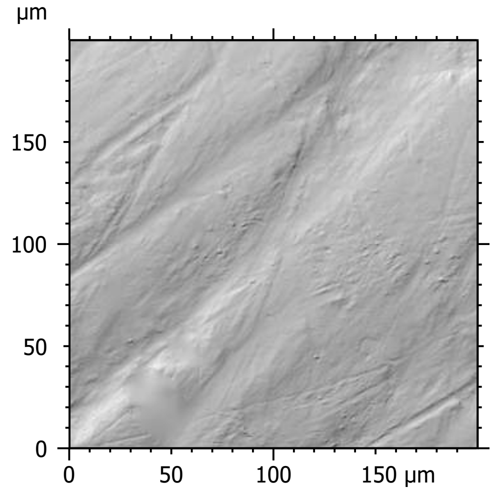

CG-Ce-c26-461-lm2-sin-cusp1-bd

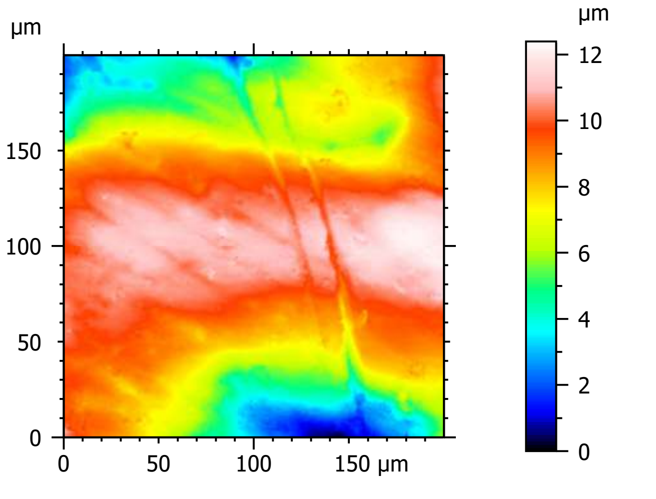

CG-Ce-c26-461-lm2-sin-cusp1-bd

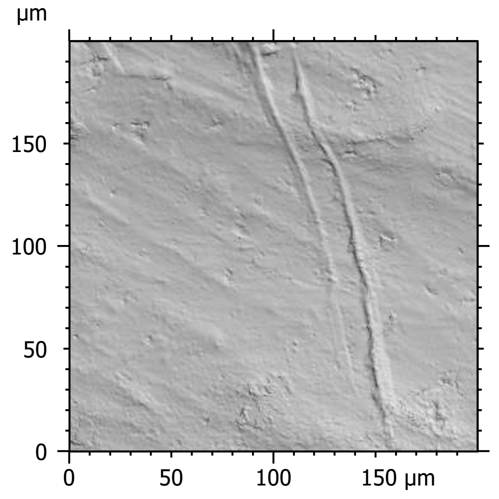

Leica Map Premium 8.2.9843

"A long-term perspective on Neandertal environment and subsistence: insights from the dental micro-texture analysis of hunted ungulates at Combe-Grenal (Dordogne, France)"

authors: Berlioz, E.; Capdepon, E.; Discamps, E.

Appendice 2:
surfaces scanned by E. Berlioz and E. Capdepon, pre-treatement by E. Berlioz and E. Capdepon, validation by E. Berlioz (2019)

***Cervus elaphus*- Block G**

CG-Ce-c27-349-lm-cusp-bd

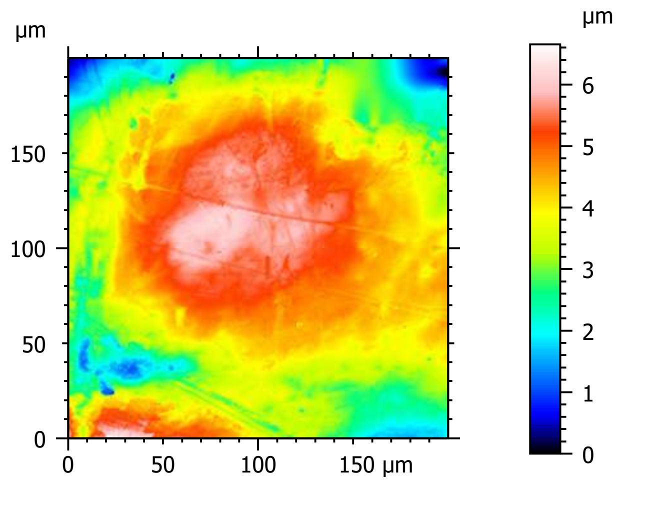

CG-Ce-c27-349-lm-cusp-bd

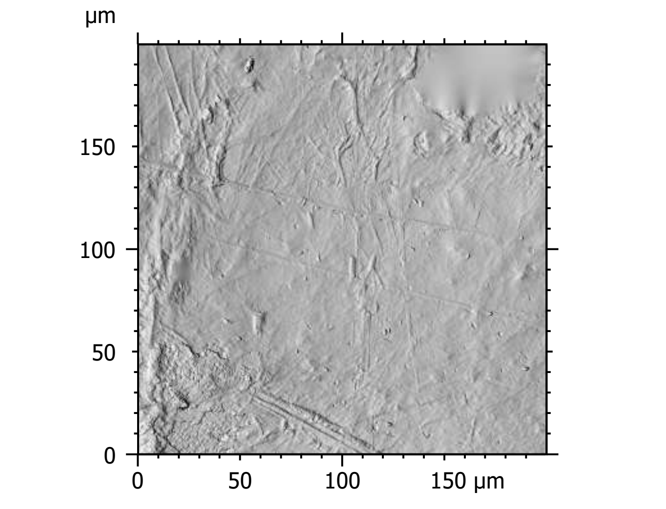

CG-Ce-c27-351-lm3-sin-cusp1-bd

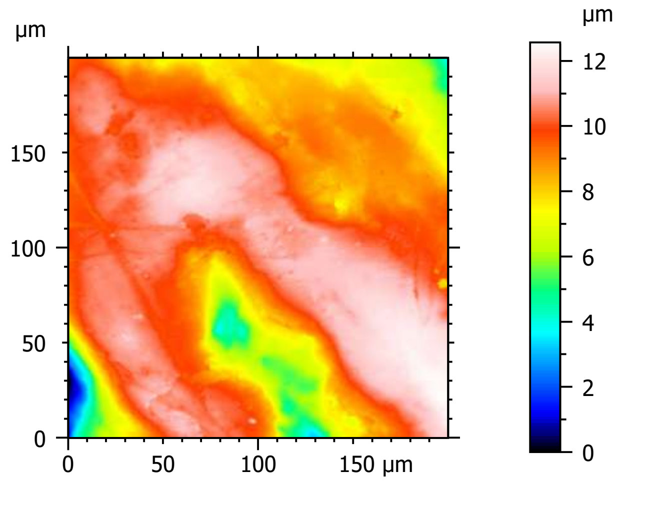

CG-Ce-c27-351-lm3-sin-cusp1-bd

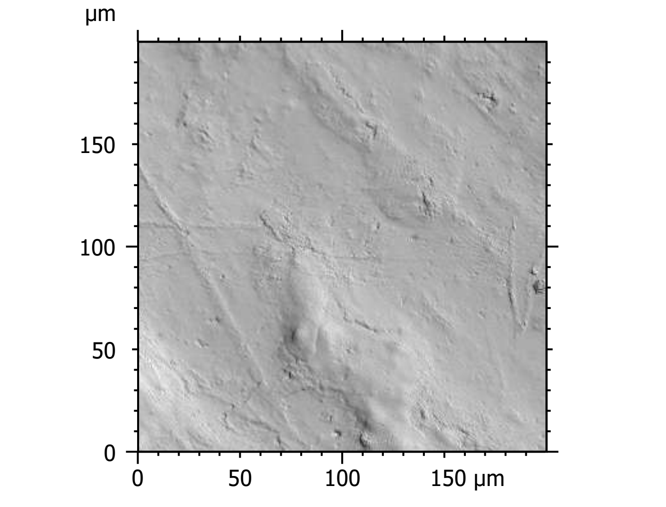

CG-Ce-c27-354-lm2-sin-cusp1-bd

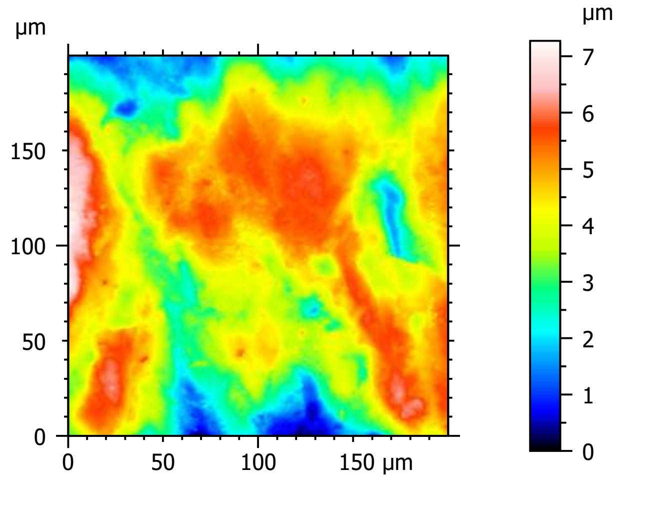

CG-Ce-c27-354-lm2-sin-cusp1-bd

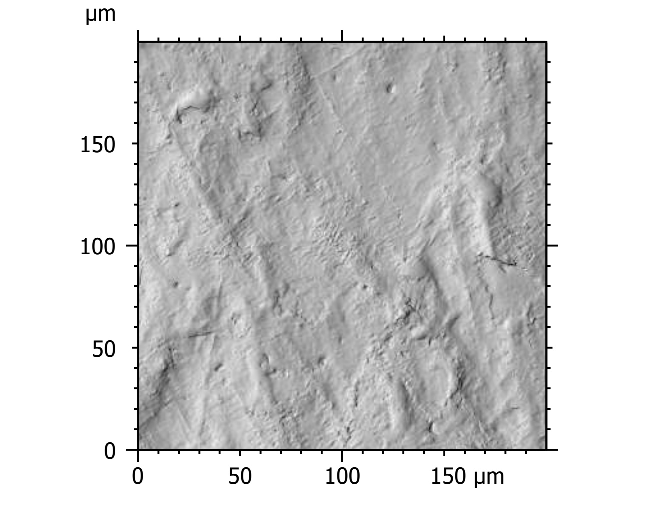

Leica Map Premium 8.2.9645

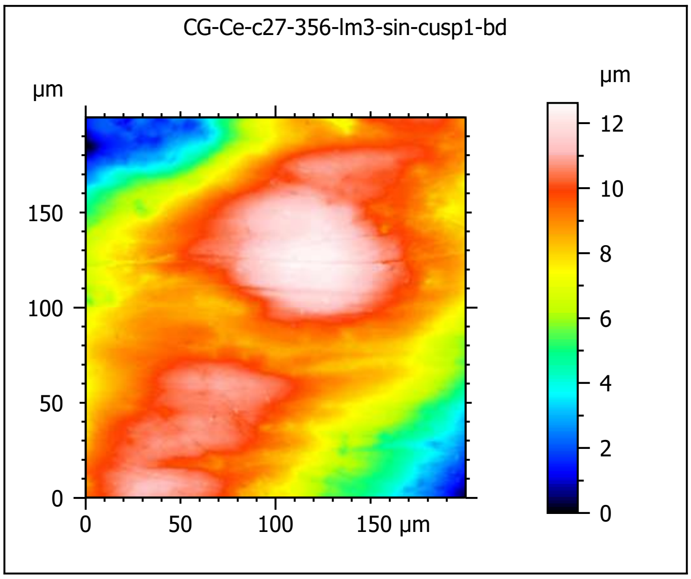

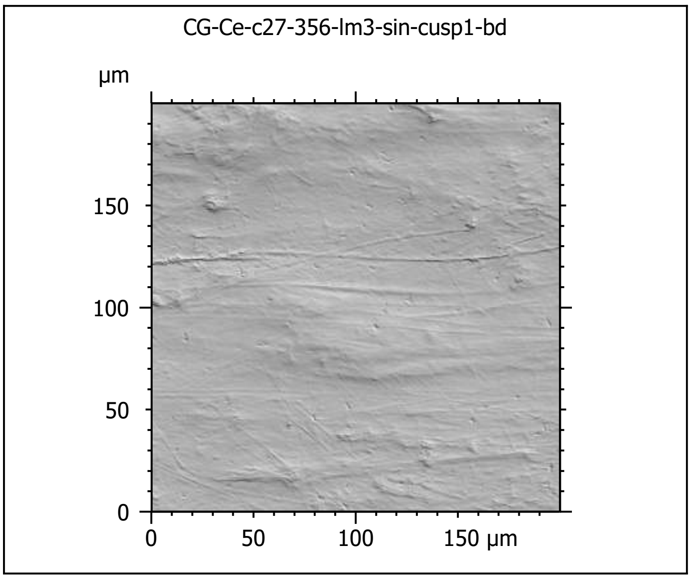

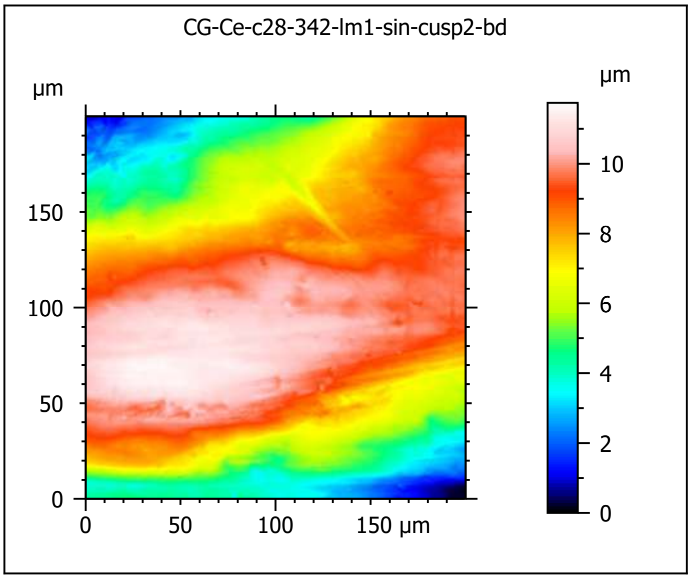

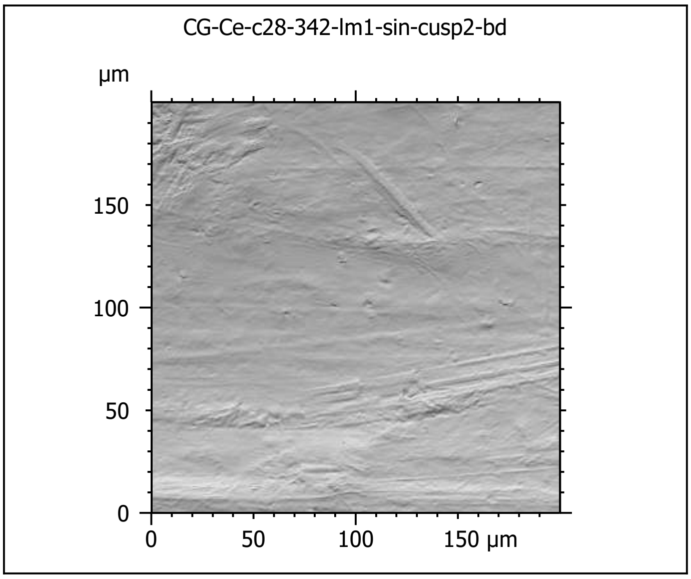

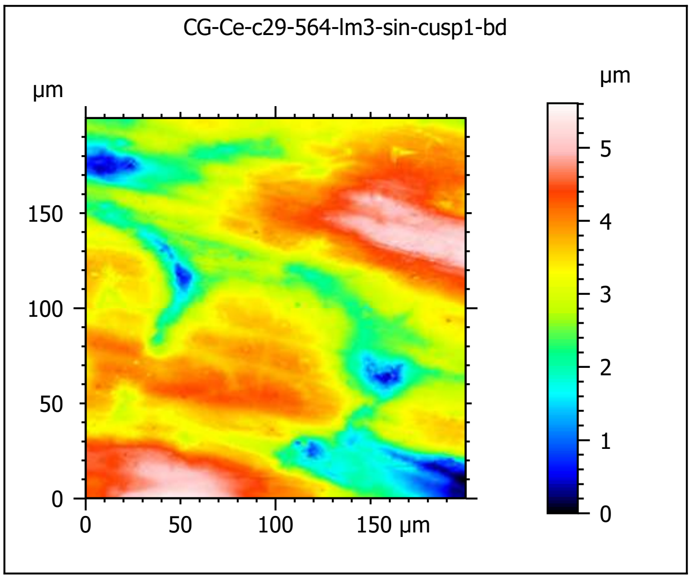

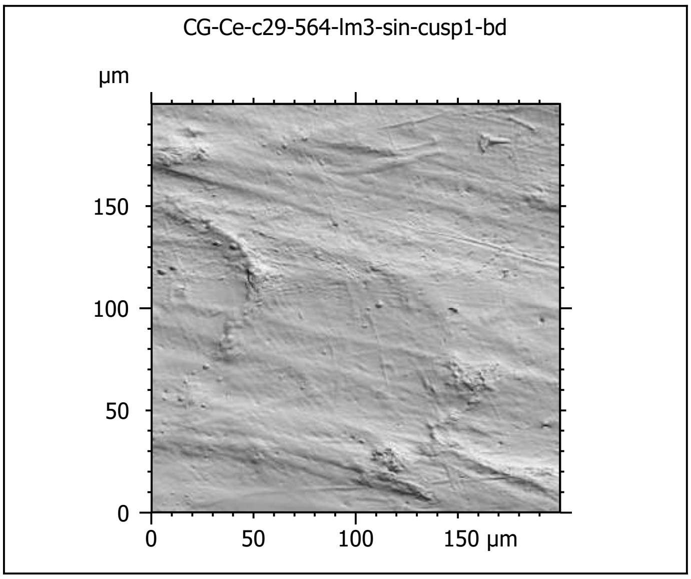

Leica Map Premium 8.2.9843

CG-Ce-c29-565-lm2-dsin-cusp1-bd

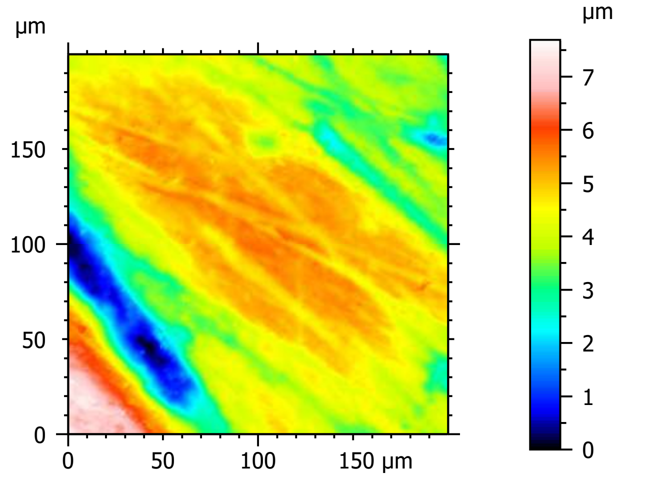

CG-Ce-c29-565-lm2-dsin-cusp1-bd

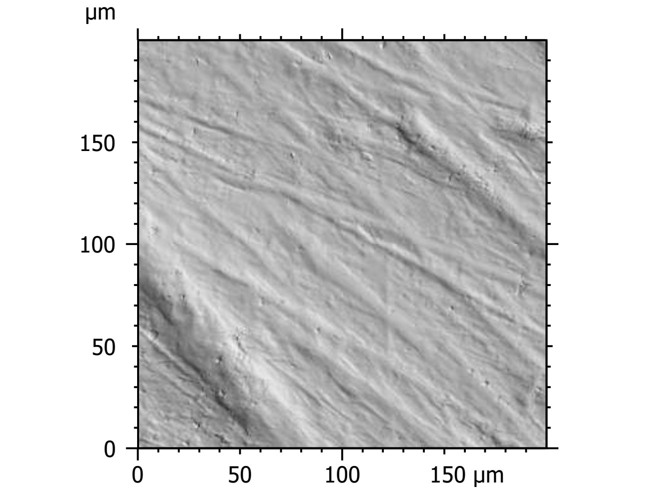

CG-Ce-c29-566-lm2-sin-cusp1-bd

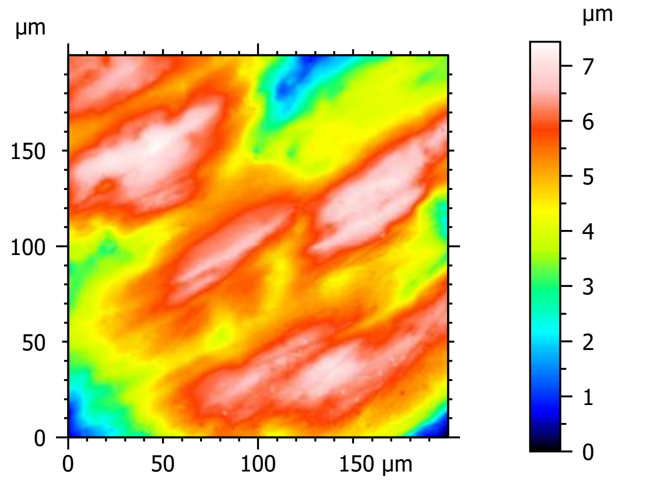

CG-Ce-c29-566-lm2-sin-cusp1-bd

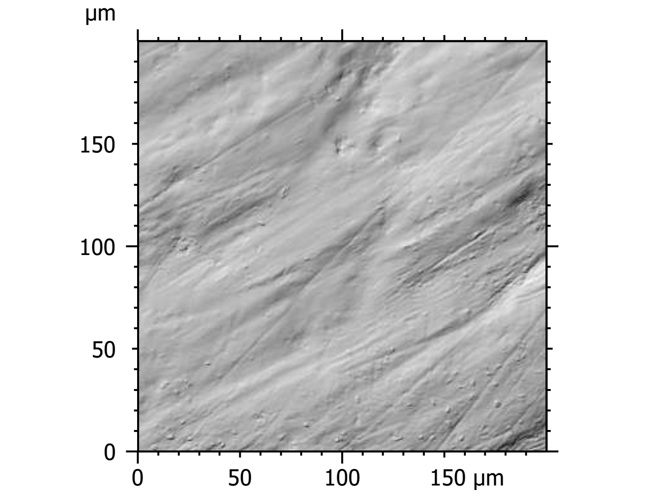

CG-Ce-c29-567-lm3-sin-cusp1-bd

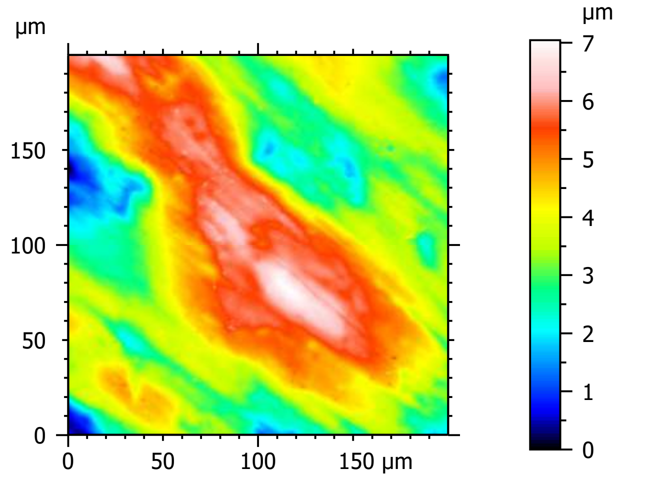

CG-Ce-c29-567-lm3-sin-cusp1-bd

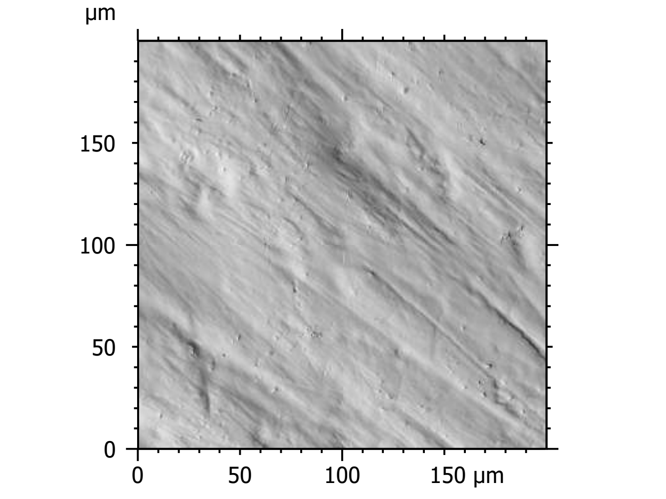

Leica Map Premium 8.2.9843

CG-Ce-c29-568-lm-dex-cusp1-bd

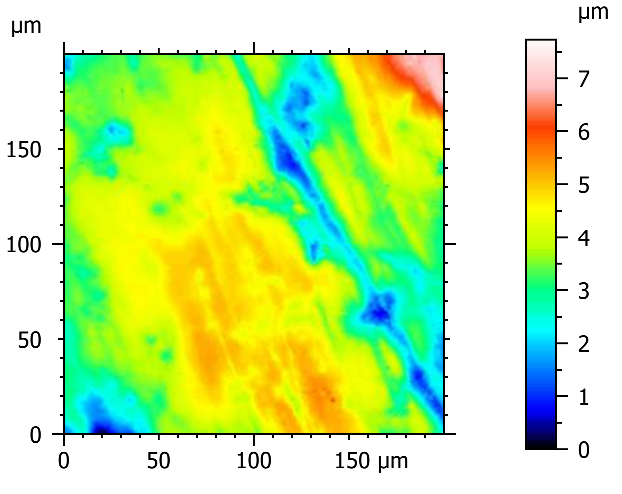

CG-Ce-c29-568-lm-dex-cusp1-bd

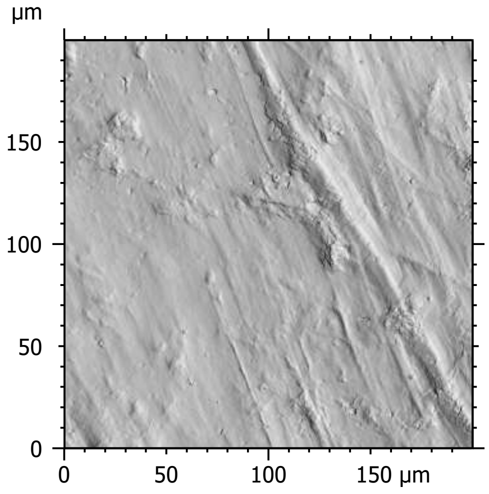

CG-Ce-c29-569-lm2-dex-cusp1-bd

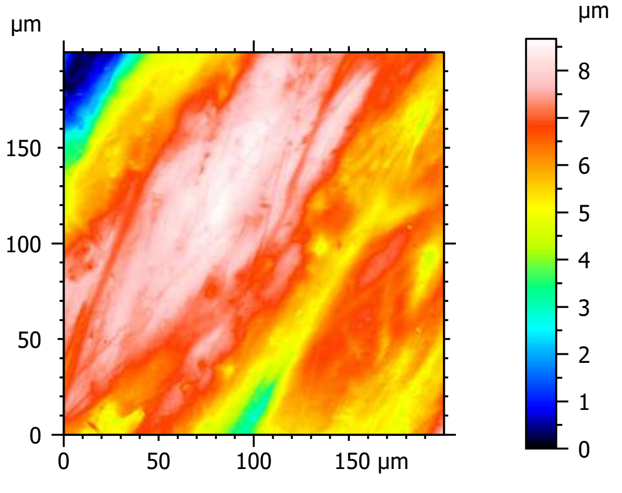

CG-Ce-c29-569-lm2-dex-cusp1-bd

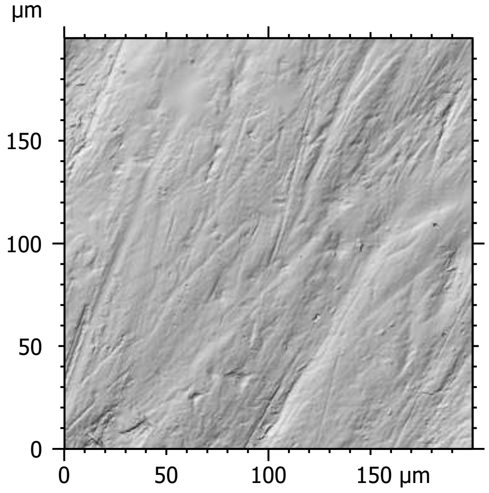

CG-Ce-c29-570-lm3-dex-cusp1-bd

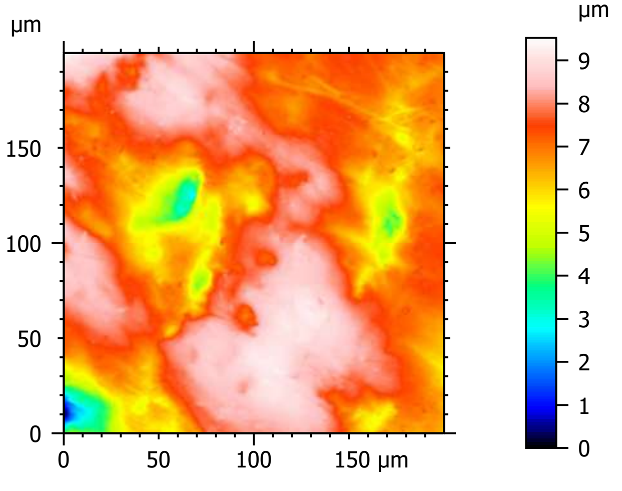

CG-Ce-c29-570-lm3-dex-cusp1-bd

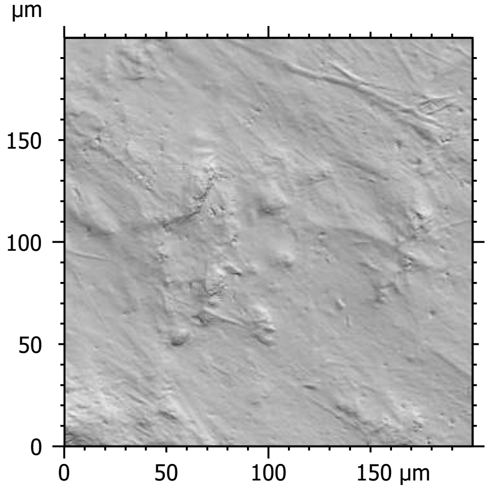

CG-Ce-c29-571-lm-dex-cusp1-bd

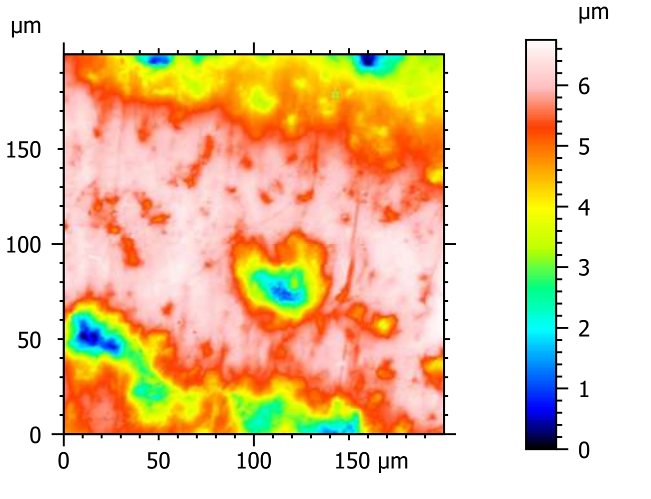

CG-Ce-c29-571-lm-dex-cusp1-bd

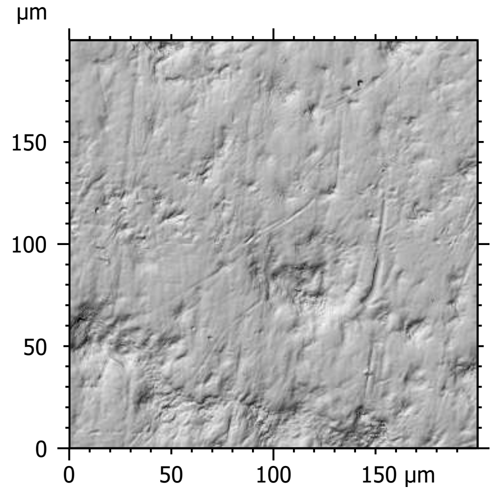

CG-Ce-c29-573-lm2-dex-cusp1-bd

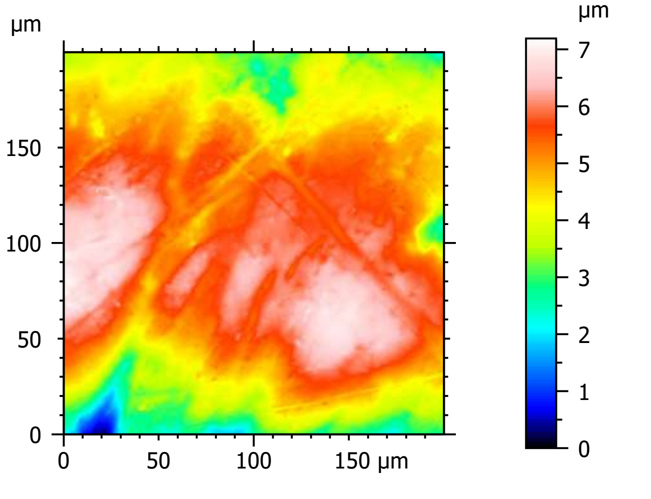

CG-Ce-c29-573-lm2-dex-cusp1-bd

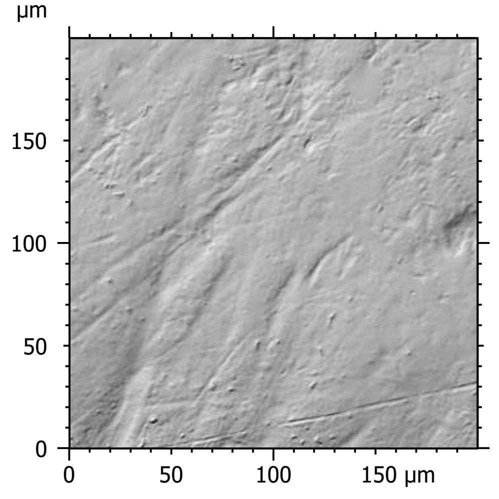

CG-Ce-c29-574-lm2-dex-cusp1-bd

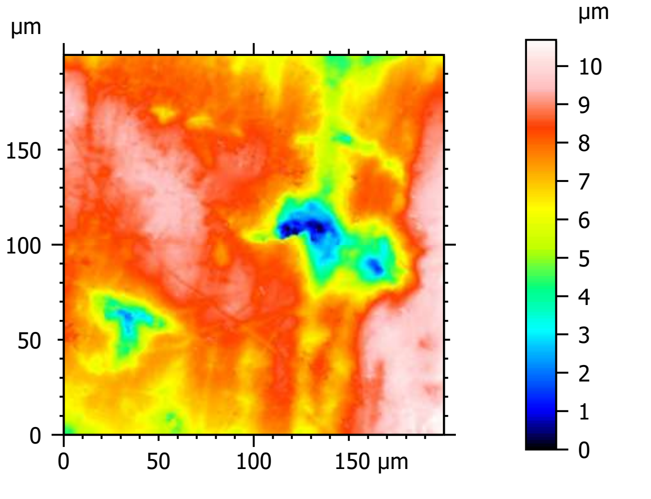

CG-Ce-c29-574-lm2-dex-cusp1-bd

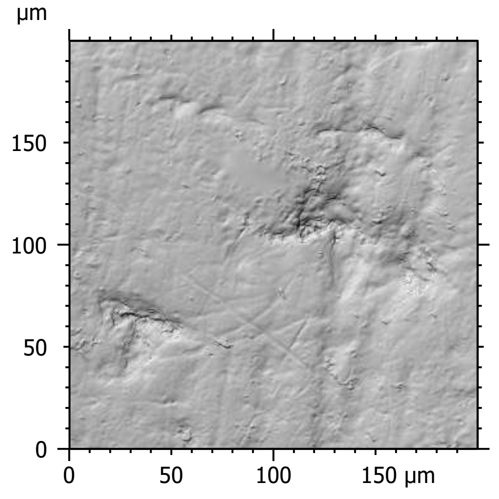

Leica Map Premium 8.2.9843

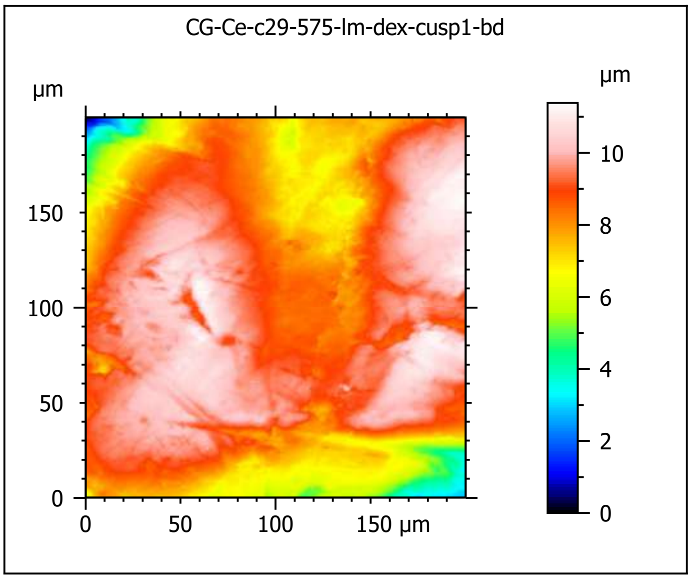

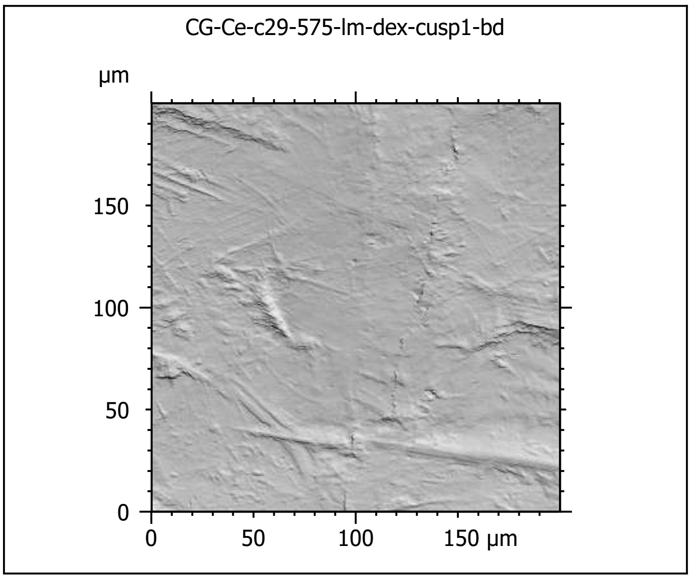

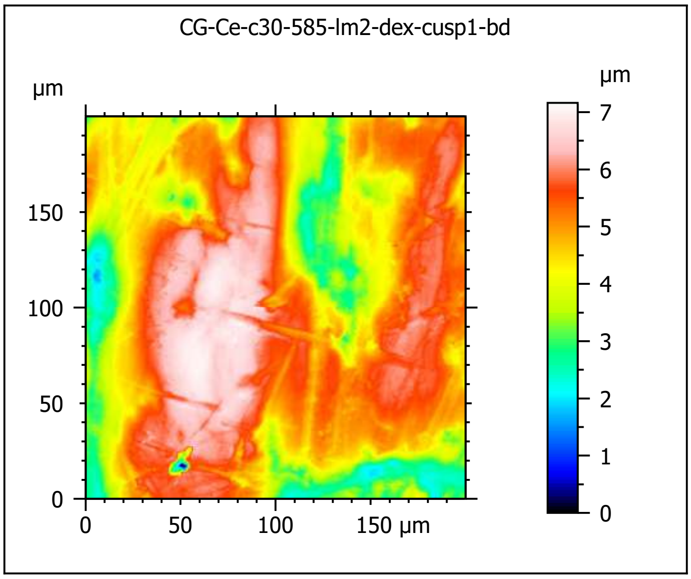

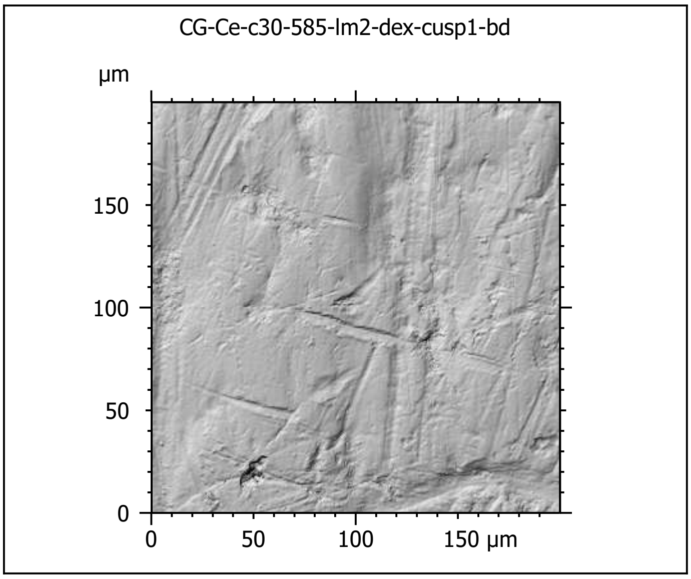

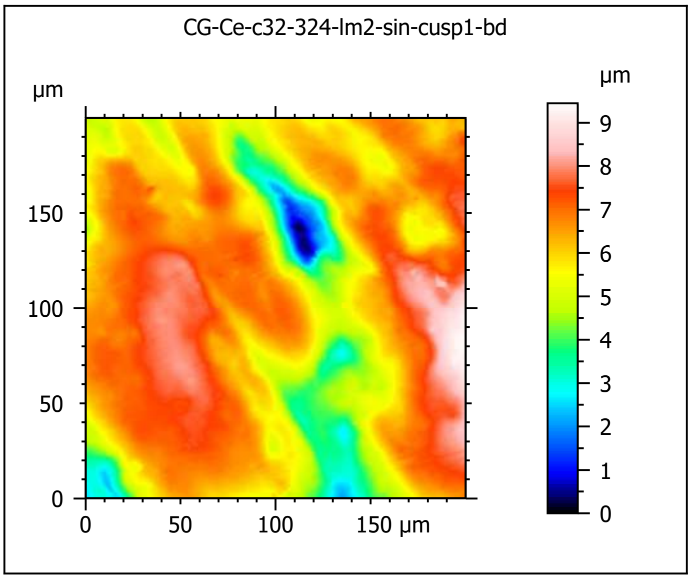

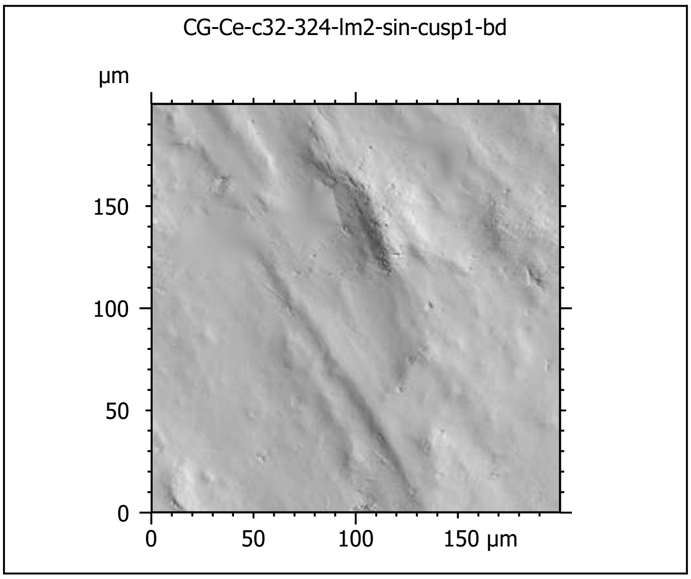

Leica Map Premium 8.2.9843

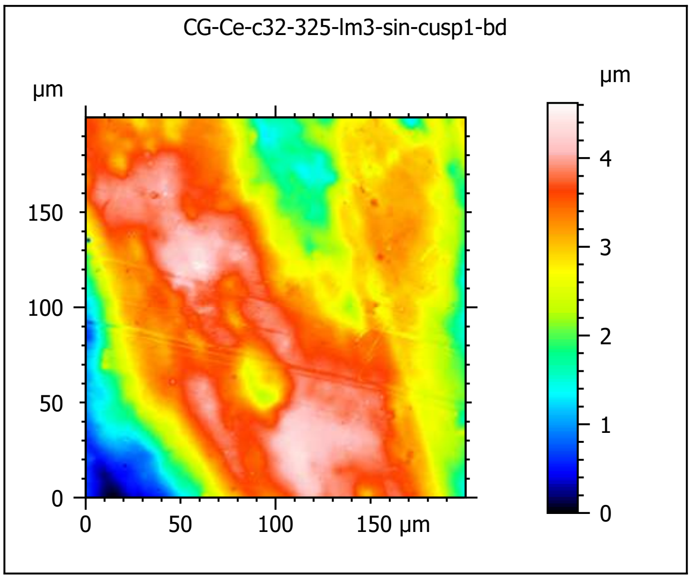
CG-Ce-c32-325-lm3-sin-cusp1-bd
µm
150
100
50
0
0
50
100
150 µm
µm
4
3
2
1
0

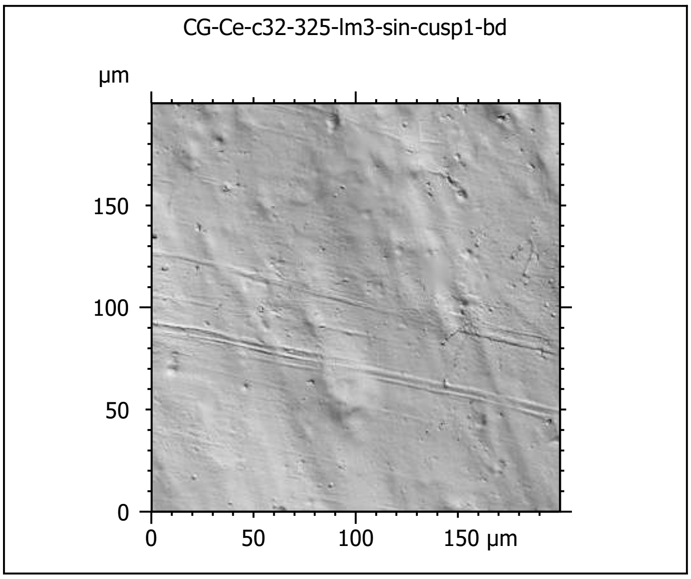
CG-Ce-c32-325-lm3-sin-cusp1-bd
µm
150
100
50
0
0
50
100
150 µm

"A long-term perspective on Neandertal environment and subsistence: insights from the dental micro-texture analysis of hunted ungulates at Combe-Grenal (Dordogne, France)"

authors: Berlioz, E.; Capdepon, E.; Discamps, E.

Appendice 2:
surfaces scanned by E. Berlioz and E. Capdepon, pre-treatement by E. Berlioz and E. Capdepon, validation by E. Berlioz (2019)

***Cervus elaphus*- Block H**

CG-Ce-c33-540-lm2-dex-cusp1-bd

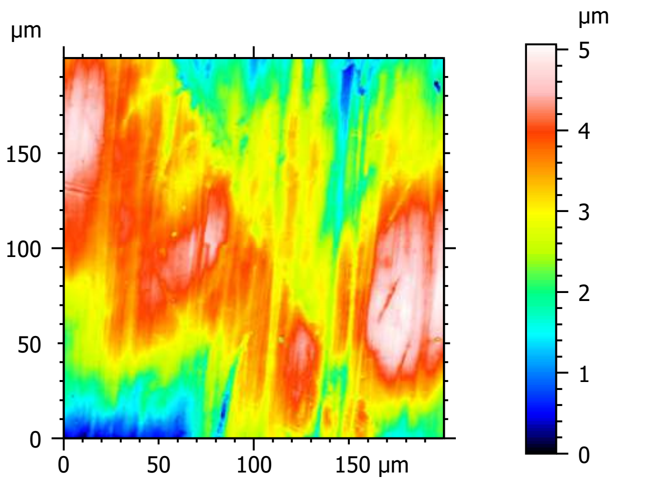

CG-Ce-c33-540-lm2-dex-cusp1-bd

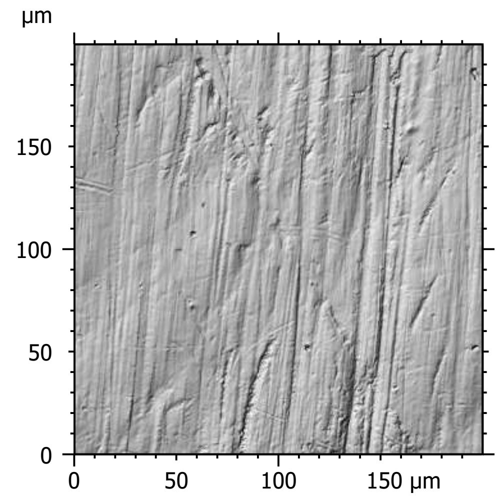

CG-Ce-c33-544-lm2-dex-cusp2-bd

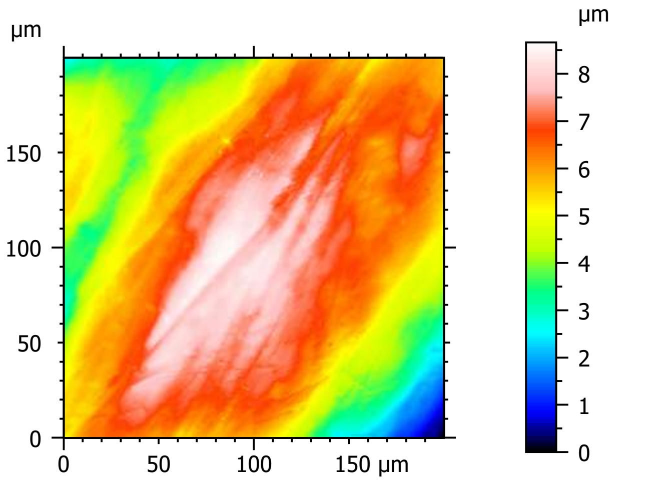

CG-Ce-c33-544-lm2-dex-cusp2-bd

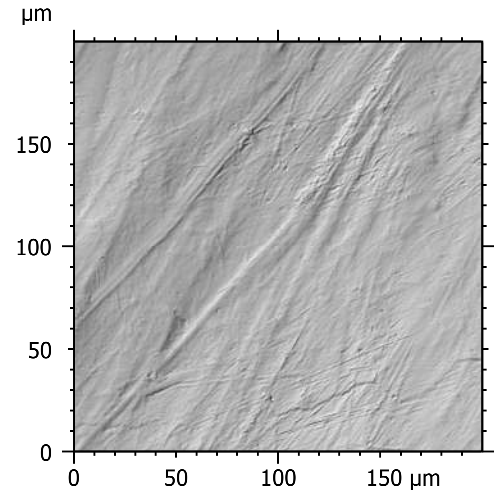

Leica Map Premium 8.2.9843

"A long-term perspective on Neandertal environment and subsistence: insights from the dental micro-texture analysis of hunted ungulates at Combe-Grenal (Dordogne, France)"

authors: Berlioz, E.; Capdepon, E.; Discamps, E.

Appendice 2:
surfaces scanned by E. Berlioz and E. Capdepon, pre-treatement by E. Berlioz and E. Capdepon, validation by E. Berlioz (2019)

***Cervus elaphus*- Block I**

CG-Ce-c35-521-lm3-sin-cusp1-bd

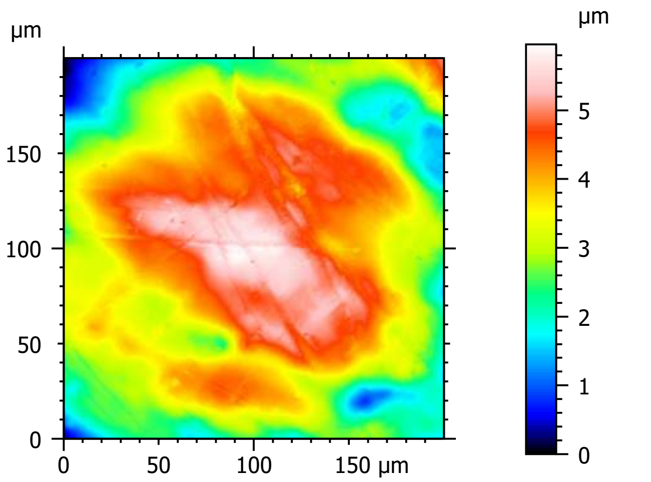

CG-Ce-c35-521-lm3-sin-cusp1-bd

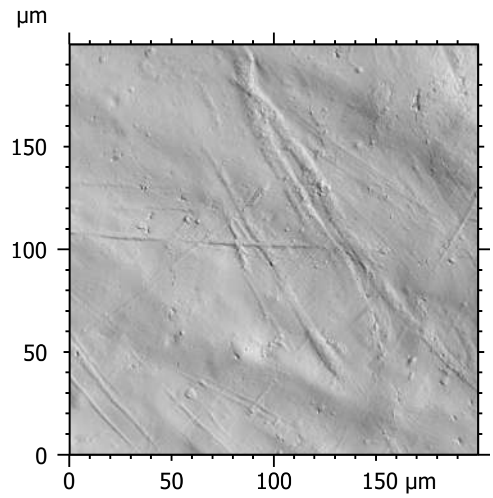

CG-Ce-c35-522-lm3-sin-cusp1-bd

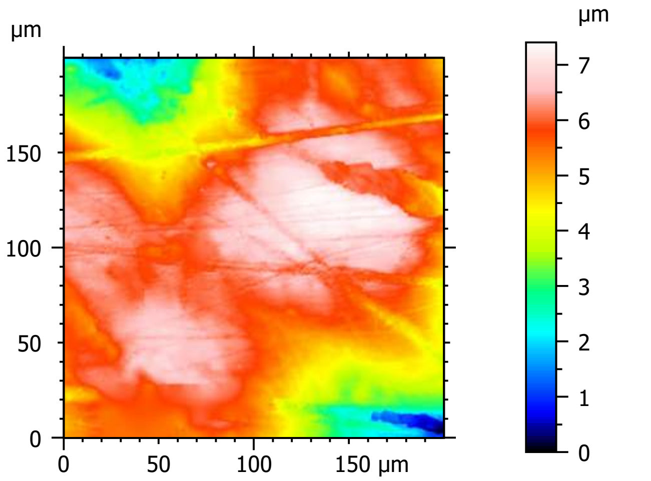

CG-Ce-c35-522-lm3-sin-cusp1-bd

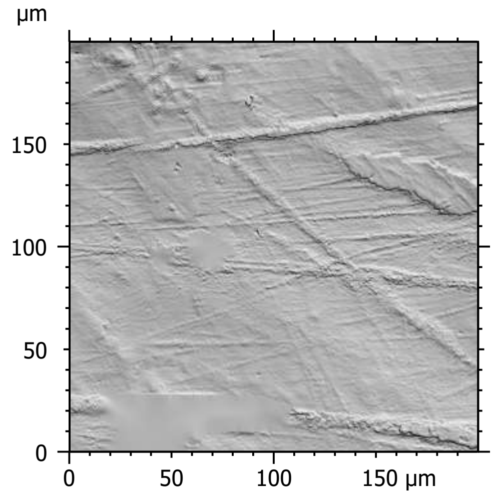

CG-Ce-c35-523-lm3-sin-cusp1-bd

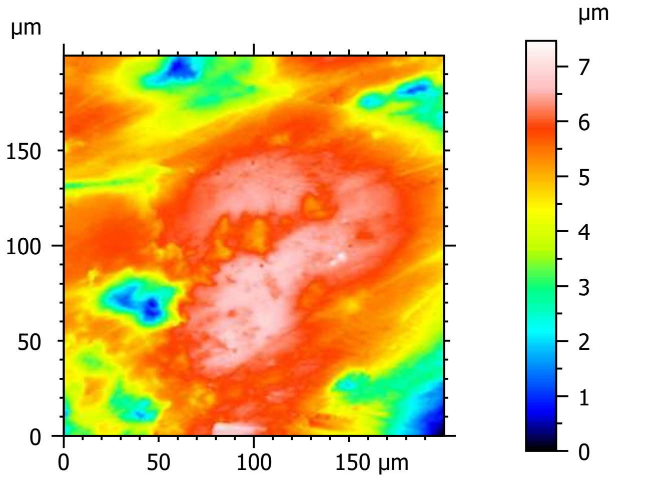

CG-Ce-c35-523-lm3-sin-cusp1-bd

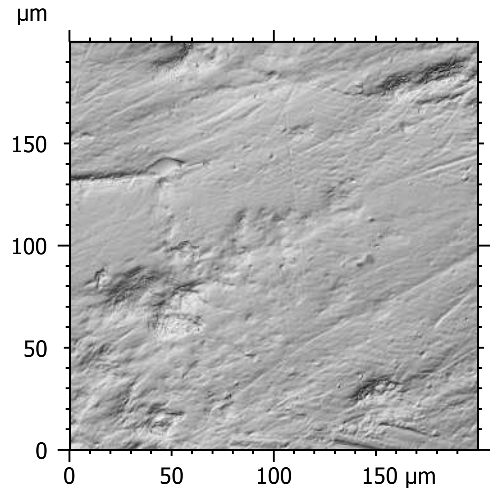

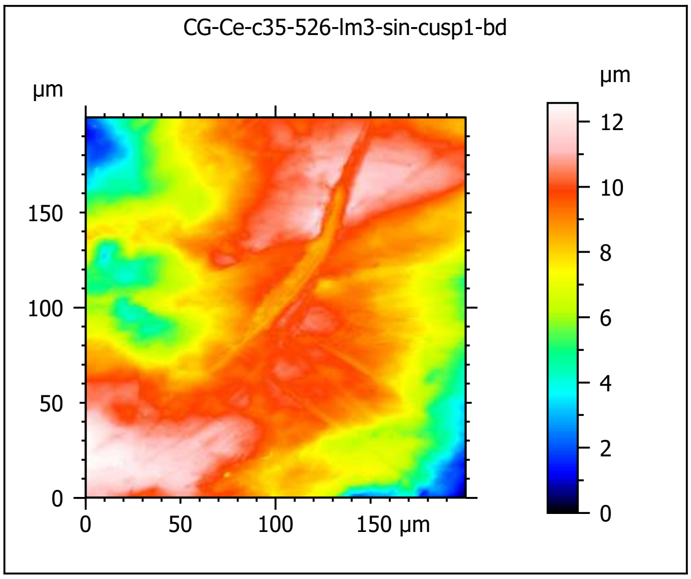

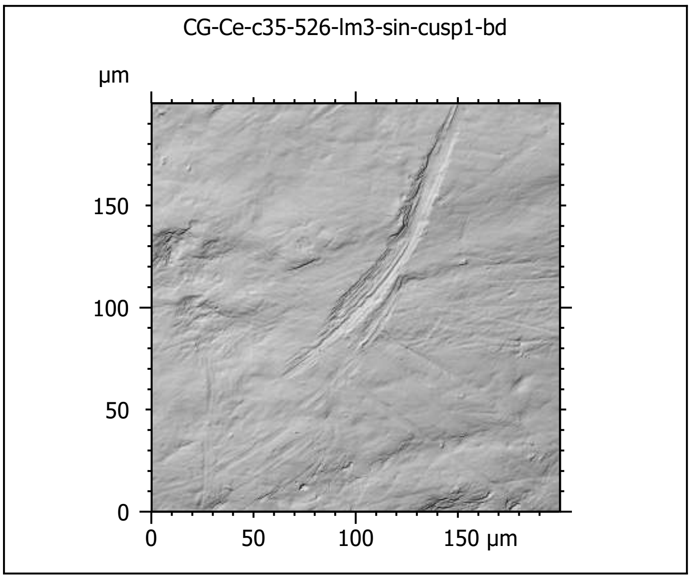

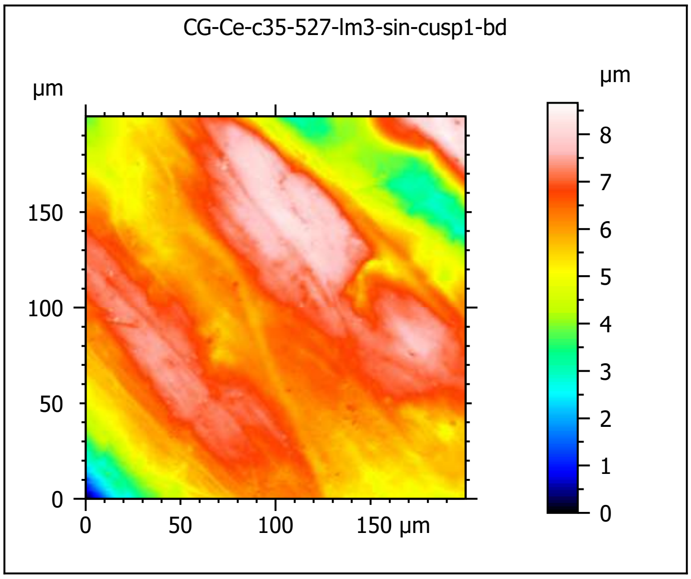

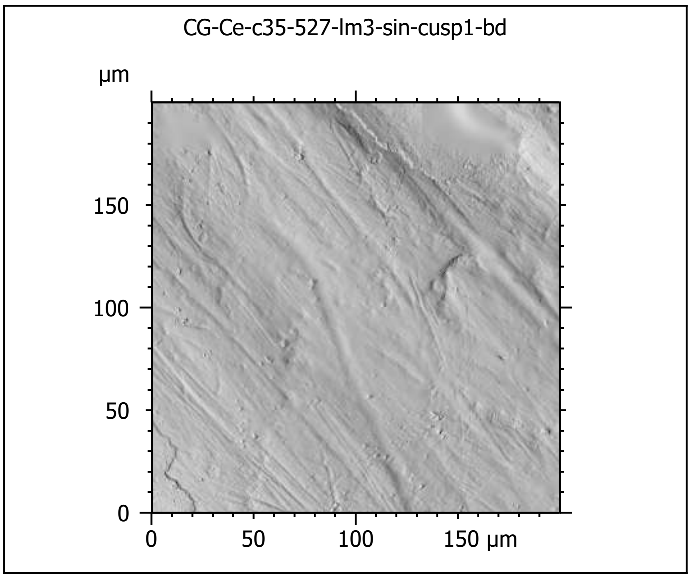

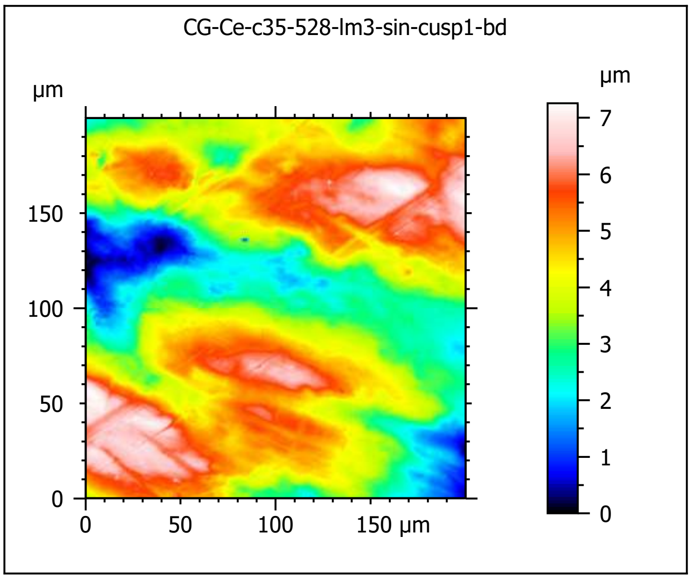

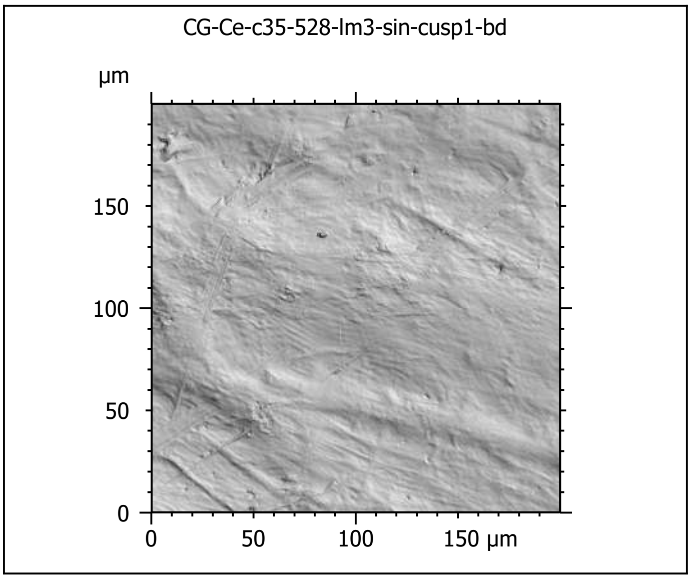

Leica Map Premium 8.2.9843

CG-Ce-c35-529-lm2-sin-cusp1-bm

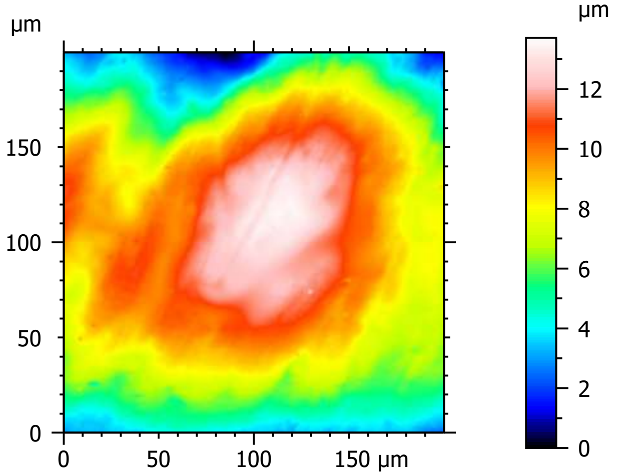

CG-Ce-c35-529-lm2-sin-cusp1-bm

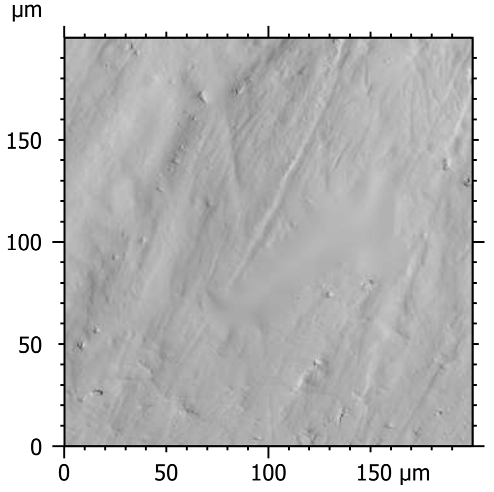

CG-Ce-c35-530-lm2-sin-cusp1-bd

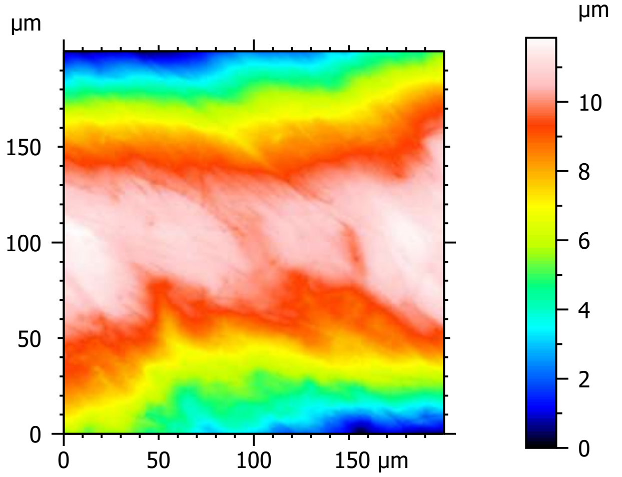

CG-Ce-c35-530-lm2-sin-cusp1-bd

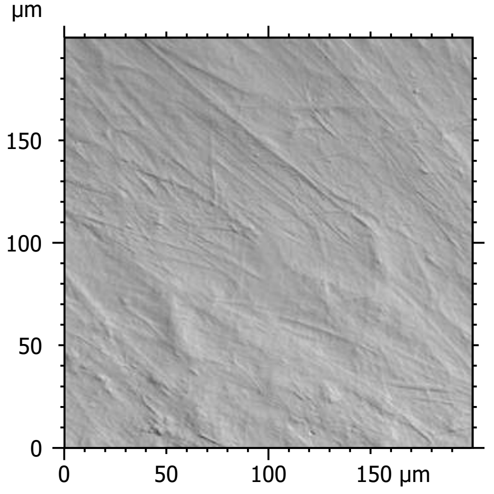

CG-Ce-c36-545-lm-sin-cusp1-bd

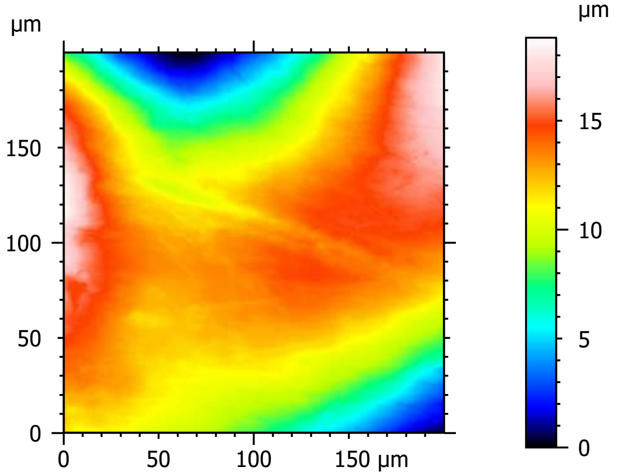

CG-Ce-c36-545-lm-sin-cusp1-bd

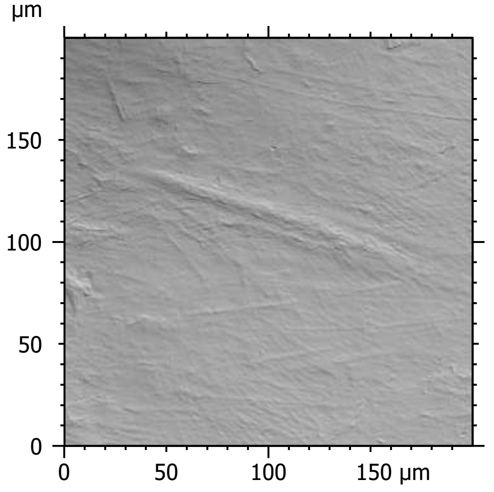

Leica Map Premium 8.2.9843

CG-Ce-c36-546-lm-dex-cusp1-bd

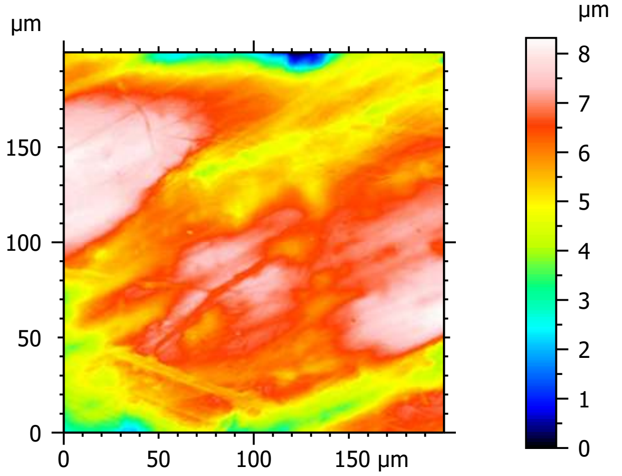

CG-Ce-c36-546-lm-dex-cusp1-bd

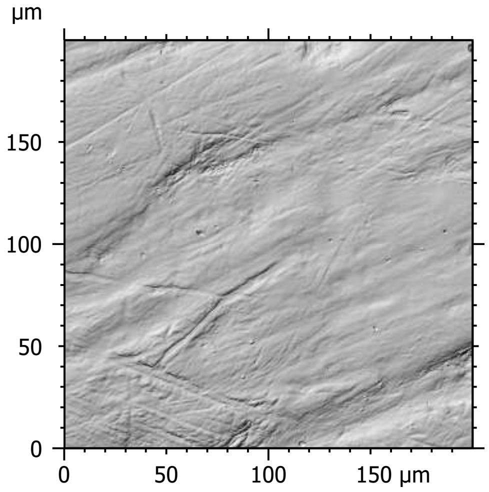

CG-Ce-c36-547-lm3-sin-cusp1-bd

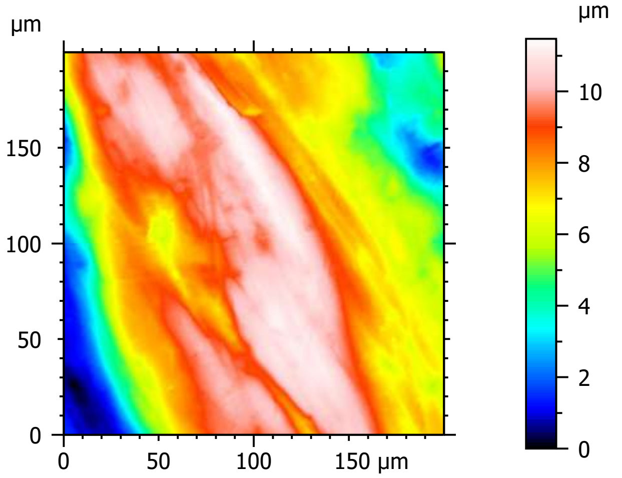

CG-Ce-c36-547-lm3-sin-cusp1-bd

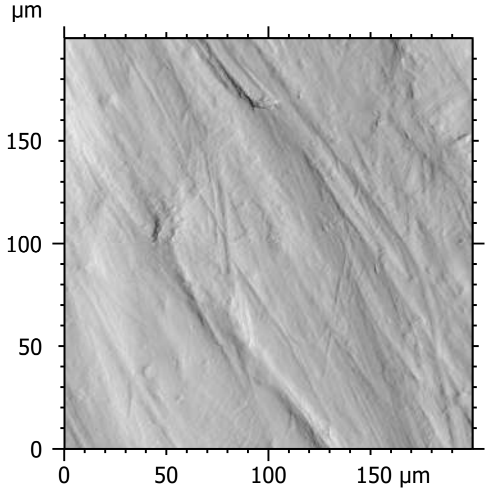

CG-Ce-c36-548-lm-sin-cusp1-bd

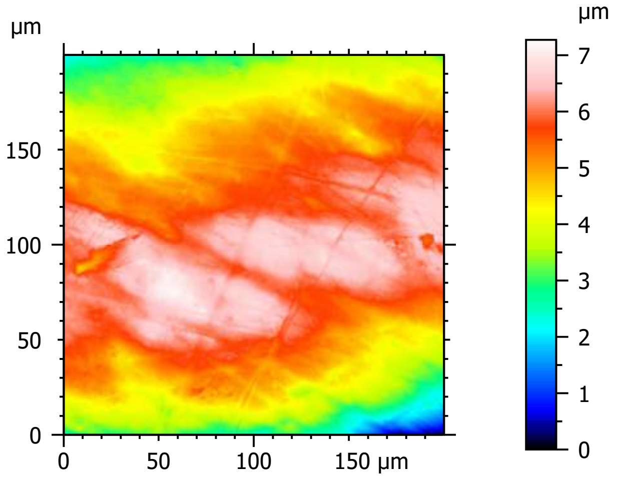

CG-Ce-c36-548-lm-sin-cusp1-bd

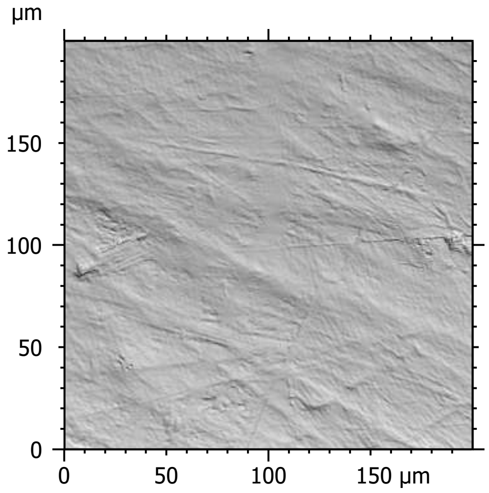

Leica Map Premium 8.2.9843

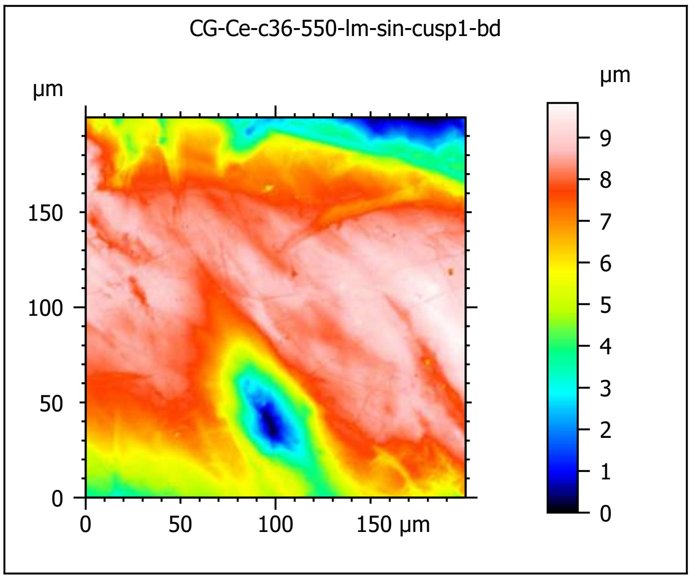

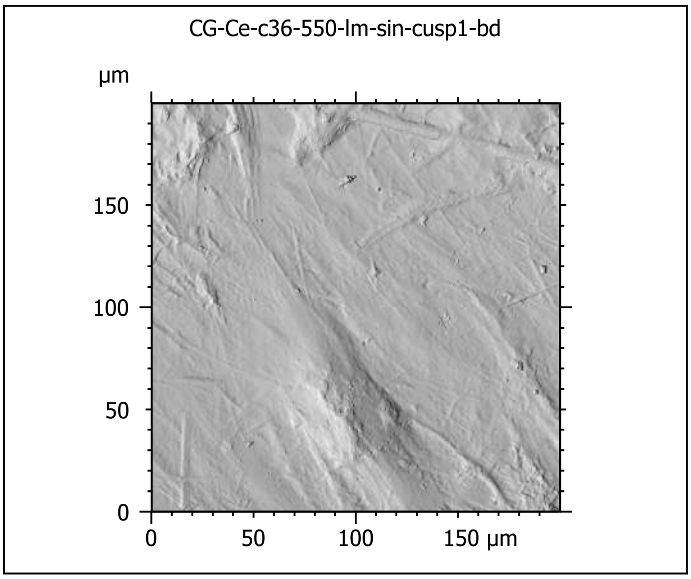

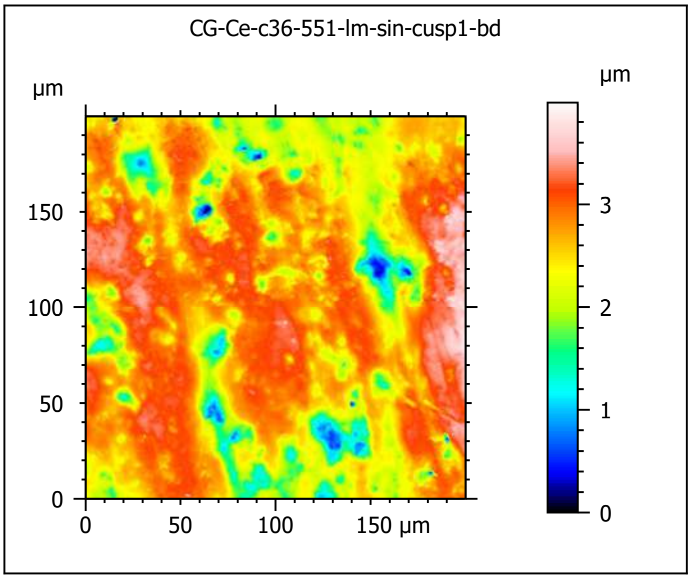

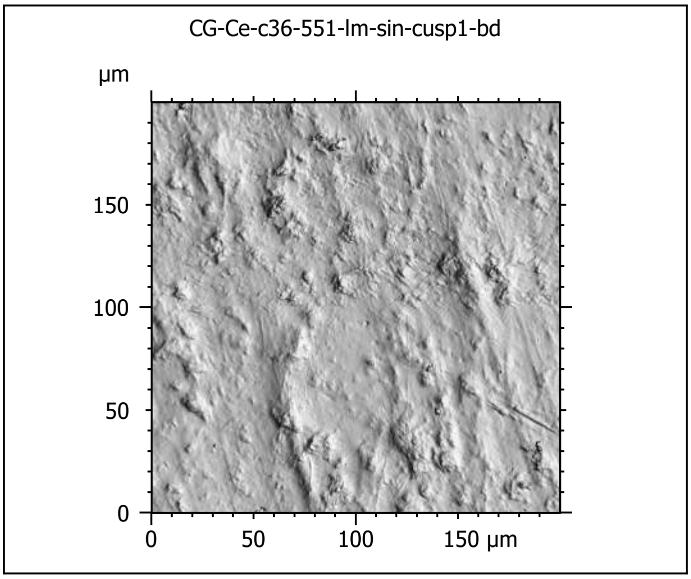

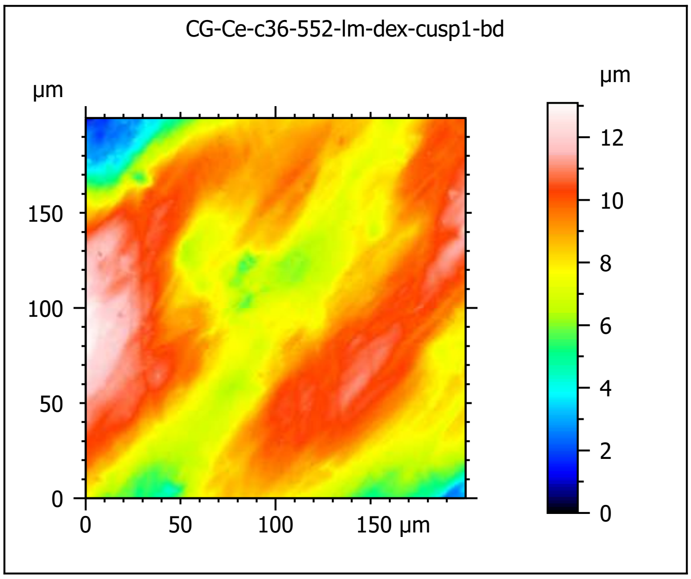

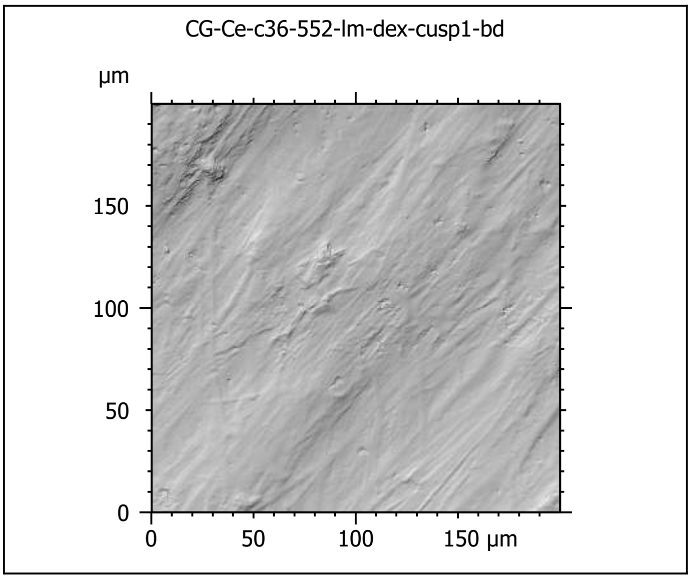

Leica Map Premium 8.2.9843

CG-Ce-c36-553-lm2-sin-cusp1-bd

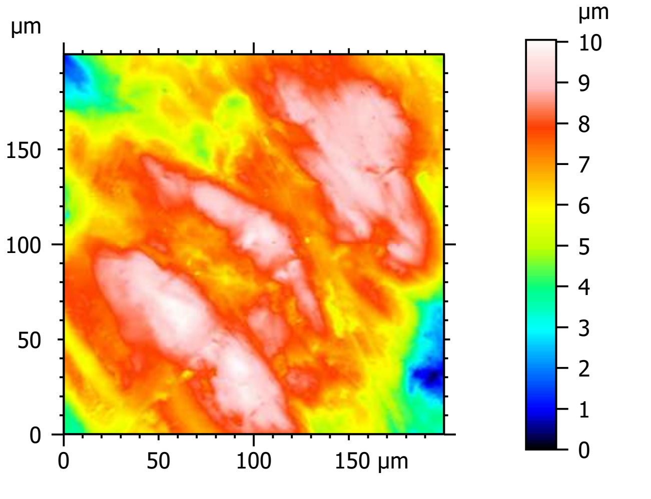

CG-Ce-c36-553-lm2-sin-cusp1-bd

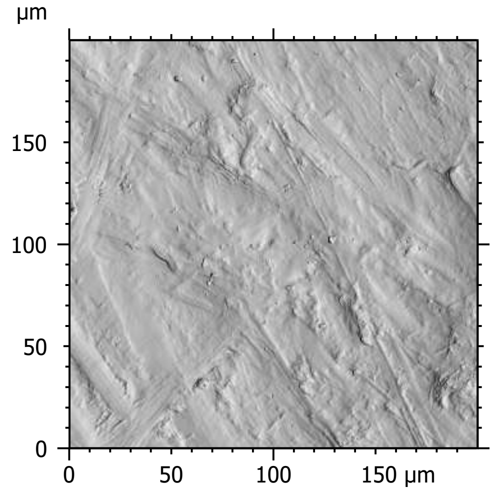

CG-Ce-c36-554-lm1-dex-cusp1-bd

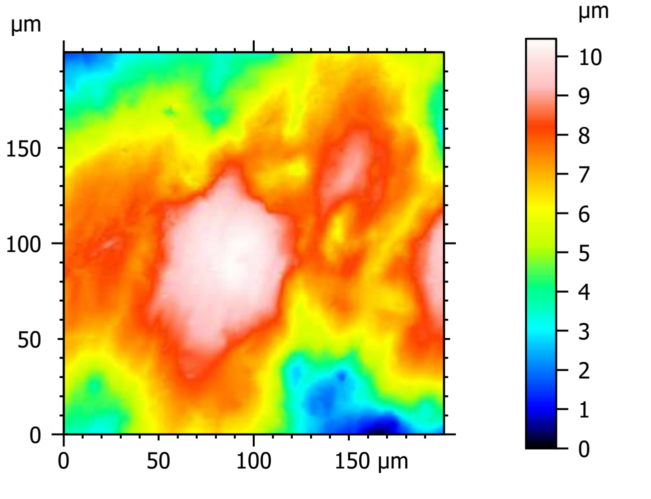

CG-Ce-c36-554-lm1-dex-cusp1-bd

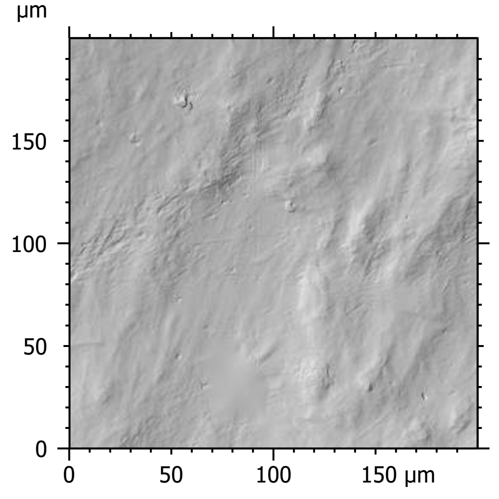

CG-Ce-c36-555-lm2-sin-cusp1-bd

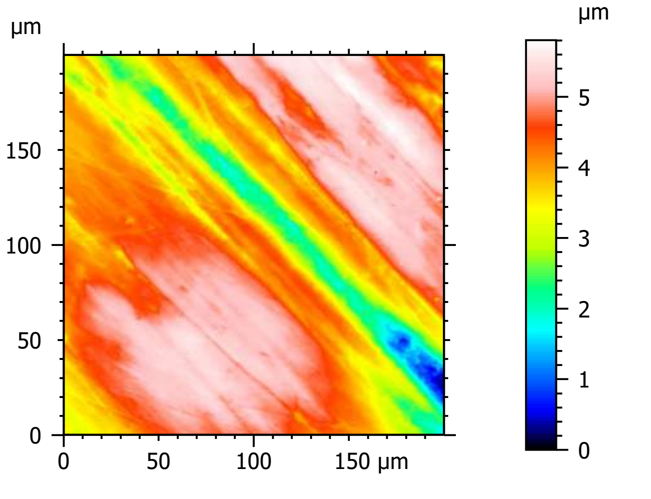

CG-Ce-c36-555-lm2-sin-cusp1-bd

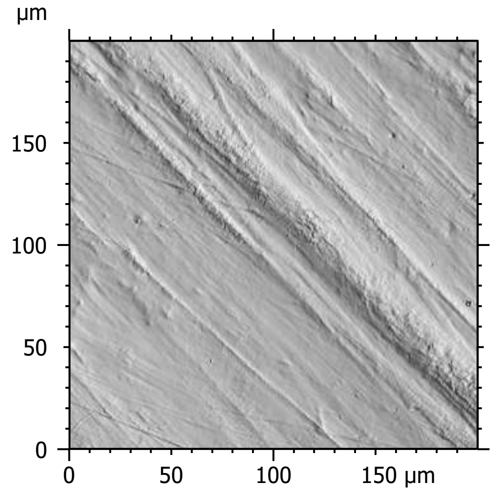

Leica Map Premium 8.2.9843

CG-Ce-c36-556-lm2-dex-cusp2-bd

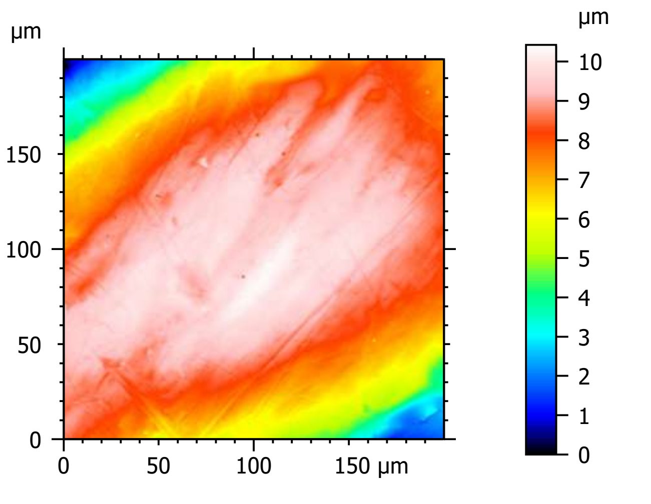

CG-Ce-c36-556-lm2-dex-cusp2-bd

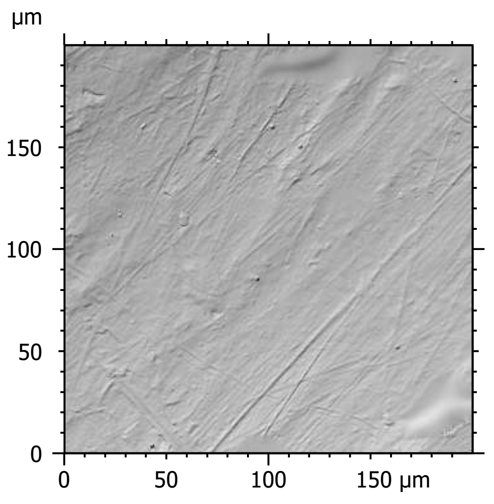

CG-Ce-c36-557-lm2-sin-cusp1-bd

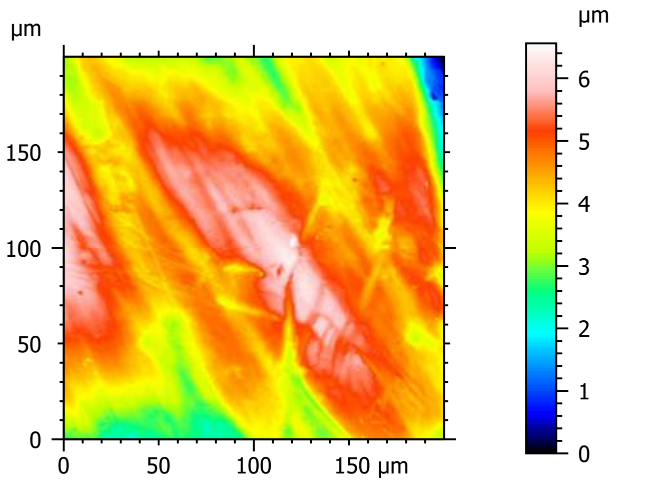

CG-Ce-c36-557-lm2-sin-cusp1-bd

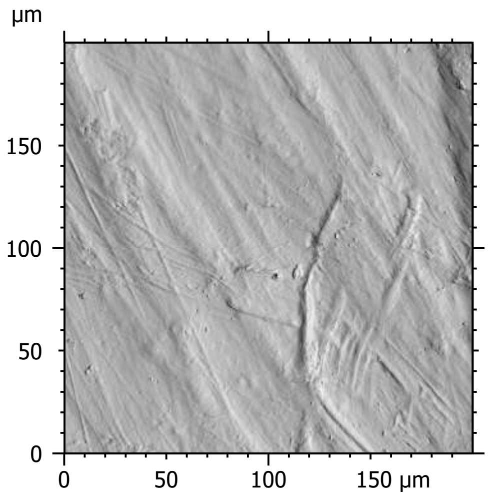

CG-Ce-c36-558-lm2-sin-cusp1-bd

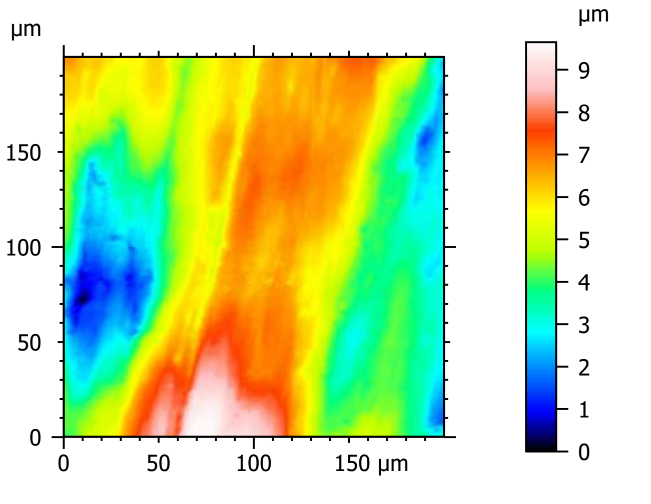

CG-Ce-c36-558-lm2-sin-cusp1-bd

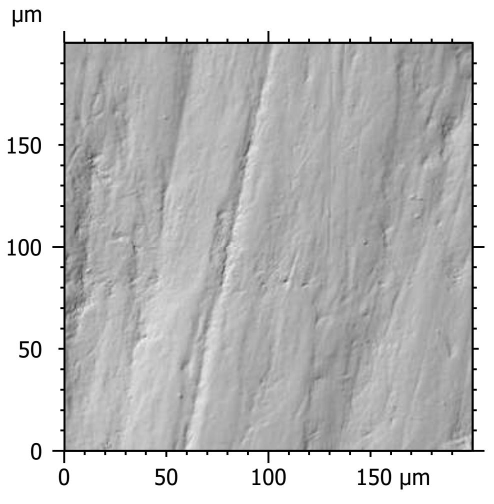

Leica Map Premium 8.2.9843

"A long-term perspective on Neandertal environment and subsistence: insights from the dental micro-texture analysis of hunted ungulates at Combe-Grenal (Dordogne, France)"

authors: Berlioz, E.; Capdepon, E.; Discamps, E.

Appendice 2:
surfaces scanned by E. Berlioz and E. Capdepon, pre-treatement by E. Berlioz and E. Capdepon, validation by E. Berlioz (2019)

***Bos primigenius* / *Bison priscus* - Bock A**

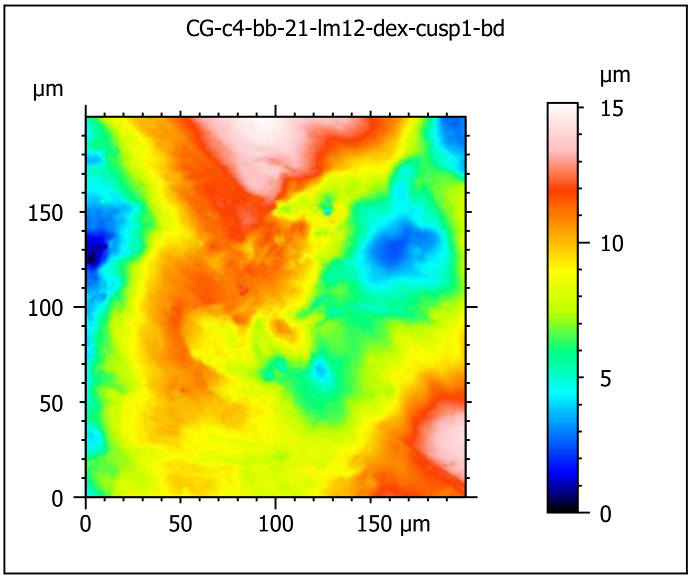

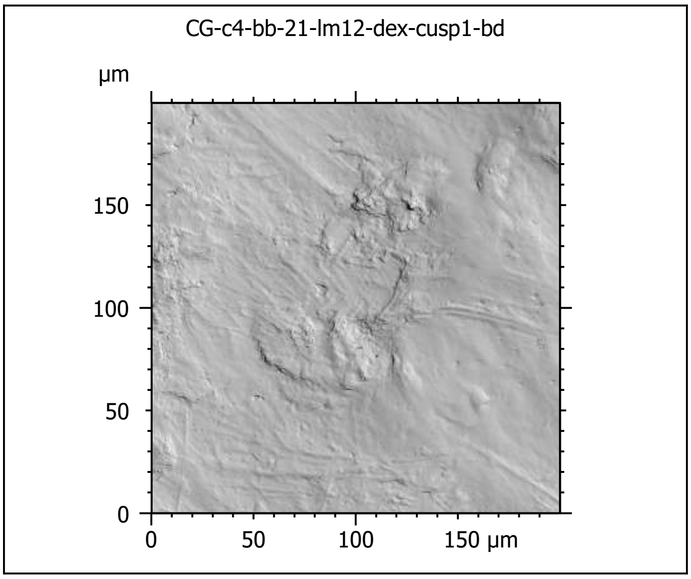

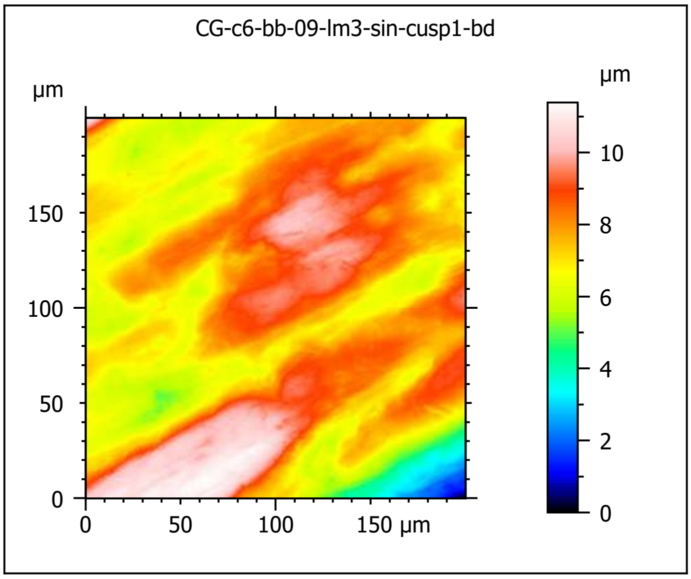

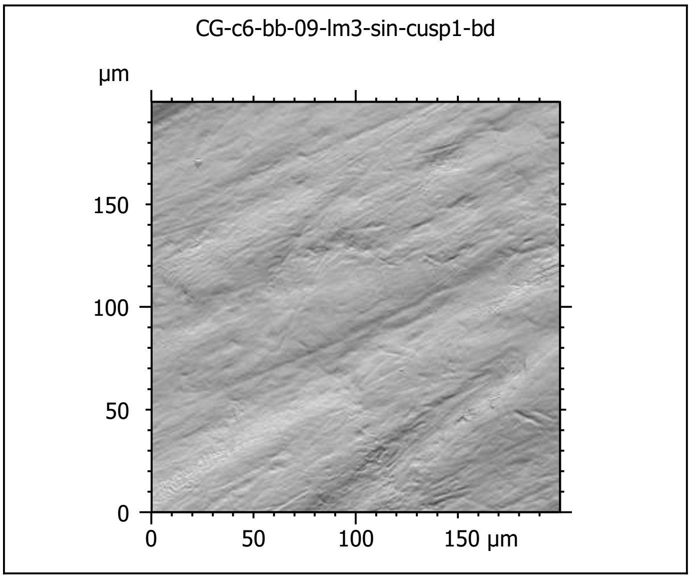

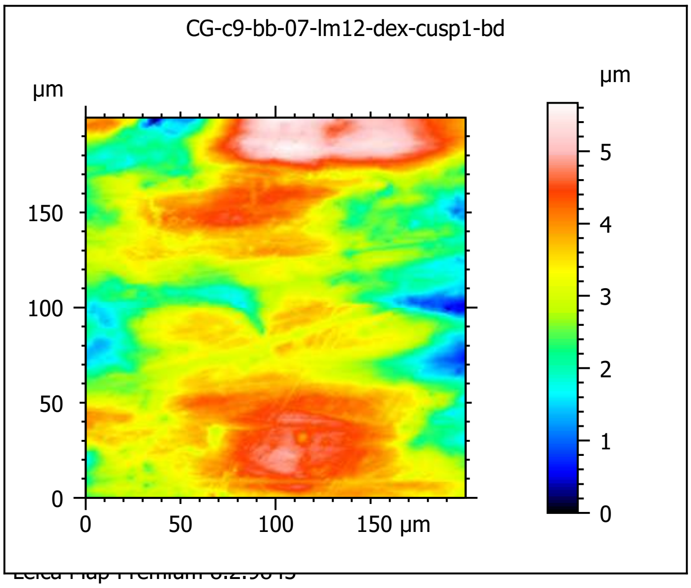

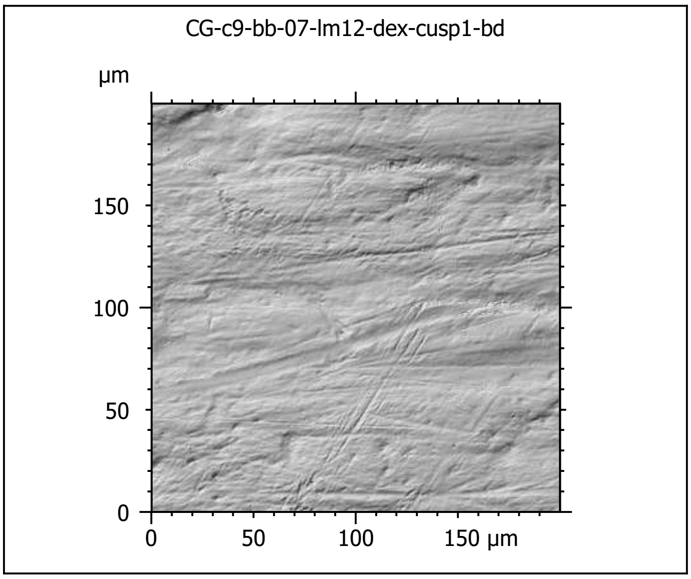

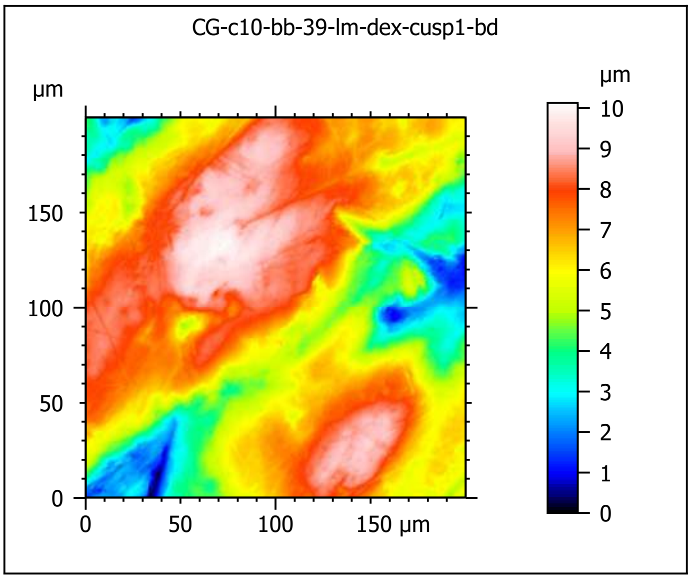

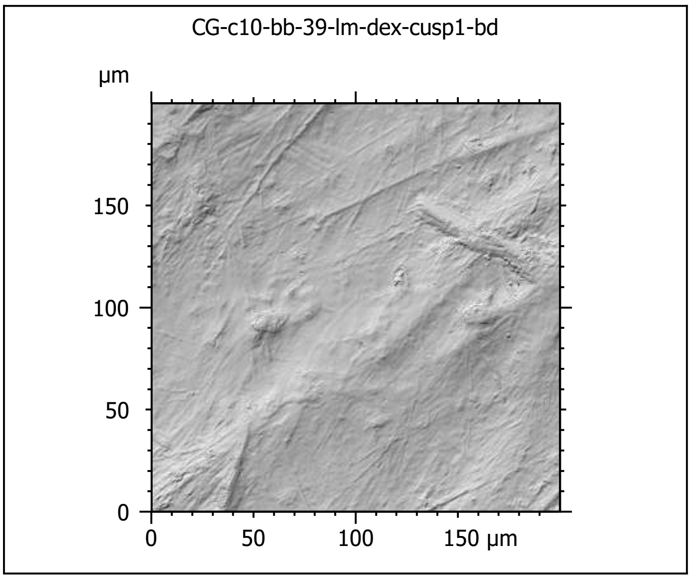

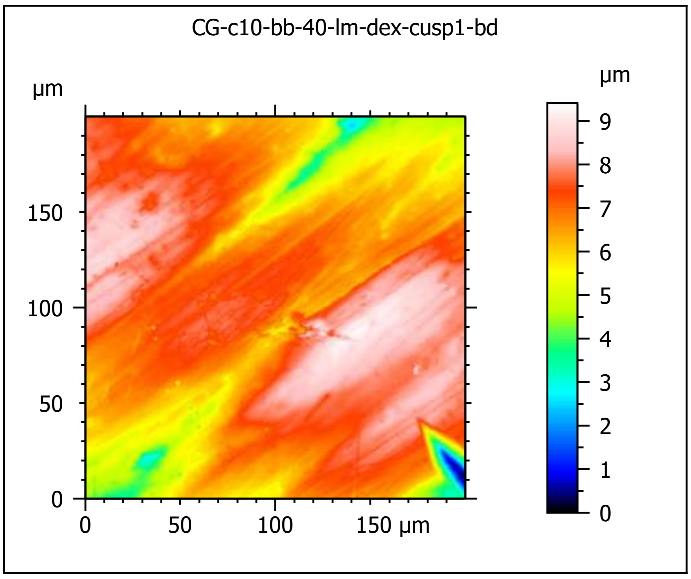

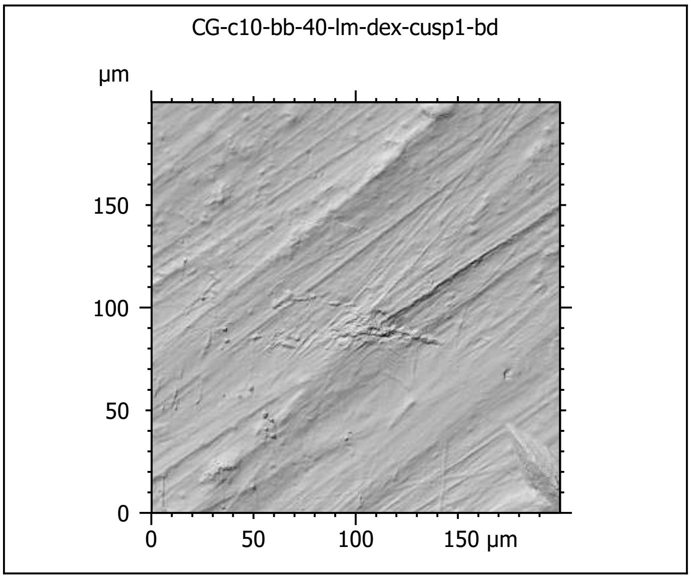

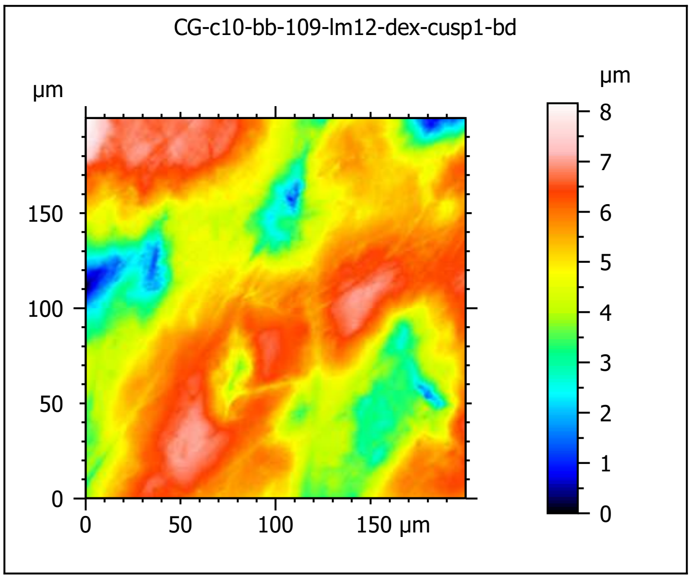

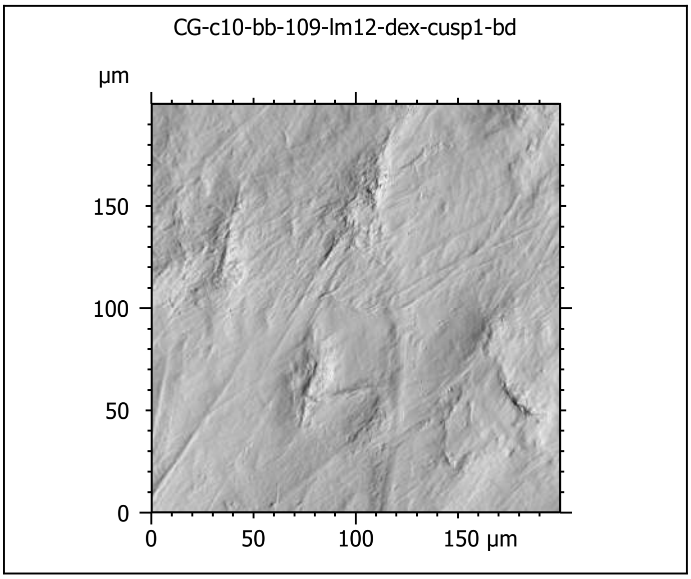

Leica Map Premium 8.2.9843

CG-c10-bb-111-lm12-dex-cusp1-bd

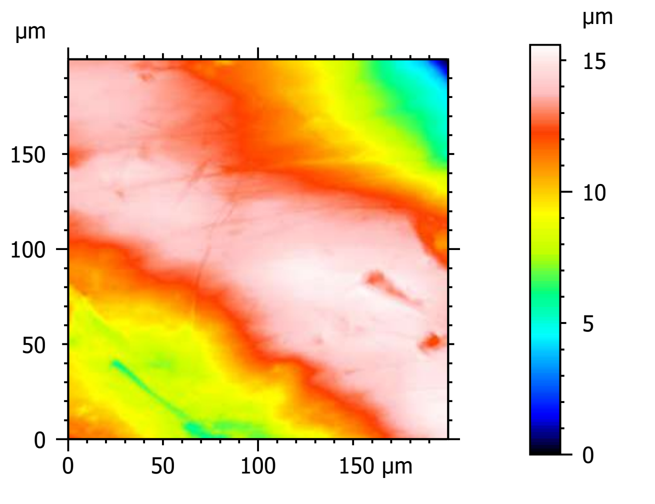

CG-c10-bb-111-lm12-dex-cusp1-bd

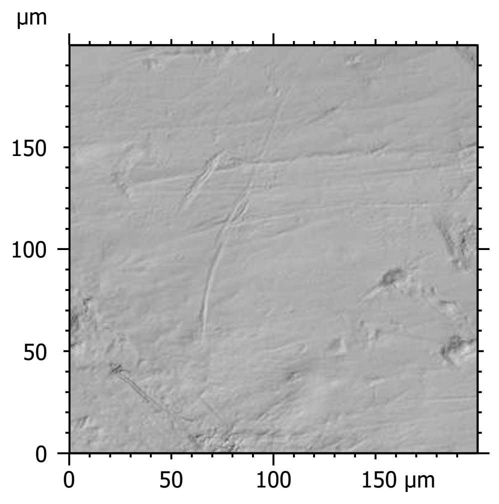

CG-c10-bb-112-lm3-dex-cusp1-bd

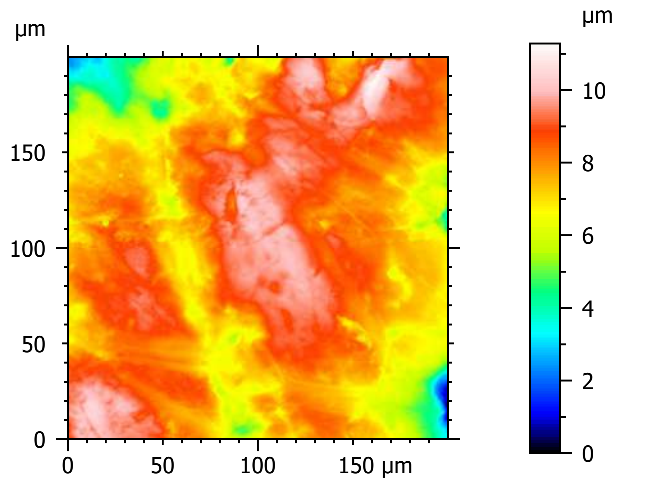

CG-c10-bb-112-lm3-dex-cusp1-bd

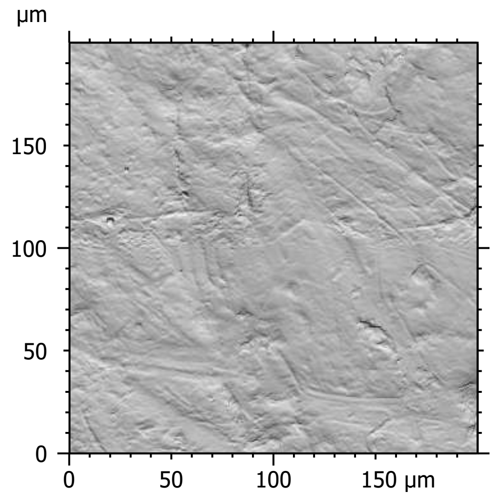

CG-c10-bb-113-lm3-sin-cusp1-bd

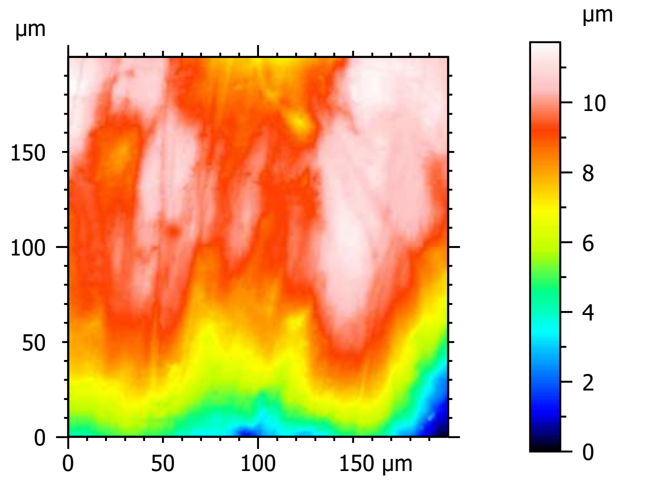

CG-c10-bb-113-lm3-sin-cusp1-bd

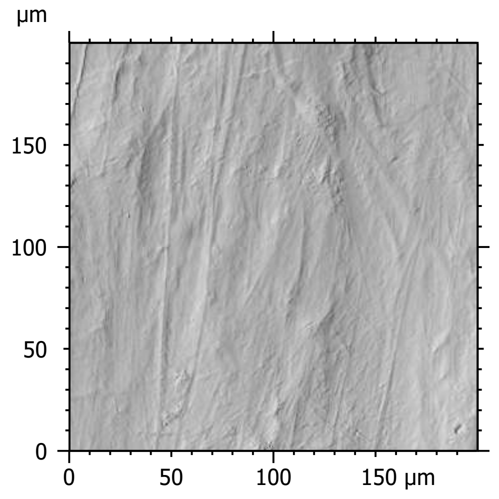

Leica Map Premium 8.2.9843

"A long-term perspective on Neandertal environment and subsistence: insights from the dental micro-texture analysis of hunted ungulates at Combe-Grenal (Dordogne, France)"

authors: Berlioz, E.; Capdepon, E.; Discamps, E.

Appendice 2:
surfaces scanned by E. Berlioz and E. Capdepon, pre-treatement by E. Berlioz and E. Capdepon, validation by E. Berlioz (2019)

***Bos primigenius* / *Bison priscus* - Bock B**

CG-c11-bb-43-lm12-sin-cusp1-bd

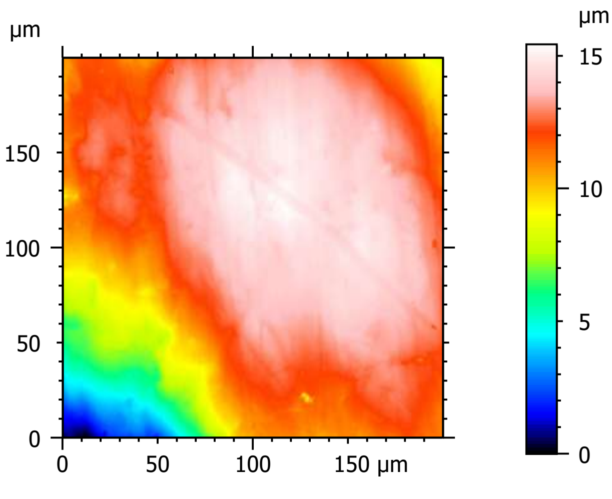

CG-c11-bb-43-lm12-sin-cusp1-bd

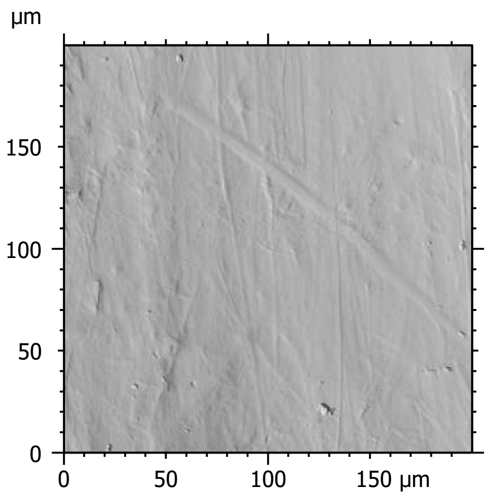

CG-c11-bb-45-lm-dex-cusp1-bd

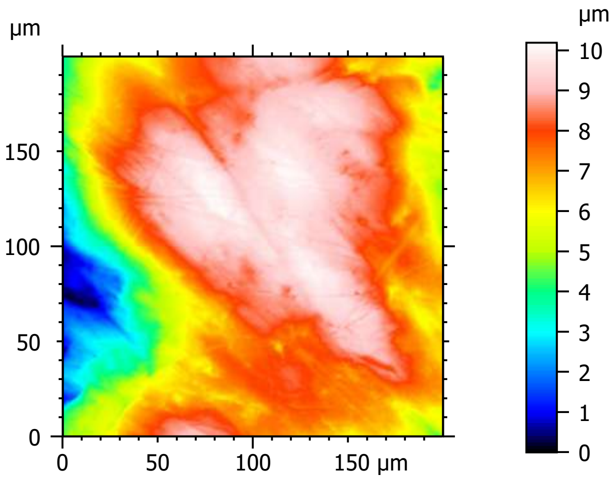

CG-c11-bb-45-lm-dex-cusp1-bd

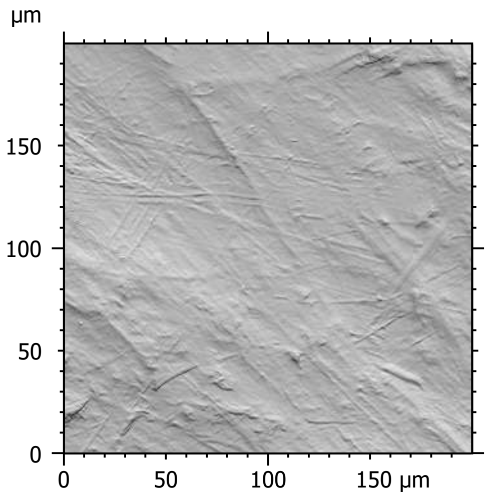

CG-c11-bb-46-lm-sin-cusp1-bd

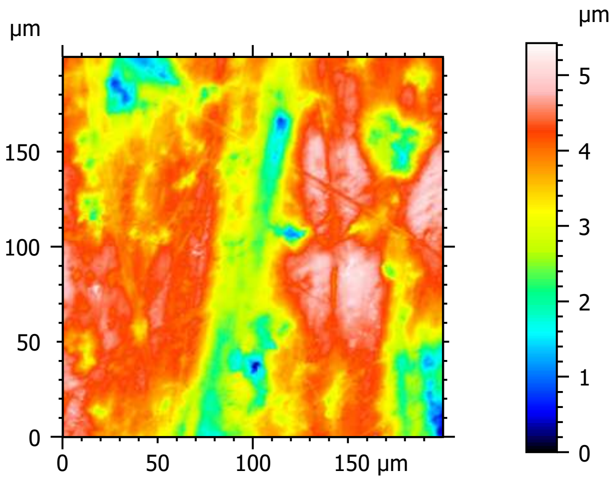

CG-c11-bb-46-lm-sin-cusp1-bd

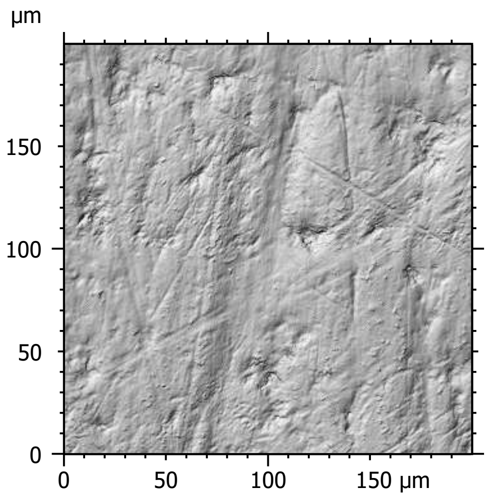

CG-c11-bb-47-lm12-sin-cusp1-bd

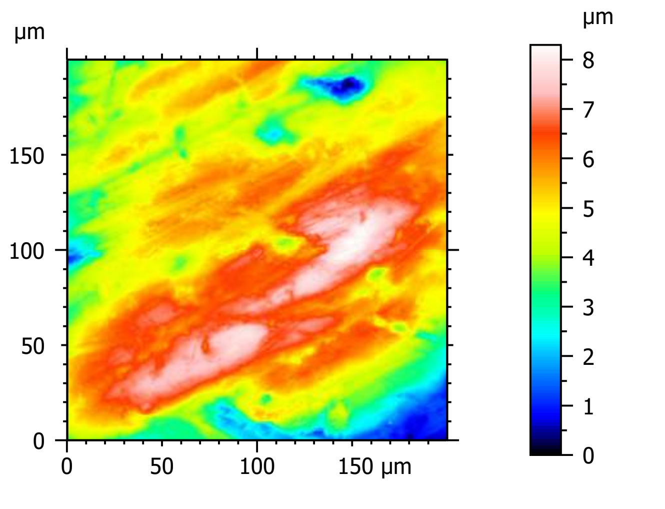

CG-c11-bb-47-lm12-sin-cusp1-bd

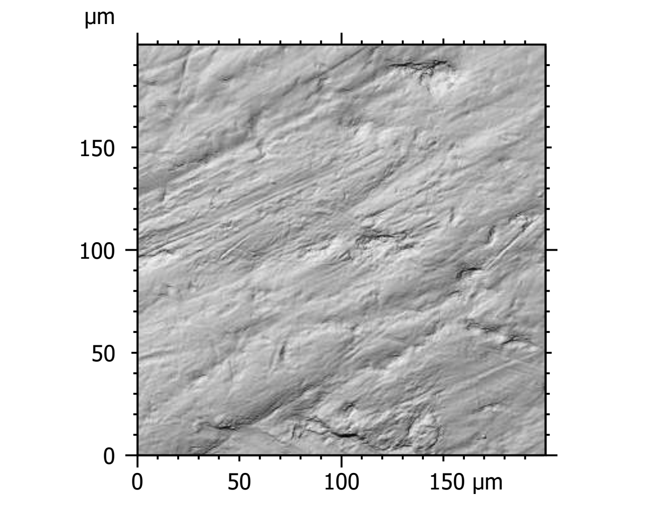

CG-c11-bb-48-lm3-sin-cusp1-bd

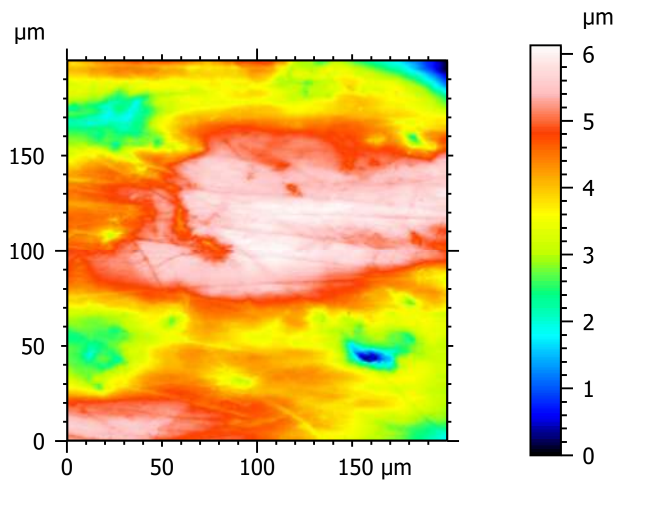

CG-c11-bb-48-lm3-sin-cusp1-bd

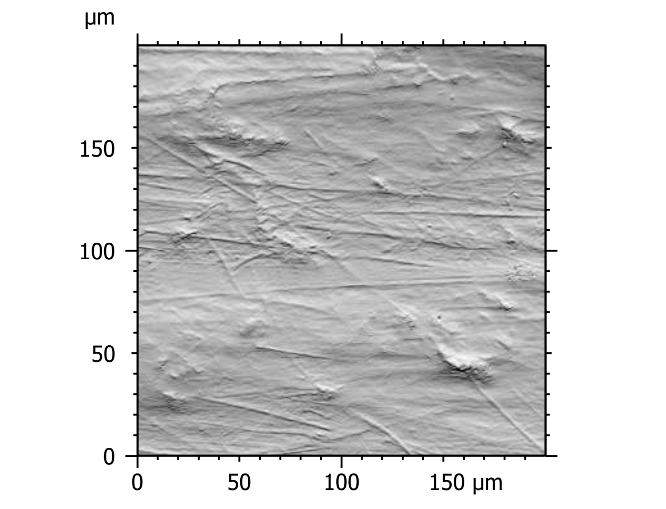

CG-c11-bb-49-lm3-sin-cusp1-bd

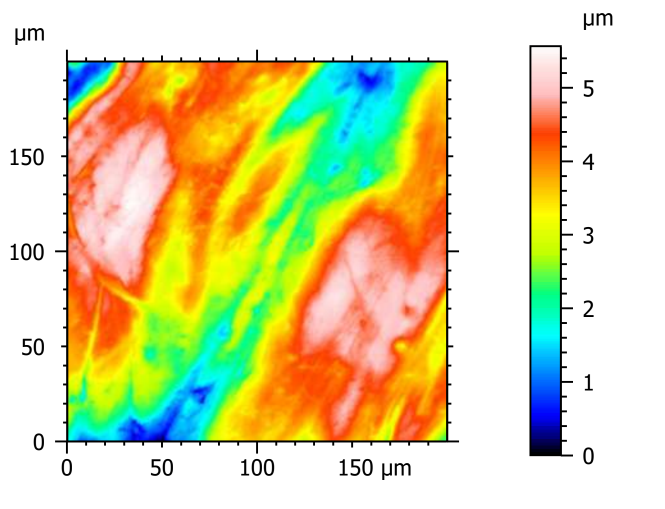

CG-c11-bb-49-lm3-sin-cusp1-bd

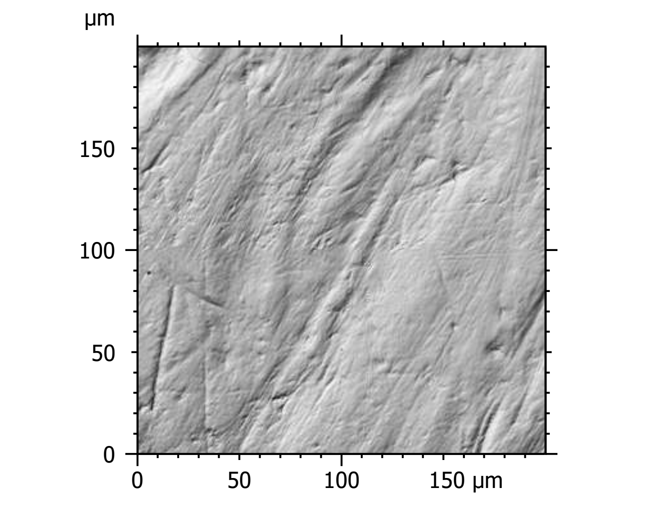

Leica Map Premium 8.2.9843

CG-c11-bb-51-lm12-sin-cusp1-bd

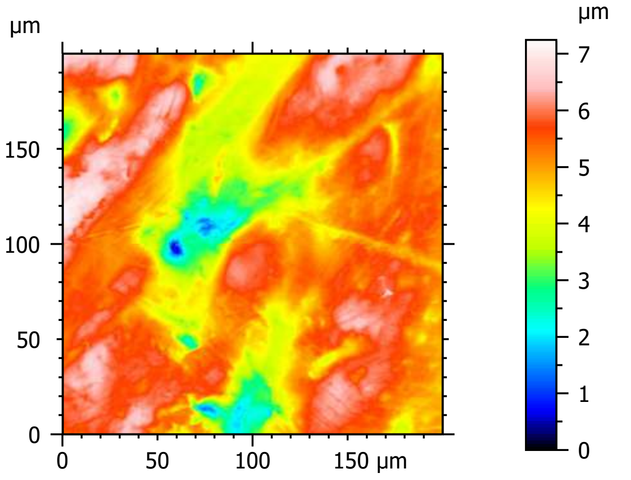

CG-c11-bb-51-lm12-sin-cusp1-bd

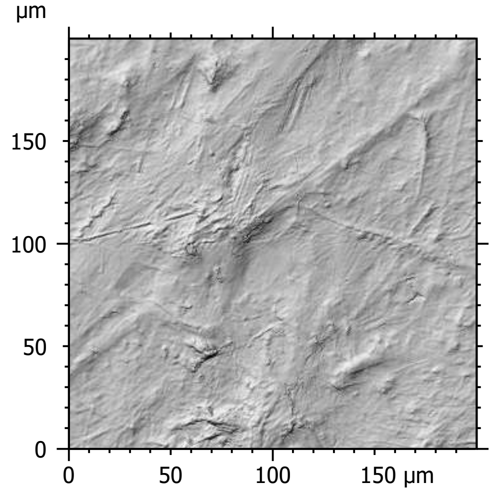

CG-c11-bb-52-lm12-sin-cusp1-bd

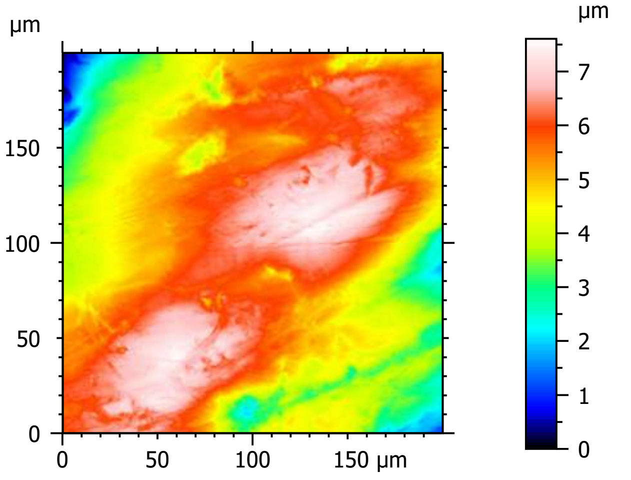

CG-c11-bb-52-lm12-sin-cusp1-bd

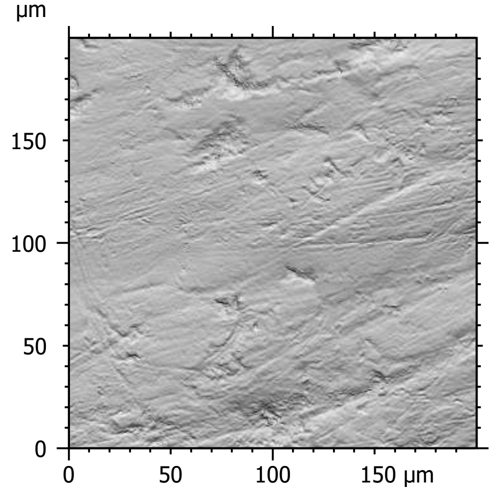

CG-c11-bb-53-lm12-sin-cusp1-bd

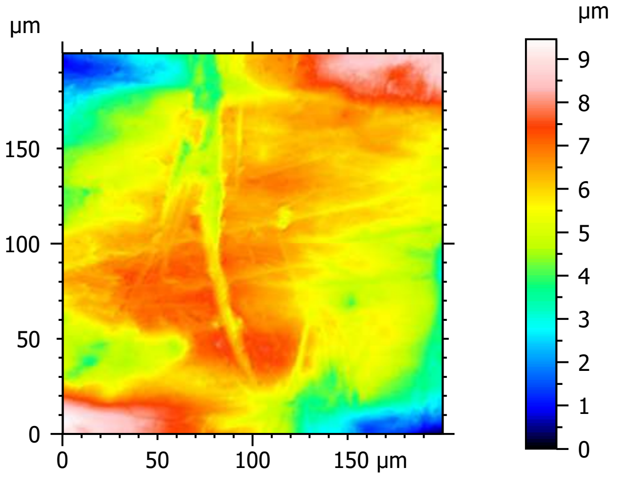

CG-c11-bb-53-lm12-sin-cusp1-bd

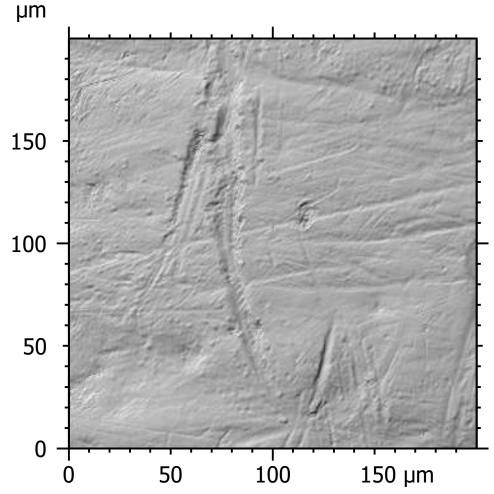

CG-c11-bb-55-lm-dex-cusp1-bd

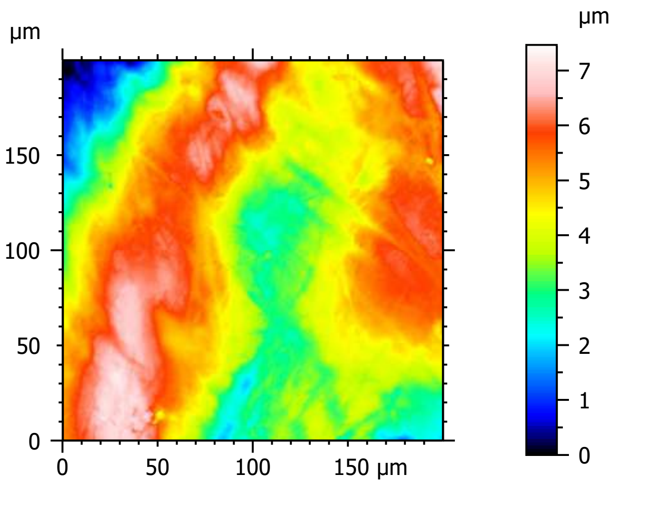

CG-c11-bb-55-lm-dex-cusp1-bd

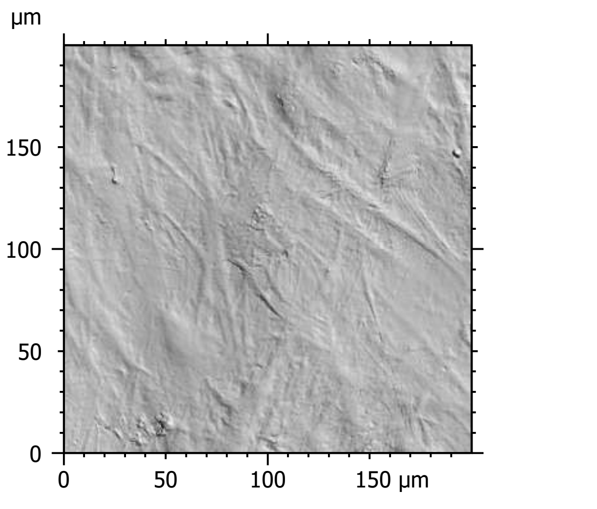

CG-c11-bb-57-lm3-dex-cusp1-bd

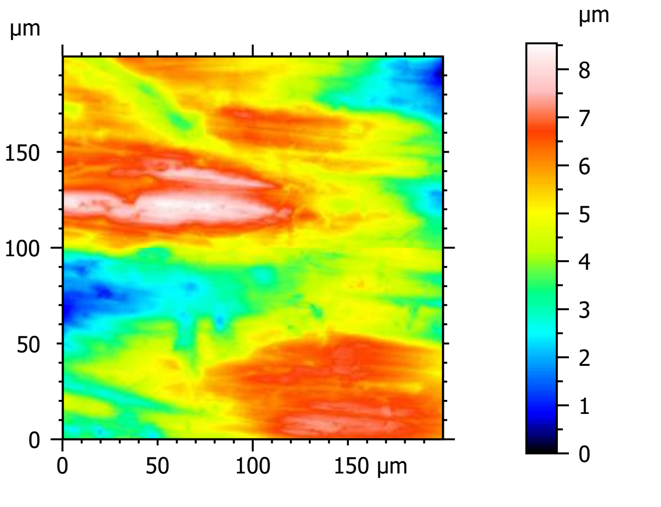

CG-c11-bb-57-lm3-dex-cusp1-bd

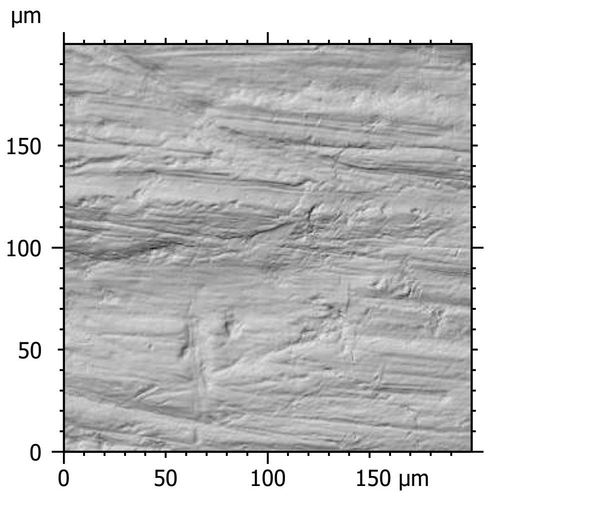

CG-c11-bb-59-lm3-dex-cusp1-bd

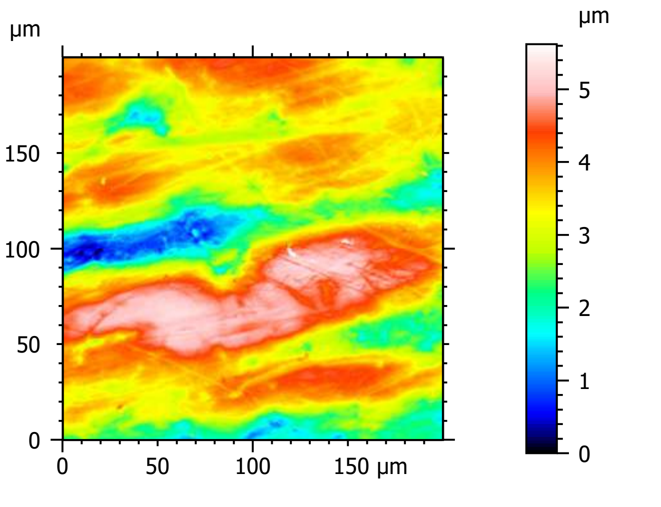

CG-c11-bb-59-lm3-dex-cusp1-bd

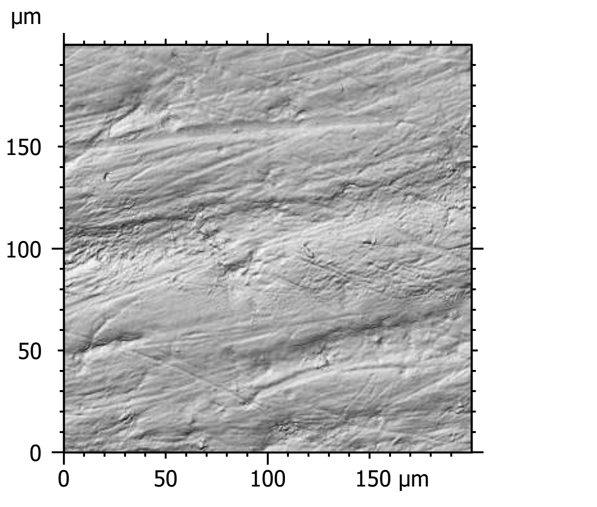

Leica Map Premium 8.2.9843

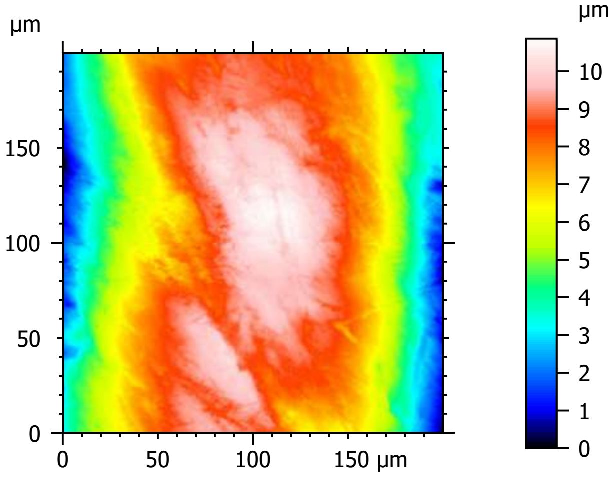

CG-c11-bb-60-lm12-dex-cusp1-bd

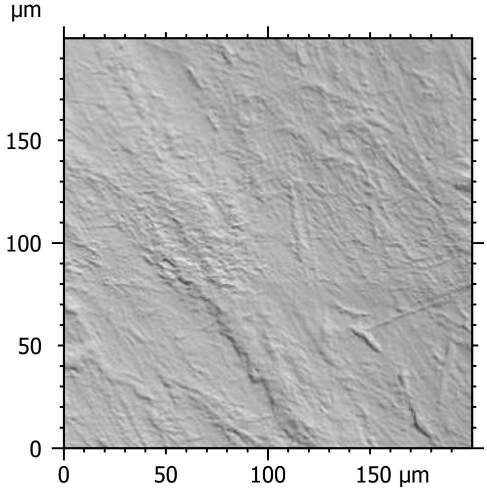

CG-c11-bb-60-lm12-dex-cusp1-bd

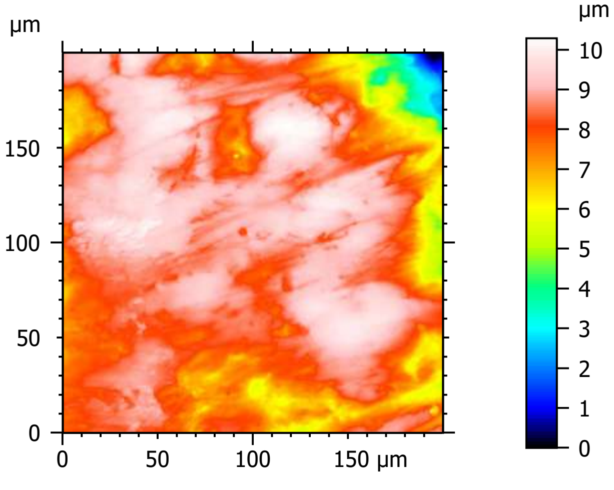

CG-c11-bb-62-lm12-dex-cusp1-bd

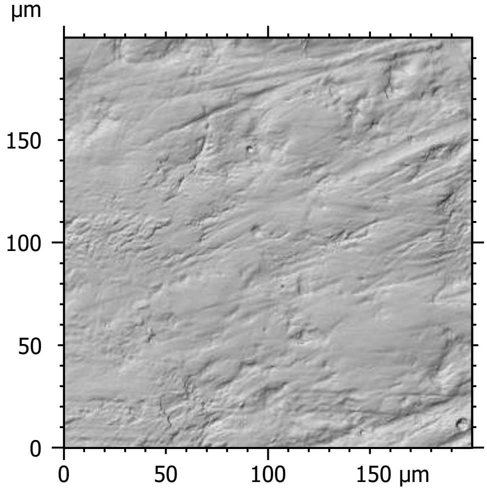

CG-c11-bb-62-lm12-dex-cusp1-bd

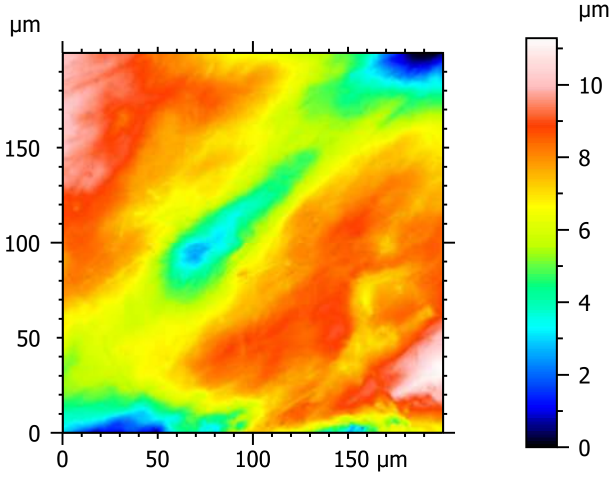

CG-c11-bb-63-lm12-dex-cusp1-bd

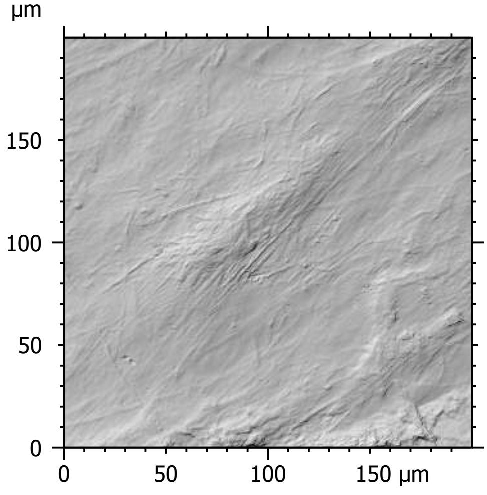

CG-c11-bb-63-lm12-dex-cusp1-bd

Leica Map Premium 8.2.9843

CG-c11-bb-64-lm12-dex-cusp1-bd

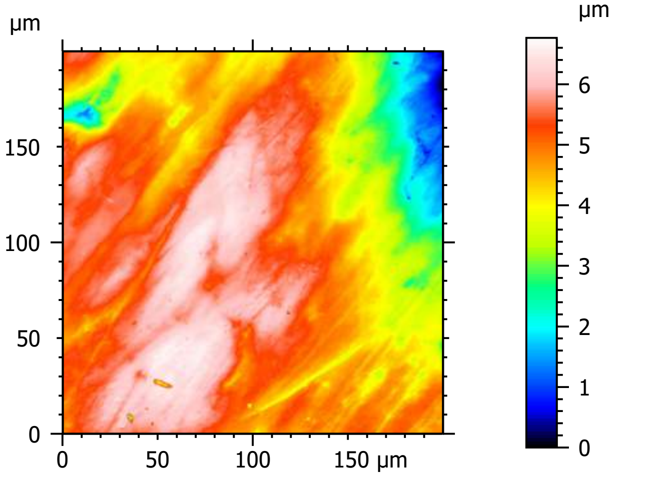

CG-c11-bb-64-lm12-dex-cusp1-bd

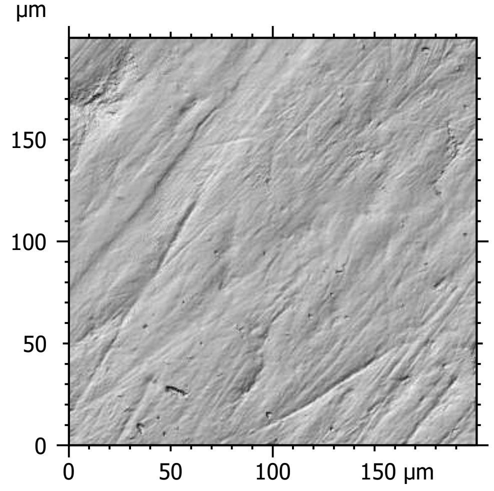

CG-c12-bb-102-lm3-sin-cusp1-bd

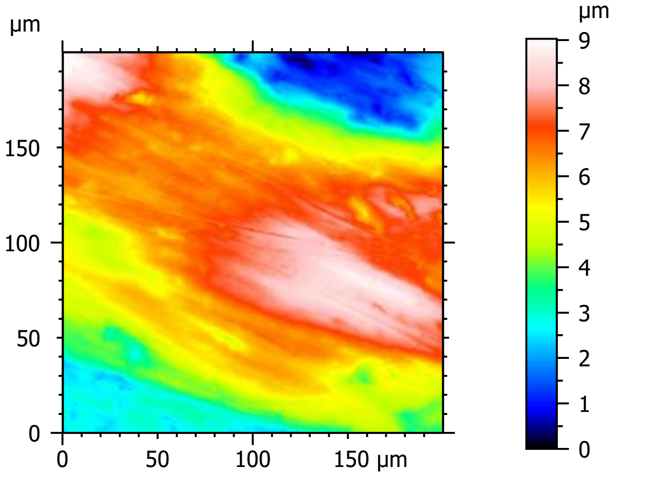

CG-c12-bb-102-lm3-sin-cusp1-bd

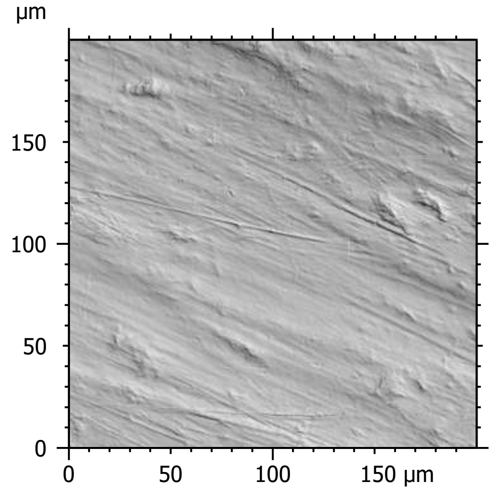

CG-c12-bb-103-lm12-sin-cusp1-bd

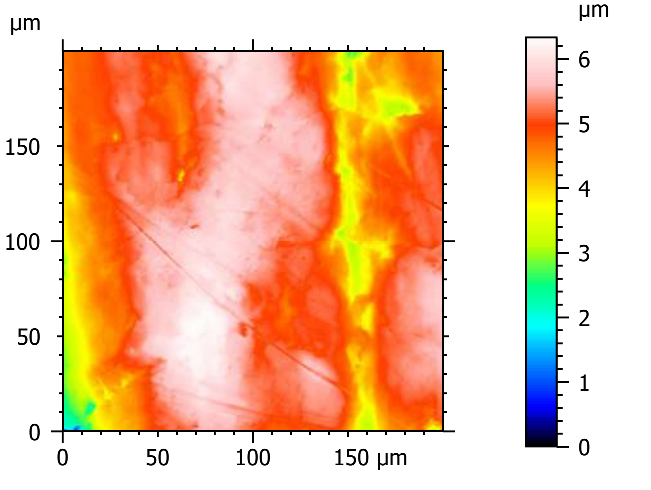

CG-c12-bb-103-lm12-sin-cusp1-bd

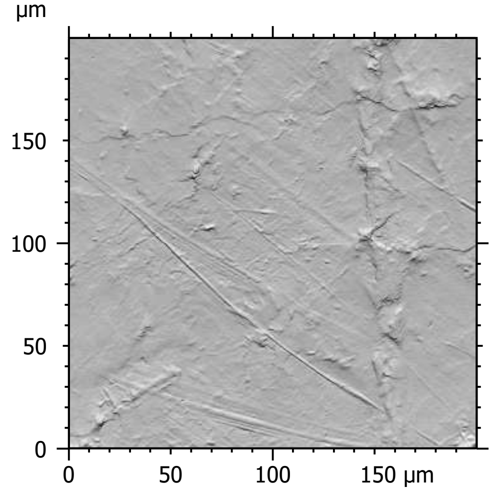

Leica Map Premium 8.2.9843

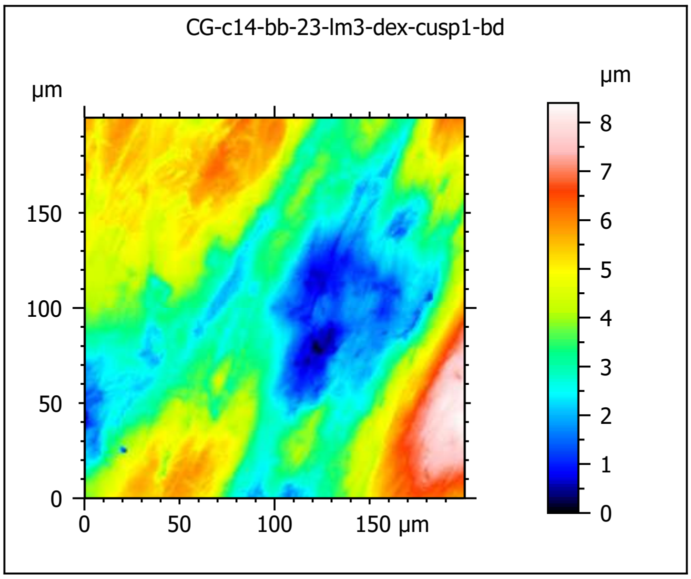

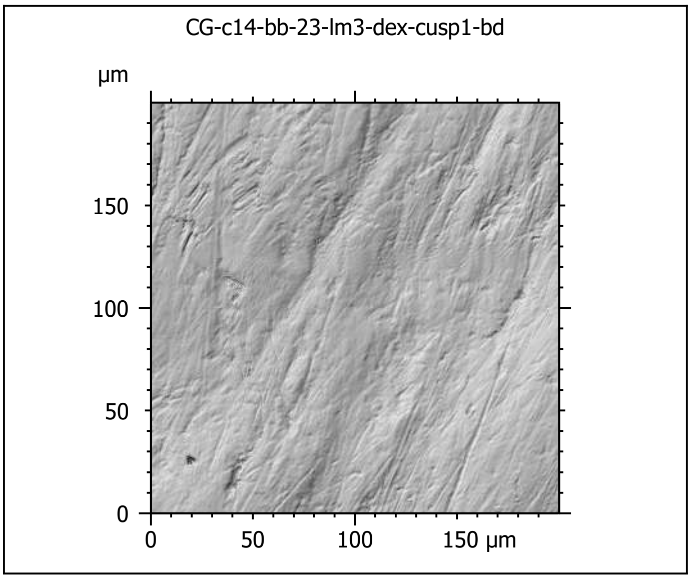

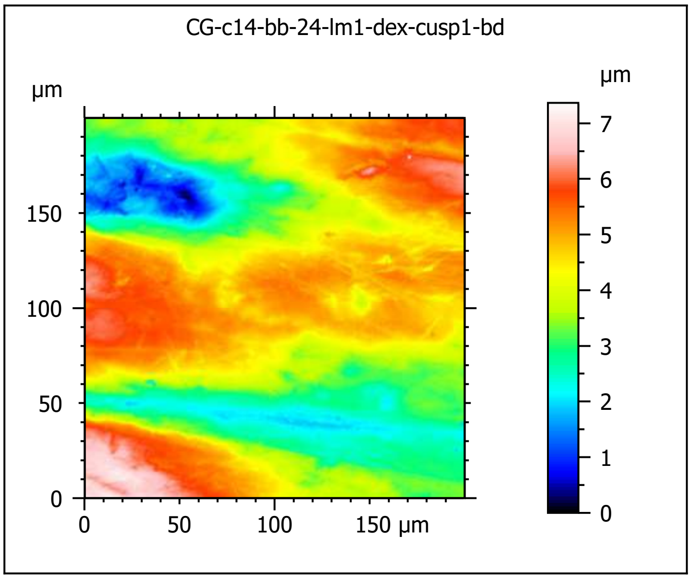

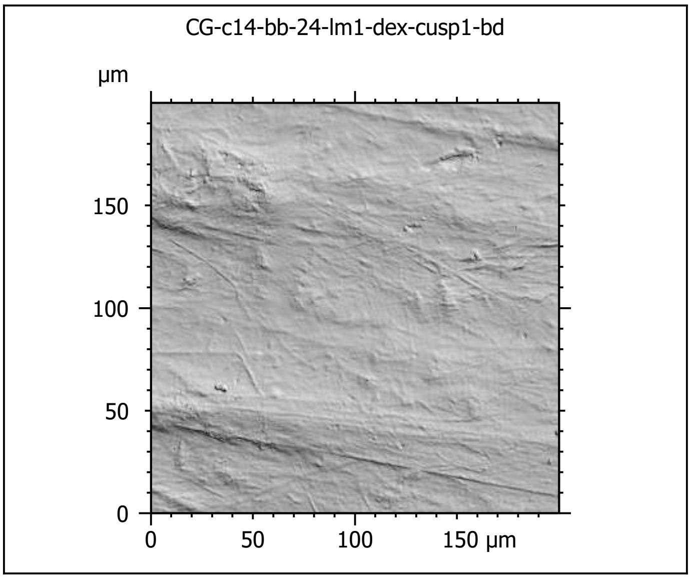

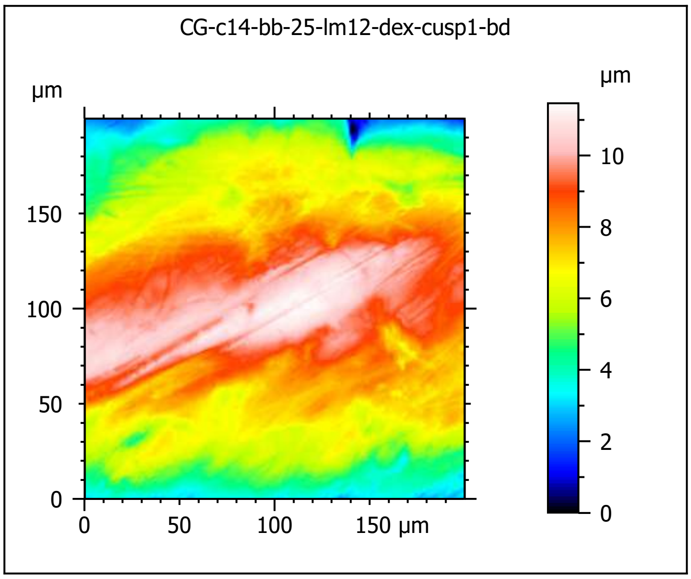

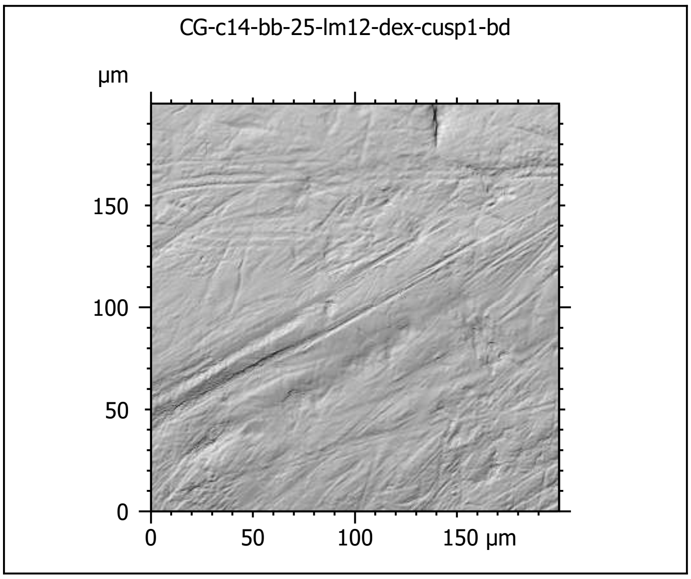

Leica Map Premium 8.2.9843

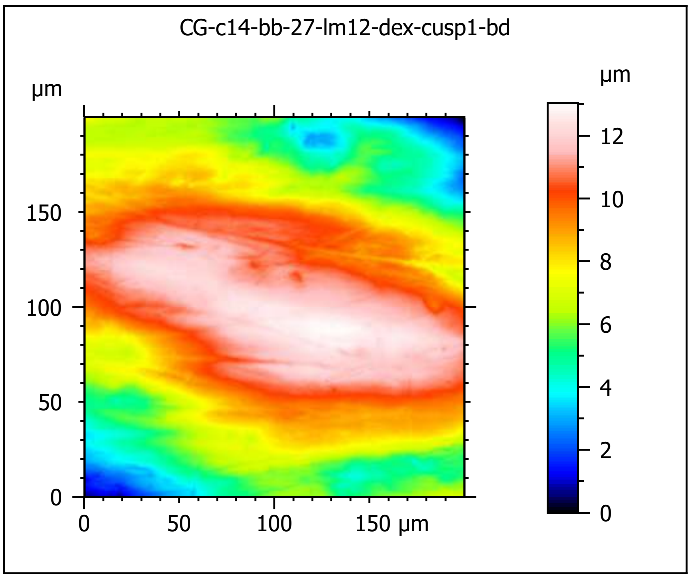

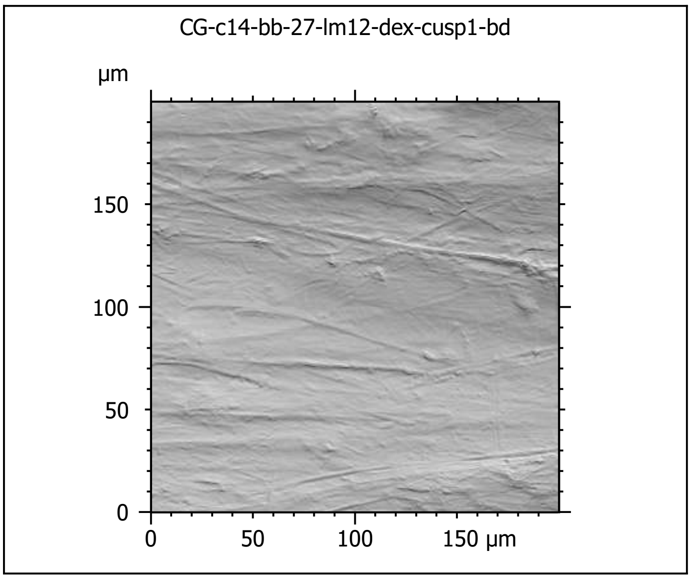

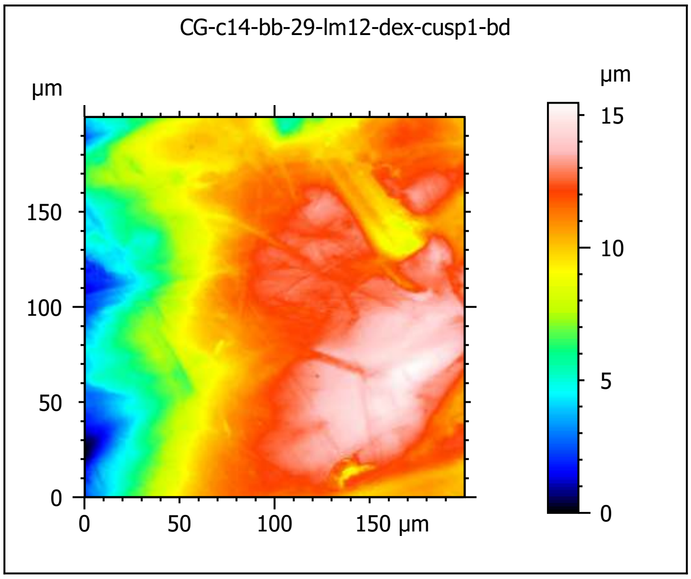

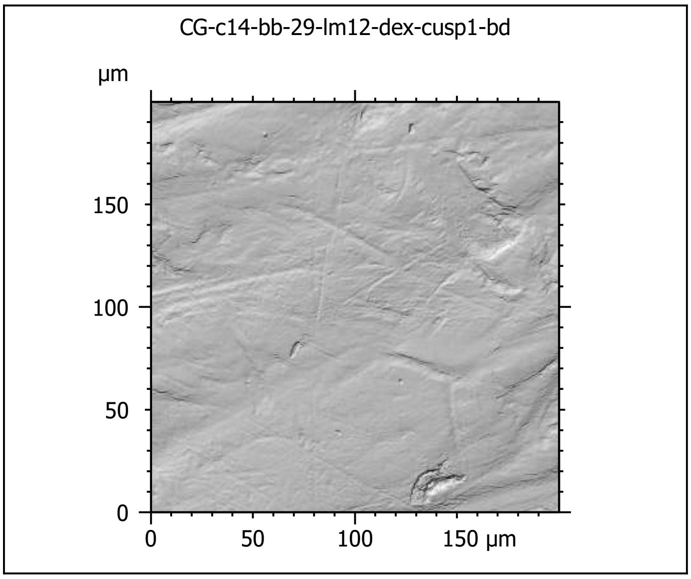

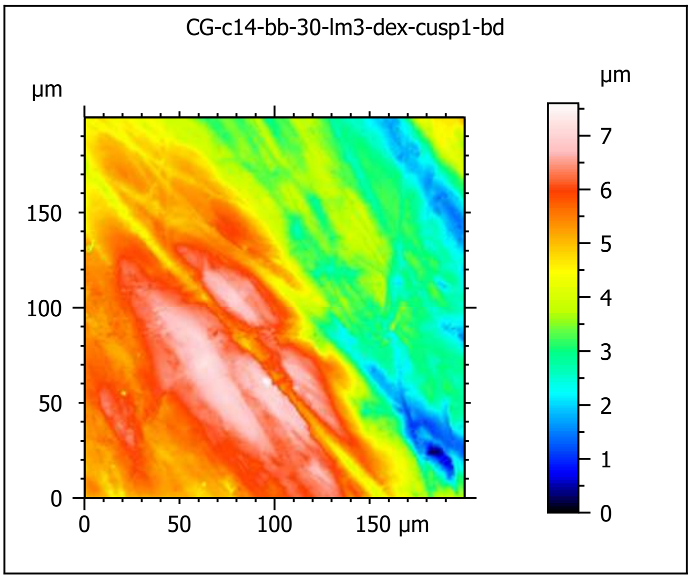

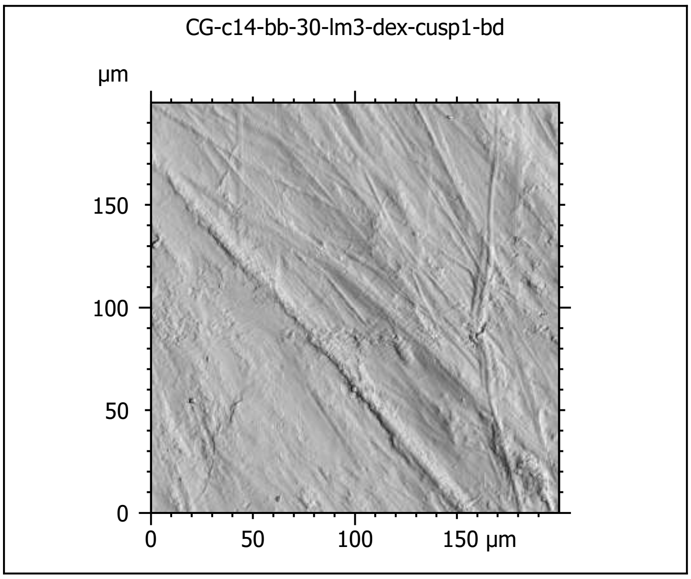

Leica Map Premium 8.2.9843

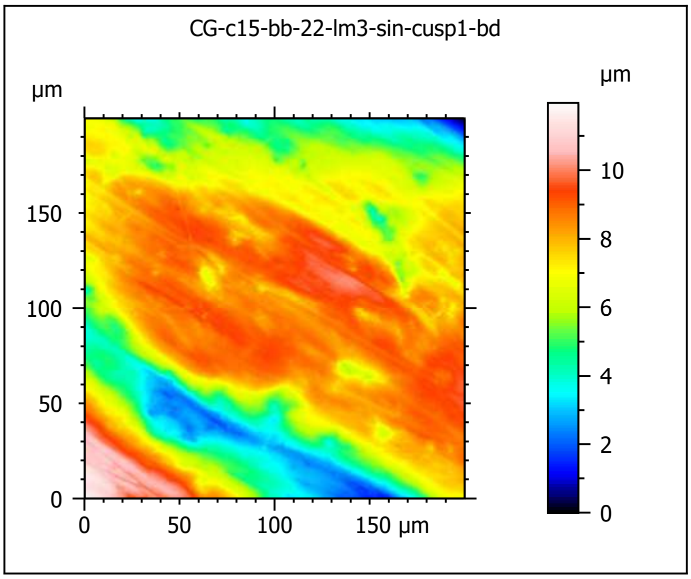

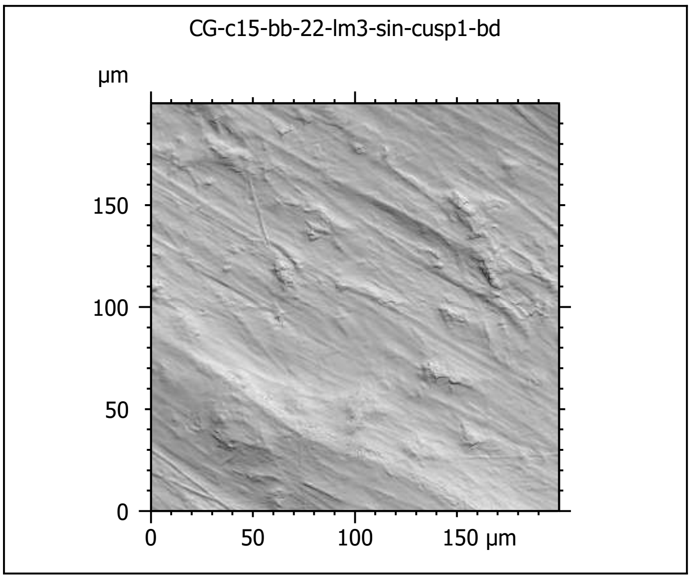

Leica Map Premium 8.2.9843

"A long-term perspective on Neandertal environment and subsistence: insights from the dental micro-texture analysis of hunted ungulates at Combe-Grenal (Dordogne, France)"

authors: Berlioz, E.; Capdepon, E.; Discamps, E.

Appendice 2:
surfaces scanned by E. Berlioz and E. Capdepon, pre-treatement by E. Berlioz and E. Capdepon, validation by E. Berlioz (2019)

***Bos primigenius* / *Bison priscus* - Bock E**

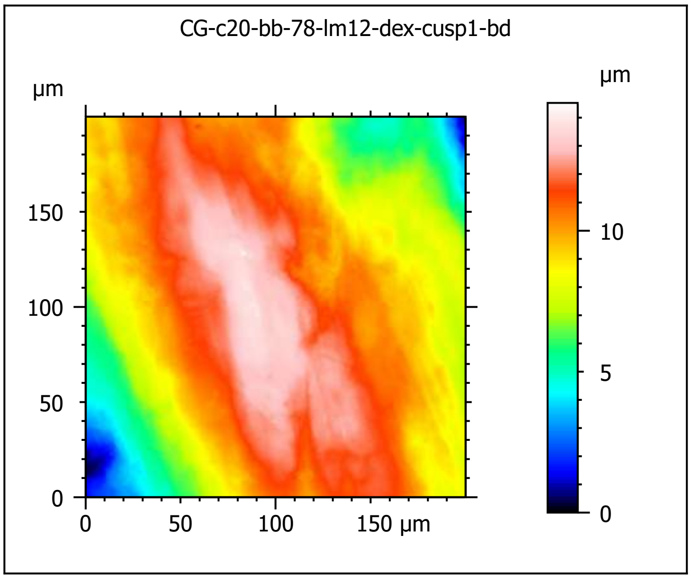

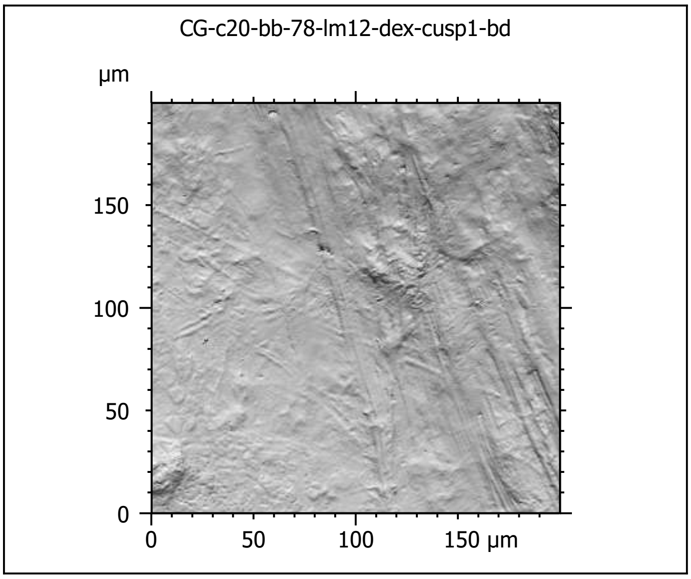

Leica Map Premium 8.2.9843

"A long-term perspective on Neandertal environment and subsistence: insights from the dental micro-texture analysis of hunted ungulates at Combe-Grenal (Dordogne, France)"

authors: Berlioz, E.; Capdepon, E.; Discamps, E.

Appendice 2:
surfaces scanned by E. Berlioz and E. Capdepon, pre-treatement by E. Berlioz and E. Capdepon, validation by E. Berlioz (2019)

***Bos primigenius* / *Bison priscus* - Bock F**

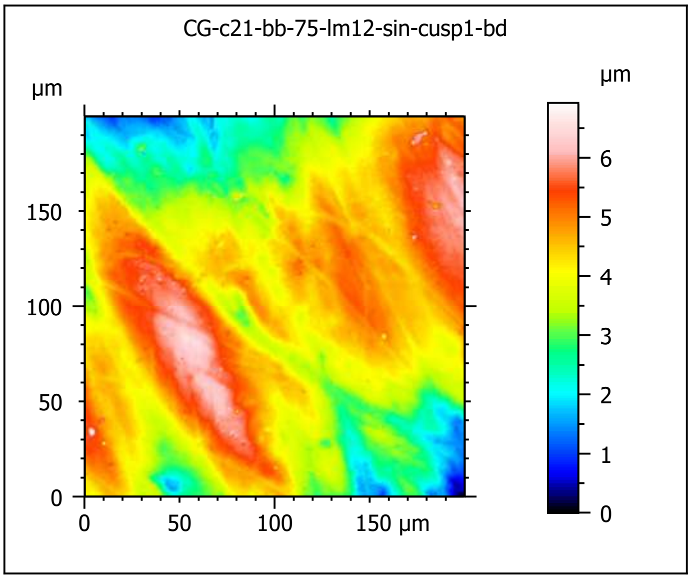

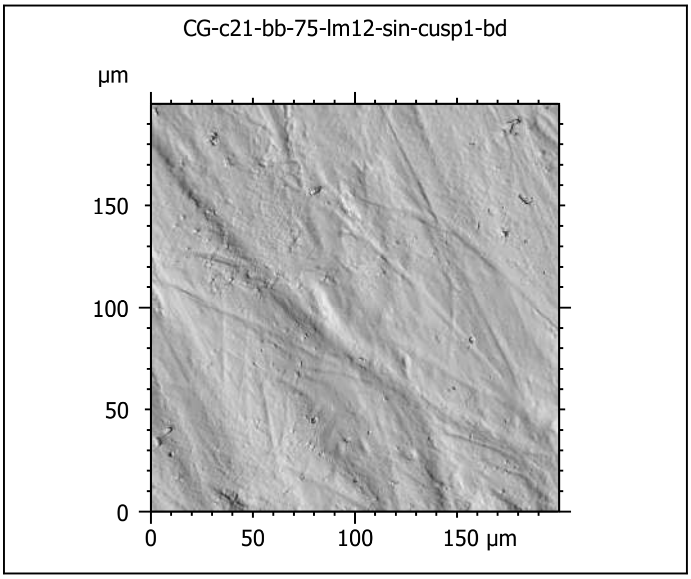

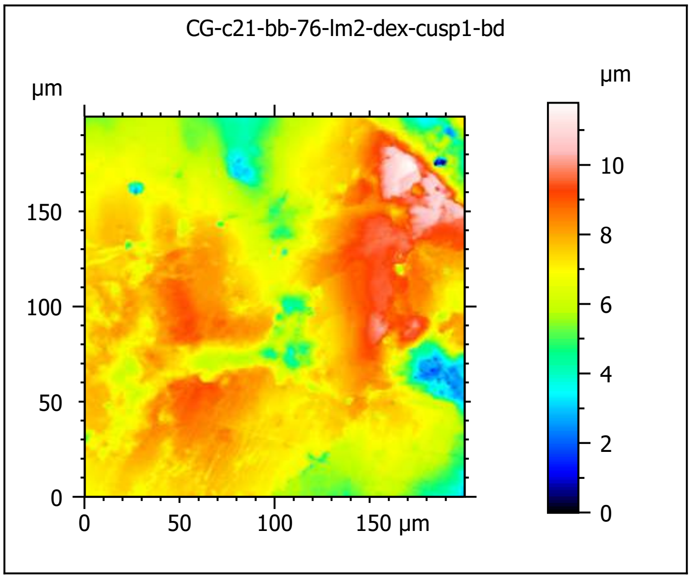

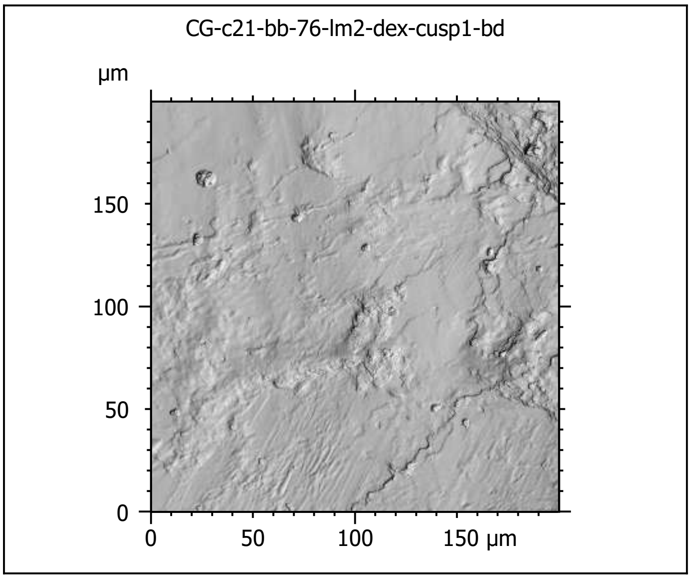

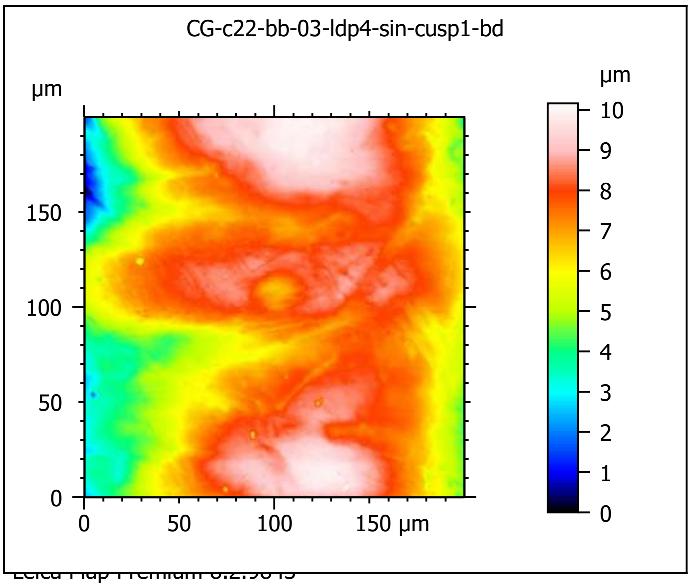

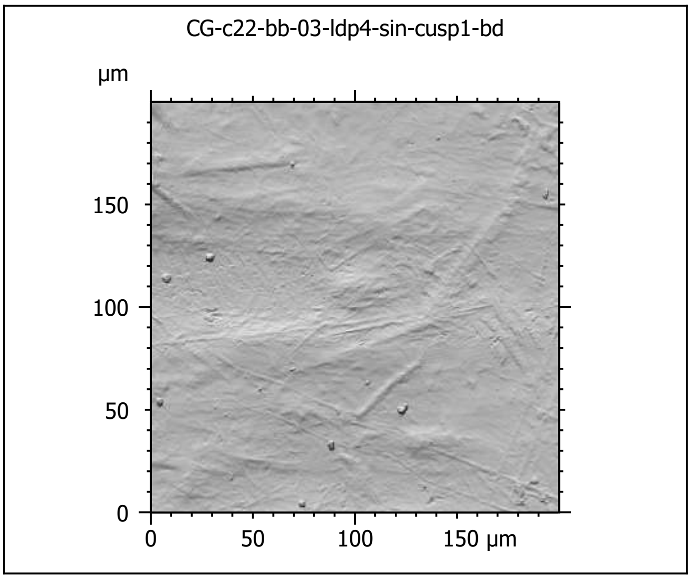

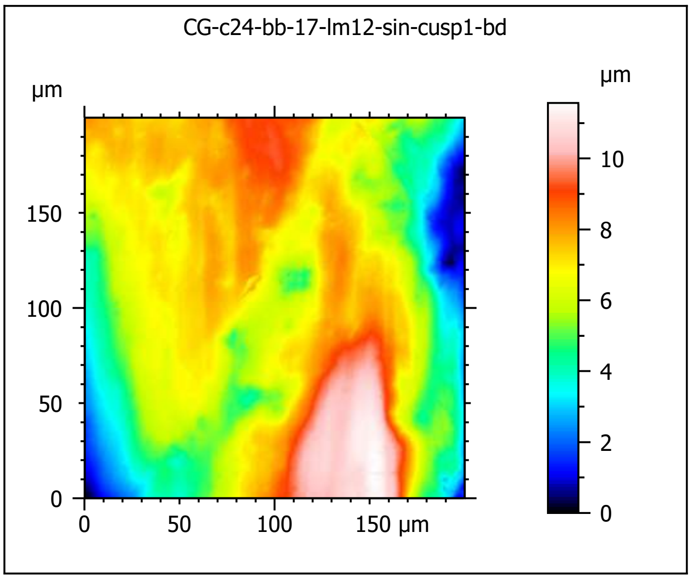
CG-c24-bb-17-lm12-sin-cusp1-bd
µm
150
100
50
0
0
50
100
150 µm
µm
10
8
6
4
2
0

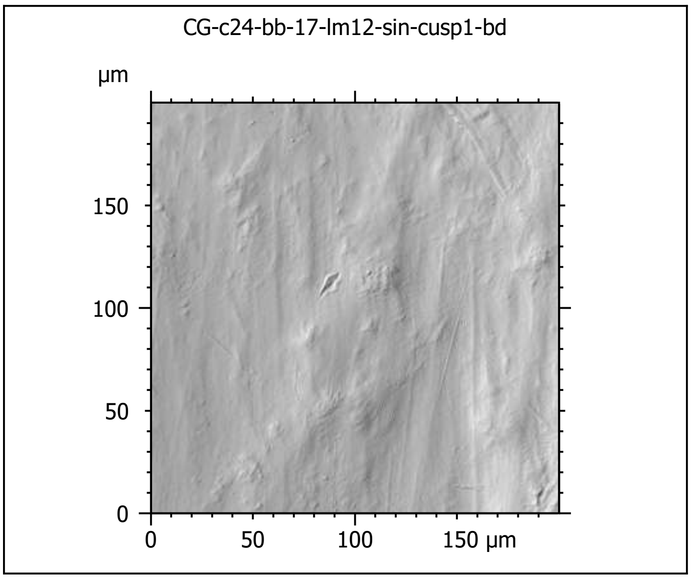
CG-c24-bb-17-lm12-sin-cusp1-bd
µm
150
100
50
0
0
50
100
150 µm

"A long-term perspective on Neandertal environment and subsistence: insights from the dental micro-texture analysis of hunted ungulates at Combe-Grenal (Dordogne, France)"

authors: Berlioz, E.; Capdepon, E.; Discamps, E.

Appendice 2:
surfaces scanned by E. Berlioz and E. Capdepon, pre-treatement by E. Berlioz and E. Capdepon, validation by E. Berlioz (2019)

Bos primigenius* / *Bison priscus **- Bock G**

CG-c29-bb-36-lm2-dex-cusp1-bd

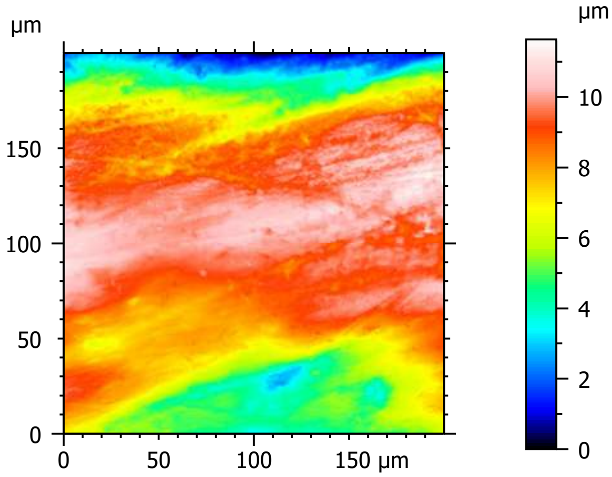

CG-c29-bb-36-lm2-dex-cusp1-bd

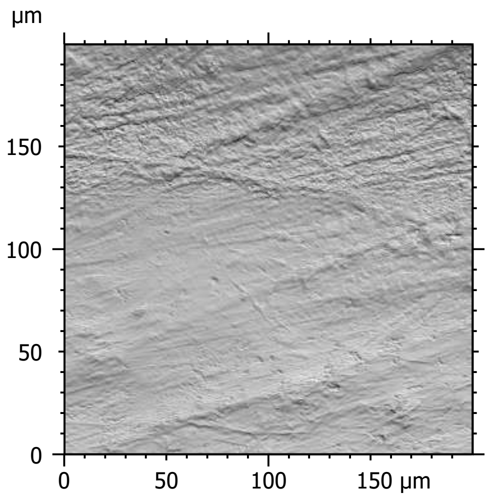

CG-c29-bb-94-lm3-sin-cusp1-bd

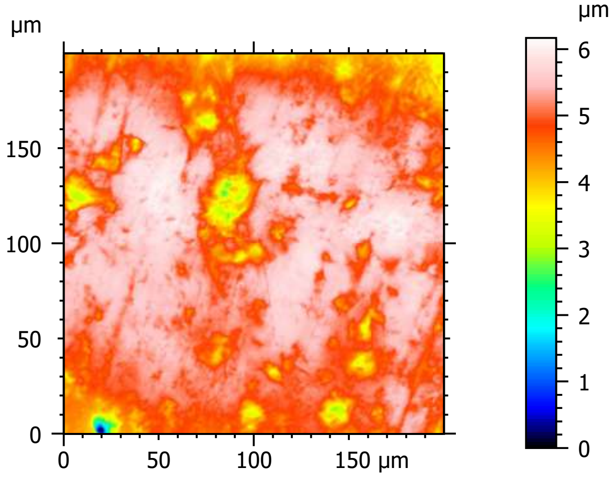

CG-c29-bb-94-lm3-sin-cusp1-bd

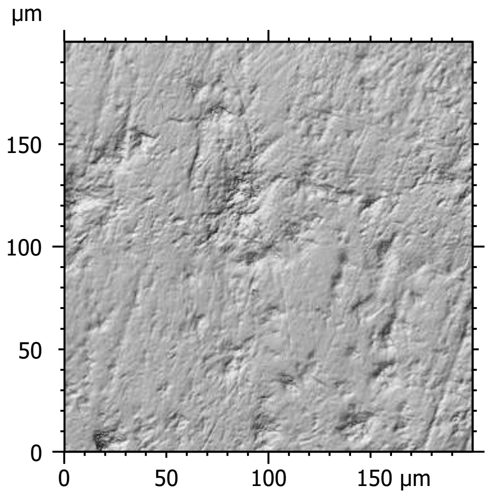

CG-c29-bb-96-lm12-sin-cusp1-bd

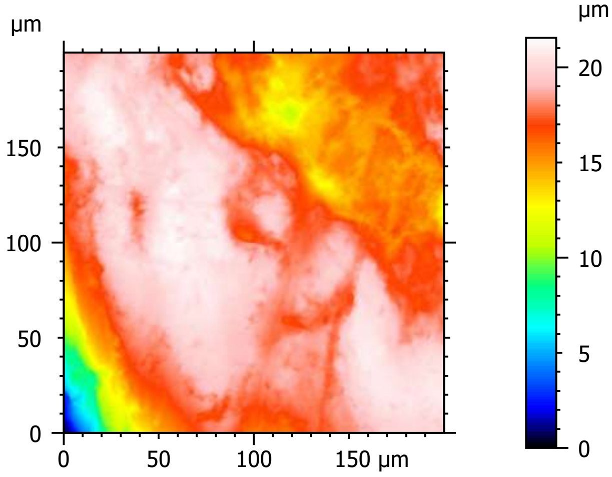

CG-c29-bb-96-lm12-sin-cusp1-bd

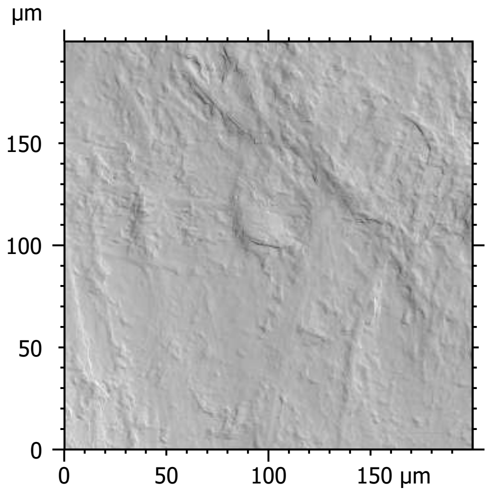

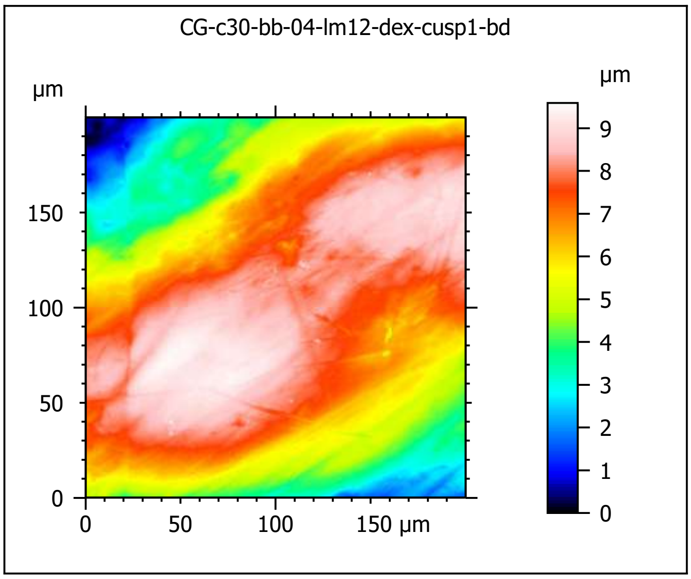

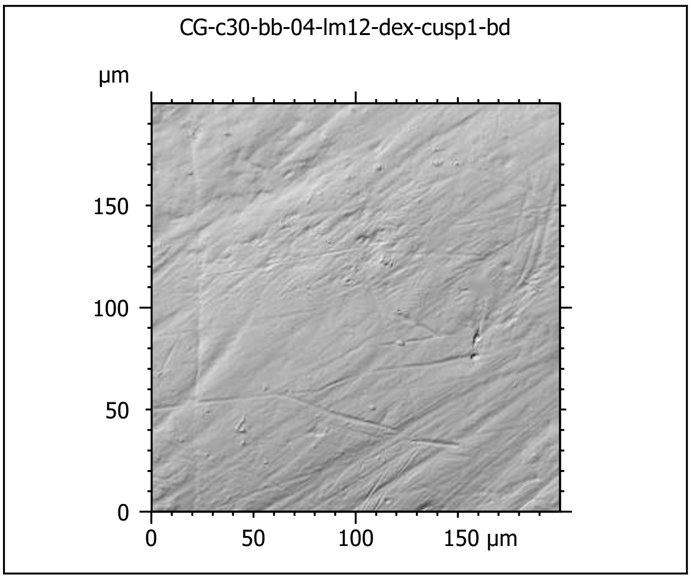

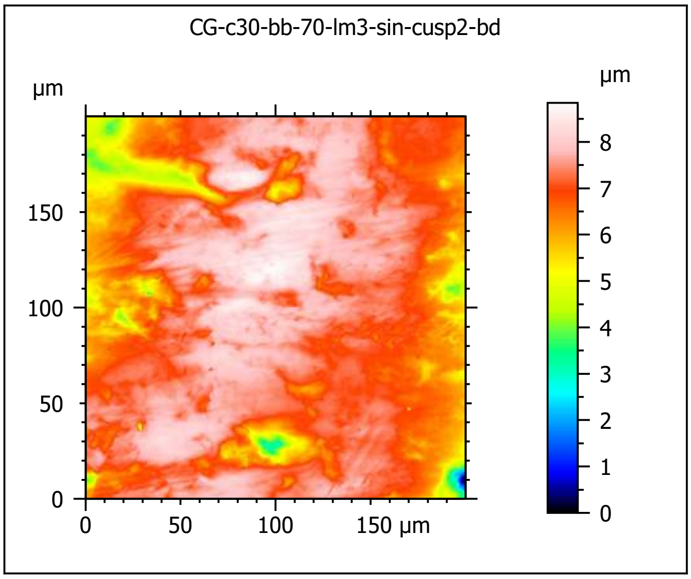

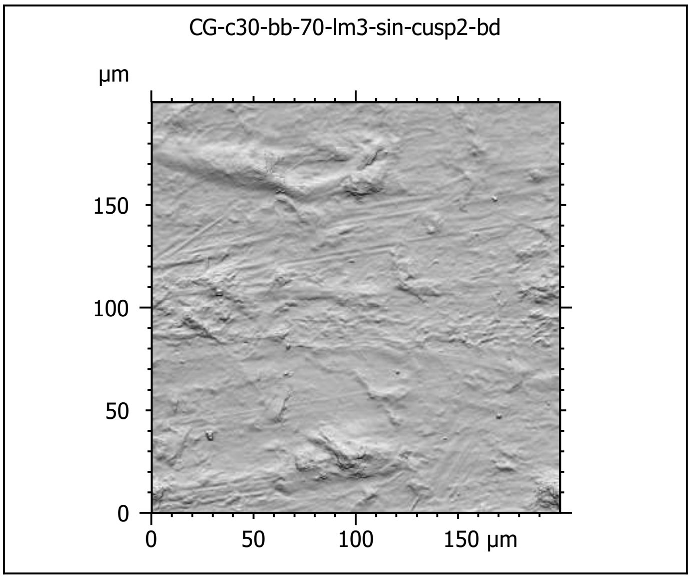

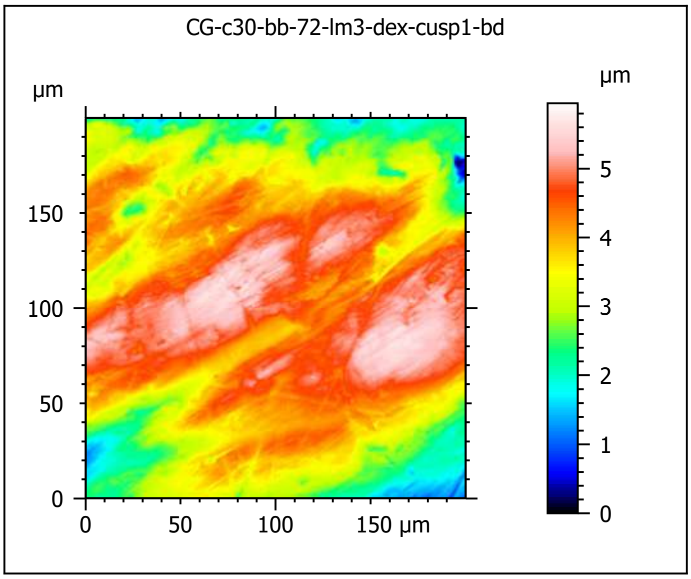

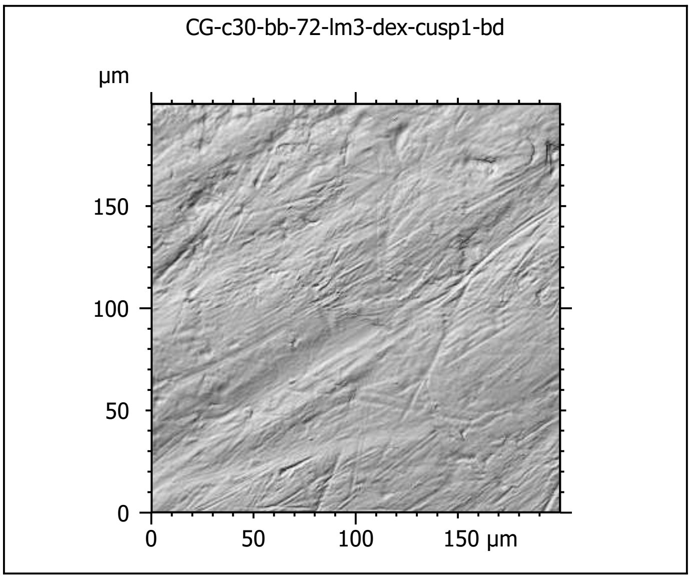

Leica Map Premium 8.2.9843

CG-c30-bb-73-lm12-dex-cusp1-bd

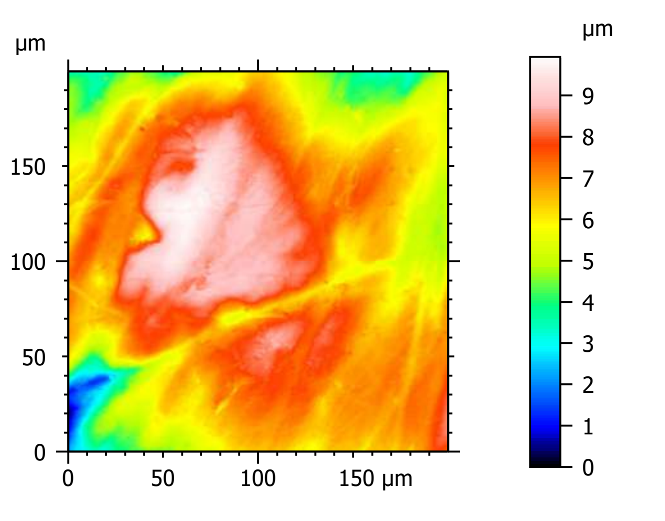

CG-c30-bb-73-lm12-dex-cusp1-bd

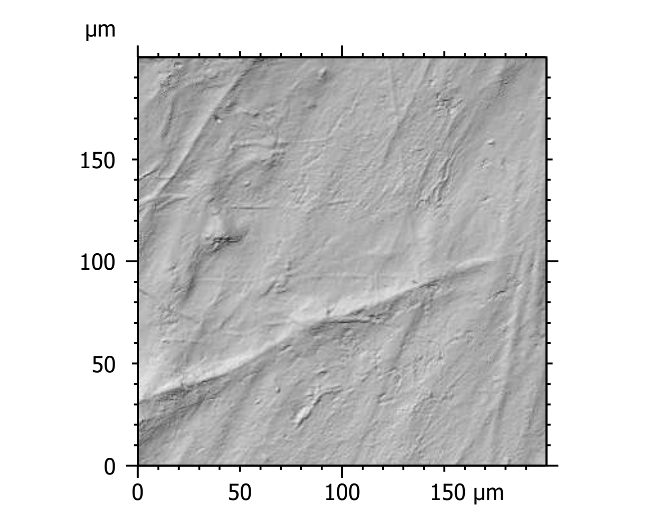

CG-c32-bb-13-lm1-dex-cusp1-bd

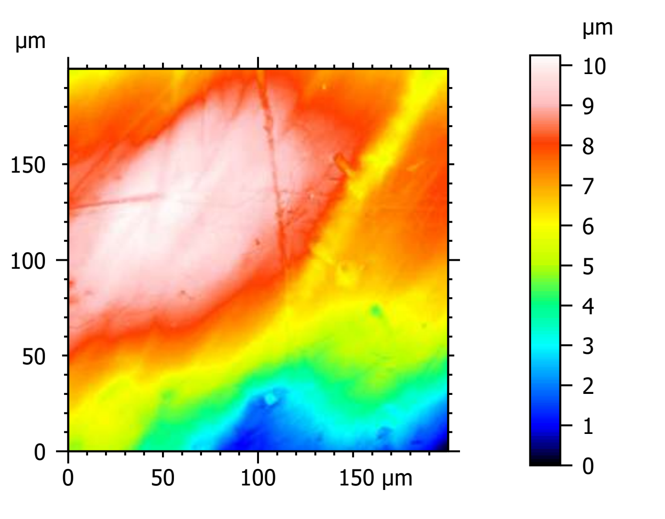

CG-c32-bb-13-lm1-dex-cusp1-bd

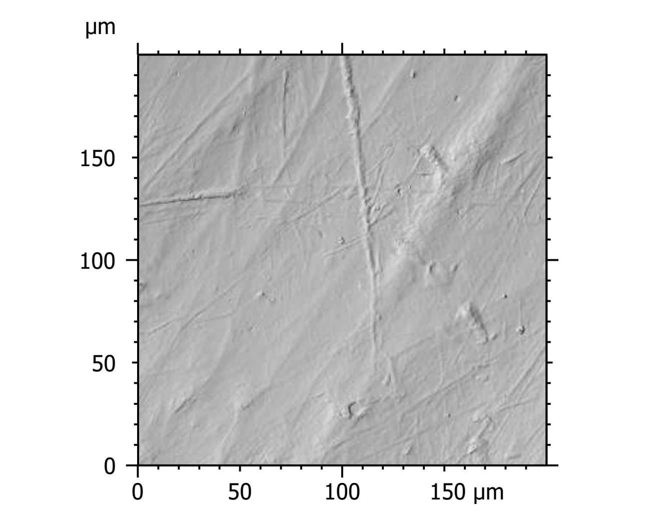

CG-c32-bb-34-lm3-dex-cusp1-bd

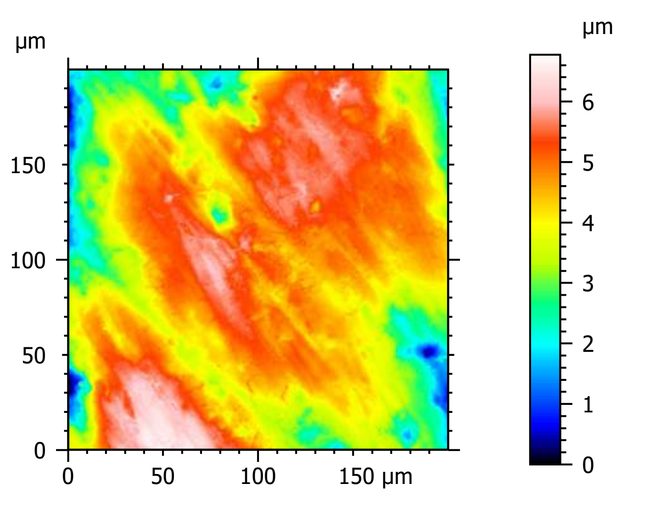

CG-c32-bb-34-lm3-dex-cusp1-bd

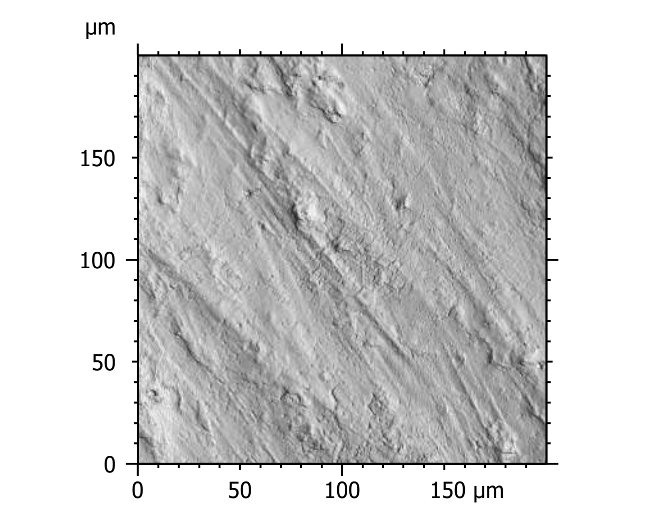

Leica Map Premium 8.2.9843

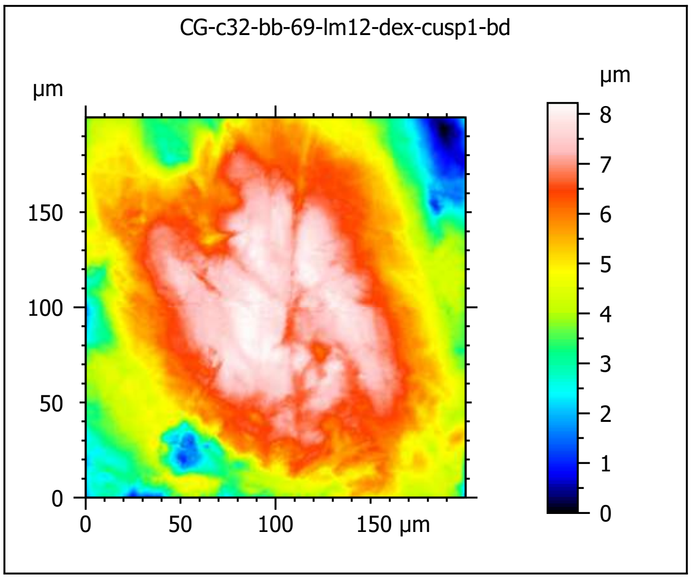
CG-c32-bb-69-lm12-dex-cusp1-bd
µm
150
100
50
0
0
50
100
150 µm
µm
8
7
6
5
4
3
2
1
0

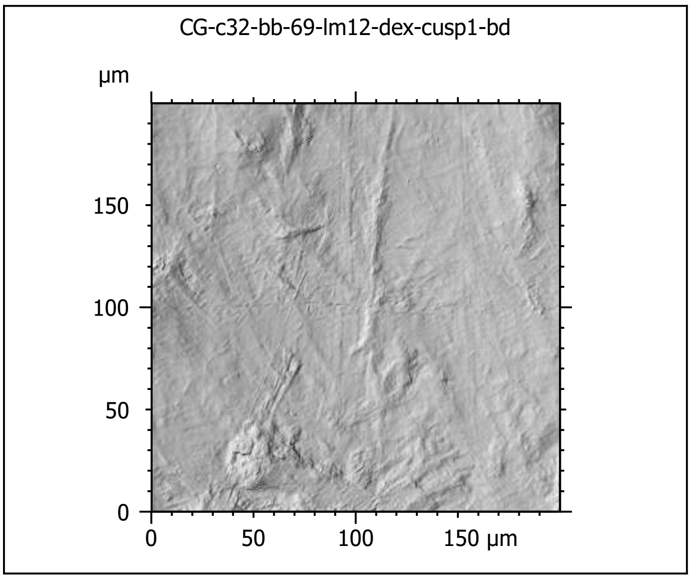
CG-c32-bb-69-lm12-dex-cusp1-bd
µm
150
100
50
0
0
50
100
150 µm

"A long-term perspective on Neandertal environment and subsistence: insights from the dental micro-texture analysis of hunted ungulates at Combe-Grenal (Dordogne, France)"

authors: Berlioz, E.; Capdepon, E.; Discamps, E.

Appendice 2:
surfaces scanned by E. Berlioz and E. Capdepon, pre-treatement by E. Berlioz and E. Capdepon, validation by E. Berlioz (2019)

***Bos primigenius* | *Bison priscus* - Bock I**

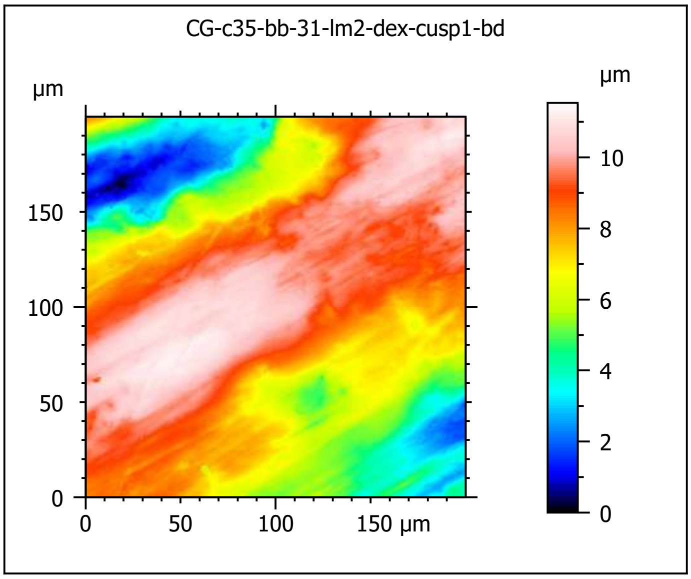

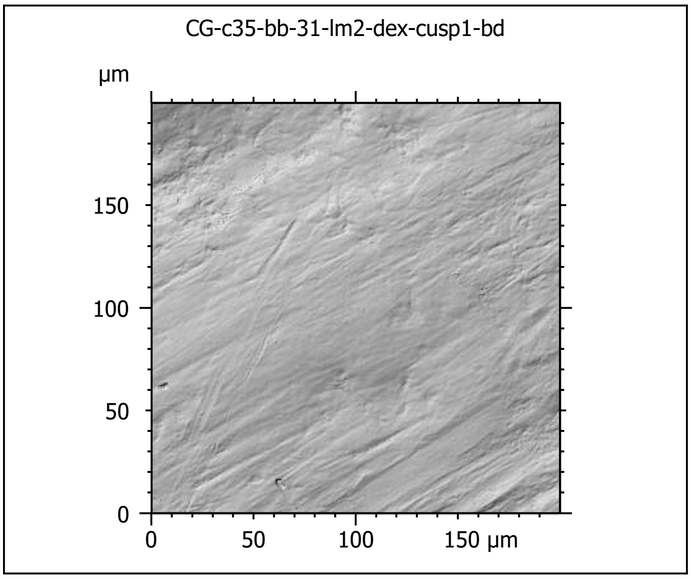

Leica Map Premium 8.2.9843
