# Supplementary material for: A long-term perspective on Neanderthal environment and subsistence: Insights from the dental microwear texture analysis of hunted ungulates at Combe-Grenal (Dordogne, France)
Source: PLoS One. 2023 Jan 18;18(1):e0278395. doi: 10.1371/journal.pone.0278395 (PMC9847971; doi:10.1371/journal.pone.0278395)
Supplement: S3 Appendix — (PDF) [file pone.0278395.s003.pdf]

A long-term perspective on Neanderthal environment and subsistence: insights from the dental micro-texture analysis of hunted ungulates at Combe-Grenal (Dordogne,France)

Appendix 3

Berlioz, Capdepon & Discamps, 2022

07/11/22

In this document are summarized the statistical analyses that have been made in order to analyse the data of the present paper. Statistical analyses were done following Smith & Warren (2019) and Zuur et al. (2010).

The questions that have been tested are the following:

```
1. Among red deer (*Cervus elaphus*), are they statistical differences in
   DMTA parameters through time ? (between blocks, between periods)

2. Among bovinae (*Bos primigenius* /*Bison priscus*), are they statistica
   l differences in DMTA parameters through time ? (between blocks, betwee
   n periods)

3. Among reindeer (*Rangifer tarandus*), are they statistical differences
   in DMTA parameters through time ? (between blocks, between periods)

4. Inside each of the blocks, are they significant inter-specific differen
   ces?

5. Inside each of the periods, are they significant inter-specific differe
   nces?
```

Load needed packages

```
require(agricolae)
require(AICcmodavg)
require(ape)
require(car)
require(corrplot)
require(emmeans)
require(FSA)
require(ggplot2)
require(Hmisc)
require(lattice)
require(lawstat)
require(MASS)
```

```
require(multcomp)
require(onewaytests)
require(outliers)
require(regclass)
require(RVAideMemoire)
require(stats)
require(vegan)

# Tools
require(dplyr)
require(Publish)
require(readr)
```

Import datasets & create subsets

```
getwd() # Working Directory = same as Rmd document's location

## [1] "C:/Users/eberl/Desktop/Ungulates Combe Grenal"

global_db_FOSSILES_withDisp <- read_delim("global db_FOSSILES_withDisp.csv",
  delim = ";", escape_double = FALSE, col_types = cols(
    `ref DMTA` = col_character(),
    Asfc = col_number(), epLsar = col_number(), Smc = col_number(), HAsfc9 =
col_number(),
    HAsfc81 = col_number(), HAsfc36 = col_number()
  ), trim_ws = TRUE
)

db_FOSSILS <- global_db_FOSSILES_withDisp[, 1:12]
summary(db_FOSSILS)

##     Group               specie              Period              Blocs
##  Length:368         Length:368         Length:368         Length:368
##  Class :character   Class :character   Class :character   Class :character
##  Mode  :character   Mode  :character   Mode  :character   Mode  :character
##
##
##
##     layer             ref DMTA              Asfc            epLsar
##  Length:368         Length:368         Min.   :0.155   Min.   : 0.427
##  Class :character   Class :character   1st Qu.:1.190   1st Qu.: 3.197
##  Mode  :character   Mode  :character   Median :1.810   Median : 4.804
##                                        Mean   :1.960   Mean   : 4.853
##                                        3rd Qu.:2.442   3rd Qu.: 6.446
##                                        Max.   :7.908   Max.   :10.607
##       Smc              HAsfc9           HAsfc81          HAsfc36
##  Min.   :  0.304   Min.   :0.0800   Min.   :0.3040   Min.   :0.2200
##  1st Qu.:  1.361   1st Qu.:0.2430   1st Qu.:0.5145   1st Qu.:0.4020
```

```
##  Median :  1.599   Median :0.3175   Median :0.6265   Median :0.4900
##  Mean   : 11.248   Mean   :0.3614   Mean   :0.6839   Mean   :0.5389
##  3rd Qu.:  3.346   3rd Qu.:0.4340   3rd Qu.:0.7605   3rd Qu.:0.5923
##  Max.   :222.612   Max.   :1.6790   Max.   :3.4940   Max.   :2.9750
```

```
db_FOSSILS$Group <- as.factor(db_FOSSILS$Group)
db_FOSSILS$specie <- as.factor(db_FOSSILS$specie)
db_FOSSILS$Period <- as.factor(db_FOSSILS$Period)
db_FOSSILS$Blocs <- as.factor(db_FOSSILS$Blocs)
db_FOSSILS$layer <- as.factor(db_FOSSILS$layer)
db_FOSSILS$Asfc <- as.numeric(db_FOSSILS$Asfc)
db_FOSSILS$epLsar <- as.numeric(db_FOSSILS$epLsar)
db_FOSSILS$Smc <- as.numeric(db_FOSSILS$Smc)
db_FOSSILS$HAsfc9 <- as.numeric(db_FOSSILS$HAsfc9)
db_FOSSILS$HAsfc36 <- as.numeric(db_FOSSILS$HAsfc36)
db_FOSSILS$HAsfc81 <- as.numeric(db_FOSSILS$HAsfc81)

db_FOSSILS <- as.data.frame(db_FOSSILS)

summary(db_FOSSILS)
```

```
##    Group     specie     Period        Blocs          layer
##  CG_BB: 50   BB: 50   AnteQ:110   F      :117   c22    : 34
##  CG_Ce:116   Ce:116   PostQ:112   B      : 73   c14    : 32
##  CG_Rt:202   Rt:202   Quina:146   G      : 72   c23    : 26
##                                   A      : 39   c21    : 25
##                                   I      : 31   c11    : 21
##                                   E      : 15   c27    : 20
##                                   (Other): 21   (Other):210
##    ref DMTA              Asfc           epLsar            Smc
##  Length:368         Min.   :0.155   Min.   : 0.427   Min.   :  0.304
##  Class :character   1st Qu.:1.190   1st Qu.: 3.197   1st Qu.:  1.361
##  Mode  :character   Median :1.810   Median : 4.804   Median :  1.599
##                     Mean   :1.960   Mean   : 4.853   Mean   : 11.248
##                     3rd Qu.:2.442   3rd Qu.: 6.446   3rd Qu.:  3.346
##                     Max.   :7.908   Max.   :10.607   Max.   :222.612
##
##      HAsfc9          HAsfc81          HAsfc36
##  Min.   :0.0800   Min.   :0.3040   Min.   :0.2200
##  1st Qu.:0.2430   1st Qu.:0.5145   1st Qu.:0.4020
##  Median :0.3175   Median :0.6265   Median :0.4900
##  Mean   :0.3614   Mean   :0.6839   Mean   :0.5389
##  3rd Qu.:0.4340   3rd Qu.:0.7605   3rd Qu.:0.5923
##  Max.   :1.6790   Max.   :3.4940   Max.   :2.9750
##
```

```
Period <- dplyr::select(db_FOSSILS, c(3))
Specie <- dplyr::select(db_FOSSILS, c(2))
Blocks <- dplyr::select(db_FOSSILS, c(4))
```

```
BB <- subset(db_FOSSILS, db_FOSSILS$specie == "BB")
Ce <- subset(db_FOSSILS, db_FOSSILS$specie == "Ce")
Rt <- subset(db_FOSSILS, db_FOSSILS$specie == "Rt")

A <- subset(db_FOSSILS, db_FOSSILS$Blocs == "A")
B <- subset(db_FOSSILS, db_FOSSILS$Blocs == "B")
C <- subset(db_FOSSILS, db_FOSSILS$Blocs == "C")
D <- subset(db_FOSSILS, db_FOSSILS$Blocs == "D")
E <- subset(db_FOSSILS, db_FOSSILS$Blocs == "E")
F <- subset(db_FOSSILS, db_FOSSILS$Blocs == "F")
G <- subset(db_FOSSILS, db_FOSSILS$Blocs == "G")
H <- subset(db_FOSSILS, db_FOSSILS$Blocs == "H")
I <- subset(db_FOSSILS, db_FOSSILS$Blocs == "I")

Asfc <- dplyr::select(db_FOSSILS, c(7))
epLsar <- dplyr::select(db_FOSSILS, c(8))
Smc <- dplyr::select(db_FOSSILS, c(9))
H9 <- dplyr::select(db_FOSSILS, c(10))
H36 <- dplyr::select(db_FOSSILS, c(12))
H81 <- dplyr::select(db_FOSSILS, c(11))
```

Multicolinearity between variables:

```
cor_db <- cor(Ce[, 7:12], use = "complete.obs")
cor_db

##                 Asfc      epLsar        Smc      HAsfc9     HAsfc81      HAs
fc36
## Asfc      1.00000000 -0.28179443 -0.2058078 -0.06590618 -0.16609330 -0.1471
4759
## epLsar   -0.28179443  1.00000000  0.3389295  0.04562464  0.08455993  0.0430
5339
## Smc      -0.20580779  0.33892947  1.0000000  0.16754076  0.31205329  0.2828
7499
## HAsfc9   -0.06590618  0.04562464  0.1675408  1.00000000  0.75718570  0.8010
8654
## HAsfc81 -0.16609330  0.08455993  0.3120533  0.75718570  1.00000000  0.9106
7037
## HAsfc36 -0.14714759  0.04305339  0.2828750  0.80108654  0.91067037  1.0000
0000

rcorr(as.matrix(Ce[, 7:12]))

##          Asfc epLsar   Smc HAsfc9 HAsfc81 HAsfc36
## Asfc     1.00  -0.28 -0.21  -0.07   -0.17   -0.15
## epLsar  -0.28   1.00  0.34   0.05    0.08    0.04
## Smc     -0.21   0.34  1.00   0.17    0.31    0.28
## HAsfc9  -0.07   0.05  0.17   1.00    0.76    0.80
## HAsfc81 -0.17   0.08  0.31   0.76    1.00    0.91
## HAsfc36 -0.15   0.04  0.28   0.80    0.91    1.00
##
## n= 116
```

```
##
##
## P
##         Asfc   epLsar Smc    HAsfc9 HAsfc81 HAsfc36
## Asfc           0.0022 0.0267 0.4821 0.0748  0.1150
## epLsar  0.0022        0.0002 0.6267 0.3668  0.6463
## Smc     0.0267 0.0002        0.0722 0.0006  0.0021
## HAsfc9  0.4821 0.6267 0.0722        0.0000  0.0000
## HAsfc81 0.0748 0.3668 0.0006 0.0000         0.0000
## HAsfc36 0.1150 0.6463 0.0021 0.0000 0.0000
```

```
symnum(cor_db, abbr.colnames = FALSE)
```

```
##         Asfc epLsar Smc HAsfc9 HAsfc81 HAsfc36
## Asfc    1
## epLsar       1
## Smc          .      1
## HAsfc9                  1
## HAsfc81             .   ,      1
## HAsfc36                 +      *       1
## attr(,"legend")
## [1] 0 ' ' 0.3 '.' 0.6 ',' 0.8 '+' 0.9 '*' 0.95 'B' 1
```

```
corrplot(cor_db, type = "upper", order = "hclust", tl.col = "black", tl.srt =
45)
```

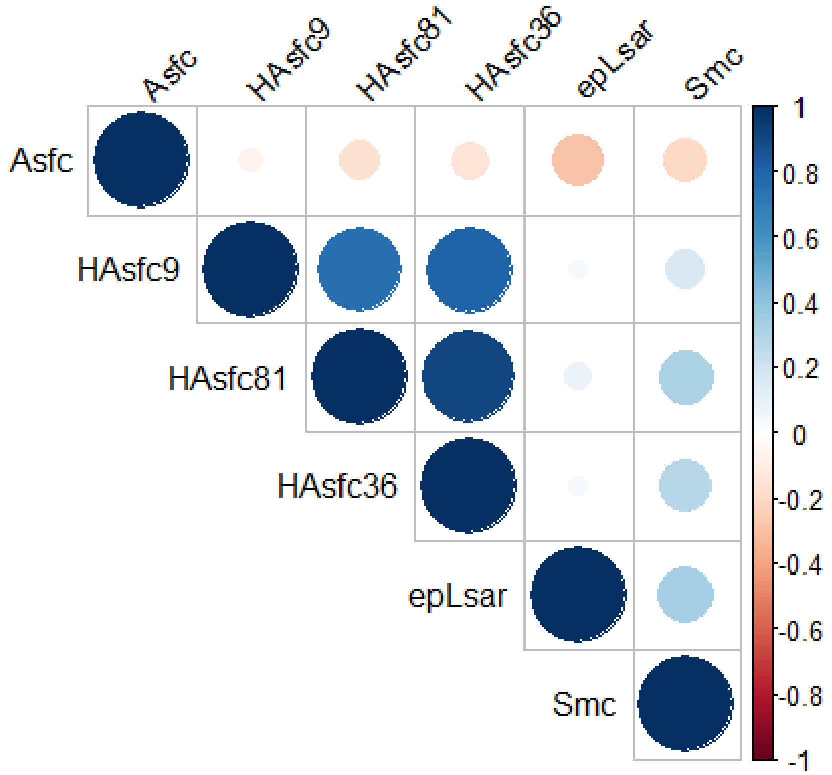


```
VIF(lm(unlist(Asfc) ~ unlist(Blocks) + unlist(Specie), data = db_FOSSILS))
```

```
##                    GVIF Df GVIF^(1/(2*Df))
## unlist(Blocks) 1.219814  7        1.014294
## unlist(Specie) 1.219814  2        1.050929
```

```
VIF(lm(unlist(epLsar) ~ unlist(Blocks) + unlist(Specie), data = db_FOSSILS))
```

```
##                    GVIF Df GVIF^(1/(2*Df))
## unlist(Blocks) 1.219814  7        1.014294
## unlist(Specie) 1.219814  2        1.050929
```

```
VIF(lm(unlist(Smc) ~ unlist(Blocks) + unlist(Specie), data = db_FOSSILS))
```

```
##                    GVIF Df GVIF^(1/(2*Df))
## unlist(Blocks) 1.219814  7        1.014294
## unlist(Specie) 1.219814  2        1.050929
```

```
VIF(lm(unlist(H9) ~ unlist(Blocks) + unlist(Specie), data = db_FOSSILS))
```

```
##                    GVIF Df GVIF^(1/(2*Df))
## unlist(Blocks) 1.219814  7        1.014294
## unlist(Specie) 1.219814  2        1.050929
```

```
VIF(lm(unlist(H36) ~ unlist(Blocks) + unlist(Specie), data = db_FOSSILS))
```

```
##                    GVIF Df GVIF^(1/(2*Df))
## unlist(Blocks) 1.219814  7        1.014294
## unlist(Specie) 1.219814  2        1.050929
```

```
VIF(lm(unlist(H81) ~ unlist(Blocks) + unlist(Specie), data = db_FOSSILS))
```

```
##                    GVIF Df GVIF^(1/(2*Df))
## unlist(Blocks) 1.219814  7        1.014294
## unlist(Specie) 1.219814  2        1.050929
```

Data exploration

```
summary(Ce)
```

```
##    Group      specie      Period        Blocs        layer        ref DMTA
##  CG_BB:  0   BB:  0   AnteQ:42   F      :37   c36    :13   Length:116
##  CG_Ce:116   Ce:116   PostQ:27   I      :21   c25    :11   Class :characte
r
##  CG_Rt:  0   Rt:  0   Quina:47   G      :19   c29    :11   Mode  :characte
r
##                                  A      :15   c21    : 8
##                                  B      :12   c23    : 8
##                                  D      : 5   c35    : 8
##                                  (Other): 7   (Other):57
##       Asfc           epLsar           Smc               HAsfc9
##  Min.   :0.272   Min.   : 0.624   Min.   :  0.304   Min.   :0.0810
```

```
##  1st Qu.:1.013   1st Qu.: 3.572   1st Qu.:  1.361   1st Qu.:0.2647
##  Median :1.389   Median : 5.527   Median :  1.837   Median :0.3310
##  Mean   :1.612   Mean   : 5.374   Mean   : 20.479   Mean   :0.3959
##  3rd Qu.:2.140   3rd Qu.: 7.301   3rd Qu.:  6.094   3rd Qu.:0.4510
##  Max.   :4.325   Max.   :10.607   Max.   :222.612   Max.   :1.6790
##
##     HAsfc81          HAsfc36
##  Min.   :0.3580   Min.   :0.2860
##  1st Qu.:0.5550   1st Qu.:0.4273
##  Median :0.6990   Median :0.5325
##  Mean   :0.7309   Mean   :0.5770
##  3rd Qu.:0.7940   3rd Qu.:0.6292
##  Max.   :3.4940   Max.   :2.9750
##
```

```
str(Ce)
```

```
## 'data.frame':    116 obs. of  12 variables:
##  $ Group   : Factor w/ 3 levels "CG_BB","CG_Ce",..: 2 2 2 2 2 2 2 2 2 2 ..
.
##  $ specie  : Factor w/ 3 levels "BB","Ce","Rt": 2 2 2 2 2 2 2 2 2 2 ...
##  $ Period  : Factor w/ 3 levels "AnteQ","PostQ",..: 2 2 2 2 2 2 2 2 2 2 ..
.
##  $ Blocs   : Factor w/ 8 levels "A","B","D","E",..: 1 1 1 1 1 1 1 1 1 1 ..
.
##  $ layer   : Factor w/ 31 levels "c10","c11","c12",..: 27 27 28 28 28 28 2
8 29 29 29 ...
##  $ ref DMTA: chr  "57" "58" "50" "51" ...
##  $ Asfc    : num  0.982 1.292 2.424 1.02 1.146 ...
##  $ epLsar  : num  7.81 8.1 5.77 7.43 5.75 ...
##  $ Smc     : num  6.09 1.84 49.71 1.36 1.36 ...
##  $ HAsfc9  : num  0.398 0.275 0.229 0.255 0.191 0.449 0.37 0.368 0.52 0.26
6 ...
##  $ HAsfc81 : num  0.556 0.835 0.731 0.413 0.358 0.753 0.811 0.746 0.696 0.
626 ...
##  $ HAsfc36 : num  0.433 0.546 0.373 0.323 0.286 0.555 0.62 0.545 0.537 0.4
7 ...
```

```
summary(Rt)
```

```
##    Group     specie     Period       Blocs        layer      ref DMTA
##  CG_BB:  0   BB:  0   AnteQ:57   F       :76   c22    :27   Length:202
##  CG_Ce:  0   Ce:  0   PostQ:51   G       :43   c14    :21   Class :characte
r
##  CG_Rt:202   Rt:202   Quina:94   B       :36   c23    :18   Mode  :characte
r
##                                  A       :15   c27    :16
##                                  D       : 9   c21    :15
##                                  E       : 9   c20    : 9
##                                  (Other):14   (Other):96
##       Asfc           epLsar            Smc              HAsfc9
```

```
## Min.   :0.155   Min.   : 0.443   Min.   :  0.410   Min.   :0.0800
## 1st Qu.:1.311   1st Qu.: 2.943   1st Qu.:  1.361   1st Qu.:0.2445
## Median :1.950   Median : 4.333   Median :  1.837   Median :0.3165
## Mean   :2.068   Mean   : 4.522   Mean   :  8.078   Mean   :0.3543
## 3rd Qu.:2.534   3rd Qu.: 5.897   3rd Qu.:  3.346   3rd Qu.:0.4148
## Max.   :7.115   Max.   :10.402   Max.   :222.612   Max.   :1.0550
##
##    HAsfc81          HAsfc36
## Min.   :0.3040   Min.   :0.2570
## 1st Qu.:0.5168   1st Qu.:0.4020
## Median :0.6055   Median :0.4850
## Mean   :0.6735   Mean   :0.5329
## 3rd Qu.:0.7535   3rd Qu.:0.5867
## Max.   :2.4350   Max.   :2.1040
##
```

```
str(Rt)
```

```
## 'data.frame':    202 obs. of  12 variables:
## $ Group   : Factor w/ 3 levels "CG_BB","CG_Ce",..: 3 3 3 3 3 3 3 3 3 3 ..
.
## $ specie  : Factor w/ 3 levels "BB","Ce","Rt": 3 3 3 3 3 3 3 3 3 3 ...
## $ Period  : Factor w/ 3 levels "AnteQ","PostQ",..: 2 2 2 2 2 2 2 2 2 2 ..
.
## $ Blocs   : Factor w/ 8 levels "A","B","D","E",..: 1 1 1 1 1 1 1 1 1 1 ..
.
## $ layer   : Factor w/ 31 levels "c10","c11","c12",..: 27 28 28 28 29 29 2
9 29 29 29 ...
## $ ref DMTA: chr  "56" "45" "46" "47" ...
## $ Asfc    : num  1.361 0.611 2.733 2.988 1.189 ...
## $ epLsar  : num  5.46 7.59 4.33 2.56 7.3 ...
## $ Smc     : num  0.747 67.093 67.093 4.516 0.41 ...
## $ HAsfc9  : num  0.363 0.343 0.435 0.366 0.106 0.282 0.247 0.222 0.267 0.
74 ...
## $ HAsfc81 : num  0.735 0.567 1.024 0.674 0.38 ...
## $ HAsfc36 : num  0.617 0.415 0.818 0.544 0.292 0.499 0.607 0.53 0.433 0.8
78 ...
```

```
summary(BB)
```

```
##   Group    specie    Period      Blocs       layer      ref DMTA
## CG_BB:50   BB:50   AnteQ:11   B      :25   c11    :16   Length:50
## CG_Ce: 0   Ce: 0   PostQ:34   G      :10   c10    : 6   Class :character
## CG_Rt: 0   Rt: 0   Quina: 5   A      : 9   c14    : 6   Mode  :character
##                               F      : 4   c30    : 4
##                               E      : 1   c29    : 3
##                               I      : 1   c32    : 3
##                               (Other): 0   (Other):12
##      Asfc          epLsar          Smc            HAsfc9
## Min.   :0.916   Min.   :0.427   Min.   : 0.304   Min.   :0.0860
## 1st Qu.:1.419   1st Qu.:3.319   1st Qu.: 1.096   1st Qu.:0.2032
```

```
##  Median :2.071   Median :5.087   Median : 1.361   Median :0.2590  
##  Mean   :2.335   Mean   :4.982   Mean   : 2.639   Mean   :0.3101  
##  3rd Qu.:2.674   3rd Qu.:7.203   3rd Qu.: 1.837   3rd Qu.:0.3740  
##  Max.   :7.908   Max.   :9.176   Max.   :49.712   Max.   :0.9210  
##                                                                   
##     HAsfc81          HAsfc36      
##  Min.   :0.3410   Min.   :0.2200  
##  1st Qu.:0.4710   1st Qu.:0.3785  
##  Median :0.5385   Median :0.4345  
##  Mean   :0.6172   Mean   :0.4748  
##  3rd Qu.:0.7298   3rd Qu.:0.5663  
##  Max.   :1.6210   Max.   :1.1460  
## 
```

```
BB$Blocs
```

```
##  [1] A A A A A A A A A B B B B B B B B B B B B B B B B B B B B B B B B B E
F F F
## [39] F G G G G G G G G G I
## Levels: A B D E F G H I
```

```
str(BB)
```

```
## 'data.frame':    50 obs. of  12 variables:
##  $ Group   : Factor w/ 3 levels "CG_BB","CG_Ce",..: 1 1 1 1 1 1 1 1 1 1 ..
.
##  $ specie  : Factor w/ 3 levels "BB","Ce","Rt": 1 1 1 1 1 1 1 1 1 1 ...
##  $ Period  : Factor w/ 3 levels "AnteQ","PostQ",..: 2 2 2 2 2 2 2 2 2 2 ..
.
##  $ Blocs   : Factor w/ 8 levels "A","B","D","E",..: 1 1 1 1 1 1 1 1 1 2 ..
.
##  $ layer   : Factor w/ 31 levels "c10","c11","c12",..: 27 28 31 1 1 1 1 1
1 2 ...
##  $ ref DMTA: chr  "21" "9" "7" "109" ...
##  $ Asfc    : num  3.99 1.51 1.27 1.43 1.9 ...
##  $ epLsar  : num  1.58 8.65 6.88 3.69 6.44 ...
##  $ Smc     : num  2.48 1.01 1.84 1.84 1.84 ...
##  $ HAsfc9  : num  0.634 0.287 0.17 0.29 0.714 0.15 0.258 0.476 0.44 0.263
...
##  $ HAsfc81 : num  0.959 0.446 0.341 0.751 0.953 0.452 0.484 0.759 0.757 0.
731 ...
##  $ HAsfc36 : num  0.729 0.336 0.22 0.585 0.763 0.451 0.375 0.558 0.624 0.5
69 ...
```

```
summary(A)
```

```
##    Group    specie    Period       Blocs        layer      ref DMTA        
##  CG_BB: 9   BB: 9   AnteQ: 0   A      :39   c7     :12   Length:39         
##  CG_Ce:15   Ce:15   PostQ:39   B      : 0   c6     : 9   Class :character  
##  CG_Rt:15   Rt:15   Quina: 0   D      : 0   c10    : 6   Mode  :character  
##                                E      : 0   c4     : 4                     
```

```
##                                F      : 0   c8     : 4
##                                G      : 0   c9     : 4
##                                (Other): 0   (Other): 0
##       Asfc           epLsar           Smc              HAsfc9
##  Min.   :0.459   Min.   :0.624   Min.   :  0.410   Min.   :0.1060
##  1st Qu.:1.275   1st Qu.:4.007   1st Qu.:  1.361   1st Qu.:0.2525
##  Median :1.719   Median :5.753   Median :  1.837   Median :0.2900
##  Mean   :2.107   Mean   :5.488   Mean   : 26.843   Mean   :0.3427
##  3rd Qu.:2.802   3rd Qu.:7.508   3rd Qu.: 16.692   3rd Qu.:0.4310
##  Max.   :5.938   Max.   :9.844   Max.   :222.612   Max.   :0.7400
##
##     HAsfc81          HAsfc36
##  Min.   :0.3410   Min.   :0.2200
##  1st Qu.:0.5395   1st Qu.:0.3950
##  Median :0.6740   Median :0.4990
##  Mean   :0.6738   Mean   :0.5068
##  3rd Qu.:0.7875   3rd Qu.:0.5960
##  Max.   :1.1690   Max.   :0.8780
##
```

```
str(A)
```

```
## 'data.frame':    39 obs. of  12 variables:
##  $ Group   : Factor w/ 3 levels "CG_BB","CG_Ce",..: 1 1 1 1 1 1 1 1 1 2 ..
.
##  $ specie  : Factor w/ 3 levels "BB","Ce","Rt": 1 1 1 1 1 1 1 1 1 2 ...
##  $ Period  : Factor w/ 3 levels "AnteQ","PostQ",..: 2 2 2 2 2 2 2 2 2 2 ..
.
##  $ Blocs   : Factor w/ 8 levels "A","B","D","E",..: 1 1 1 1 1 1 1 1 1 1 ..
.
##  $ layer   : Factor w/ 31 levels "c10","c11","c12",..: 27 28 31 1 1 1 1 1
1 27 ...
##  $ ref DMTA: chr  "21" "9" "7" "109" ...
##  $ Asfc    : num  3.99 1.51 1.27 1.43 1.9 ...
##  $ epLsar  : num  1.58 8.65 6.88 3.69 6.44 ...
##  $ Smc     : num  2.48 1.01 1.84 1.84 1.84 ...
##  $ HAsfc9  : num  0.634 0.287 0.17 0.29 0.714 0.15 0.258 0.476 0.44 0.398
...
##  $ HAsfc81 : num  0.959 0.446 0.341 0.751 0.953 0.452 0.484 0.759 0.757 0.
556 ...
##  $ HAsfc36 : num  0.729 0.336 0.22 0.585 0.763 0.451 0.375 0.558 0.624 0.4
33 ...
```

```
summary(B)
```

```
##    Group    specie    Period       Blocs        layer      ref DMTA
##  CG_BB:25   BB:25   AnteQ: 0   B      :73   c14    :32   Length:73
##  CG_Ce:12   Ce:12   PostQ:73   A      : 0   c11    :21   Class :character
##  CG_Rt:36   Rt:36   Quina: 0   D      : 0   c12    : 8   Mode  :character
##                                E      : 0   c13    : 7
##                                F      : 0   c15    : 5
```

```
##                                G      : 0   c10    : 0
##                                (Other): 0   (Other): 0
##       Asfc           epLsar           Smc              HAsfc9
##  Min.   :0.814   Min.   : 0.874   Min.   :  0.410   Min.   :0.0860
##  1st Qu.:1.541   1st Qu.: 3.092   1st Qu.:  1.008   1st Qu.:0.2270
##  Median :2.111   Median : 4.309   Median :  1.361   Median :0.2820
##  Mean   :2.156   Mean   : 4.779   Mean   :  4.800   Mean   :0.3072
##  3rd Qu.:2.515   3rd Qu.: 6.276   3rd Qu.:  1.837   3rd Qu.:0.3750
##  Max.   :4.537   Max.   :10.402   Max.   :164.942   Max.   :0.7550
##
##     HAsfc81          HAsfc36
##  Min.   :0.3470   Min.   :0.2650
##  1st Qu.:0.5060   1st Qu.:0.3890
##  Median :0.5680   Median :0.4410
##  Mean   :0.6154   Mean   :0.4818
##  3rd Qu.:0.7170   3rd Qu.:0.5570
##  Max.   :1.3360   Max.   :1.0050
##
```

```
str(B)
```

```
## 'data.frame':    73 obs. of  12 variables:
##  $ Group   : Factor w/ 3 levels "CG_BB","CG_Ce",..: 1 1 1 1 1 1 1 1 1 1 ..
.
##  $ specie  : Factor w/ 3 levels "BB","Ce","Rt": 1 1 1 1 1 1 1 1 1 1 ...
##  $ Period  : Factor w/ 3 levels "AnteQ","PostQ",..: 2 2 2 2 2 2 2 2 2 2 ..
.
##  $ Blocs   : Factor w/ 8 levels "A","B","D","E",..: 2 2 2 2 2 2 2 2 2 2 ..
.
##  $ layer   : Factor w/ 31 levels "c10","c11","c12",..: 2 2 2 2 2 2 2 2 2 2
...
##  $ ref DMTA: chr  "43" "45" "46" "47" ...
##  $ Asfc    : num  1.16 1.66 2.22 2.91 1.17 ...
##  $ epLsar  : num  4 2.32 3.38 5.66 7.92 ...
##  $ Smc     : num  1.01 1.36 1.01 1.84 1.36 ...
##  $ HAsfc9  : num  0.263 0.247 0.086 0.234 0.243 0.207 0.377 0.236 0.324 0.
219 ...
##  $ HAsfc81 : num  0.731 0.459 0.391 0.583 0.496 0.702 0.726 0.463 0.633 0.
529 ...
##  $ HAsfc36 : num  0.569 0.354 0.294 0.384 0.474 0.451 0.606 0.38 0.499 0.3
81 ...
```

```
summary(D)
```

```
##    Group   specie   Period       Blocs        layer     ref DMTA
##  CG_BB:0   BB:0   AnteQ: 0   D      :14   c17    :7   Length:14
##  CG_Ce:5   Ce:5   PostQ: 0   A      : 0   c19    :5   Class :character
##  CG_Rt:9   Rt:9   Quina:14   B      : 0   c18    :2   Mode  :character
##                              E      : 0   c10    :0
##                              F      : 0   c11    :0
##                              G      : 0   c12    :0
```

```
##                               (Other): 0   (Other):0
##       Asfc           epLsar           Smc            HAsfc9
##  Min.   :1.300   Min.   :0.443   Min.   :0.304   Min.   :0.1170
##  1st Qu.:1.565   1st Qu.:2.379   1st Qu.:1.096   1st Qu.:0.2095
##  Median :2.083   Median :2.759   Median :1.361   Median :0.3360
##  Mean   :2.574   Mean   :3.252   Mean   :1.631   Mean   :0.3304
##  3rd Qu.:2.592   3rd Qu.:4.096   3rd Qu.:1.718   3rd Qu.:0.4258
##  Max.   :7.115   Max.   :6.117   Max.   :6.094   Max.   :0.6900
##
##     HAsfc81          HAsfc36
##  Min.   :0.4150   Min.   :0.3140
##  1st Qu.:0.4803   1st Qu.:0.3420
##  Median :0.6365   Median :0.5330
##  Mean   :0.6101   Mean   :0.4839
##  3rd Qu.:0.7057   3rd Qu.:0.5850
##  Max.   :0.8120   Max.   :0.6460
##
```

```
str(D)
```

```
## 'data.frame':    14 obs. of  12 variables:
##  $ Group   : Factor w/ 3 levels "CG_BB","CG_Ce",..: 2 2 2 2 2 3 3 3 3 3 ..
.
##  $ specie  : Factor w/ 3 levels "BB","Ce","Rt": 2 2 2 2 2 3 3 3 3 3 ...
##  $ Period  : Factor w/ 3 levels "AnteQ","PostQ",..: 3 3 3 3 3 3 3 3 3 3 ..
.
##  $ Blocs   : Factor w/ 8 levels "A","B","D","E",..: 3 3 3 3 3 3 3 3 3 3 ..
.
##  $ layer   : Factor w/ 31 levels "c10","c11","c12",..: 7 7 7 7 9 7 7 7 8 8
...
##  $ ref DMTA: chr  "118" "119" "120" "121" ...
##  $ Asfc    : num  2.17 1.99 1.83 4.14 1.3 ...
##  $ epLsar  : num  2.22 2.47 3.93 5.88 6.12 ...
##  $ Smc     : num  1.008 1.837 1.008 0.304 1.361 ...
##  $ HAsfc9  : num  0.331 0.256 0.455 0.69 0.456 0.398 0.195 0.171 0.356 0.3
41 ...
##  $ HAsfc81 : num  0.693 0.419 0.674 0.764 0.756 0.648 0.453 0.568 0.562 0.
625 ...
##  $ HAsfc36 : num  0.564 0.326 0.563 0.646 0.639 0.592 0.314 0.426 0.537 0.
529 ...
```

```
summary(E)
```

```
##    Group    specie   Period       Blocs        layer      ref DMTA
##  CG_BB:1   BB:1   AnteQ: 0   E      :15   c20    :15   Length:15
##  CG_Ce:5   Ce:5   PostQ: 0   A      : 0   c10    : 0   Class :character
##  CG_Rt:9   Rt:9   Quina:15   B      : 0   c11    : 0   Mode  :character
##                              D      : 0   c12    : 0
##                              F      : 0   c13    : 0
##                              G      : 0   c14    : 0
##                              (Other): 0   (Other): 0
```

```
##       Asfc           epLsar          Smc             HAsfc9
##  Min.   :0.871   Min.   :2.067   Min.   : 0.554   Min.   :0.0810
##  1st Qu.:1.331   1st Qu.:2.828   1st Qu.: 1.361   1st Qu.:0.2285
##  Median :2.113   Median :3.205   Median : 1.361   Median :0.3500
##  Mean   :2.132   Mean   :4.402   Mean   : 5.702   Mean   :0.3699
##  3rd Qu.:2.905   3rd Qu.:6.122   3rd Qu.: 2.591   3rd Qu.:0.4860
##  Max.   :3.487   Max.   :8.430   Max.   :49.712   Max.   :0.7670
##
##     HAsfc81          HAsfc36
##  Min.   :0.4150   Min.   :0.3090
##  1st Qu.:0.5410   1st Qu.:0.3950
##  Median :0.6310   Median :0.5170
##  Mean   :0.6617   Mean   :0.5265
##  3rd Qu.:0.7795   3rd Qu.:0.6320
##  Max.   :0.9380   Max.   :0.7960
##
```

```
str(E)
```

```
## 'data.frame':    15 obs. of  12 variables:
##  $ Group   : Factor w/ 3 levels "CG_BB","CG_Ce",..: 1 2 2 2 2 2 3 3 3 3 ..
.
##  $ specie  : Factor w/ 3 levels "BB","Ce","Rt": 1 2 2 2 2 2 3 3 3 3 ...
##  $ Period  : Factor w/ 3 levels "AnteQ","PostQ",..: 3 3 3 3 3 3 3 3 3 3 ..
.
##  $ Blocs   : Factor w/ 8 levels "A","B","D","E",..: 4 4 4 4 4 4 4 4 4 4 ..
.
##  $ layer   : Factor w/ 31 levels "c10","c11","c12",..: 10 10 10 10 10 10 1
0 10 10 10 ...
##  $ ref DMTA: chr  "78" "102" "103" "104" ...
##  $ Asfc    : num  0.916 1.376 0.871 1.144 2.689 ...
##  $ epLsar  : num  8.43 7.68 7.7 2.47 2.07 ...
##  $ Smc     : num  3.346 1.837 1.361 49.712 0.747 ...
##  $ HAsfc9  : num  0.162 0.141 0.555 0.221 0.471 0.081 0.35 0.307 0.545 0.3
31 ...
##  $ HAsfc81 : num  0.508 0.443 0.838 0.477 0.623 0.415 0.574 0.721 0.629 0.
631 ...
##  $ HAsfc36 : num  0.384 0.329 0.646 0.363 0.597 0.309 0.476 0.583 0.661 0.
406 ...
```

```
summary(F)
```

```
##    Group    specie    Period       Blocs         layer      ref DMTA
##  CG_BB: 4   BB: 4   AnteQ:  0   F      :117   c22    :34   Length:117
##  CG_Ce:37   Ce:37   PostQ:  0   A      :  0   c23    :26   Class :characte
r
##  CG_Rt:76   Rt:76   Quina:117   B      :  0   c21    :25   Mode  :characte
r
##                                 D      :  0   c25    :17
##                                 E      :  0   c24    : 9
##                                 G      :  0   c26    : 6
```

```
##                                 (Other):  0   (Other): 0
##       Asfc           epLsar           Smc              HAsfc9
##  Min.   :0.155   Min.   :0.666   Min.   :  0.410   Min.   :0.0800
##  1st Qu.:1.036   1st Qu.:3.203   1st Qu.:  1.361   1st Qu.:0.2600
##  Median :1.448   Median :4.751   Median :  1.837   Median :0.3390
##  Mean   :1.768   Mean   :4.740   Mean   : 15.777   Mean   :0.4101
##  3rd Qu.:2.279   3rd Qu.:6.315   3rd Qu.:  3.346   3rd Qu.:0.4720
##  Max.   :6.715   Max.   :9.085   Max.   :222.612   Max.   :1.6790
##
##     HAsfc81          HAsfc36
##  Min.   :0.3040   Min.   :0.2570
##  1st Qu.:0.5370   1st Qu.:0.4170
##  Median :0.6390   Median :0.5020
##  Mean   :0.7532   Mean   :0.5863
##  3rd Qu.:0.8390   3rd Qu.:0.6410
##  Max.   :3.4940   Max.   :2.9750
##
```

```
str(F)
```

```
## 'data.frame':    117 obs. of  12 variables:
##  $ Group   : Factor w/ 3 levels "CG_BB","CG_Ce",..: 1 1 1 1 2 2 2 2 2 2 ..
.
##  $ specie  : Factor w/ 3 levels "BB","Ce","Rt": 1 1 1 1 2 2 2 2 2 2 ...
##  $ Period  : Factor w/ 3 levels "AnteQ","PostQ",..: 3 3 3 3 3 3 3 3 3 3 ..
.
##  $ Blocs   : Factor w/ 8 levels "A","B","D","E",..: 5 5 5 5 5 5 5 5 5 5 ..
.
##  $ layer   : Factor w/ 31 levels "c10","c11","c12",..: 11 11 12 14 11 11 1
1 11 11 11 ...
##  $ ref DMTA: chr  "75" "76" "3" "17" ...
##  $ Asfc    : num  1.416 6.715 1.799 1.268 0.825 ...
##  $ epLsar  : num  3.203 4.895 0.786 7.469 4.615 ...
##  $ Smc     : num  1.36 1.84 2.48 49.71 67.09 ...
##  $ HAsfc9  : num  0.222 0.446 0.339 0.921 0.454 1.19 0.367 0.237 0.892 0.2
32 ...
##  $ HAsfc81 : num  0.62 0.873 0.632 1.621 0.674 ...
##  $ HAsfc36 : num  0.454 0.533 0.502 1.146 0.486 ...
```

```
summary(G)
```

```
##    Group    specie    Period       Blocs        layer      ref DMTA
##  CG_BB:10   BB:10   AnteQ:72   G      :72   c27    :20   Length:72
##  CG_Ce:19   Ce:19   PostQ: 0   A      : 0   c29    :20   Class :character
##  CG_Rt:43   Rt:43   Quina: 0   B      : 0   c28    :10   Mode  :character
##                                D      : 0   c30    :10
##                                E      : 0   c32    : 7
##                                F      : 0   c31    : 5
##                                (Other): 0   (Other): 0
##       Asfc           epLsar           Smc              HAsfc9
##  Min.   :0.579   Min.   :0.427   Min.   :  0.304   Min.   :0.1120
```

```
##  1st Qu.:1.137   1st Qu.:3.250   1st Qu.:  1.008   1st Qu.:0.2425
##  Median :1.876   Median :5.093   Median :  1.599   Median :0.3145
##  Mean   :1.975   Mean   :4.714   Mean   :  7.850   Mean   :0.3659
##  3rd Qu.:2.364   3rd Qu.:6.319   3rd Qu.:  3.346   3rd Qu.:0.4253
##  Max.   :7.908   Max.   :8.400   Max.   :122.212   Max.   :1.0550
##
##     HAsfc81          HAsfc36
##  Min.   :0.3690   Min.   :0.2920
##  1st Qu.:0.4938   1st Qu.:0.3895
##  Median :0.6125   Median :0.4870
##  Mean   :0.6784   Mean   :0.5609
##  3rd Qu.:0.7445   3rd Qu.:0.6150
##  Max.   :2.4350   Max.   :2.1040
##
```

```
str(G)
```

```
## 'data.frame':    72 obs. of  12 variables:
##  $ Group   : Factor w/ 3 levels "CG_BB","CG_Ce",..: 1 1 1 1 1 1 1 1 1 1 ..
.
##  $ specie  : Factor w/ 3 levels "BB","Ce","Rt": 1 1 1 1 1 1 1 1 1 1 ...
##  $ Period  : Factor w/ 3 levels "AnteQ","PostQ",..: 1 1 1 1 1 1 1 1 1 1 ..
.
##  $ Blocs   : Factor w/ 8 levels "A","B","D","E",..: 6 6 6 6 6 6 6 6 6 6 ..
.
##  $ layer   : Factor w/ 31 levels "c10","c11","c12",..: 19 19 19 20 20 20 2
0 22 22 22 ...
##  $ ref DMTA: chr  "36" "94" "96" "4" ...
##  $ Asfc    : num  7.91 2.77 5.54 1.23 3.02 ...
##  $ epLsar  : num  6.8 0.427 3.45 7.312 3.299 ...
##  $ Smc     : num  0.554 1.361 2.479 1.361 1.361 ...
##  $ HAsfc9  : num  0.56 0.148 0.212 0.261 0.26 0.172 0.153 0.255 0.202 0.45
9 ...
##  $ HAsfc81 : num  0.64 0.439 0.474 0.485 0.657 0.436 0.416 0.853 0.369 0.8
21 ...
##  $ HAsfc36 : num  0.613 0.39 0.386 0.353 0.503 0.386 0.297 0.649 0.319 0.5
9 ...
```

```
summary(H)
```

```
##    Group    specie   Period      Blocs       layer     ref DMTA
##  CG_BB:0   BB:0   AnteQ:7   H      :7   c33    :7   Length:7
##  CG_Ce:2   Ce:2   PostQ:0   A      :0   c10    :0   Class :character
##  CG_Rt:5   Rt:5   Quina:0   B      :0   c11    :0   Mode  :character
##                             D      :0   c12    :0
##                             E      :0   c13    :0
##                             F      :0   c14    :0
##                             (Other):0   (Other):0
##       Asfc           epLsar           Smc              HAsfc9
##  Min.   :1.173   Min.   :4.132   Min.   :0.7470   Min.   :0.1370
##  1st Qu.:1.210   1st Qu.:5.263   1st Qu.:0.8775   1st Qu.:0.3180
```

```
##  Median :1.311   Median :7.331   Median :1.3610   Median :0.4290
##  Mean   :1.680   Mean   :6.611   Mean   :1.3629   Mean   :0.3693
##  3rd Qu.:2.064   3rd Qu.:7.688   3rd Qu.:1.5990   3rd Qu.:0.4475
##  Max.   :2.727   Max.   :8.914   Max.   :2.4790   Max.   :0.4880
##
##     HAsfc81          HAsfc36
##  Min.   :0.4970   Min.   :0.3290
##  1st Qu.:0.5905   1st Qu.:0.4825
##  Median :0.7050   Median :0.6290
##  Mean   :0.7453   Mean   :0.6117
##  3rd Qu.:0.7995   3rd Qu.:0.7160
##  Max.   :1.2350   Max.   :0.9270
##
```

```
str(H)
```

```
## 'data.frame':    7 obs. of  12 variables:
##  $ Group   : Factor w/ 3 levels "CG_BB","CG_Ce",..: 2 2 3 3 3 3 3
##  $ specie  : Factor w/ 3 levels "BB","Ce","Rt": 2 2 3 3 3 3 3
##  $ Period  : Factor w/ 3 levels "AnteQ","PostQ",..: 1 1 1 1 1 1 1
##  $ Blocs   : Factor w/ 8 levels "A","B","D","E",..: 7 7 7 7 7 7 7
##  $ layer   : Factor w/ 31 levels "c10","c11","c12",..: 23 23 23 23 23 23 2
3
##  $ ref DMTA: chr  "540" "544" "539" "541" ...
##  $ Asfc    : num  2.73 1.24 1.31 1.18 1.17 ...
##  $ epLsar  : num  8.91 7.99 7.33 5.6 4.92 ...
##  $ Smc     : num  1.361 1.008 1.361 0.747 1.837 ...
##  $ HAsfc9  : num  0.461 0.418 0.218 0.137 0.488 0.429 0.434
##  $ HAsfc81 : num  0.705 0.709 0.525 0.497 0.89 ...
##  $ HAsfc36 : num  0.629 0.682 0.415 0.329 0.75 0.927 0.55
```

```
summary(I)
```

```
##    Group    specie    Period       Blocs        layer      ref DMTA
##  CG_BB: 1   BB: 1   AnteQ:31   I      :31   c36    :16   Length:31
##  CG_Ce:21   Ce:21   PostQ: 0   A      : 0   c35    :14   Class :character
##  CG_Rt: 9   Rt: 9   Quina: 0   B      : 0   c34    : 1   Mode  :character
##                                D      : 0   c10    : 0
##                                E      : 0   c11    : 0
##                                F      : 0   c12    : 0
##                                (Other): 0   (Other): 0
##       Asfc           epLsar            Smc             HAsfc9
##  Min.   :0.516   Min.   : 1.658   Min.   : 0.410   Min.   :0.1220
##  1st Qu.:1.139   1st Qu.: 4.471   1st Qu.: 1.185   1st Qu.:0.2680
##  Median :1.638   Median : 5.491   Median : 1.837   Median :0.3410
##  Mean   :1.709   Mean   : 5.525   Mean   : 6.872   Mean   :0.3265
##  3rd Qu.:2.127   3rd Qu.: 6.274   3rd Qu.: 2.913   3rd Qu.:0.3920
##  Max.   :3.776   Max.   :10.607   Max.   :67.093   Max.   :0.5090
##
##     HAsfc81          HAsfc36
##  Min.   :0.3600   Min.   :0.2940
```

```
##   1st Qu.:0.5365   1st Qu.:0.4225  
##   Median :0.6270   Median :0.4990  
##   Mean   :0.6393   Mean   :0.4985  
##   3rd Qu.:0.7355   3rd Qu.:0.5655  
##   Max.   :1.2000   Max.   :0.9100  
##
```

```
str(I)
```

```
## 'data.frame':    31 obs. of  12 variables:
##  $ Group   : Factor w/ 3 levels "CG_BB","CG_Ce",..: 1 2 2 2 2 2 2 2 2 2 ..
.
##  $ specie  : Factor w/ 3 levels "BB","Ce","Rt": 1 2 2 2 2 2 2 2 2 2 ...
##  $ Period  : Factor w/ 3 levels "AnteQ","PostQ",..: 1 1 1 1 1 1 1 1 1 1 ..
.
##  $ Blocs   : Factor w/ 8 levels "A","B","D","E",..: 8 8 8 8 8 8 8 8 8 8 ..
.
##  $ layer   : Factor w/ 31 levels "c10","c11","c12",..: 25 25 25 25 25 25 2
5 25 25 26 ...
##  $ ref DMTA: chr  "31" "521" "522" "523" ...
##  $ Asfc    : num  2.118 0.516 2.135 2.424 2.536 ...
##  $ epLsar  : num  6.22 4.77 6.82 5.33 4.43 ...
##  $ Smc     : num  1.361 0.747 0.41 1.361 1.008 ...
##  $ HAsfc9  : num  0.396 0.246 0.369 0.445 0.341 0.308 0.42 0.326 0.269 0.1
48 ...
##  $ HAsfc81 : num  0.563 0.54 0.747 0.838 0.847 0.533 0.638 0.737 0.398 0.4
43 ...
##  $ HAsfc36 : num  0.469 0.428 0.575 0.653 0.697 0.477 0.421 0.499 0.407 0.
339 ...
```

Inter-block differences between contemporaneous ungulates

Red deer from Combe-Grenal: inter-block differences:

```
Ce_species <- Ce %>%
  dplyr::select(c(2)) %>%
  unlist(c(1))

Ce_Period <- Ce %>%
  dplyr::select(c(3)) %>%
  unlist(c(1))
Ce_layer <- Ce %>%
  dplyr::select(c(5)) %>%
  unlist(c(1))
Ce_Blocs <- Ce %>%
  dplyr::select(c(4)) %>%
  unlist(c(1))

Ce_Asfc <- Ce %>%
  dplyr::select(c(7)) %>%
  unlist(c(1))
```

```
Ce_epLsar <- Ce %>%
  dplyr::select(c(8)) %>%
  unlist(c(1))
Ce_Smc <- Ce %>%
  dplyr::select(c(9)) %>%
  unlist(c(1))
Ce_H9 <- Ce %>%
  dplyr::select(c(10)) %>%
  unlist(c(1))
Ce_H36 <- Ce %>%
  dplyr::select(c(12)) %>%
  unlist(c(1))
Ce_H81 <- Ce %>%
  dplyr::select(c(11)) %>%
  unlist(c(1))
```

Data exploration

Search for Zeros

```
sum(Ce_Asfc == 0, na.rm = TRUE) * 100 / nrow(Ce)

## [1] 0

sum(Ce_epLsar == 0, na.rm = TRUE) * 100 / nrow(Ce)

## [1] 0

sum(Ce_Smc == 0, na.rm = TRUE) * 100 / nrow(Ce)

## [1] 0

sum(Ce_H9 == 0, na.rm = TRUE) * 100 / nrow(Ce)

## [1] 0

sum(Ce_H36 == 0, na.rm = TRUE) * 100 / nrow(Ce)

## [1] 0

sum(Ce_H81 == 0, na.rm = TRUE) * 100 / nrow(Ce)

## [1] 0
```

Search for missing data:

```
colSums(is.na(Ce))

##     Group    specie    Period     Blocs     layer ref DMTA      Asfc    epLsar
##         0         0         0         0         0        0         0         0
##       Smc    HAsfc9   HAsfc81   HAsfc36
##         0         0         0         0
```

Checking data distribution and outliers:

```
x <- Ce[order(Ce_Asfc), ]
x$Blocs <- factor(x$Blocs)
dotchart(x$Asfc, cex = 1, pch = 16, groups = x$Blocks, xlab = "Ce_Asfc per Bl
ock")
```

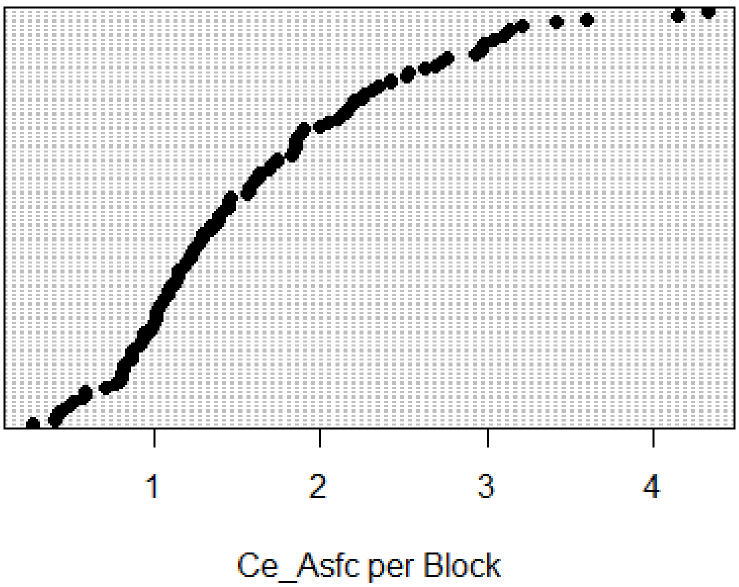


```
x <- Ce[order(Ce_epLsar), ]
x$Blocs <- factor(x$Blocs)
dotchart(x$epLsar, cex = 1, pch = 16, groups = x$Blocks, xlab = "Ce_epLsar pe
r Block")
```

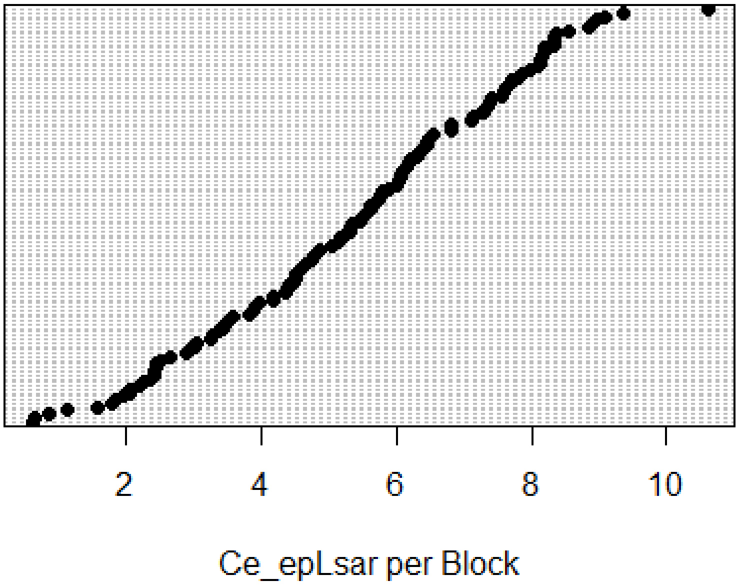


```
x <- Ce[order(Ce_Smc), ]
x$Blocs <- factor(x$Blocs)
dotchart(x$Smc, cex = 1, pch = 16, groups = x$Blocks, xlab = "Ce_Smc per Bloc
k")
```

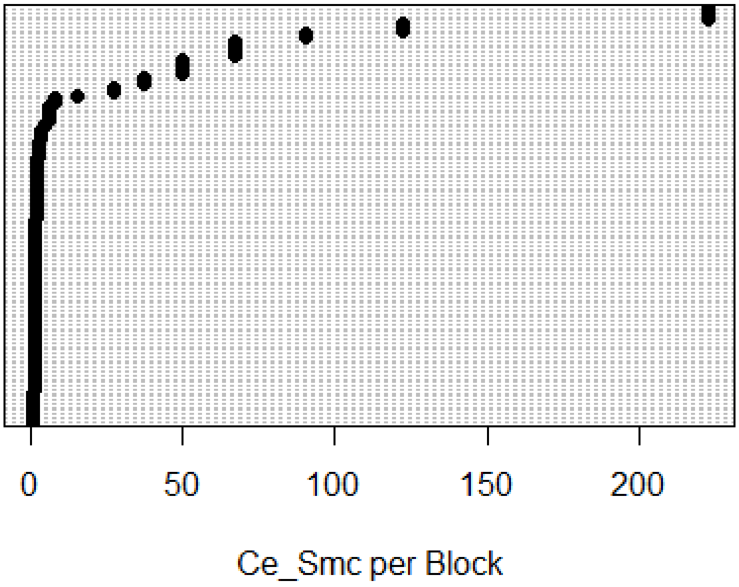


```
x <- Ce[order(Ce_H9), ]
x$Blocs <- factor(x$Blocs)
dotchart(x$HAsfc9, cex = 1, pch = 16, groups = x$Blocks, xlab = "Ce_HAsfc9 pe
r Block")
```

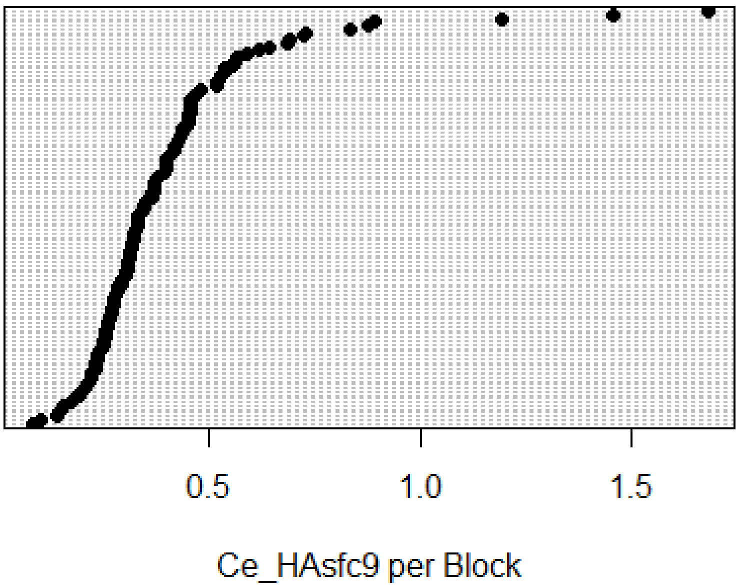


```
x <- Ce[order(Ce_H36), ]
x$Blocs <- factor(x$Blocs)
dotchart(x$HAsfc36, cex = 1, pch = 16, groups = x$Blocks, xlab = "Ce_HAsfc36
per Block")
```

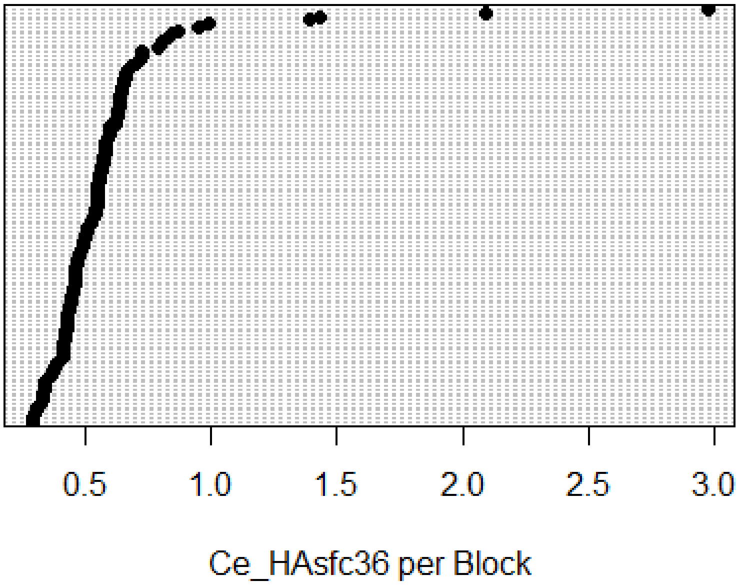


```
x <- Ce[order(Ce_H81), ]
x$Blocs <- factor(x$Blocs)
dotchart(x$HAsfc81, cex = 1, pch = 16, groups = x$Blocks, xlab = "Ce_HAsfc81
per Block")
```

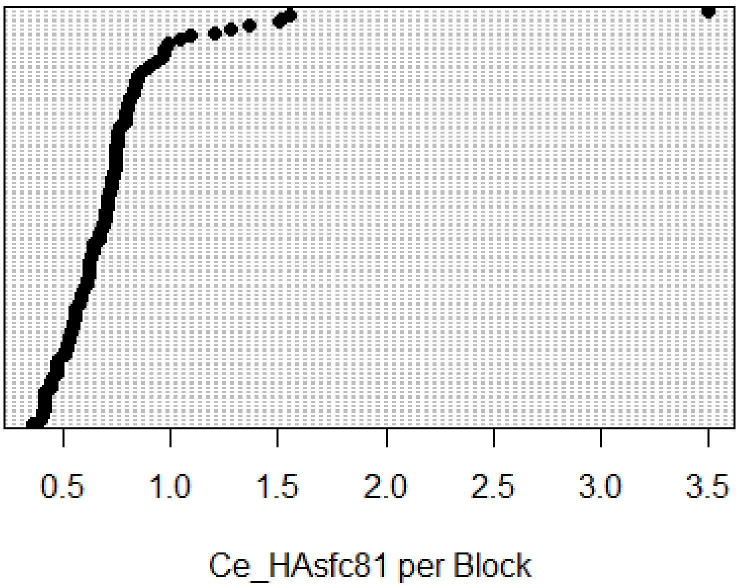

Graphical evaluation of the tests' applicability:

Normality and homoscedasticity of the variables. ##### Normality

```
ggplot(Ce) +
  geom_freqpoly(aes(x = Asfc), bins = 7) +
  labs(
    x = "Value Asfc",
    y = "Frequency"
  )
```

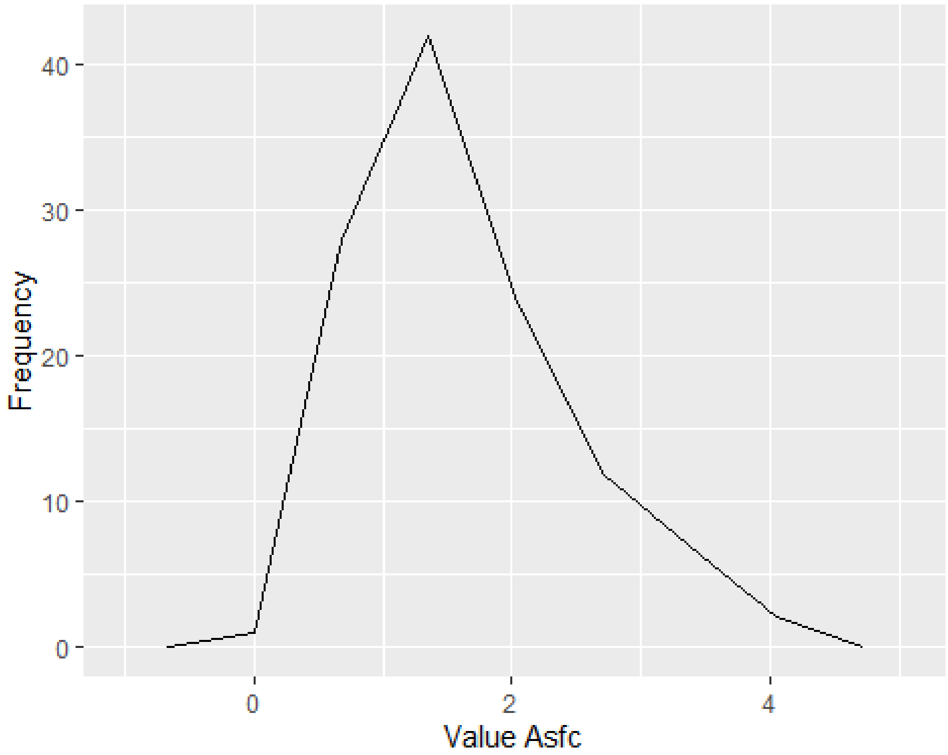


```r
ggplot(Ce) +
  geom_freqpoly(aes(x = epLsar), bins = 7) +
  labs(
    x = "Value epLsar",
    y = "Frequency"
  )
```

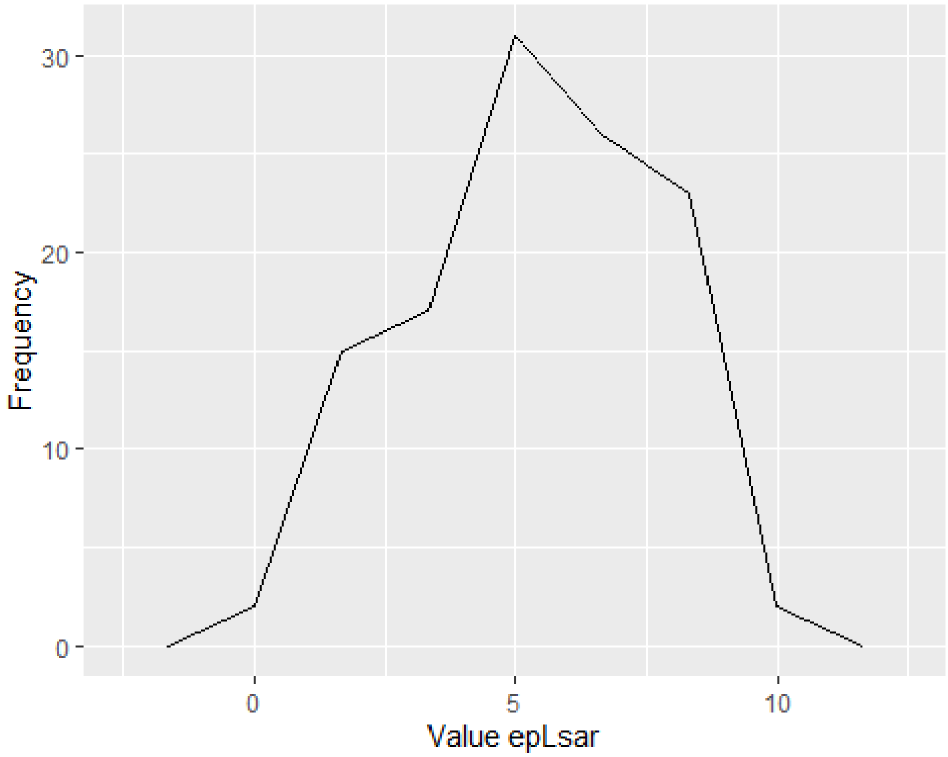


```
ggplot(Ce) +
  geom_freqpoly(aes(x = Smc), bins = 7) +
  labs(
    x = "Value Smc",
    y = "Frequency"
  )
```

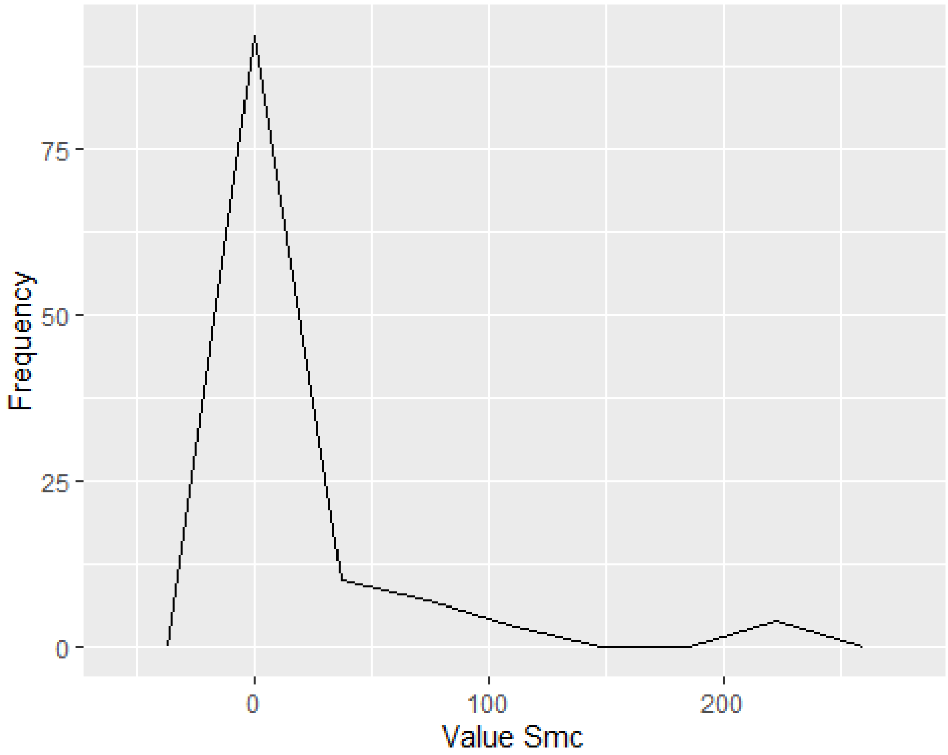


```
ggplot(Ce) +
  geom_freqpoly(aes(x = HAsfc9), bins = 7) +
  labs(
    x = "Value H9",
    y = "Frequency"
  )
```

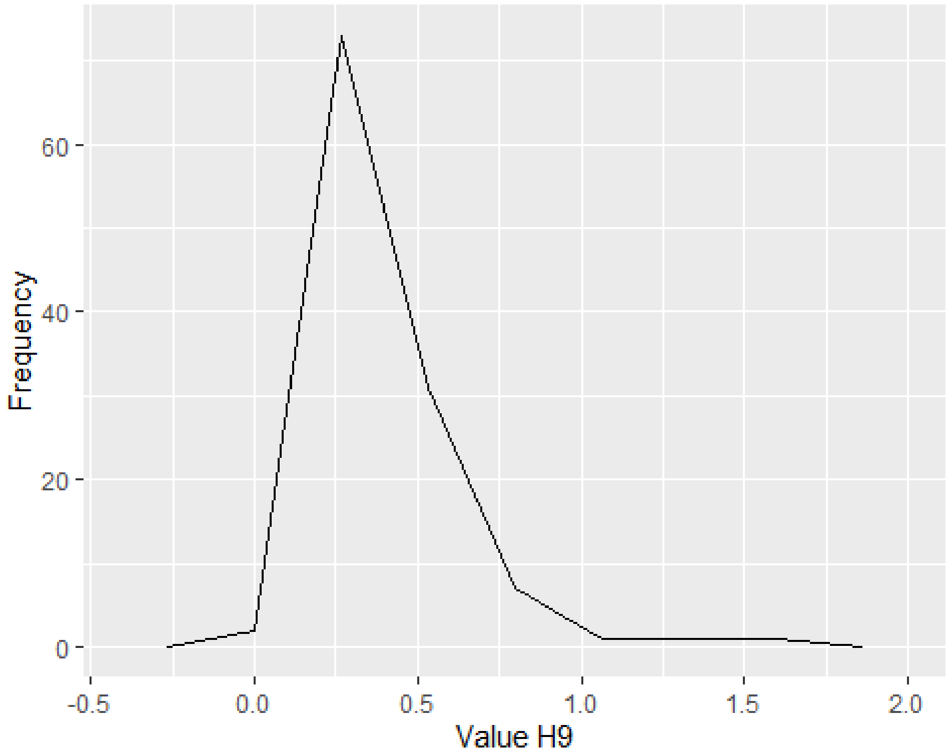


```
ggplot(Ce) +
  geom_freqpoly(aes(x = HAsfc36), bins = 7) +
  labs(
    x = "Value H36",
    y = "Frequency"
  )
```

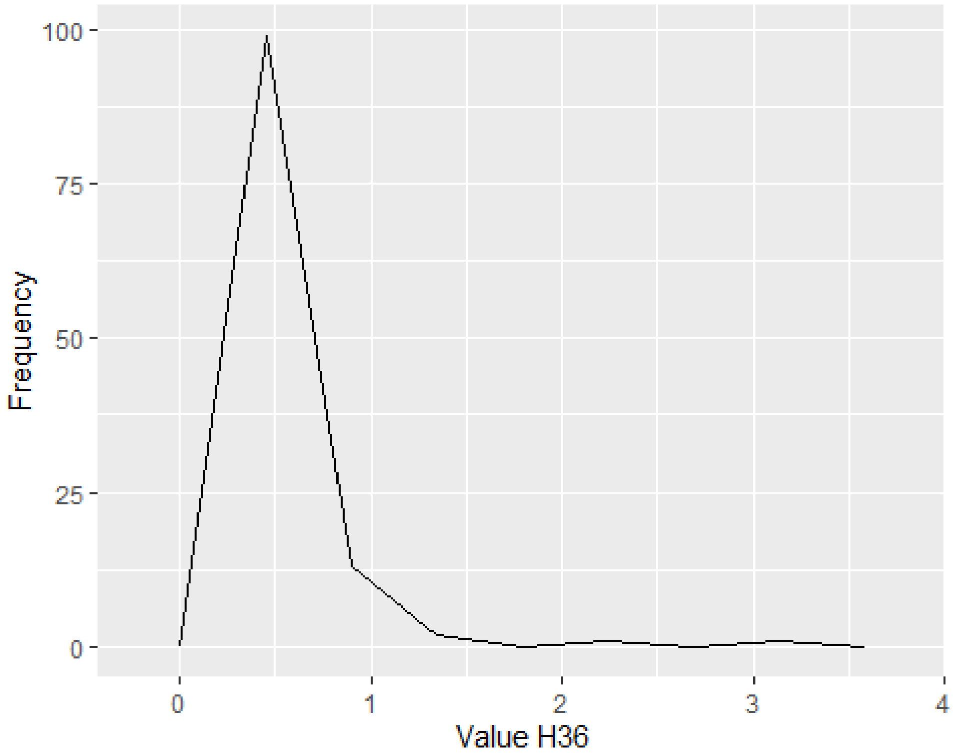


```
ggplot(Ce) +
  geom_freqpoly(aes(x = HAsfc81), bins = 7) +
  labs(
    x = "Value H81",
    y = "Frequency"
  )
```

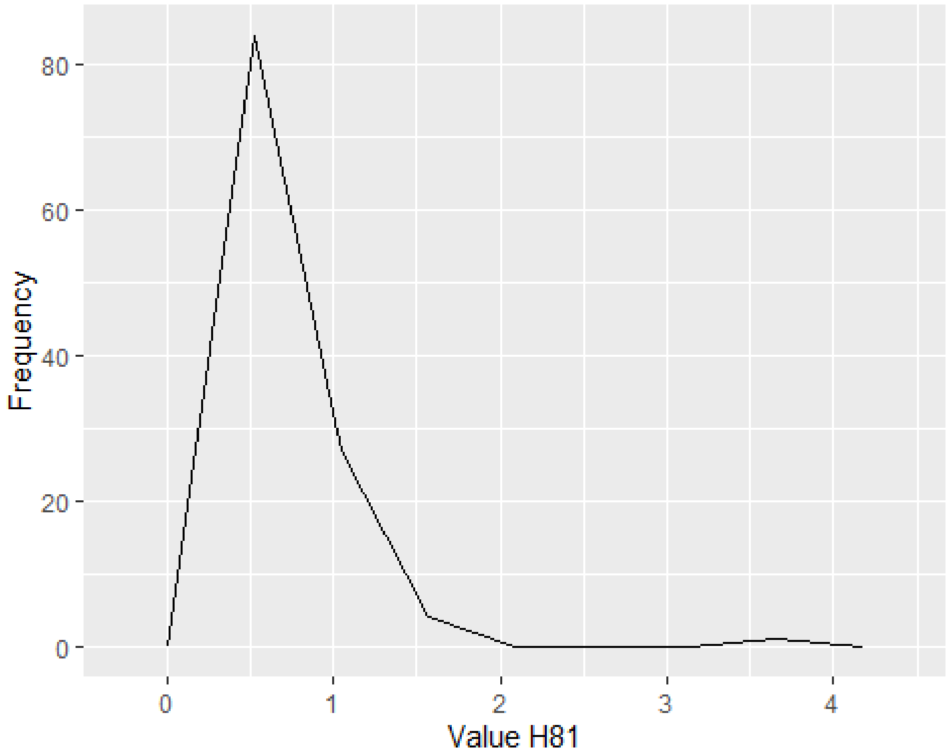

Homoscedasticity: Brown & Forsythe test (and data transformation whenever needed)

```
bf.test(Ce_Asfc ~ Ce_Blocs, data = Ce)

##
##   Brown-Forsythe Test (alpha = 0.05)
## -------------------------------------------------------------
##   data : Ce_Asfc and Ce_Blocs
##
##   statistic  : 1.748979
##   num df     : 7
##   denom df   : 19.90943
##   p.value    : 0.154503
##
##   Result     : Difference is not statistically significant.
## -------------------------------------------------------------

ggplot(Ce) +
  geom_boxplot(aes(x = Ce_Blocs, y = Ce_Asfc)) +
  labs(
    x = "Blocks",
    y = "Asfc"
  )
```

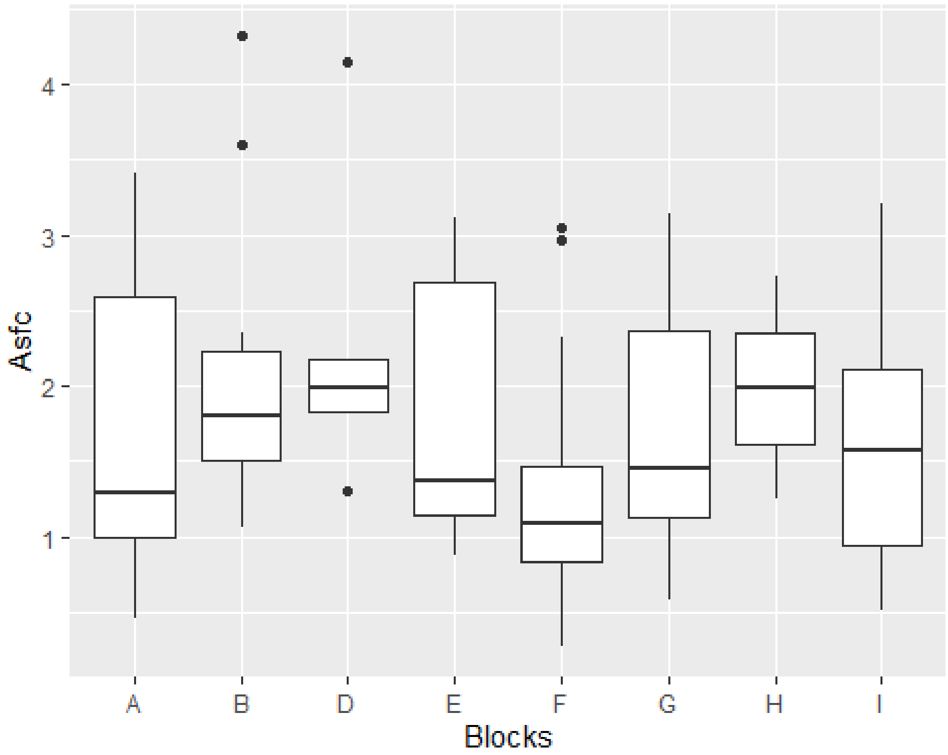


```
bf.test(Ce_epLsar ~ Ce_Blocs, data = Ce)

##
##   Brown-Forsythe Test (alpha = 0.05)
## -------------------------------------------------------------
##   data : Ce_epLsar and Ce_Blocs
##
##   statistic  : 1.673001
##   num df     : 7
##   denom df   : 43.89027
##   p.value    : 0.1407834
##
##   Result     : Difference is not statistically significant.
## -------------------------------------------------------------

ggplot(Ce) +
  geom_boxplot(aes(x = Ce_Blocs, y = Ce_epLsar)) +
  labs(
    x = "Blocks",
    y = "epLsar"
  )
```

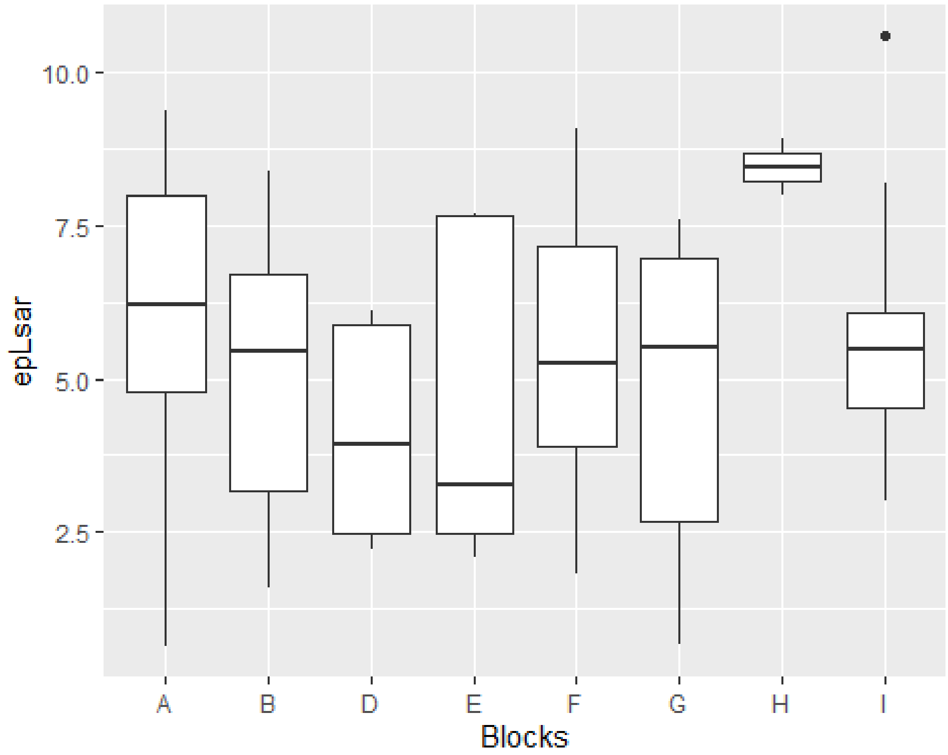


```
bf.test(Ce_Smc ~ Ce_Blocs, data = Ce)

##
##   Brown-Forsythe Test (alpha = 0.05)
## -------------------------------------------------------------
##   data : Ce_Smc and Ce_Blocs
##
##   statistic  : 2.205481
##   num df     : 7
##   denom df   : 39.85731
##   p.value    : 0.05427927
##
##   Result     : Difference is not statistically significant.
## -------------------------------------------------------------

ggplot(Ce) +
  geom_boxplot(aes(x = Ce_Blocs, y = Ce_Smc)) +
  labs(
    x = "Blocks",
    y = "Smc"
  )
```

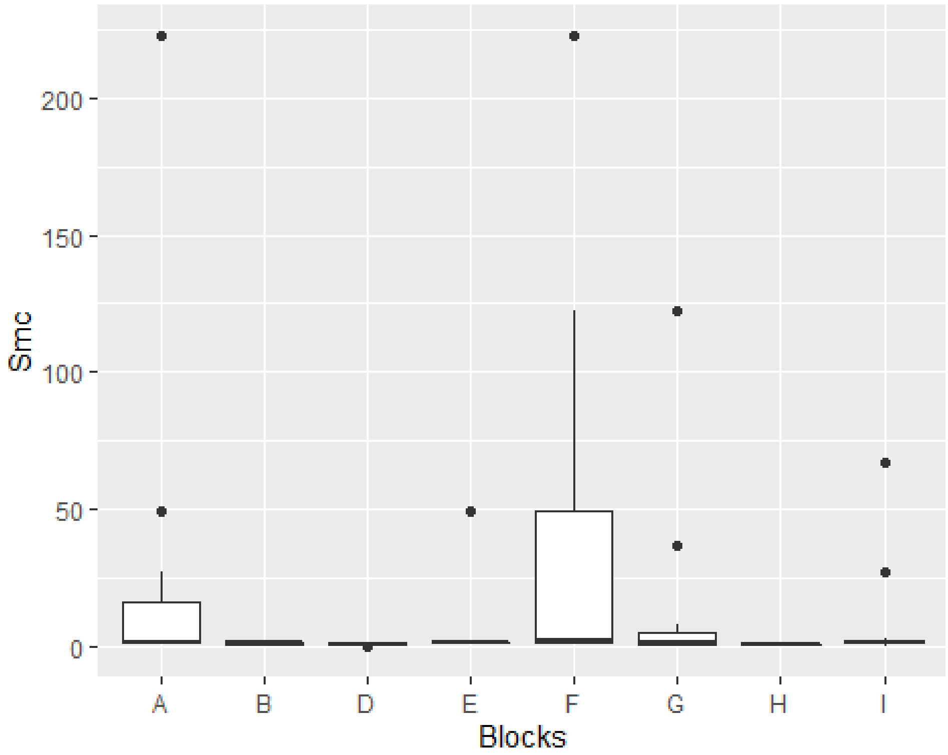


```
bf.test(Ce_H9 ~ Ce_Blocs, data = Ce)

##
##   Brown-Forsythe Test (alpha = 0.05)
## -------------------------------------------------------------
##   data : Ce_H9 and Ce_Blocs
##
##   statistic  : 1.854374
##   num df     : 7
##   denom df   : 52.75351
##   p.value    : 0.09612489
##
##   Result     : Difference is not statistically significant.
## -------------------------------------------------------------

ggplot(Ce) +
  geom_boxplot(aes(x = Ce_Blocs, y = Ce_H9)) +
  labs(
    x = "Blocks",
    y = "H9"
  )
```

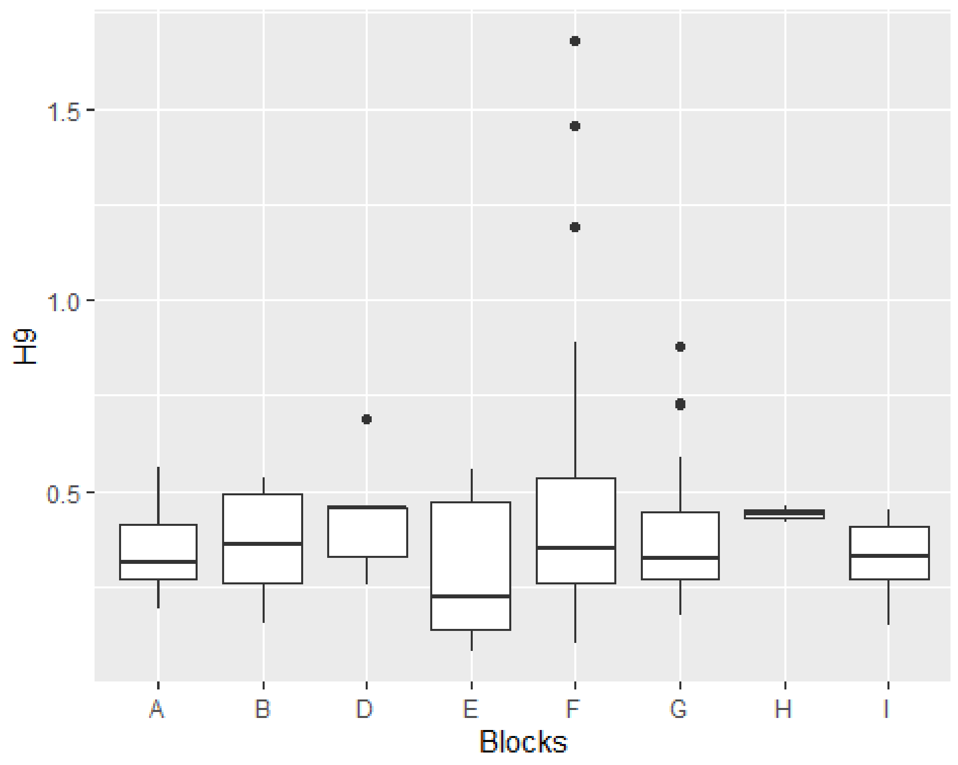


```
bf.test(unlist(Ce_H36) ~ unlist(Ce_Blocs), data = Ce)

##
##   Brown-Forsythe Test (alpha = 0.05)
## -------------------------------------------------------------
##   data : unlist(Ce_H36) and unlist(Ce_Blocs)
##
##   statistic  : 2.022587
##   num df     : 7
##   denom df   : 71.64772
##   p.value    : 0.06382101
##
##   Result     : Difference is not statistically significant.
## -------------------------------------------------------------

ggplot(Ce) +
  geom_boxplot(aes(x = Ce_Blocs, y = Ce_H36)) +
  labs(
    x = "Blocks",
    y = "H36"
  )
```

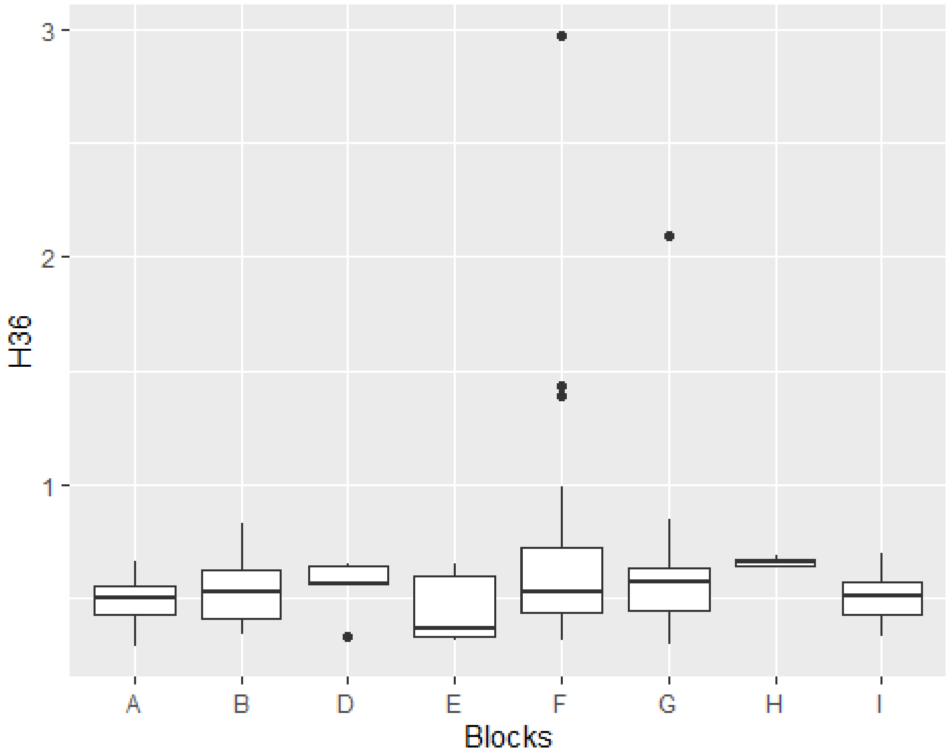


```
bf.test(unlist(Ce_H81) ~ unlist(Ce_Blocs), data = Ce)

##
##   Brown-Forsythe Test (alpha = 0.05)
## -------------------------------------------------------------
##   data : unlist(Ce_H81) and unlist(Ce_Blocs)
##
##   statistic  : 3.044767
##   num df     : 7
##   denom df   : 75.33016
##   p.value    : 0.007097534
##
##   Result     : Difference is statistically significant.
## -------------------------------------------------------------

ggplot(Ce) +
  geom_boxplot(aes(x = Ce_Blocs, y = Ce_H81)) +
  labs(
    x = "Blocks",
    y = "H81"
  )
```

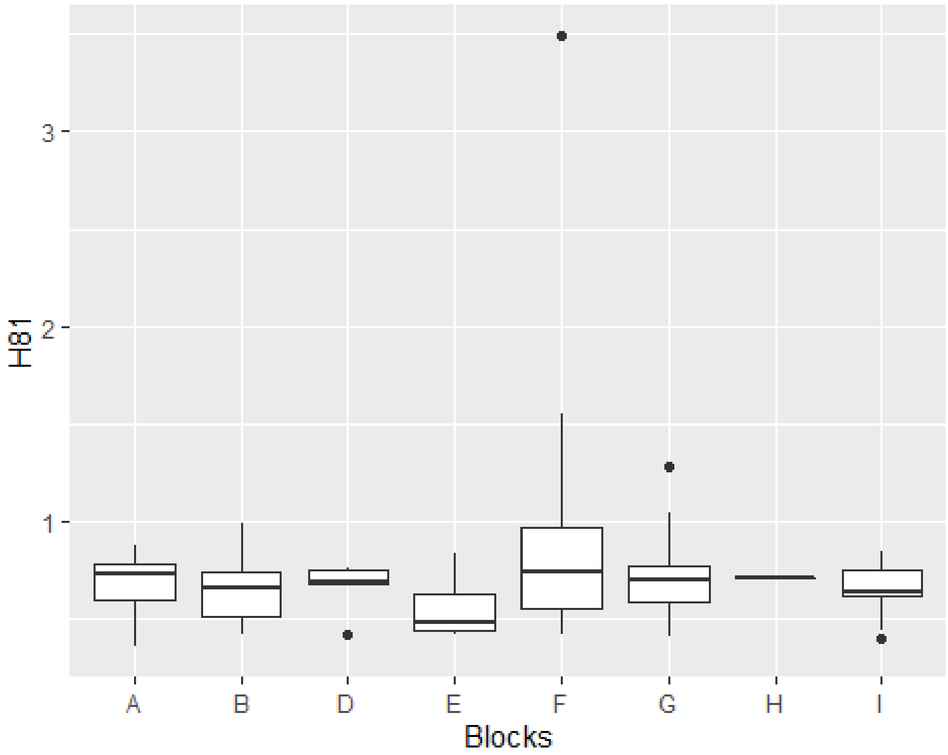


```r
GLM_Ce_H81 <- glm(formula = Ce_H81 ~ Ce_Blocs, family = gaussian)
bc_Ce_H81 <- boxcox(GLM_Ce_H81, lambda = seq(-3, 3))
```

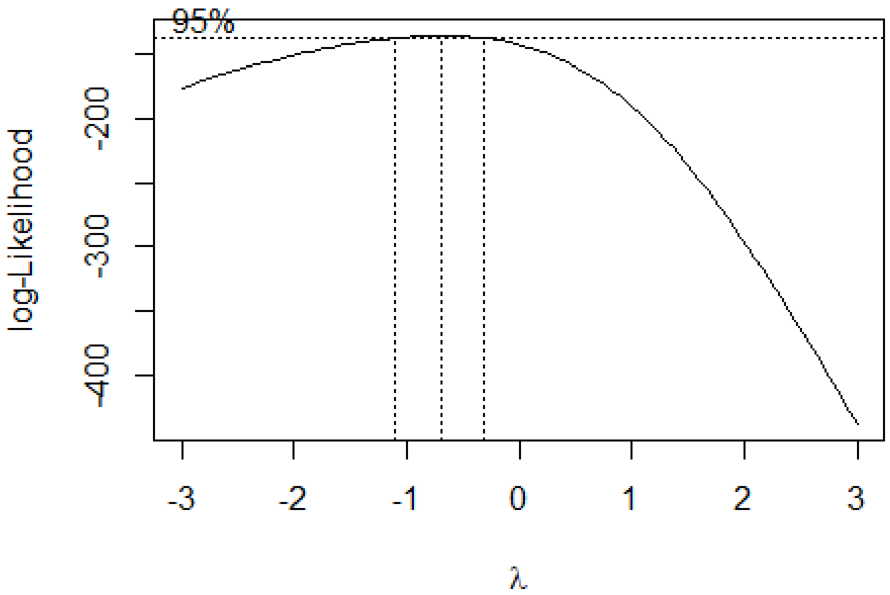

```
best.lam <- bc_Ce_H81$x[which(bc_Ce_H81$y == max(bc_Ce_H81$y))]
best.lam # -0.6969697

## [1] -0.6969697

new_Ce_H81 <- (Ce_H81)^-0.6969697
bf.test(new_Ce_H81 ~ Ce_Blocs, data = Ce) # Difference is not statistically s
ignificant.

##
##   Brown-Forsythe Test (alpha = 0.05)
## -------------------------------------------------------------
##   data : new_Ce_H81 and Ce_Blocs
##
##   statistic  : 1.387393
##   num df     : 7
##   denom df   : 51.45728
##   p.value    : 0.2307317
##
##   Result     : Difference is not statistically significant.
## -------------------------------------------------------------

ggplot(Ce) +
  geom_boxplot(aes(x = Ce_Blocs, y = new_Ce_H81)) +
  labs(
    x = "Blocks",
    y = "H81"
  )
```

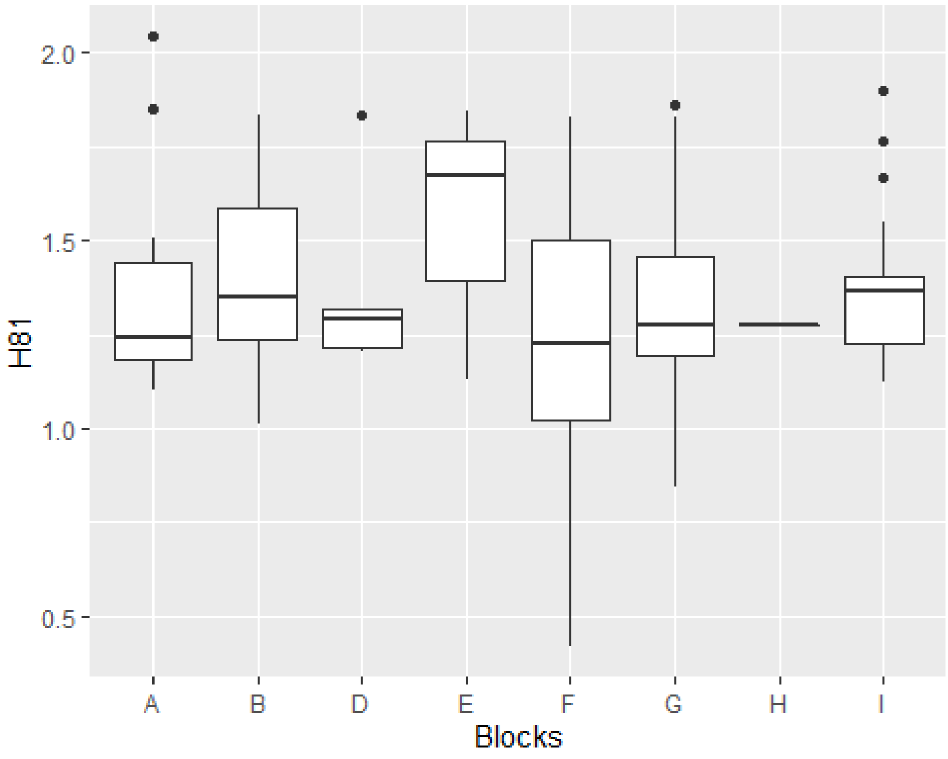

```r
Ce_H81 <- (Ce_H81)^-0.6969697

rm(bx1Asfc, bx1epLsar, bx1Smc, bx1H9, bx1H36, bx1H81)

bf.test(unlist(Ce_Asfc) ~ unlist(Ce_Period), data = Ce)

##
##   Brown-Forsythe Test (alpha = 0.05)
## -------------------------------------------------------------
##   data : unlist(Ce_Asfc) and unlist(Ce_Period)
##
##   statistic  : 2.463095
##   num df     : 2
##   denom df   : 82.55081
##   p.value    : 0.09140984
##
##   Result     : Difference is not statistically significant.
## -------------------------------------------------------------

ggplot(Ce) +
  geom_boxplot(aes(x = Ce_Period, y = Ce_Asfc)) +
  labs(
    x = "Period",
    y = "Asfc"
  )
```

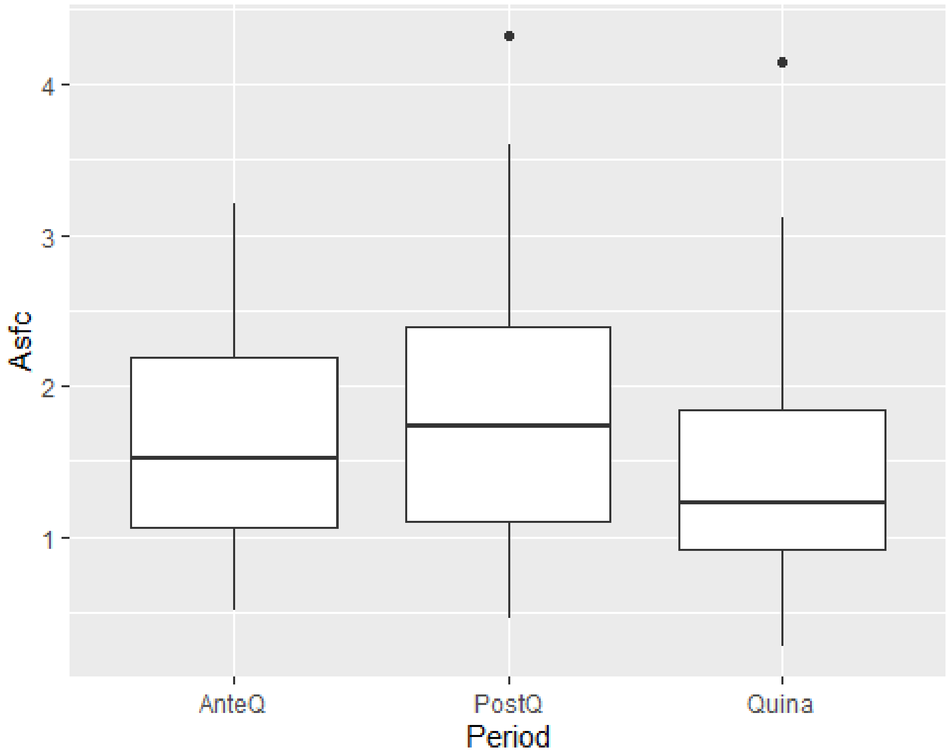


```r
bf.test(unlist(Ce_epLsar) ~ unlist(Ce_Period), data = Ce)
```

```
##
##   Brown-Forsythe Test (alpha = 0.05)
## -------------------------------------------------------------
##   data : unlist(Ce_epLsar) and unlist(Ce_Period)
##
##   statistic  : 0.5517372
##   num df     : 2
##   denom df   : 94.60729
##   p.value    : 0.5777903
##
##   Result     : Difference is not statistically significant.
## -------------------------------------------------------------
```

```
ggplot(Ce) +
  geom_boxplot(aes(x = Ce_Period, y = Ce_epLsar)) +
  labs(
    x = "Period",
    y = "epLsar"
  )
```

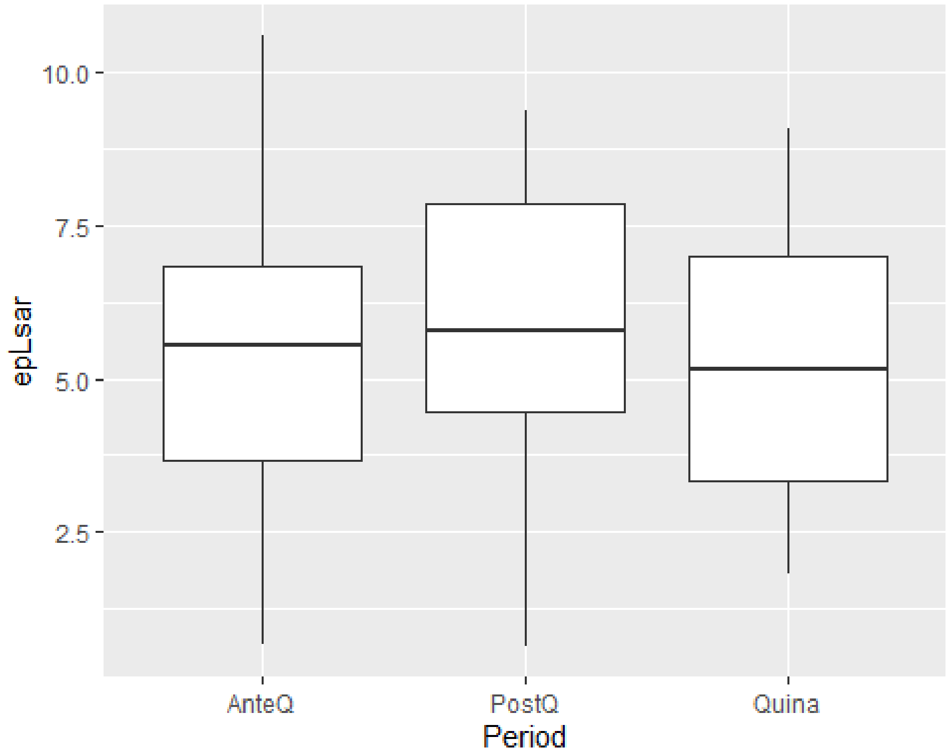


```
bf.test(unlist(Ce_Smc) ~ unlist(Ce_Period), data = Ce)

##
##   Brown-Forsythe Test (alpha = 0.05)
## -------------------------------------------------------------
##   data : unlist(Ce_Smc) and unlist(Ce_Period)
##
```

```
##   statistic  : 1.111591
##   num df     : 2
##   denom df   : 68.08676
##   p.value    : 0.3349316
##
##   Result     : Difference is not statistically significant.
## -------------------------------------------------------------

ggplot(Ce) +
  geom_boxplot(aes(x = Ce_Period, y = Ce_Smc)) +
  labs(
    x = "Period",
    y = "Smc"
  )
```

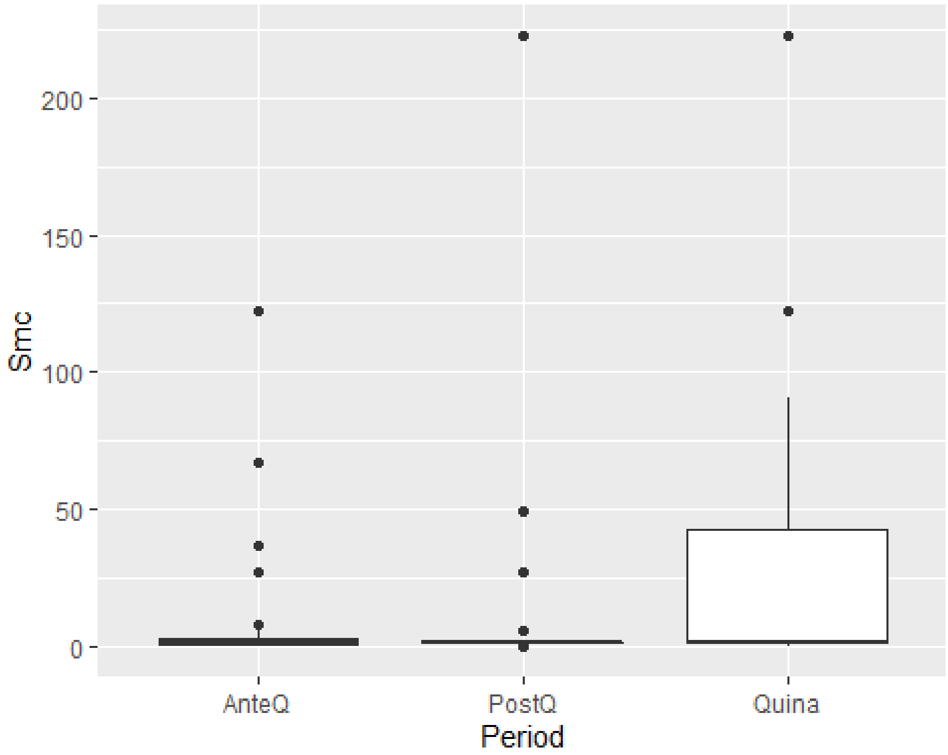


```
bf.test(unlist(Ce_H9) ~ unlist(Ce_Period), data = Ce)

##
##   Brown-Forsythe Test (alpha = 0.05)
## -------------------------------------------------------------
##   data : unlist(Ce_H9) and unlist(Ce_Period)
##
##   statistic  : 2.486296
##   num df     : 2
##   denom df   : 81.56737
##   p.value    : 0.08950547
##
```

```
##   Result      : Difference is not statistically significant.
## -------------------------------------------------------------

ggplot(Ce) +
  geom_boxplot(aes(x = Ce_Period, y = Ce_H9)) +
  labs(
    x = "Period",
    y = "H9"
  )
```

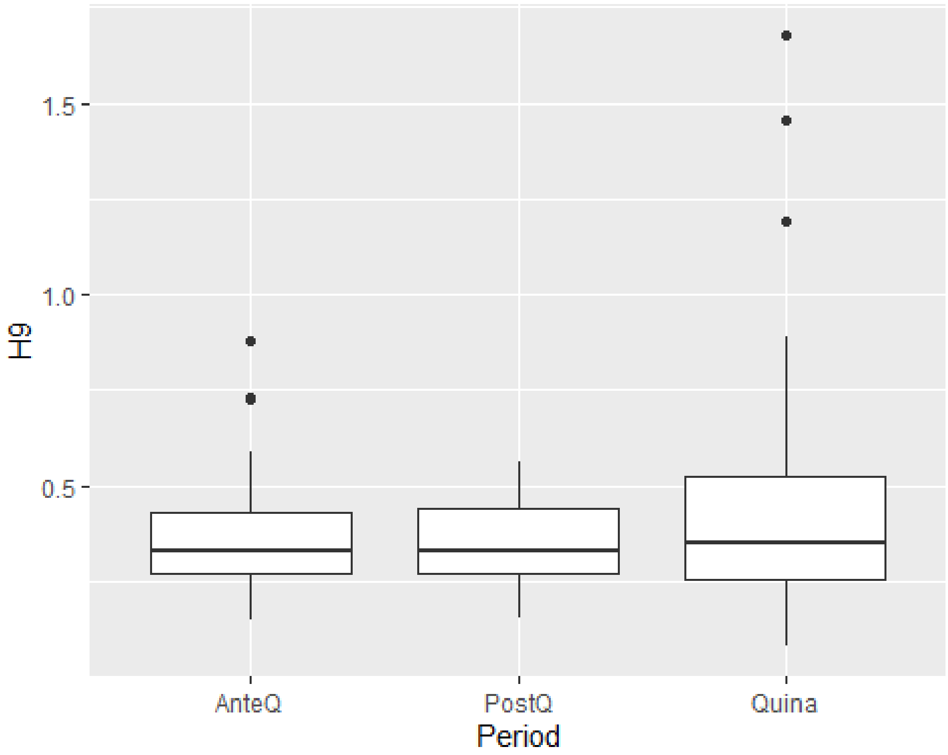


```
bf.test(unlist(Ce_H36) ~ unlist(Ce_Period), data = Ce)

##
##   Brown-Forsythe Test (alpha = 0.05)
## -------------------------------------------------------------
##   data : unlist(Ce_H36) and unlist(Ce_Period)
##
##   statistic   : 1.721607
##   num df      : 2
##   denom df    : 89.72958
##   p.value     : 0.184631
##
##   Result      : Difference is not statistically significant.
## -------------------------------------------------------------

ggplot(Ce) +
  geom_boxplot(aes(x = Ce_Period, y = Ce_H36)) +
  labs(
```

```
    x = "Period",
    y = "H36"
  )
```

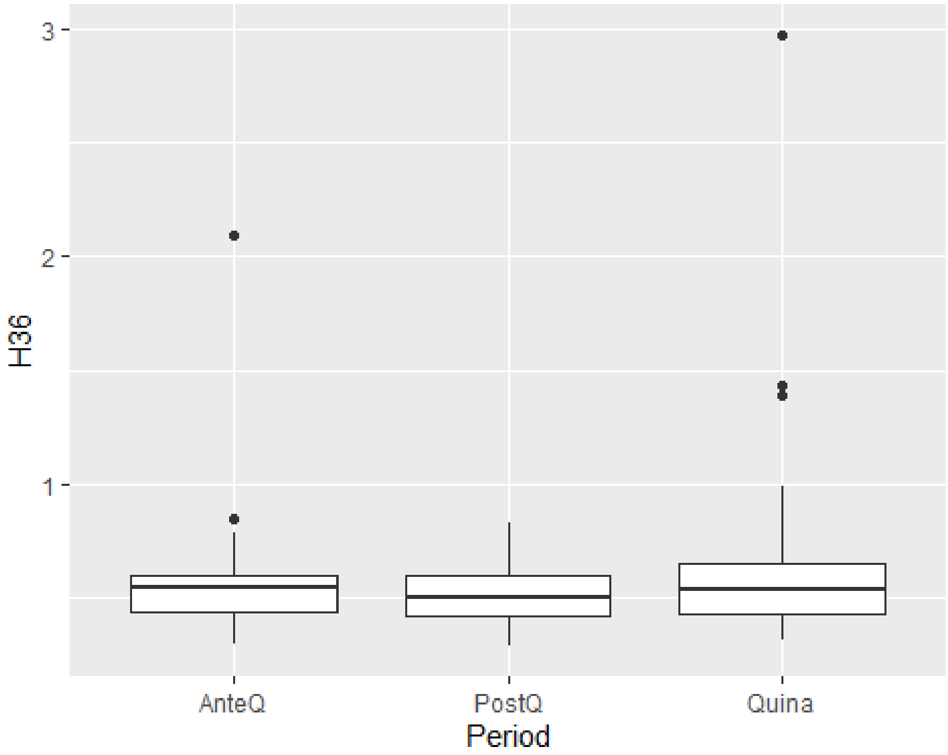


```
bf.test(unlist(Ce_H81) ~ unlist(Ce_Period), data = Ce)

##
##   Brown-Forsythe Test (alpha = 0.05)
## -------------------------------------------------------------
##   data : unlist(Ce_H81) and unlist(Ce_Period)
##
##   statistic  : 0.7872617
##   num df     : 2
##   denom df   : 102.0599
##   p.value    : 0.457833
##
##   Result     : Difference is not statistically significant.
## -------------------------------------------------------------

ggplot(Ce) +
  geom_boxplot(aes(x = Ce_Period, y = Ce_H81)) +
  labs(
    x = "Period",
    y = "H81"
  )
```

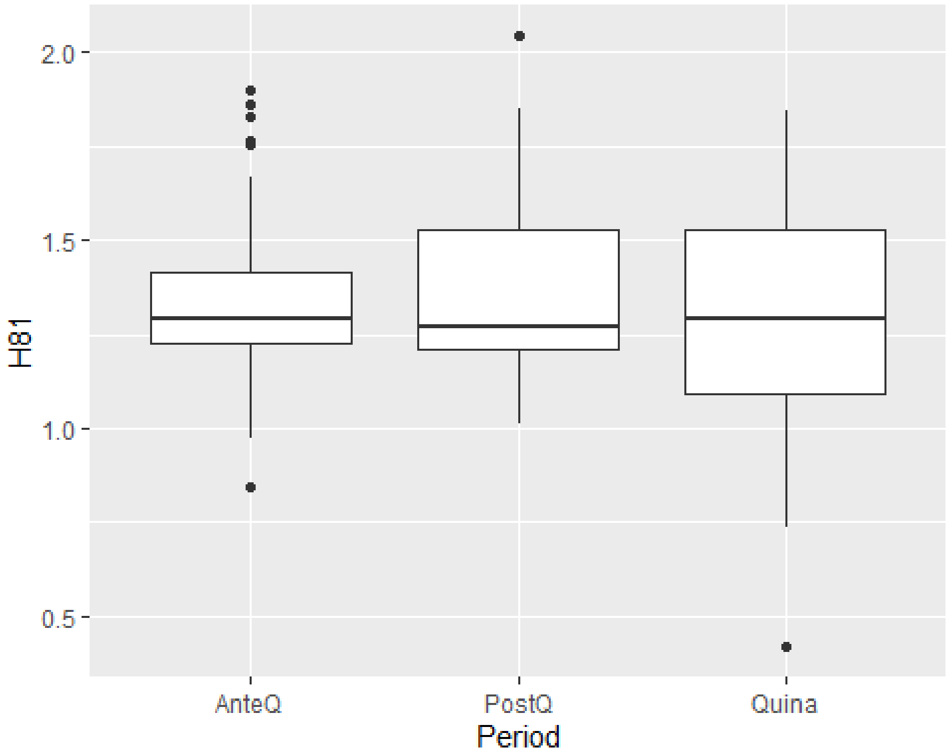

GLM analyses

What is the impact of random (1), blocks (Ce_Blocks) and period (Ce_Period) over each DMTA parameter?

```
glm_Ce_Asfc0 <- glm(Ce_Asfc ~ 1, data = Ce)
glm_Ce_Asfc1 <- glm(Ce_Asfc ~ Ce_Blocs, data = Ce)
glm_Ce_Asfc2 <- glm(Ce_Asfc ~ Ce_Period, data = Ce)
glm_Ce_Asfc3 <- glm(Ce_Asfc ~ Ce_layer, data = Ce)

Cand.models <- list()
Cand.models[[1]] <- glm_Ce_Asfc0
Cand.models[[2]] <- glm_Ce_Asfc1
Cand.models[[3]] <- glm_Ce_Asfc2
Cand.models[[4]] <- glm_Ce_Asfc3
Modnames <- lapply(Cand.models, "formula")
aictab(cand.set = Cand.models, modnames = paste0(Modnames), sort = TRUE)

##
## Model selection based on AICc:
##
##                         K   AICc Delta_AICc AICcWt Cum.Wt      LL
## Ce_Asfc ~ Ce_Period  4 291.31       0.00   0.41   0.41 -141.47
## Ce_Asfc ~ Ce_Blocs   9 291.69       0.38   0.34   0.75 -136.00
## Ce_Asfc ~ 1          2 292.32       1.01   0.25   1.00 -144.11
## Ce_Asfc ~ Ce_layer  27 310.86      19.55   0.00   1.00 -119.84
```

```
summary(glm_Ce_Asfc2)

##
## Call:
## glm(formula = Ce_Asfc ~ Ce_Period, data = Ce)
##
## Deviance Residuals:
##     Min       1Q   Median       3Q      Max
## -1.4129  -0.5890  -0.1875   0.4780   2.7225
##
## Coefficients:
##                 Estimate Std. Error t value Pr(>|t|)
## (Intercept)       1.6575     0.1281  12.940   <2e-16 ***
## Ce_PeriodPostQ    0.2145     0.2048   1.047    0.297
## Ce_PeriodQuina   -0.2360     0.1763  -1.339    0.183
## ---
## Signif. codes:  0 '***' 0.001 '**' 0.01 '*' 0.05 '.' 0.1 ' ' 1
##
## (Dispersion parameter for gaussian family taken to be 0.6890195)
##
##     Null deviance: 81.476  on 115  degrees of freedom
## Residual deviance: 77.859  on 113  degrees of freedom
## AIC: 290.95
##
## Number of Fisher Scoring iterations: 2

marginal <- emmeans(glm_Ce_Asfc2, ~Ce_Period)
pairs(marginal)

##  contrast      estimate    SE  df t.ratio p.value
##  AnteQ - PostQ   -0.214 0.205 113  -1.047  0.5486
##  AnteQ - Quina    0.236 0.176 113   1.339  0.3767
##  PostQ - Quina    0.450 0.200 113   2.247  0.0677
##
## P value adjustment: tukey method for comparing a family of 3 estimates

glm_Ce_epLsar0 <- glm(Ce_epLsar ~ 1, data = Ce)
glm_Ce_epLsar1 <- glm(Ce_epLsar ~ Ce_Blocs, data = Ce)
glm_Ce_epLsar2 <- glm(Ce_epLsar ~ Ce_Period, data = Ce)
glm_Ce_epLsar3 <- glm(Ce_epLsar ~ Ce_layer, data = Ce)
Cand.models <- list()
Cand.models[[1]] <- glm_Ce_epLsar0
Cand.models[[2]] <- glm_Ce_epLsar1
Cand.models[[3]] <- glm_Ce_epLsar2
Cand.models[[4]] <- glm_Ce_epLsar3
Modnames <- lapply(Cand.models, "formula")
aictab(cand.set = Cand.models, modnames = paste0(Modnames), sort = TRUE)

##
## Model selection based on AICc:
##
```

```
##                          K   AICc Delta_AICc AICcWt Cum.Wt      LL
## Ce_epLsar ~ 1            2 520.02       0.00   0.76   0.76 -257.96
## Ce_epLsar ~ Ce_Period    4 523.12       3.10   0.16   0.92 -257.38
## Ce_epLsar ~ Ce_Blocs     9 524.40       4.38   0.08   1.00 -252.35
## Ce_epLsar ~ Ce_layer    27 559.91      39.89   0.00   1.00 -244.36

glm_Ce_Smc0 <- glm(Ce_Smc ~ 1, data = Ce)
glm_Ce_Smc1 <- glm(Ce_Smc ~ Ce_Blocs, data = Ce)
glm_Ce_Smc2 <- glm(Ce_Smc ~ Ce_Period, data = Ce)
glm_Ce_Smc3 <- glm(Ce_Smc ~ Ce_layer, data = Ce)
Cand.models <- list()
Cand.models[[1]] <- glm_Ce_Smc0
Cand.models[[2]] <- glm_Ce_Smc1
Cand.models[[3]] <- glm_Ce_Smc2
Cand.models[[4]] <- glm_Ce_Smc3
Modnames <- lapply(Cand.models, "formula")
aictab(cand.set = Cand.models, modnames = paste0(Modnames), sort = TRUE)

## 
## Model selection based on AICc:
## 
##                       K    AICc Delta_AICc AICcWt Cum.Wt      LL
## Ce_Smc ~ 1            2 1224.59       0.00   0.68   0.68 -610.24
## Ce_Smc ~ Ce_Period    4 1226.37       1.78   0.28   0.96 -609.00
## Ce_Smc ~ Ce_Blocs     9 1230.37       5.78   0.04   1.00 -605.34
## Ce_Smc ~ Ce_layer    27 1256.90      32.31   0.00   1.00 -592.86

glm_Ce_H9_0 <- glm(Ce_H9 ~ 1, data = Ce)
glm_Ce_H9_1 <- glm(Ce_H9 ~ Ce_Blocs, data = Ce)
glm_Ce_H9_2 <- glm(Ce_H9 ~ Ce_Period, data = Ce)
glm_Ce_H9_3 <- glm(Ce_H9 ~ Ce_layer, data = Ce)
Cand.models <- list()
Cand.models[[1]] <- glm_Ce_H9_0
Cand.models[[2]] <- glm_Ce_H9_1
Cand.models[[3]] <- glm_Ce_H9_2
Cand.models[[4]] <- glm_Ce_H9_3
Modnames <- lapply(Cand.models, "formula")
aictab(cand.set = Cand.models, modnames = paste0(Modnames), sort = TRUE)

## 
## Model selection based on AICc:
## 
##                      K  AICc Delta_AICc AICcWt Cum.Wt    LL
## Ce_H9 ~ 1            2 -5.95       0.00   0.52   0.52  5.03
## Ce_H9 ~ Ce_Period    4 -5.76       0.19   0.47   0.99  7.06
## Ce_H9 ~ Ce_Blocs     9  1.94       7.89   0.01   1.00  8.88
## Ce_H9 ~ Ce_layer    27 26.65      32.60   0.00   1.00 22.27

glm_Ce_H36_0 <- glm(Ce_H36 ~ 1, data = Ce)
glm_Ce_H36_1 <- glm(Ce_H36 ~ Ce_Blocs, data = Ce)
glm_Ce_H36_2 <- glm(Ce_H36 ~ Ce_Period, data = Ce)
```

```
glm_Ce_H36_3 <- glm(Ce_H36 ~ Ce_layer, data = Ce)
Cand.models <- list()
Cand.models[[1]] <- glm_Ce_H36_0
Cand.models[[2]] <- glm_Ce_H36_1
Cand.models[[3]] <- glm_Ce_H36_2
Cand.models[[4]] <- glm_Ce_H36_3
Modnames <- lapply(Cand.models, "formula")
aictab(cand.set = Cand.models, modnames = paste0(Modnames), sort = TRUE)

##
## Model selection based on AICc:
##
##                      K   AICc Delta_AICc AICcWt Cum.Wt     LL
## Ce_H36 ~ 1           2  69.99       0.00   0.67   0.67 -32.94
## Ce_H36 ~ Ce_Period   4  71.45       1.45   0.32   0.99 -31.54
## Ce_H36 ~ Ce_Blocs    9  78.48       8.49   0.01   1.00 -29.39
## Ce_H36 ~ Ce_layer   27 100.87      30.87   0.00   1.00 -14.84

glm_Ce_H81_0 <- glm(Ce_H81 ~ 1, data = Ce)
glm_Ce_H81_1 <- glm(Ce_H81 ~ Ce_Blocs, data = Ce)
glm_Ce_H81_2 <- glm(Ce_H81 ~ Ce_Period, data = Ce)
glm_Ce_H81_3 <- glm(Ce_H81 ~ Ce_layer, data = Ce)
Cand.models <- list()
Cand.models[[1]] <- glm_Ce_H81_0
Cand.models[[2]] <- glm_Ce_H81_1
Cand.models[[3]] <- glm_Ce_H81_2
Cand.models[[4]] <- glm_Ce_H81_3
Modnames <- lapply(Cand.models, "formula")
aictab(cand.set = Cand.models, modnames = paste0(Modnames), sort = TRUE)

##
## Model selection based on AICc:
##
##                      K  AICc Delta_AICc AICcWt Cum.Wt     LL
## Ce_H81 ~ 1           2 39.68       0.00   0.78   0.78 -17.79
## Ce_H81 ~ Ce_Period   4 42.41       2.73   0.20   0.98 -17.03
## Ce_H81 ~ Ce_Blocs    9 47.22       7.53   0.02   1.00 -13.76
## Ce_H81 ~ Ce_layer   27 70.59      30.91   0.00   1.00   0.29
```

Reindeer from Combe-Grenal: inter-block differences:

```
Rt_species <- Rt %>%
  dplyr::select(c(2)) %>%
  unlist(c(1))

Rt_Period <- Rt %>%
  dplyr::select(c(3)) %>%
  unlist(c(1))
Rt_layer <- Rt %>%
  dplyr::select(c(5)) %>%
  unlist(c(1))
Rt_Blocs <- Rt %>%
```

```
  dplyr::select(c(4)) %>%
  unlist(c(1))

Rt_Asfc <- Rt %>%
  dplyr::select(c(7)) %>%
  unlist(c(1))
Rt_epLsar <- Rt %>%
  dplyr::select(c(8)) %>%
  unlist(c(1))
Rt_Smc <- Rt %>%
  dplyr::select(c(9)) %>%
  unlist(c(1))
Rt_H9 <- Rt %>%
  dplyr::select(c(10)) %>%
  unlist(c(1))
Rt_H36 <- Rt %>%
  dplyr::select(c(12)) %>%
  unlist(c(1))
Rt_H81 <- Rt %>%
  dplyr::select(c(11)) %>%
  unlist(c(1))
```

Data exploration

Search for Zeros

```
sum(Rt_Asfc == 0, na.rm = TRUE) * 100 / nrow(Rt)

## [1] 0

sum(Rt_epLsar == 0, na.rm = TRUE) * 100 / nrow(Rt)

## [1] 0

sum(Rt_Smc == 0, na.rm = TRUE) * 100 / nrow(Rt)

## [1] 0

sum(Rt_H9 == 0, na.rm = TRUE) * 100 / nrow(Rt)

## [1] 0

sum(Rt_H36 == 0, na.rm = TRUE) * 100 / nrow(Rt)

## [1] 0

sum(Rt_H81 == 0, na.rm = TRUE) * 100 / nrow(Rt)

## [1] 0
```

Search for missing data:

```
colSums(is.na(Rt))
```

```
##     Group    specie    Period     Blocs     layer ref DMTA      Asfc    epLsar
##         0         0         0         0         0         0         0         0
##       Smc    HAsfc9   HAsfc81   HAsfc36
##         0         0         0         0
```

Checking data distribution and outliers:

```
x <- Rt[order(Rt_Asfc), ]
x$Blocs <- factor(x$Blocs)
dotchart(x$Asfc, cex = 1, pch = 16, groups = x$Blocks, xlab = "Rt_Asfc per Bl
ock")
```

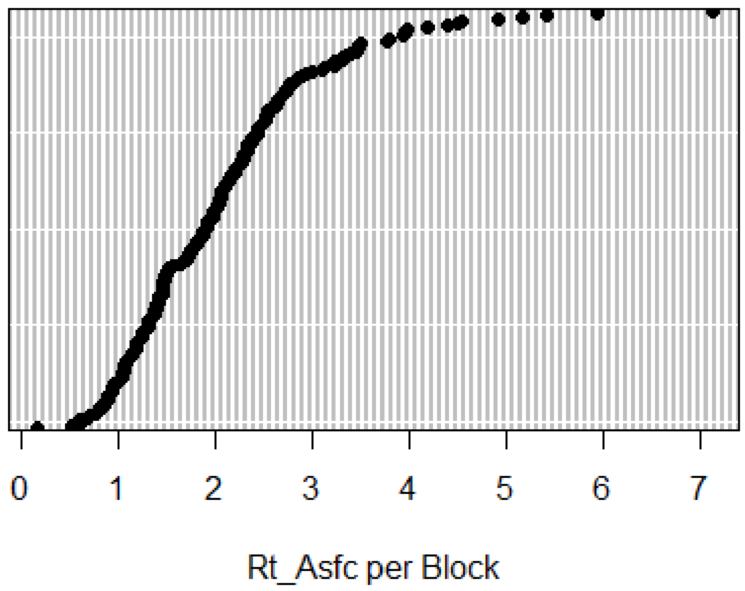


```
x <- Rt[order(Rt_epLsar), ]
x$Blocs <- factor(x$Blocs)
dotchart(x$epLsar, cex = 1, pch = 16, groups = x$Blocks, xlab = "Rt_epLsar pe
r Block")
```

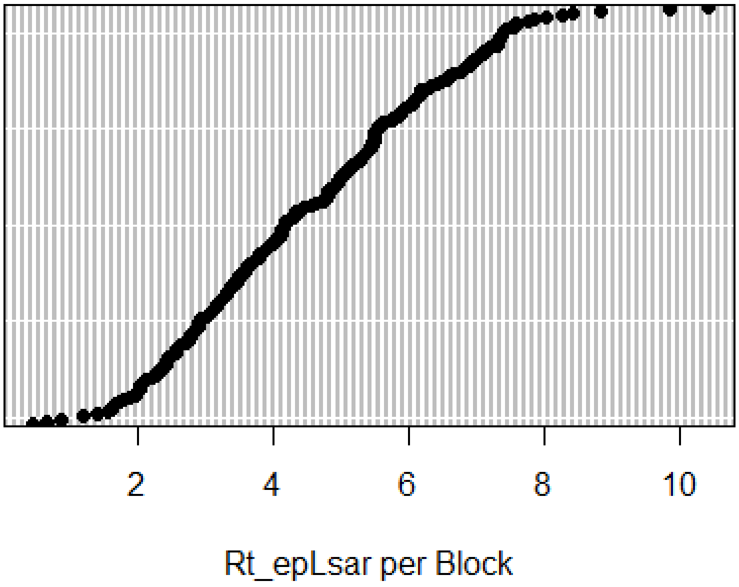


```
x <- Rt[order(Rt_Smc), ]
x$Blocs <- factor(x$Blocs)
dotchart(x$Smc, cex = 1, pch = 16, groups = x$Blocks, xlab = "Rt_Smc per Bloc
k")
```

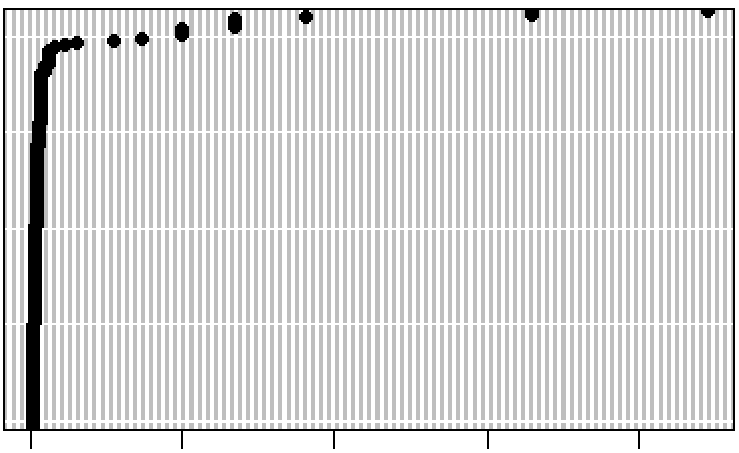


```
x <- Rt[order(Rt_H9), ]
x$Blocs <- factor(x$Blocs)
dotchart(x$HAsfc9, cex = 1, pch = 16, groups = x$Blocks, xlab = "Rt_HAsfc9 pe
r Block")
```

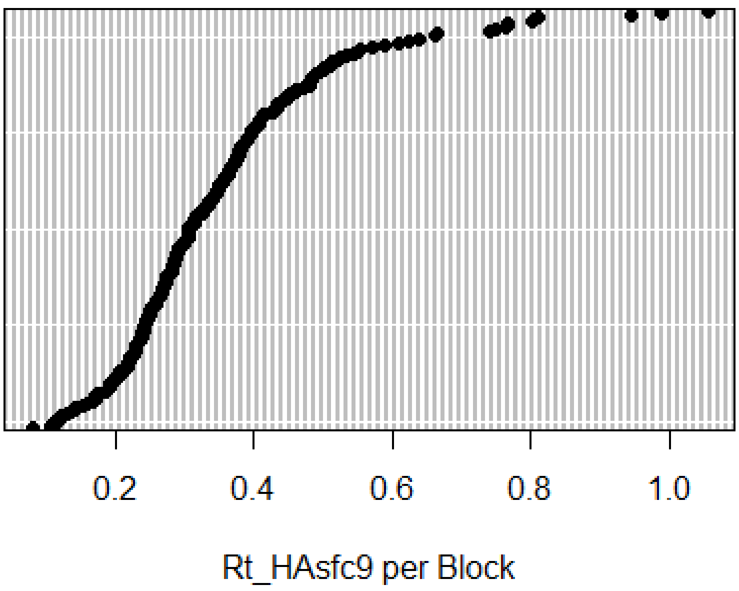


```
x <- Rt[order(Rt_H36), ]
x$Blocs <- factor(x$Blocs)
dotchart(x$HAsfc36, cex = 1, pch = 16, groups = x$Blocks, xlab = "Rt_HAsfc36
per Block")
```

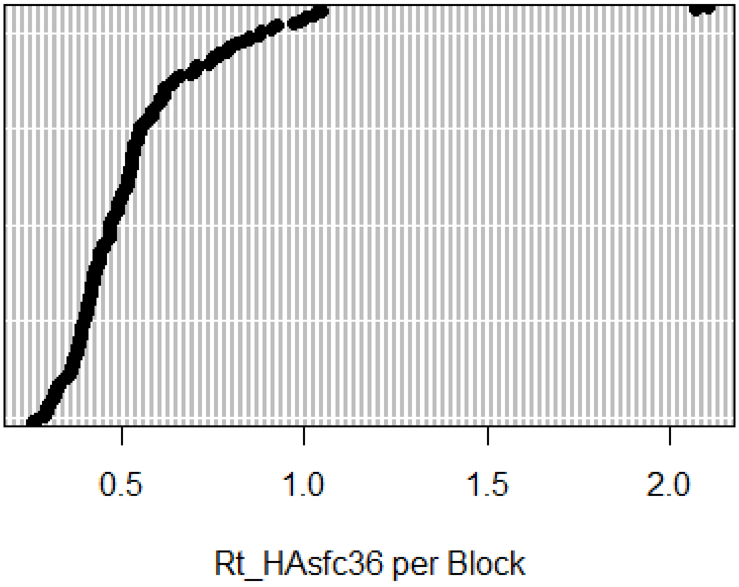


```
x <- Rt[order(Rt_H81), ]
x$Blocs <- factor(x$Blocs)
dotchart(x$HAsfc81, cex = 1, pch = 16, groups = x$Blocks, xlab = "Rt_HAsfc81
per Block")
```

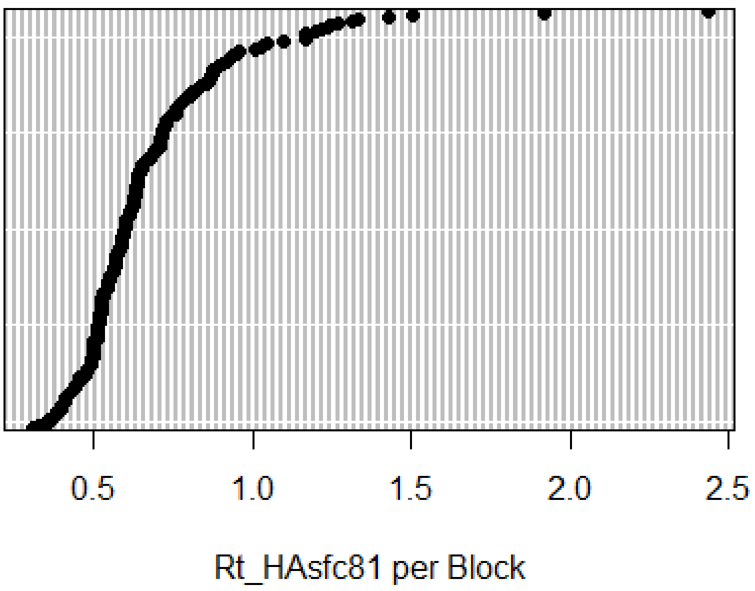

Graphical evaluation of the tests' applicability:

Normality and homoscedasticity of the variables. ##### Normality

```
ggplot(Rt) +
  geom_freqpoly(aes(x = Asfc), bins = 7) +
  labs(
    x = "Value Asfc",
    y = "Frequency"
  )
```

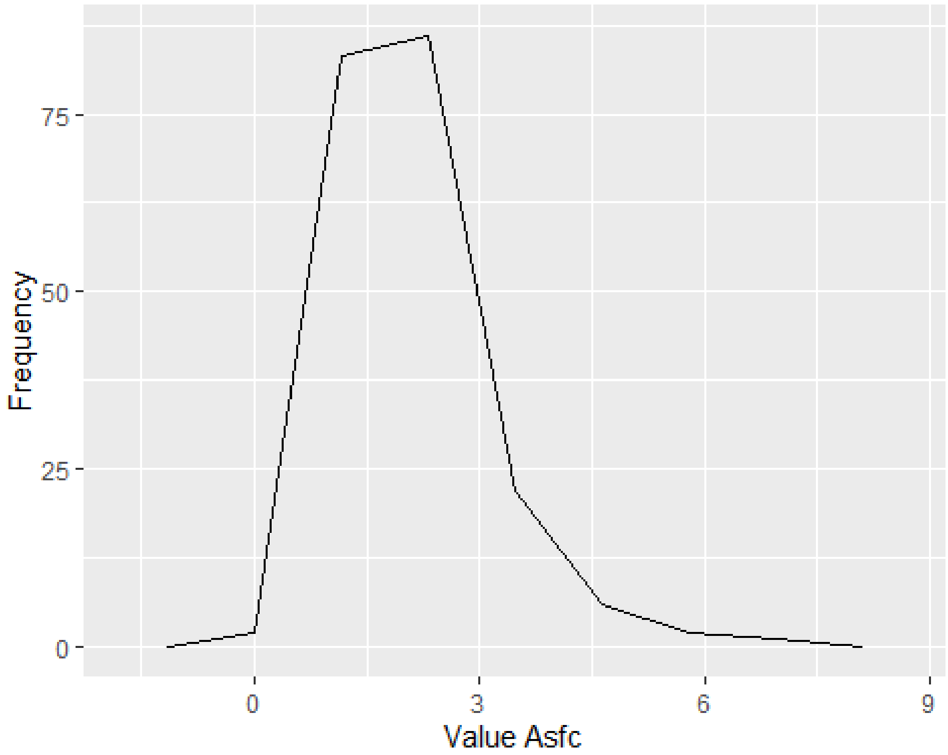


```
ggplot(Rt) +
  geom_freqpoly(aes(x = epLsar), bins = 7) +
  labs(
    x = "Value epLsar",
    y = "Frequency"
  )
```

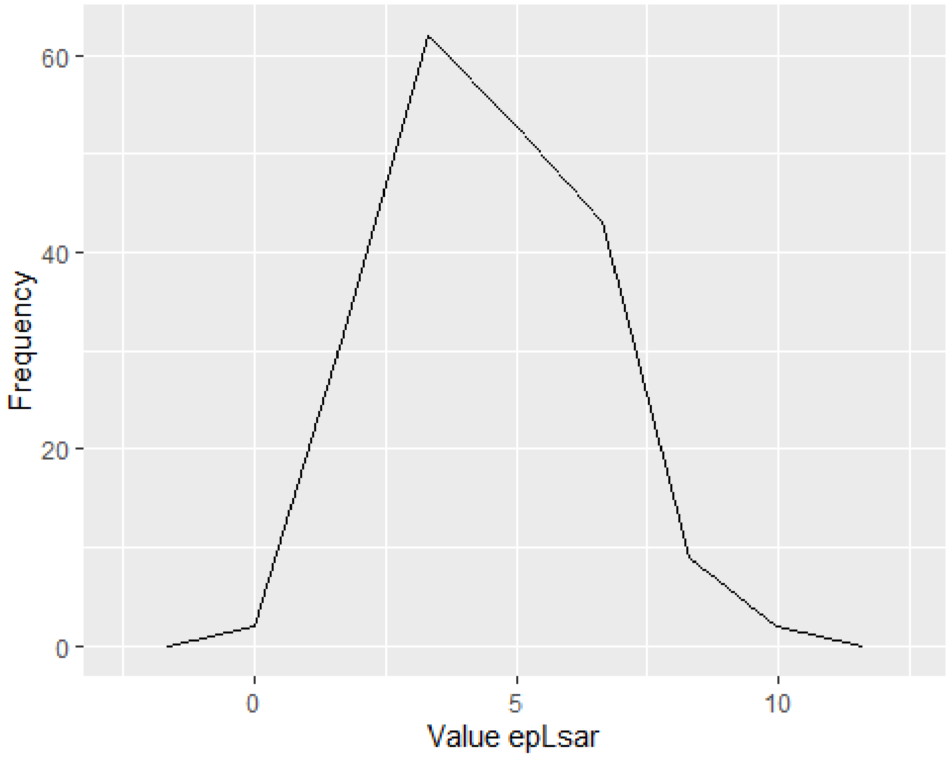


```
ggplot(Rt) +
  geom_freqpoly(aes(x = Smc), bins = 7) +
  labs(
    x = "Value Smc",
    y = "Frequency"
  )
```

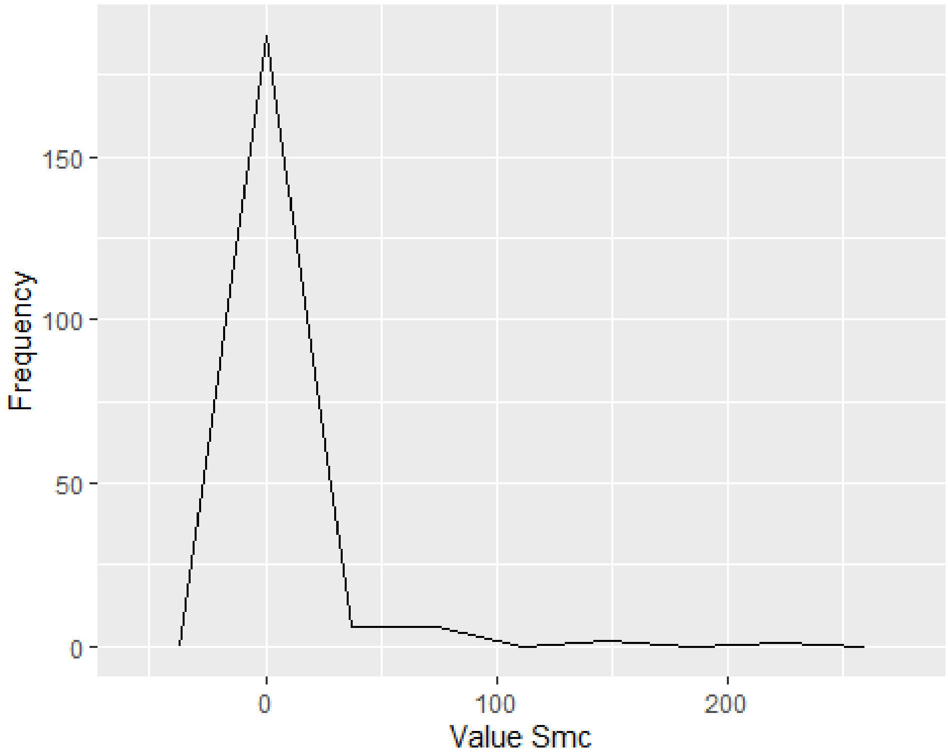


```
ggplot(Rt) +
  geom_freqpoly(aes(x = HAsfc9), bins = 7) +
  labs(
    x = "Value H9",
    y = "Frequency"
  )
```

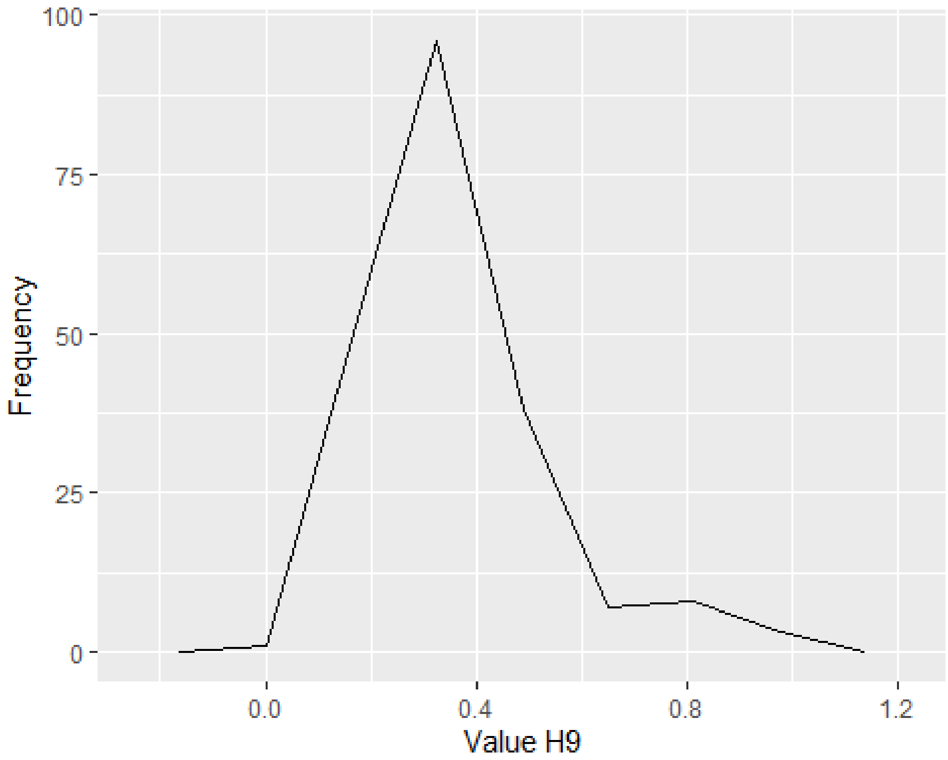


```
ggplot(Rt) +
  geom_freqpoly(aes(x = HAsfc36), bins = 7) +
  labs(
    x = "Value H36",
    y = "Frequency"
  )
```

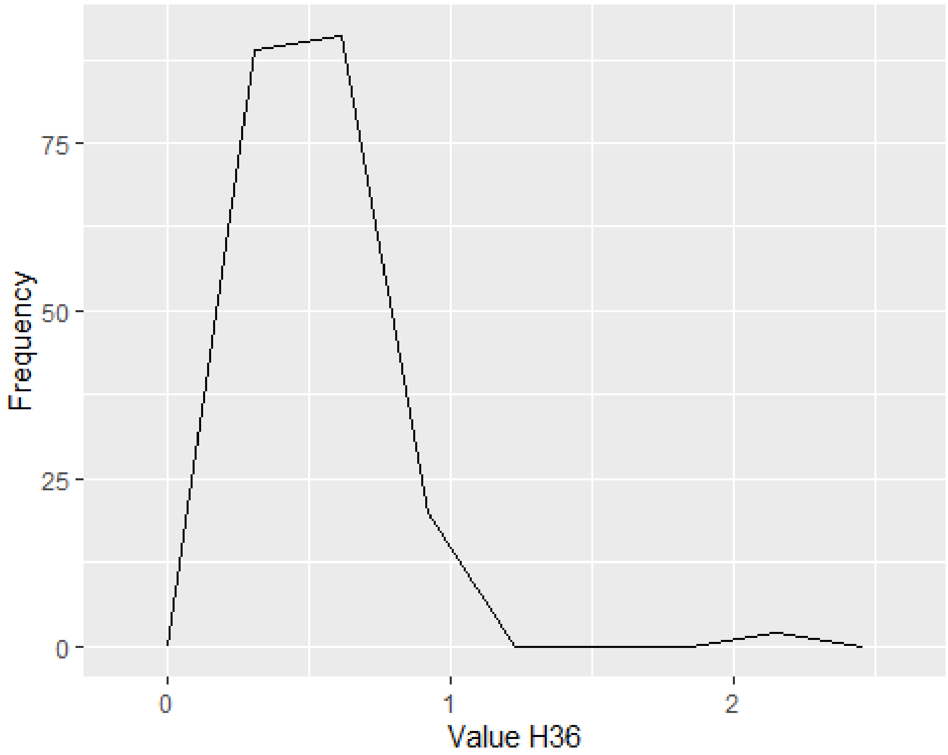


```
ggplot(Rt) +
  geom_freqpoly(aes(x = HAsfc81), bins = 7) +
  labs(
    x = "Value H81",
    y = "Frequency"
  )
```

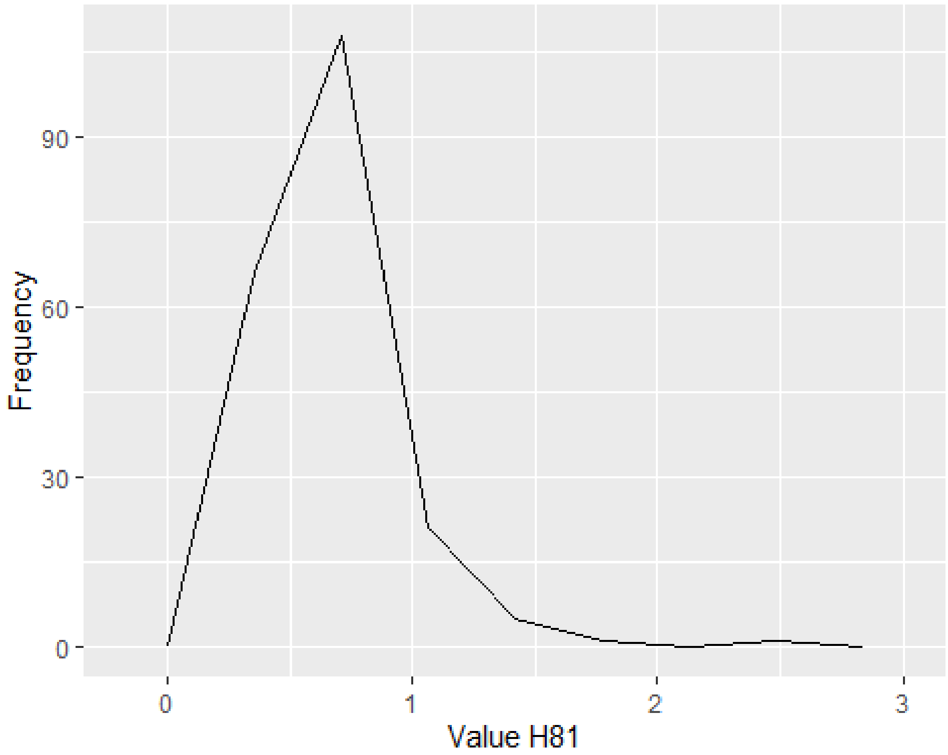

Homoscedasticity: Brown & Forsythe test (and data transformation whenever needed)

```
bf.test(Rt_Asfc ~ Rt_Blocs, data = Rt)

##
##   Brown-Forsythe Test (alpha = 0.05)
## -------------------------------------------------------------
##   data : Rt_Asfc and Rt_Blocs
##
##   statistic  : 1.435567
##   num df     : 7
##   denom df   : 45.19699
##   p.value    : 0.214935
##
##   Result     : Difference is not statistically significant.
## -------------------------------------------------------------

ggplot(Rt) +
  geom_boxplot(aes(x = Rt_Blocs, y = Rt_Asfc)) +
  labs(
    x = "Blocks",
    y = "Asfc"
  )
```

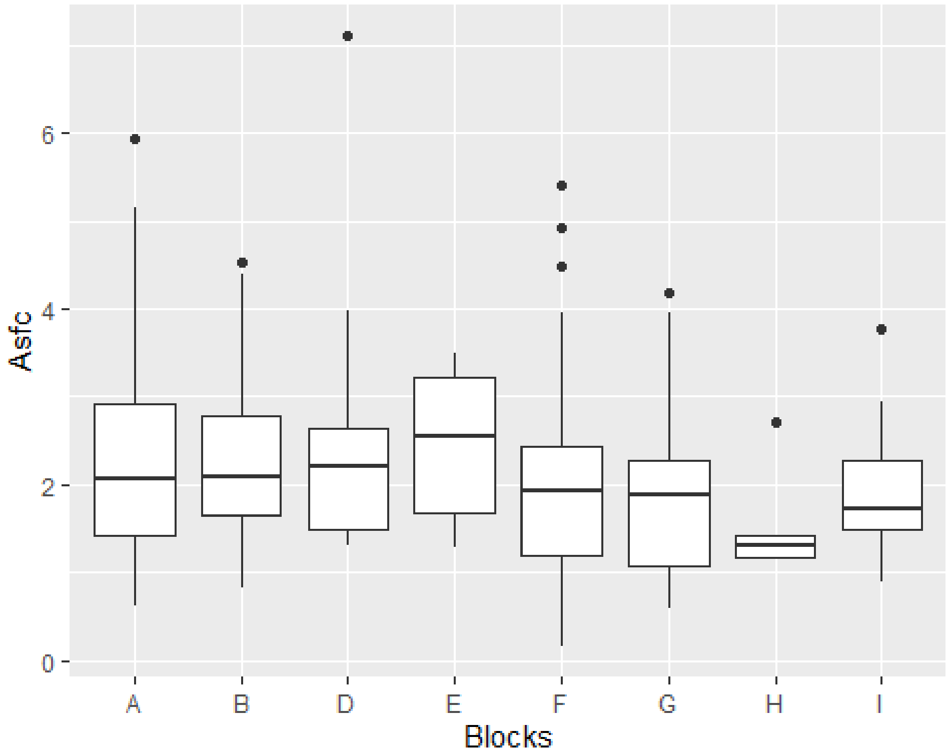


```
bf.test(Rt_epLsar ~ Rt_Blocs, data = Rt) # /!\ The population variances are n
ot equal: a data transformation is needed

##
##   Brown-Forsythe Test (alpha = 0.05)
## -------------------------------------------------------------
##   data : Rt_epLsar and Rt_Blocs
##
##   statistic  : 2.984574
##   num df     : 7
##   denom df   : 82.70421
##   p.value    : 0.007656274
##
##   Result     : Difference is statistically significant.
## -------------------------------------------------------------

logged <- log(Rt_epLsar + 1)
bf.test(logged ~ Rt_Blocs, data = Rt) # the variances remain significantly di
fferent

##
##   Brown-Forsythe Test (alpha = 0.05)
## -------------------------------------------------------------
##   data : logged and Rt_Blocs
##
##   statistic  : 2.997684
##   num df     : 7
```

```
##    denom df    : 85.45104
##    p.value     : 0.007302256
##
##    Result      : Difference is statistically significant.
## -------------------------------------------------------------
```

```
sqrted <- sqrt(Rt_epLsar)
bf.test(sqrted ~ Rt_Blocs, data = Rt) # the variances remain significantly di
fferent
```

```
##
##    Brown-Forsythe Test (alpha = 0.05)
## -------------------------------------------------------------
##    data : sqrted and Rt_Blocs
##
##    statistic   : 3.04216
##    num df      : 7
##    denom df    : 88.07428
##    p.value     : 0.00650251
##
##    Result      : Difference is statistically significant.
## -------------------------------------------------------------
```

```
GLM_Rt_epLsar <- glm(formula = Rt_epLsar ~ Rt_Blocs, family = gaussian)
bc_Rt_epLsar <- boxcox(GLM_Rt_epLsar, lambda = seq(-3, 3))
```

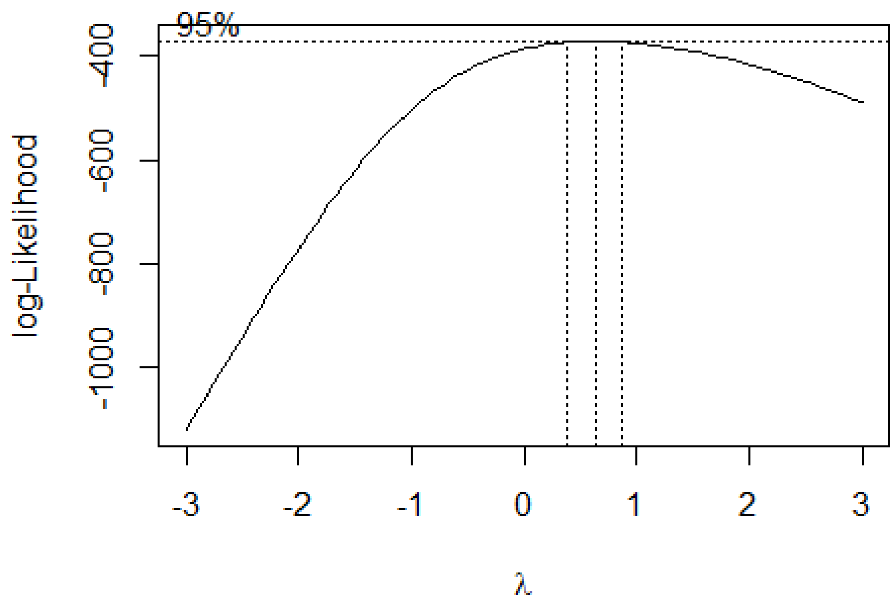


```
best.lam <- bc_Rt_epLsar$x[which(bc_Rt_epLsar$y == max(bc_Rt_epLsar$y))]
best.lam # 0.6363636
```

```
## [1] 0.6363636

new_Rt_epLsar <- (Rt_epLsar)^0.6363636
bf.test(new_Rt_epLsar ~ Rt_Blocs, data = Rt) # the variances remain significa
ntly different

##
##   Brown-Forsythe Test (alpha = 0.05)
## -------------------------------------------------------------
##   data : new_Rt_epLsar and Rt_Blocs
##
##   statistic  : 3.042074
##   num df     : 7
##   denom df   : 87.79867
##   p.value    : 0.006515284
##
##   Result     : Difference is statistically significant.
## -------------------------------------------------------------

ggplot(Rt) +
  geom_boxplot(aes(x = Rt_Blocs, y = new_Rt_epLsar)) +
  labs(
    x = "Blocks",
    y = "epLsar"
  )
```

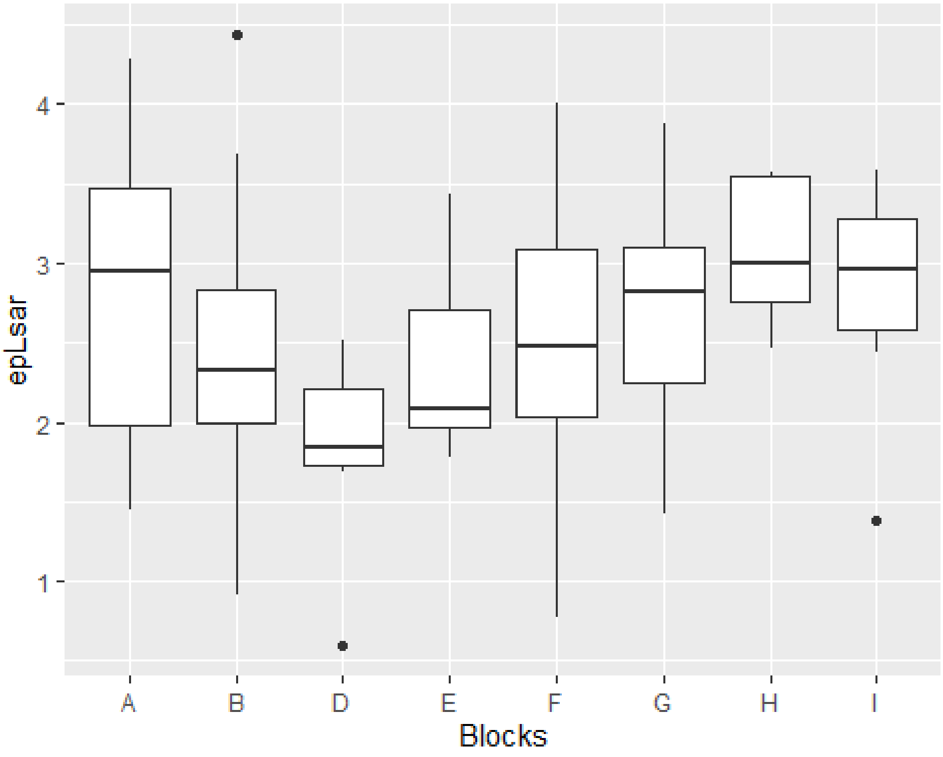

```r
ranked <- rank(Rt_epLsar)
bf.test(ranked ~ Rt_Blocs, data = Rt) # the variances remain significantly di
fferent

## 
##   Brown-Forsythe Test (alpha = 0.05) 
## ------------------------------------------------------------- 
##   data : ranked and Rt_Blocs 
## 
##   statistic  : 3.163601 
##   num df     : 7 
##   denom df   : 84.59382 
##   p.value    : 0.005083279 
## 
##   Result     : Difference is statistically significant. 
## -------------------------------------------------------------

bf.test(Rt_Smc ~ Rt_Blocs, data = Rt) # /!\ The population variances are not
equal.

## 
##   Brown-Forsythe Test (alpha = 0.05) 
## ------------------------------------------------------------- 
##   data : Rt_Smc and Rt_Blocs 
## 
##   statistic  : 3.158163 
##   num df     : 7 
##   denom df   : 35.6394 
##   p.value    : 0.01054902 
## 
##   Result     : Difference is statistically significant. 
## -------------------------------------------------------------

GLM_Rt_Smc <- glm(formula = Rt_Smc ~ Rt_Blocs, family = gaussian)
bc_Rt_Smc <- boxcox(GLM_Rt_Smc, lambda = seq(-3, 3))
```

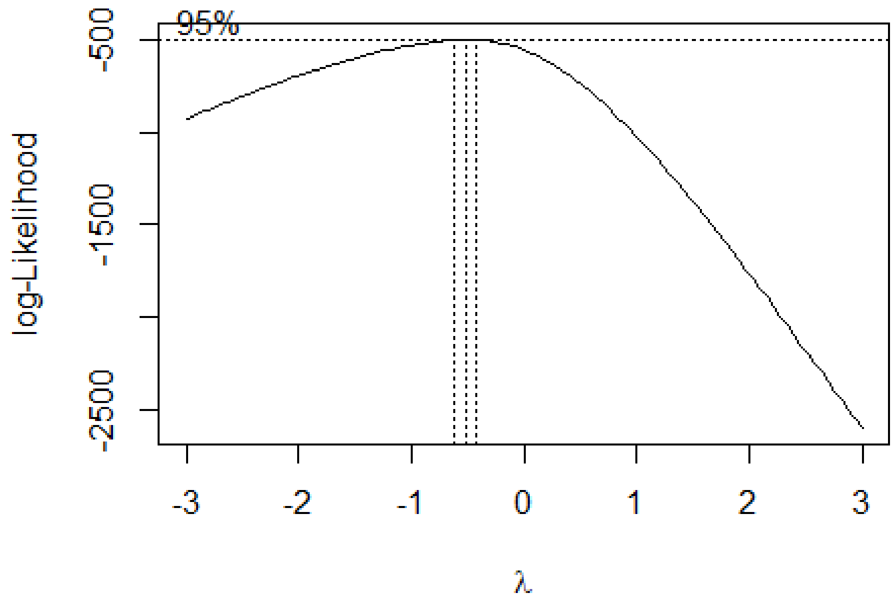


```
best.lam <- bc_Rt_Smc$x[which(bc_Rt_Smc$y == max(bc_Rt_Smc$y))]
best.lam # -0.5151515

## [1] -0.5151515

Rt_Smc <- (Rt_Smc)^-0.5151515
bf.test(Rt_Smc ~ Rt_Blocs, data = Rt) # ok

##
##   Brown-Forsythe Test (alpha = 0.05)
## -------------------------------------------------------------
##   data : Rt_Smc and Rt_Blocs
##
##   statistic  : 1.610571
##   num df     : 7
##   denom df   : 69.90502
##   p.value    : 0.1467835
##
##   Result     : Difference is not statistically significant.
## -------------------------------------------------------------

ggplot(Rt) + geom_boxplot(aes(x = Rt_Blocs, y = Rt_Smc)) +labs(  x = "Blocks"
,  y = "Smc"  )
```

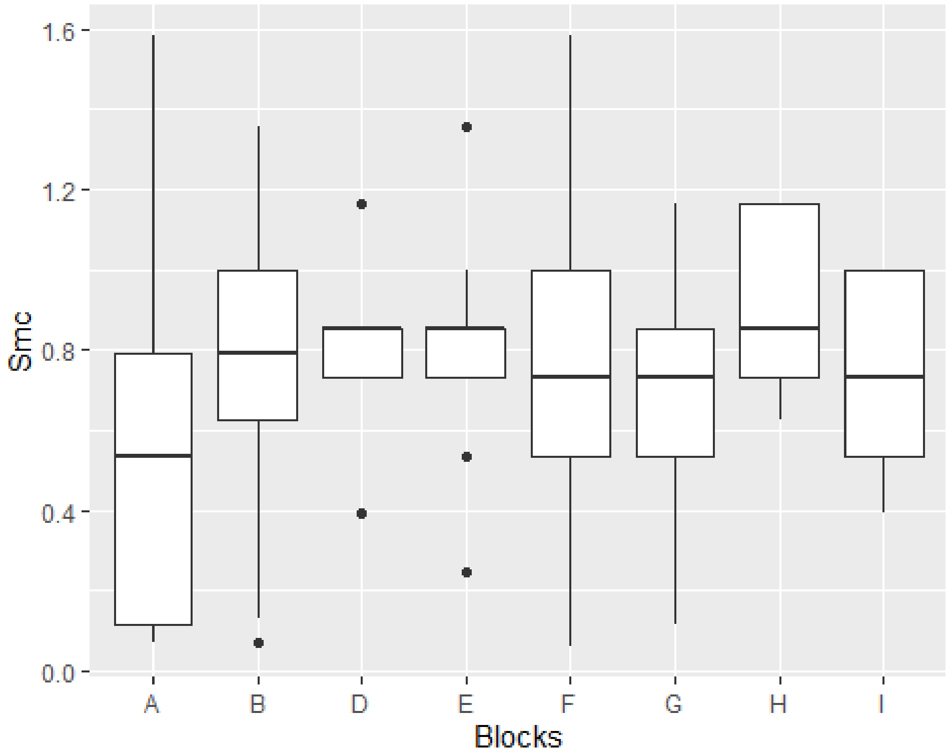


```
bf.test(Rt_H9 ~ Rt_Blocs, data = Rt)

##
##   Brown-Forsythe Test (alpha = 0.05)
## -------------------------------------------------------------
##   data : Rt_H9 and Rt_Blocs
##
##   statistic  : 1.936102
##   num df     : 7
##   denom df   : 76.14769
##   p.value    : 0.07526283
##
##   Result     : Difference is not statistically significant.
## -------------------------------------------------------------

ggplot(Rt) +
  geom_boxplot(aes(x = Rt_Blocs, y = Rt_H9)) +
  labs(
    x = "Blocks",
    y = "H9"
  )
```

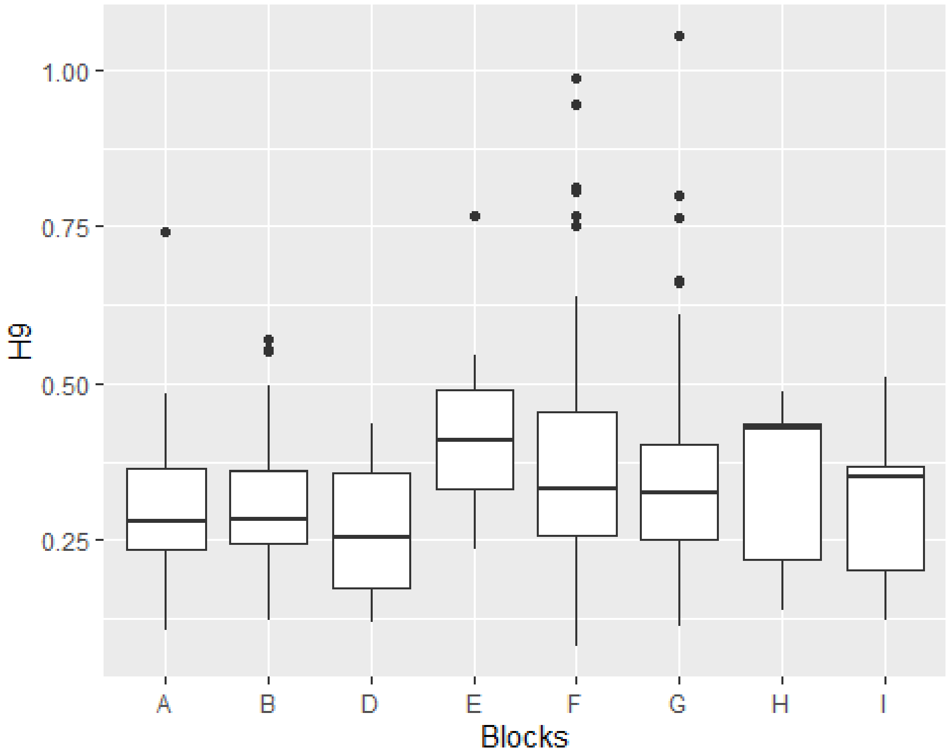


```
bf.test(Rt_H36 ~ Rt_Blocs, data = Rt)

##
##   Brown-Forsythe Test (alpha = 0.05)
## -------------------------------------------------------------
##   data : Rt_H36 and Rt_Blocs
##
##   statistic  : 0.7956053
##   num df     : 7
##   denom df   : 61.87303
##   p.value    : 0.5938707
##
##   Result     : Difference is not statistically significant.
## -------------------------------------------------------------

ggplot(Rt) +
  geom_boxplot(aes(x = Rt_Blocs, y = Rt_H36)) +
  labs(
    x = "Blocks",
    y = "H36"
  )
```

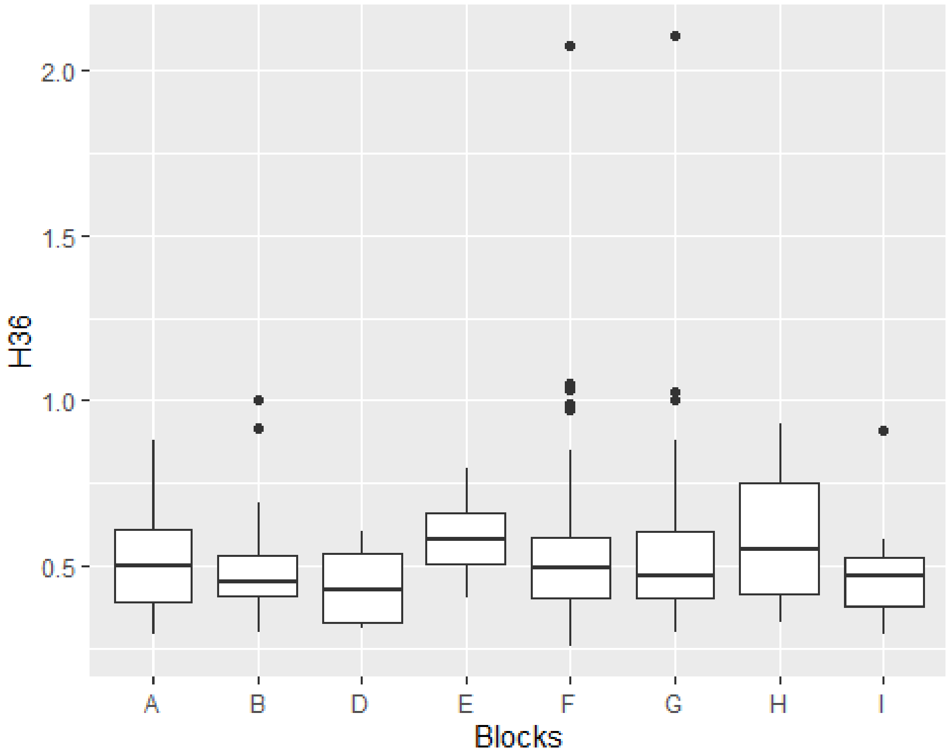


```
bf.test(Rt_H81 ~ Rt_Blocs, data = Rt)

##
##   Brown-Forsythe Test (alpha = 0.05)
## -------------------------------------------------------------
##   data : Rt_H81 and Rt_Blocs
##
##   statistic  : 0.7326512
##   num df     : 7
##   denom df   : 55.43768
##   p.value    : 0.6451119
##
##   Result     : Difference is not statistically significant.
## -------------------------------------------------------------

ggplot(Rt) +
  geom_boxplot(aes(x = Rt_Blocs, y = Rt_H81)) +
  labs(
    x = "Blocks",
    y = "H81"
  )
```

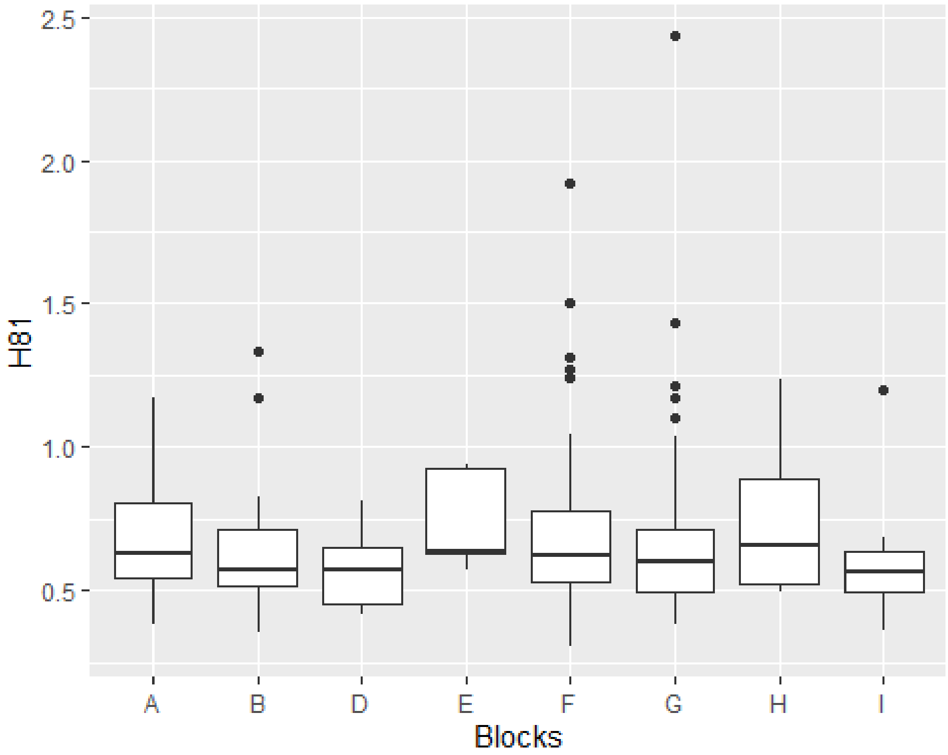


```
bf.test(Rt_Asfc ~ Rt_Period, data = Rt)

##
##   Brown-Forsythe Test (alpha = 0.05)
## -------------------------------------------------------------
##   data : Rt_Asfc and Rt_Period
##
##   statistic  : 2.891923
##   num df     : 2
##   denom df   : 167.2665
##   p.value    : 0.05824791
##
##   Result     : Difference is not statistically significant.
## -------------------------------------------------------------

ggplot(Rt) +
  geom_boxplot(aes(x = Rt_Period, y = Rt_Asfc)) +
  labs(
    x = "Period",
    y = "Asfc"
  )
```

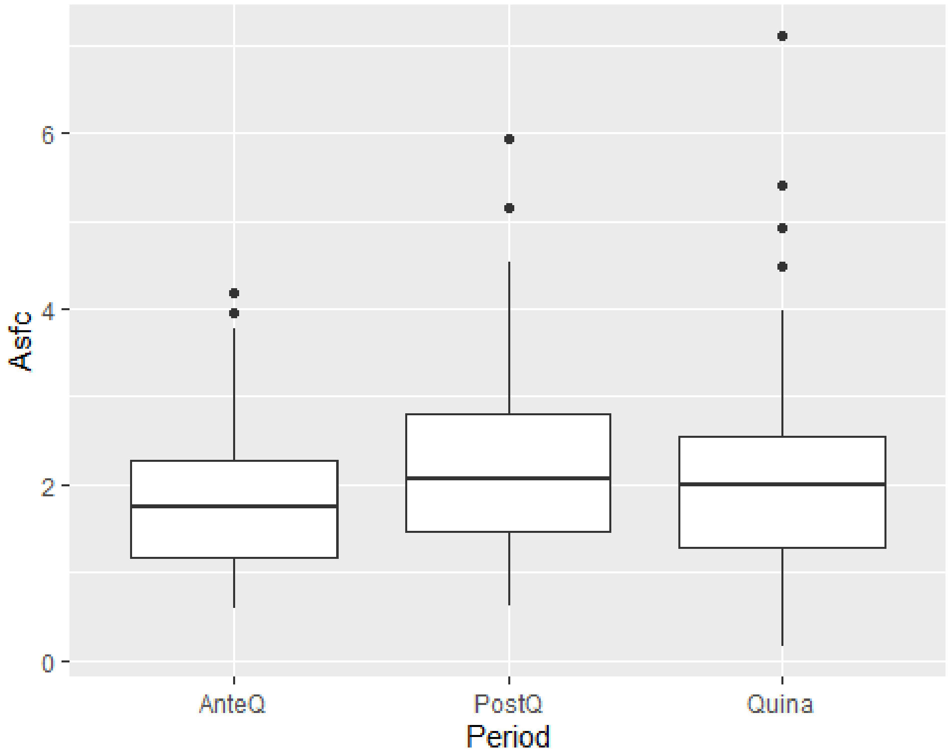


```
bf.test(Rt_epLsar ~ Rt_Period, data = Rt)

##
##   Brown-Forsythe Test (alpha = 0.05)
## -------------------------------------------------------------
##   data : Rt_epLsar and Rt_Period
##
##   statistic  : 3.157404
##   num df     : 2
##   denom df   : 150.7518
##   p.value    : 0.0453627
##
##   Result     : Difference is statistically significant.
## -------------------------------------------------------------

GLM_Rt_epLsar <- glm(formula = Rt_epLsar ~ Rt_Period, family = gaussian)
bc_Rt_epLsar <- boxcox(GLM_Rt_epLsar, lambda = seq(-3, 3))
```

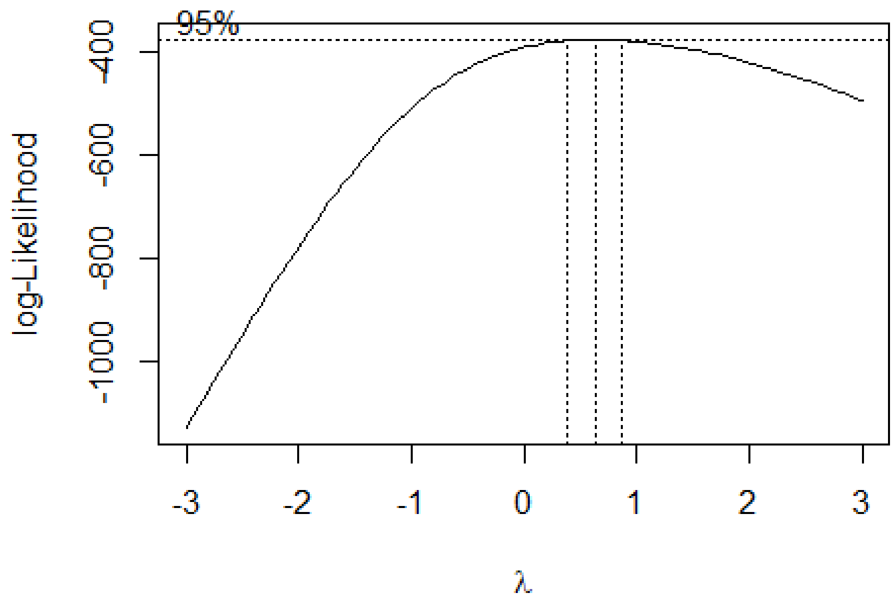


```
best.lam <- bc_Rt_epLsar$x[which(bc_Rt_epLsar$y == max(bc_Rt_epLsar$y))]
best.lam # 0.6363636

## [1] 0.6363636

Rt_epLsar <- (Rt_epLsar)^0.6363636
bf.test(Rt_epLsar ~ Rt_Period, data = Rt)

##
##   Brown-Forsythe Test (alpha = 0.05)
## -------------------------------------------------------------
##   data : Rt_epLsar and Rt_Period
##
##   statistic  : 3.3783
##   num df     : 2
##   denom df   : 155.0961
##   p.value    : 0.03663381
##
##   Result     : Difference is statistically significant.
## -------------------------------------------------------------

ggplot(Rt) +
  geom_boxplot(aes(x = Rt_Period, y = Rt_epLsar)) +
  labs(
    x = "Period",
    y = "epLsar"
  )
```

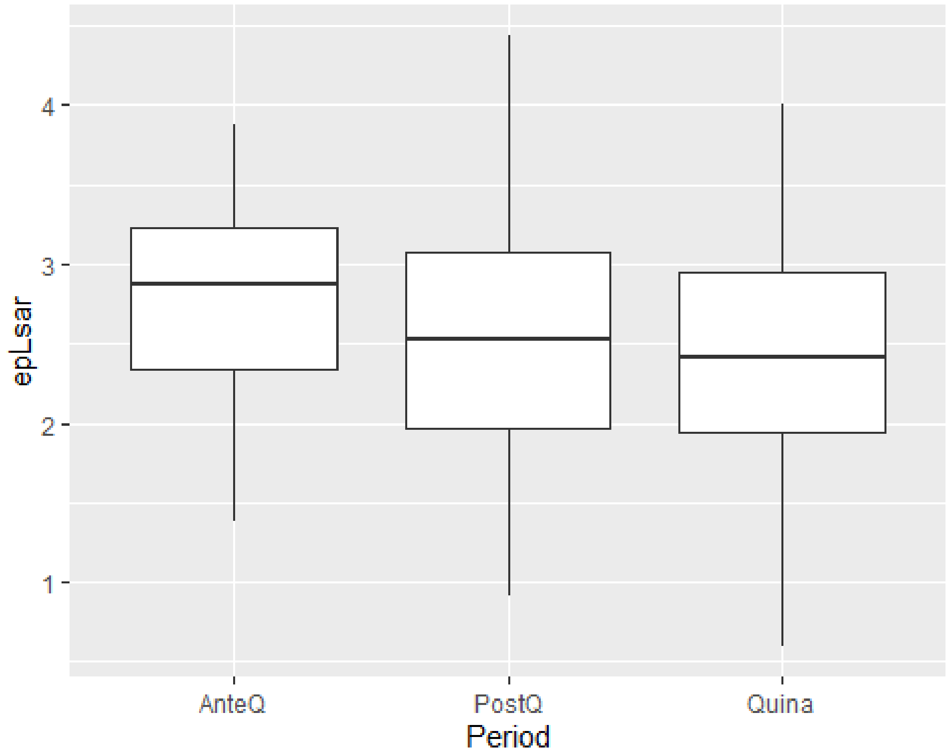


```
bf.test(Rt_Smc ~ Rt_Period, data = Rt)

##
##   Brown-Forsythe Test (alpha = 0.05)
## -------------------------------------------------------------
##   data : Rt_Smc and Rt_Period
##
##   statistic  : 1.717734
##   num df     : 2
##   denom df   : 143.187
##   p.value    : 0.1831497
##
##   Result     : Difference is not statistically significant.
## -------------------------------------------------------------

ggplot(Rt) +
  geom_boxplot(aes(x = Rt_Period, y = Rt_Smc)) +
  labs(
    x = "Period",
    y = "Smc"
  )
```

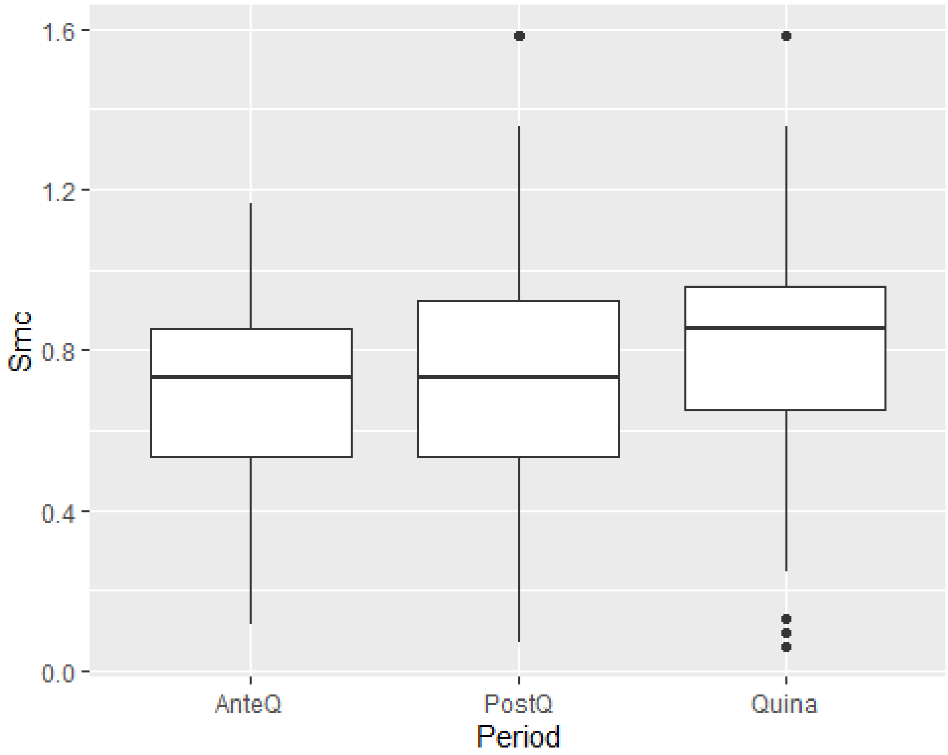


```
bf.test(Rt_H9 ~ Rt_Period, data = Rt)

##
##   Brown-Forsythe Test (alpha = 0.05)
## -------------------------------------------------------------
##   data : Rt_H9 and Rt_Period
##
##   statistic  : 2.499414
##   num df     : 2
##   denom df   : 174.1969
##   p.value    : 0.08507486
##
##   Result     : Difference is not statistically significant.
## -------------------------------------------------------------

ggplot(Rt) +
  geom_boxplot(aes(x = Rt_Period, y = Rt_H9)) +
  labs(
    x = "Period",
    y = "H9"
  )
```

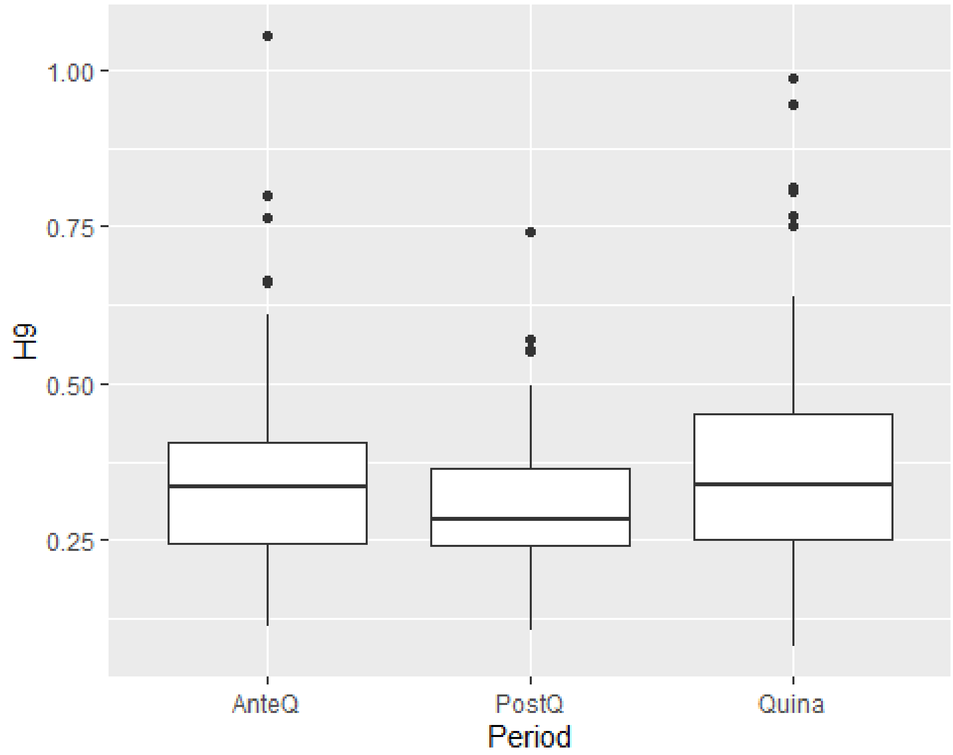


```
bf.test(Rt_H36 ~ Rt_Period, data = Rt)

##
##   Brown-Forsythe Test (alpha = 0.05)
## -------------------------------------------------------------
##   data : Rt_H36 and Rt_Period
##
##   statistic  : 0.7563553
##   num df     : 2
##   denom df   : 149.8799
##   p.value    : 0.471157
##
##   Result     : Difference is not statistically significant.
## -------------------------------------------------------------

ggplot(Rt) +
  geom_boxplot(aes(x = Rt_Period, y = Rt_H36)) +
  labs(
    x = "Period",
    y = "H36"
  )
```

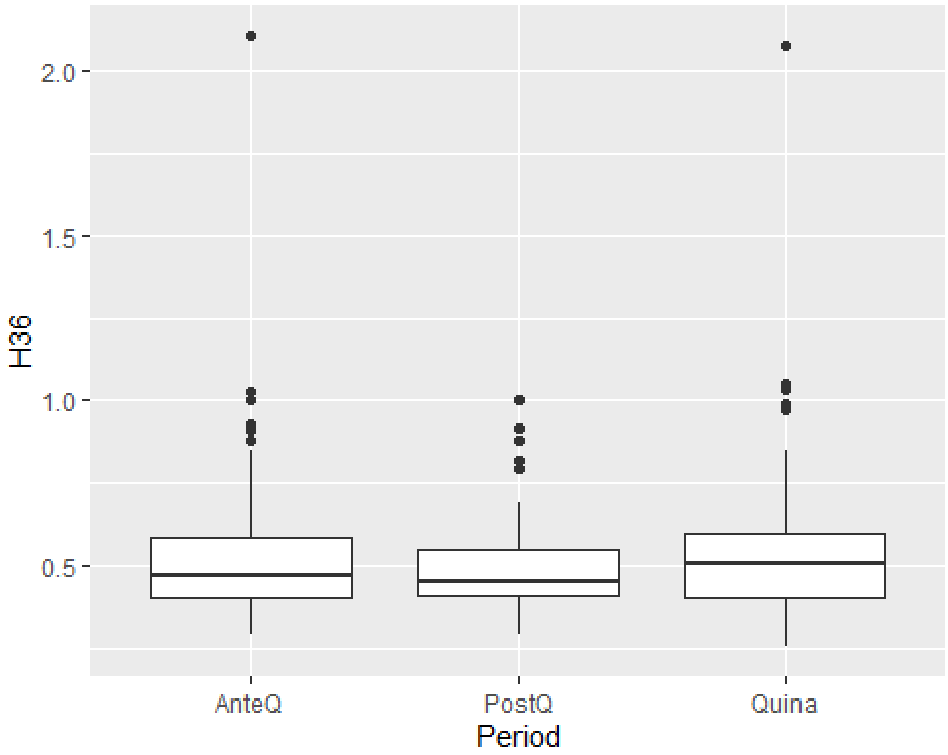


```
bf.test(Rt_H81 ~ Rt_Period, data = Rt)

##
##   Brown-Forsythe Test (alpha = 0.05)
## -------------------------------------------------------------
##   data : Rt_H81 and Rt_Period
##
##   statistic  : 0.4894905
##   num df     : 2
##   denom df   : 141.502
##   p.value    : 0.6139726
##
##   Result     : Difference is not statistically significant.
## -------------------------------------------------------------

ggplot(Rt) +
  geom_boxplot(aes(x = Rt_Period, y = Rt_H81)) +
  labs(
    x = "Period",
    y = "H81"
  )
```

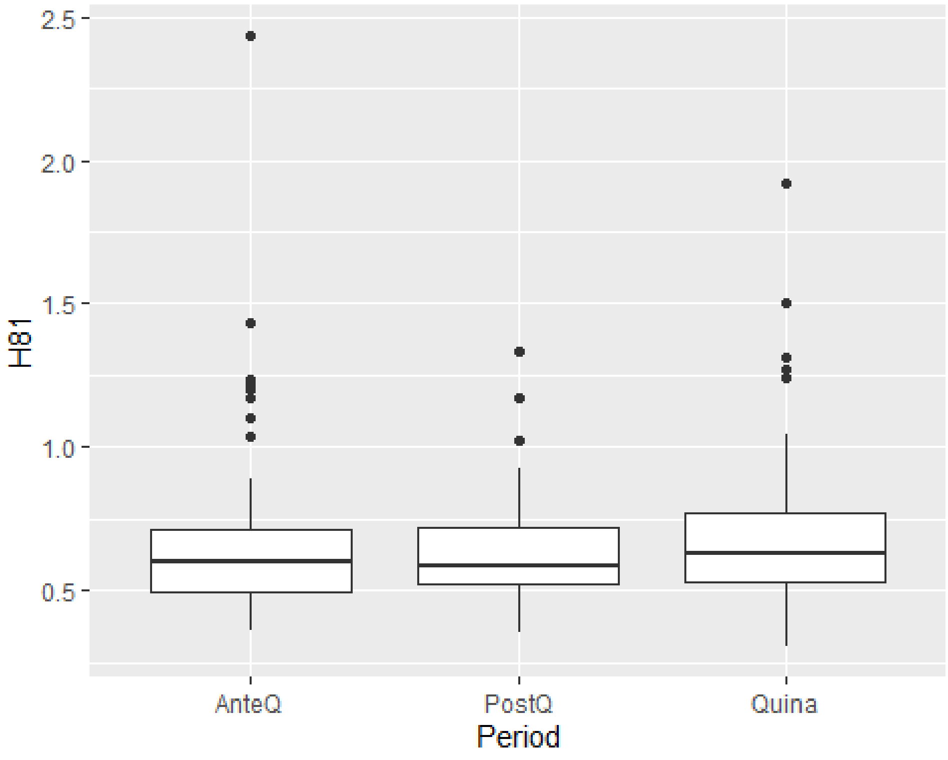

GLM analyses

What is the impact of random (1), blocks (Rt_Blocks) and period (Rt_Period) over each DMTA parameter?

```
glm_Rt_Asfc0 <- glm(Rt_Asfc ~ 1, data = Rt)
glm_Rt_Asfc1 <- glm(Rt_Asfc ~ Rt_Blocs, data = Rt)
glm_Rt_Asfc2 <- glm(Rt_Asfc ~ Rt_Period, data = Rt)
glm_Rt_Asfc3 <- glm(Rt_Asfc ~ Rt_layer, data = Rt)

Cand.models <- list()
Cand.models[[1]] <- glm_Rt_Asfc0
Cand.models[[2]] <- glm_Rt_Asfc1
Cand.models[[3]] <- glm_Rt_Asfc2
Cand.models[[4]] <- glm_Rt_Asfc3
Modnames <- lapply(Cand.models, "formula")
aictab(cand.set = Cand.models, modnames = paste0(Modnames), sort = TRUE)

##
## Model selection based on AICc:
##
##                         K   AICc Delta_AICc AICcWt Cum.Wt      LL
## Rt_Asfc ~ Rt_Period  4 593.64       0.00   0.61   0.61 -292.72
## Rt_Asfc ~ 1          2 595.00       1.36   0.31   0.91 -295.47
## Rt_Asfc ~ Rt_Blocs   9 597.66       4.02   0.08   1.00 -289.36
## Rt_Asfc ~ Rt_layer  30 603.51       9.87   0.00   1.00 -266.32
```

```r
marginal <- emmeans(glm_Rt_Asfc2, ~Rt_Period)
pairs(marginal)

##  contrast      estimate    SE  df t.ratio p.value
##  AnteQ - PostQ   -0.467 0.200 199  -2.334  0.0534
##  AnteQ - Quina   -0.253 0.174 199  -1.453  0.3159
##  PostQ - Quina    0.214 0.181 199   1.185  0.4637
##
## P value adjustment: tukey method for comparing a family of 3 estimates

# For epLsar, We perform non-parametric tests to identify differences
kruskal.test(Rt_epLsar ~ Rt_Blocs, data = Rt)

##
##  Kruskal-Wallis rank sum test
##
## data:  Rt_epLsar by Rt_Blocs
## Kruskal-Wallis chi-squared = 18.613, df = 7, p-value = 0.009491

dunnTest(Rt_epLsar, Rt_Blocs, method = "bonferroni")

##    Comparison          Z      P.unadj      P.adj
## 1       A - B  1.6738033 0.094169255 1.00000000
## 2       A - D  2.9121820 0.003589135 0.10049578
## 3       B - D  1.9145042 0.055555765 1.00000000
## 4       A - E  1.7107942 0.087119110 1.00000000
## 5       B - E  0.5552891 0.578696939 1.00000000
## 6       D - E -1.0745539 0.282574510 1.00000000
## 7       A - F  1.2781446 0.201198456 1.00000000
## 8       B - F -0.7575553 0.448717229 1.00000000
## 9       D - F -2.4587858 0.013940778 0.39034180
## 10      E - F -1.0218395 0.306856875 1.00000000
## 11      A - G  0.4103849 0.681523629 1.00000000
## 12      B - G -1.7322578 0.083227665 1.00000000
## 13      D - G -3.0140180 0.002578125 0.07218749
## 14      E - G -1.6321208 0.102654042 1.00000000
## 15      F - G -1.2475114 0.212210026 1.00000000
## 16      A - H -0.8635120 0.387856035 1.00000000
## 17      B - H -2.0121200 0.044207286 1.00000000
## 18      D - H -3.0008594 0.002692189 0.07538128
## 19      E - H -2.0926956 0.036376336 1.00000000
## 20      F - H -1.7479964 0.080464642 1.00000000
## 21      G - H -1.2041876 0.228517062 1.00000000
## 22      A - I -0.3921979 0.694912010 1.00000000
## 23      B - I -1.8239748 0.068155875 1.00000000
## 24      D - I -2.9555272 0.003121352 0.08739785
## 25      E - I -1.8809733 0.059975551 1.00000000
## 26      F - I -1.4934905 0.135308849 1.00000000
## 27      G - I -0.7868476 0.431371114 1.00000000
## 28      H - I  0.5029830 0.614976212 1.00000000
```

```
kruskal.test(Rt_epLsar ~ Rt_Period, data = Rt)

##
##  Kruskal-Wallis rank sum test
##
## data:  Rt_epLsar by Rt_Period
## Kruskal-Wallis chi-squared = 7.3311, df = 2, p-value = 0.02559

dunnTest(Rt_epLsar, Rt_Period, method = "bonferroni")

##      Comparison         Z     P.unadj      P.adj
## 1 AnteQ - PostQ 1.7405548 0.081761653 0.24528496
## 2 AnteQ - Quina 2.6864178 0.007222272 0.02166682
## 3 PostQ - Quina 0.6640946 0.506629757 1.00000000

glm_Rt_Smc0 <- glm(Rt_Smc ~ 1, data = Rt)
glm_Rt_Smc1 <- glm(Rt_Smc ~ Rt_Blocs, data = Rt)
glm_Rt_Smc2 <- glm(Rt_Smc ~ Rt_Period, data = Rt)
glm_Rt_Smc3 <- glm(Rt_Smc ~ Rt_layer, data = Rt)
Cand.models <- list()
Cand.models[[1]] <- glm_Rt_Smc0
Cand.models[[2]] <- glm_Rt_Smc1
Cand.models[[3]] <- glm_Rt_Smc2
Cand.models[[4]] <- glm_Rt_Smc3
Modnames <- lapply(Cand.models, "formula")
aictab(cand.set = Cand.models, modnames = paste0(Modnames), sort = TRUE)

##
## Model selection based on AICc:
##
##                       K   AICc Delta_AICc AICcWt Cum.Wt     LL
## Rt_Smc ~ 1            2  89.69       0.00   0.52   0.52 -42.81
## Rt_Smc ~ Rt_Period    4  90.22       0.53   0.40   0.92 -41.01
## Rt_Smc ~ Rt_Blocs     9  93.39       3.70   0.08   1.00 -37.23
## Rt_Smc ~ Rt_layer    30 117.98      28.29   0.00   1.00 -23.55

glm_Rt_H9_0 <- glm(Rt_H9 ~ 1, data = Rt)
glm_Rt_H9_1 <- glm(Rt_H9 ~ Rt_Blocs, data = Rt)
glm_Rt_H9_2 <- glm(Rt_H9 ~ Rt_Period, data = Rt)
glm_Rt_H9_3 <- glm(Rt_H9 ~ Rt_layer, data = Rt)
Cand.models <- list()
Cand.models[[1]] <- glm_Rt_H9_0
Cand.models[[2]] <- glm_Rt_H9_1
Cand.models[[3]] <- glm_Rt_H9_2
Cand.models[[4]] <- glm_Rt_H9_3
Modnames <- lapply(Cand.models, "formula")
aictab(cand.set = Cand.models, modnames = paste0(Modnames), sort = TRUE)

##
## Model selection based on AICc:
##
##                      K    AICc Delta_AICc AICcWt Cum.Wt    LL
```

```
## Rt_H9 ~ Rt_Period  4 -150.73       0.00   0.53   0.53 79.47
## Rt_H9 ~ 1          2 -150.21       0.53   0.41   0.93 77.13
## Rt_H9 ~ Rt_Blocs   9 -146.61       4.13   0.07   1.00 82.77
## Rt_H9 ~ Rt_layer  30 -112.34      38.39   0.00   1.00 91.61

marginal <- emmeans(glm_Rt_H9_2, ~Rt_Period)
pairs(marginal)

##  contrast      estimate     SE  df t.ratio p.value
##  AnteQ - PostQ   0.0498 0.0317 199   1.571  0.2607
##  AnteQ - Quina  -0.0108 0.0276 199  -0.391  0.9193
##  PostQ - Quina  -0.0606 0.0286 199  -2.118  0.0887
##
## P value adjustment: tukey method for comparing a family of 3 estimates

glm_Rt_H36_0 <- glm(Rt_H36 ~ 1, data = Rt)
glm_Rt_H36_1 <- glm(Rt_H36 ~ Rt_Blocs, data = Rt)
glm_Rt_H36_2 <- glm(Rt_H36 ~ Rt_Period, data = Rt)
glm_Rt_H36_3 <- glm(Rt_H36 ~ Rt_layer, data = Rt)
Cand.models <- list()
Cand.models[[1]] <- glm_Rt_H36_0
Cand.models[[2]] <- glm_Rt_H36_1
Cand.models[[3]] <- glm_Rt_H36_2
Cand.models[[4]] <- glm_Rt_H36_3
Modnames <- lapply(Cand.models, "formula")
aictab(cand.set = Cand.models, modnames = paste0(Modnames), sort = TRUE)

##
## Model selection based on AICc:
##
##                      K   AICc Delta_AICc AICcWt Cum.Wt    LL
## Rt_H36 ~ 1           2 -16.25       0.00   0.79   0.79 10.16
## Rt_H36 ~ Rt_Period   4 -13.59       2.67   0.21   1.00 10.90
## Rt_H36 ~ Rt_Blocs    9  -5.60      10.65   0.00   1.00 12.27
## Rt_H36 ~ Rt_layer   30  33.16      49.41   0.00   1.00 18.86

glm_Rt_H81_0 <- glm(Rt_H81 ~ 1, data = Rt)
glm_Rt_H81_1 <- glm(Rt_H81 ~ Rt_Blocs, data = Rt)
glm_Rt_H81_2 <- glm(Rt_H81 ~ Rt_Period, data = Rt)
glm_Rt_H81_3 <- glm(Rt_H81 ~ Rt_layer, data = Rt)
Cand.models <- list()
Cand.models[[1]] <- glm_Rt_H81_0
Cand.models[[2]] <- glm_Rt_H81_1
Cand.models[[3]] <- glm_Rt_H81_2
Cand.models[[4]] <- glm_Rt_H81_3
Modnames <- lapply(Cand.models, "formula")
aictab(cand.set = Cand.models, modnames = paste0(Modnames), sort = TRUE)

##
## Model selection based on AICc:
##
```

```
##                      K  AICc Delta_AICc AICcWt Cum.Wt     LL
## Rt_H81 ~ 1           2 42.86       0.00   0.82   0.82 -19.40
## Rt_H81 ~ Rt_Period   4 45.99       3.13   0.17   1.00 -18.89
## Rt_H81 ~ Rt_Blocs    9 53.42      10.56   0.00   1.00 -17.24
## Rt_H81 ~ Rt_layer   30 85.11      42.25   0.00   1.00  -7.12
```

Bovinae from Combe-Grenal: inter-block differences:

```
global_db_FOSSILES_BB <- read_delim("global db_FOSSILES_BB.csv", delim = ";", 
escape_double = FALSE, trim_ws = TRUE)
# In blocks E and I, there are only one bovids. They were taken out of as the
ir occurrence avoid performing bf test.
BB<-global_db_FOSSILES_BB
summary (BB)
```

```
##     Group              specie              Period              Blocs          
##  Length:48          Length:48          Length:48          Length:48         
##  Class :character   Class :character   Class :character   Class :character  
##  Mode  :character   Mode  :character   Mode  :character   Mode  :character  
##                                                                             
##                                                                             
##                                                                             
##     layer             ref DMTA          Asfc           epLsar     
##  Length:48          Min.   :  3.00   Min.   :0.996   Min.   :0.427  
##  Class :character   1st Qu.: 28.50   1st Qu.:1.424   1st Qu.:3.275  
##  Mode  :character   Median : 50.00   Median :2.071   Median :4.753  
##                     Mean   : 52.75   Mean   :2.369   Mean   :4.884  
##                     3rd Qu.: 70.50   3rd Qu.:2.699   3rd Qu.:6.986  
##                     Max.   :113.00   Max.   :7.908   Max.   :9.176  
##       Smc             HAsfc9          HAsfc81          HAsfc36      
##  Min.   : 0.304   Min.   :0.0860   Min.   :0.3410   Min.   :0.2200  
##  1st Qu.: 1.008   1st Qu.:0.2057   1st Qu.:0.4682   1st Qu.:0.3772  
##  Median : 1.361   Median :0.2590   Median :0.5385   Median :0.4345  
##  Mean   : 2.651   Mean   :0.3114   Mean   :0.6206   Mean   :0.4769  
##  3rd Qu.: 1.837   3rd Qu.:0.3720   3rd Qu.:0.7360   3rd Qu.:0.5690  
##  Max.   :49.712   Max.   :0.9210   Max.   :1.6210   Max.   :1.1460  
##   Disp-Asfc-i      Disp-epLsar-i      Disp-H9-i      
##  Min.   :0.0000   Min.   :0.0000   Min.   :0.00000  
##  1st Qu.:0.0740   1st Qu.:0.1840   1st Qu.:0.02075  
##  Median :0.1820   Median :0.3010   Median :0.05400  
##  Mean   :0.2410   Mean   :0.3942   Mean   :0.08069  
##  3rd Qu.:0.3665   3rd Qu.:0.4692   3rd Qu.:0.11125  
##  Max.   :1.0870   Max.   :1.2920   Max.   :0.34500
```

```
BB_Species <- BB %>%
  dplyr::select(c(2)) %>%
  unlist(c(1))

BB_Period <- BB %>%
  dplyr::select(c(3)) %>%
  unlist(c(1))
```

```
BB_layer <- BB %>%
  dplyr::select(c(5)) %>%
  unlist(c(1))
BB_Blocs <- BB %>%
  dplyr::select(c(4)) %>%
  unlist(c(1))

BB_Asfc <- BB %>%
  dplyr::select(c(7)) %>%
  unlist(c(1))
BB_epLsar <- BB %>%
  dplyr::select(c(8)) %>%
  unlist(c(1))
BB_Smc <- BB %>%
  dplyr::select(c(9)) %>%
  unlist(c(1))
BB_H9 <- BB %>%
  dplyr::select(c(10)) %>%
  unlist(c(1))
BB_H36 <- BB %>%
  dplyr::select(c(12)) %>%
  unlist(c(1))
BB_H81 <- BB %>%
  dplyr::select(c(11)) %>%
  unlist(c(1))
```

Data exploration

Search for Zeros

```
sum(BB_Asfc == 0, na.rm = TRUE) * 100 / nrow(BB)

## [1] 0

sum(BB_epLsar == 0, na.rm = TRUE) * 100 / nrow(BB)

## [1] 0

sum(BB_Smc == 0, na.rm = TRUE) * 100 / nrow(BB)

## [1] 0

sum(BB_H9 == 0, na.rm = TRUE) * 100 / nrow(BB)

## [1] 0

sum(BB_H36 == 0, na.rm = TRUE) * 100 / nrow(BB)

## [1] 0

sum(BB_H81 == 0, na.rm = TRUE) * 100 / nrow(BB)

## [1] 0
```

Search for missing data:

```
colSums(is.na(BB))

##          Group         specie         Period          Blocs          layer
##              0              0              0              0              0
##       ref DMTA           Asfc         epLsar            Smc         HAsfc9
##              0              0              0              0              0
##        HAsfc81        HAsfc36    Disp-Asfc-i Disp-epLsar-i      Disp-H9-i
##              0              0              0              0              0
```

Checking data distribution and outliers:

```
x <- BB[order(BB_Asfc), ]
x$Blocs <- factor(x$Blocs)
dotchart(x$Asfc, cex = 1, pch = 16, groups = x$Blocks, xlab = "BB_Asfc per Bl
ock")
```

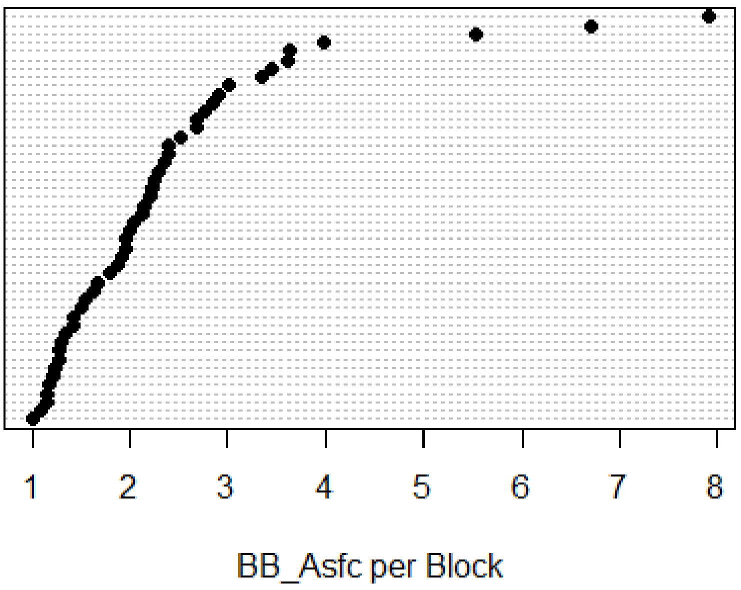


```
x <- BB[order(BB_epLsar), ]
x$Blocs <- factor(x$Blocs)
dotchart(x$epLsar, cex = 1, pch = 16, groups = x$Blocks, xlab = "BB_epLsar pe
r Block")
```

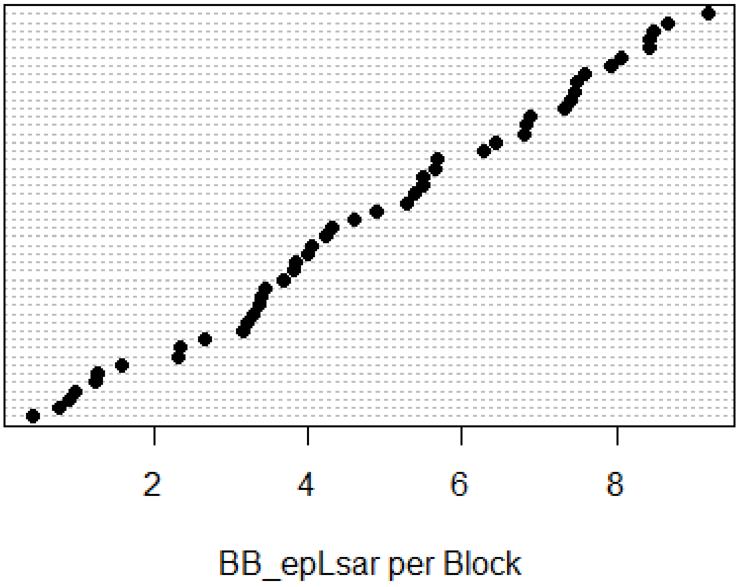


```
x <- BB[order(BB_Smc), ]
x$Blocs <- factor(x$Blocs)
dotchart(x$Smc, cex = 1, pch = 16, groups = x$Blocks, xlab = "BB_Smc per Bloc
k")
```

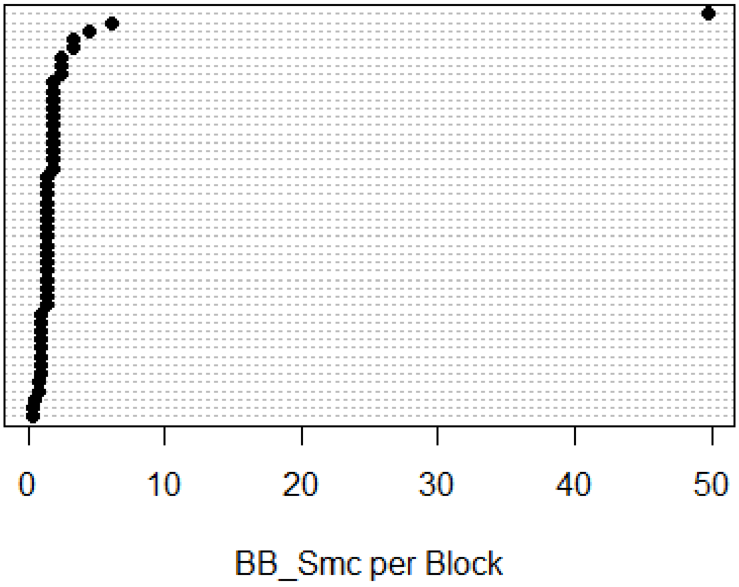


```
x <- BB[order(BB_H9), ]
x$Blocs <- factor(x$Blocs)
dotchart(x$HAsfc9, cex = 1, pch = 16, groups = x$Blocks, xlab = "BB_HAsfc9 pe
r Block")
```

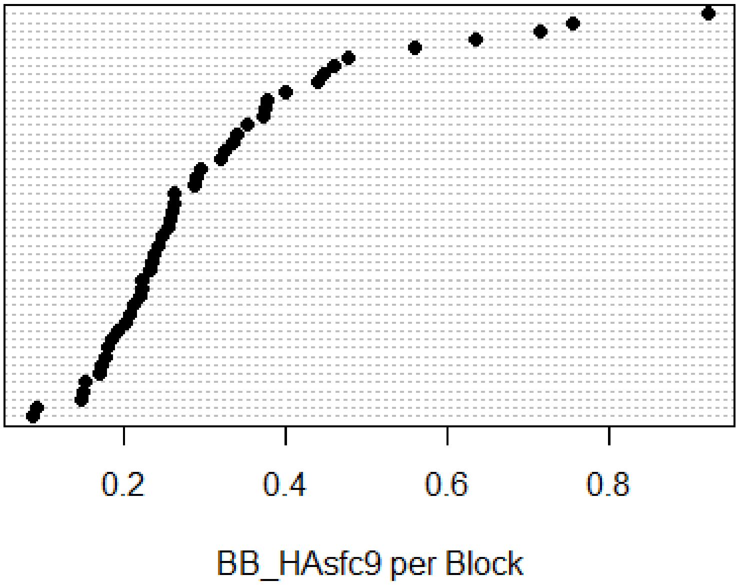


```
x <- BB[order(BB_H36), ]
x$Blocs <- factor(x$Blocs)
dotchart(x$HAsfc36, cex = 1, pch = 16, groups = x$Blocks, xlab = "BB_HAsfc36
per Block")
```

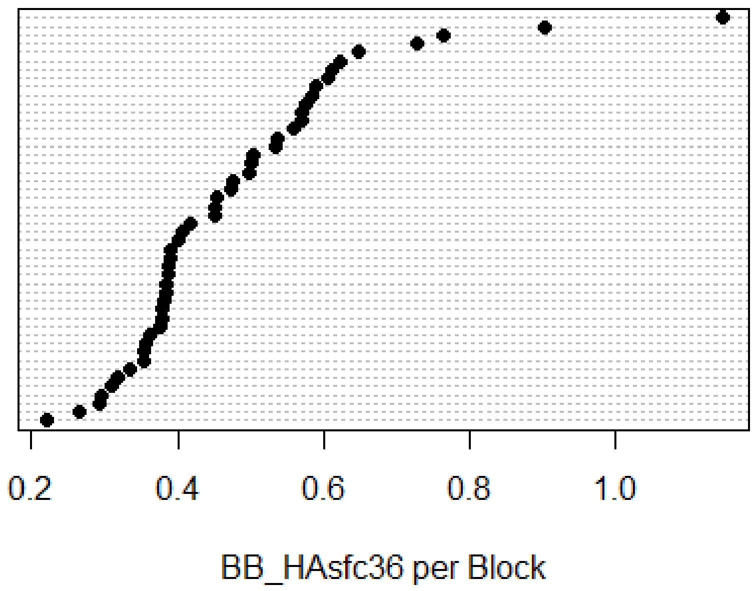


```
x <- BB[order(BB_H81), ]
x$Blocs <- factor(x$Blocs)
dotchart(x$HAsfc81, cex = 1, pch = 16, groups = x$Blocks, xlab = "BB_HAsfc81
per Block")
```

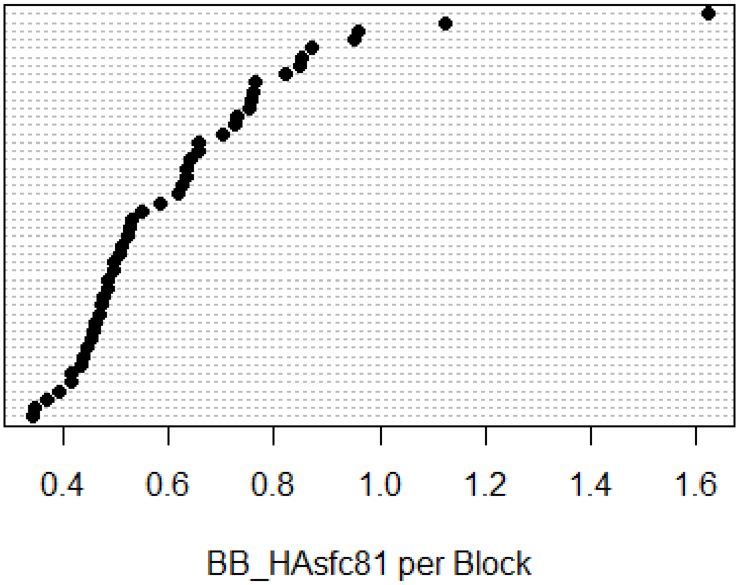

Graphical evaluation of the tests' applicability:

Normality and homoscedasticity of the variables. ##### Normality

```
ggplot(BB) +
  geom_freqpoly(aes(x = Asfc), bins = 7) +
  labs(
    x = "Value Asfc",
    y = "Frequency"
  )
```

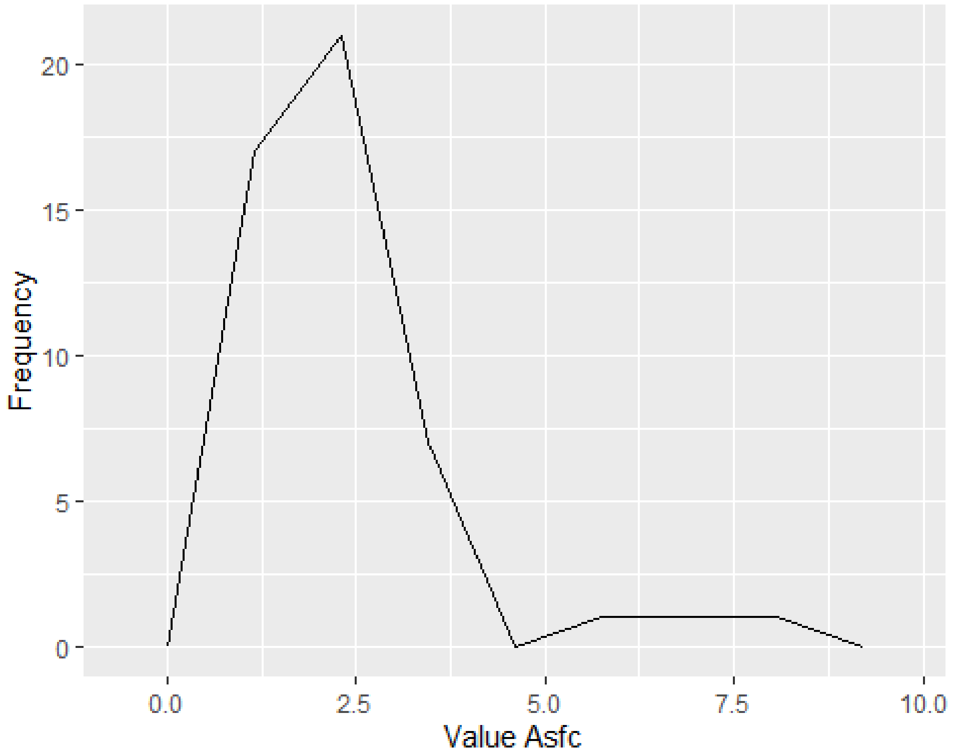


```r
ggplot(BB) +
  geom_freqpoly(aes(x = epLsar), bins = 7) +
  labs(
    x = "Value epLsar",
    y = "Frequency"
  )
```

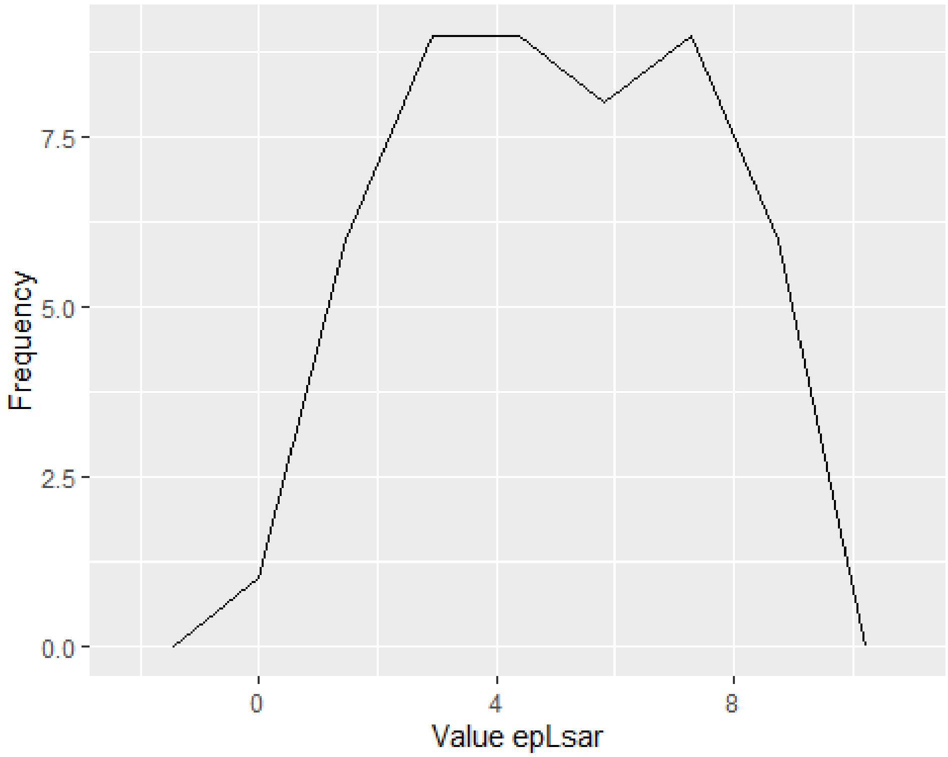


```
ggplot(BB) +
  geom_freqpoly(aes(x = Smc), bins = 7) +
  labs(
    x = "Value Smc",
    y = "Frequency"
  )
```

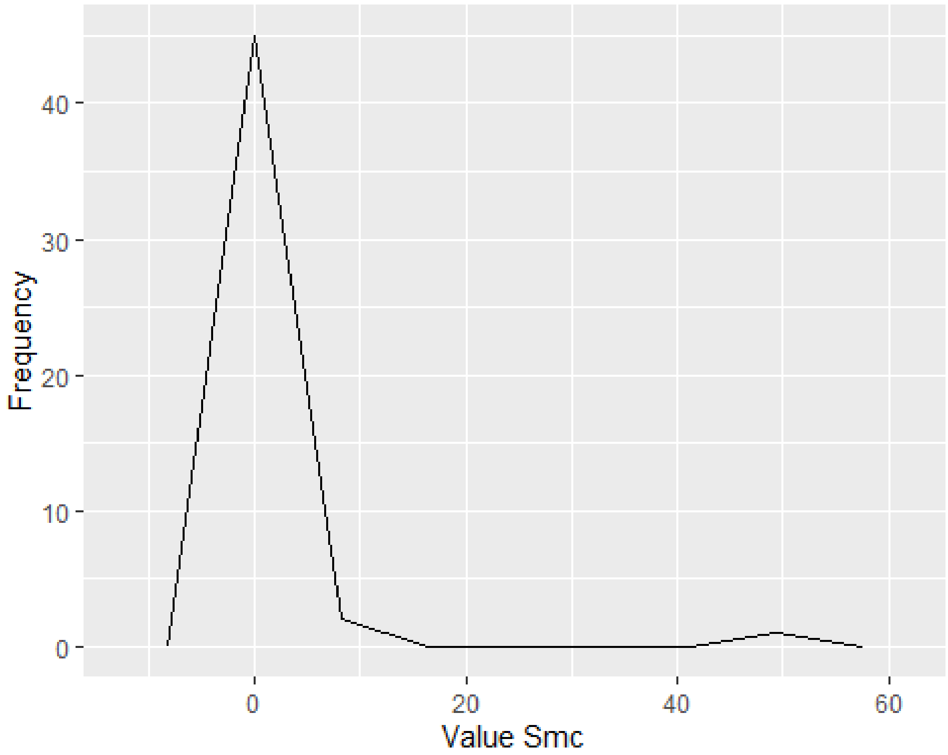


```
ggplot(BB) +
  geom_freqpoly(aes(x = HAsfc9), bins = 7) +
  labs(
    x = "Value H9",
    y = "Frequency"
  )
```

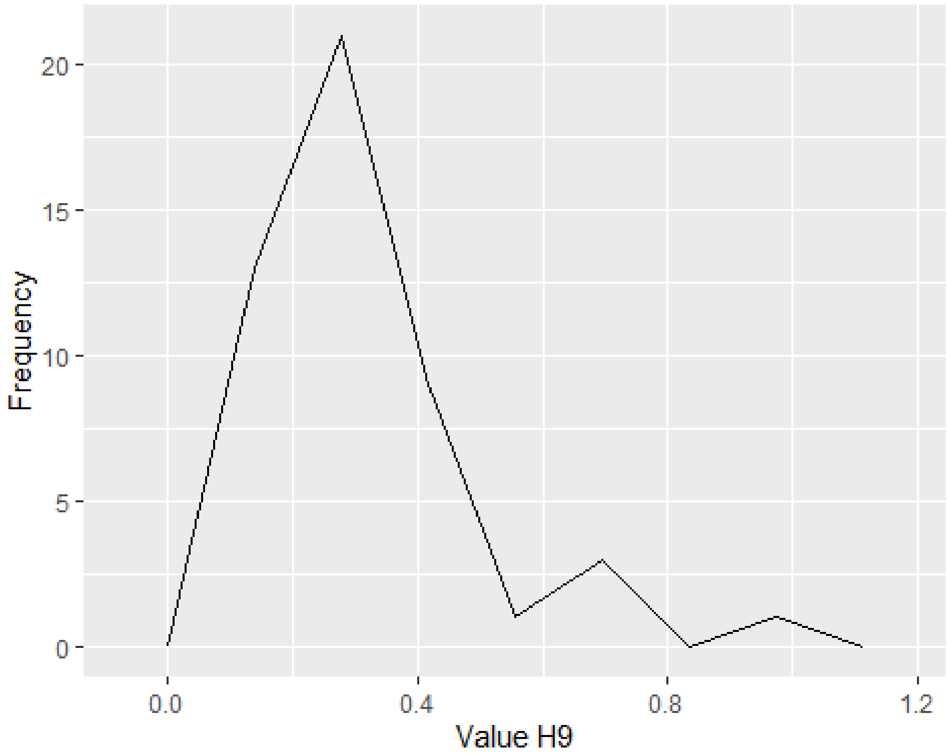


```
ggplot(BB) +
  geom_freqpoly(aes(x = HAsfc36), bins = 7) +
  labs(
    x = "Value H36",
    y = "Frequency"
  )
```

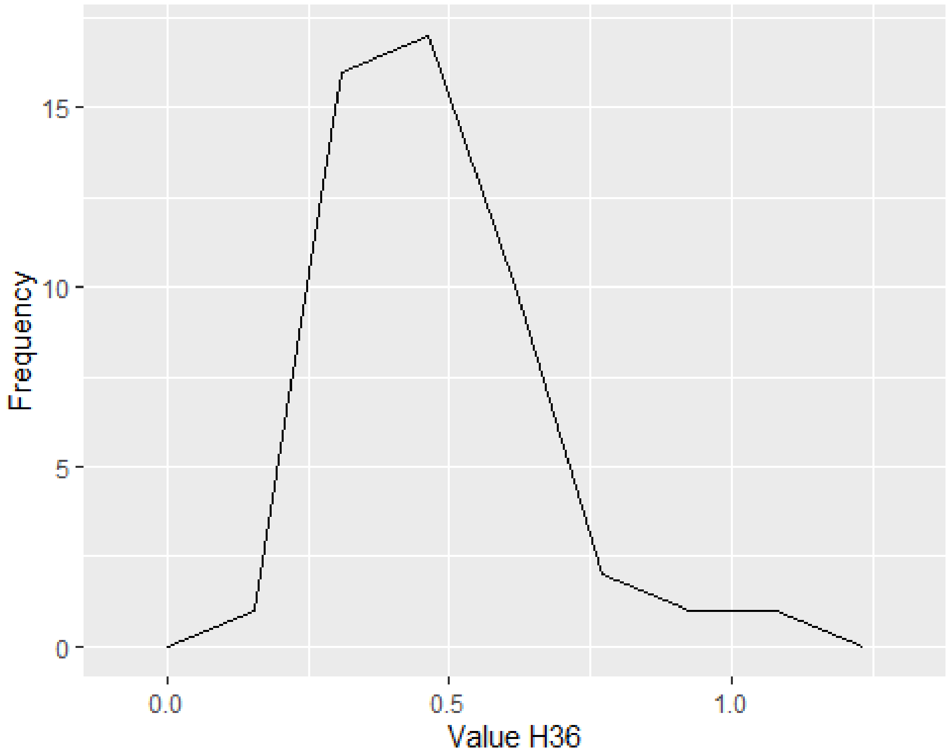


```
ggplot(BB) +
  geom_freqpoly(aes(x = HAsfc81), bins = 7) +
  labs(
    x = "Value H81",
    y = "Frequency"
  )
```

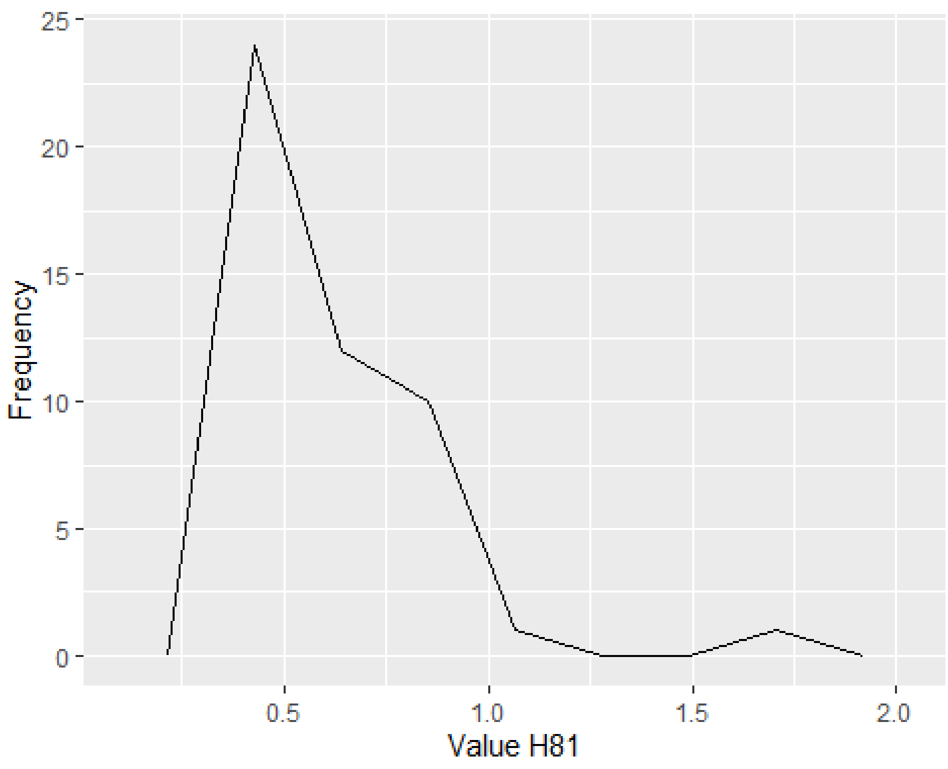

Homoscedasticity: Brown & Forsythe test (and data transformation whenever needed)

```
bf.test(BB_Asfc ~ BB_Blocs, data = BB)

##
##   Brown-Forsythe Test (alpha = 0.05)
## -------------------------------------------------------------
##   data : BB_Asfc and BB_Blocs
##
##   statistic  : 0.740204
##   num df     : 3
##   denom df   : 8.171339
##   p.value    : 0.5566975
##
##   Result     : Difference is not statistically significant.
## -------------------------------------------------------------

ggplot(BB) +
  geom_boxplot(aes(x = BB_Blocs, y = BB_Asfc)) +
  labs(
    x = "Blocks",
    y = "Asfc"
  )
```

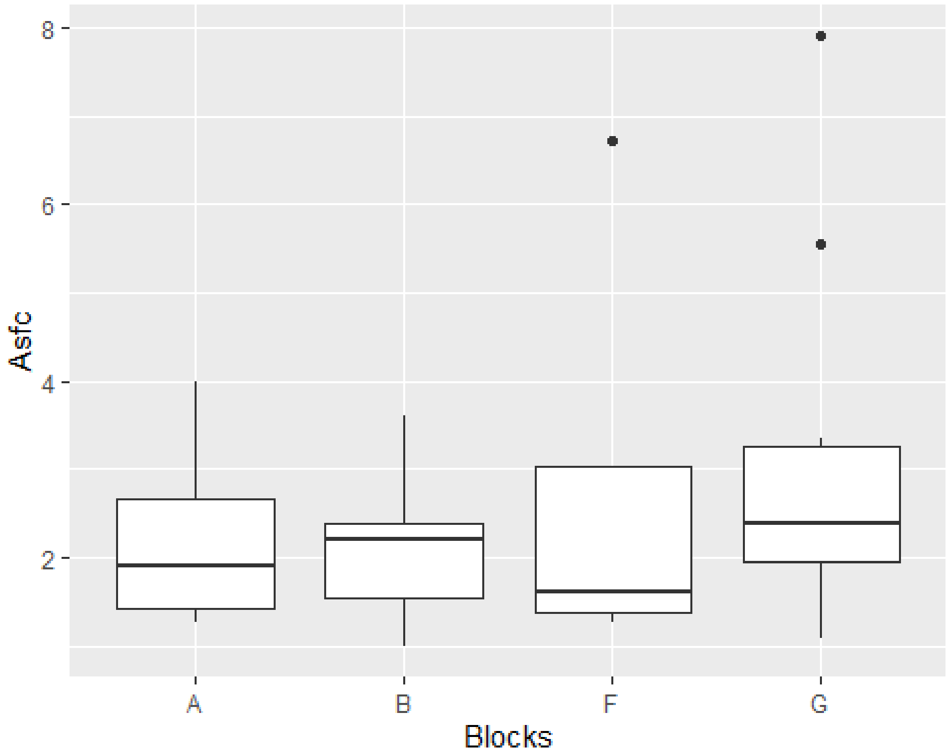


```
bf.test(BB_epLsar ~ BB_Blocs, data = BB)

##
##   Brown-Forsythe Test (alpha = 0.05)
## -------------------------------------------------------------
##   data : BB_epLsar and BB_Blocs
##
##   statistic  : 0.8613846
##   num df     : 3
##   denom df   : 16.57939
##   p.value    : 0.4805002
##
##   Result     : Difference is not statistically significant.
## -------------------------------------------------------------

ggplot(BB) +
  geom_boxplot(aes(x = BB_Blocs, y = BB_epLsar)) +
  labs(
    x = "Blocks",
    y = "epLsar"
  )
```

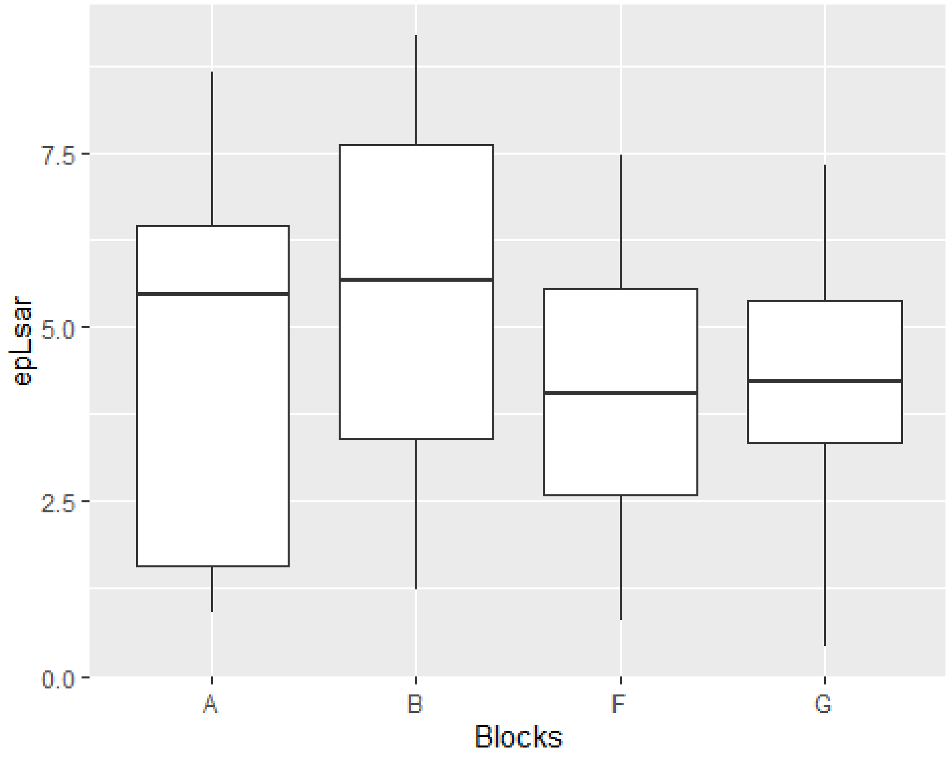


```
bf.test(BB_Smc ~ BB_Blocs, data = BB)

##
##   Brown-Forsythe Test (alpha = 0.05)
## -------------------------------------------------------------
##   data : BB_Smc and BB_Blocs
##
##   statistic  : 1.041545
##   num df     : 3
##   denom df   : 3.018441
##   p.value    : 0.4865402
##
##   Result     : Difference is not statistically significant.
## -------------------------------------------------------------

ggplot(BB) +
  geom_boxplot(aes(x = BB_Blocs, y = BB_Smc)) +
  labs(
    x = "Blocks",
    y = "Smc"
  )
```

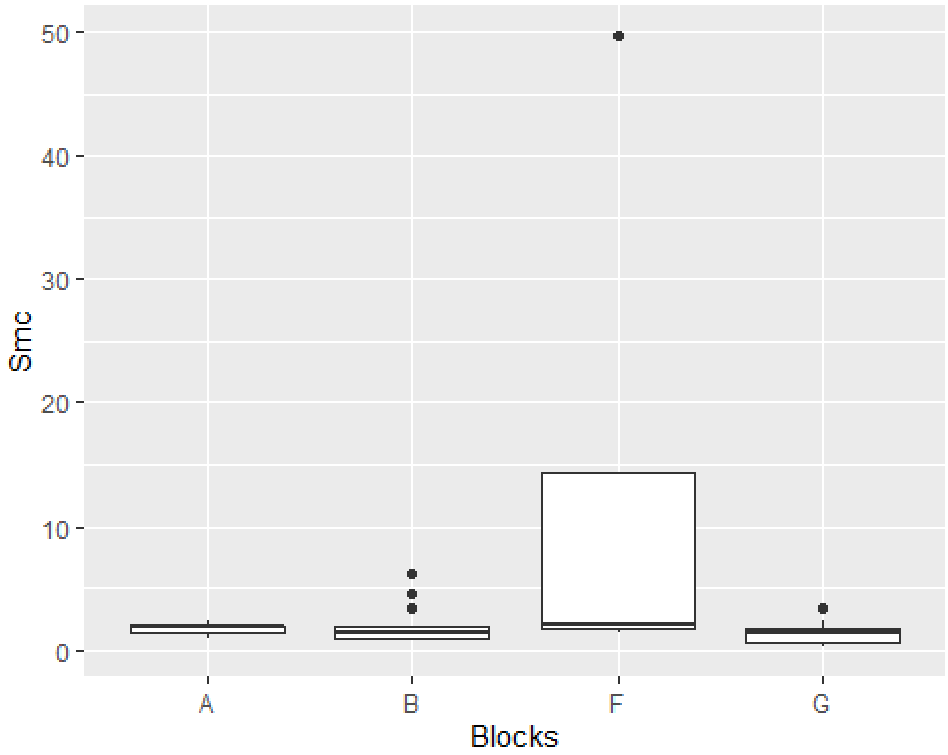


```
bf.test(BB_H9 ~ BB_Blocs, data = BB)

##
##   Brown-Forsythe Test (alpha = 0.05)
## -------------------------------------------------------------
##   data : BB_H9 and BB_Blocs
##
##   statistic  : 1.46713
##   num df     : 3
##   denom df   : 7.578074
##   p.value    : 0.2983018
##
##   Result     : Difference is not statistically significant.
## -------------------------------------------------------------

ggplot(BB) +
  geom_boxplot(aes(x = BB_Blocs, y = BB_H9)) +
  labs(
    x = "Blocks",
    y = "H9"
  )
```

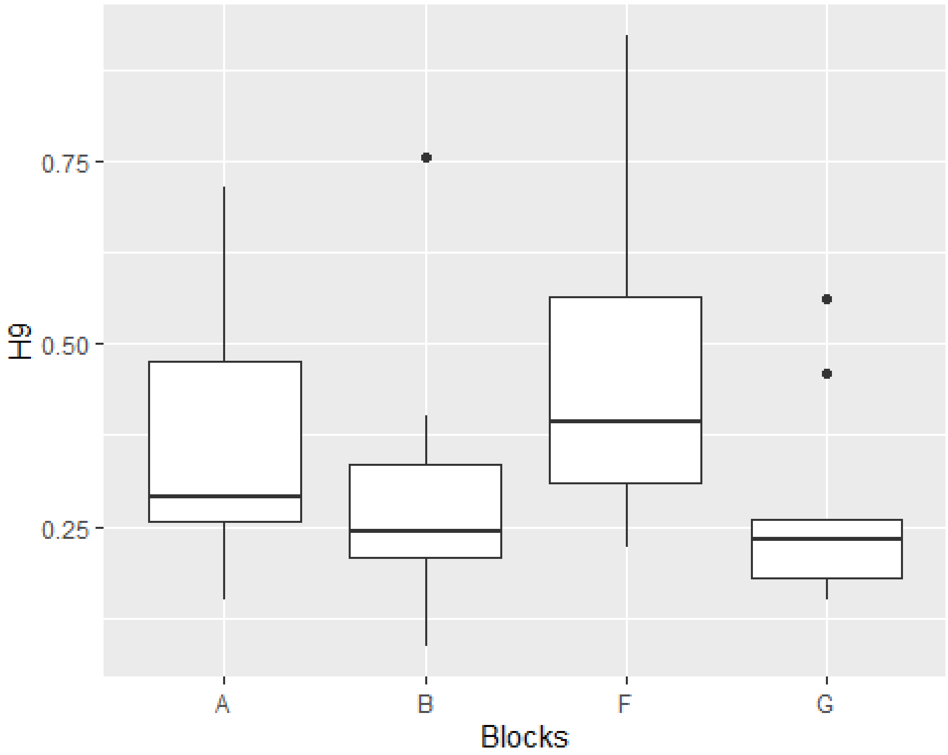


```
bf.test(BB_H36 ~ BB_Blocs, data = BB)

##
##   Brown-Forsythe Test (alpha = 0.05)
## -------------------------------------------------------------
##   data : BB_H36 and BB_Blocs
##
##   statistic  : 1.221552
##   num df     : 3
##   denom df   : 6.522412
##   p.value    : 0.3750121
##
##   Result     : Difference is not statistically significant.
## -------------------------------------------------------------

ggplot(BB) +
  geom_boxplot(aes(x = BB_Blocs, y = BB_H36)) +
  labs(
    x = "Blocks",
    y = "H36"
  )
```

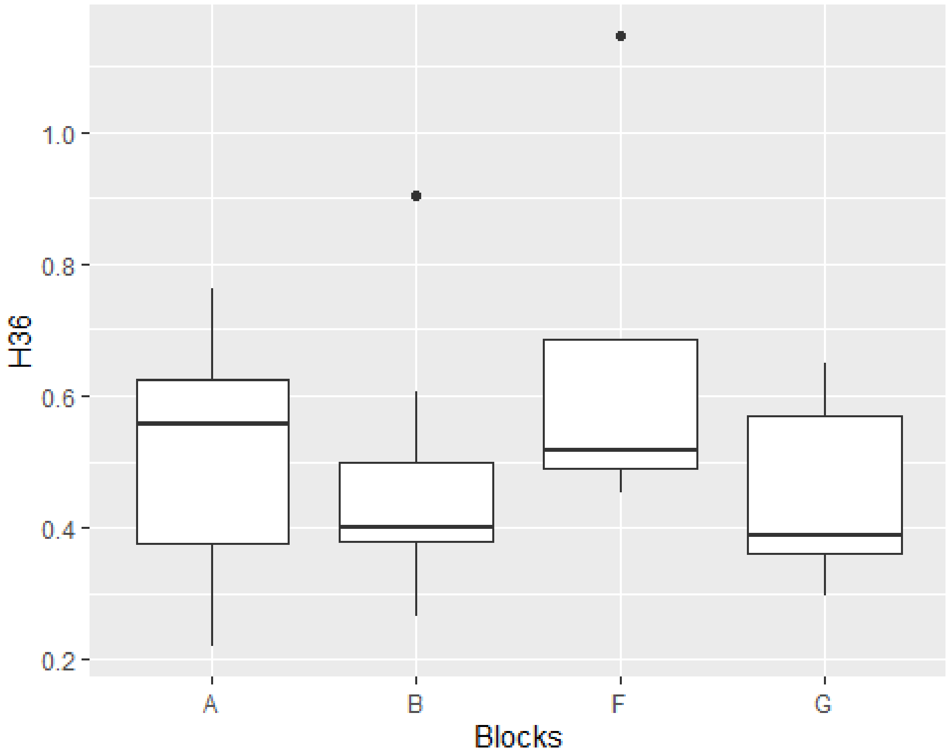


```
bf.test(BB_H81 ~ BB_Blocs, data = BB)

##
##   Brown-Forsythe Test (alpha = 0.05)
## -------------------------------------------------------------
##   data : BB_H81 and BB_Blocs
##
##   statistic  : 1.711175
##   num df     : 3
##   denom df   : 5.710132
##   p.value    : 0.2675153
##
##   Result     : Difference is not statistically significant.
## -------------------------------------------------------------

ggplot(BB) +
  geom_boxplot(aes(x = BB_Blocs, y = BB_H81)) +
  labs(
    x = "Blocks",
    y = "H81"
  )
```

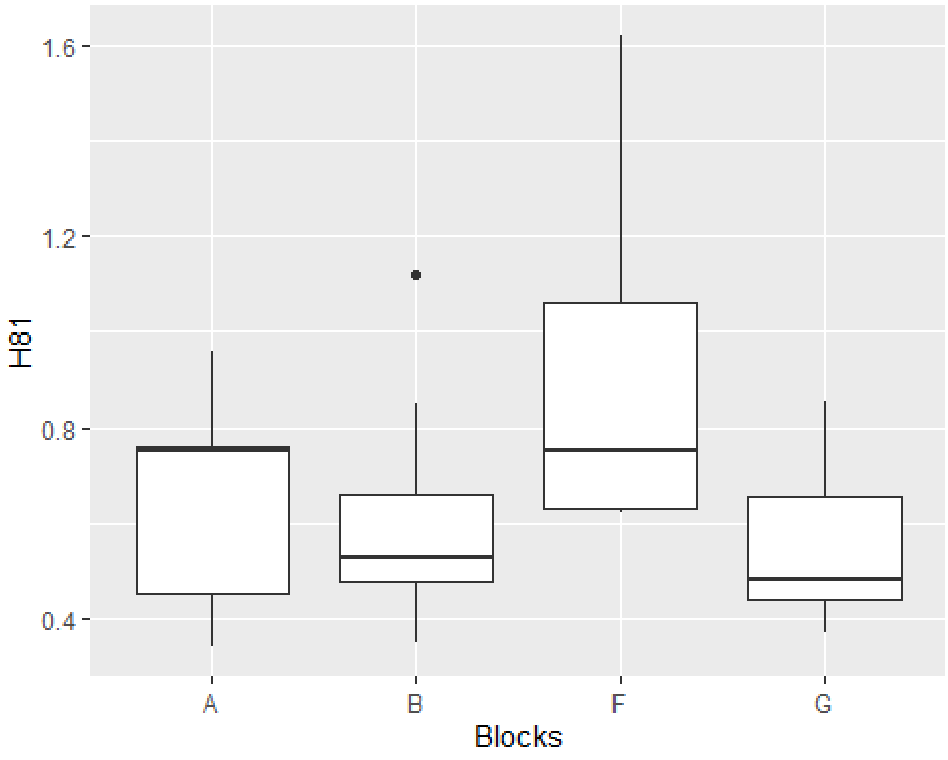


```
bf.test(BB_Asfc ~ BB_Period, data = BB)

##
##   Brown-Forsythe Test (alpha = 0.05)
## -------------------------------------------------------------
##   data : BB_Asfc and BB_Period
##
##   statistic  : 0.7994627
##   num df     : 2
##   denom df   : 6.914003
##   p.value    : 0.4871491
##
##   Result     : Difference is not statistically significant.
## -------------------------------------------------------------

ggplot(BB) +
  geom_boxplot(aes(x = BB_Period, y = BB_Asfc)) +
  labs(
    x = "Period",
    y = "Asfc"
  )
```

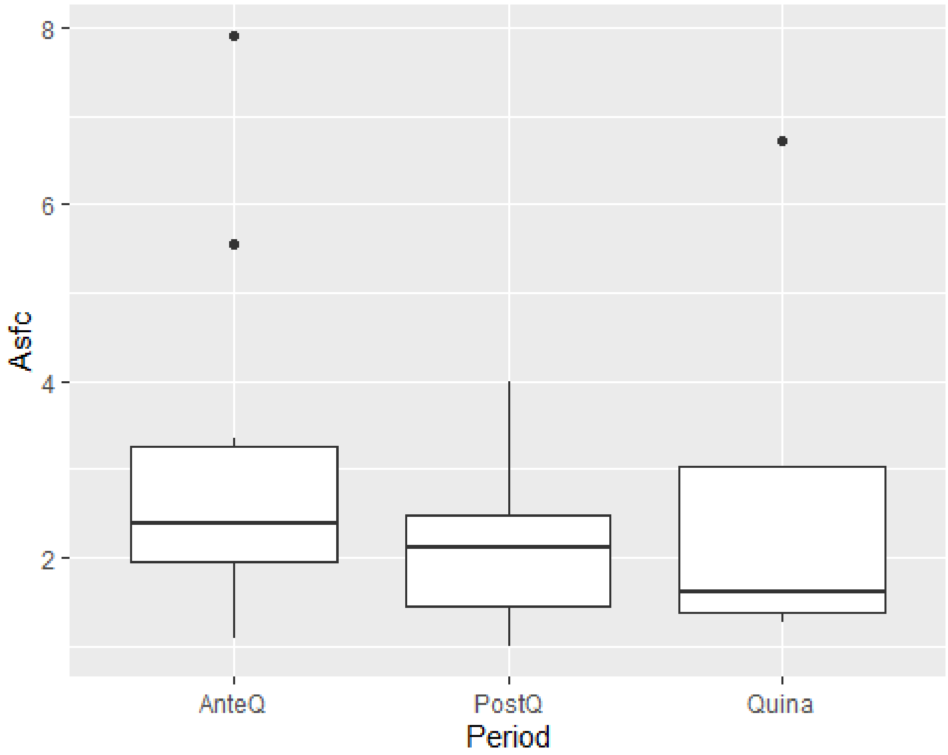


```
bf.test(BB_epLsar ~ BB_Period, data = BB)

##
##   Brown-Forsythe Test (alpha = 0.05)
## -------------------------------------------------------------
##   data : BB_epLsar and BB_Period
##
##   statistic  : 0.840351
##   num df     : 2
##   denom df   : 8.628661
##   p.value    : 0.4640366
##
##   Result     : Difference is not statistically significant.
## -------------------------------------------------------------

ggplot(BB) +
  geom_boxplot(aes(x = BB_Period, y = BB_epLsar)) +
  labs(
    x = "Period",
    y = "epLsar"
  )
```

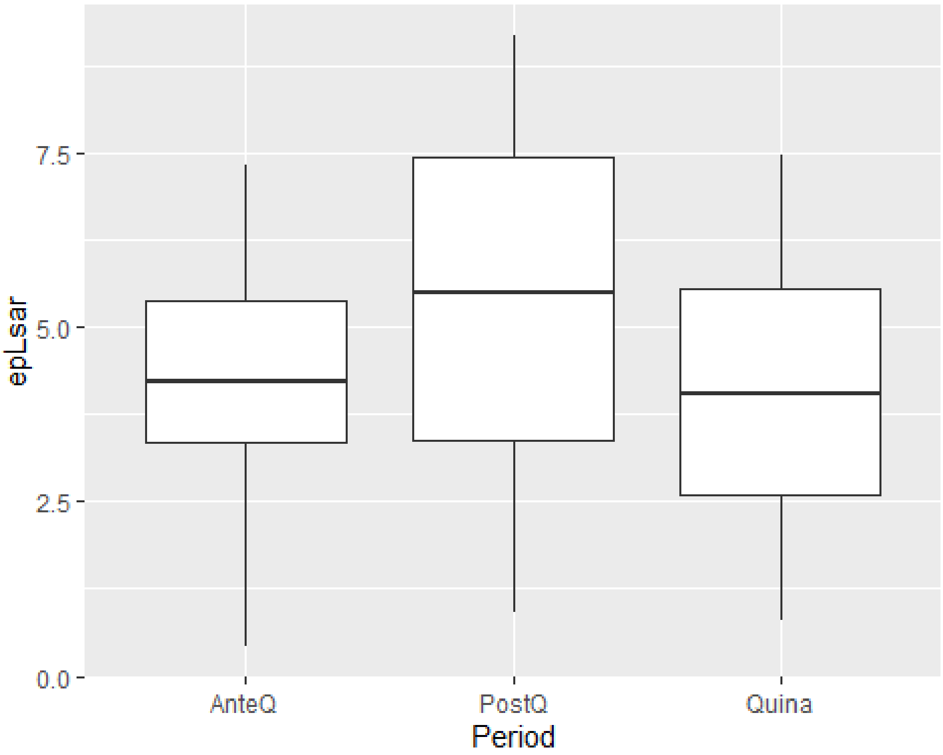


```
bf.test(BB_Smc ~ BB_Period, data = BB)

##
##   Brown-Forsythe Test (alpha = 0.05)
## -------------------------------------------------------------
##   data : BB_Smc and BB_Period
##
##   statistic  : 1.042631
##   num df     : 2
##   denom df   : 3.012033
##   p.value    : 0.4527989
##
##   Result     : Difference is not statistically significant.
## -------------------------------------------------------------

ggplot(BB) +
  geom_boxplot(aes(x = BB_Period, y = BB_Smc)) +
  labs(
    x = "Period",
    y = "Smc"
  )
```

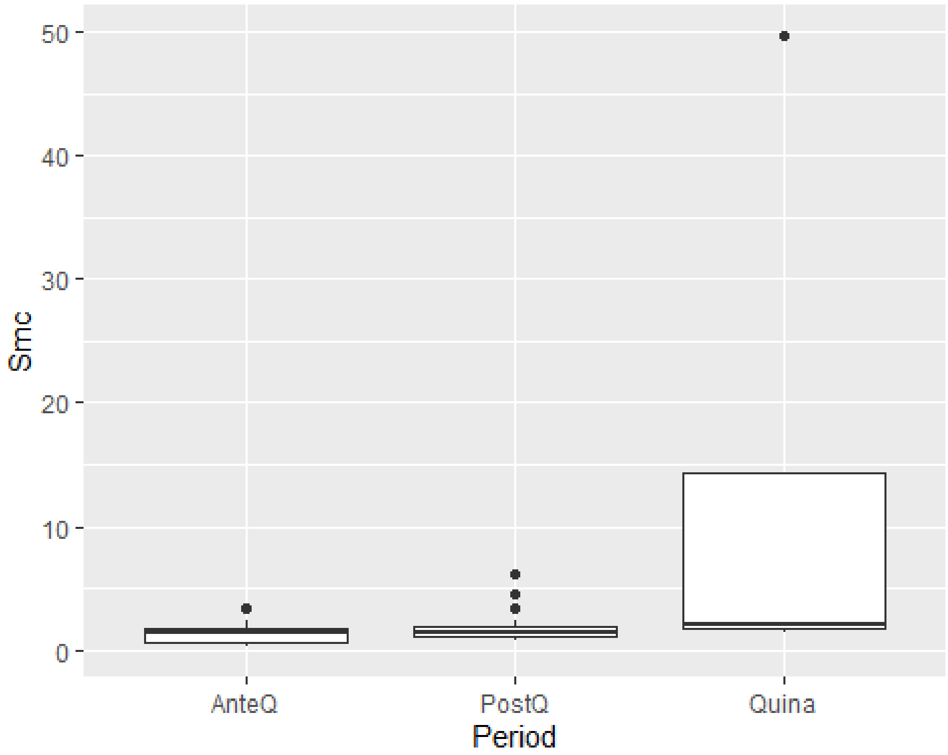


```
bf.test(BB_H9 ~ BB_Period, data = BB)

##
##   Brown-Forsythe Test (alpha = 0.05)
## -------------------------------------------------------------
##   data : BB_H9 and BB_Period
##
##   statistic  : 1.26912
##   num df     : 2
##   denom df   : 4.651687
##   p.value    : 0.363204
##
##   Result     : Difference is not statistically significant.
## -------------------------------------------------------------

ggplot(BB) +
  geom_boxplot(aes(x = BB_Period, y = BB_H9)) +
  labs(
    x = "Period",
    y = "H9"
  )
```

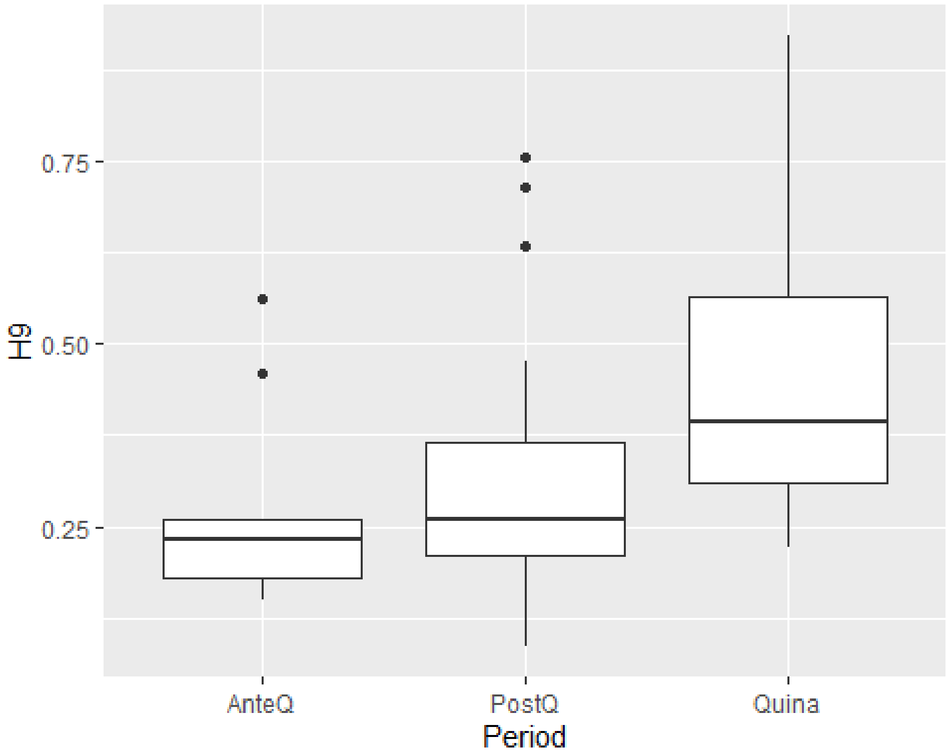


```
bf.test(BB_H36 ~ BB_Period, data = BB)

##
##   Brown-Forsythe Test (alpha = 0.05)
## -------------------------------------------------------------
##   data : BB_H36 and BB_Period
##
##   statistic  : 1.245816
##   num df     : 2
##   denom df   : 4.298312
##   p.value    : 0.3743224
##
##   Result     : Difference is not statistically significant.
## -------------------------------------------------------------

ggplot(BB) +
  geom_boxplot(aes(x = BB_Period, y = BB_H36)) +
  labs(
    x = "Period",
    y = "H36"
  )
```

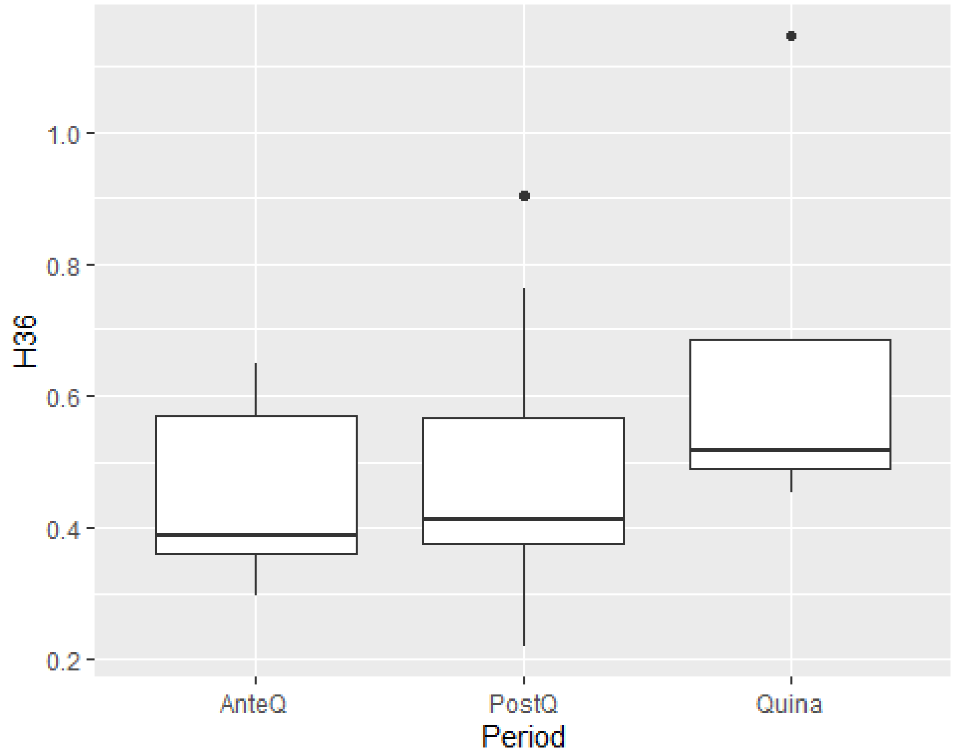


```
bf.test(BB_H81 ~ BB_Period, data = BB)

##
##   Brown-Forsythe Test (alpha = 0.05)
## -------------------------------------------------------------
##   data : BB_H81 and BB_Period
##
##   statistic  : 1.895356
##   num df     : 2
##   denom df   : 4.059262
##   p.value    : 0.262223
##
##   Result     : Difference is not statistically significant.
## -------------------------------------------------------------

ggplot(BB) +
  geom_boxplot(aes(x = BB_Period, y = BB_H81)) +
  labs(
    x = "Period",
    y = "H81"
  )
```

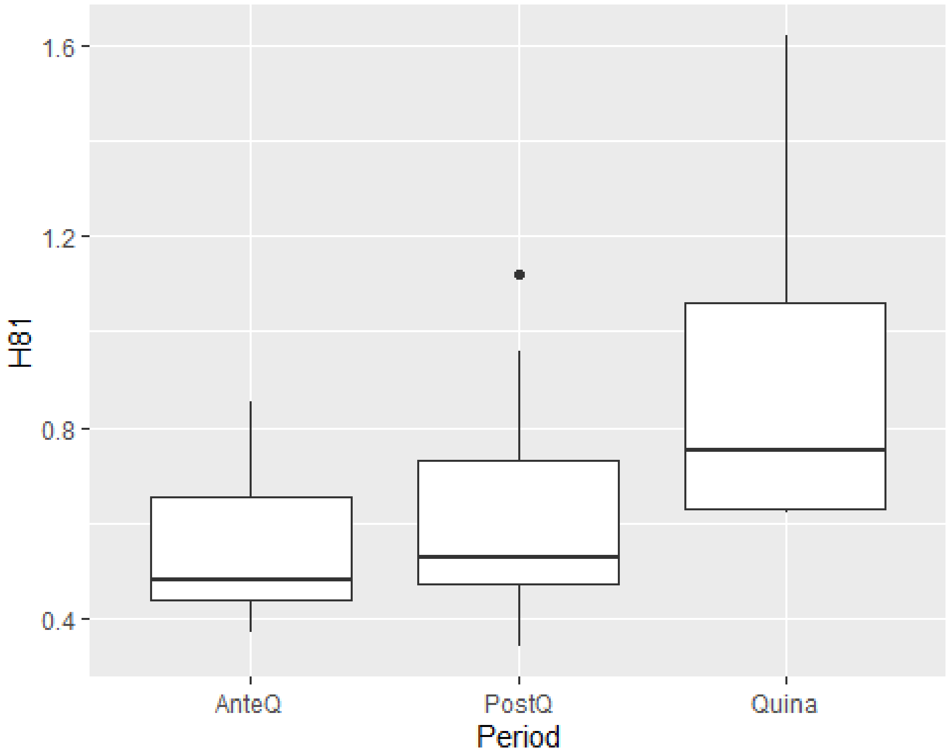

GLM analyses

What is the impact of random (1), blocks (BB_Blocks) and period (BB_Period) over each DMTA parameter?

```
glm_BB_Asfc0 <- glm(BB_Asfc ~ 1, data = BB)
glm_BB_Asfc1 <- glm(BB_Asfc ~ BB_Blocs, data = BB)
glm_BB_Asfc2 <- glm(BB_Asfc ~ BB_Period, data = BB)
glm_BB_Asfc3 <- glm(BB_Asfc ~ BB_layer, data = BB)

Cand.models <- list()
Cand.models[[1]] <- glm_BB_Asfc0
Cand.models[[2]] <- glm_BB_Asfc1
Cand.models[[3]] <- glm_BB_Asfc2
Cand.models[[4]] <- glm_BB_Asfc3
Modnames <- lapply(Cand.models, "formula")
aictab(cand.set = Cand.models, modnames = paste0(Modnames), sort = TRUE)

##
## Model selection based on AICc:
##
##                        K   AICc Delta_AICc AICcWt Cum.Wt     LL
## BB_Asfc ~ 1            2 170.63       0.00   0.45   0.45 -83.18
## BB_Asfc ~ BB_Period    4 170.81       0.17   0.41   0.87 -80.94
## BB_Asfc ~ BB_Blocs     5 173.26       2.62   0.12   0.99 -80.91
## BB_Asfc ~ BB_layer    15 177.73       7.09   0.01   1.00 -66.36
```

```r
marginal <- emmeans(glm_BB_Asfc2, ~BB_Period)
pairs(marginal)
```

```
##  contrast      estimate    SE df t.ratio p.value
##  AnteQ - PostQ    0.967 0.485 45   1.993  0.1256
##  AnteQ - Quina    0.278 0.798 45   0.349  0.9352
##  PostQ - Quina   -0.689 0.713 45  -0.966  0.6019
##
## P value adjustment: tukey method for comparing a family of 3 estimates
```

```r
glm_BB_epLsar0 <- glm(BB_epLsar ~ 1, data = BB)
glm_BB_epLsar1 <- glm(BB_epLsar ~ BB_Blocs, data = BB)
glm_BB_epLsar2 <- glm(BB_epLsar ~ BB_Period, data = BB)
glm_BB_epLsar3 <- glm(BB_epLsar ~ BB_layer, data = BB)
Cand.models <- list()
Cand.models[[1]] <- glm_BB_epLsar0
Cand.models[[2]] <- glm_BB_epLsar1
Cand.models[[3]] <- glm_BB_epLsar2
Cand.models[[4]] <- glm_BB_epLsar3
Modnames <- lapply(Cand.models, "formula")
aictab(cand.set = Cand.models, modnames = paste0(Modnames), sort = TRUE)
```

```
##
## Model selection based on AICc:
##
##                         K   AICc Delta_AICc AICcWt Cum.Wt      LL
## BB_epLsar ~ 1           2 225.86       0.00   0.73   0.73 -110.80
## BB_epLsar ~ BB_Period   4 228.66       2.80   0.18   0.91 -109.87
## BB_epLsar ~ BB_Blocs    5 229.99       4.13   0.09   1.00 -109.28
## BB_epLsar ~ BB_layer   15 242.55      16.69   0.00   1.00  -98.78
```

```r
glm_BB_Smc0 <- glm(BB_Smc ~ 1, data = BB)
glm_BB_Smc1 <- glm(BB_Smc ~ BB_Blocs, data = BB)
glm_BB_Smc2 <- glm(BB_Smc ~ BB_Period, data = BB)
glm_BB_Smc3 <- glm(BB_Smc ~ BB_layer, data = BB)
Cand.models <- list()
Cand.models[[1]] <- glm_BB_Smc0
Cand.models[[2]] <- glm_BB_Smc1
Cand.models[[3]] <- glm_BB_Smc2
Cand.models[[4]] <- glm_BB_Smc3
Modnames <- lapply(Cand.models, "formula")
aictab(cand.set = Cand.models, modnames = paste0(Modnames), sort = TRUE)
```

```
##
## Model selection based on AICc:
##
##                      K   AICc Delta_AICc AICcWt Cum.Wt      LL
## BB_Smc ~ BB_layer   15 146.69       0.00      1      1  -50.84
## BB_Smc ~ BB_Period   4 318.07     171.38      0      1 -154.57
## BB_Smc ~ BB_Blocs    5 320.57     173.88      0      1 -154.57
## BB_Smc ~ 1           2 326.40     179.72      0      1 -161.07
```

```
marginal <- emmeans(glm_BB_Smc3, ~BB_layer)
pairs(marginal)
```

```
##  contrast  estimate    SE df t.ratio p.value
##  c10 - c11  -0.0254 0.397 34  -0.064  1.0000
##  c10 - c12   0.0205 0.677 34   0.030  1.0000
##  c10 - c14   0.4197 0.479 34   0.877  0.9997
##  c10 - c15  -4.4745 0.896 34  -4.996  0.0012
##  c10 - c21   0.0205 0.677 34   0.030  1.0000
##  c10 - c22  -0.8595 0.896 34  -0.960  0.9992
##  c10 - c24 -48.0925 0.896 34 -53.696  <.0001
##  c10 - c29   0.1548 0.586 34   0.264  1.0000
##  c10 - c30   0.3772 0.535 34   0.705  1.0000
##  c10 - c32   0.0668 0.586 34   0.114  1.0000
##  c10 - c4   -0.8595 0.896 34  -0.960  0.9992
##  c10 - c6    0.6115 0.896 34   0.683  1.0000
##  c10 - c9   -0.2175 0.896 34  -0.243  1.0000
##  c11 - c12   0.0459 0.622 34   0.074  1.0000
##  c11 - c14   0.4450 0.397 34   1.121  0.9965
##  c11 - c15  -4.4491 0.855 34  -5.205  0.0007
##  c11 - c21   0.0459 0.622 34   0.074  1.0000
##  c11 - c22  -0.8341 0.855 34  -0.976  0.9991
##  c11 - c24 -48.0671 0.855 34 -56.237  <.0001
##  c11 - c29   0.1802 0.522 34   0.345  1.0000
##  c11 - c30   0.4026 0.464 34   0.869  0.9997
##  c11 - c32   0.0922 0.522 34   0.177  1.0000
##  c11 - c4   -0.8341 0.855 34  -0.976  0.9991
##  c11 - c6    0.6369 0.855 34   0.745  1.0000
##  c11 - c9   -0.1921 0.855 34  -0.225  1.0000
##  c12 - c14   0.3992 0.677 34   0.590  1.0000
##  c12 - c15  -4.4950 1.016 34  -4.426  0.0060
##  c12 - c21   0.0000 0.829 34   0.000  1.0000
##  c12 - c22  -0.8800 1.016 34  -0.867  0.9997
##  c12 - c24 -48.1130 1.016 34 -47.376  <.0001
##  c12 - c29   0.1343 0.757 34   0.177  1.0000
##  c12 - c30   0.3568 0.718 34   0.497  1.0000
##  c12 - c32   0.0463 0.757 34   0.061  1.0000
##  c12 - c4   -0.8800 1.016 34  -0.867  0.9997
##  c12 - c6    0.5910 1.016 34   0.582  1.0000
##  c12 - c9   -0.2380 1.016 34  -0.234  1.0000
##  c14 - c15  -4.8942 0.896 34  -5.464  0.0003
##  c14 - c21  -0.3992 0.677 34  -0.590  1.0000
##  c14 - c22  -1.2792 0.896 34  -1.428  0.9713
##  c14 - c24 -48.5122 0.896 34 -54.165  <.0001
##  c14 - c29  -0.2648 0.586 34  -0.452  1.0000
##  c14 - c30  -0.0424 0.535 34  -0.079  1.0000
##  c14 - c32  -0.3528 0.586 34  -0.602  1.0000
##  c14 - c4   -1.2792 0.896 34  -1.428  0.9713
##  c14 - c6    0.1918 0.896 34   0.214  1.0000
##  c14 - c9   -0.6372 0.896 34  -0.711  1.0000
```

```
##   c15 - c21    4.4950 1.016 34   4.426  0.0060
##   c15 - c22    3.6150 1.173 34   3.083  0.1589
##   c15 - c24 -43.6180 1.173 34 -37.195  <.0001
##   c15 - c29    4.6293 0.957 34   4.835  0.0019
##   c15 - c30    4.8518 0.927 34   5.233  0.0006
##   c15 - c32    4.5413 0.957 34   4.743  0.0025
##   c15 - c4     3.6150 1.173 34   3.083  0.1589
##   c15 - c6     5.0860 1.173 34   4.337  0.0077
##   c15 - c9     4.2570 1.173 34   3.630  0.0471
##   c21 - c22   -0.8800 1.016 34  -0.867  0.9997
##   c21 - c24 -48.1130 1.016 34 -47.376  <.0001
##   c21 - c29    0.1343 0.757 34   0.177  1.0000
##   c21 - c30    0.3568 0.718 34   0.497  1.0000
##   c21 - c32    0.0463 0.757 34   0.061  1.0000
##   c21 - c4    -0.8800 1.016 34  -0.867  0.9997
##   c21 - c6     0.5910 1.016 34   0.582  1.0000
##   c21 - c9    -0.2380 1.016 34  -0.234  1.0000
##   c22 - c24 -47.2330 1.173 34 -40.278  <.0001
##   c22 - c29    1.0143 0.957 34   1.059  0.9979
##   c22 - c30    1.2368 0.927 34   1.334  0.9834
##   c22 - c32    0.9263 0.957 34   0.967  0.9992
##   c22 - c4     0.0000 1.173 34   0.000  1.0000
##   c22 - c6     1.4710 1.173 34   1.254  0.9902
##   c22 - c9     0.6420 1.173 34   0.547  1.0000
##   c24 - c29   48.2473 0.957 34  50.390  <.0001
##   c24 - c30   48.4697 0.927 34  52.282  <.0001
##   c24 - c32   48.1593 0.957 34  50.298  <.0001
##   c24 - c4    47.2330 1.173 34  40.278  <.0001
##   c24 - c6    48.7040 1.173 34  41.532  <.0001
##   c24 - c9    47.8750 1.173 34  40.826  <.0001
##   c29 - c30    0.2224 0.633 34   0.351  1.0000
##   c29 - c32   -0.0880 0.677 34  -0.130  1.0000
##   c29 - c4    -1.0143 0.957 34  -1.059  0.9979
##   c29 - c6     0.4567 0.957 34   0.477  1.0000
##   c29 - c9    -0.3723 0.957 34  -0.389  1.0000
##   c30 - c32   -0.3104 0.633 34  -0.490  1.0000
##   c30 - c4    -1.2368 0.927 34  -1.334  0.9834
##   c30 - c6     0.2343 0.927 34   0.253  1.0000
##   c30 - c9    -0.5948 0.927 34  -0.642  1.0000
##   c32 - c4    -0.9263 0.957 34  -0.967  0.9992
##   c32 - c6     0.5447 0.957 34   0.569  1.0000
##   c32 - c9    -0.2843 0.957 34  -0.297  1.0000
##   c4 - c6      1.4710 1.173 34   1.254  0.9902
##   c4 - c9      0.6420 1.173 34   0.547  1.0000
##   c6 - c9     -0.8290 1.173 34  -0.707  1.0000
##
## P value adjustment: tukey method for comparing a family of 14 estimates

glm_BB_H9_0 <- glm(BB_H9 ~ 1, data = BB)
glm_BB_H9_1 <- glm(BB_H9 ~ BB_Blocs, data = BB)
```

```
glm_BB_H9_2 <- glm(BB_H9 ~ BB_Period, data = BB)
glm_BB_H9_3 <- glm(BB_H9 ~ BB_layer, data = BB)
Cand.models <- list()
Cand.models[[1]] <- glm_BB_H9_0
Cand.models[[2]] <- glm_BB_H9_1
Cand.models[[3]] <- glm_BB_H9_2
Cand.models[[4]] <- glm_BB_H9_3
Modnames <- lapply(Cand.models, "formula")
aictab(cand.set = Cand.models, modnames = paste0(Modnames), sort = TRUE)

##
## Model selection based on AICc:
##
##                      K   AICc Delta_AICc AICcWt Cum.Wt    LL
## BB_H9 ~ BB_Blocs    5 -30.41       0.00   0.39   0.39 20.92
## BB_H9 ~ BB_Period   4 -30.12       0.29   0.33   0.72 19.52
## BB_H9 ~ 1           2 -29.78       0.63   0.28   1.00 17.02
## BB_H9 ~ BB_layer   15 -17.74      12.67   0.00   1.00 31.37

marginal <- emmeans(glm_BB_H9_1, ~BB_Blocs)
pairs(marginal)

##  contrast estimate     SE df t.ratio p.value
##  A - B      0.1031 0.0635 44   1.622  0.3768
##  A - F     -0.1021 0.0982 44  -1.040  0.7273
##  A - G      0.1117 0.0751 44   1.487  0.4537
##  B - F     -0.2052 0.0880 44  -2.331  0.1064
##  B - G      0.0086 0.0612 44   0.141  0.9990
##  F - G      0.2138 0.0967 44   2.211  0.1361
##
## P value adjustment: tukey method for comparing a family of 4 estimates

glm_BB_H36_0 <- glm(BB_H36 ~ 1, data = BB)
glm_BB_H36_1 <- glm(BB_H36 ~ BB_Blocs, data = BB)
glm_BB_H36_2 <- glm(BB_H36 ~ BB_Period, data = BB)
glm_BB_H36_3 <- glm(BB_H36 ~ BB_layer, data = BB)
Cand.models <- list()
Cand.models[[1]] <- glm_BB_H36_0
Cand.models[[2]] <- glm_BB_H36_1
Cand.models[[3]] <- glm_BB_H36_2
Cand.models[[4]] <- glm_BB_H36_3
Modnames <- lapply(Cand.models, "formula")
aictab(cand.set = Cand.models, modnames = paste0(Modnames), sort = TRUE)

##
## Model selection based on AICc:
##
##                      K   AICc Delta_AICc AICcWt Cum.Wt    LL
## BB_H36 ~ BB_Period   4 -32.15       0.00   0.44   0.44 20.54
## BB_H36 ~ 1           2 -31.28       0.88   0.28   0.72 17.77
```

```
## BB_H36 ~ BB_Blocs   5 -31.00       1.15   0.25   0.97 21.21
## BB_H36 ~ BB_layer  15 -26.95       5.20   0.03   1.00 35.98
```

```
marginal <- emmeans(glm_BB_H36_2, ~BB_Period)
pairs(marginal)
```

```
##  contrast      estimate     SE df t.ratio p.value
##  AnteQ - PostQ  -0.0152 0.0586 45  -0.259  0.9638
##  AnteQ - Quina  -0.2102 0.0964 45  -2.181  0.0855
##  PostQ - Quina  -0.1950 0.0861 45  -2.264  0.0714
##
## P value adjustment: tukey method for comparing a family of 3 estimates
```

```
glm_BB_H81_0 <- glm(BB_H81 ~ 1, data = BB)
glm_BB_H81_1 <- glm(BB_H81 ~ BB_Blocs, data = BB)
glm_BB_H81_2 <- glm(BB_H81 ~ BB_Period, data = BB)
glm_BB_H81_3 <- glm(BB_H81 ~ BB_layer, data = BB)
Cand.models <- list()
Cand.models[[1]] <- glm_BB_H81_0
Cand.models[[2]] <- glm_BB_H81_1
Cand.models[[3]] <- glm_BB_H81_2
Cand.models[[4]] <- glm_BB_H81_3
Modnames <- lapply(Cand.models, "formula")
aictab(cand.set = Cand.models, modnames = paste0(Modnames), sort = TRUE)
```

```
##
## Model selection based on AICc:
##
##                      K  AICc Delta_AICc AICcWt Cum.Wt    LL
## BB_H81 ~ BB_Period  4 -5.82       0.00   0.59   0.59  7.37
## BB_H81 ~ BB_Blocs   5 -4.16       1.66   0.26   0.84  7.79
## BB_H81 ~ BB_layer  15 -2.31       3.50   0.10   0.95 23.66
## BB_H81 ~ 1          2 -1.04       4.78   0.05   1.00  2.65
```

```
marginal <- emmeans(glm_BB_H81_2, ~BB_Period)
pairs(marginal)
```

```
##  contrast      estimate     SE df t.ratio p.value
##  AnteQ - PostQ  -0.0426 0.0771 45  -0.553  0.8456
##  AnteQ - Quina  -0.3775 0.1268 45  -2.977  0.0127
##  PostQ - Quina  -0.3349 0.1133 45  -2.956  0.0135
##
## P value adjustment: tukey method for comparing a family of 3 estimates
```

Inter-specific differences between contemporaneous ungulates (per block)

Data visualization

```
xyplot(Asfc ~ specie | Blocs, data = db_FOSSILS, pch = 20, cex = 2)
```

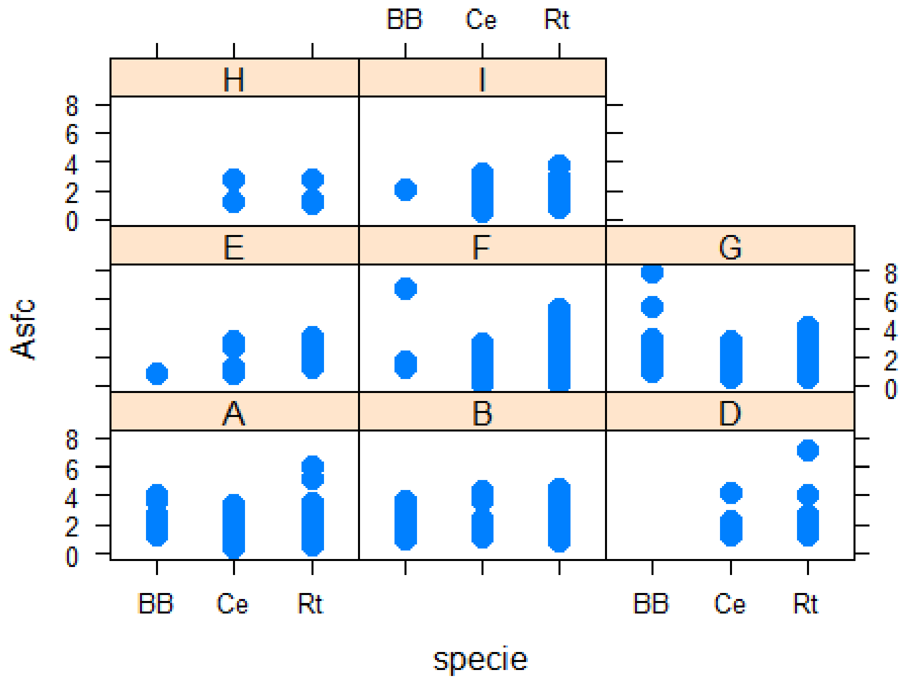


```
xyplot(epLsar ~ specie | Blocs, data = db_FOSSILS, pch = 20, cex = 2)
```

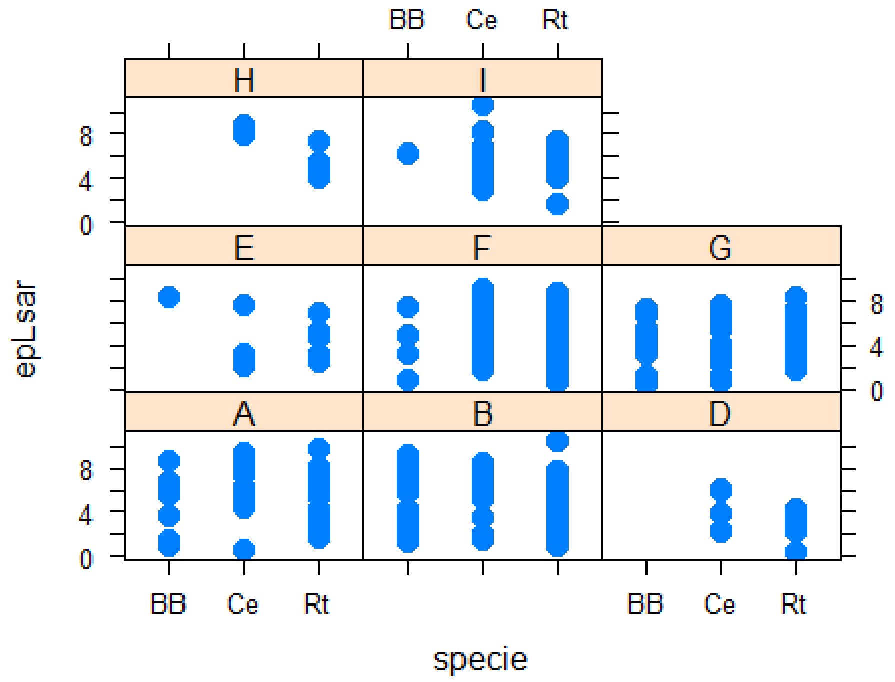


```
xyplot(Smc ~ specie | Blocs, data = db_FOSSILS, pch = 20, cex = 2)
```

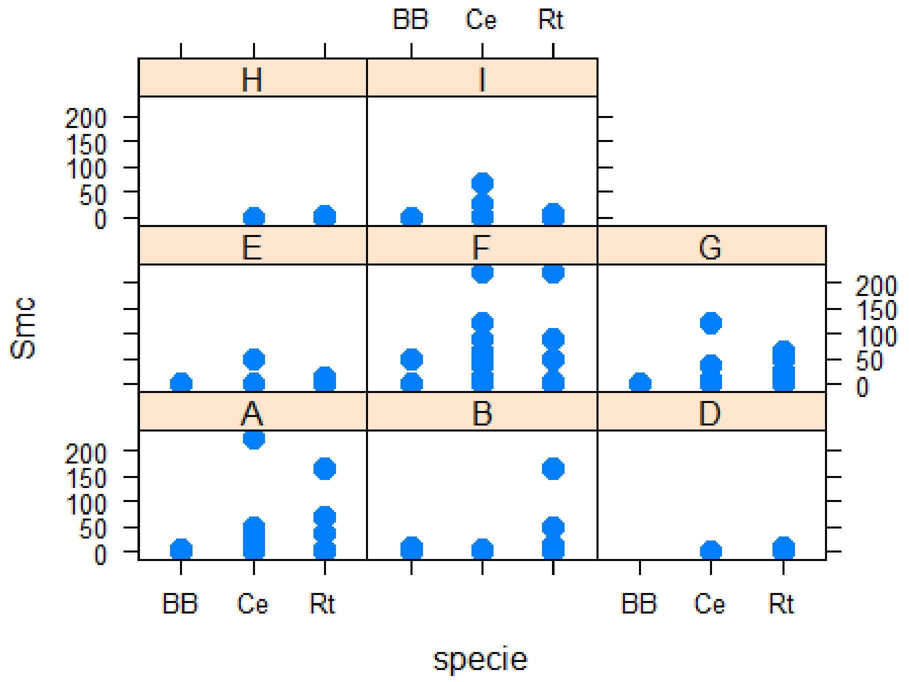


```
xyplot(HAsfc9 ~ specie | Blocs, data = db_FOSSILS, pch = 20, cex = 2)
```

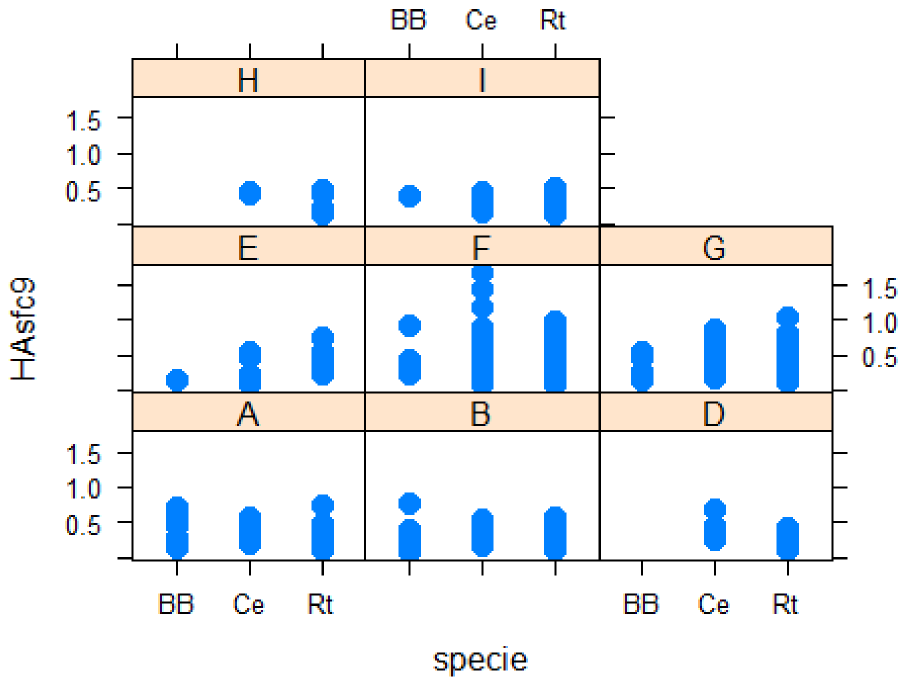


```
xyplot(HAsfc81 ~ specie | Blocs, data = db_FOSSILS, pch = 20, cex = 2)
```

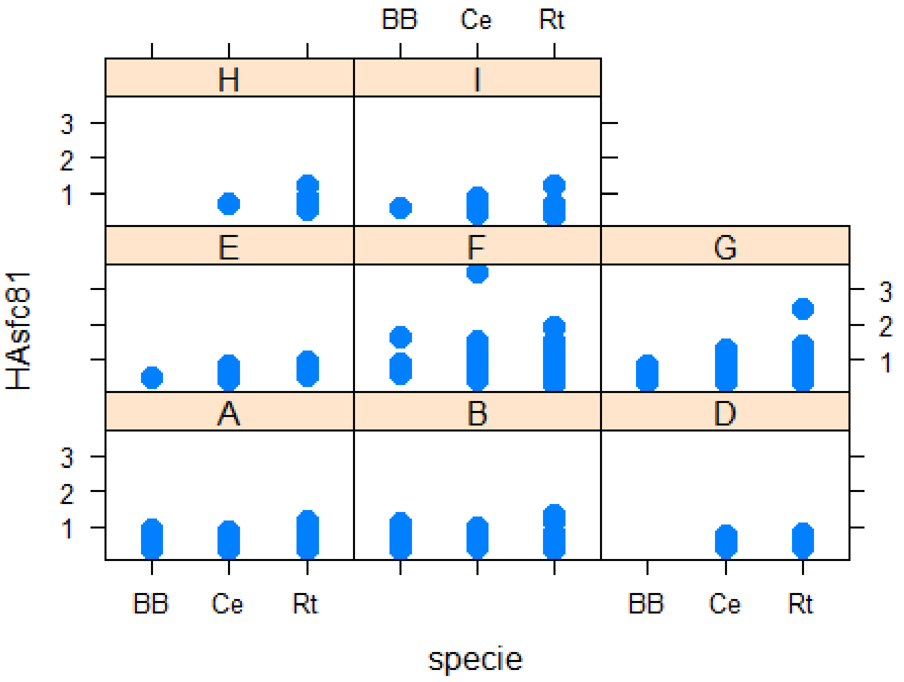


```r
xyplot(HAsfc36 ~ specie | Blocs, data = db_FOSSILS, pch = 20, cex = 2)
```

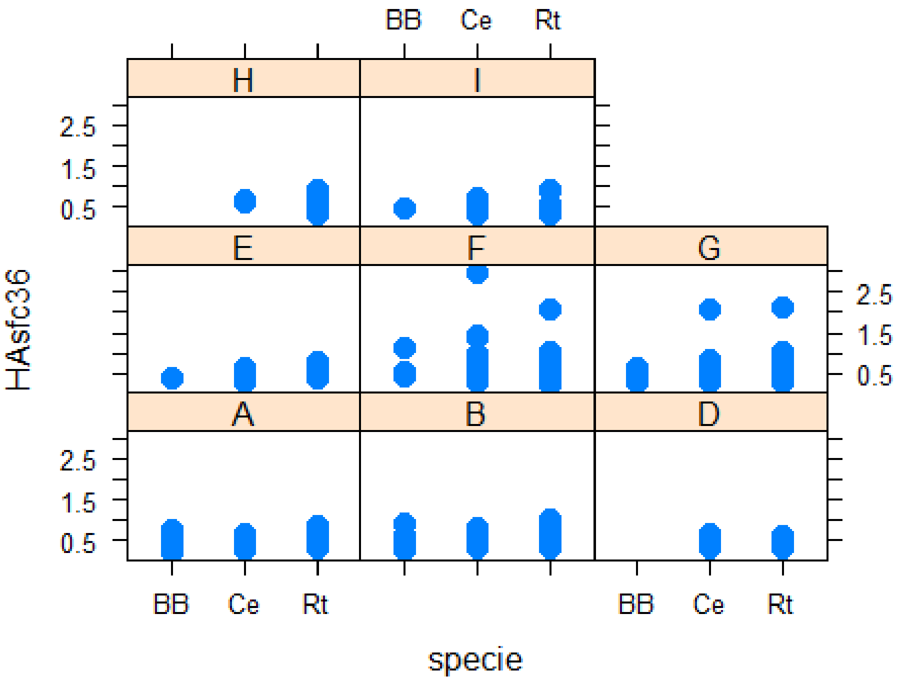

Block A

```
A_Species <- A %>%
  dplyr::select(c(2)) %>%
  unlist(c(1))

A_Asfc <- A %>%
  dplyr::select(c(7)) %>%
  unlist(c(1))
A_epLsar <- A %>%
  dplyr::select(c(8)) %>%
  unlist(c(1))
A_Smc <- A %>%
  dplyr::select(c(9)) %>%
  unlist(c(1))
A_H9 <- A %>%
  dplyr::select(c(10)) %>%
  unlist(c(1))
A_H36 <- A %>%
  dplyr::select(c(12)) %>%
  unlist(c(1))
A_H81 <- A %>%
  dplyr::select(c(11)) %>%
  unlist(c(1))
```

Checking data distribution and outliers:

```
x <- A[order(A_Asfc), ]
x$specie <- factor(x$specie)
dotchart(x$Asfc, cex = 1, pch = 16, groups = x$specie, xlab = "A_Asfc per spe
cie")
```

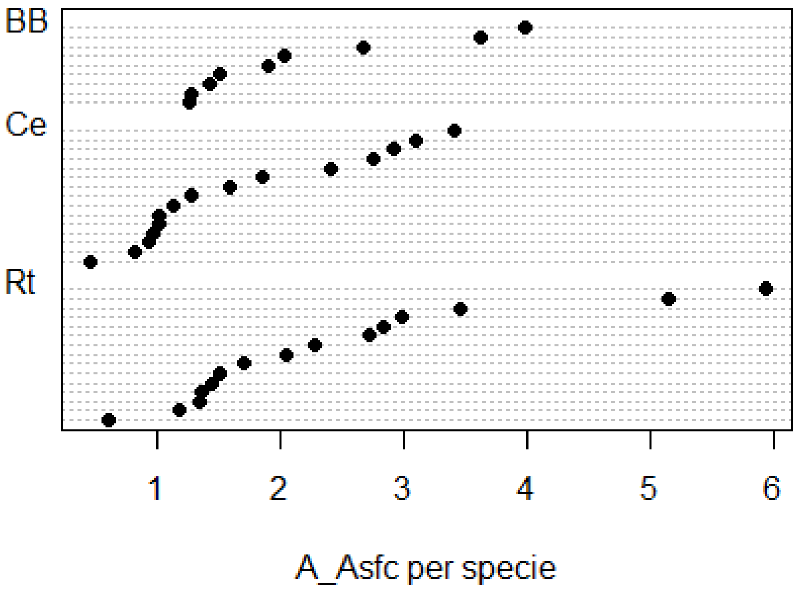


```
x <- A[order(A_epLsar), ]
x$specie <- factor(x$specie)
dotchart(x$ epLsar, cex = 1, pch = 16, groups = x$specie, xlab = "A_epLsar pe
r specie")
```

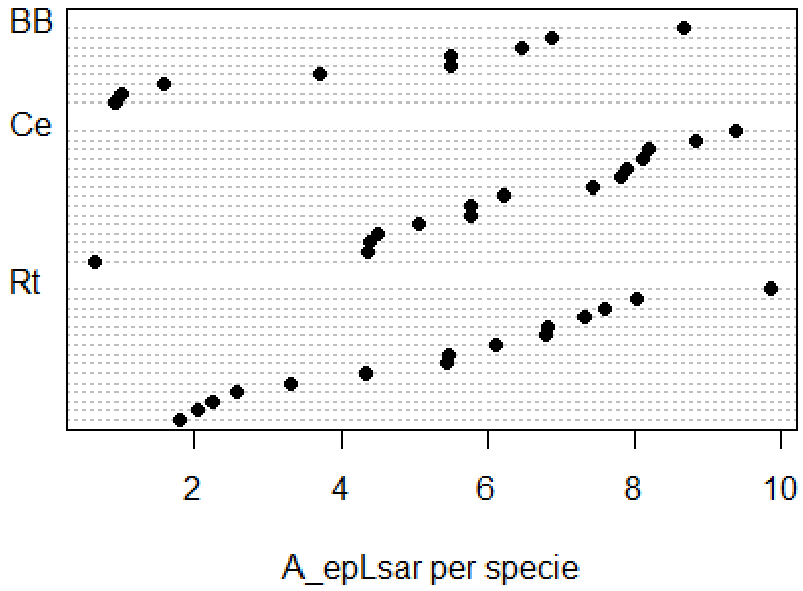


```
x <- A[order(A_Smc), ]
x$specie <- factor(x$specie)
dotchart(x$Smc, cex = 1, pch = 16, groups = x$specie, xlab = "A_Smc per speci
e")
```

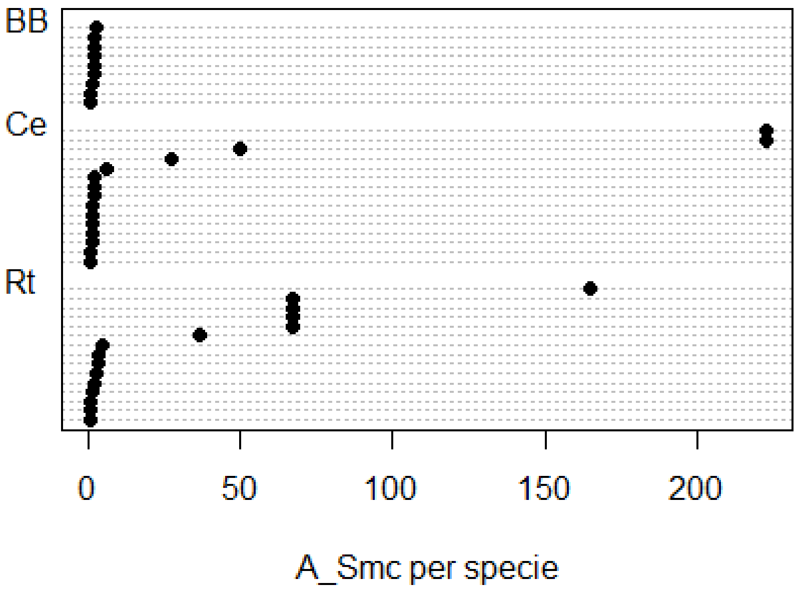


```
x <- A[order(A_H9), ]
x$specie <- factor(x$specie)
dotchart(x$HAsfc9, cex = 1, pch = 16, groups = x$specie, xlab = "A_H9 per spe
cie")
```

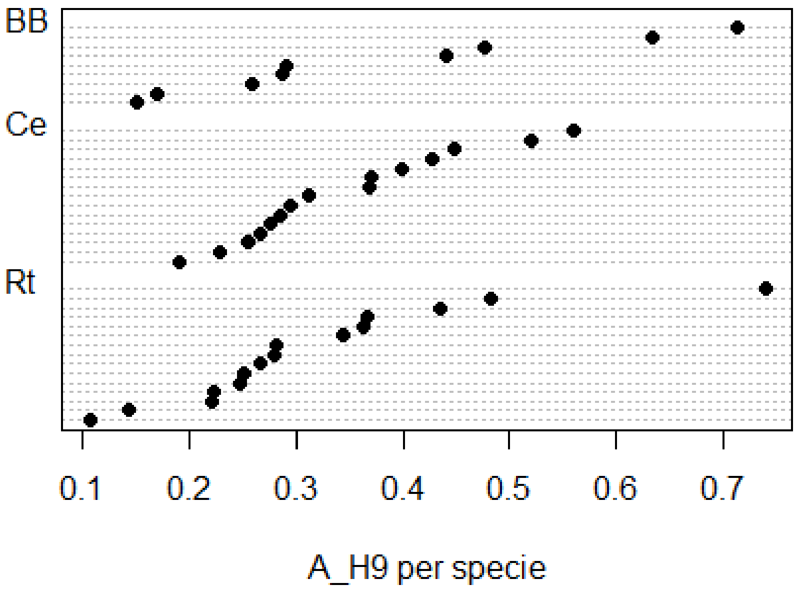


```
x <- A[order(A_H36), ]
x$specie <- factor(x$specie)
dotchart(x$HAsfc36, cex = 1, pch = 16, groups = x$specie, xlab = "A_H36 per s
pecie")
```

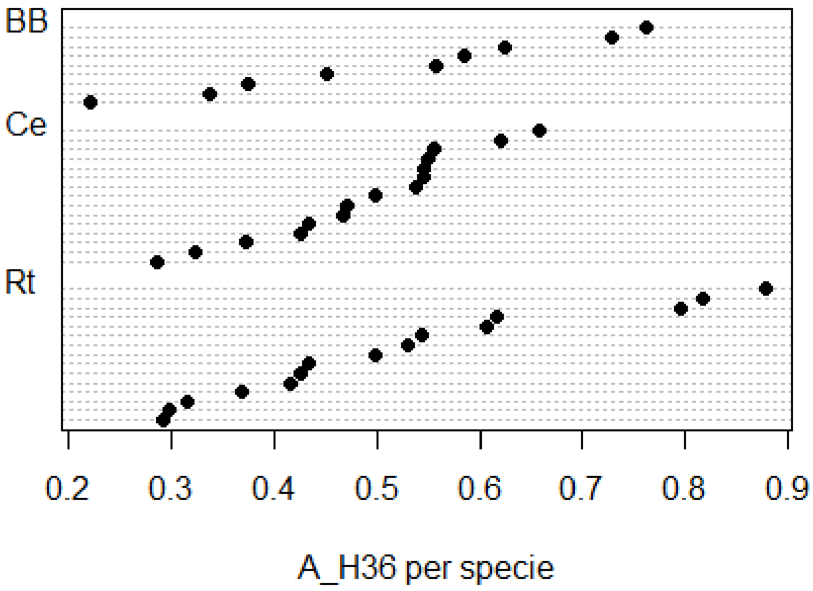


```
x <- A[order(A_H81), ]
x$specie <- factor(x$specie)
dotchart(x$HAsfc81, cex = 1, pch = 16, groups = x$specie, xlab = "A_H81 per s
pecie")
```

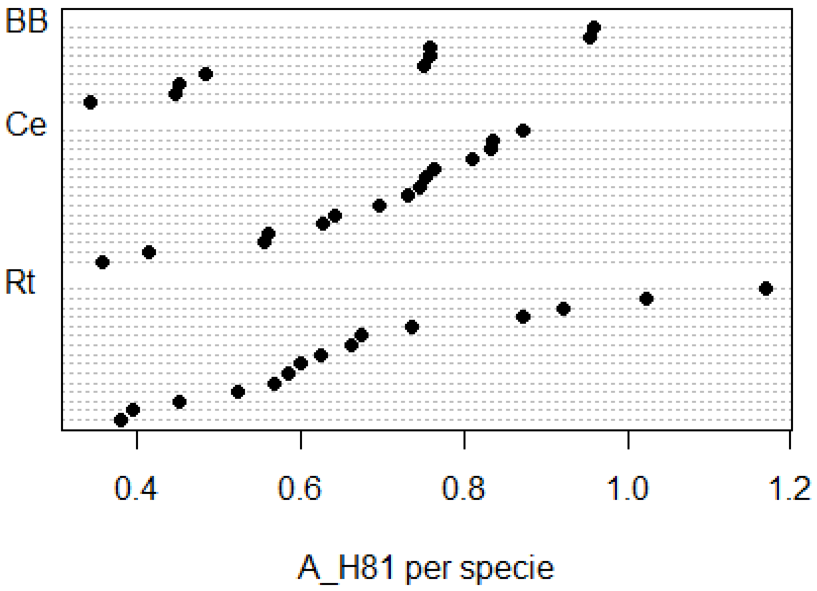

Graphical evaluation of the tests’ applicability:

Normality and homoscedasticity of the variables. #### Normality

```
ggplot(A) +
  geom_freqpoly(aes(x = Asfc), bins = 7) +
  labs(
    x = "Value Asfc",
    y = "Frequency"
  )
```

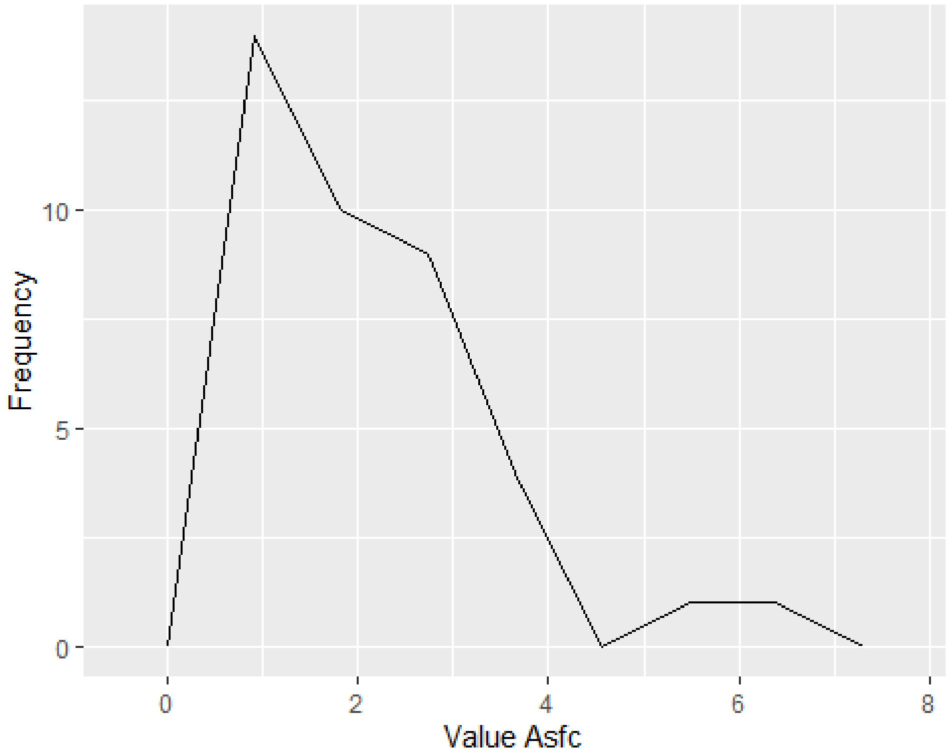


```
ggplot(A) +
  geom_freqpoly(aes(x = epLsar), bins = 7) +
  labs(
    x = "Value epLsar",
    y = "Frequency"
  )
```

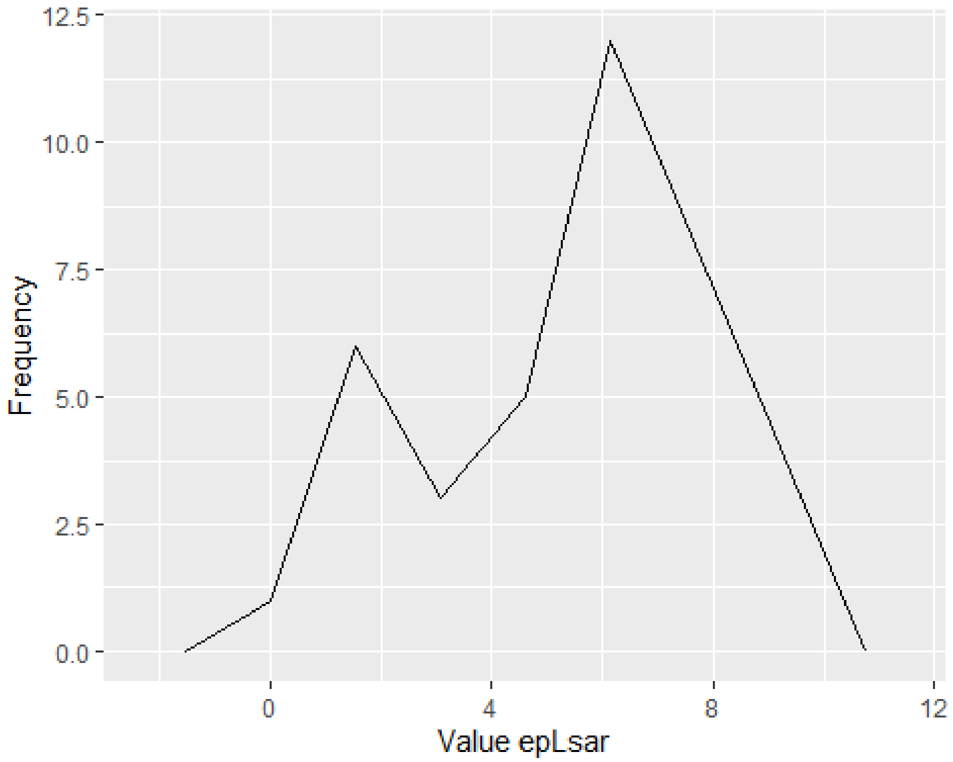


```
ggplot(A) +
  geom_freqpoly(aes(x = Smc), bins = 7) +
  labs(
    x = "Value Smc",
    y = "Frequency"
  )
```

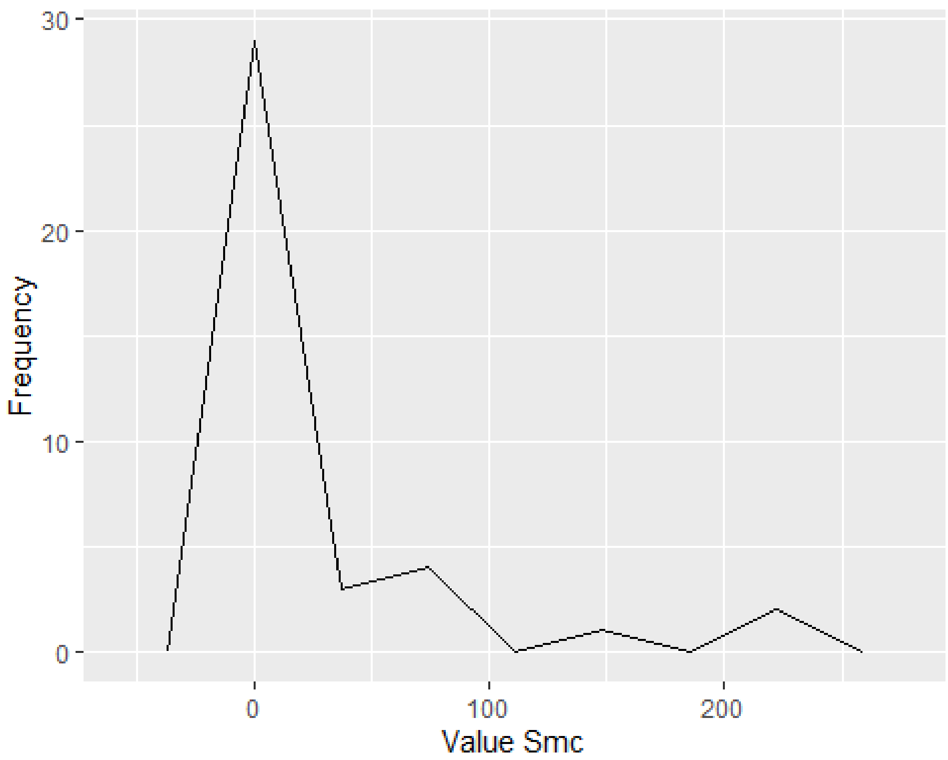


```
ggplot(A) +
  geom_freqpoly(aes(x = HAsfc9), bins = 7) +
  labs(
    x = "Value H9",
    y = "Frequency"
  )
```

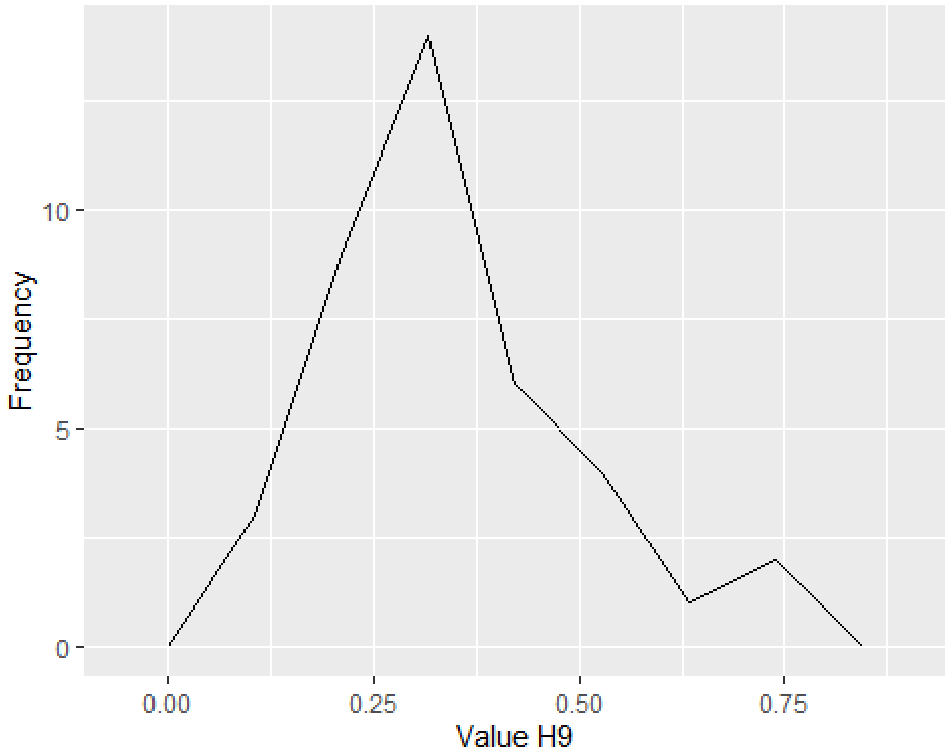


```
ggplot(A) +
  geom_freqpoly(aes(x = HAsfc36), bins = 7) +
  labs(
    x = "Value H36",
    y = "Frequency"
  )
```

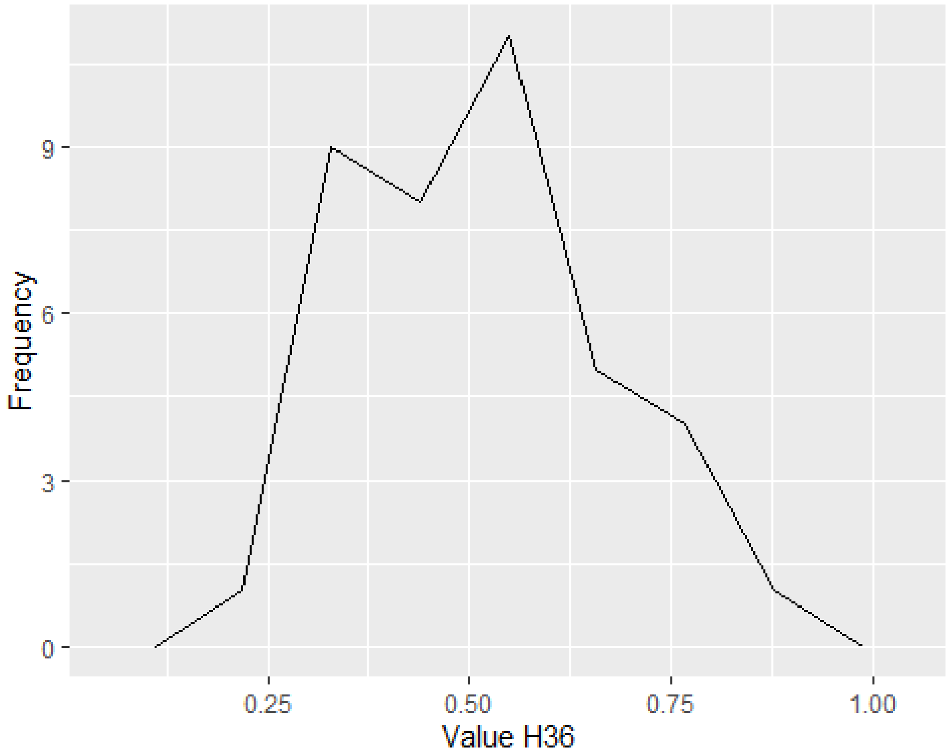


```
ggplot(A) +
  geom_freqpoly(aes(x = HAsfc81), bins = 7) +
  labs(
    x = "Value H81",
    y = "Frequency"
  )
```

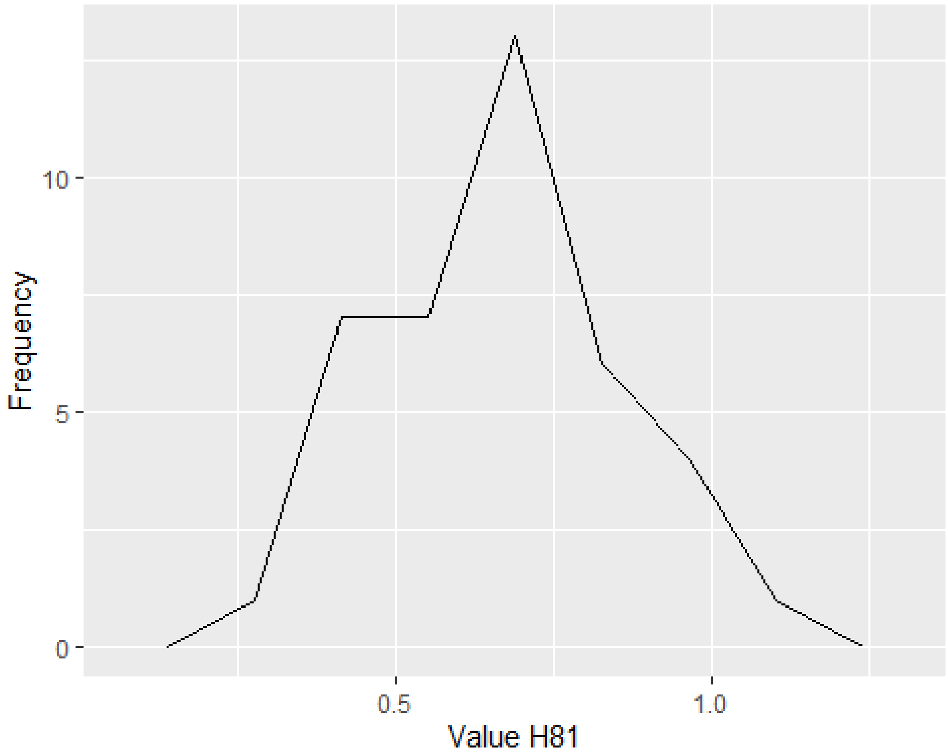

Homoscedasticity: Brown & Forsythe test (and data transformation whenever needed)

```
bf.test(A_Asfc ~ A_Species, data = A)

##
##   Brown-Forsythe Test (alpha = 0.05)
## -------------------------------------------------------------
##   data : A_Asfc and A_Species
##
##   statistic  : 1.485543
##   num df     : 2
##   denom df   : 31.59355
##   p.value    : 0.2417662
##
##   Result     : Difference is not statistically significant.
## -------------------------------------------------------------

ggplot(A) +
  geom_boxplot(aes(x = A_Species, y = A_Asfc)) +
  labs(
    x = "Species",
    y = "Asfc"
  )
```

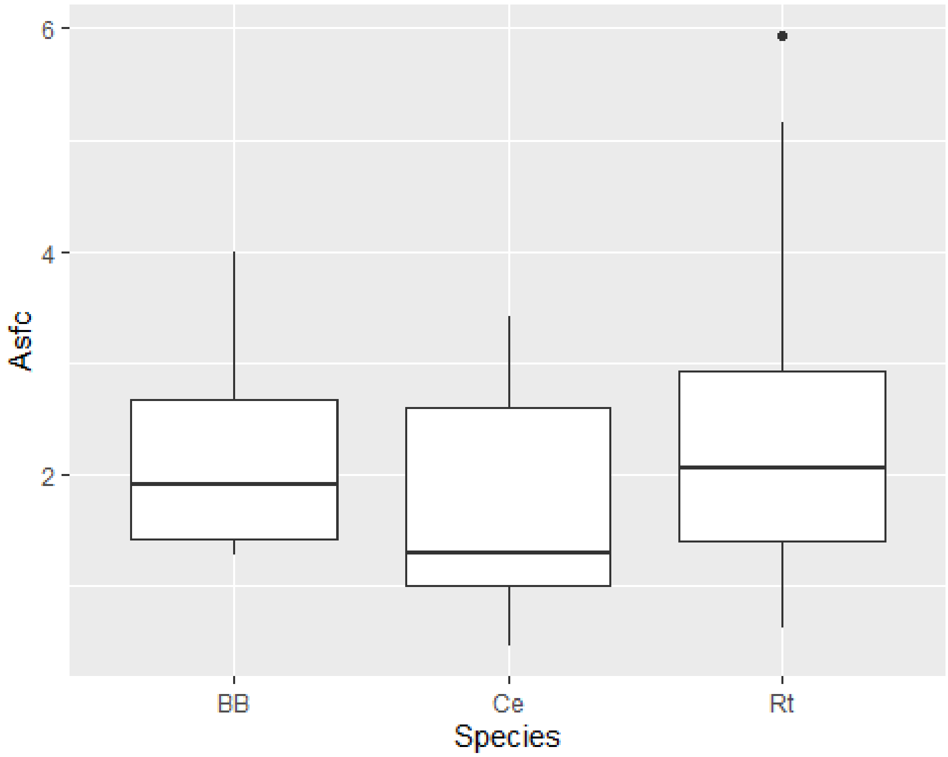


```
bf.test(A_epLsar ~ A_Species, data = A)

##
##   Brown-Forsythe Test (alpha = 0.05)
## -------------------------------------------------------------
##   data : A_epLsar and A_Species
##
##   statistic  : 1.490972
##   num df     : 2
##   denom df   : 27.04229
##   p.value    : 0.2430834
##
##   Result     : Difference is not statistically significant.
## -------------------------------------------------------------

ggplot(A) +
  geom_boxplot(aes(x = A_Species, y = A_epLsar)) +
  labs(
    x = "Species",
    y = "epLsar"
  )
```

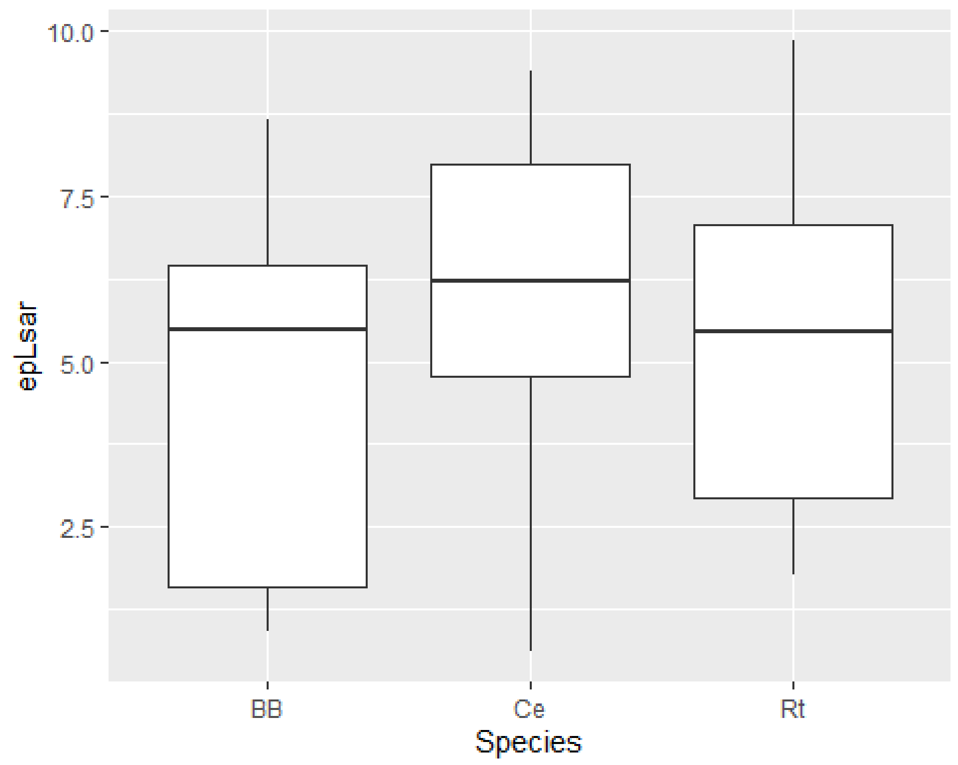


```
bf.test(A_Smc ~ A_Species, data = A)

##
##   Brown-Forsythe Test (alpha = 0.05)
## -------------------------------------------------------------
##   data : A_Smc and A_Species
##
##   statistic  : 1.506006
##   num df     : 2
##   denom df   : 23.1255
##   p.value    : 0.2427575
##
##   Result     : Difference is not statistically significant.
## -------------------------------------------------------------

ggplot(A) +
  geom_boxplot(aes(x = A_Species, y = A_Smc)) +
  labs(
    x = "Species",
    y = "Smc"
  )
```

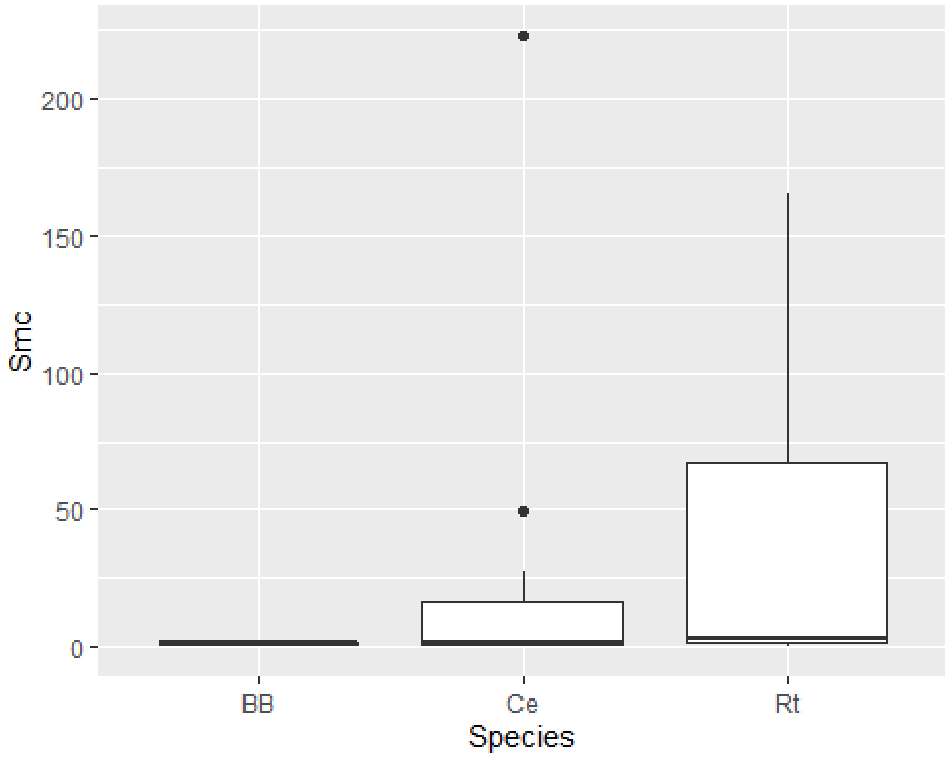


```
bf.test(A_H9 ~ A_Species, data = A)

##
##   Brown-Forsythe Test (alpha = 0.05)
## -------------------------------------------------------------
##   data : A_H9 and A_Species
##
##   statistic  : 0.4395635
##   num df     : 2
##   denom df   : 20.2048
##   p.value    : 0.6503339
##
##   Result     : Difference is not statistically significant.
## -------------------------------------------------------------

ggplot(A) +
  geom_boxplot(aes(x = A_Species, y = A_H9)) +
  labs(
    x = "Species",
    y = "H9"
  )
```

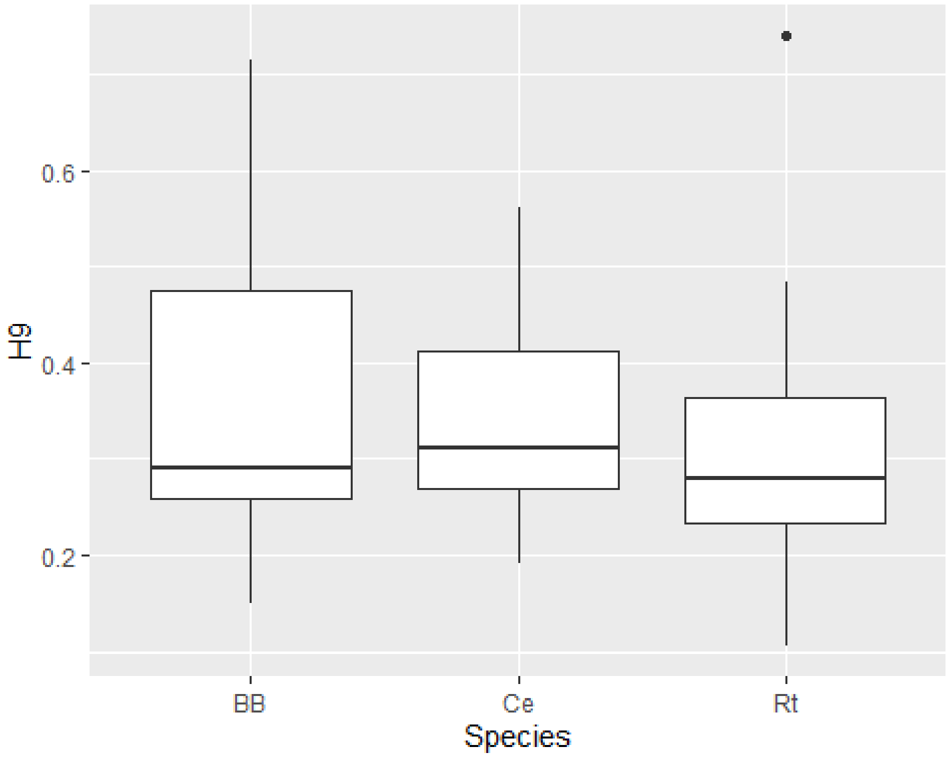


```
bf.test(A_H36 ~ A_Species, data = A)

##
##   Brown-Forsythe Test (alpha = 0.05)
## -------------------------------------------------------------
##   data : A_H36 and A_Species
##
##   statistic  : 0.2004466
##   num df     : 2
##   denom df   : 24.59662
##   p.value    : 0.8196887
##
##   Result     : Difference is not statistically significant.
## -------------------------------------------------------------

ggplot(A) +
  geom_boxplot(aes(x = A_Species, y = A_H36)) +
  labs(
    x = "Species",
    y = "H36"
  )
```

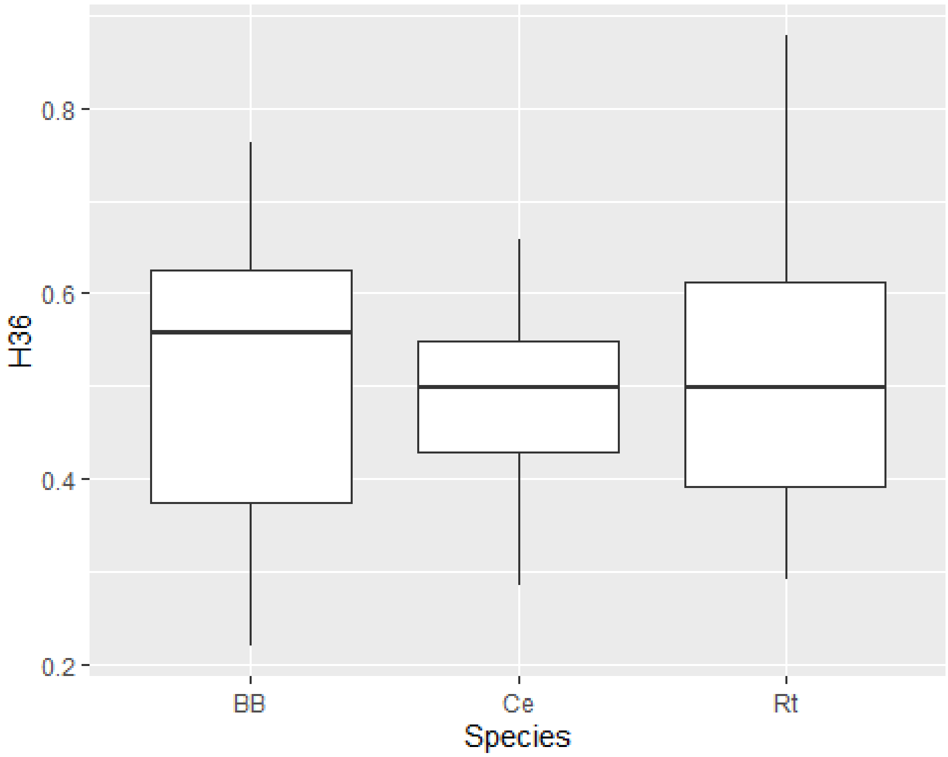


```
bf.test(A_H81 ~ A_Species, data = A)

##
##   Brown-Forsythe Test (alpha = 0.05)
## -------------------------------------------------------------
##   data : A_H81 and A_Species
##
##   statistic  : 0.04349263
##   num df     : 2
##   denom df   : 25.79596
##   p.value    : 0.9575097
##
##   Result     : Difference is not statistically significant.
## -------------------------------------------------------------

ggplot(A) +
  geom_boxplot(aes(x = A_Species, y = A_H81)) +
  labs(
    x = "Species",
    y = "H81"
  )
```

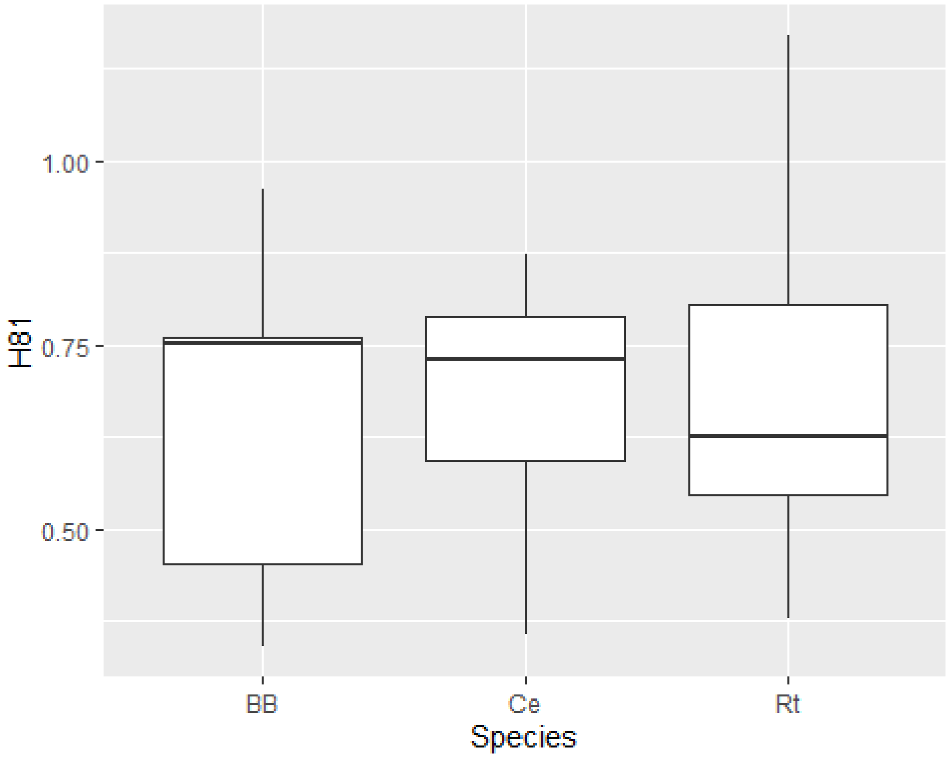

Glm: Impact of blocks over each DMTA parameter

```
glm_A_Asfc0 <- glm(A_Asfc ~ 1, data = A)
glm_A_Asfc1 <- glm(A_Asfc ~ A_Species, data = A)
Cand.models <- list()
Cand.models[[1]] <- glm_A_Asfc0
Cand.models[[2]] <- glm_A_Asfc1
Modnames <- lapply(Cand.models, "formula")
aictab(cand.set = Cand.models, modnames = paste0(Modnames), sort = TRUE)

##
## Model selection based on AICc:
##
##                      K   AICc Delta_AICc AICcWt Cum.Wt     LL
## A_Asfc ~ 1           2 129.30       0.00   0.72   0.72 -62.49
## A_Asfc ~ A_Species 4 131.23       1.92   0.28   1.00 -61.03

glm_A_epLsar0 <- glm(A_epLsar ~ 1, data = A)
glm_A_epLsar1 <- glm(A_epLsar ~ A_Species, data = A)
Cand.models <- list()
Cand.models[[1]] <- glm_A_epLsar0
Cand.models[[2]] <- glm_A_epLsar1
Modnames <- lapply(Cand.models, "formula")
aictab(cand.set = Cand.models, modnames = paste0(Modnames), sort = TRUE)

##
## Model selection based on AICc:
##
```

```
##                        K   AICc Delta_AICc AICcWt Cum.Wt     LL
## A_epLsar ~ 1          2 186.45       0.00   0.69   0.69 -91.06
## A_epLsar ~ A_Species 4 188.02       1.58   0.31   1.00 -89.42
```

```
glm_A_Smc0 <- glm(A_Smc ~ 1, data = A)
glm_A_Smc1 <- glm(A_Smc ~ A_Species, data = A)
Cand.models <- list()
Cand.models[[1]] <- glm_A_Smc0
Cand.models[[2]] <- glm_A_Smc1
Modnames <- lapply(Cand.models, "formula")
aictab(cand.set = Cand.models, modnames = paste0(Modnames), sort = TRUE)
```

```
##
## Model selection based on AICc:
##
##                     K   AICc Delta_AICc AICcWt Cum.Wt      LL
## A_Smc ~ 1          2 428.55       0.00   0.76   0.76 -212.11
## A_Smc ~ A_Species 4 430.89       2.34   0.24   1.00 -210.86
```

```
glm_A_H9_0 <- glm(A_H9 ~ 1, data = A)
glm_A_H9_1 <- glm(A_H9 ~ A_Species, data = A)
Cand.models <- list()
Cand.models[[1]] <- glm_A_H9_0
Cand.models[[2]] <- glm_A_H9_1
Modnames <- lapply(Cand.models, "formula")
aictab(cand.set = Cand.models, modnames = paste0(Modnames), sort = TRUE)
```

```
##
## Model selection based on AICc:
##
##                    K   AICc Delta_AICc AICcWt Cum.Wt    LL
## A_H9 ~ 1          2 -34.84       0.00   0.87   0.87 19.59
## A_H9 ~ A_Species 4 -31.09       3.76   0.13   1.00 20.13
```

```
glm_A_H36_0 <- glm(A_H36 ~ 1, data = A)
glm_A_H36_1 <- glm(A_H36 ~ A_Species, data = A)
Cand.models <- list()
Cand.models[[1]] <- glm_A_H36_0
Cand.models[[2]] <- glm_A_H36_1
Modnames <- lapply(Cand.models, "formula")
aictab(cand.set = Cand.models, modnames = paste0(Modnames), sort = TRUE)
```

```
##
## Model selection based on AICc:
##
##                     K   AICc Delta_AICc AICcWt Cum.Wt    LL
## A_H36 ~ 1          2 -30.39       0.00    0.9    0.9 17.36
## A_H36 ~ A_Species 4 -26.01       4.38    0.1    1.0 17.59
```

```
glm_A_H81_0 <- glm(A_H81 ~ 1, data = A)
glm_A_H81_1 <- glm(A_H81 ~ A_Species, data = A)
Cand.models <- list()
```

```
Cand.models[[1]] <- glm_A_H81_0
Cand.models[[2]] <- glm_A_H81_1
Modnames <- lapply(Cand.models, "formula")
aictab(cand.set = Cand.models, modnames = paste0(Modnames), sort = TRUE)

##
## Model selection based on AICc:
##
##                     K   AICc Delta_AICc AICcWt Cum.Wt   LL
## A_H81 ~ 1           2 -12.20       0.00   0.91   0.91 8.27
## A_H81 ~ A_Species 4  -7.46       4.74   0.09   1.00 8.32
```

Block B

```
B_Species <- B %>%
  dplyr::select(c(2)) %>%
  unlist(c(1))

B_Asfc <- B %>%
  dplyr::select(c(7)) %>%
  unlist(c(1))
B_epLsar <- B %>%
  dplyr::select(c(8)) %>%
  unlist(c(1))
B_Smc <- B %>%
  dplyr::select(c(9)) %>%
  unlist(c(1))
B_H9 <- B %>%
  dplyr::select(c(10)) %>%
  unlist(c(1))
B_H36 <- B %>%
  dplyr::select(c(12)) %>%
  unlist(c(1))
B_H81 <- B %>%
  dplyr::select(c(11)) %>%
  unlist(c(1))
```

Checking data distribution and outliers:

```
x <- B[order(B_Asfc), ]
x$specie <- factor(x$specie)
dotchart(x$Asfc, cex = 1, pch = 16, groups = x$specie, xlab = "B_Asfc per spe
cie")
```

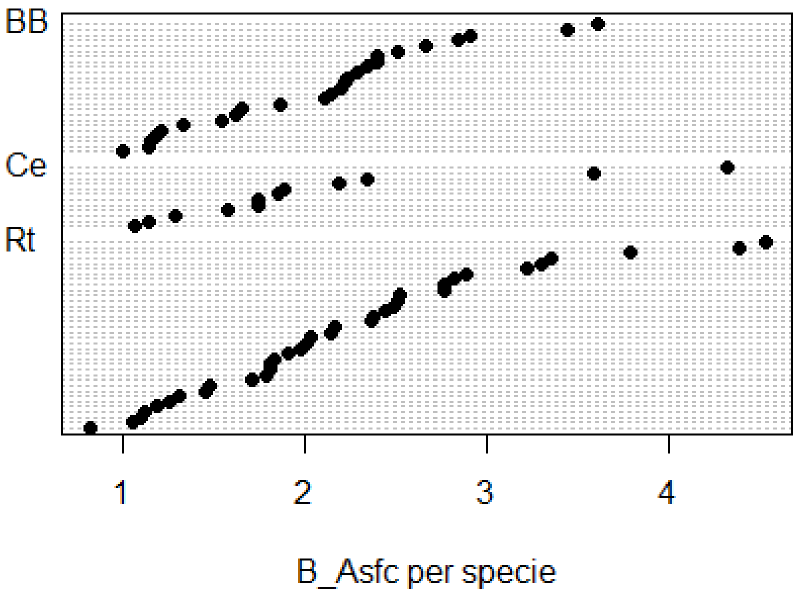


```
x <- B[order(B_epLsar), ]
x$specie <- factor(x$specie)
dotchart(x$epLsar, cex = 1, pch = 16, groups = x$specie, xlab = "B_epLsar per
specie")
```

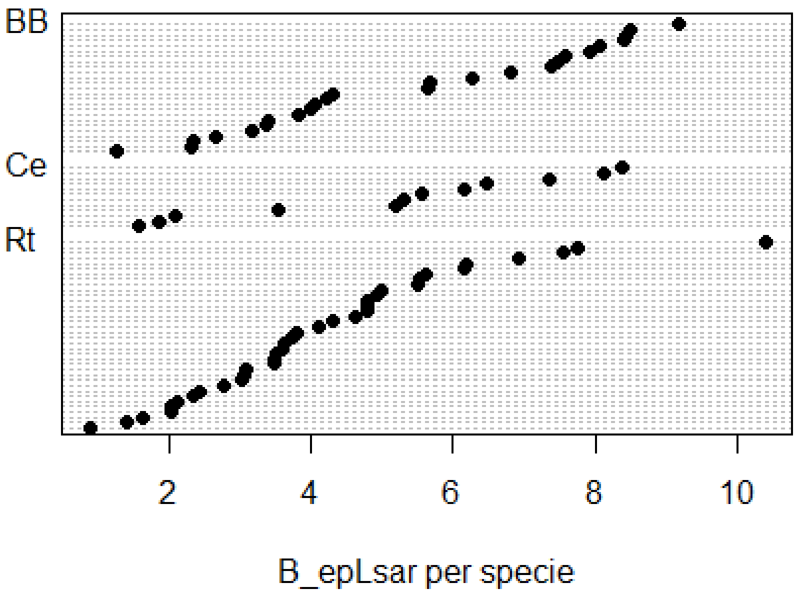


```
x <- B[order(B_Smc), ]
x$specie <- factor(x$specie)
dotchart(x$Smc, cex = 1, pch = 16, groups = x$specie, xlab = "B_Smc per speci
e")
```

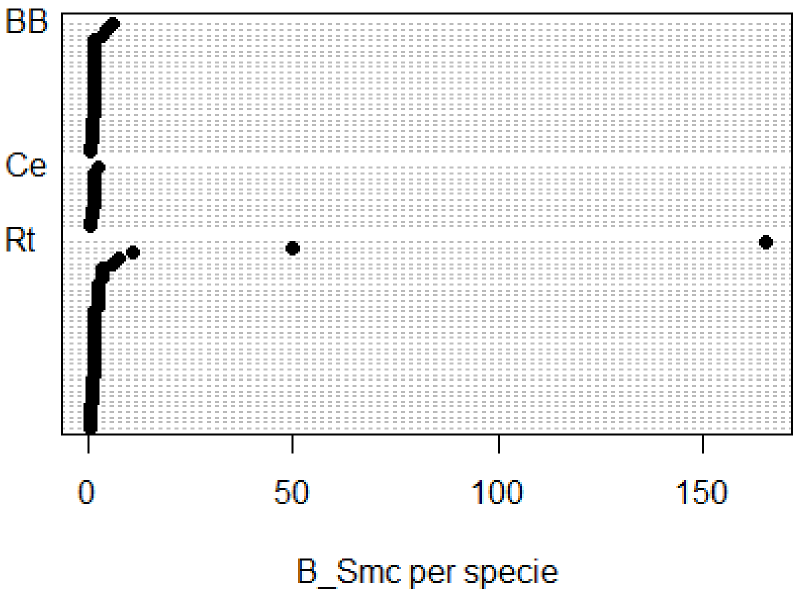


```
x <- B[order(B_H9), ]
x$specie <- factor(x$specie)
dotchart(x$HAsfc9, cex = 1, pch = 16, groups = x$specie, xlab = "B_H9 per spe
cie")
```

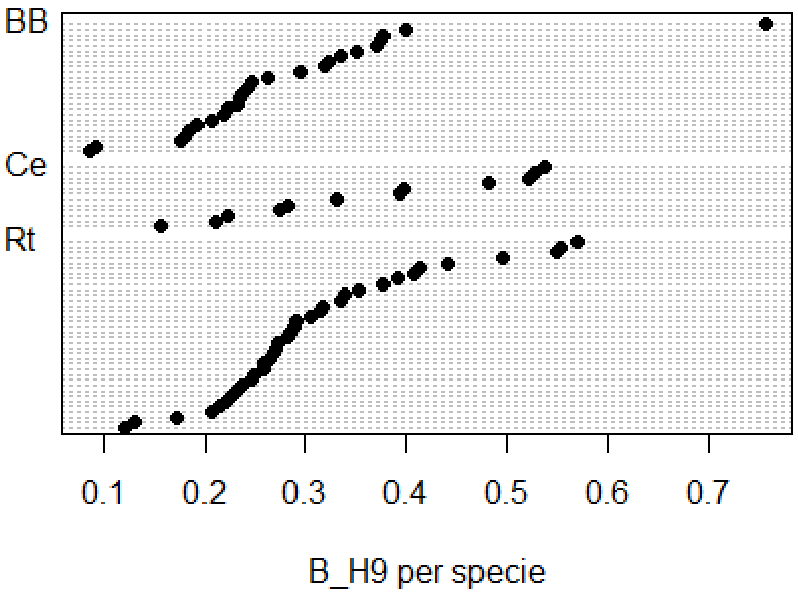


```
x <- B[order(B_H36), ]
x$specie <- factor(x$specie)
dotchart(x$HAsfc36, cex = 1, pch = 16, groups = x$specie, xlab = "B_H36 per s
pecie")
```

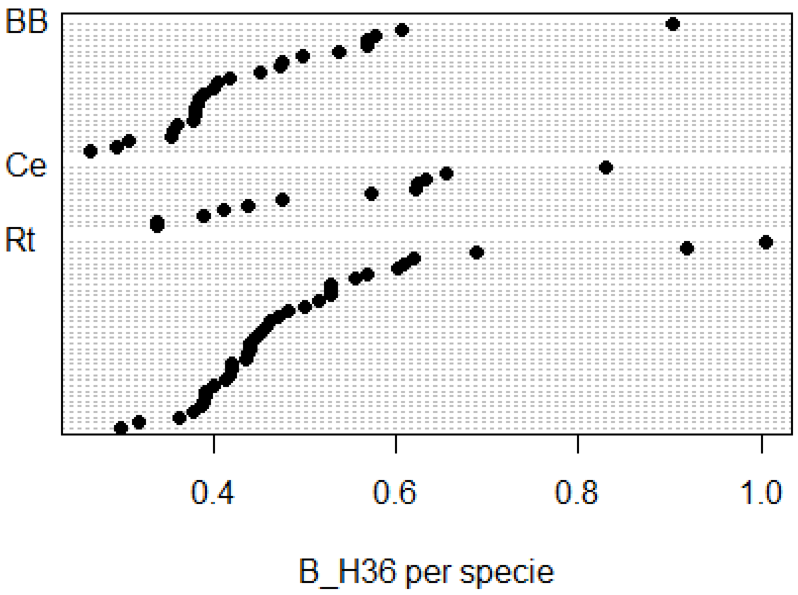


```
x <- B[order(B_H81), ]
x$specie <- factor(x$specie)
dotchart(x$HAsfc81, cex = 1, pch = 16, groups = x$specie, xlab = "B_H81 per s
pecie")
```

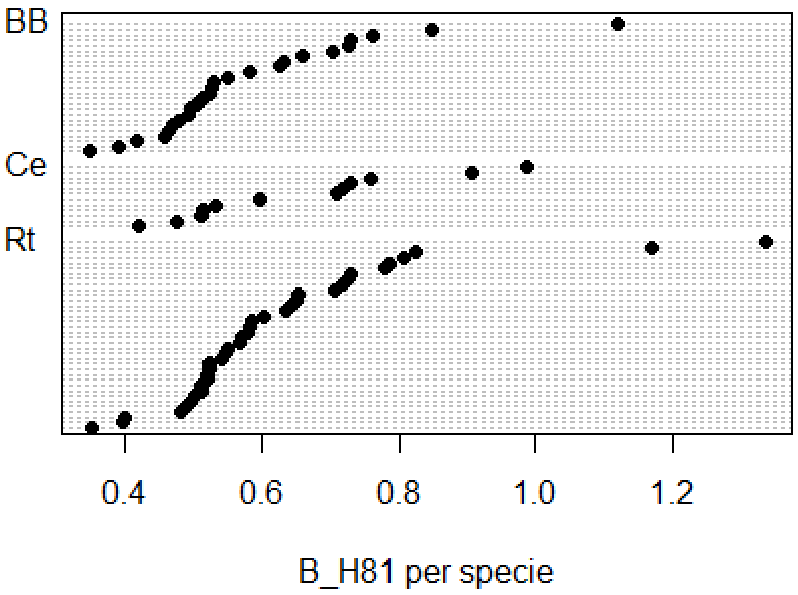

Graphical evaluation of the tests’ applicability:

Normality and homoscedasticity of the variables. #### Normality

```
ggplot(B) +
  geom_freqpoly(aes(x = Asfc), bins = 7) +
  labs(
    x = "Value Asfc",
    y = "Frequency"
  )
```

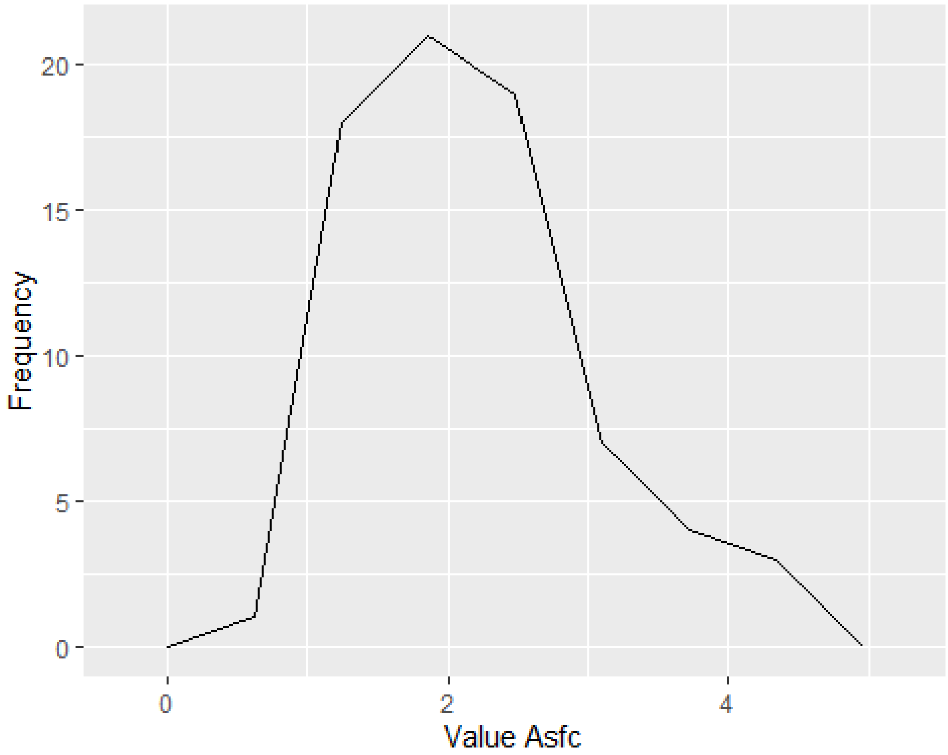


```
ggplot(B) +
  geom_freqpoly(aes(x = epLsar), bins = 7) +
  labs(
    x = "Value epLsar",
    y = "Frequency"
  )
```

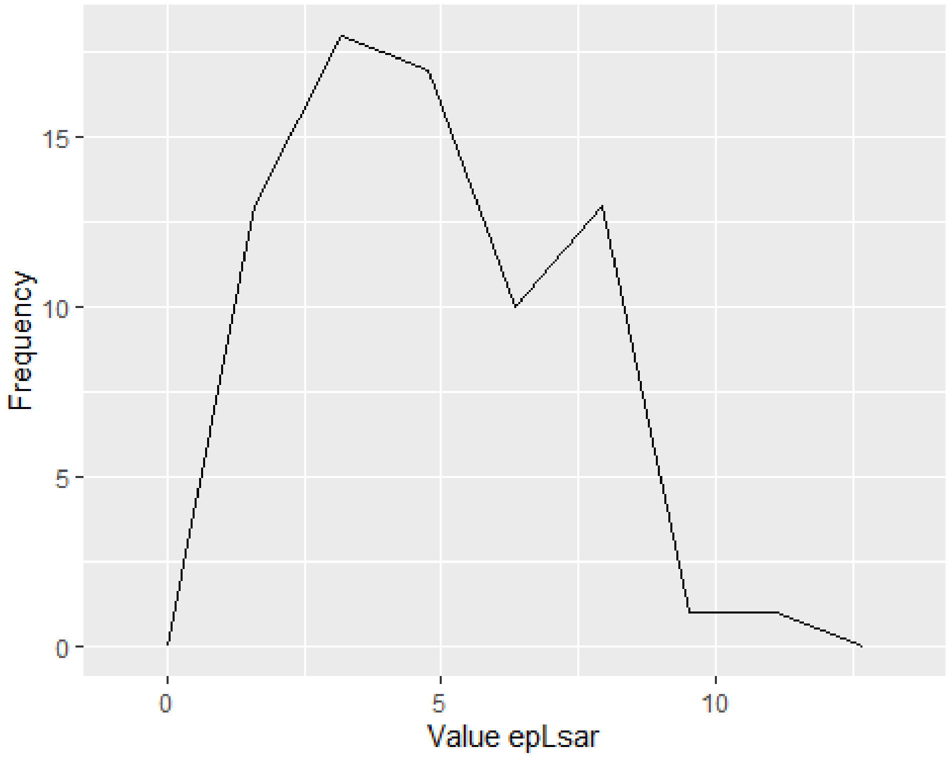


```
ggplot(B) +
  geom_freqpoly(aes(x = Smc), bins = 7) +
  labs(
    x = "Value Smc",
    y = "Frequency"
  )
```

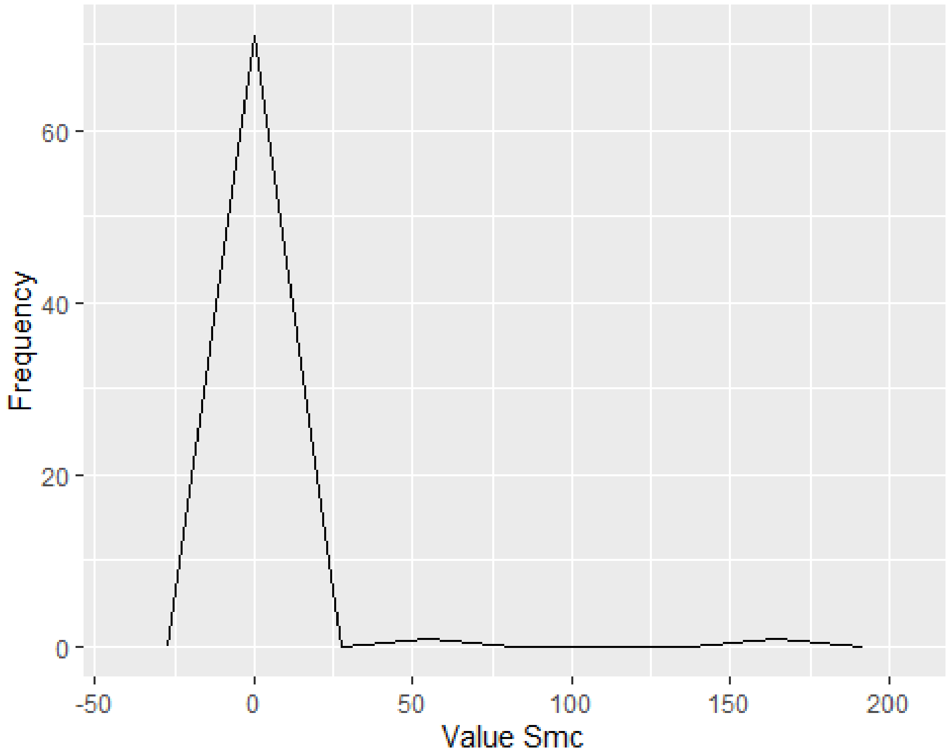


```r
ggplot(B) +
  geom_freqpoly(aes(x = HAsfc9), bins = 7) +
  labs(
    x = "Value H9",
    y = "Frequency"
  )
```

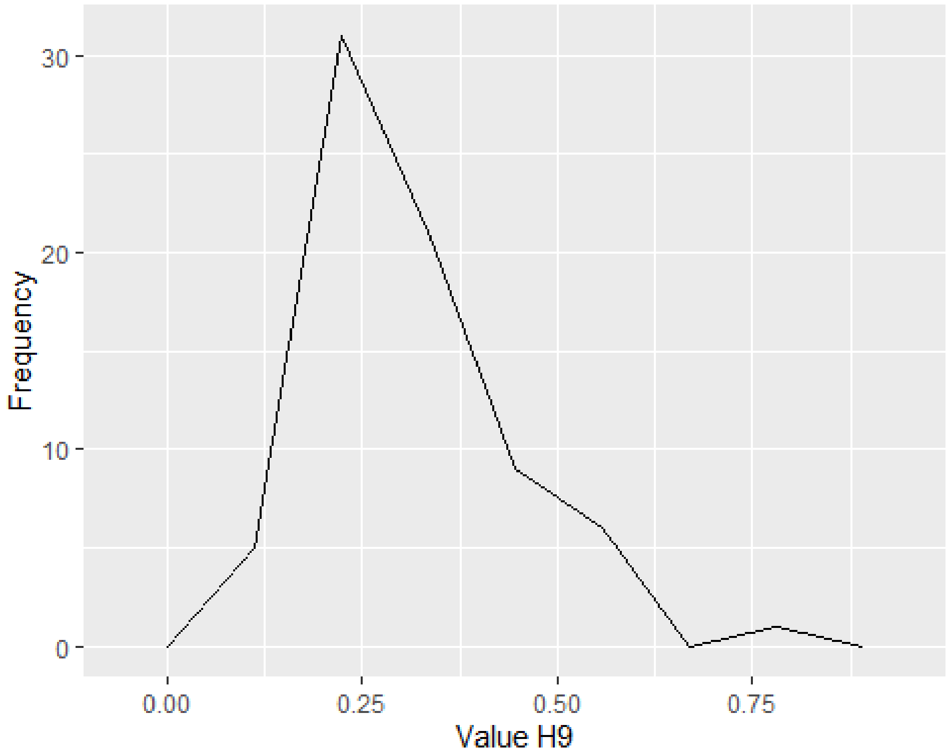


```
ggplot(B) +
  geom_freqpoly(aes(x = HAsfc36), bins = 7) +
  labs(
    x = "Value H36",
    y = "Frequency"
  )
```

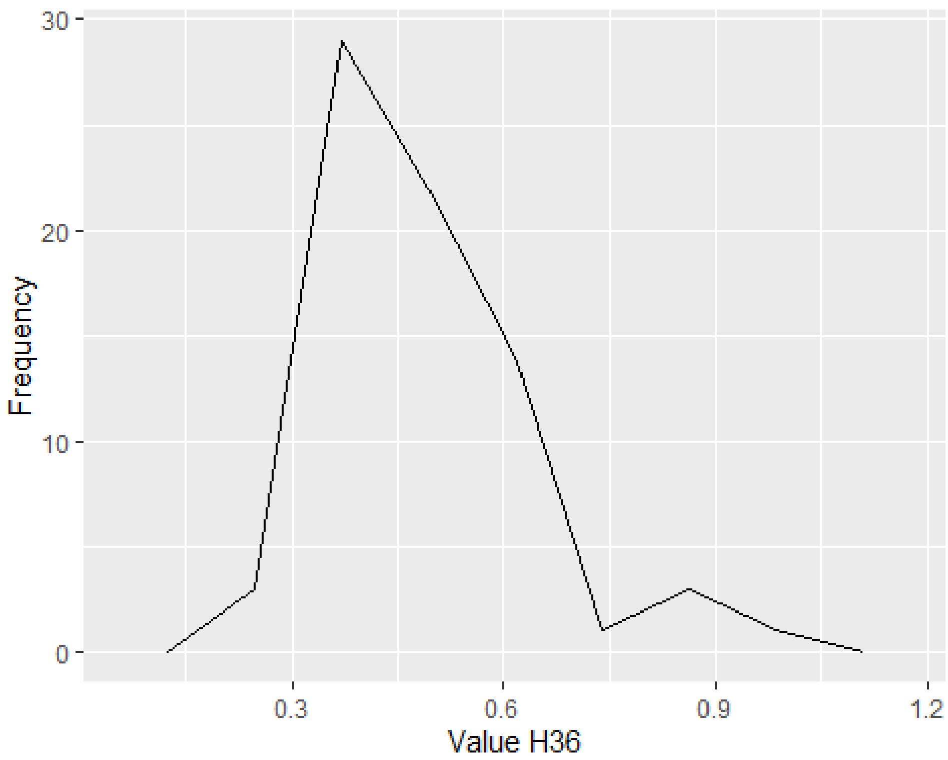


```
ggplot(B) +
  geom_freqpoly(aes(x = HAsfc81), bins = 7) +
  labs(
    x = "Value H81",
    y = "Frequency"
  )
```

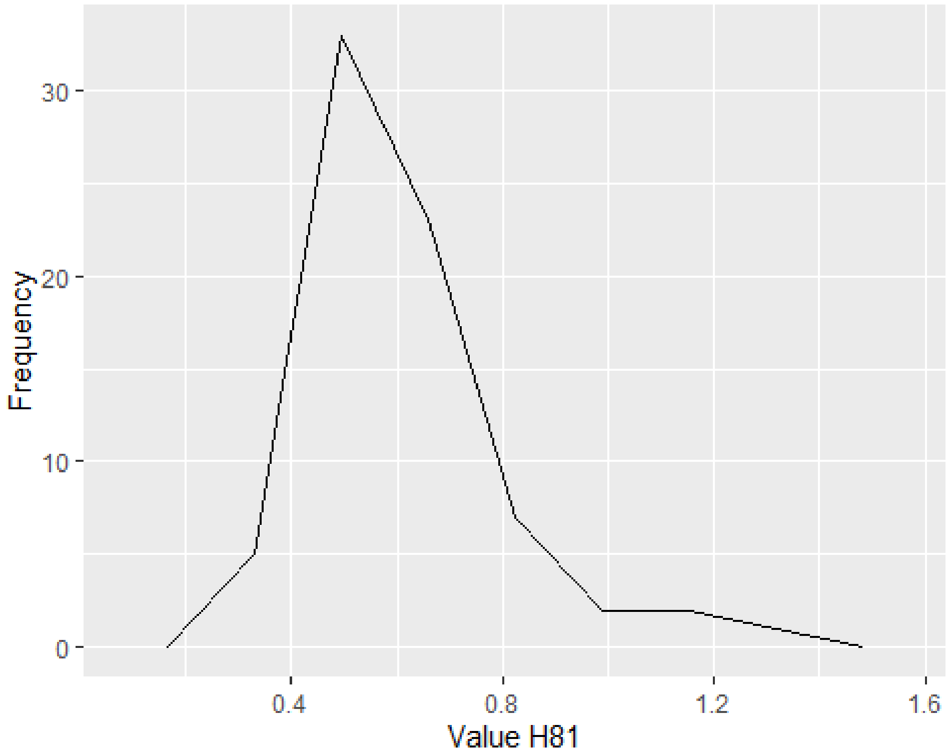

Homoscedasticity: Brown & Forsythe test (and data transformation whenever needed)

```
bf.test(B_Asfc ~ B_Species, data = B)

##
##   Brown-Forsythe Test (alpha = 0.05)
## -------------------------------------------------------------
##   data : B_Asfc and B_Species
##
##   statistic  : 0.3091285
##   num df     : 2
##   denom df   : 35.48762
##   p.value    : 0.7360431
##
##   Result     : Difference is not statistically significant.
## -------------------------------------------------------------

ggplot(B) +
  geom_boxplot(aes(x = B_Species, y = B_Asfc)) +
  labs(
    x = "Species",
    y = "Asfc"
  )
```

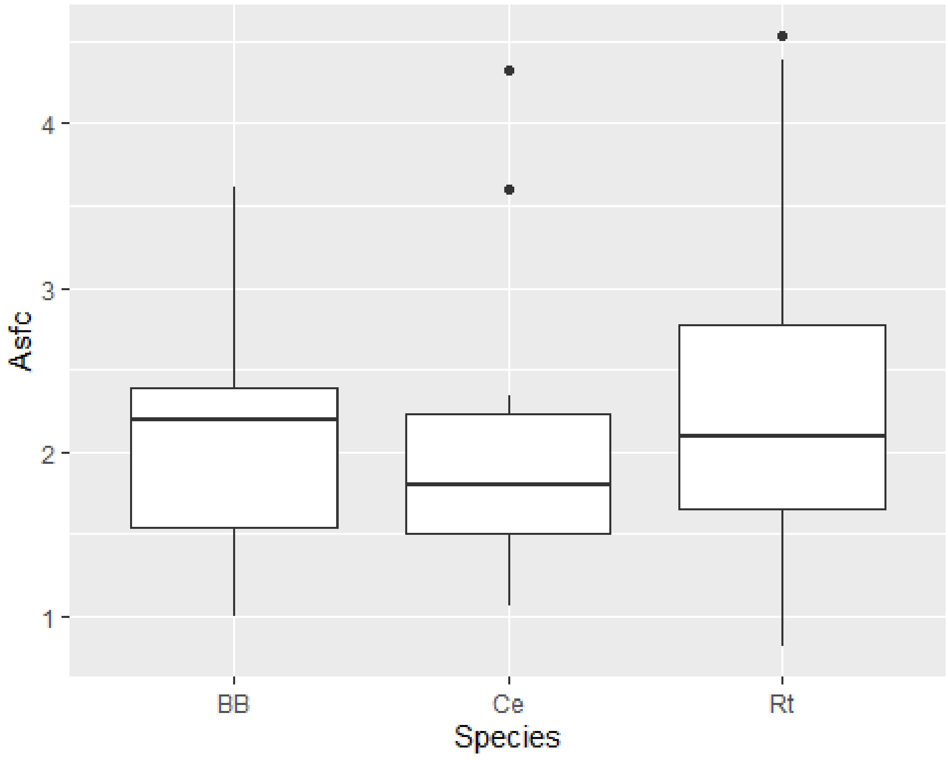


```
bf.test(B_epLsar ~ B_Species, data = B)

##
##   Brown-Forsythe Test (alpha = 0.05)
## -------------------------------------------------------------
##   data : B_epLsar and B_Species
##
##   statistic  : 2.38737
##   num df     : 2
##   denom df   : 40.16194
##   p.value    : 0.1047909
##
##   Result     : Difference is not statistically significant.
## -------------------------------------------------------------

ggplot(B) +
  geom_boxplot(aes(x = B_Species, y = B_epLsar)) +
  labs(
    x = "Species",
    y = "epLsar"
  )
```

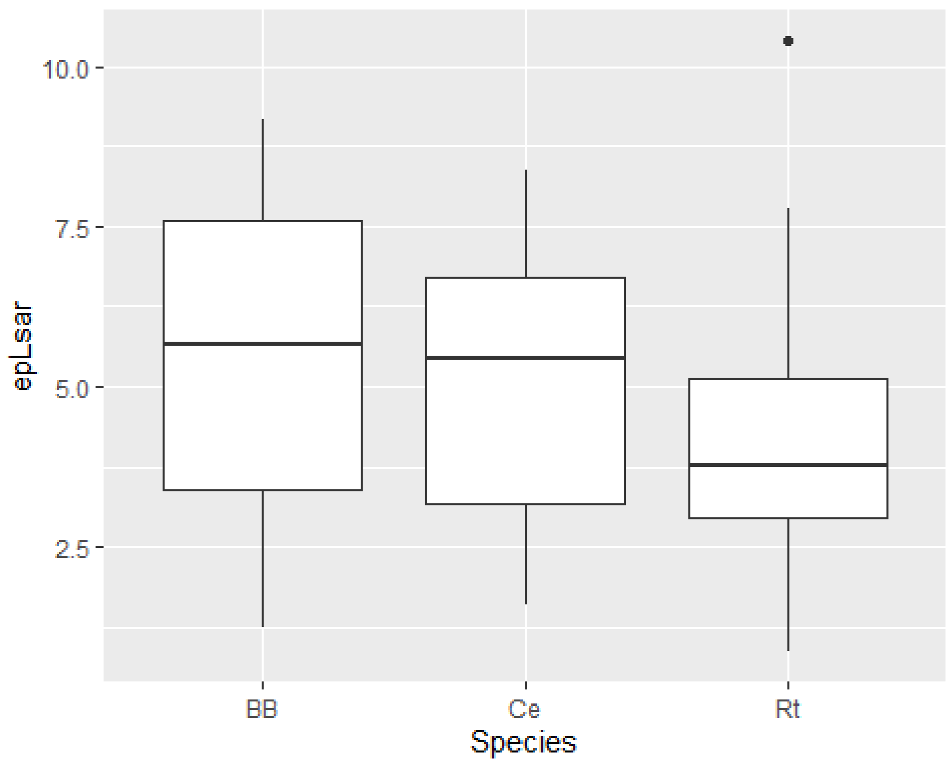


```
bf.test(B_Smc ~ B_Species, data = B)

##
##   Brown-Forsythe Test (alpha = 0.05)
## -------------------------------------------------------------
##   data : B_Smc and B_Species
##
##   statistic   : 1.892354
##   num df      : 2
##   denom df    : 35.21828
##   p.value     : 0.1657263
##
##   Result      : Difference is not statistically significant.
## -------------------------------------------------------------

ggplot(B) +
  geom_boxplot(aes(x = B_Species, y = B_Smc)) +
  labs(
    x = "Species",
    y = "Smc"
  )
```

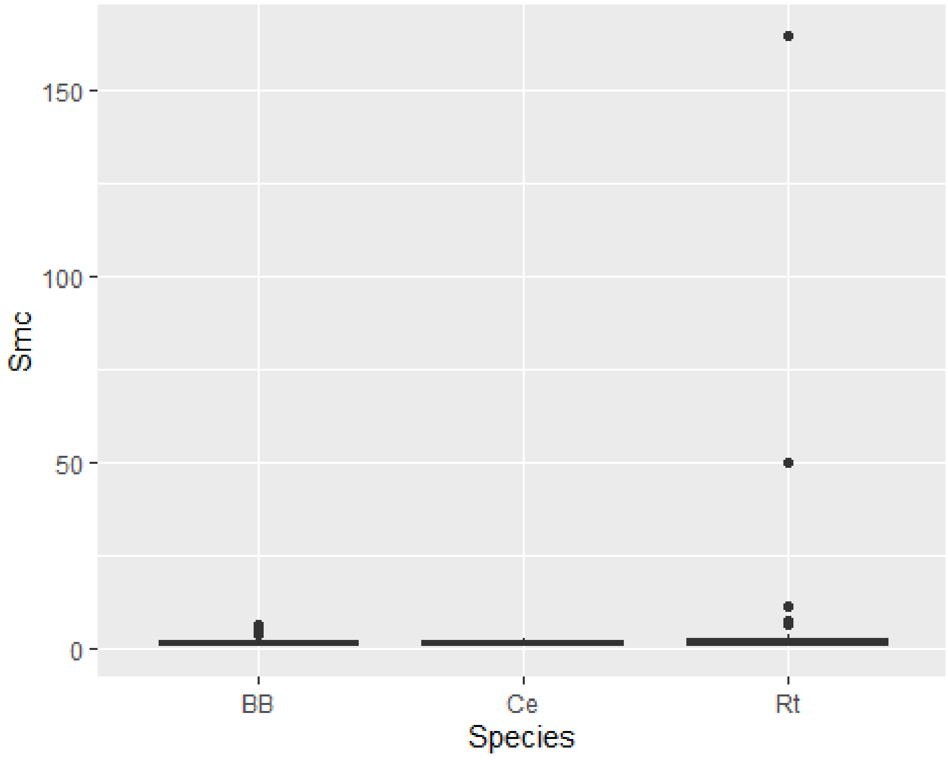


```r
bf.test(B_H9 ~ B_Species, data = B)

##
##   Brown-Forsythe Test (alpha = 0.05)
## -------------------------------------------------------------
##   data : B_H9 and B_Species
##
##   statistic  : 1.80894
##   num df     : 2
##   denom df   : 38.95334
##   p.value    : 0.1773199
##
##   Result     : Difference is not statistically significant.
## -------------------------------------------------------------

ggplot(B) +
  geom_boxplot(aes(x = B_Species, y = B_H9)) +
  labs(
    x = "Species",
    y = "H9"
  )
```

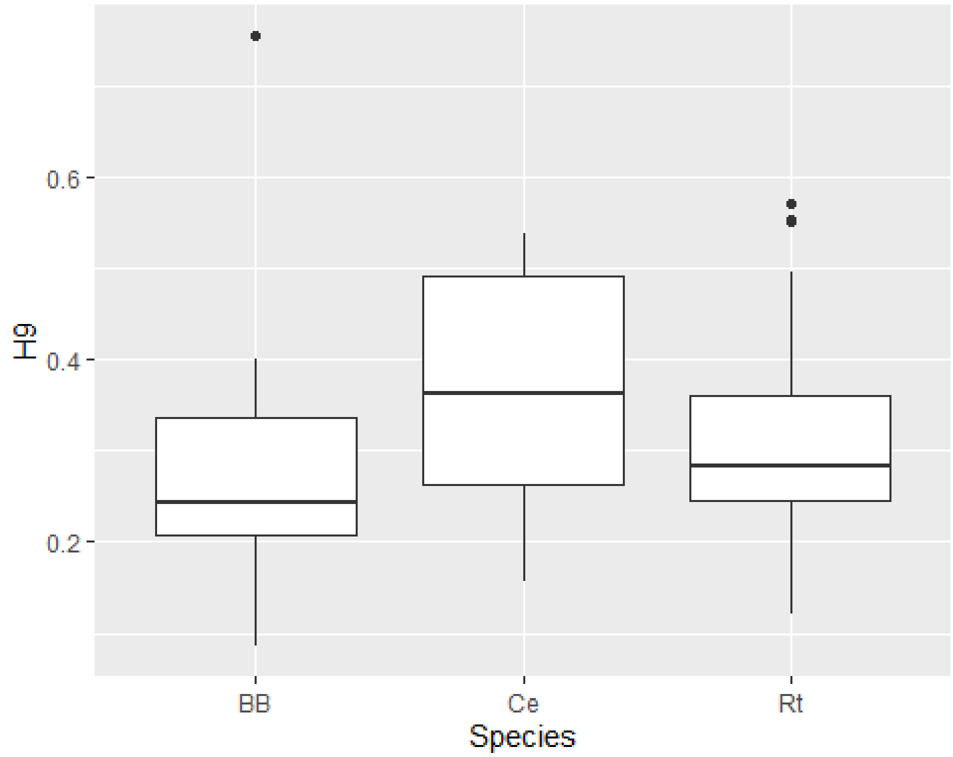


```
bf.test(B_H36 ~ B_Species, data = B)

##
##   Brown-Forsythe Test (alpha = 0.05)
## -------------------------------------------------------------
##   data : B_H36 and B_Species
##
##   statistic  : 1.53086
##   num df     : 2
##   denom df   : 40.35063
##   p.value    : 0.2286505
##
##   Result     : Difference is not statistically significant.
## -------------------------------------------------------------

ggplot(B) +
  geom_boxplot(aes(x = B_Species, y = B_H36)) +
  labs(
    x = "Species",
    y = "H36"
  )
```

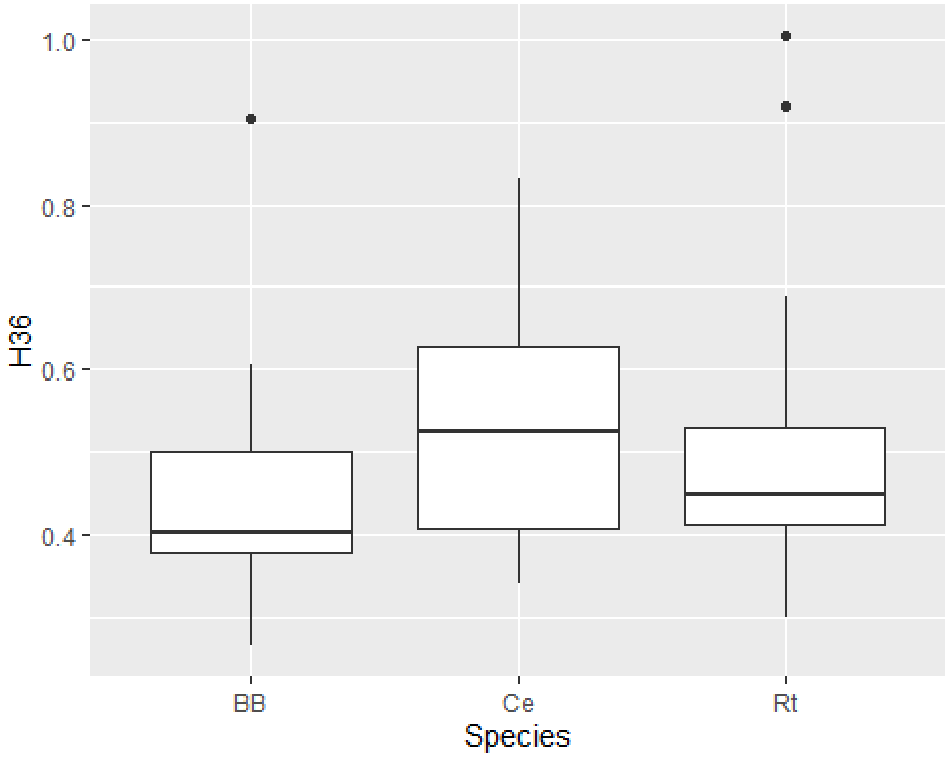


```
bf.test(B_H81 ~ B_Species, data = B)

## 
##   Brown-Forsythe Test (alpha = 0.05) 
## ------------------------------------------------------------- 
##   data : B_H81 and B_Species 
## 
##   statistic  : 0.7870624 
##   num df     : 2 
##   denom df   : 46.62961 
##   p.value    : 0.4611328 
## 
##   Result     : Difference is not statistically significant. 
## -------------------------------------------------------------

ggplot(B) +
  geom_boxplot(aes(x = B_Species, y = B_H81)) +
  labs(
    x = "Species",
    y = "H81"
  )
```

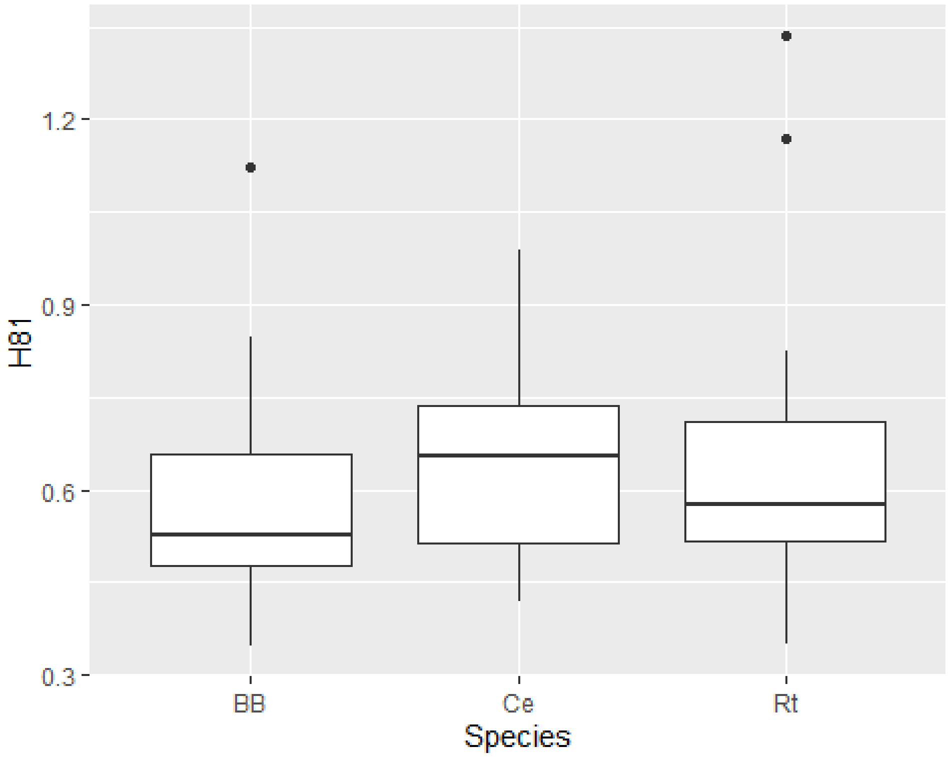

Glm: Impact of blocks over each DMTA parameter

```
glm_B_Asfc0 <- glm(B_Asfc ~ 1, data = B)
glm_B_Asfc1 <- glm(B_Asfc ~ B_Species, data = B)
Cand.models <- list()
Cand.models[[1]] <- glm_B_Asfc0
Cand.models[[2]] <- glm_B_Asfc1
Modnames <- lapply(Cand.models, "formula")
aictab(cand.set = Cand.models, modnames = paste0(Modnames), sort = TRUE)

##
## Model selection based on AICc:
##
##                      K   AICc Delta_AICc AICcWt Cum.Wt     LL
## B_Asfc ~ 1           2 186.45       0.00   0.87   0.87 -91.14
## B_Asfc ~ B_Species 4 190.19       3.74   0.13   1.00 -90.80

glm_B_epLsar0 <- glm(B_epLsar ~ 1, data = B)
glm_B_epLsar1 <- glm(B_epLsar ~ B_Species, data = B)
Cand.models <- list()
Cand.models[[1]] <- glm_B_epLsar0
Cand.models[[2]] <- glm_B_epLsar1
Modnames <- lapply(Cand.models, "formula")
aictab(cand.set = Cand.models, modnames = paste0(Modnames), sort = TRUE)

##
## Model selection based on AICc:
##
```

```
##                        K   AICc Delta_AICc AICcWt Cum.Wt      LL
## B_epLsar ~ B_Species 4 327.98       0.0    0.6    0.6 -159.70
## B_epLsar ~ 1         2 328.78       0.8    0.4    1.0 -162.31
```

```
summary(glm_B_epLsar1)
```

```
##
## Call:
## glm(formula = B_epLsar ~ B_Species, data = B)
##
## Deviance Residuals:
##     Min       1Q   Median       3Q      Max
## -4.2094  -1.6184   0.0486   1.4271   6.2081
##
## Coefficients:
##             Estimate Std. Error t value Pr(>|t|)
## (Intercept)   5.4524     0.4405  12.377   <2e-16 ***
## B_SpeciesCe  -0.3190     0.7735  -0.412   0.6813
## B_SpeciesRt  -1.2585     0.5734  -2.195   0.0315 *
## ---
## Signif. codes:  0 '***' 0.001 '**' 0.01 '*' 0.05 '.' 0.1 ' ' 1
##
## (Dispersion parameter for gaussian family taken to be 4.851638)
##
##     Null deviance: 364.78  on 72  degrees of freedom
## Residual deviance: 339.61  on 70  degrees of freedom
## AIC: 327.39
##
## Number of Fisher Scoring iterations: 2
```

```
marginal <- emmeans(glm_B_epLsar1, ~B_Species)
pairs(marginal)
```

```
##  contrast estimate    SE df t.ratio p.value
##  BB - Ce     0.319 0.774 70   0.412  0.9107
##  BB - Rt     1.258 0.573 70   2.195  0.0791
##  Ce - Rt     0.939 0.734 70   1.280  0.4114
##
## P value adjustment: tukey method for comparing a family of 3 estimates
```

```
glm_B_Smc0 <- glm(B_Smc ~ 1, data = B)
glm_B_Smc1 <- glm(B_Smc ~ B_Species, data = B)
Cand.models <- list()
Cand.models[[1]] <- glm_B_Smc0
Cand.models[[2]] <- glm_B_Smc1
Modnames <- lapply(Cand.models, "formula")
aictab(cand.set = Cand.models, modnames = paste0(Modnames), sort = TRUE)
```

```
##
## Model selection based on AICc:
##
```

```
##                      K   AICc Delta_AICc AICcWt Cum.Wt       LL
## B_Smc ~ 1            2 646.82       0.00   0.77   0.77 -321.33
## B_Smc ~ B_Species 4 649.26       2.44   0.23   1.00 -320.34
```

```
glm_B_H9_0 <- glm(B_H9 ~ 1, data = B)
glm_B_H9_1 <- glm(B_H9 ~ B_Species, data = B)
Cand.models <- list()
Cand.models[[1]] <- glm_B_H9_0
Cand.models[[2]] <- glm_B_H9_1
Modnames <- lapply(Cand.models, "formula")
aictab(cand.set = Cand.models, modnames = paste0(Modnames), sort = TRUE)
```

```
##
## Model selection based on AICc:
##
##                    K   AICc Delta_AICc AICcWt Cum.Wt    LL
## B_H9 ~ 1           2 -95.23       0.00   0.55   0.55 49.70
## B_H9 ~ B_Species 4 -94.85       0.38   0.45   1.00 51.72
```

```
glm_B_H36_0 <- glm(B_H36 ~ 1, data = B)
glm_B_H36_1 <- glm(B_H36 ~ B_Species, data = B)
Cand.models <- list()
Cand.models[[1]] <- glm_B_H36_0
Cand.models[[2]] <- glm_B_H36_1
Modnames <- lapply(Cand.models, "formula")
aictab(cand.set = Cand.models, modnames = paste0(Modnames), sort = TRUE)
```

```
##
## Model selection based on AICc:
##
##                     K   AICc Delta_AICc AICcWt Cum.Wt    LL
## B_H36 ~ 1           2 -74.04        0.0   0.65   0.65 39.11
## B_H36 ~ B_Species 4 -72.85        1.2   0.35   1.00 40.72
```

```
glm_B_H81_0 <- glm(B_H81 ~ 1, data = B)
glm_B_H81_1 <- glm(B_H81 ~ B_Species, data = B)
Cand.models <- list()
Cand.models[[1]] <- glm_B_H81_0
Cand.models[[2]] <- glm_B_H81_1
Modnames <- lapply(Cand.models, "formula")
aictab(cand.set = Cand.models, modnames = paste0(Modnames), sort = TRUE)
```

```
##
## Model selection based on AICc:
##
##                     K   AICc Delta_AICc AICcWt Cum.Wt    LL
## B_H81 ~ 1           2 -38.56       0.00   0.81   0.81 21.37
## B_H81 ~ B_Species 4 -35.70       2.87   0.19   1.00 22.14
```

Block D

```
D_Species <- D %>%
  dplyr::select(c(2)) %>%
  unlist(c(1))

D_Asfc <- D %>%
  dplyr::select(c(7)) %>%
  unlist(c(1))
D_epLsar <- D %>%
  dplyr::select(c(8)) %>%
  unlist(c(1))
D_Smc <- D %>%
  dplyr::select(c(9)) %>%
  unlist(c(1))
D_H9 <- D %>%
  dplyr::select(c(10)) %>%
  unlist(c(1))
D_H36 <- D %>%
  dplyr::select(c(12)) %>%
  unlist(c(1))
D_H81 <- D %>%
  dplyr::select(c(11)) %>%
  unlist(c(1))
```

Checking data distribution and outliers:

```
x <- D[order(D_Asfc), ]
x$specie <- factor(x$specie)
dotchart(x$Asfc, cex = 1, pch = 16, groups = x$specie, xlab = "D_Asfc per spe
cie")
```

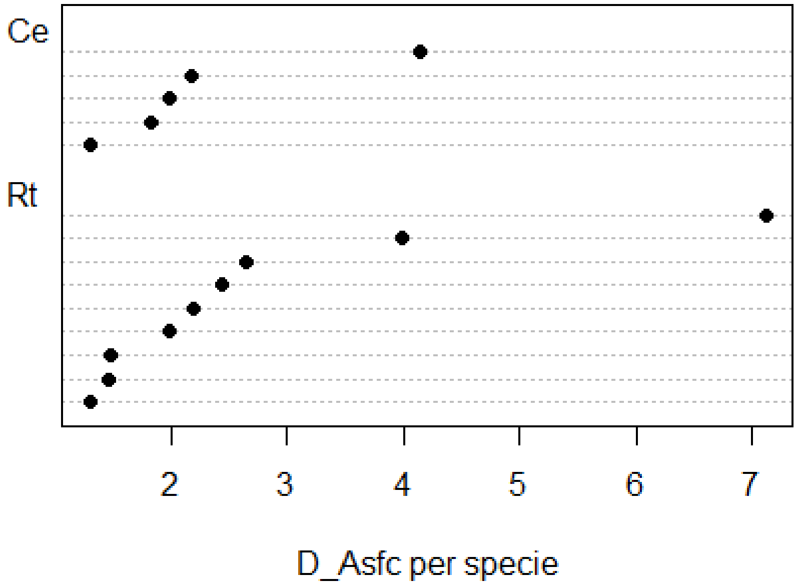


```
x <- D[order(D_epLsar), ]
x$specie <- factor(x$specie)
dotchart(x$epLsar, cex = 1, pch = 16, groups = x$specie, xlab = "D_epLsar per
specie")
```

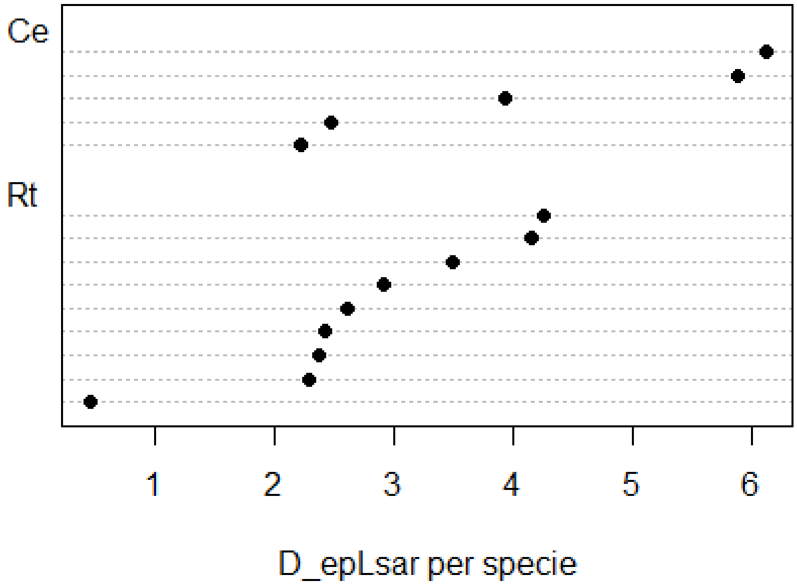


```
x <- D[order(D_Smc), ]
x$specie <- factor(x$specie)
dotchart(x$Smc, cex = 1, pch = 16, groups = x$specie, xlab = "D_Smc per speci
e")
```

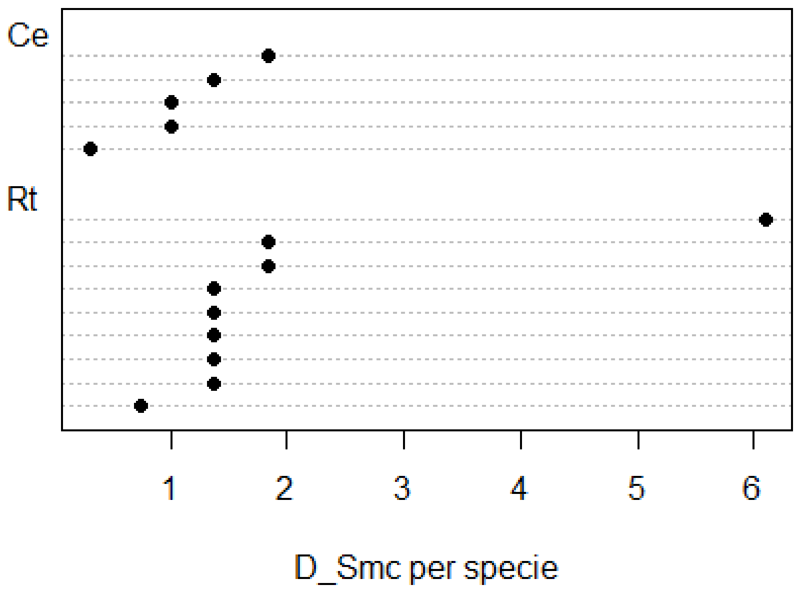


```
x <- D[order(D_H9), ]
x$specie <- factor(x$specie)
dotchart(x$HAsfc9, cex = 1, pch = 16, groups = x$specie, xlab = "D_H9 per spe
cie")
```

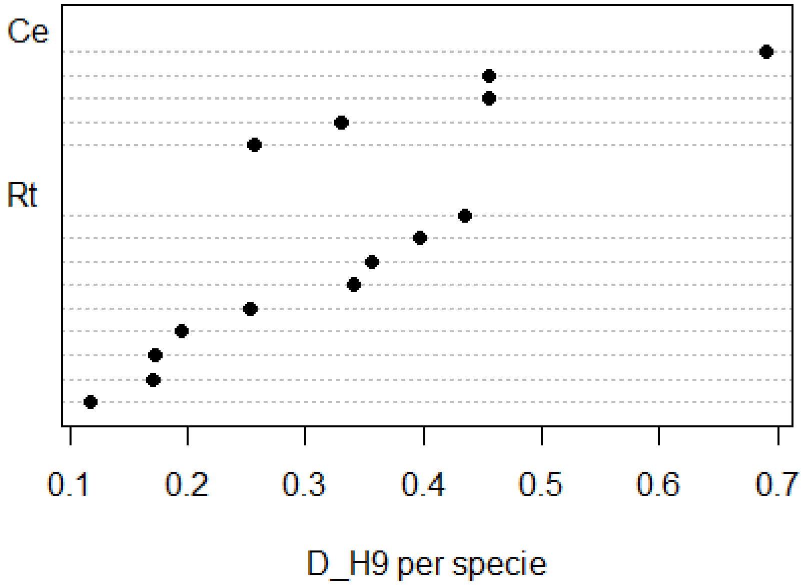


```
x <- D[order(D_H36), ]
x$specie <- factor(x$specie)
dotchart(x$HAsfc36, cex = 1, pch = 16, groups = x$specie, xlab = "D_H36 per s
pecie")
```

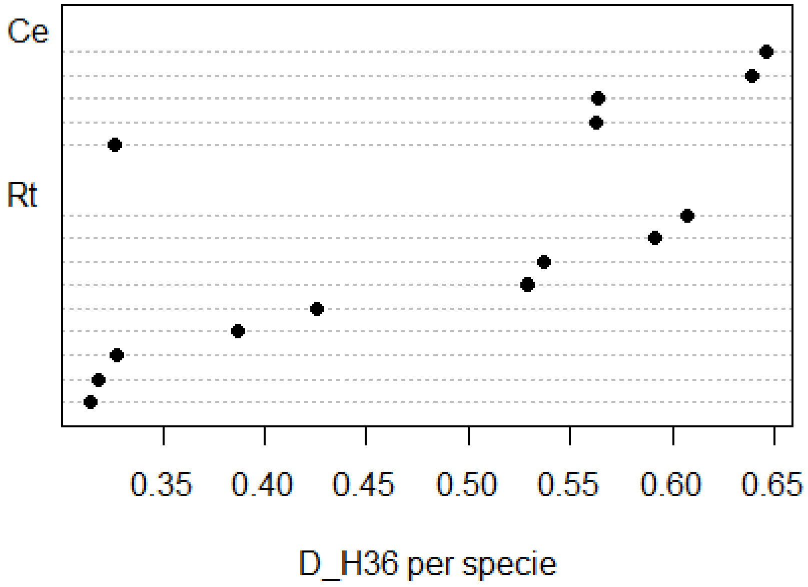


```
x <- D[order(D_H81), ]
x$specie <- factor(x$specie)
dotchart(x$HAsfc81, cex = 1, pch = 16, groups = x$specie, xlab = "D_H81 per s
pecie")
```

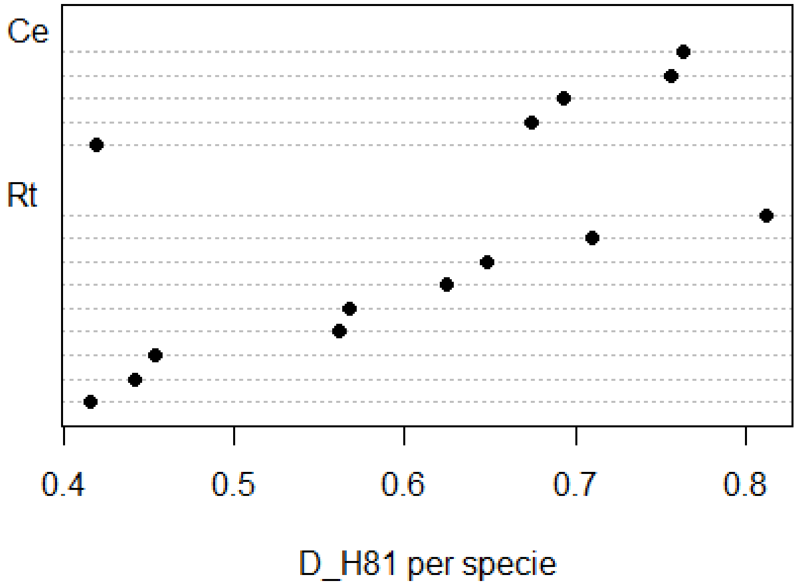

Graphical evaluation of the tests’ applicability:

Normality and homoscedasticity of the variables. #### Normality

```
ggplot(D) +
  geom_freqpoly(aes(x = Asfc), bins = 7) +
  labs(
    x = "Value Asfc",
    y = "Frequency"
  )
```

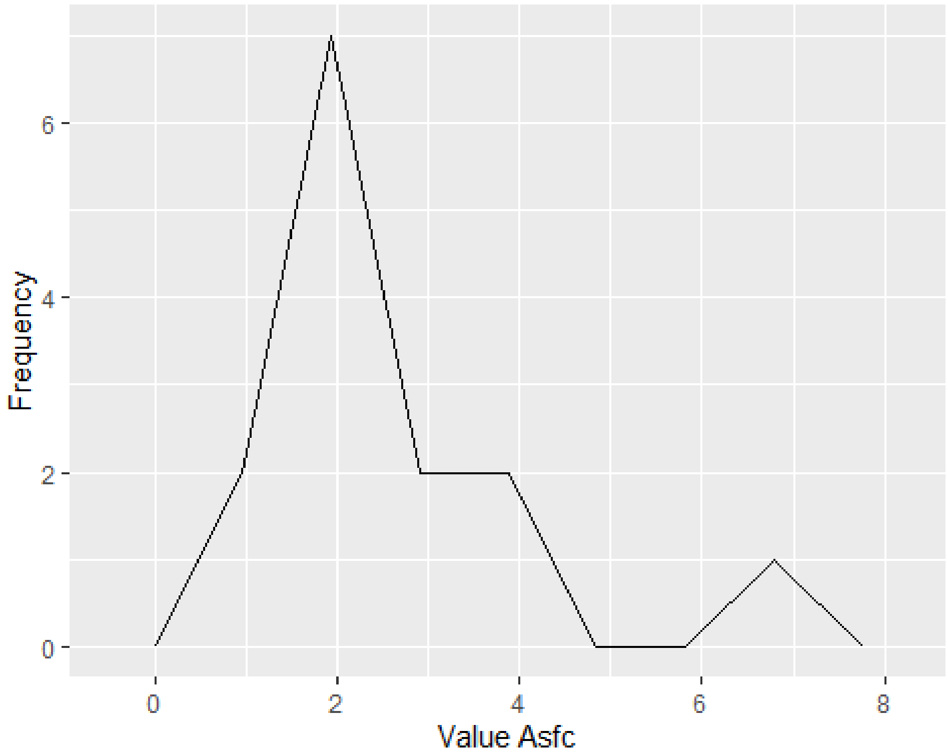


```
ggplot(D) +
  geom_freqpoly(aes(x = epLsar), bins = 7) +
  labs(
    x = "Value epLsar",
    y = "Frequency"
  )
```

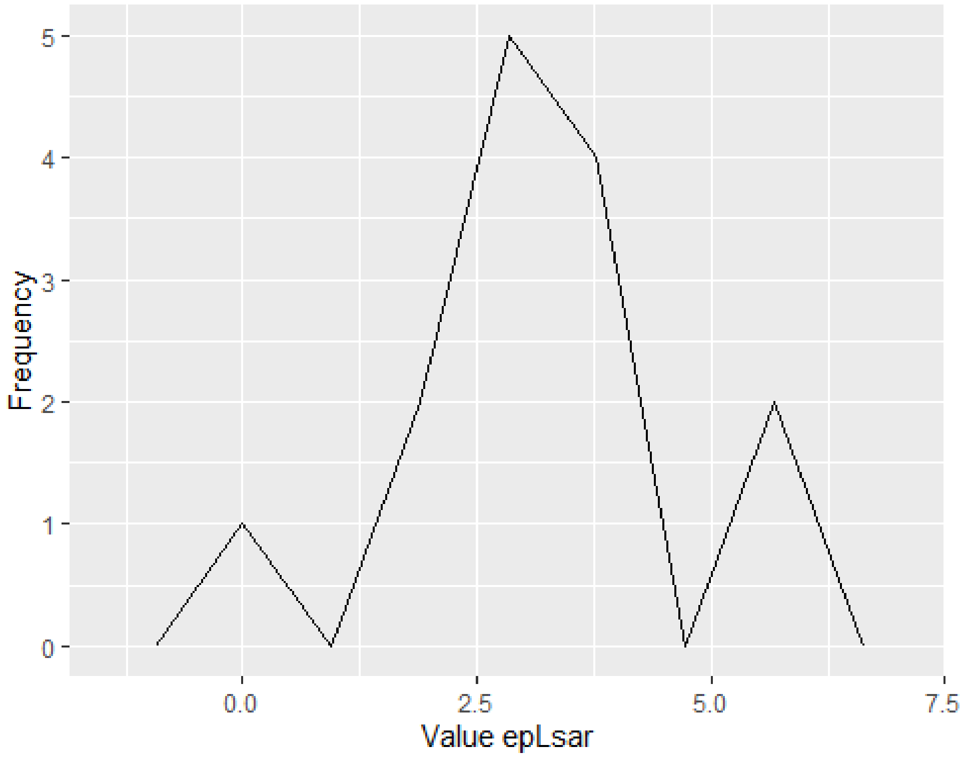


```
ggplot(D) +
  geom_freqpoly(aes(x = Smc), bins = 7) +
  labs(
    x = "Value Smc",
    y = "Frequency"
  )
```

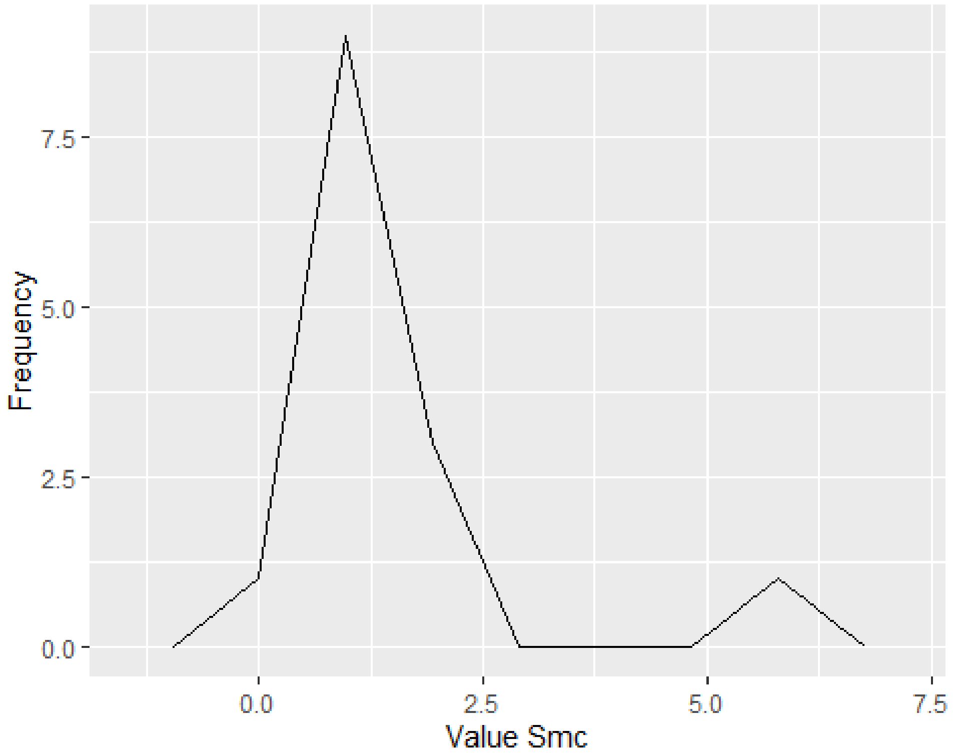


```
ggplot(D) +
  geom_freqpoly(aes(x = HAsfc9), bins = 7) +
  labs(
    x = "Value H9",
    y = "Frequency"
  )
```

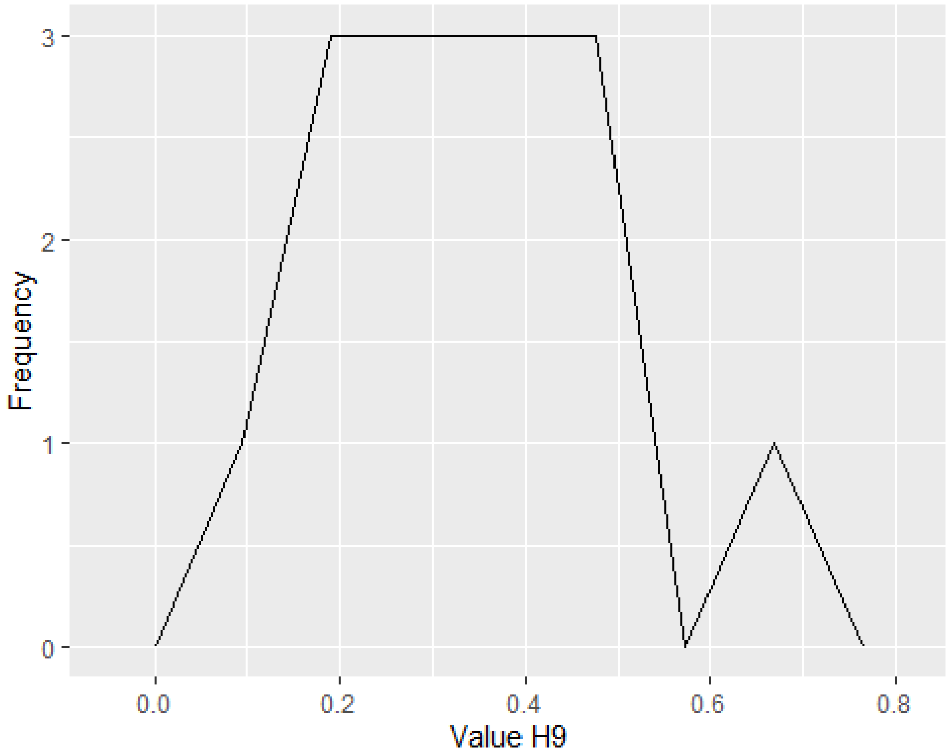


```
ggplot(D) +
  geom_freqpoly(aes(x = HAsfc36), bins = 7) +
  labs(
    x = "Value H36",
    y = "Frequency"
  )
```

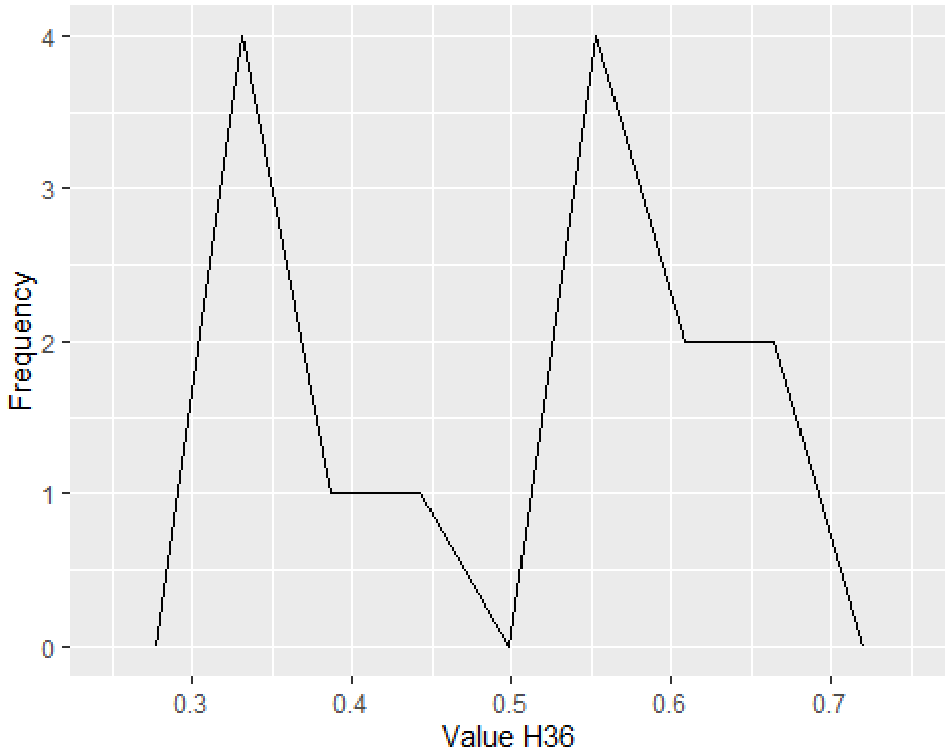


```
ggplot(D) +
  geom_freqpoly(aes(x = HAsfc81), bins = 7) +
  labs(
    x = "Value H81",
    y = "Frequency"
  )
```

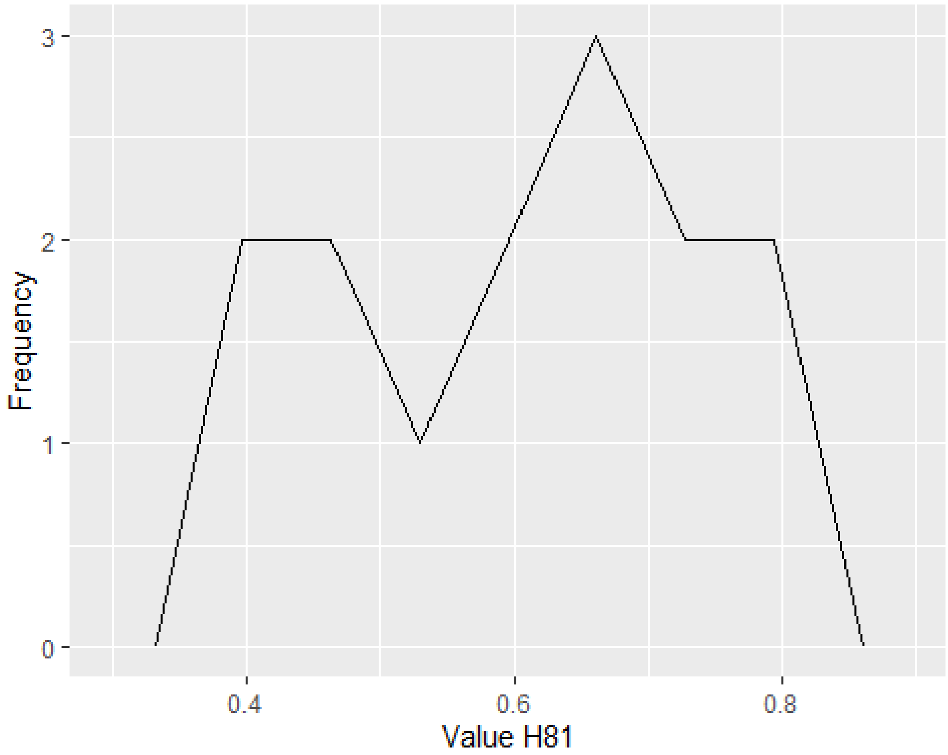

Homoscedasticity: Brown & Forsythe test (and data transformation whenever needed)

```
bf.test(D_Asfc ~ D_Species, data = D)

##
##   Brown-Forsythe Test (alpha = 0.05)
## -------------------------------------------------------------
##   data : D_Asfc and D_Species
##
##   statistic  : 0.3270361
##   num df     : 1
##   denom df   : 11.84389
##   p.value    : 0.578102
##
##   Result     : Difference is not statistically significant.
## -------------------------------------------------------------

ggplot(D) +
  geom_boxplot(aes(x = D_Species, y = D_Asfc)) +
  labs(
    x = "Species",
    y = "Asfc"
  )
```

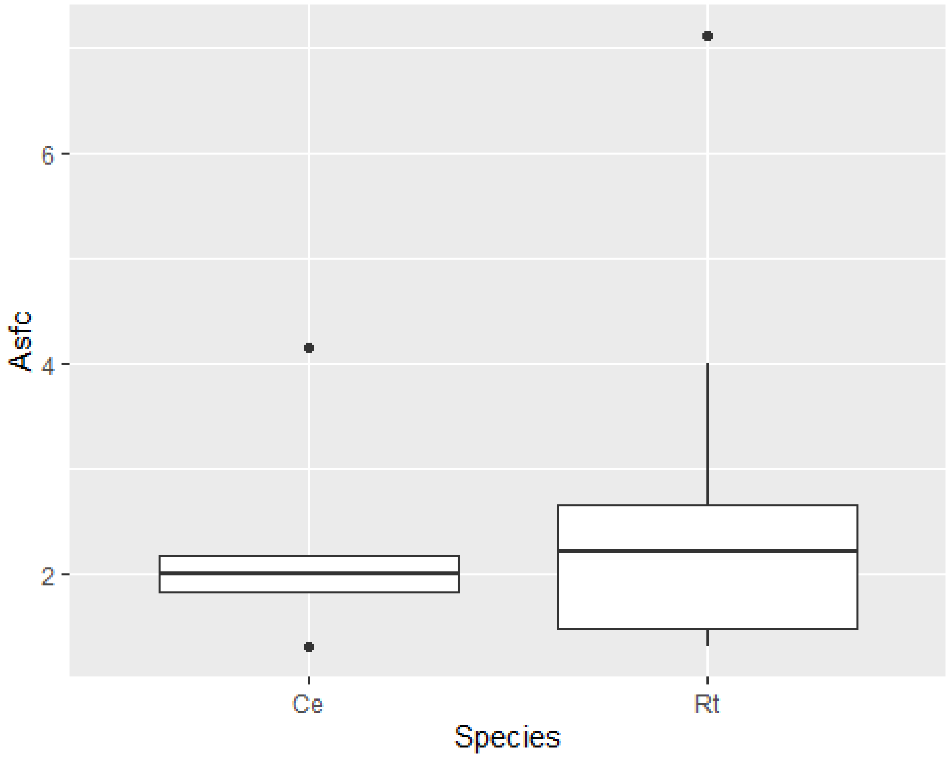


```
bf.test(D_epLsar ~ D_Species, data = D)

##
##   Brown-Forsythe Test (alpha = 0.05)
## -------------------------------------------------------------
##   data : D_epLsar and D_Species
##
##   statistic  : 2.23538
##   num df     : 1
##   denom df   : 5.801727
##   p.value    : 0.1871656
##
##   Result     : Difference is not statistically significant.
## -------------------------------------------------------------

ggplot(D) +
  geom_boxplot(aes(x = D_Species, y = D_epLsar)) +
  labs(
    x = "Species",
    y = "epLsar"
  )
```

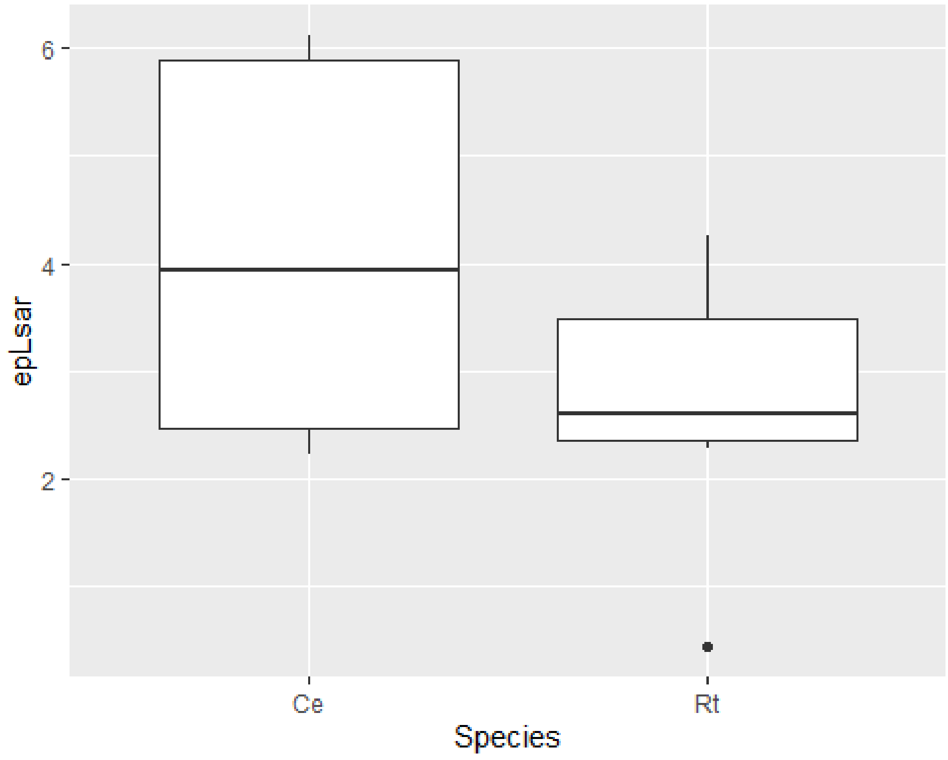


```
bf.test(D_Smc ~ D_Species, data = D)

##
##   Brown-Forsythe Test (alpha = 0.05)
## -------------------------------------------------------------
##   data : D_Smc and D_Species
##
##   statistic  : 1.946999
##   num df     : 1
##   denom df   : 10.88292
##   p.value    : 0.1907299
##
##   Result     : Difference is not statistically significant.
## -------------------------------------------------------------

ggplot(D) +
  geom_boxplot(aes(x = D_Species, y = D_Smc)) +
  labs(
    x = "Species",
    y = "Smc"
  )
```

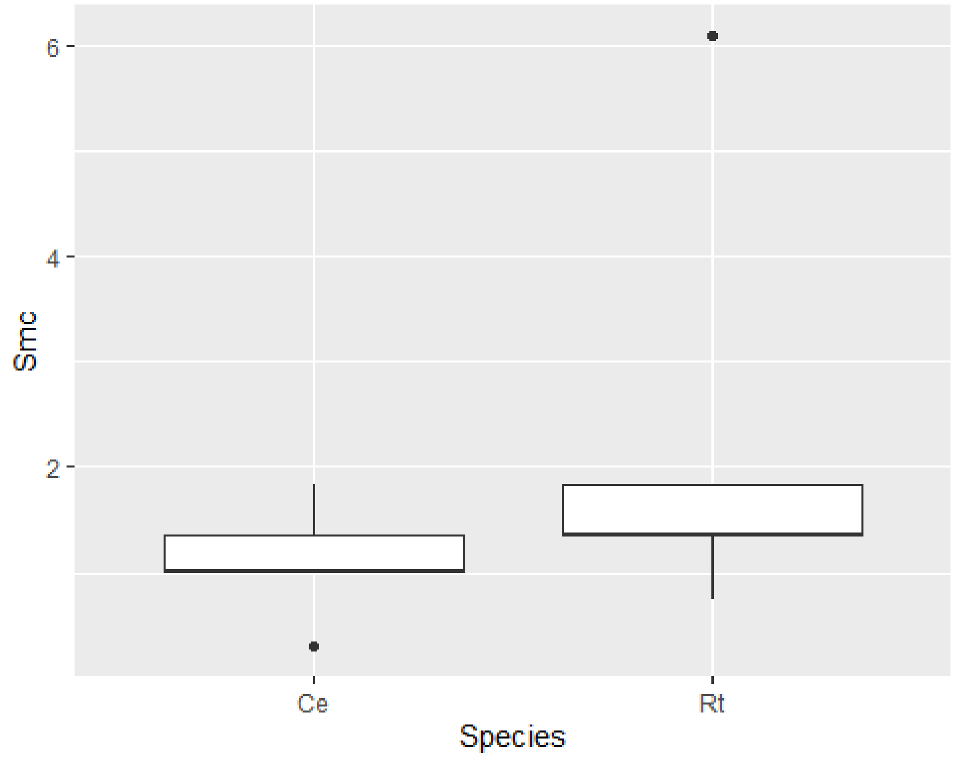


```
bf.test(D_H9 ~ D_Species, data = D)

##
##   Brown-Forsythe Test (alpha = 0.05)
## -------------------------------------------------------------
##   data : D_H9 and D_Species
##
##   statistic  : 4.034018
##   num df     : 1
##   denom df   : 6.206324
##   p.value    : 0.0897655
##
##   Result     : Difference is not statistically significant.
## -------------------------------------------------------------

ggplot(D) +
  geom_boxplot(aes(x = D_Species, y = D_H9)) +
  labs(
    x = "Species",
    y = "H9"
  )
```

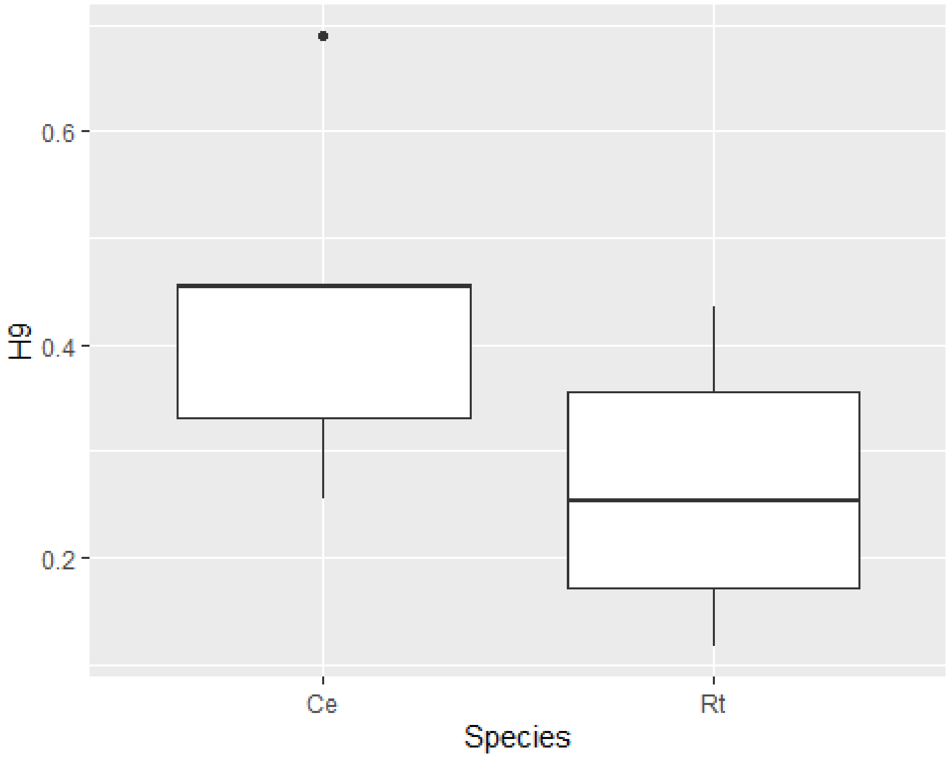


```
bf.test(D_H36 ~ D_Species, data = D)

##
##   Brown-Forsythe Test (alpha = 0.05)
## -------------------------------------------------------------
##   data : D_H36 and D_Species
##
##   statistic  : 1.974381
##   num df     : 1
##   denom df   : 7.775952
##   p.value    : 0.1986521
##
##   Result     : Difference is not statistically significant.
## -------------------------------------------------------------

ggplot(D) +
  geom_boxplot(aes(x = D_Species, y = D_H36)) +
  labs(
    x = "Species",
    y = "H36"
  )
```

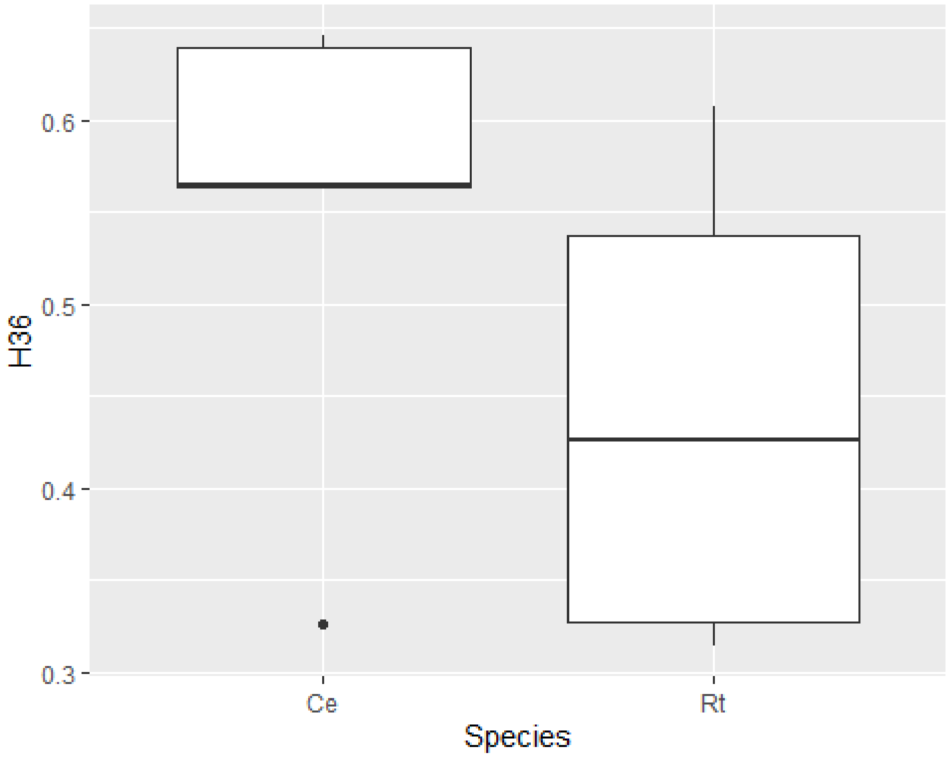


```
bf.test(D_H81 ~ D_Species, data = D)

## 
##   Brown-Forsythe Test (alpha = 0.05) 
## ------------------------------------------------------------- 
##   data : D_H81 and D_Species 
## 
##   statistic  : 1.069055 
##   num df     : 1 
##   denom df   : 7.932806 
##   p.value    : 0.3316475 
## 
##   Result     : Difference is not statistically significant. 
## -------------------------------------------------------------

ggplot(D) +
  geom_boxplot(aes(x = D_Species, y = D_H81)) +
  labs(
    x = "Species",
    y = "H81"
  )
```

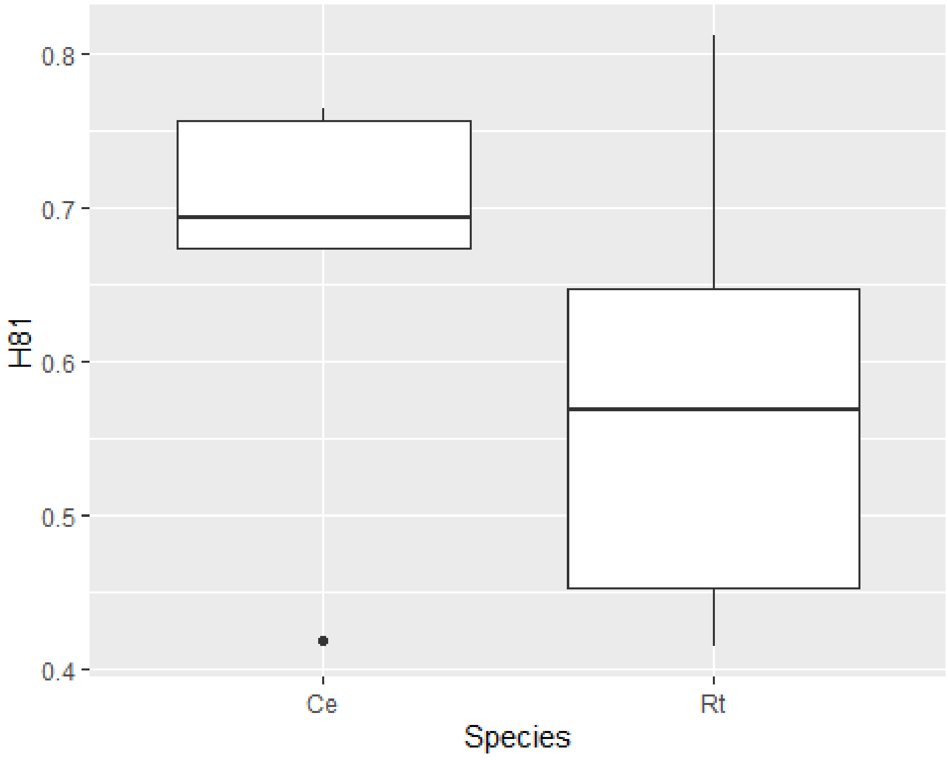

Glm: Impact of blocks over each DMTA parameter

```
glm_D_Asfc0 <- glm(D_Asfc ~ 1, data = D)
glm_D_Asfc1 <- glm(D_Asfc ~ D_Species, data = D)
Cand.models <- list()
Cand.models[[1]] <- glm_D_Asfc0
Cand.models[[2]] <- glm_D_Asfc1
Modnames <- lapply(Cand.models, "formula")
aictab(cand.set = Cand.models, modnames = paste0(Modnames), sort = TRUE)

##
## Model selection based on AICc:
##
##                    K  AICc Delta_AICc AICcWt Cum.Wt     LL
## D_Asfc ~ 1         2 56.54       0.00   0.82   0.82 -25.72
## D_Asfc ~ D_Species 3 59.56       3.03   0.18   1.00 -25.58

glm_D_epLsar0 <- glm(D_epLsar ~ 1, data = D)
glm_D_epLsar1 <- glm(D_epLsar ~ D_Species, data = D)
Cand.models <- list()
Cand.models[[1]] <- glm_D_epLsar0
Cand.models[[2]] <- glm_D_epLsar1
Modnames <- lapply(Cand.models, "formula")
aictab(cand.set = Cand.models, modnames = paste0(Modnames), sort = TRUE)

##
## Model selection based on AICc:
##
```

```
##                        K  AICc Delta_AICc AICcWt Cum.Wt     LL
## D_epLsar ~ 1           2 55.48       0.00   0.53   0.53 -25.19
## D_epLsar ~ D_Species 3 55.72       0.24   0.47   1.00 -23.66
```

```
glm_D_Smc0 <- glm(D_Smc ~ 1, data = D)
glm_D_Smc1 <- glm(D_Smc ~ D_Species, data = D)
Cand.models <- list()
Cand.models[[1]] <- glm_D_Smc0
Cand.models[[2]] <- glm_D_Smc1
Modnames <- lapply(Cand.models, "formula")
aictab(cand.set = Cand.models, modnames = paste0(Modnames), sort = TRUE)
```

```
##
## Model selection based on AICc:
##
##                     K  AICc Delta_AICc AICcWt Cum.Wt     LL
## D_Smc ~ 1           2 52.25       0.00   0.73   0.73 -23.58
## D_Smc ~ D_Species 3 54.22       1.97   0.27   1.00 -22.91
```

```
glm_D_H9_0 <- glm(D_H9 ~ 1, data = D)
glm_D_H9_1 <- glm(D_H9 ~ D_Species, data = D)
Cand.models <- list()
Cand.models[[1]] <- glm_D_H9_0
Cand.models[[2]] <- glm_D_H9_1
Modnames <- lapply(Cand.models, "formula")
aictab(cand.set = Cand.models, modnames = paste0(Modnames), sort = TRUE)
```

```
##
## Model selection based on AICc:
##
##                    K   AICc Delta_AICc AICcWt Cum.Wt   LL
## D_H9 ~ D_Species 3 -10.44       0.00   0.69   0.69 9.42
## D_H9 ~ 1           2  -8.85       1.59   0.31   1.00 6.97
```

```
summary(glm_D_H9_1)
```

```
##
## Call:
## glm(formula = D_H9 ~ D_Species, data = D)
##
## Deviance Residuals:
##       Min         1Q     Median         3Q        Max
## -0.181600  -0.099639  -0.000244   0.081361   0.252400
##
## Coefficients:
##             Estimate Std. Error t value Pr(>|t|)
## (Intercept)  0.43760    0.05965   7.337 9.02e-06 ***
## D_SpeciesRt -0.16671    0.07439  -2.241   0.0447 *
## ---
## Signif. codes:  0 '***' 0.001 '**' 0.01 '*' 0.05 '.' 0.1 ' ' 1
##
```

```
## (Dispersion parameter for gaussian family taken to be 0.01778801)
##
##     Null deviance: 0.30279  on 13  degrees of freedom
## Residual deviance: 0.21346  on 12  degrees of freedom
## AIC: -12.837
##
## Number of Fisher Scoring iterations: 2
```

```
marginal <- emmeans(glm_D_H9_1, ~D_Species)
pairs(marginal)
```

```
##  contrast estimate     SE df t.ratio p.value
##  Ce - Rt     0.167 0.0744 12   2.241  0.0447
```

```
glm_D_H36_0 <- glm(D_H36 ~ 1, data = D)
glm_D_H36_1 <- glm(D_H36 ~ D_Species, data = D)
Cand.models <- list()
Cand.models[[1]] <- glm_D_H36_0
Cand.models[[2]] <- glm_D_H36_1
Modnames <- lapply(Cand.models, "formula")
aictab(cand.set = Cand.models, modnames = paste0(Modnames), sort = TRUE)
```

```
##
## Model selection based on AICc:
##
##                    K   AICc Delta_AICc AICcWt Cum.Wt    LL
## D_H36 ~ 1          2 -13.75       0.00   0.63   0.63  9.42
## D_H36 ~ D_Species  3 -12.68       1.07   0.37   1.00 10.54
```

```
glm_D_H81_0 <- glm(D_H81 ~ 1, data = D)
glm_D_H81_1 <- glm(D_H81 ~ D_Species, data = D)
Cand.models <- list()
Cand.models[[1]] <- glm_D_H81_0
Cand.models[[2]] <- glm_D_H81_1
Modnames <- lapply(Cand.models, "formula")
aictab(cand.set = Cand.models, modnames = paste0(Modnames), sort = TRUE)
```

```
##
## Model selection based on AICc:
##
##                    K   AICc Delta_AICc AICcWt Cum.Wt   LL
## D_H81 ~ 1          2 -12.11       0.00   0.74   0.74 8.60
## D_H81 ~ D_Species  3 -10.04       2.07   0.26   1.00 9.22
```

Block E

```
global_db_FOSSILES_blocE <- read_delim("global db_FOSSILES_blocE.csv", delim
= ";", escape_double = FALSE, trim_ws = TRUE)
# the only bovid of Block E is taken out of as its occurrence avoid performin
g bf test.
E<-global_db_FOSSILES_blocE
summary (E)
```

```
##     Group              specie             Period             Blocs          
##  Length:14          Length:14          Length:14          Length:14         
##  Class :character   Class :character   Class :character   Class :character  
##  Mode  :character   Mode  :character   Mode  :character   Mode  :character  
##                                                                             
##                                                                             
##                                                                             
##     layer              ref DMTA           Asfc            epLsar     
##  Length:14          Min.   : 92.00   Min.   :0.871   Min.   :2.067  
##  Class :character   1st Qu.: 95.25   1st Qu.:1.379   1st Qu.:2.793  
##  Mode  :character   Median : 99.50   Median :2.329   Median :3.193  
##                     Mean   : 99.07   Mean   :2.219   Mean   :4.114  
##                     3rd Qu.:102.75   3rd Qu.:3.011   3rd Qu.:5.176  
##                     Max.   :106.00   Max.   :3.487   Max.   :7.704  
##       Smc            HAsfc9          HAsfc81          HAsfc36      
##  Min.   : 0.554   Min.   :0.0810   Min.   :0.4150   Min.   :0.3090  
##  1st Qu.: 1.361   1st Qu.:0.2537   1st Qu.:0.5863   1st Qu.:0.4235  
##  Median : 1.361   Median :0.3795   Median :0.6330   Median :0.5500  
##  Mean   : 5.871   Mean   :0.3847   Mean   :0.6726   Mean   :0.5366  
##  3rd Qu.: 1.837   3rd Qu.:0.4880   3rd Qu.:0.8087   3rd Qu.:0.6390  
##  Max.   :49.712   Max.   :0.7670   Max.   :0.9380   Max.   :0.7960  
##   Disp-Asfc-i     Disp-epLsar-i       Disp-H9-i      
##  Min.   :0.0000   Min.   :0.00000   Min.   :0.00000  
##  1st Qu.:0.1098   1st Qu.:0.06925   1st Qu.:0.05225  
##  Median :0.2340   Median :0.19750   Median :0.07100  
##  Mean   :0.2329   Mean   :0.26957   Mean   :0.09629  
##  3rd Qu.:0.3668   3rd Qu.:0.39150   3rd Qu.:0.12850  
##  Max.   :0.5500   Max.   :0.71300   Max.   :0.24200
```

```
E_Species <- E %>%
  dplyr::select(c(2)) %>%
  unlist(c(1))

E_Asfc <- E %>%
  dplyr::select(c(7)) %>%
  unlist(c(1))
E_epLsar <- E %>%
  dplyr::select(c(8)) %>%
  unlist(c(1))
E_Smc <- E %>%
  dplyr::select(c(9)) %>%
  unlist(c(1))
E_H9 <- E %>%
  dplyr::select(c(10)) %>%
  unlist(c(1))
E_H36 <- E %>%
  dplyr::select(c(12)) %>%
  unlist(c(1))
E_H81 <- E %>%
```

```
  dplyr::select(c(11)) %>%
  unlist(c(1))
```

Checking data distribution and outliers:

```
x <- E[order(E_Asfc), ]
x$specie <- factor(x$specie)
dotchart(x$Asfc, cex = 1, pch = 16, groups = x$specie, xlab = "E_Asfc per spe
cie")
```

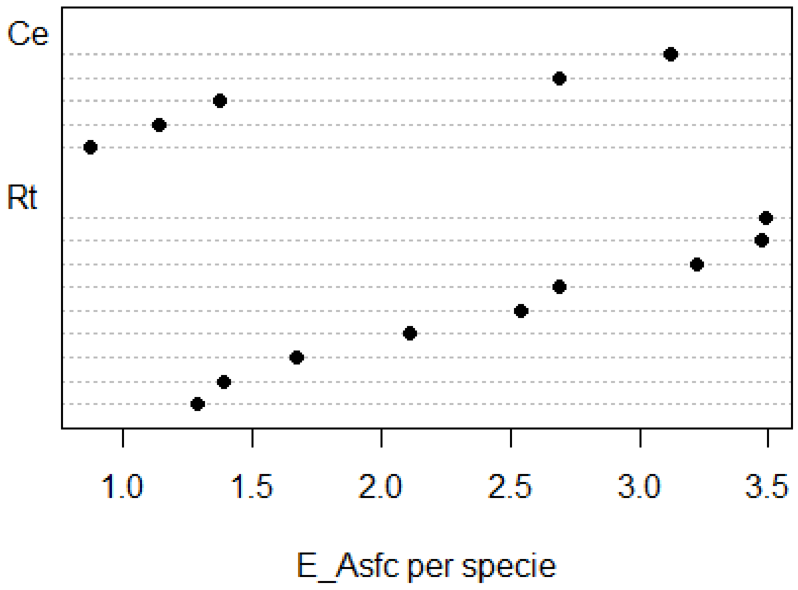


```
x <- E[order(E_epLsar), ]
x$specie <- factor(x$specie)
dotchart(x$epLsar, cex = 1, pch = 16, groups = x$specie, xlab = "E_epLsar per
specie")
```

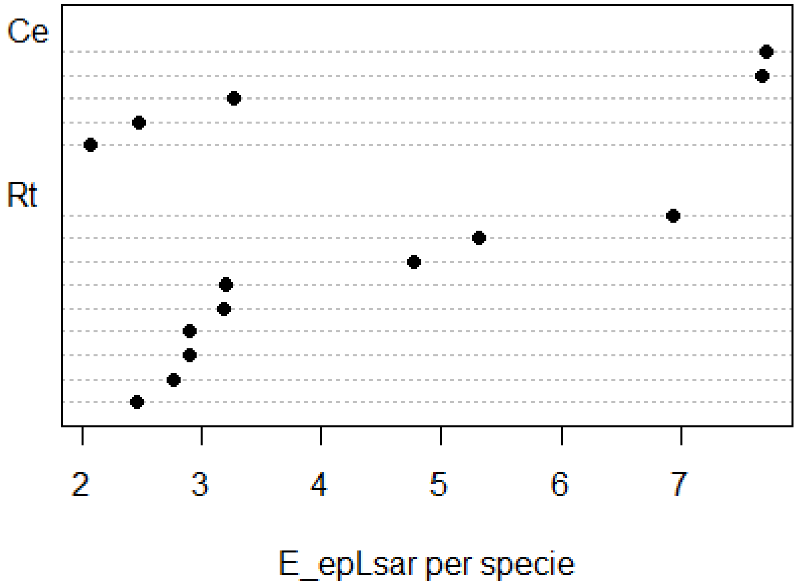


```
x <- E[order(E_Smc), ]
x$specie <- factor(x$specie)
dotchart(x$Smc, cex = 1, pch = 16, groups = x$specie, xlab = "E_Smc per speci
e")
```

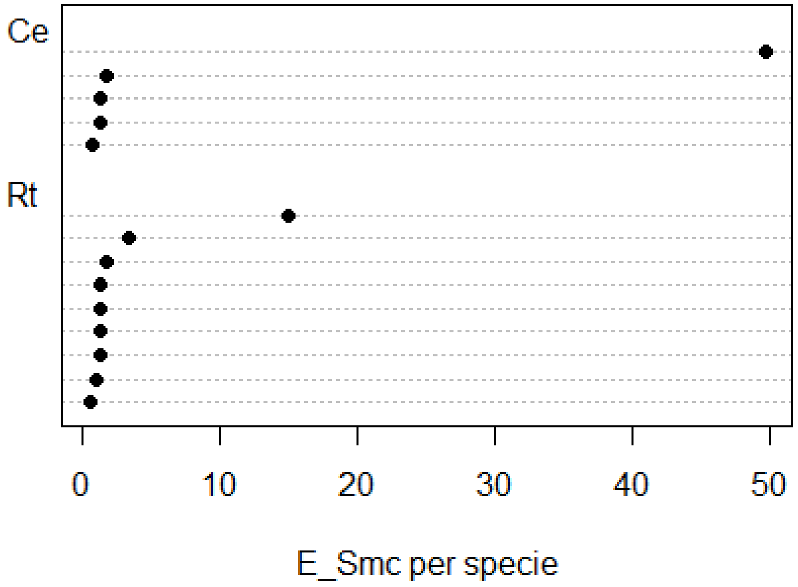


```
x <- E[order(E_H9), ]
x$specie <- factor(x$specie)
dotchart(x$HAsfc9, cex = 1, pch = 16, groups = x$specie, xlab = "E_H9 per spe
cie")
```

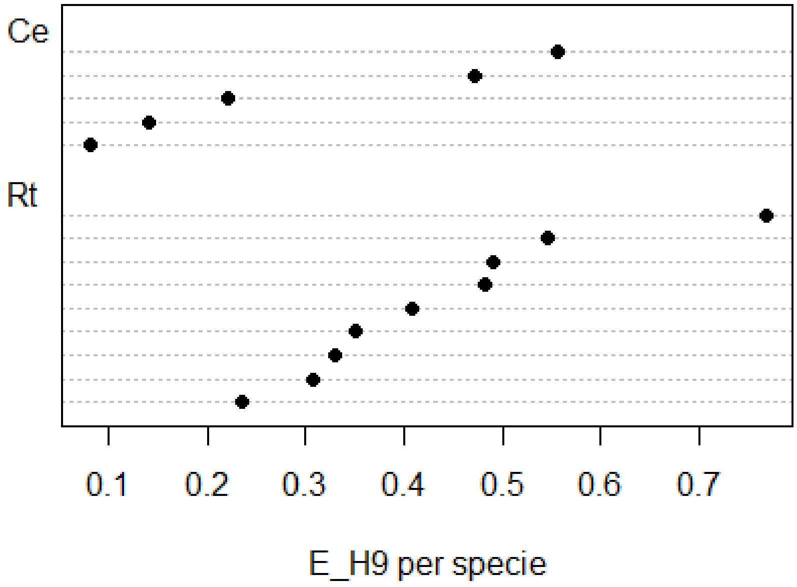


```
x <- E[order(E_H36), ]
x$specie <- factor(x$specie)
dotchart(x$HAsfc36, cex = 1, pch = 16, groups = x$specie, xlab = "E_H36 per s
pecie")
```

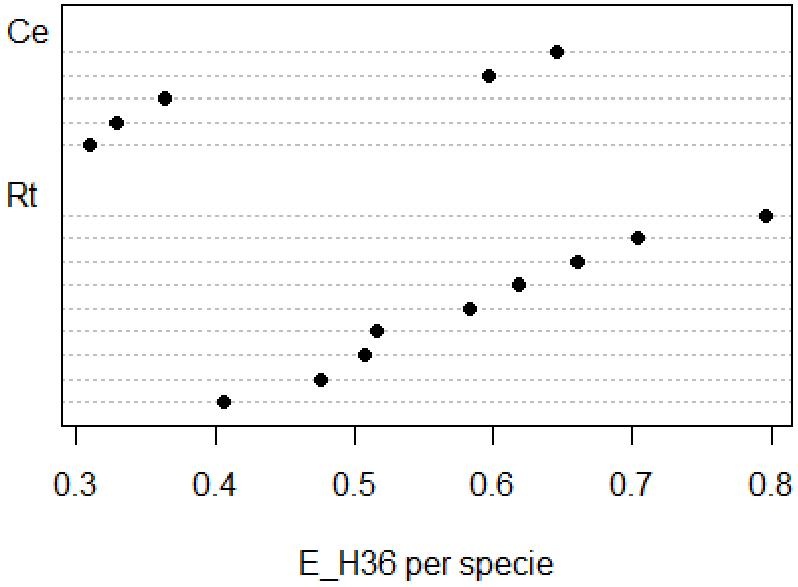


```r
x <- E[order(E_H81), ]
x$specie <- factor(x$specie)
dotchart(x$HAsfc81, cex = 1, pch = 16, groups = x$specie, xlab = "E_H81 per s
pecie")
```

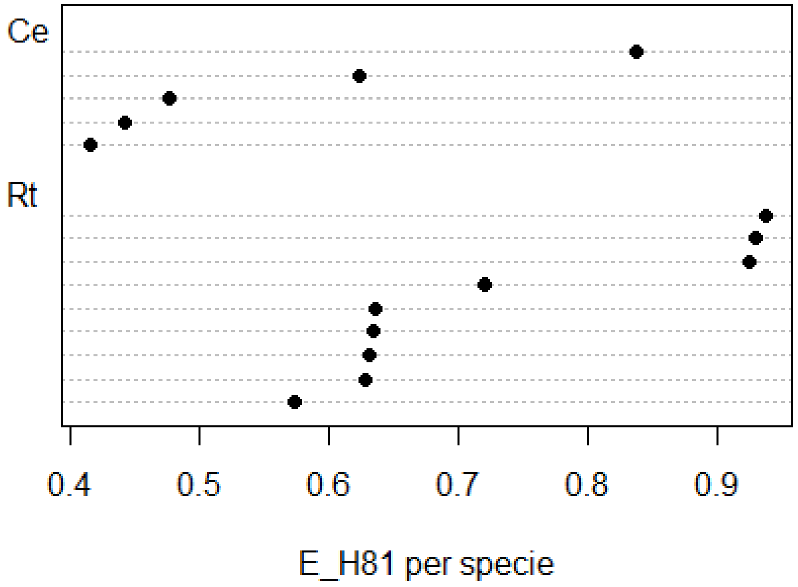

Graphical evaluation of the tests’ applicability:

Normality and homoscedasticity of the variables. #### Normality

```
ggplot(E) +
  geom_freqpoly(aes(x = Asfc), bins = 7) +
  labs(
    x = "Value Asfc",
    y = "Frequency"
  )
```

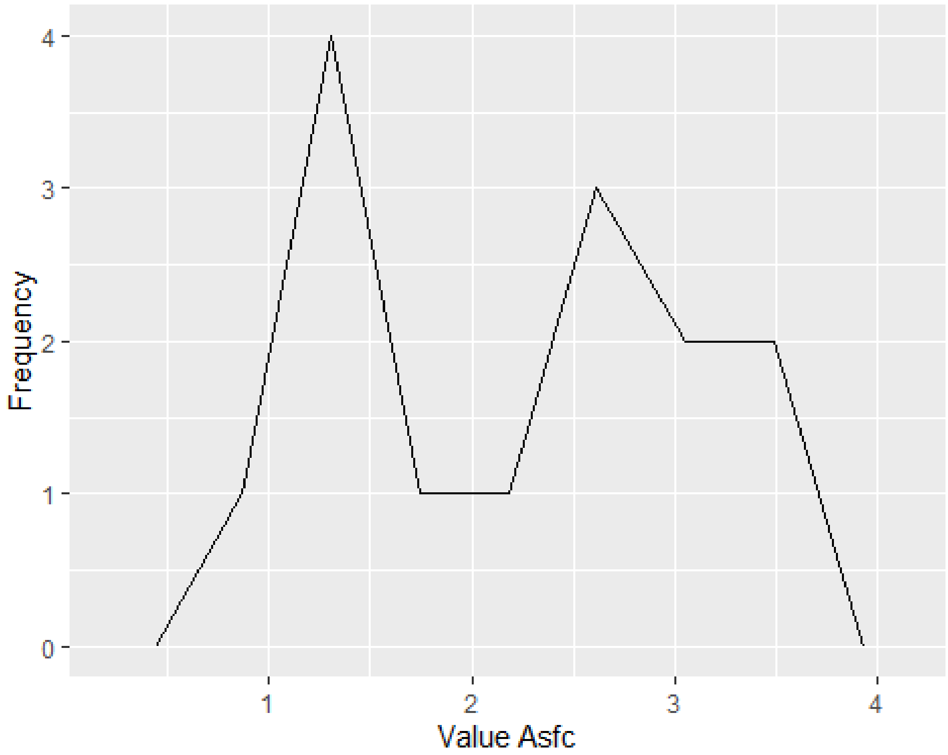


```
ggplot(E) +
  geom_freqpoly(aes(x = epLsar), bins = 7) +
  labs(
    x = "Value epLsar",
    y = "Frequency"
  )
```

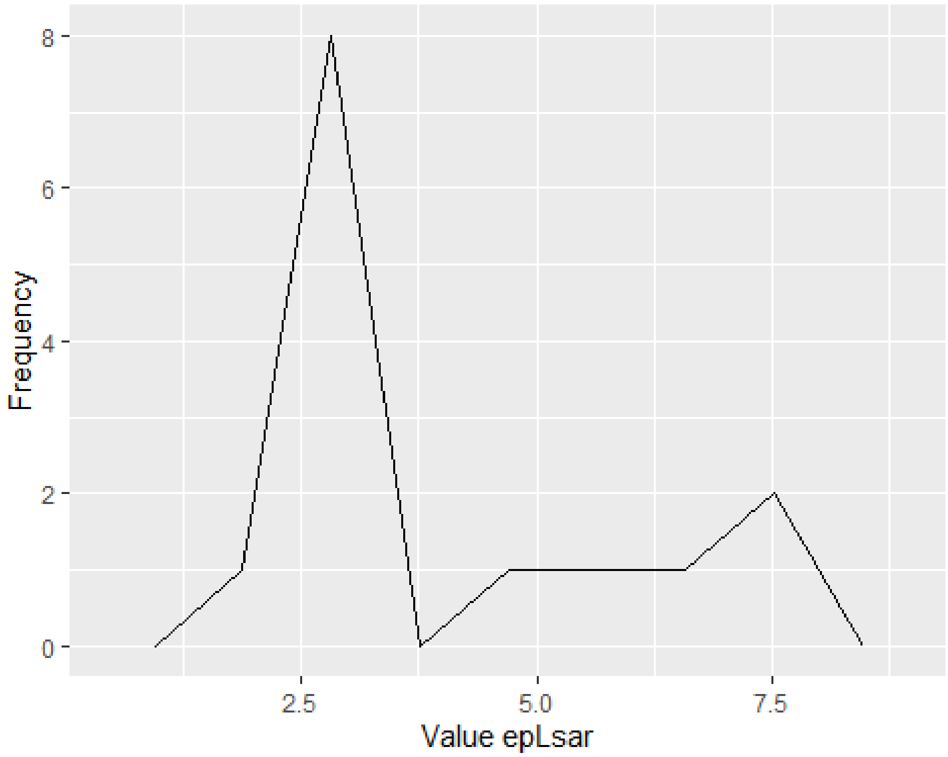


```
ggplot(E) +
  geom_freqpoly(aes(x = Smc), bins = 7) +
  labs(
    x = "Value Smc",
    y = "Frequency"
  )
```

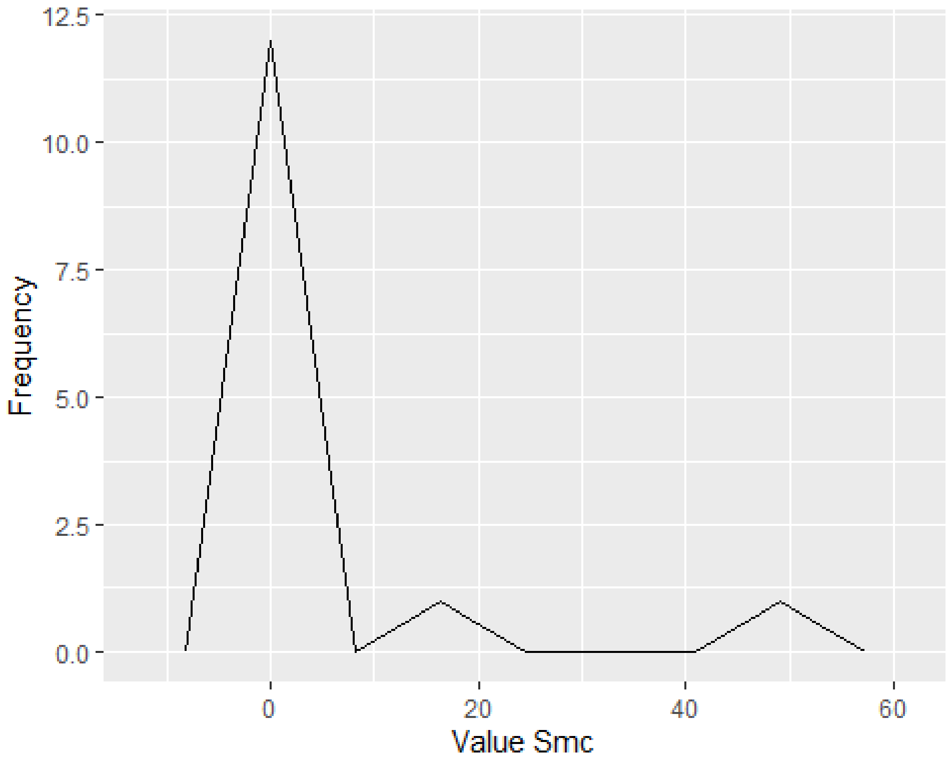


```
ggplot(E) +
  geom_freqpoly(aes(x = HAsfc9), bins = 7) +
  labs(
    x = "Value H9",
    y = "Frequency"
  )
```

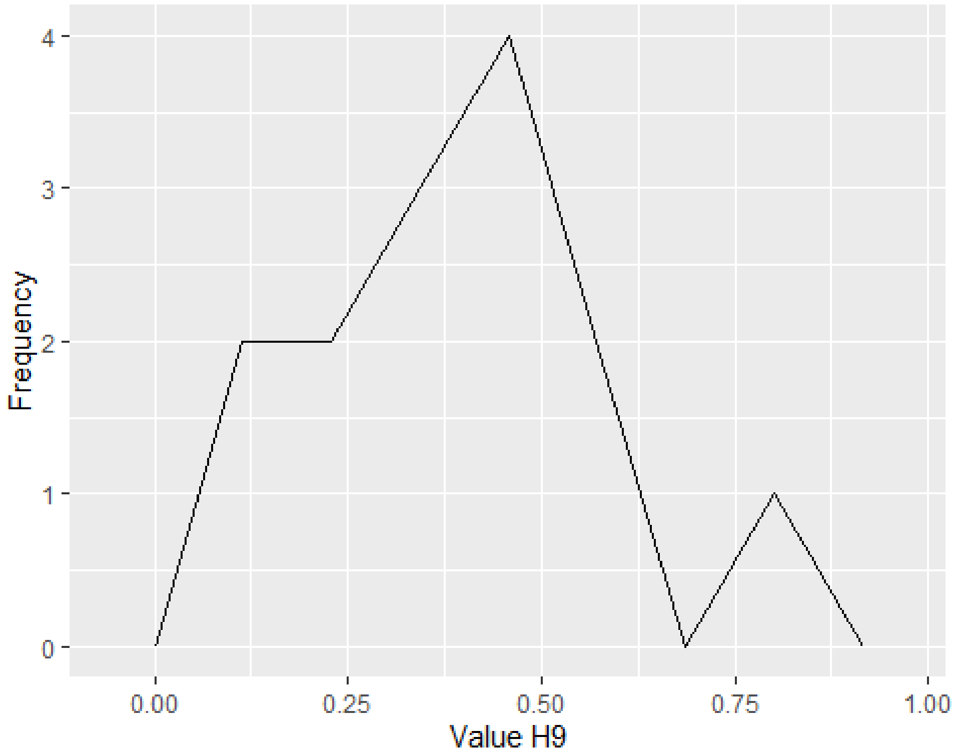


```
ggplot(E) +
  geom_freqpoly(aes(x = HAsfc36), bins = 7) +
  labs(
    x = "Value H36",
    y = "Frequency"
  )
```

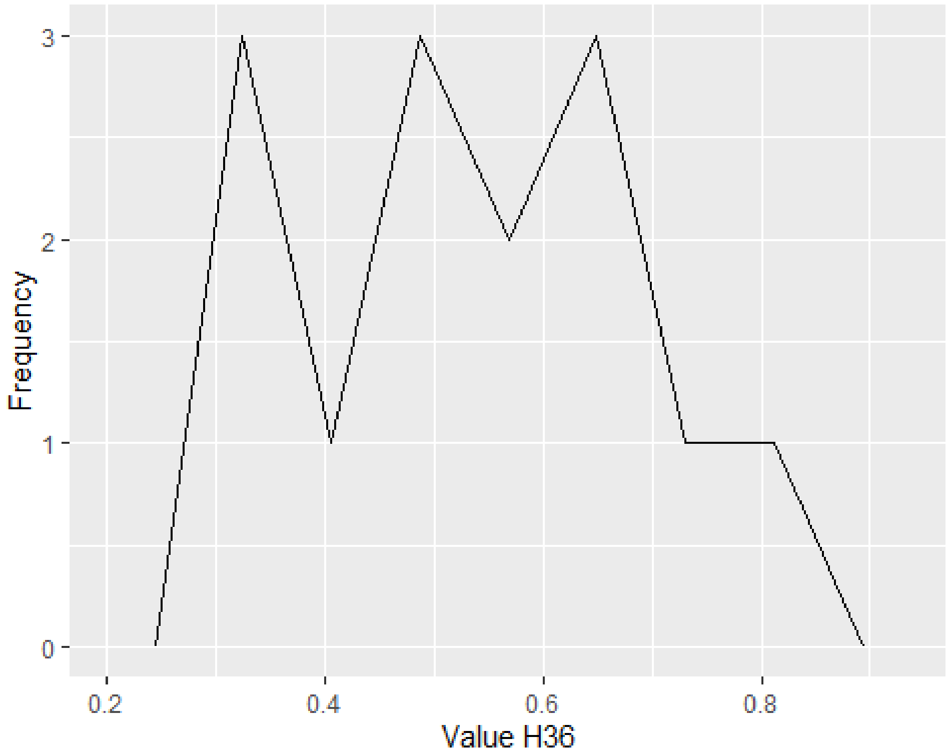


```
ggplot(E) +
  geom_freqpoly(aes(x = HAsfc81), bins = 7) +
  labs(
    x = "Value H81",
    y = "Frequency"
  )
```

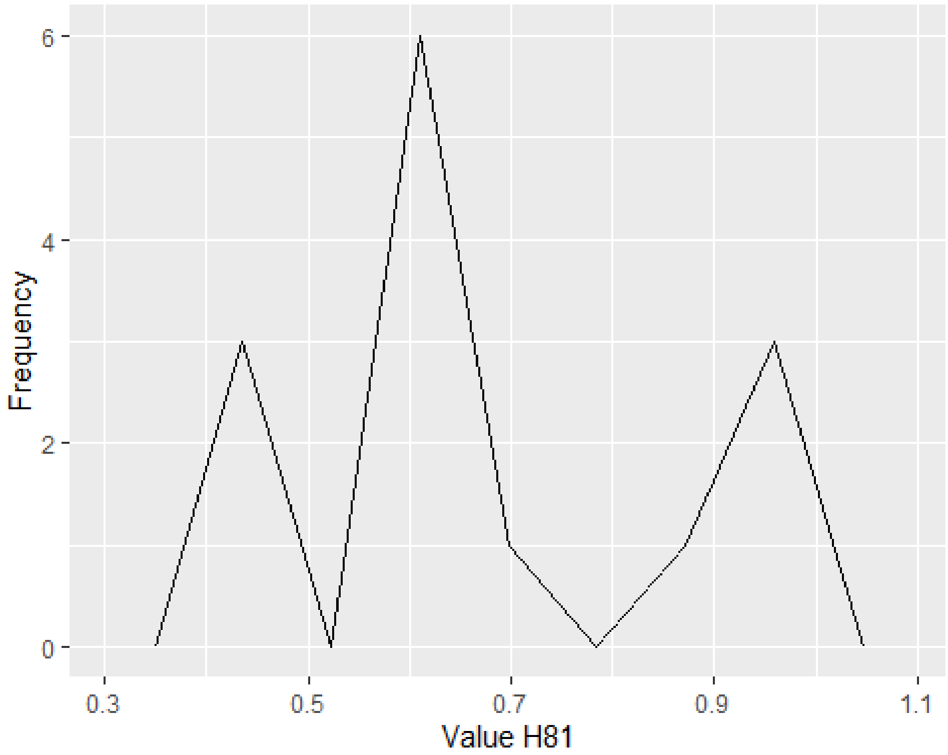

Homoscedasticity: Brown & Forsythe test (and data transformation whenever needed)

```
bf.test(E_Asfc ~ E_Species, data = E)

##
##   Brown-Forsythe Test (alpha = 0.05)
## -------------------------------------------------------------
##   data : E_Asfc and E_Species
##
##   statistic  : 1.234343
##   num df     : 1
##   denom df   : 7.374432
##   p.value    : 0.3014704
##
##   Result     : Difference is not statistically significant.
## -------------------------------------------------------------

ggplot(E) +
  geom_boxplot(aes(x = E_Species, y = E_Asfc)) +
  labs(
    x = "Species",
    y = "Asfc"
  )
```

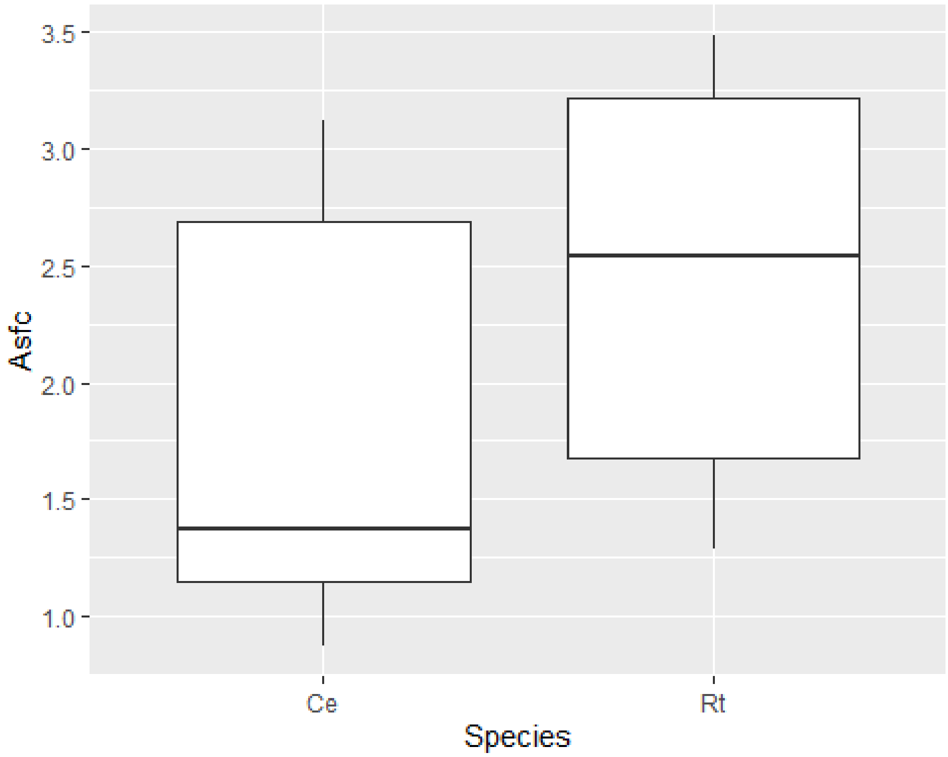


```
bf.test(E_epLsar ~ E_Species, data = E)

##
##   Brown-Forsythe Test (alpha = 0.05)
## -------------------------------------------------------------
##   data : E_epLsar and E_Species
##
##   statistic  : 0.3583321
##   num df     : 1
##   denom df   : 5.310747
##   p.value    : 0.5740537
##
##   Result     : Difference is not statistically significant.
## -------------------------------------------------------------

ggplot(E) +
  geom_boxplot(aes(x = E_Species, y = E_epLsar)) +
  labs(
    x = "Species",
    y = "epLsar"
  )
```

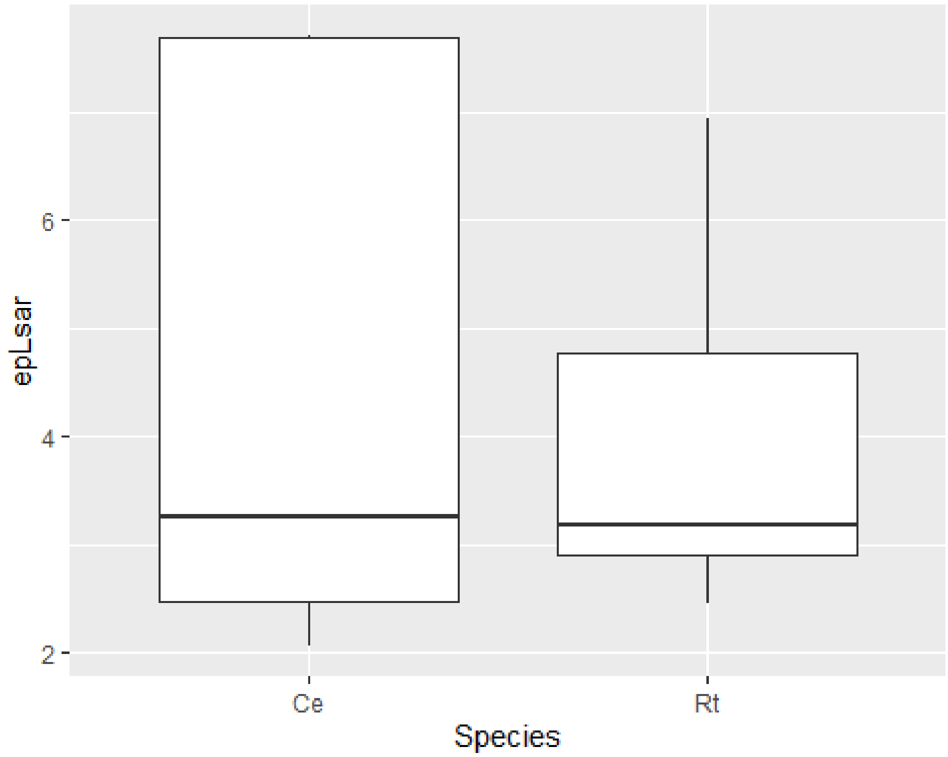


```
bf.test(E_Smc ~ E_Species, data = E)

## 
##   Brown-Forsythe Test (alpha = 0.05) 
## ------------------------------------------------------------- 
##   data : E_Smc and E_Species 
## 
##   statistic  : 0.6642335 
##   num df     : 1 
##   denom df   : 4.197756 
##   p.value    : 0.4587879 
## 
##   Result     : Difference is not statistically significant. 
## -------------------------------------------------------------

ggplot(E) +
  geom_boxplot(aes(x = E_Species, y = E_Smc)) +
  labs(
    x = "Species",
    y = "Smc"
  )
```

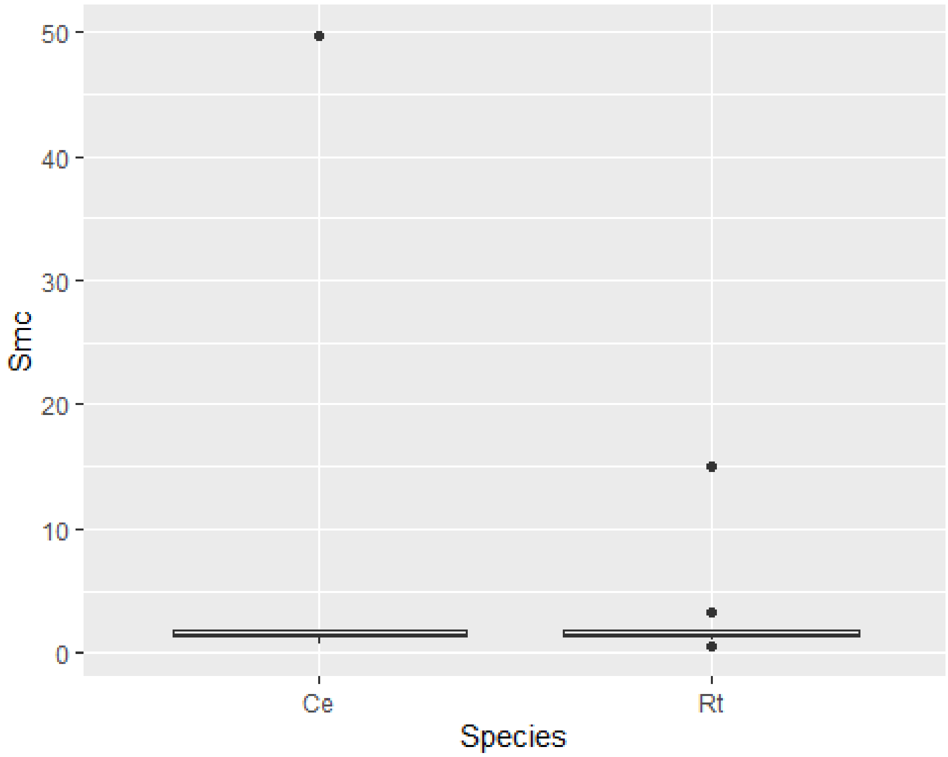


```
bf.test(E_H9 ~ E_Species, data = E)

##
##   Brown-Forsythe Test (alpha = 0.05)
## -------------------------------------------------------------
##   data : E_H9 and E_Species
##
##   statistic  : 1.74112
##   num df     : 1
##   denom df   : 6.659946
##   p.value    : 0.2305453
##
##   Result     : Difference is not statistically significant.
## -------------------------------------------------------------

ggplot(E) +
  geom_boxplot(aes(x = E_Species, y = E_H9)) +
  labs(
    x = "Species",
    y = "H9"
  )
```

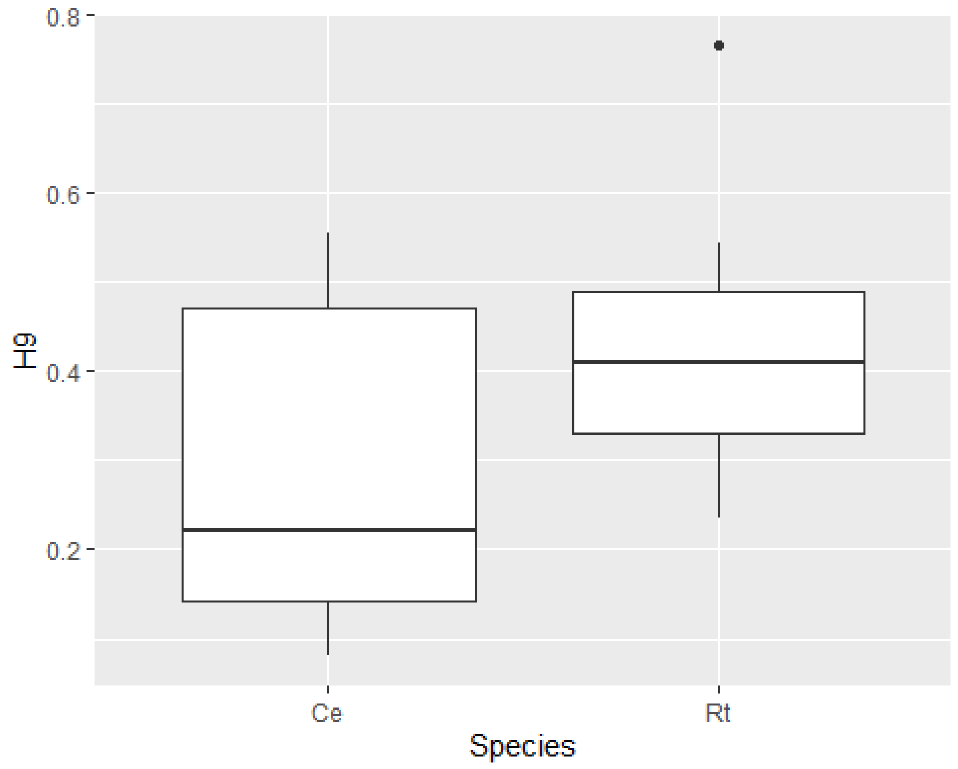


```
bf.test(E_H36 ~ E_Species, data = E)

##
##   Brown-Forsythe Test (alpha = 0.05)
## -------------------------------------------------------------
##   data : E_H36 and E_Species
##
##   statistic  : 2.757012
##   num df     : 1
##   denom df   : 6.682359
##   p.value    : 0.1428224
##
##   Result     : Difference is not statistically significant.
## -------------------------------------------------------------

ggplot(E) +
  geom_boxplot(aes(x = E_Species, y = E_H36)) +
  labs(
    x = "Species",
    y = "H36"
  )
```

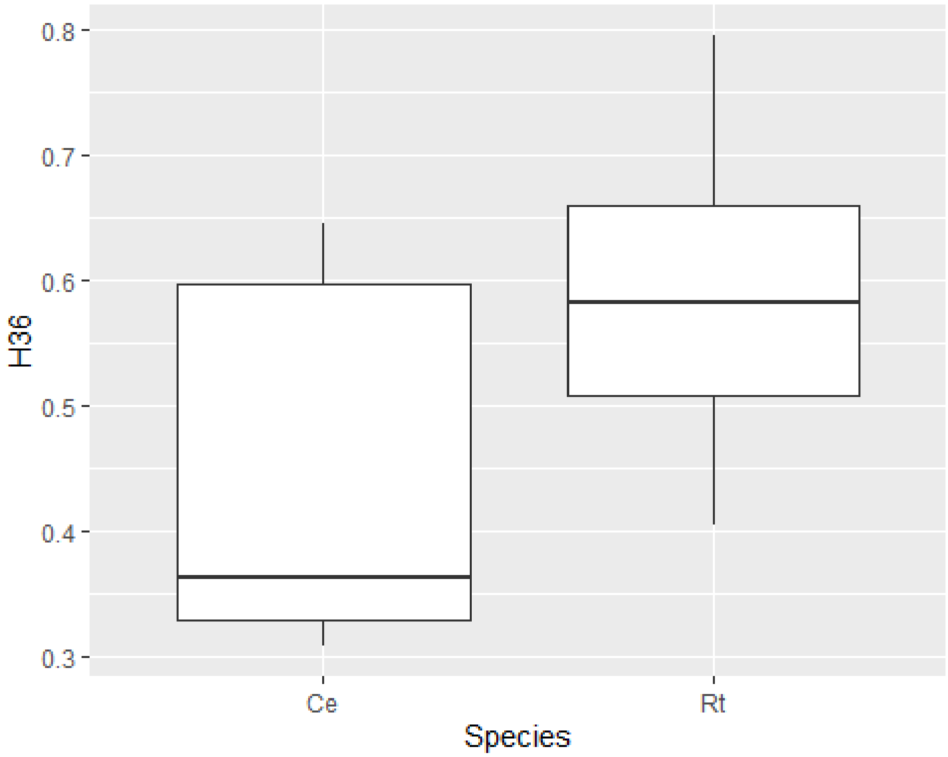


```
bf.test(E_H81 ~ E_Species, data = E)

##
##   Brown-Forsythe Test (alpha = 0.05)
## -------------------------------------------------------------
##   data : E_H81 and E_Species
##
##   statistic  : 3.579697
##   num df     : 1
##   denom df   : 7.383022
##   p.value    : 0.09820219
##
##   Result     : Difference is not statistically significant.
## -------------------------------------------------------------

ggplot(E) +
  geom_boxplot(aes(x = E_Species, y = E_H81)) +
  labs(
    x = "Species",
    y = "H81"
  )
```

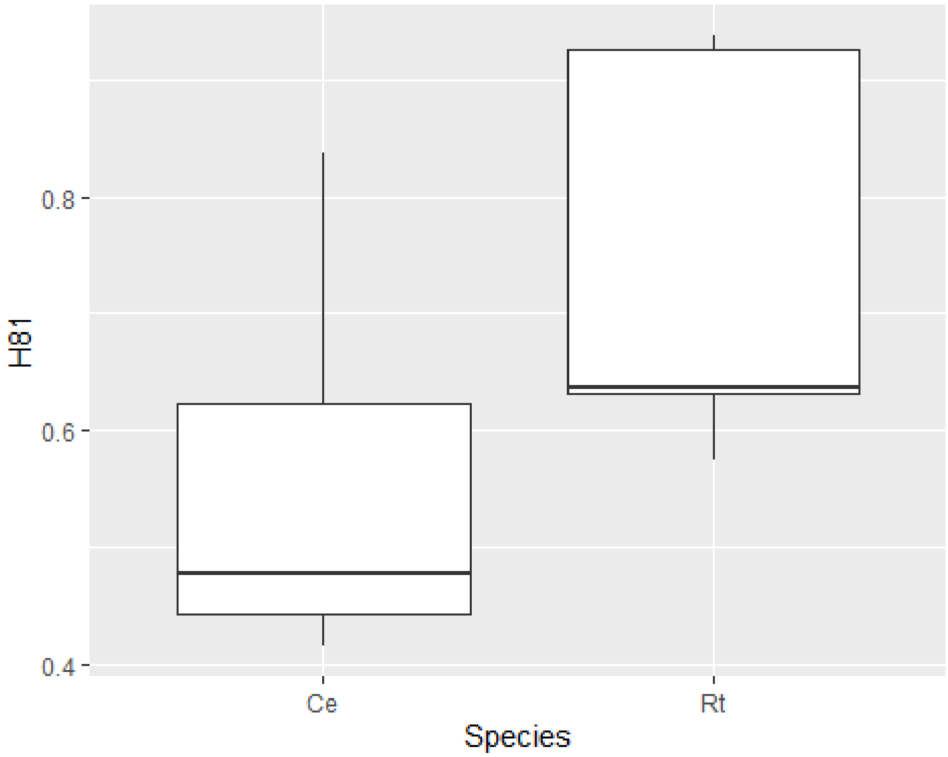

Glm: Impact of blocks over each DMTA parameter

```
glm_E_Asfc0 <- glm(E_Asfc ~ 1, data = E)
glm_E_Asfc1 <- glm(E_Asfc ~ E_Species, data = E)
Cand.models <- list()
Cand.models[[1]] <- glm_E_Asfc0
Cand.models[[2]] <- glm_E_Asfc1
Modnames <- lapply(Cand.models, "formula")
aictab(cand.set = Cand.models, modnames = paste0(Modnames), sort = TRUE)

##
## Model selection based on AICc:
##
##                      K  AICc Delta_AICc AICcWt Cum.Wt     LL
## E_Asfc ~ 1           2 41.55       0.00   0.71   0.71 -18.23
## E_Asfc ~ E_Species 3 43.36       1.82   0.29   1.00 -17.48

glm_E_epLsar0 <- glm(E_epLsar ~ 1, data = E)
glm_E_epLsar1 <- glm(E_epLsar ~ E_Species, data = E)
Cand.models <- list()
Cand.models[[1]] <- glm_E_epLsar0
Cand.models[[2]] <- glm_E_epLsar1
Modnames <- lapply(Cand.models, "formula")
aictab(cand.set = Cand.models, modnames = paste0(Modnames), sort = TRUE)

##
## Model selection based on AICc:
##
```

```
##                          K  AICc Delta_AICc AICcWt Cum.Wt     LL
## E_epLsar ~ 1          2 63.25       0.00    0.8    0.8 -29.08
## E_epLsar ~ E_Species 3 65.98       2.73    0.2    1.0 -28.79

glm_E_Smc0 <- glm(E_Smc ~ 1, data = E)
glm_E_Smc1 <- glm(E_Smc ~ E_Species, data = E)
Cand.models <- list()
Cand.models[[1]] <- glm_E_Smc0
Cand.models[[2]] <- glm_E_Smc1
Modnames <- lapply(Cand.models, "formula")
aictab(cand.set = Cand.models, modnames = paste0(Modnames), sort = TRUE)

##
## Model selection based on AICc:
##
##                       K   AICc Delta_AICc AICcWt Cum.Wt     LL
## E_Smc ~ 1          2 115.90       0.00   0.73   0.73 -55.40
## E_Smc ~ E_Species 3 117.87       1.97   0.27   1.00 -54.73

glm_E_H9_0 <- glm(E_H9 ~ 1, data = E)
glm_E_H9_1 <- glm(E_H9 ~ E_Species, data = E)
Cand.models <- list()
Cand.models[[1]] <- glm_E_H9_0
Cand.models[[2]] <- glm_E_H9_1
Modnames <- lapply(Cand.models, "formula")
aictab(cand.set = Cand.models, modnames = paste0(Modnames), sort = TRUE)

##
## Model selection based on AICc:
##
##                      K  AICc Delta_AICc AICcWt Cum.Wt   LL
## E_H9 ~ 1          2 -3.62        0.0   0.63   0.63 4.35
## E_H9 ~ E_Species 3 -2.52        1.1   0.37   1.00 5.46

glm_E_H36_0 <- glm(E_H36 ~ 1, data = E)
glm_E_H36_1 <- glm(E_H36 ~ E_Species, data = E)
Cand.models <- list()
Cand.models[[1]] <- glm_E_H36_0
Cand.models[[2]] <- glm_E_H36_1
Modnames <- lapply(Cand.models, "formula")
aictab(cand.set = Cand.models, modnames = paste0(Modnames), sort = TRUE)

##
## Model selection based on AICc:
##
##                       K  AICc Delta_AICc AICcWt Cum.Wt   LL
## E_H36 ~ E_Species 3 -9.88       0.00    0.5    0.5 9.14
## E_H36 ~ 1          2 -9.84       0.04    0.5    1.0 7.47

summary(glm_E_H36_1)
```

```
##
## Call:
## glm(formula = E_H36 ~ E_Species, data = E)
##
## Deviance Residuals:
##      Min        1Q    Median        3Q       Max
## -0.17944  -0.10353  -0.03544   0.10781   0.21056
##
## Coefficients:
##             Estimate Std. Error t value Pr(>|t|)
## (Intercept)  0.44880    0.06085   7.376 8.55e-06 ***
## E_SpeciesRt  0.13664    0.07589   1.801   0.0969 .
## ---
## Signif. codes:  0 '***' 0.001 '**' 0.01 '*' 0.05 '.' 0.1 ' ' 1
##
## (Dispersion parameter for gaussian family taken to be 0.01851109)
##
##     Null deviance: 0.28215  on 13  degrees of freedom
## Residual deviance: 0.22213  on 12  degrees of freedom
## AIC: -12.279
##
## Number of Fisher Scoring iterations: 2
```

```
marginal <- emmeans(glm_E_H36_1, ~E_Species)
pairs(marginal)
```

```
##  contrast estimate     SE df t.ratio p.value
##  Ce - Rt    -0.137 0.0759 12  -1.801  0.0969
```

```
glm_E_H81_0 <- glm(E_H81 ~ 1, data = E)
glm_E_H81_1 <- glm(E_H81 ~ E_Species, data = E)
Cand.models <- list()
Cand.models[[1]] <- glm_E_H81_0
Cand.models[[2]] <- glm_E_H81_1
Modnames <- lapply(Cand.models, "formula")
aictab(cand.set = Cand.models, modnames = paste0(Modnames), sort = TRUE)
```

```
##
## Model selection based on AICc:
##
##                    K  AICc Delta_AICc AICcWt Cum.Wt   LL
## E_H81 ~ E_Species 3 -5.35       0.00   0.58   0.58 6.88
## E_H81 ~ 1         2 -4.71       0.64   0.42   1.00 4.90
```

```
summary(glm_E_H81_1)
```

```
##
## Call:
## glm(formula = E_H81 ~ E_Species, data = E)
##
## Deviance Residuals:
```

```
##      Min        1Q    Median        3Q       Max
## -0.16167  -0.10617  -0.09093   0.15870   0.27880
##
## Coefficients:
##             Estimate Std. Error t value Pr(>|t|)
## (Intercept)  0.55920    0.07152   7.819 4.75e-06 ***
## E_SpeciesRt  0.17647    0.08920   1.978   0.0713 .
## ---
## Signif. codes:  0 '***' 0.001 '**' 0.01 '*' 0.05 '.' 0.1 ' ' 1
##
## (Dispersion parameter for gaussian family taken to be 0.02557707)
##
##     Null deviance: 0.40702  on 13  degrees of freedom
## Residual deviance: 0.30692  on 12  degrees of freedom
## AIC: -7.7527
##
## Number of Fisher Scoring iterations: 2
```

```
marginal <- emmeans(glm_E_H81_1, ~E_Species)
pairs(marginal)
```

```
##  contrast estimate     SE df t.ratio p.value
##  Ce - Rt    -0.176 0.0892 12  -1.978  0.0713
```

Block F

```
F_Species <- F %>%
  dplyr::select(c(2)) %>%
  unlist(c(1))

F_Asfc <- F %>%
  dplyr::select(c(7)) %>%
  unlist(c(1))
F_epLsar <- F %>%
  dplyr::select(c(8)) %>%
  unlist(c(1))
F_Smc <- F %>%
  dplyr::select(c(9)) %>%
  unlist(c(1))
F_H9 <- F %>%
  dplyr::select(c(10)) %>%
  unlist(c(1))
F_H36 <- F %>%
  dplyr::select(c(12)) %>%
  unlist(c(1))
F_H81 <- F %>%
  dplyr::select(c(11)) %>%
  unlist(c(1))
```

Checking data distribution and outliers:

```
x <- F[order(F_Asfc), ]
x$specie <- factor(x$specie)
```

```
dotchart(x$Asfc, cex = 1, pch = 16, groups = x$specie, xlab = "F_Asfc per spe
cie")
```

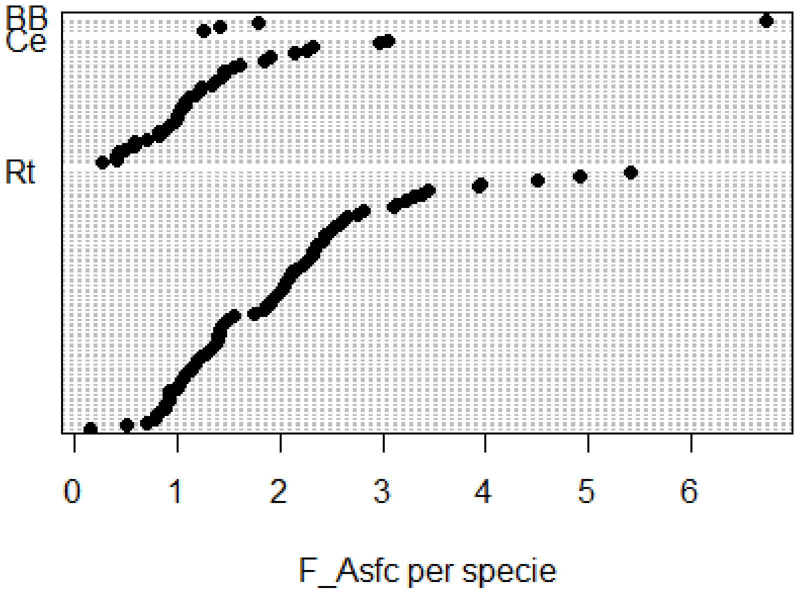


```
x <- F[order(F_epLsar), ]
x$specie <- factor(x$specie)
dotchart(x$epLsar, cex = 1, pch = 16, groups = x$specie, xlab = "F_epLsar per
specie")
```

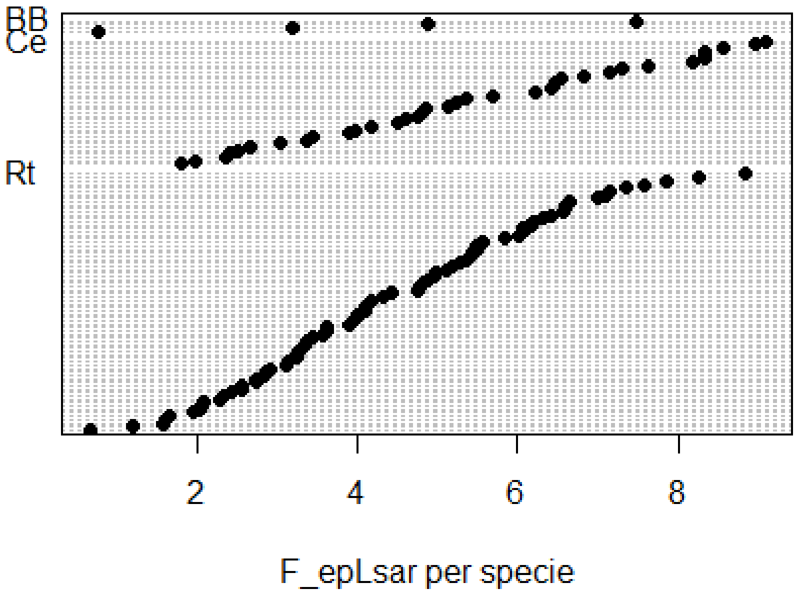


```
x <- F[order(F_Smc), ]
x$specie <- factor(x$specie)
dotchart(x$Smc, cex = 1, pch = 16, groups = x$specie, xlab = "F_Smc per speci
e")
```

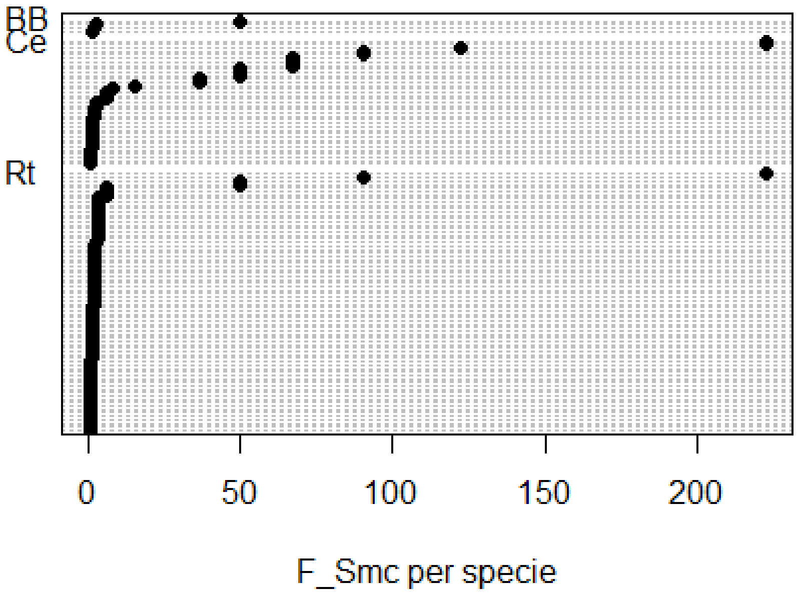


```
x <- F[order(F_H9), ]
x$specie <- factor(x$specie)
dotchart(x$HAsfc9, cex = 1, pch = 16, groups = x$specie, xlab = "F_H9 per spe
cie")
```

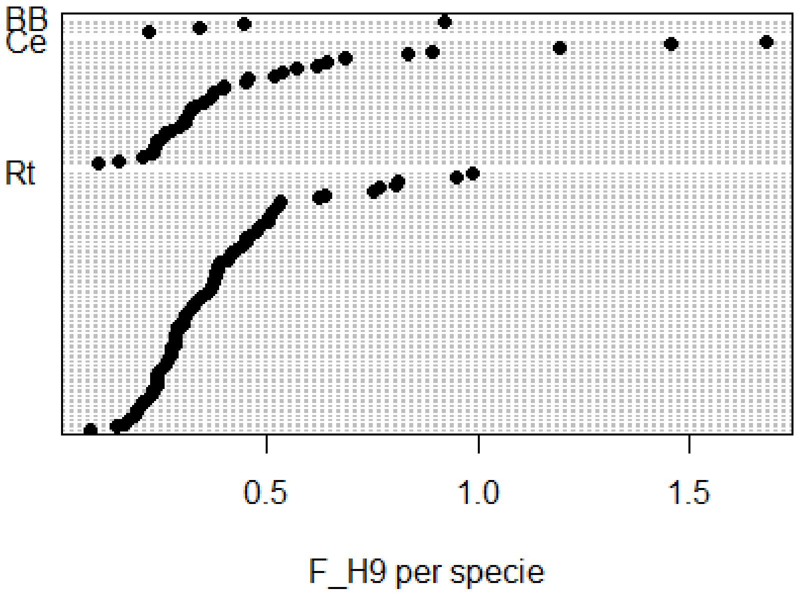


```
x <- F[order(F_H36), ]
x$specie <- factor(x$specie)
dotchart(x$HAsfc36, cex = 1, pch = 16, groups = x$specie, xlab = "F_H36 per s
pecie")
```

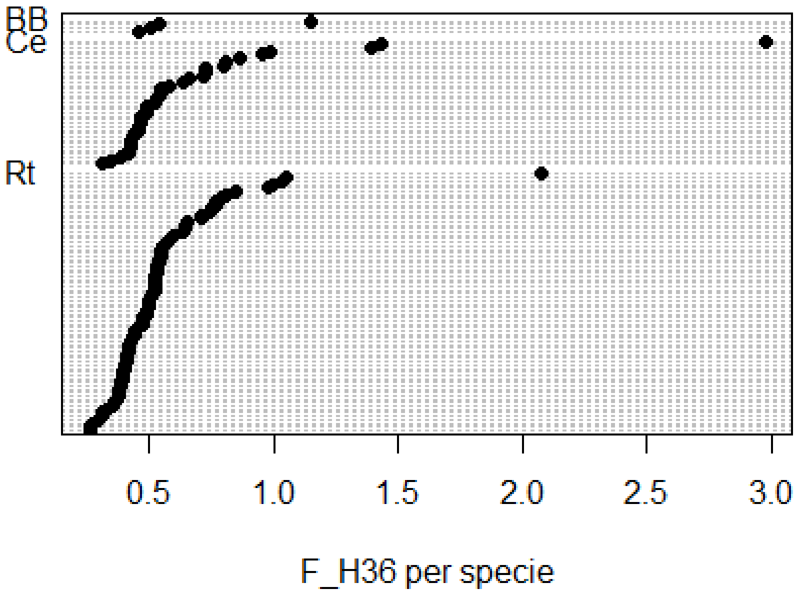


```
x <- F[order(F_H81), ]
x$specie <- factor(x$specie)
dotchart(x$HAsfc81, cex = 1, pch = 16, groups = x$specie, xlab = "F_H81 per s
pecie")
```

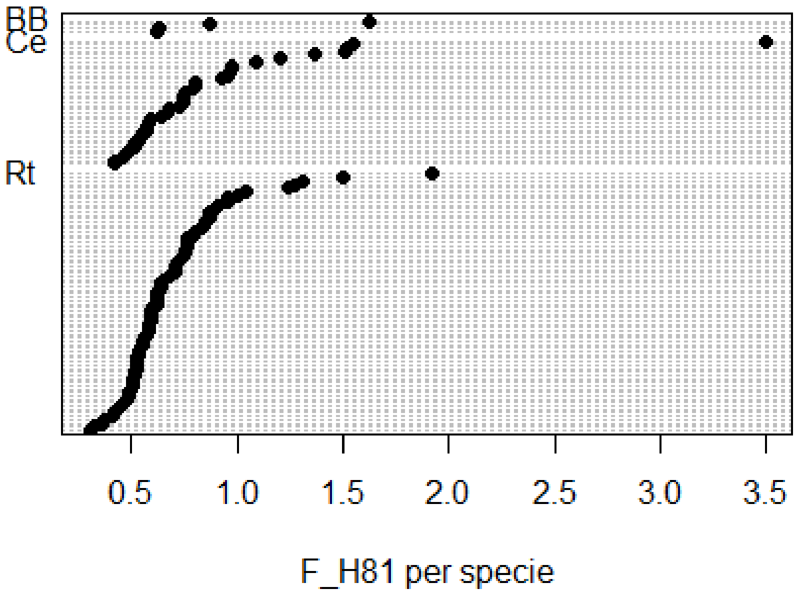

Graphical evaluation of the tests' applicability:

Normality and homoscedasticity of the variables. #### Normality

```
ggplot(F) +
  geom_freqpoly(aes(x = Asfc), bins = 7) +
  labs(
    x = "Value Asfc",
    y = "Frequency"
  )
```

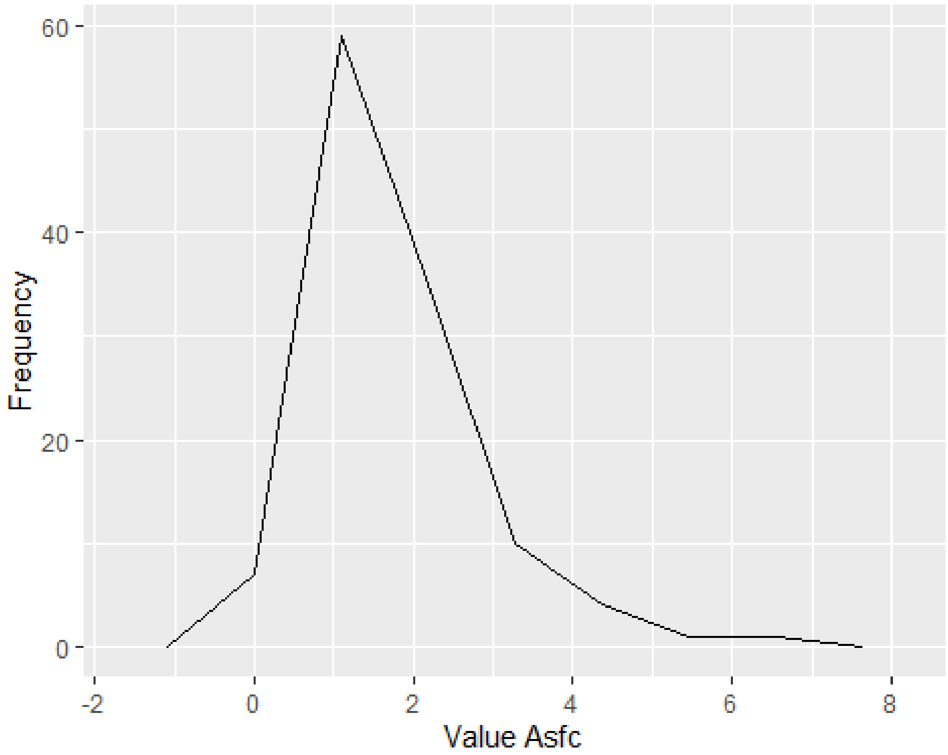


```
ggplot(F) +
  geom_freqpoly(aes(x = epLsar), bins = 7) +
  labs(
    x = "Value epLsar",
    y = "Frequency"
  )
```

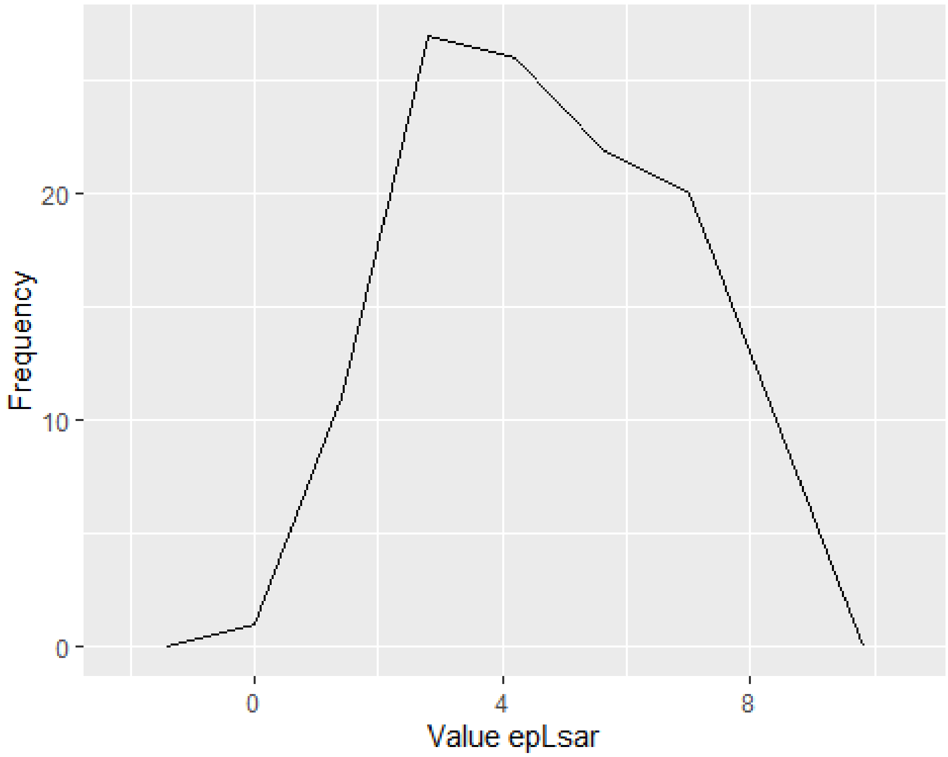


```
ggplot(F) +
  geom_freqpoly(aes(x = Smc), bins = 7) +
  labs(
    x = "Value Smc",
    y = "Frequency"
  )
```

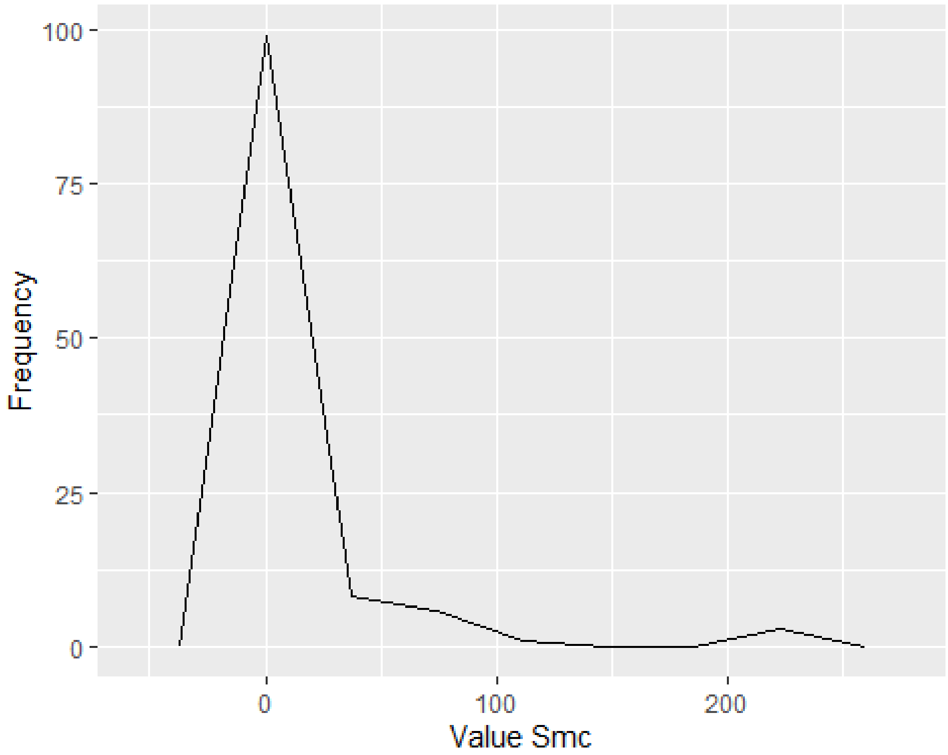


```
ggplot(F) +
  geom_freqpoly(aes(x = HAsfc9), bins = 7) +
  labs(
    x = "Value H9",
    y = "Frequency"
  )
```

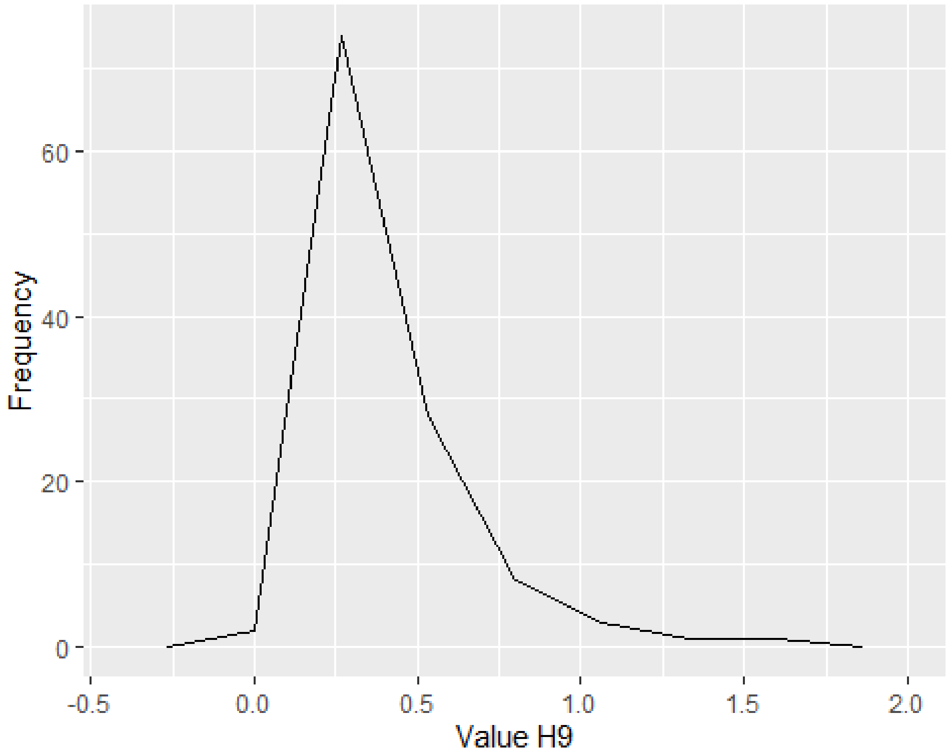


```
ggplot(F) +
  geom_freqpoly(aes(x = HAsfc36), bins = 7) +
  labs(
    x = "Value H36",
    y = "Frequency"
  )
```

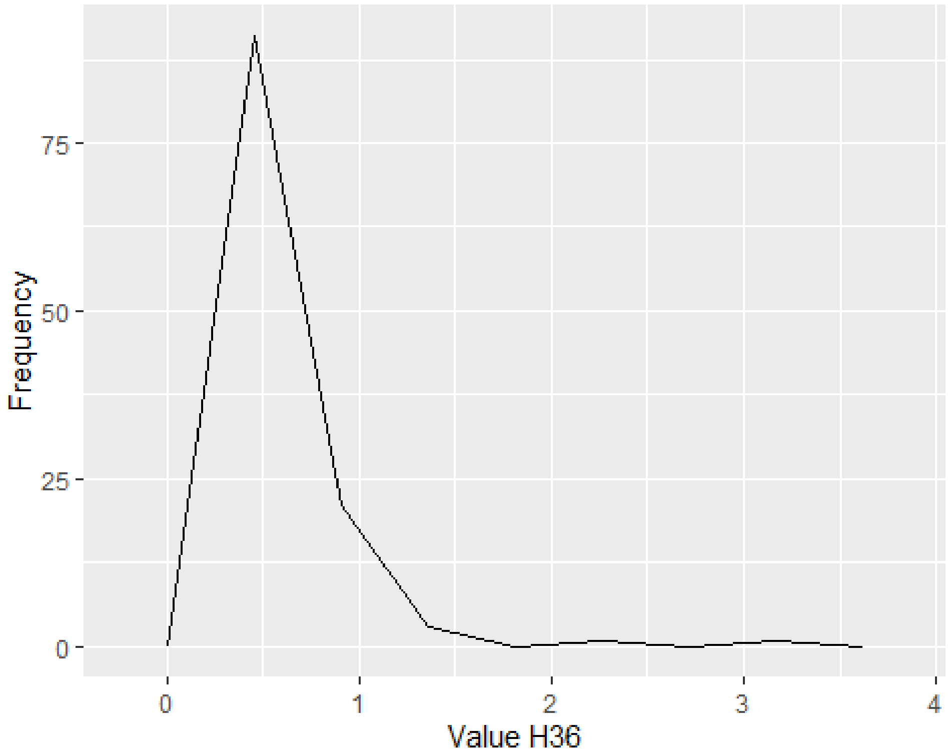


```
ggplot(F) +
  geom_freqpoly(aes(x = HAsfc81), bins = 7) +
  labs(
    x = "Value H81",
    y = "Frequency"
  )
```

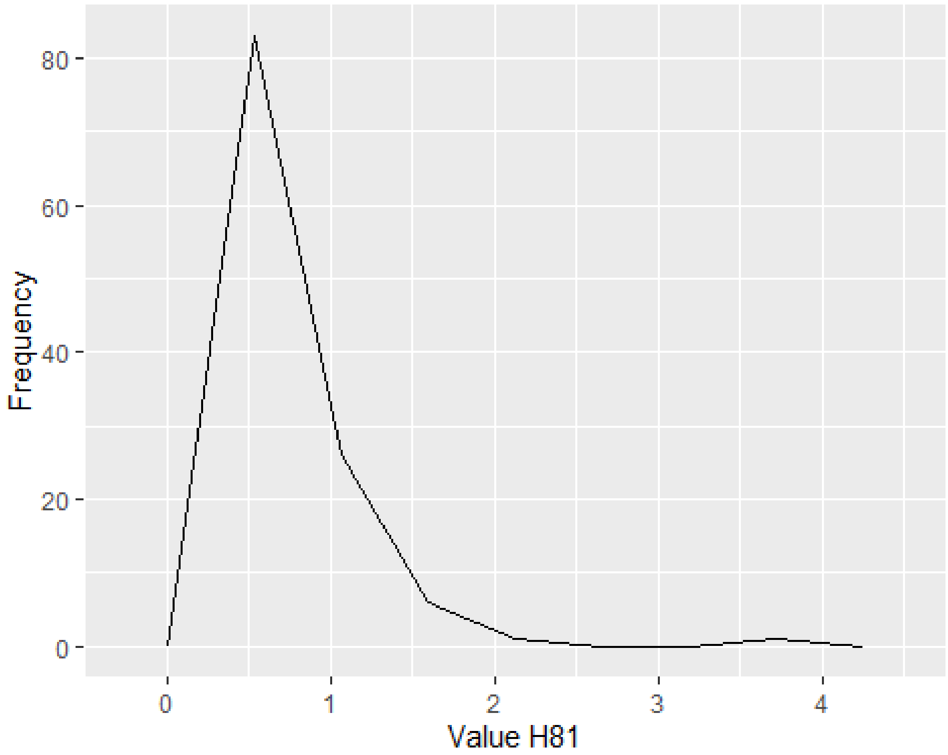

Homoscedasticity: Brown & Forsythe test (and data transformation whenever needed)

```
bf.test(F_Asfc ~ F_Species, data = F)

##
##   Brown-Forsythe Test (alpha = 0.05)
## -------------------------------------------------------------
##   data : F_Asfc and F_Species
##
##   statistic  : 2.367499
##   num df     : 2
##   denom df   : 3.636071
##   p.value    : 0.2195841
##
##   Result     : Difference is not statistically significant.
## -------------------------------------------------------------

ggplot(F) +
  geom_boxplot(aes(x = F_Species, y = F_Asfc)) +
  labs(
    x = "Species",
    y = "Asfc"
  )
```

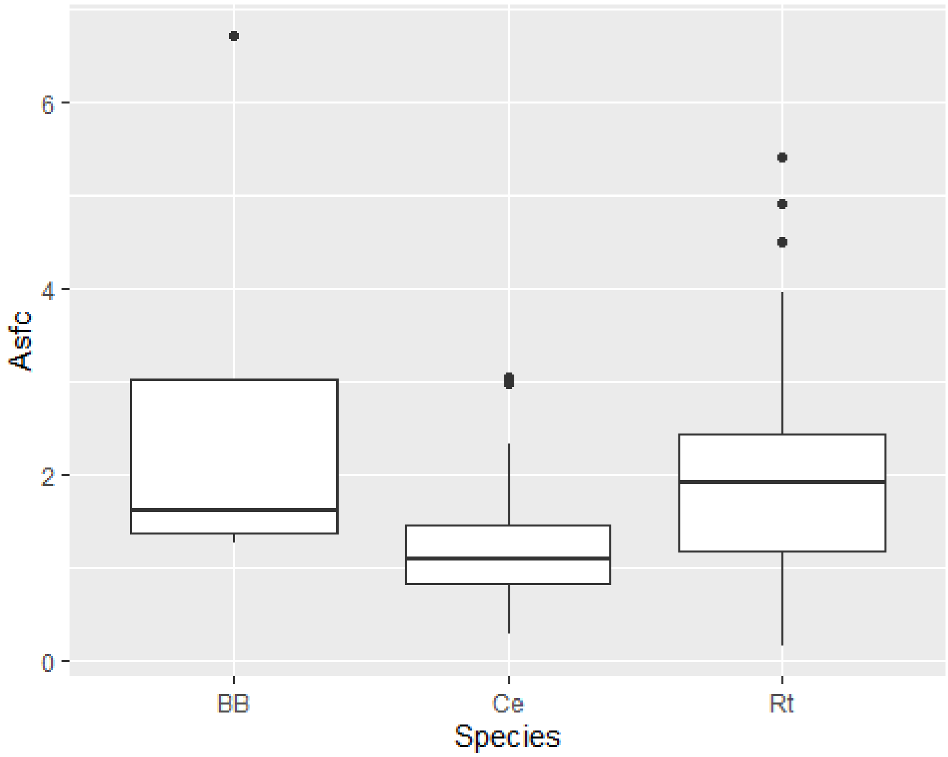


```
bf.test(F_epLsar ~ F_Species, data = F)

##
##   Brown-Forsythe Test (alpha = 0.05)
## -------------------------------------------------------------
##   data : F_epLsar and F_Species
##
##   statistic  : 2.264441
##   num df     : 2
##   denom df   : 7.341448
##   p.value    : 0.1713915
##
##   Result     : Difference is not statistically significant.
## -------------------------------------------------------------

ggplot(F) +
  geom_boxplot(aes(x = F_Species, y = F_epLsar)) +
  labs(
    x = "Species",
    y = "epLsar"
  )
```

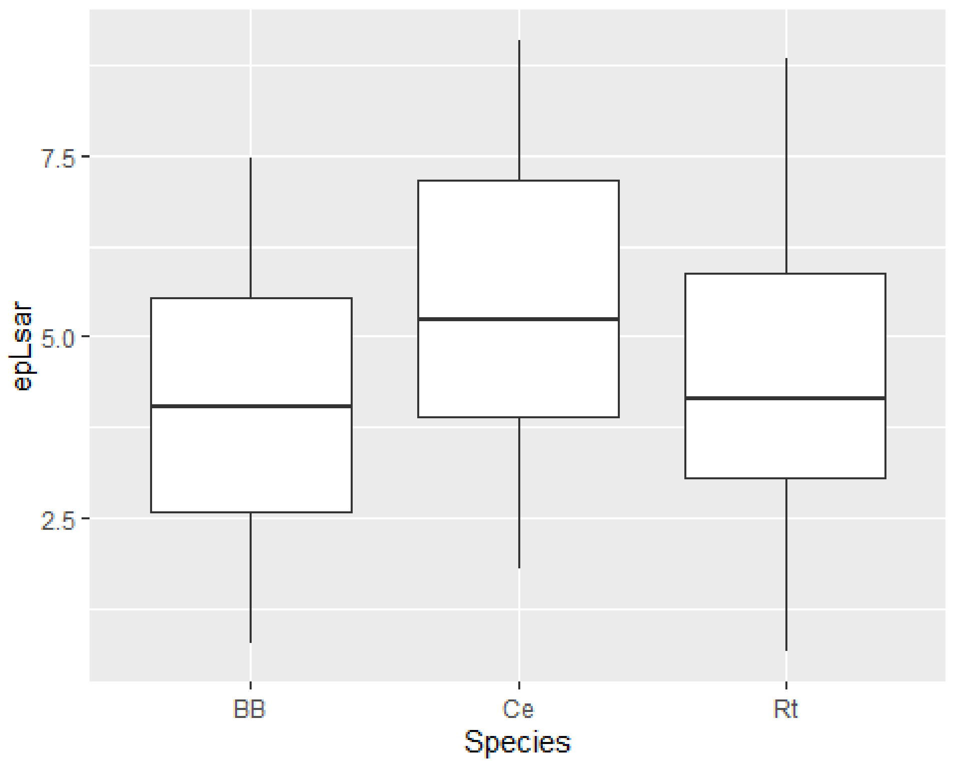


```
bf.test(F_Smc ~ F_Species, data = F)

##
##   Brown-Forsythe Test (alpha = 0.05)
## -------------------------------------------------------------
##   data : F_Smc and F_Species
##
##   statistic  : 5.816922
##   num df     : 2
##   denom df   : 38.34597
##   p.value    : 0.006218285
##
##   Result     : Difference is statistically significant.
## -------------------------------------------------------------

ggplot(F) +
  geom_boxplot(aes(x = F_Species, y = F_Smc)) +
  labs(
    x = "Species",
    y = "Smc"
  )
```

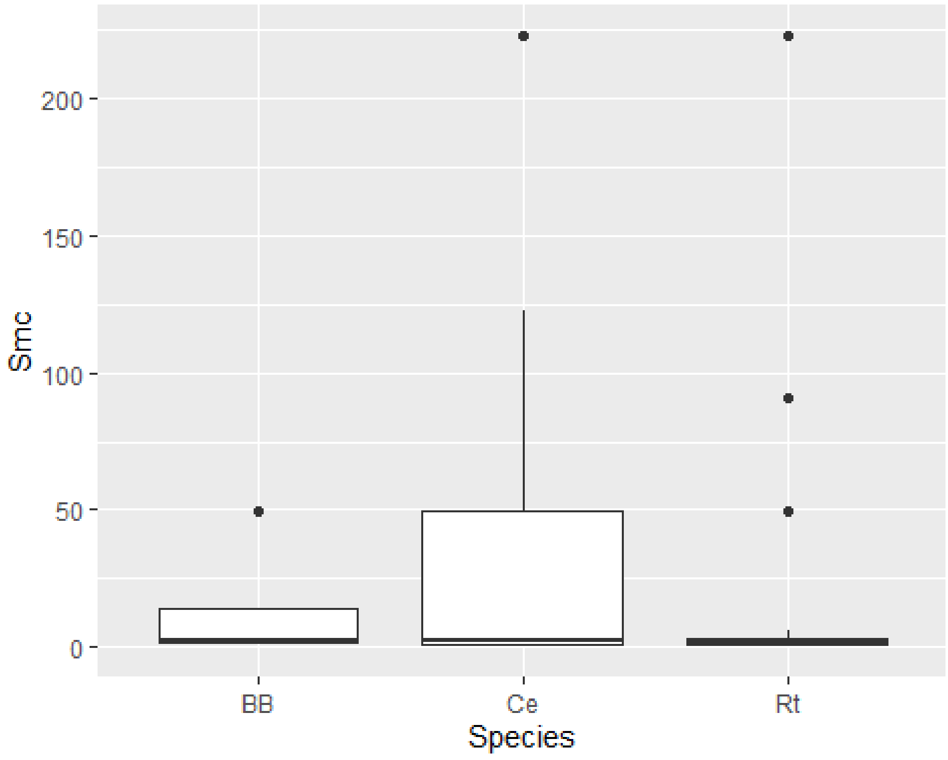


```
GLM_F_Smc <- glm(formula = F_Smc ~ F_Species, family = gaussian)
bc_F_Smc <- boxcox(GLM_F_Smc, lambda = seq(-3, 3))
```

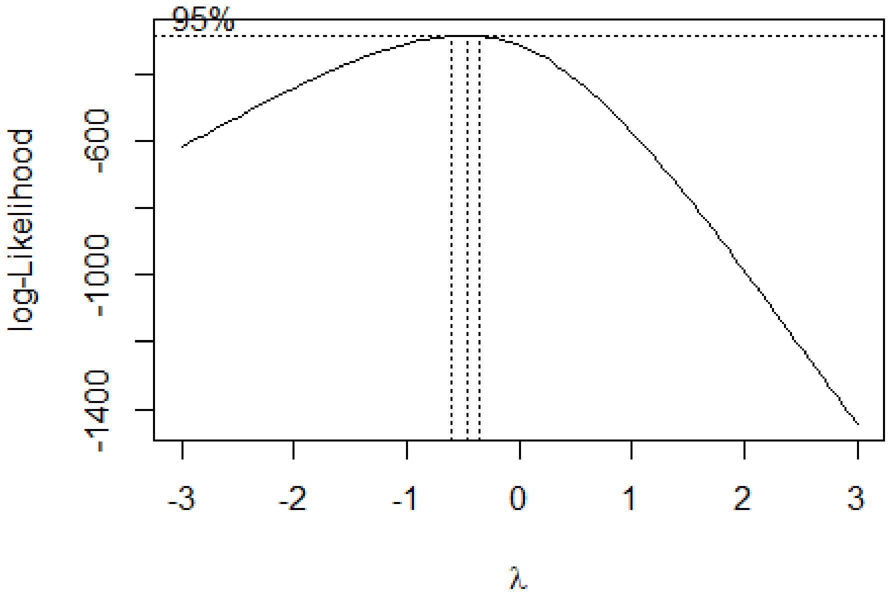

```
best.lam <- bc_F_Smc$x[which(bc_F_Smc$y == max(bc_F_Smc$y))]
best.lam # -0.4545455

## [1] -0.4545455

F_Smc <- (F_Smc)^-0.4545455
bf.test(F_Smc ~ F_Species, data = F) # the variances remain significantly dif
ferent

##
##   Brown-Forsythe Test (alpha = 0.05)
## -------------------------------------------------------------
##   data : F_Smc and F_Species
##
##   statistic  : 8.495979
##   num df     : 2
##   denom df   : 13.39166
##   p.value    : 0.004145622
##
##   Result     : Difference is statistically significant.
## -------------------------------------------------------------

ggplot(F) +
  geom_boxplot(aes(x = F_Species, y = F_Smc)) +
  labs(
    x = "F",
    y = "Smc"
  )
```

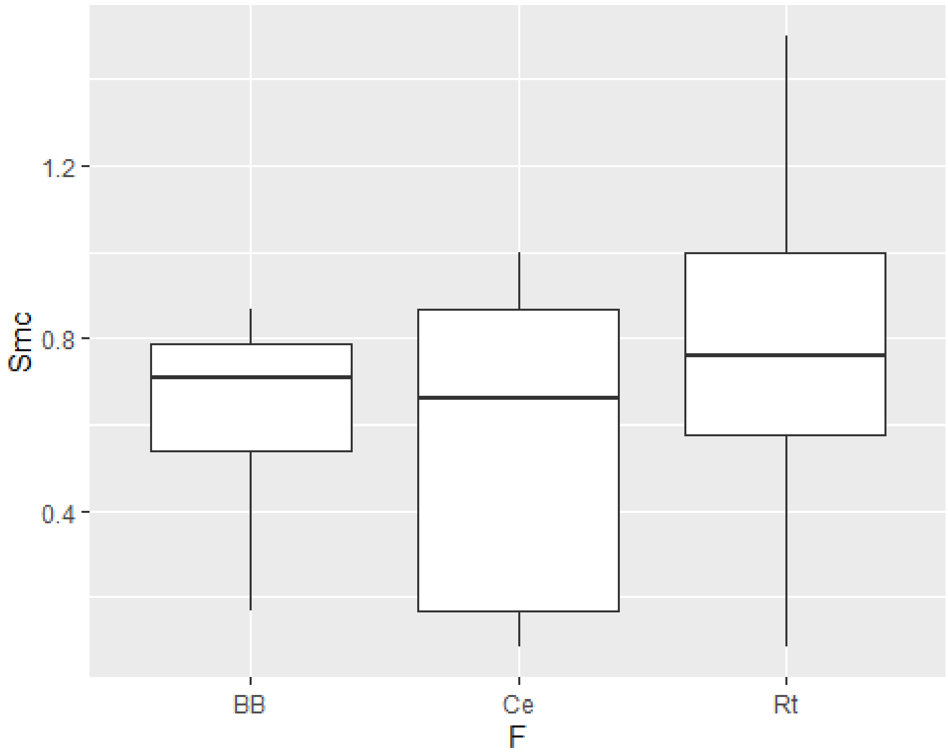

```
bf.test(F_H9 ~ F_Species, data = F)

##
##   Brown-Forsythe Test (alpha = 0.05)
## -------------------------------------------------------------
##   data : F_H9 and F_Species
##
##   statistic  : 1.275895
##   num df     : 2
##   denom df   : 11.55787
##   p.value    : 0.3157332
##
##   Result     : Difference is not statistically significant.
## -------------------------------------------------------------

ggplot(F) +
  geom_boxplot(aes(x = F_Species, y = F_H9)) +
  labs(
    x = "Species",
    y = "H9"
  )
```

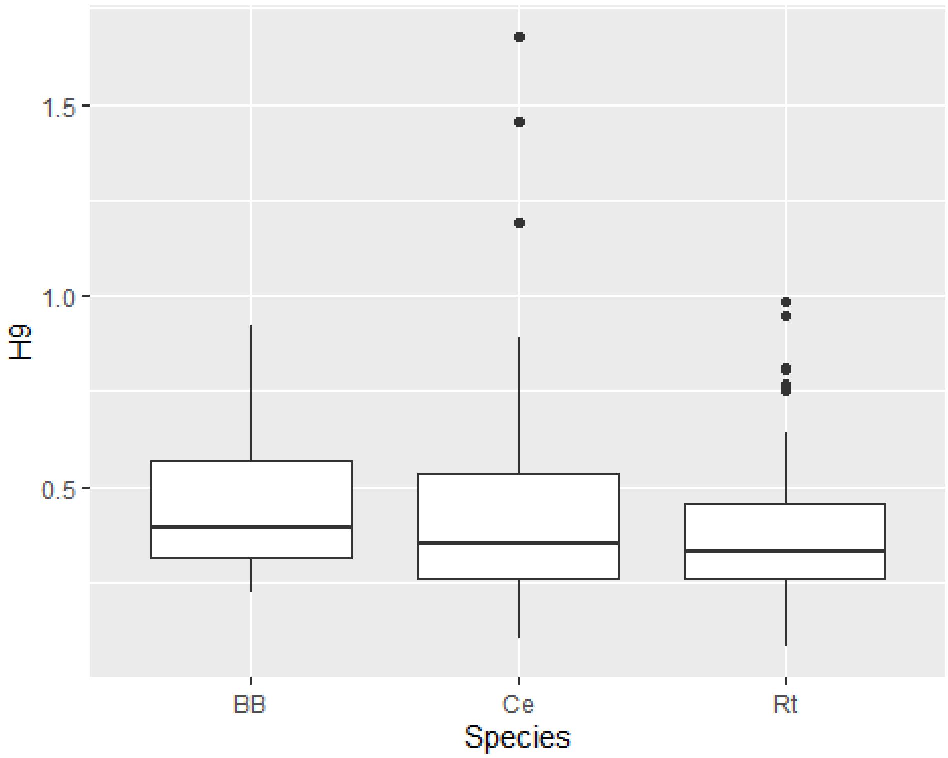


```
bf.test(F_H36 ~ F_Species, data = F)

##
##   Brown-Forsythe Test (alpha = 0.05)
## -------------------------------------------------------------
##   data : F_H36 and F_Species
```

```
##
##   statistic  : 1.466933
##   num df     : 2
##   denom df   : 18.05902
##   p.value    : 0.2568437
##
##   Result     : Difference is not statistically significant.
## -------------------------------------------------------------
```

```
ggplot(F) +
  geom_boxplot(aes(x = F_Species, y = F_H36)) +
  labs(
    x = "Species",
    y = "H36"
  )
```

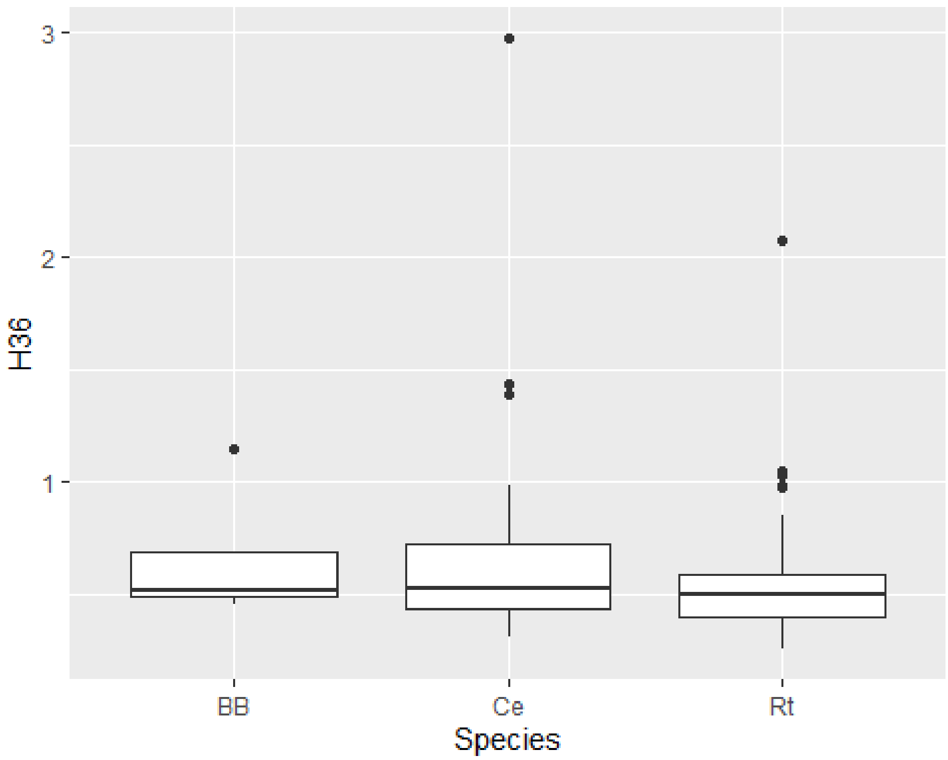


```
bf.test(F_H81 ~ F_Species, data = F)

##
##   Brown-Forsythe Test (alpha = 0.05)
## -------------------------------------------------------------
##   data : F_H81 and F_Species
##
##   statistic  : 2.019496
##   num df     : 2
##   denom df   : 11.80928
##   p.value    : 0.1760484
```

```
##
##   Result     : Difference is not statistically significant.
## -------------------------------------------------------------

ggplot(F) +
  geom_boxplot(aes(x = F_Species, y = F_H81)) +
  labs(
    x = "Species",
    y = "H81"
  )
```

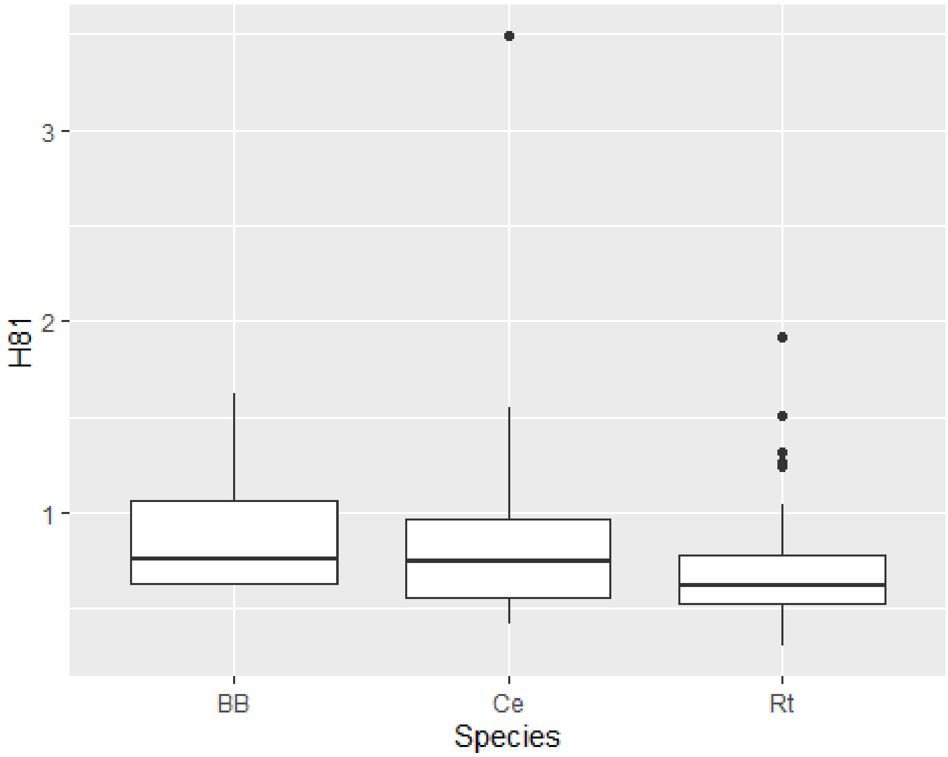

Glm: Impact of blocks over each DMTA parameter

```
glm_F_Asfc0 <- glm(F_Asfc ~ 1, data = F)
glm_F_Asfc1 <- glm(F_Asfc ~ F_Species, data = F)
Cand.models <- list()
Cand.models[[1]] <- glm_F_Asfc0
Cand.models[[2]] <- glm_F_Asfc1
Modnames <- lapply(Cand.models, "formula")
aictab(cand.set = Cand.models, modnames = paste0(Modnames), sort = TRUE)

##
## Model selection based on AICc:
##
##                       K   AICc Delta_AICc AICcWt Cum.Wt       LL
## F_Asfc ~ F_Species 4 338.32       0.00      1      1 -164.98
## F_Asfc ~ 1         2 350.46      12.13      0      1 -173.17
```

```
summary(glm_F_Asfc1)

##
## Call:
## glm(formula = F_Asfc ~ F_Species, data = F)
##
## Deviance Residuals:
##     Min       1Q   Median       3Q      Max
## -1.8124  -0.7174  -0.1184   0.4006   3.9155
##
## Coefficients:
##               Estimate Std. Error t value Pr(>|t|)
## (Intercept)    2.7995     0.5021   5.576 1.68e-07 ***
## F_SpeciesCe   -1.5515     0.5285  -2.936  0.00403 **
## F_SpeciesRt   -0.8321     0.5151  -1.615  0.10898
## ---
## Signif. codes:  0 '***' 0.001 '**' 0.01 '*' 0.05 '.' 0.1 ' ' 1
##
## (Dispersion parameter for gaussian family taken to be 1.008333)
##
##     Null deviance: 132.23  on 116  degrees of freedom
## Residual deviance: 114.95  on 114  degrees of freedom
## AIC: 337.96
##
## Number of Fisher Scoring iterations: 2

marginal <- emmeans(glm_F_Asfc1, ~F_Species)
pairs(marginal)

##  contrast estimate    SE  df t.ratio p.value
##  BB - Ce     1.552 0.529 114   2.936  0.0112
##  BB - Rt     0.832 0.515 114   1.615  0.2433
##  Ce - Rt    -0.719 0.201 114  -3.574  0.0015
##
## P value adjustment: tukey method for comparing a family of 3 estimates

glm_F_epLsar0 <- glm(F_epLsar ~ 1, data = F)
glm_F_epLsar1 <- glm(F_epLsar ~ F_Species, data = F)
Cand.models <- list()
Cand.models[[1]] <- glm_F_epLsar0
Cand.models[[2]] <- glm_F_epLsar1
Modnames <- lapply(Cand.models, "formula")
aictab(cand.set = Cand.models, modnames = paste0(Modnames), sort = TRUE)

##
## Model selection based on AICc:
##
##                      K   AICc Delta_AICc AICcWt Cum.Wt      LL
## F_epLsar ~ F_Species 4 497.90       0.00   0.79   0.79 -244.77
## F_epLsar ~ 1         2 500.55       2.65   0.21   1.00 -248.22
```

```
summary(glm_F_epLsar1)

##
## Call:
## glm(formula = F_epLsar ~ F_Species, data = F)
##
## Deviance Residuals:
##     Min       1Q   Median       3Q      Max
## -3.7652  -1.5242  -0.2452   1.5758   4.3988
##
## Coefficients:
##               Estimate Std. Error t value Pr(>|t|)
## (Intercept)   4.0882     0.9930   4.117 7.29e-05 ***
## F_SpeciesCe   1.3564     1.0453   1.298    0.197
## F_SpeciesRt   0.3429     1.0188   0.337    0.737
## ---
## Signif. codes:  0 '***' 0.001 '**' 0.01 '*' 0.05 '.' 0.1 ' ' 1
##
## (Dispersion parameter for gaussian family taken to be 3.944269)
##
##     Null deviance: 476.96  on 116  degrees of freedom
## Residual deviance: 449.65  on 114  degrees of freedom
## AIC: 497.55
##
## Number of Fisher Scoring iterations: 2

marginal <- emmeans(glm_F_epLsar1, ~F_Species)
pairs(marginal)

##  contrast estimate    SE  df t.ratio p.value
##  BB - Ce    -1.356 1.045 114  -1.298  0.3995
##  BB - Rt    -0.343 1.019 114  -0.337  0.9395
##  Ce - Rt     1.013 0.398 114   2.546  0.0326
##
## P value adjustment: tukey method for comparing a family of 3 estimates

# For Smc, We perform non-parametric tests to identify differences
kruskal.test(F_Smc ~ F_Species, data = F)

##
##  Kruskal-Wallis rank sum test
##
## data:  F_Smc by F_Species
## Kruskal-Wallis chi-squared = 10.225, df = 2, p-value = 0.00602

dunnTest(F_Smc, F_Species, method = "bonferroni")

##   Comparison           Z     P.unadj       P.adj
## 1    BB - Ce  0.09411339 0.925019080 1.000000000
## 2    BB - Rt -1.12021955 0.262620217 0.787860650
## 3    Ce - Rt -3.11378841 0.001847019 0.005541057
```

```
glm_F_H9_0 <- glm(F_H9 ~ 1, data = F)
glm_F_H9_1 <- glm(F_H9 ~ F_Species, data = F)
Cand.models <- list()
Cand.models[[1]] <- glm_F_H9_0
Cand.models[[2]] <- glm_F_H9_1
Modnames <- lapply(Cand.models, "formula")
aictab(cand.set = Cand.models, modnames = paste0(Modnames), sort = TRUE)
```

```
##
## Model selection based on AICc:
##
##                   K  AICc Delta_AICc AICcWt Cum.Wt    LL
## F_H9 ~ 1          2 11.05       0.00   0.55   0.55 -3.47
## F_H9 ~ F_Species 4 11.45       0.41   0.45   1.00 -1.55
```

```
glm_F_H36_0 <- glm(F_H36 ~ 1, data = F)
glm_F_H36_1 <- glm(F_H36 ~ F_Species, data = F)
Cand.models <- list()
Cand.models[[1]] <- glm_F_H36_0
Cand.models[[2]] <- glm_F_H36_1
Modnames <- lapply(Cand.models, "formula")
aictab(cand.set = Cand.models, modnames = paste0(Modnames), sort = TRUE)
```

```
##
## Model selection based on AICc:
##
##                    K  AICc Delta_AICc AICcWt Cum.Wt     LL
## F_H36 ~ 1          2 81.59       0.00   0.58   0.58 -38.74
## F_H36 ~ F_Species 4 82.25       0.66   0.42   1.00 -36.95
```

```
glm_F_H81_0 <- glm(F_H81 ~ 1, data = F)
glm_F_H81_1 <- glm(F_H81 ~ F_Species, data = F)
Cand.models <- list()
Cand.models[[1]] <- glm_F_H81_0
Cand.models[[2]] <- glm_F_H81_1
Modnames <- lapply(Cand.models, "formula")
aictab(cand.set = Cand.models, modnames = paste0(Modnames), sort = TRUE)
```

```
##
## Model selection based on AICc:
##
##                    K   AICc Delta_AICc AICcWt Cum.Wt     LL
## F_H81 ~ F_Species 4 111.93       0.00   0.72   0.72 -51.79
## F_H81 ~ 1          2 113.78       1.86   0.28   1.00 -54.84
```

```
summary(glm_F_H81_1)
```

```
##
## Call:
## glm(formula = F_H81 ~ F_Species, data = F)
##
## Deviance Residuals:
```

```
##      Min        1Q    Median        3Q       Max
## -0.44357  -0.18989  -0.09389   0.10211   2.63043
##
## Coefficients:
##             Estimate Std. Error t value Pr(>|t|)
## (Intercept)  0.93650    0.19081   4.908 3.09e-06 ***
## F_SpeciesCe -0.07293    0.20086  -0.363    0.717
## F_SpeciesRt -0.24661    0.19577  -1.260    0.210
## ---
## Signif. codes:  0 '***' 0.001 '**' 0.01 '*' 0.05 '.' 0.1 ' ' 1
##
## (Dispersion parameter for gaussian family taken to be 0.145632)
##
##     Null deviance: 17.492  on 116  degrees of freedom
## Residual deviance: 16.602  on 114  degrees of freedom
## AIC: 111.57
##
## Number of Fisher Scoring iterations: 2
```

```
marginal <- emmeans(glm_F_H81_1, ~F_Species)
pairs(marginal)
```

```
##  contrast estimate     SE  df t.ratio p.value
##  BB - Ce    0.0729 0.2009 114   0.363  0.9300
##  BB - Rt    0.2466 0.1958 114   1.260  0.4209
##  Ce - Rt    0.1737 0.0765 114   2.270  0.0642
##
## P value adjustment: tukey method for comparing a family of 3 estimates
```

Block G

```
G_Species <- G %>%
  dplyr::select(c(2)) %>%
  unlist(c(1))

G_Asfc <- G %>%
  dplyr::select(c(7)) %>%
  unlist(c(1))
G_epLsar <- G %>%
  dplyr::select(c(8)) %>%
  unlist(c(1))
G_Smc <- G %>%
  dplyr::select(c(9)) %>%
  unlist(c(1))
G_H9 <- G %>%
  dplyr::select(c(10)) %>%
  unlist(c(1))
G_H36 <- G %>%
  dplyr::select(c(12)) %>%
  unlist(c(1))
G_H81 <- G %>%
```

```
  dplyr::select(c(11)) %>%
  unlist(c(1))
```

Checking data distribution and outliers:

```
x <- G[order(G_Asfc), ]
x$specie <- factor(x$specie)
dotchart(x$Asfc, cex = 1, pch = 16, groups = x$specie, xlab = "G_Asfc per spe
cie")
```

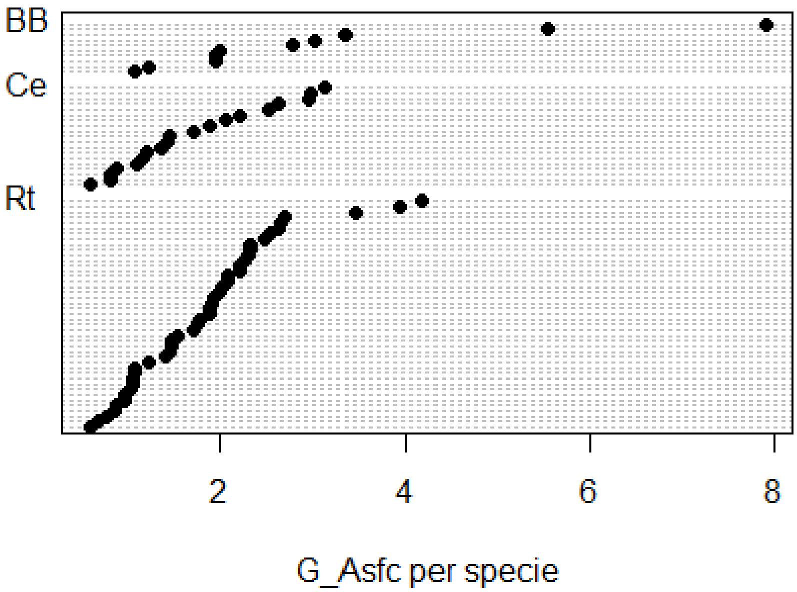


```
x <- G[order(G_epLsar), ]
x$specie <- factor(x$specie)
dotchart(x$epLsar, cex = 1, pch = 16, groups = x$specie, xlab = "G_epLsar per
specie")
```

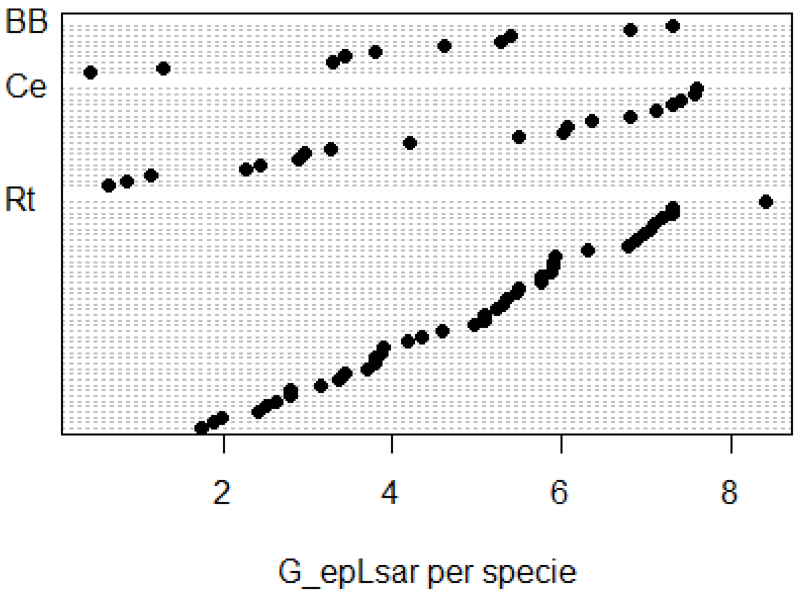


```
x <- G[order(G_Smc), ]
x$specie <- factor(x$specie)
dotchart(x$Smc, cex = 1, pch = 16, groups = x$specie, xlab = "G_Smc per speci
e")
```

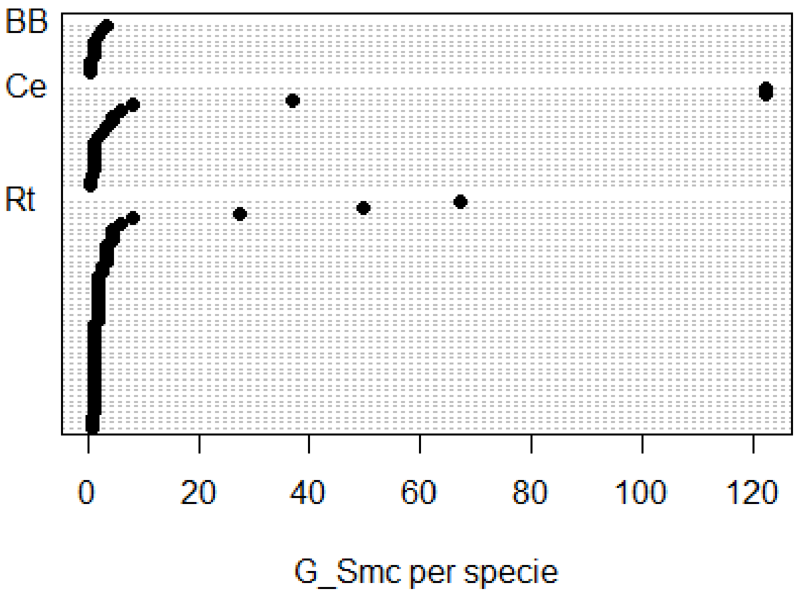


```
x <- G[order(G_H9), ]
x$specie <- factor(x$specie)
dotchart(x$HAsfc9, cex = 1, pch = 16, groups = x$specie, xlab = "G_H9 per spe
cie")
```

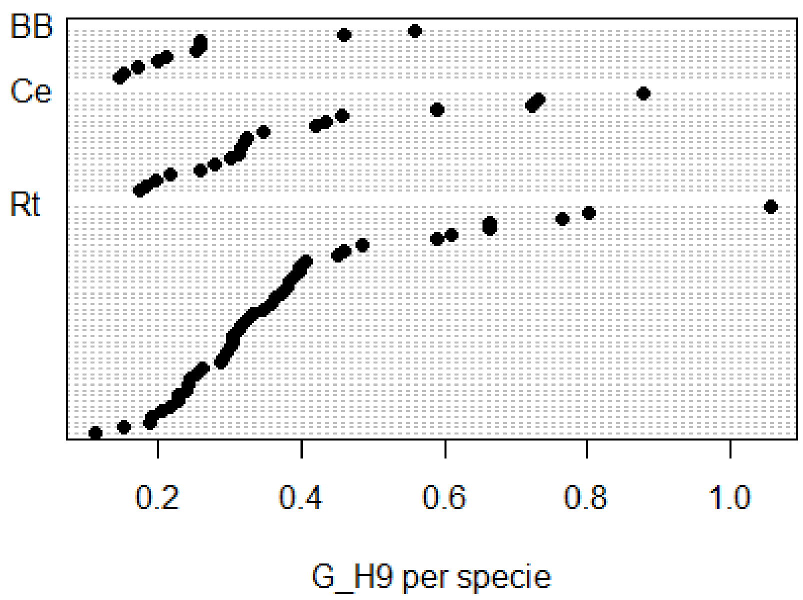


```
x <- G[order(G_H36), ]
x$specie <- factor(x$specie)
dotchart(x$HAsfc36, cex = 1, pch = 16, groups = x$specie, xlab = "G_H36 per s
pecie")
```

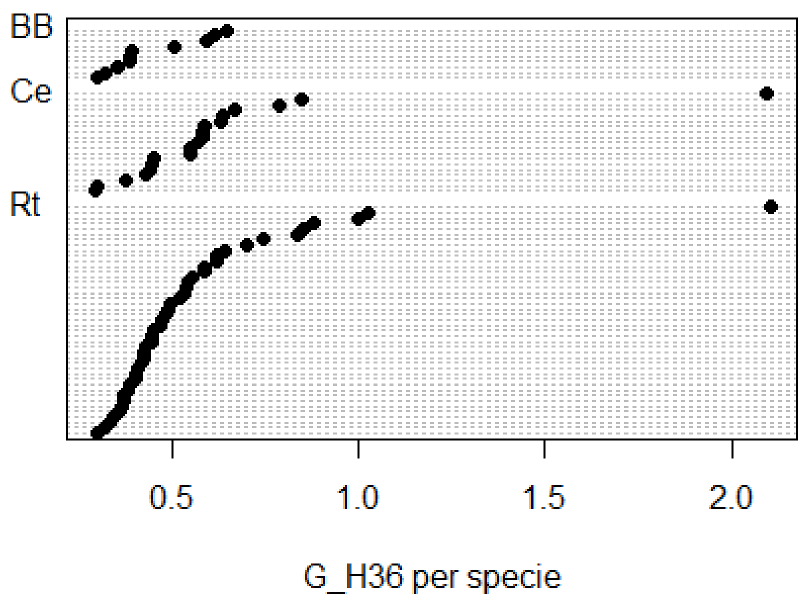


```
x <- G[order(G_H81), ]
x$specie <- factor(x$specie)
dotchart(x$HAsfc81, cex = 1, pch = 16, groups = x$specie, xlab = "G_H81 per s
pecie")
```

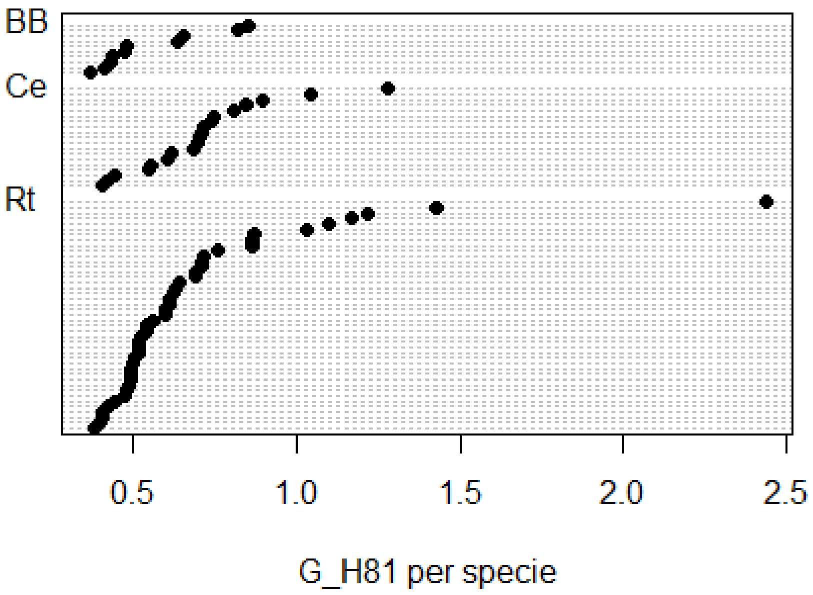

Graphical evaluation of the tests’ applicability:

Normality and homoscedasticity of the variables. #### Normality

```
ggplot(G) +
  geom_freqpoly(aes(x = Asfc), bins = 7) +
  labs(
    x = "Value Asfc",
    y = "Frequency"
  )
```

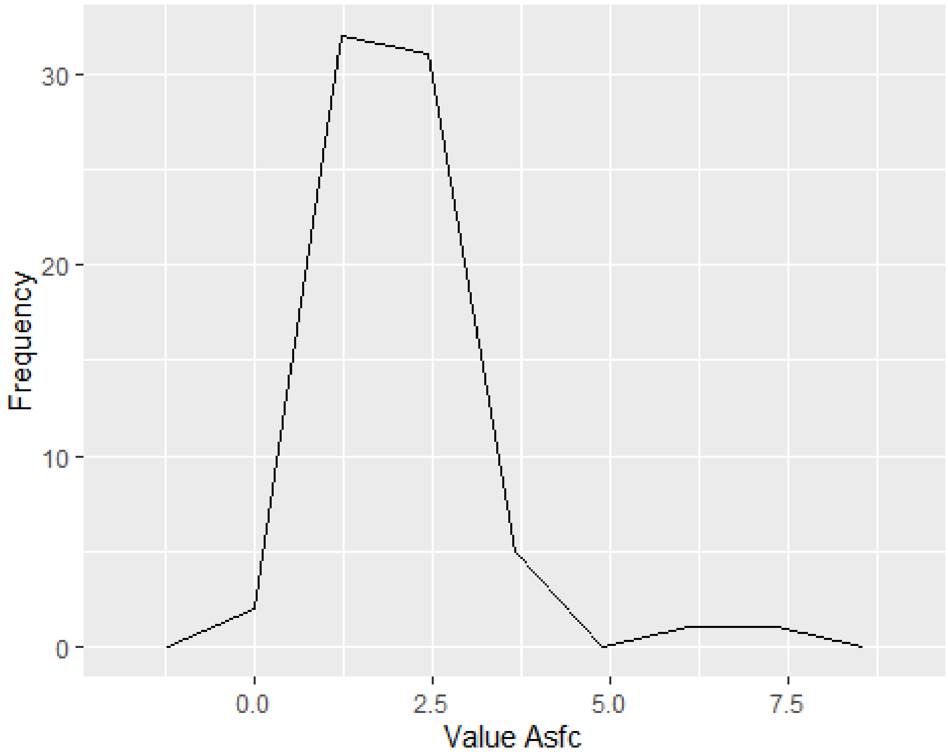


```
ggplot(G) +
  geom_freqpoly(aes(x = epLsar), bins = 7) +
  labs(
    x = "Value epLsar",
    y = "Frequency"
  )
```

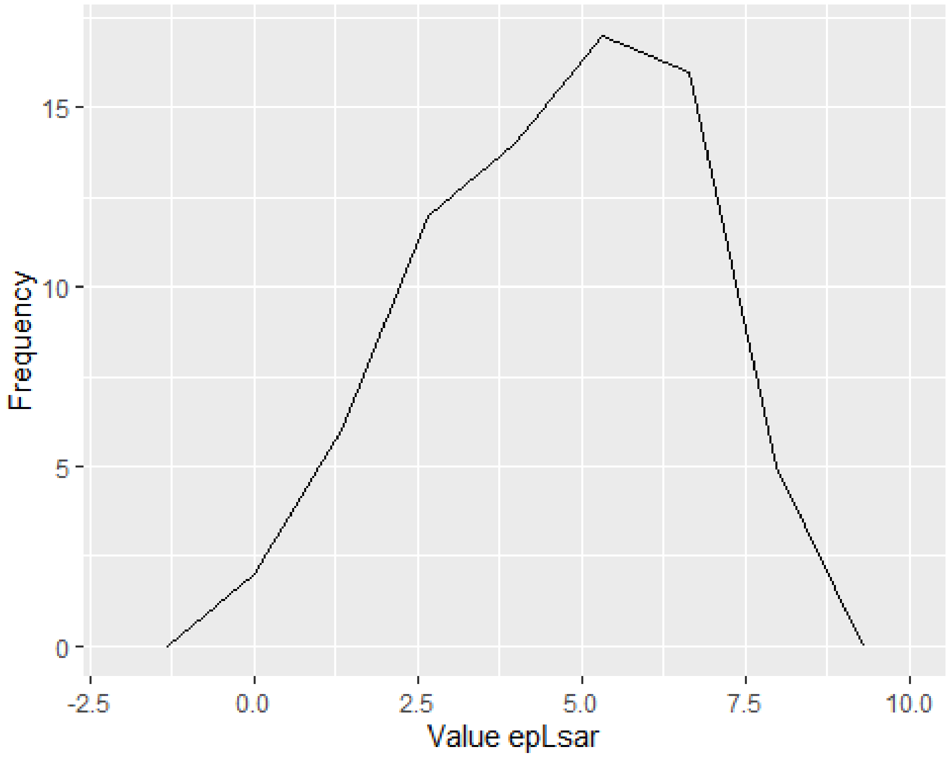


```
ggplot(G) +
  geom_freqpoly(aes(x = Smc), bins = 7) +
  labs(
    x = "Value Smc",
    y = "Frequency"
  )
```

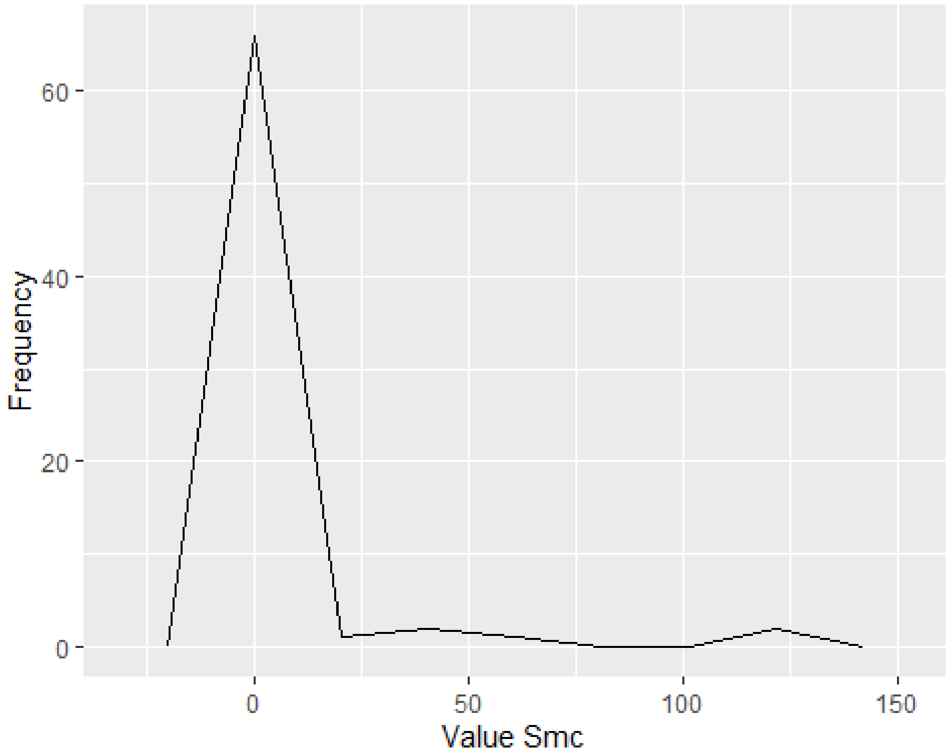


```
ggplot(G) +
  geom_freqpoly(aes(x = HAsfc9), bins = 7) +
  labs(
    x = "Value H9",
    y = "Frequency"
  )
```

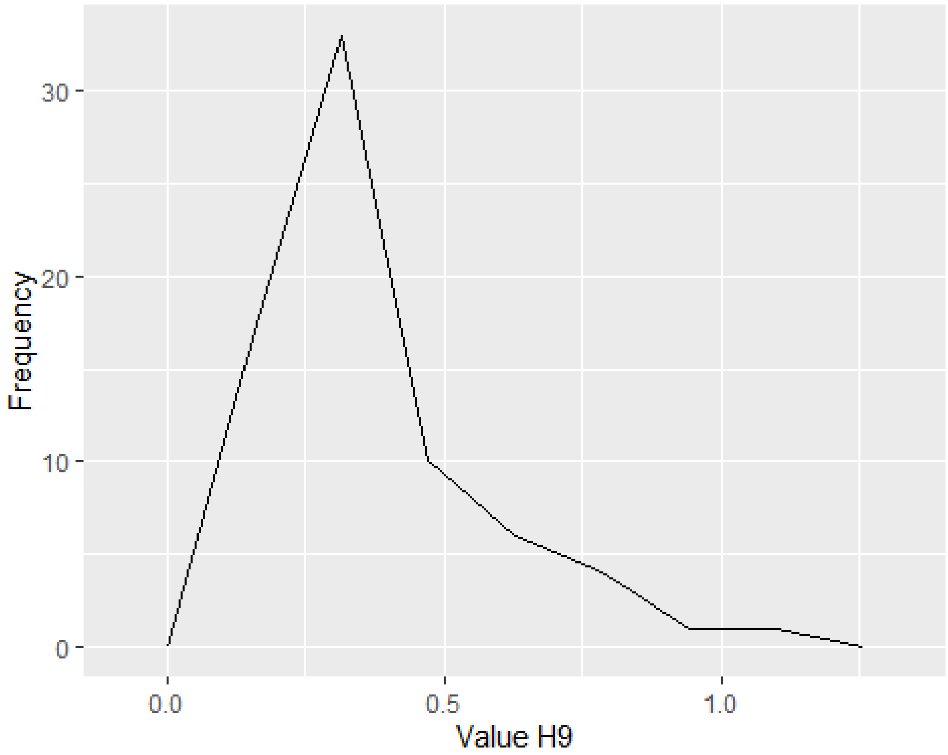


```
ggplot(G) +
  geom_freqpoly(aes(x = HAsfc36), bins = 7) +
  labs(
    x = "Value H36",
    y = "Frequency"
  )
```

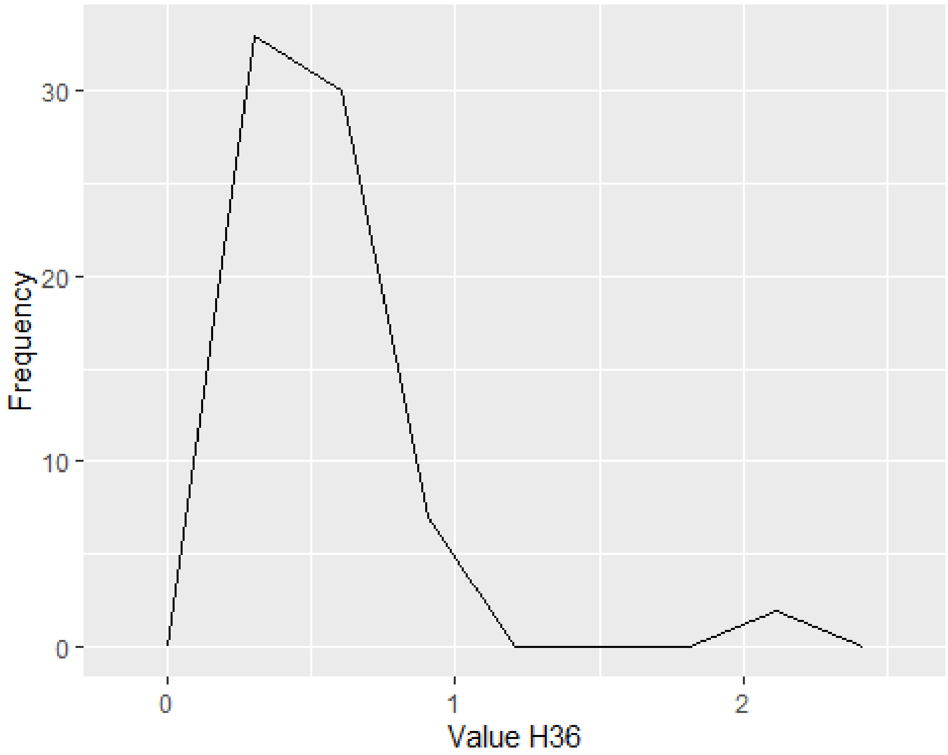


```
ggplot(G) +
  geom_freqpoly(aes(x = HAsfc81), bins = 7) +
  labs(
    x = "Value H81",
    y = "Frequency"
  )
```

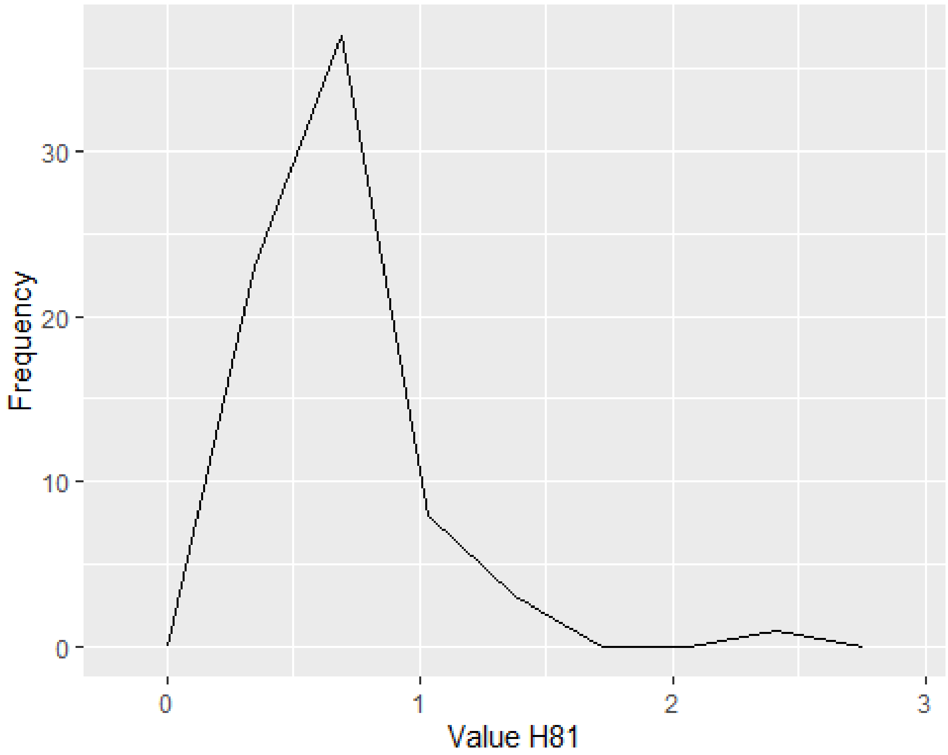

Homoscedasticity: Brown & Forsythe test (and data transformation whenever needed)

```
bf.test(G_Asfc ~ G_Species, data = G)

##
##   Brown-Forsythe Test (alpha = 0.05)
## -------------------------------------------------------------
##   data : G_Asfc and G_Species
##
##   statistic  : 3.063543
##   num df     : 2
##   denom df   : 12.7595
##   p.value    : 0.08192231
##
##   Result     : Difference is not statistically significant.
## -------------------------------------------------------------

ggplot(G) +
  geom_boxplot(aes(x = G_Species, y = G_Asfc)) +
  labs(
    x = "Species",
    y = "Asfc"
  )
```

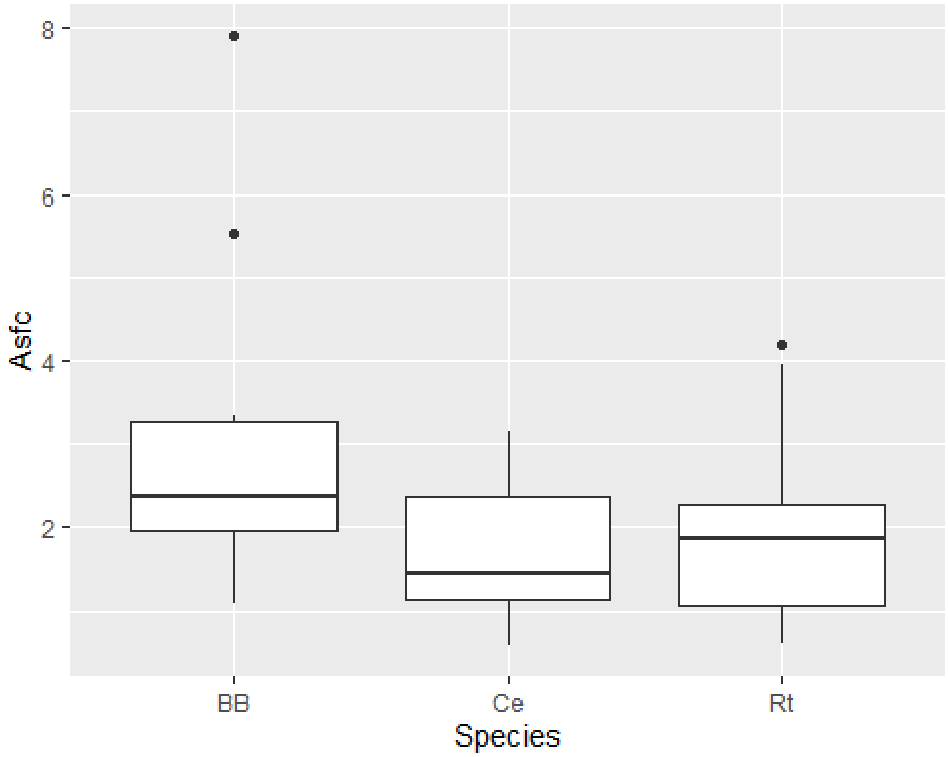


```
bf.test(G_epLsar ~ G_Species, data = G)

##
##   Brown-Forsythe Test (alpha = 0.05)
## -------------------------------------------------------------
##   data : G_epLsar and G_Species
##
##   statistic  : 0.4114377
##   num df     : 2
##   denom df   : 31.67939
##   p.value    : 0.666187
##
##   Result     : Difference is not statistically significant.
## -------------------------------------------------------------

ggplot(G) +
  geom_boxplot(aes(x = G_Species, y = G_epLsar)) +
  labs(
    x = "Species",
    y = "epLsar"
  )
```

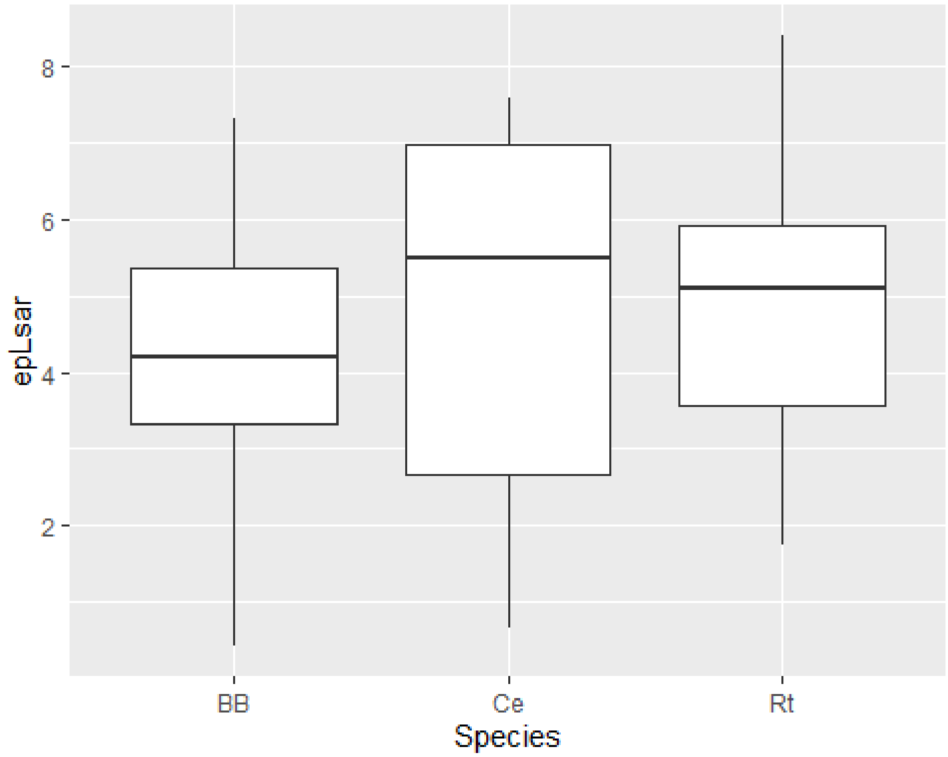


```
bf.test(G_Smc ~ G_Species, data = G)

##
##   Brown-Forsythe Test (alpha = 0.05)
## -------------------------------------------------------------
##   data : G_Smc and G_Species
##
##   statistic  : 1.977922
##   num df     : 2
##   denom df   : 20.26033
##   p.value    : 0.1641828
##
##   Result     : Difference is not statistically significant.
## -------------------------------------------------------------

ggplot(G) +
  geom_boxplot(aes(x = G_Species, y = G_Smc)) +
  labs(
    x = "Species",
    y = "Smc"
  )
```

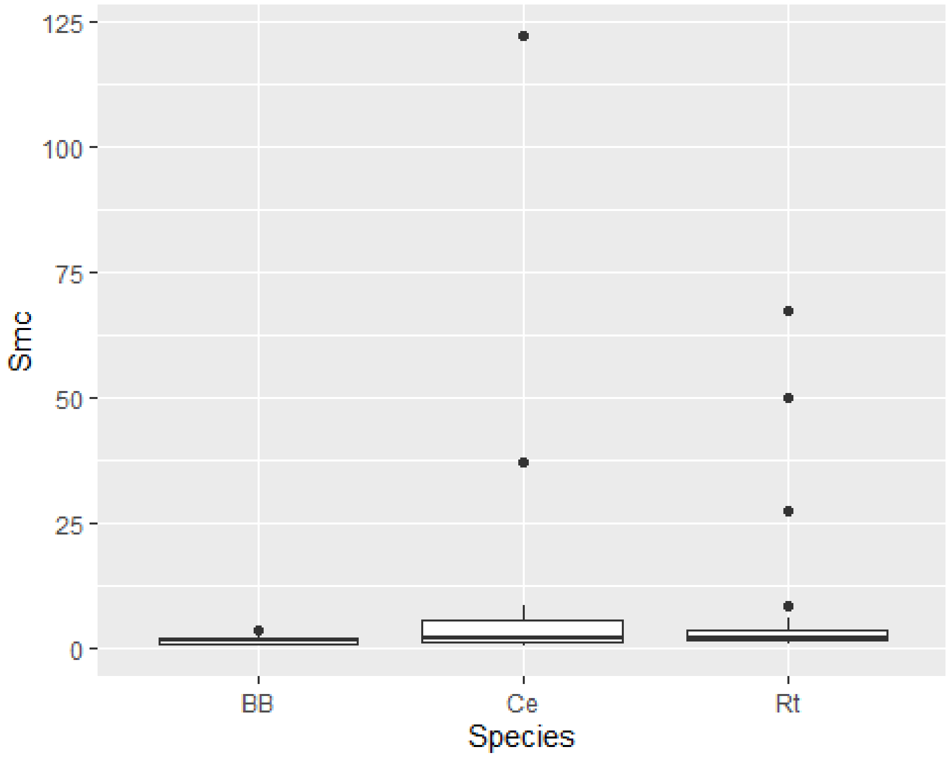


```
bf.test(G_H9 ~ G_Species, data = G)

##
##   Brown-Forsythe Test (alpha = 0.05)
## -------------------------------------------------------------
##   data : G_H9 and G_Species
##
##   statistic  : 1.908295
##   num df     : 2
##   denom df   : 43.86276
##   p.value    : 0.1604465
##
##   Result     : Difference is not statistically significant.
## -------------------------------------------------------------

ggplot(G) +
  geom_boxplot(aes(x = G_Species, y = G_H9)) +
  labs(
    x = "Species",
    y = "H9"
  )
```

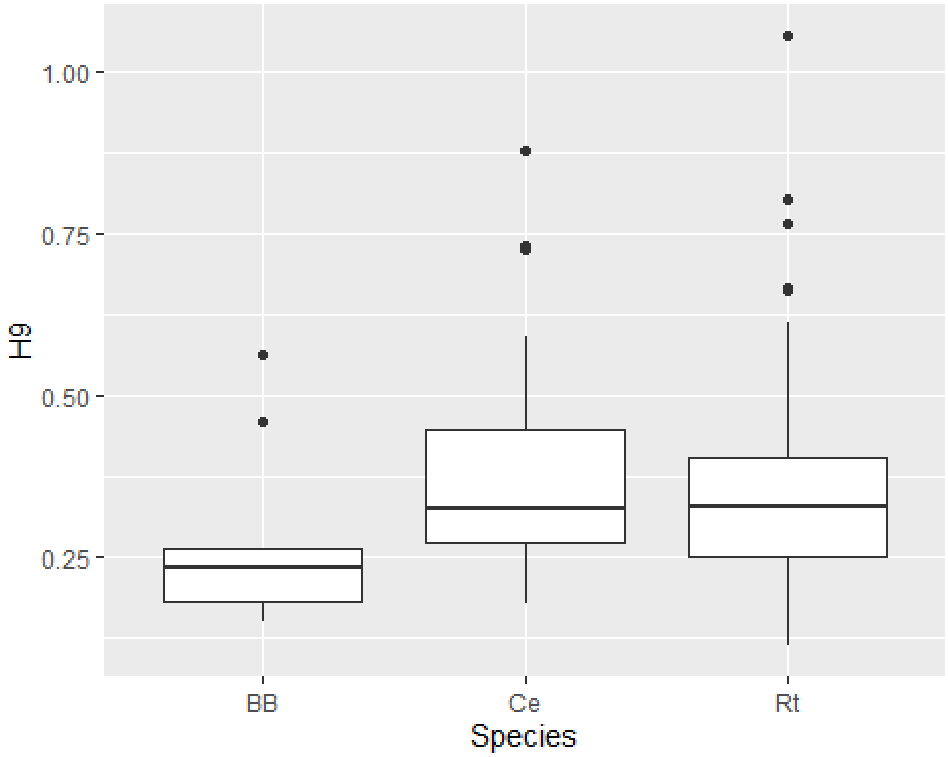


```
bf.test(G_H36 ~ G_Species, data = G)

##
##   Brown-Forsythe Test (alpha = 0.05)
## -------------------------------------------------------------
##   data : G_H36 and G_Species
##
##   statistic  : 1.239541
##   num df     : 2
##   denom df   : 35.65305
##   p.value    : 0.3016931
##
##   Result     : Difference is not statistically significant.
## -------------------------------------------------------------
```

```
ggplot(G) +
  geom_boxplot(aes(x = G_Species, y = G_H36)) +
  labs(
    x = "Species",
    y = "H36"
  )
```

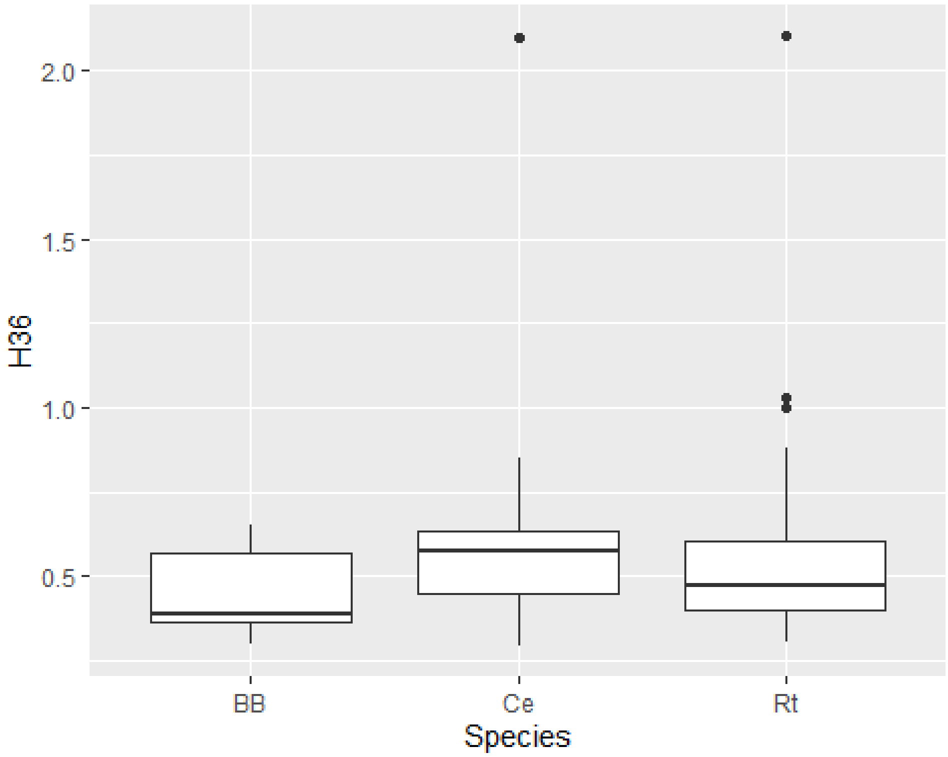


```
bf.test(G_H81 ~ G_Species, data = G)

##
##   Brown-Forsythe Test (alpha = 0.05)
## -------------------------------------------------------------
##   data : G_H81 and G_Species
##
##   statistic  : 1.526839
##   num df     : 2
##   denom df   : 61.87731
##   p.value    : 0.2252917
##
##   Result     : Difference is not statistically significant.
## -------------------------------------------------------------

ggplot(G) +
  geom_boxplot(aes(x = G_Species, y = G_H81)) +
  labs(
    x = "Species",
    y = "H81"
  )
```

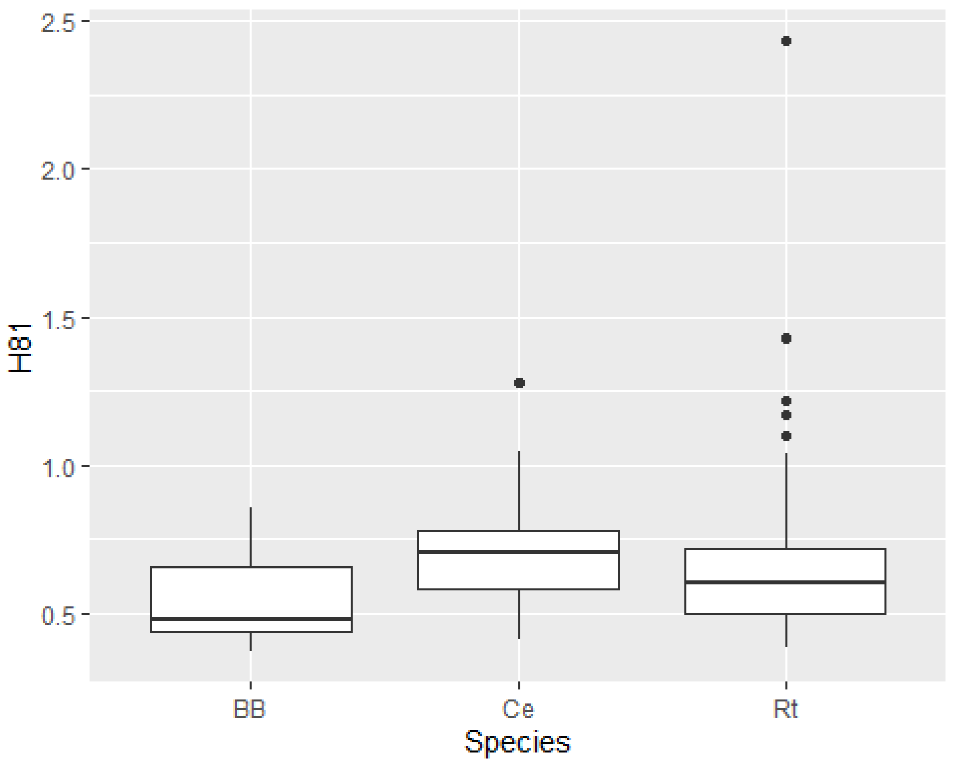

Glm: Impact of blocks over each DMTA parameter

```
glm_G_Asfc0 <- glm(G_Asfc ~ 1, data = G)
glm_G_Asfc1 <- glm(G_Asfc ~ G_Species, data = G)
Cand.models <- list()
Cand.models[[1]] <- glm_G_Asfc0
Cand.models[[2]] <- glm_G_Asfc1
Modnames <- lapply(Cand.models, "formula")
aictab(cand.set = Cand.models, modnames = paste0(Modnames), sort = TRUE)

##
## Model selection based on AICc:
##
##                      K   AICc Delta_AICc AICcWt Cum.Wt      LL
## G_Asfc ~ G_Species 4 221.56       0.00   0.97   0.97 -106.48
## G_Asfc ~ 1         2 228.80       7.24   0.03   1.00 -112.31

summary(glm_F_Asfc1)

##
## Call:
## glm(formula = F_Asfc ~ F_Species, data = F)
##
## Deviance Residuals:
##     Min       1Q   Median       3Q      Max
## -1.8124  -0.7174  -0.1184   0.4006   3.9155
##
## Coefficients:
```

```
##              Estimate Std. Error t value Pr(>|t|)
## (Intercept)   2.7995     0.5021   5.576 1.68e-07 ***
## F_SpeciesCe  -1.5515     0.5285  -2.936  0.00403 **
## F_SpeciesRt  -0.8321     0.5151  -1.615  0.10898
## ---
## Signif. codes:  0 '***' 0.001 '**' 0.01 '*' 0.05 '.' 0.1 ' ' 1
##
## (Dispersion parameter for gaussian family taken to be 1.008333)
##
##     Null deviance: 132.23  on 116  degrees of freedom
## Residual deviance: 114.95  on 114  degrees of freedom
## AIC: 337.96
##
## Number of Fisher Scoring iterations: 2
```

```
marginal <- emmeans(glm_F_Asfc1, ~F_Species)
pairs(marginal)
```

```
##  contrast estimate    SE  df t.ratio p.value
##  BB - Ce     1.552 0.529 114   2.936  0.0112
##  BB - Rt     0.832 0.515 114   1.615  0.2433
##  Ce - Rt    -0.719 0.201 114  -3.574  0.0015
##
## P value adjustment: tukey method for comparing a family of 3 estimates
```

```
glm_G_epLsar0 <- glm(G_epLsar ~ 1, data = G)
glm_G_epLsar1 <- glm(G_epLsar ~ G_Species, data = G)
Cand.models <- list()
Cand.models[[1]] <- glm_G_epLsar0
Cand.models[[2]] <- glm_G_epLsar1
Modnames <- lapply(Cand.models, "formula")
aictab(cand.set = Cand.models, modnames = paste0(Modnames), sort = TRUE)
```

```
##
## Model selection based on AICc:
##
##                      K   AICc Delta_AICc AICcWt Cum.Wt      LL
## G_epLsar ~ 1         2 307.57       0.00   0.84   0.84 -151.70
## G_epLsar ~ G_Species 4 310.95       3.39   0.16   1.00 -151.18
```

```
glm_G_Smc0 <- glm(G_Smc ~ 1, data = G)
glm_G_Smc1 <- glm(G_Smc ~ G_Species, data = G)
Cand.models <- list()
Cand.models[[1]] <- glm_G_Smc0
Cand.models[[2]] <- glm_G_Smc1
Modnames <- lapply(Cand.models, "formula")
aictab(cand.set = Cand.models, modnames = paste0(Modnames), sort = TRUE)
```

```
##
## Model selection based on AICc:
##
```

```
##                     K   AICc Delta_AICc AICcWt Cum.Wt      LL
## G_Smc ~ G_Species 4 653.59       0.00   0.54   0.54 -322.50
## G_Smc ~ 1         2 653.91       0.32   0.46   1.00 -324.87
```

```
summary(glm_G_Smc1)
```

```
## 
## Call:
## glm(formula = G_Smc ~ G_Species, data = G)
## 
## Deviance Residuals: 
##     Min       1Q   Median       3Q      Max  
## -16.471   -4.612   -3.760   -0.847  105.331  
## 
## Coefficients:
##             Estimate Std. Error t value Pr(>|t|)  
## (Intercept)    1.402      6.891   0.203   0.8394  
## G_SpeciesCe   15.479      8.513   1.818   0.0734 .
## G_SpeciesRt    3.957      7.650   0.517   0.6066  
## ---
## Signif. codes:  0 '***' 0.001 '**' 0.01 '*' 0.05 '.' 0.1 ' ' 1
## 
## (Dispersion parameter for gaussian family taken to be 474.8135)
## 
##     Null deviance: 34994  on 71  degrees of freedom
## Residual deviance: 32762  on 69  degrees of freedom
## AIC: 652.99
## 
## Number of Fisher Scoring iterations: 2
```

```
marginal <- emmeans(glm_G_Smc1, ~G_Species)
pairs(marginal)
```

```
##  contrast estimate   SE df t.ratio p.value
##  BB - Ce    -15.48 8.51 69  -1.818  0.1712
##  BB - Rt     -3.96 7.65 69  -0.517  0.8632
##  Ce - Rt     11.52 6.00 69   1.919  0.1409
## 
## P value adjustment: tukey method for comparing a family of 3 estimates
```

```
glm_G_H9_0 <- glm(G_H9 ~ 1, data = G)
glm_G_H9_1 <- glm(G_H9 ~ G_Species, data = G)
Cand.models <- list()
Cand.models[[1]] <- glm_G_H9_0
Cand.models[[2]] <- glm_G_H9_1
Modnames <- lapply(Cand.models, "formula")
aictab(cand.set = Cand.models, modnames = paste0(Modnames), sort = TRUE)
```

```
## 
## Model selection based on AICc:
## 
```

```
##                    K   AICc Delta_AICc AICcWt Cum.Wt    LL
## G_H9 ~ 1          2 -33.27       0.00   0.63   0.63 18.72
## G_H9 ~ G_Species 4 -32.21       1.06   0.37   1.00 20.40
```

```
glm_G_H36_0 <- glm(G_H36 ~ 1, data = G)
glm_G_H36_1 <- glm(G_H36 ~ G_Species, data = G)
Cand.models <- list()
Cand.models[[1]] <- glm_G_H36_0
Cand.models[[2]] <- glm_G_H36_1
Modnames <- lapply(Cand.models, "formula")
aictab(cand.set = Cand.models, modnames = paste0(Modnames), sort = TRUE)
```

```
##
## Model selection based on AICc:
##
##                     K  AICc Delta_AICc AICcWt Cum.Wt     LL
## G_H36 ~ 1           2 38.64       0.00   0.76   0.76 -17.23
## G_H36 ~ G_Species 4 40.93       2.29   0.24   1.00 -16.17
```

```
glm_G_H81_0 <- glm(G_H81 ~ 1, data = G)
glm_G_H81_1 <- glm(G_H81 ~ G_Species, data = G)
Cand.models <- list()
Cand.models[[1]] <- glm_G_H81_0
Cand.models[[2]] <- glm_G_H81_1
Modnames <- lapply(Cand.models, "formula")
aictab(cand.set = Cand.models, modnames = paste0(Modnames), sort = TRUE)
```

```
##
## Model selection based on AICc:
##
##                     K  AICc Delta_AICc AICcWt Cum.Wt     LL
## G_H81 ~ 1           2 37.79       0.00   0.78   0.78 -16.81
## G_H81 ~ G_Species 4 40.37       2.58   0.22   1.00 -15.89
```

Block H

```
H_Species <- H %>%
  dplyr::select(c(2)) %>%
  unlist(c(1))

H_Asfc <- H %>%
  dplyr::select(c(7)) %>%
  unlist(c(1))
H_epLsar <- H %>%
  dplyr::select(c(8)) %>%
  unlist(c(1))
H_Smc <- H %>%
  dplyr::select(c(9)) %>%
  unlist(c(1))
H_H9 <- H %>%
  dplyr::select(c(10)) %>%
  unlist(c(1))
```

```
H_H36 <- H %>%
  dplyr::select(c(12)) %>%
  unlist(c(1))
H_H81 <- H %>%
  dplyr::select(c(11)) %>%
  unlist(c(1))
```

But N<5 for red deer so we don't go further.

Block I

```
global_db_FOSSILES_blocI <- read_delim("global db_FOSSILES_blocI.csv", delim
= ";", escape_double = FALSE, trim_ws = TRUE)
I<-global_db_FOSSILES_blocI # the only bovid of Block I is taken out of as it
s occurrence avoid performing bf test.
summary(I)

##     Group              specie             Period              Blocs          
##  Length:30          Length:30          Length:30          Length:30         
##  Class :character   Class :character   Class :character   Class :character  
##  Mode  :character   Mode  :character   Mode  :character   Mode  :character  
##                                                                             
##                                                                             
##                                                                             
##     layer              ref DMTA           Asfc           epLsar      
##  Length:30          Min.   : 521.0   Min.   :0.516   Min.   : 1.658  
##  Class :character   1st Qu.: 530.5   1st Qu.:1.098   1st Qu.: 4.453  
##  Mode  :character   Median : 546.5   Median :1.637   Median : 5.481  
##                     Mean   : 591.1   Mean   :1.695   Mean   : 5.502  
##                     3rd Qu.: 554.8   3rd Qu.:2.128   3rd Qu.: 6.261  
##                     Max.   :1028.0   Max.   :3.776   Max.   :10.607  
##       Smc             HAsfc9           HAsfc81          HAsfc36      
##  Min.   : 0.410   Min.   :0.1220   Min.   :0.3600   Min.   :0.2940  
##  1st Qu.: 1.096   1st Qu.:0.2675   1st Qu.:0.5347   1st Qu.:0.4218  
##  Median : 1.837   Median :0.3360   Median :0.6305   Median :0.5005  
##  Mean   : 7.055   Mean   :0.3242   Mean   :0.6418   Mean   :0.4994  
##  3rd Qu.: 3.129   3rd Qu.:0.3862   3rd Qu.:0.7362   3rd Qu.:0.5697  
##  Max.   :67.093   Max.   :0.5090   Max.   :1.2000   Max.   :0.9100  
##   Disp-Asfc-i      Disp-epLsar-i      Disp-H9-i      
##  Min.   :0.00000   Min.   :0.0000   Min.   :0.00000  
##  1st Qu.:0.08975   1st Qu.:0.0825   1st Qu.:0.01825  
##  Median :0.19450   Median :0.1540   Median :0.05350  
##  Mean   :0.21723   Mean   :0.2064   Mean   :0.05707  
##  3rd Qu.:0.31750   3rd Qu.:0.2828   3rd Qu.:0.07575  
##  Max.   :0.56200   Max.   :0.8930   Max.   :0.18500  

I_Species <- I %>%
  dplyr::select(c(2)) %>%
  unlist(c(1))

I_Asfc <- I %>%
  dplyr::select(c(7)) %>%
```

```
  unlist(c(1))
I_epLsar <- I %>%
  dplyr::select(c(8)) %>%
  unlist(c(1))
I_Smc <- I %>%
  dplyr::select(c(9)) %>%
  unlist(c(1))
I_H9 <- I %>%
  dplyr::select(c(10)) %>%
  unlist(c(1))
I_H36 <- I %>%
  dplyr::select(c(12)) %>%
  unlist(c(1))
I_H81 <- I %>%
  dplyr::select(c(11)) %>%
  unlist(c(1))
```

Checking data distribution and outliers:

```
x <- I[order(I_Asfc), ]
x$specie <- factor(x$specie)
dotchart(x$Asfc, cex = 1, pch = 16, groups = x$specie, xlab = "I_Asfc per spe
cie")
```

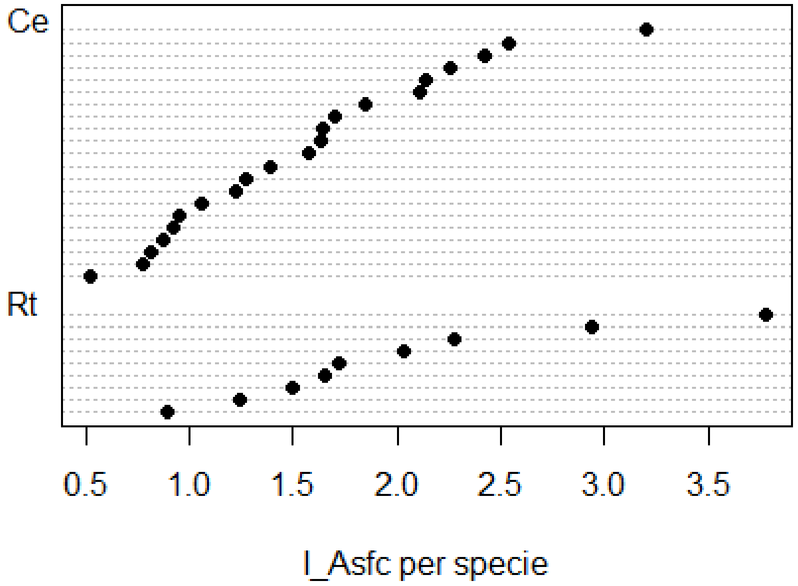


```
x <- I[order(I_epLsar), ]
x$specie <- factor(x$specie)
dotchart(x$epLsar, cex = 1, pch = 16, groups = x$specie, xlab = "I_epLsar per
specie")
```

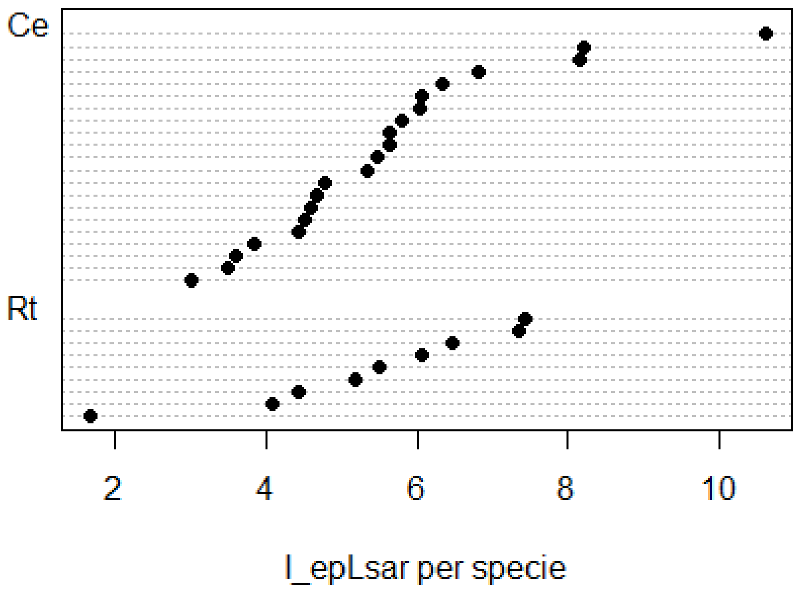


```
x <- I[order(I_Smc), ]
x$specie <- factor(x$specie)
dotchart(x$Smc, cex = 1, pch = 16, groups = x$specie, xlab = "I_Smc per speci
e")
```

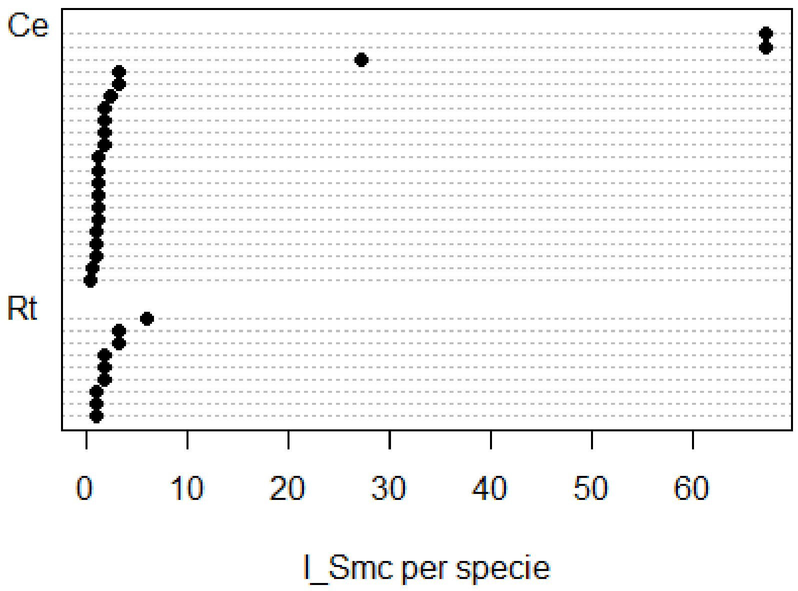


```
x <- I[order(I_H9), ]
x$specie <- factor(x$specie)
dotchart(x$HAsfc9, cex = 1, pch = 16, groups = x$specie, xlab = "I_H9 per spe
cie")
```

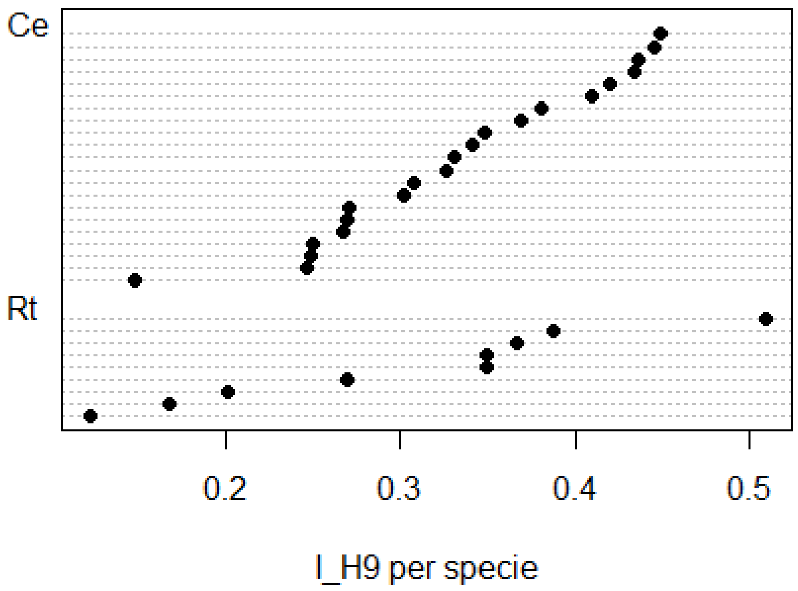


```
x <- I[order(I_H36), ]
x$specie <- factor(x$specie)
dotchart(x$HAsfc36, cex = 1, pch = 16, groups = x$specie, xlab = "I_H36 per s
pecie")
```

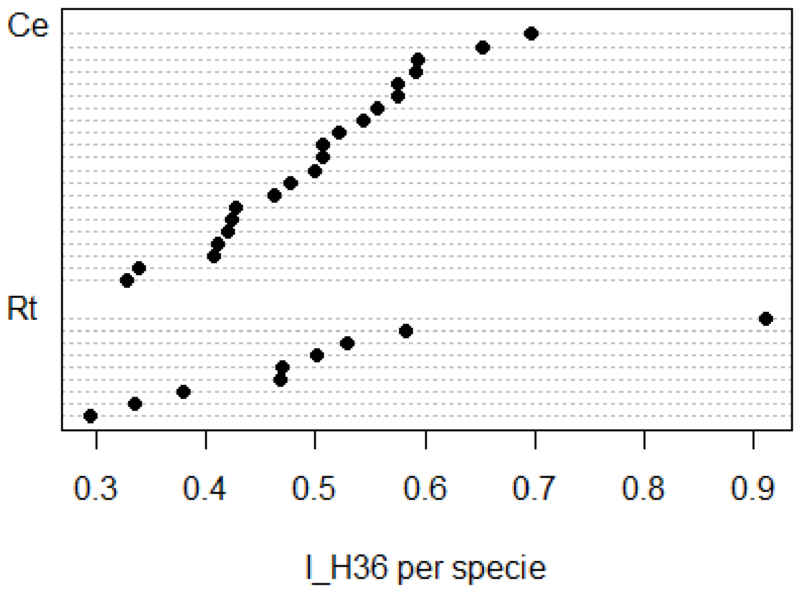


```
x <- I[order(I_H81), ]
x$specie <- factor(x$specie)
dotchart(x$HAsfc81, cex = 1, pch = 16, groups = x$specie, xlab = "I_H81 per s
pecie")
```

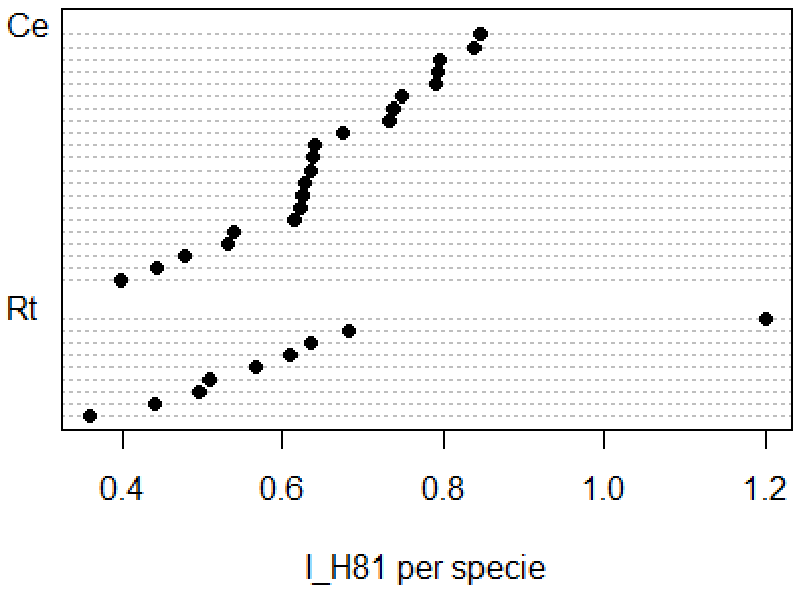

Graphical evaluation of the tests’ applicability:

Normality and homoscedasticity of the variables. #### Normality

```
ggplot(I) +
  geom_freqpoly(aes(x = Asfc), bins = 7) +
  labs(
    x = "Value Asfc",
    y = "Frequency"
  )
```

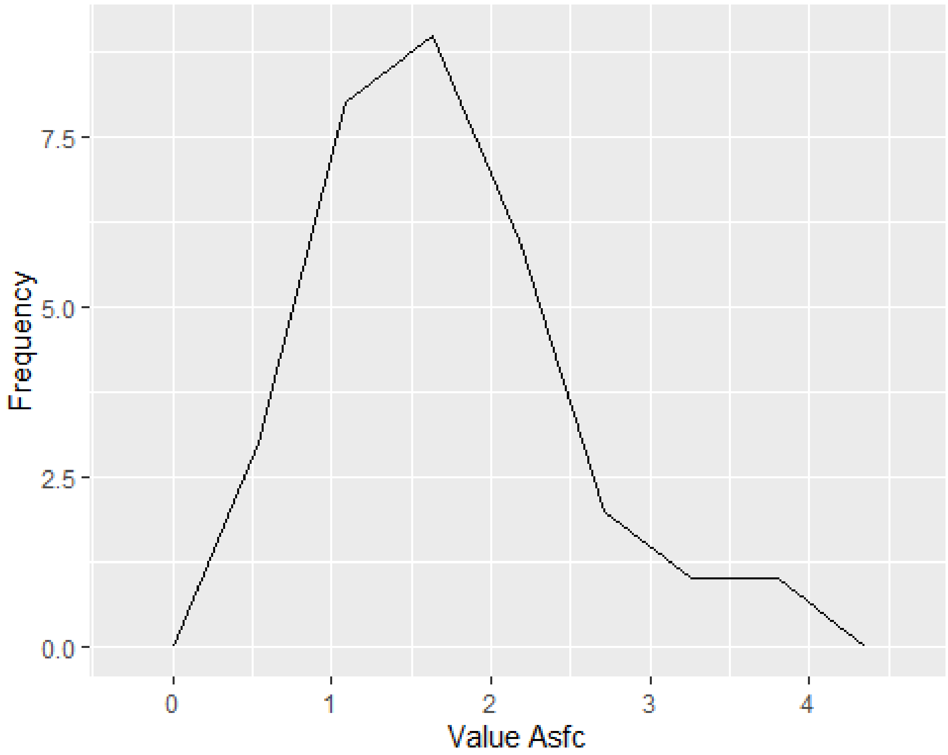


```r
ggplot(I) +
  geom_freqpoly(aes(x = epLsar), bins = 7) +
  labs(
    x = "Value epLsar",
    y = "Frequency"
  )
```

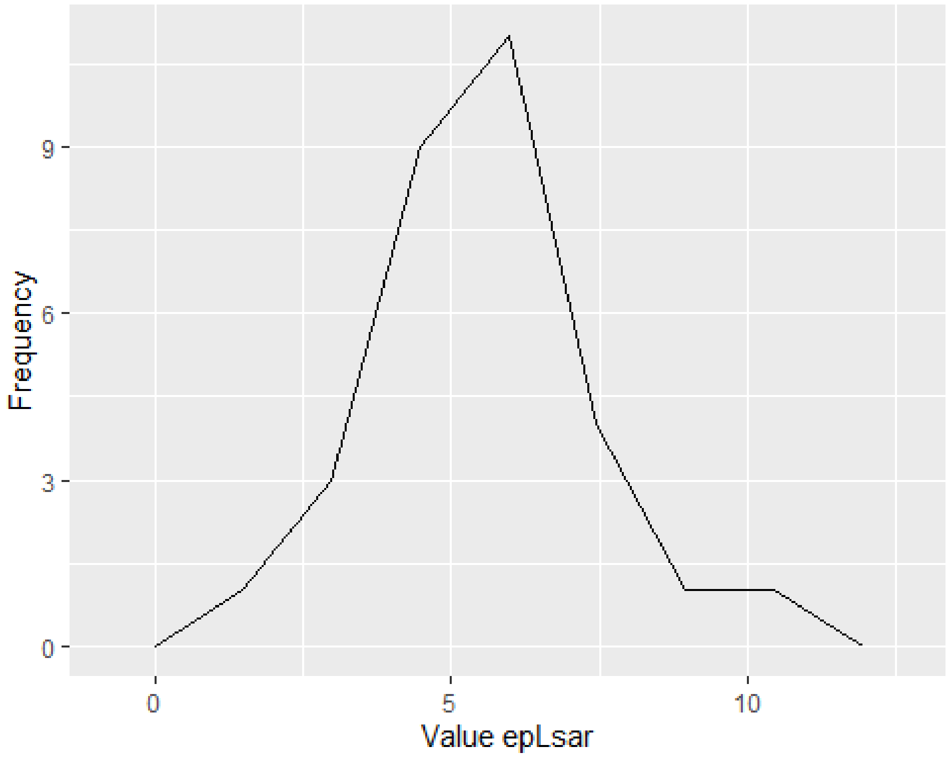


```
ggplot(I) +
  geom_freqpoly(aes(x = Smc), bins = 7) +
  labs(
    x = "Value Smc",
    y = "Frequency"
  )
```

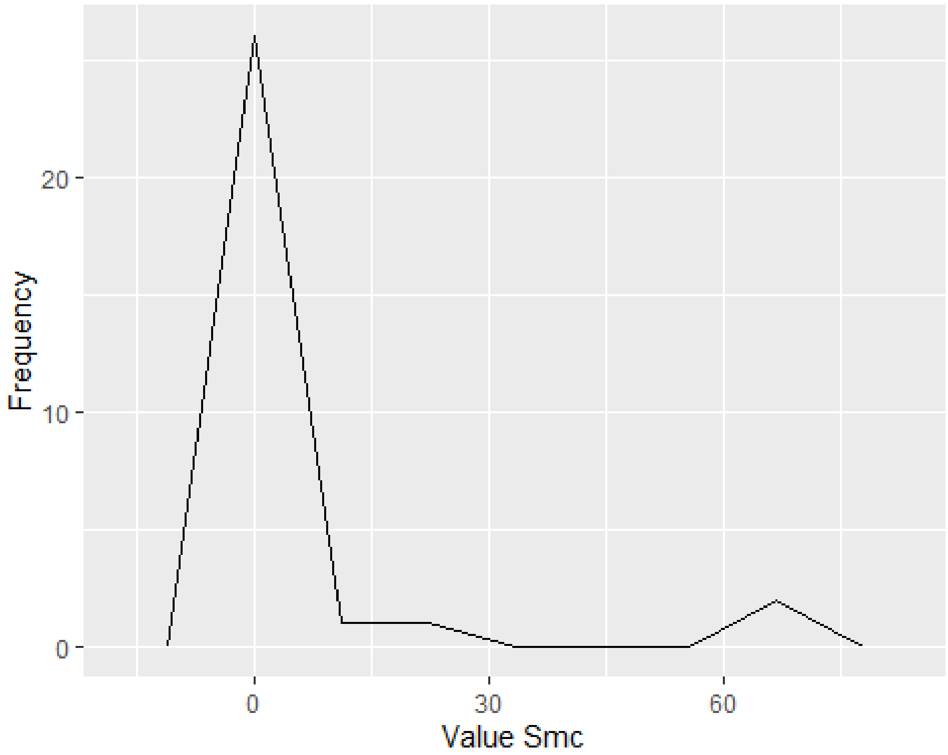


```
ggplot(I) +
  geom_freqpoly(aes(x = HAsfc9), bins = 7) +
  labs(
    x = "Value H9",
    y = "Frequency"
  )
```

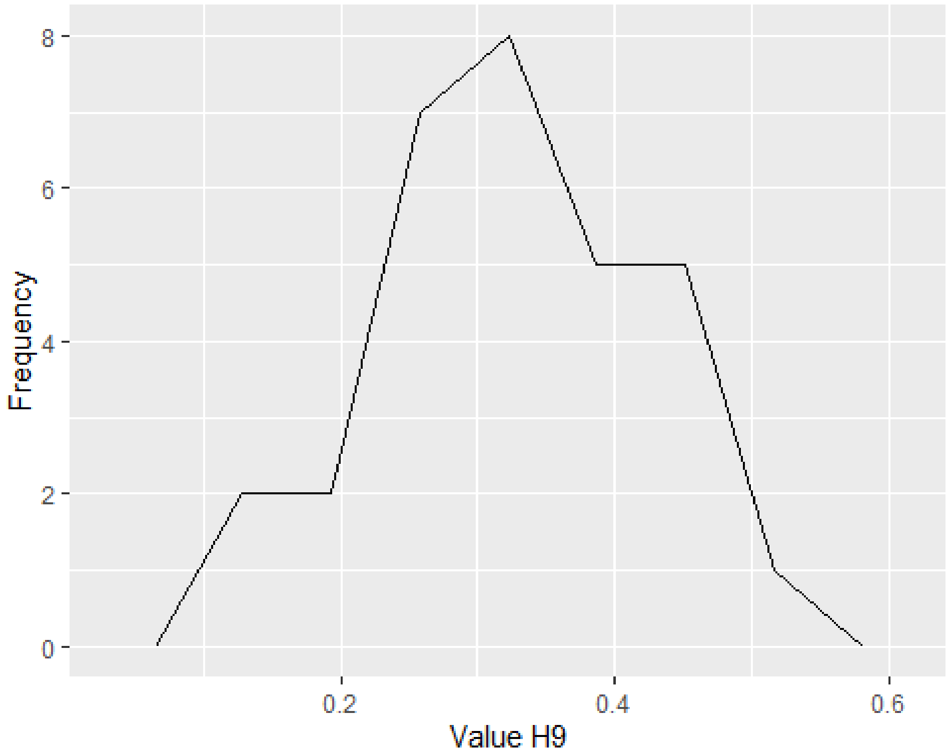


```
ggplot(I) +
  geom_freqpoly(aes(x = HAsfc36), bins = 7) +
  labs(
    x = "Value H36",
    y = "Frequency"
  )
```

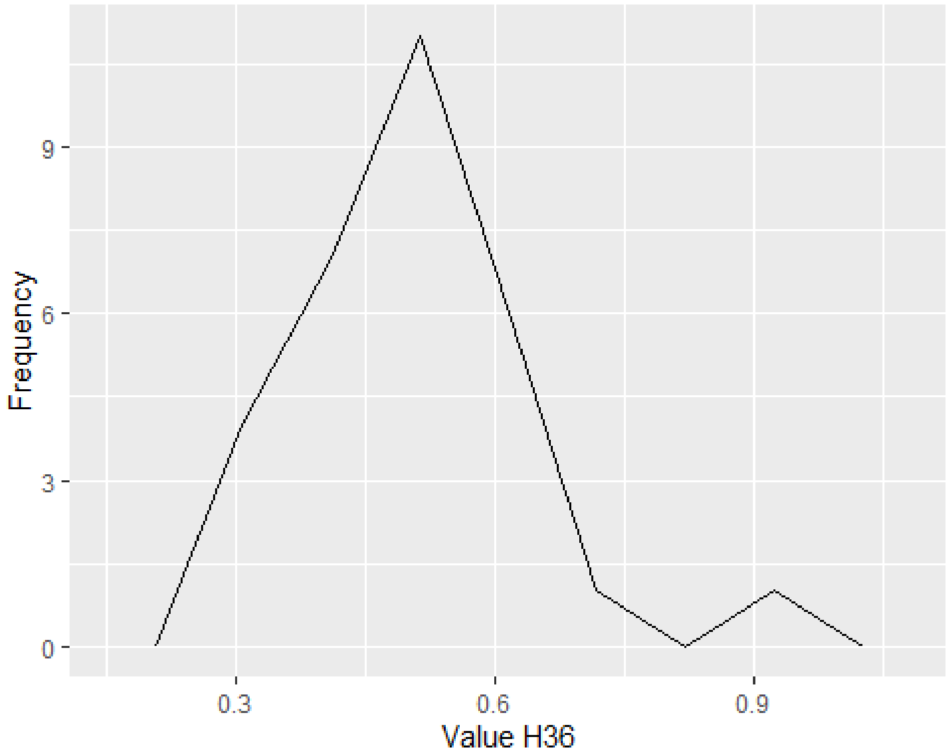


```
ggplot(I) +
  geom_freqpoly(aes(x = HAsfc81), bins = 7) +
  labs(
    x = "Value H81",
    y = "Frequency"
  )
```

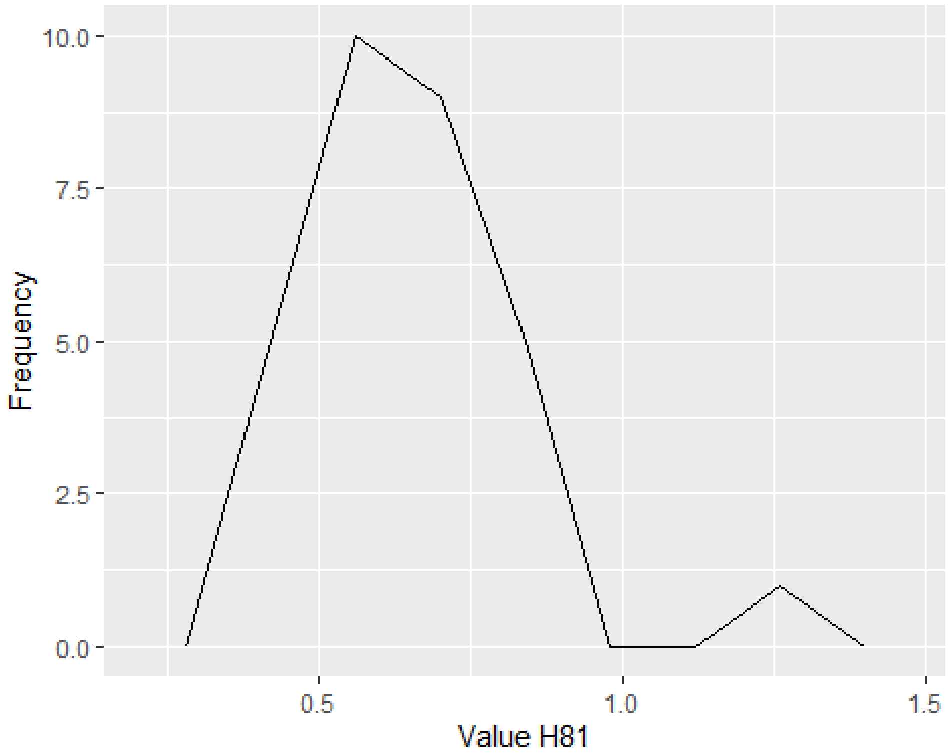

Homoscedasticity: Brown & Forsythe test (and data transformation whenever needed)

```
bf.test(I_Asfc ~ I_Species, data = I)

##
##   Brown-Forsythe Test (alpha = 0.05)
## -------------------------------------------------------------
##   data : I_Asfc and I_Species
##
##   statistic  : 1.727292
##   num df     : 1
##   denom df   : 12.37468
##   p.value    : 0.2125974
##
##   Result     : Difference is not statistically significant.
## -------------------------------------------------------------

ggplot(I) +
  geom_boxplot(aes(x = I_Species, y = I_Asfc)) +
  labs(
    x = "Species",
    y = "Asfc"
  )
```

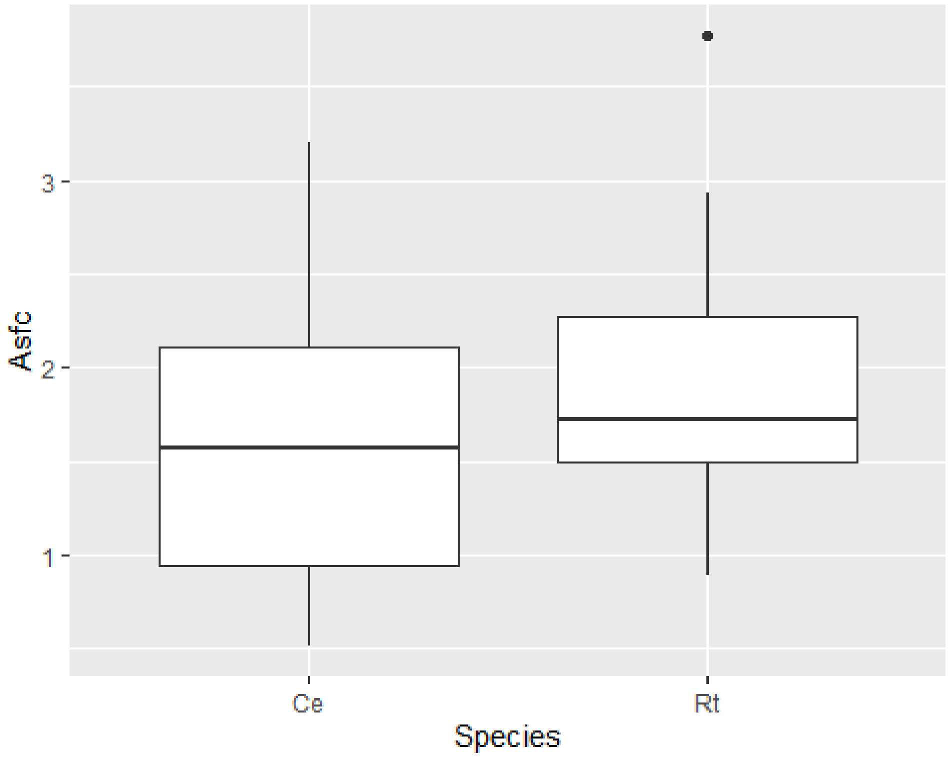


```
bf.test(I_epLsar ~ I_Species, data = I)

##
##   Brown-Forsythe Test (alpha = 0.05)
## -------------------------------------------------------------
##   data : I_epLsar and I_Species
##
##   statistic  : 0.09574845
##   num df     : 1
##   denom df   : 15.08519
##   p.value    : 0.7612256
##
##   Result     : Difference is not statistically significant.
## -------------------------------------------------------------

ggplot(I) +
  geom_boxplot(aes(x = I_Species, y = I_epLsar)) +
  labs(
    x = "Species",
    y = "epLsar"
  )
```

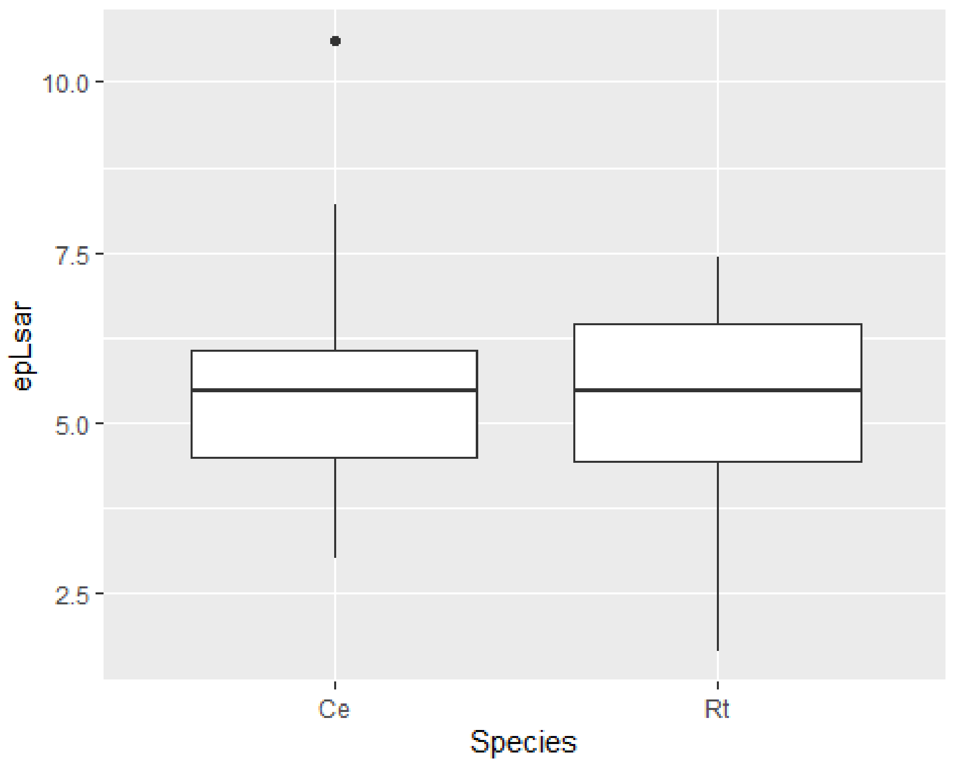


```
bf.test(I_Smc ~ I_Species, data = I)

##
##   Brown-Forsythe Test (alpha = 0.05)
## -------------------------------------------------------------
##   data : I_Smc and I_Species
##
##   statistic  : 2.293306
##   num df     : 1
##   denom df   : 20.63276
##   p.value    : 0.1451025
##
##   Result     : Difference is not statistically significant.
## -------------------------------------------------------------

ggplot(I) +
  geom_boxplot(aes(x = I_Species, y = I_Smc)) +
  labs(
    x = "Species",
    y = "Smc"
  )
```

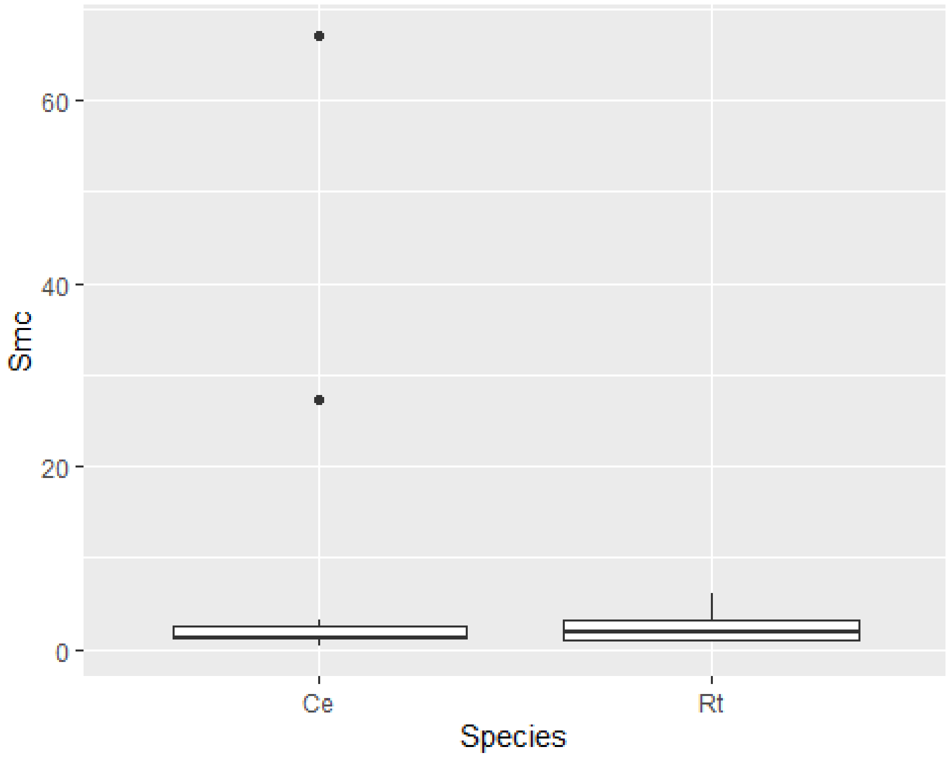


```
bf.test(I_H9 ~ I_Species, data = H)

##
##   Brown-Forsythe Test (alpha = 0.05)
## -------------------------------------------------------------
##   data : I_H9 and I_Species
##
##   statistic  : 0.4681133
##   num df     : 1
##   denom df   : 11.17105
##   p.value    : 0.5078016
##
##   Result     : Difference is not statistically significant.
## -------------------------------------------------------------

ggplot(I) +
  geom_boxplot(aes(x = I_Species, y = I_H9)) +
  labs(
    x = "Species",
    y = "H9"
  )
```

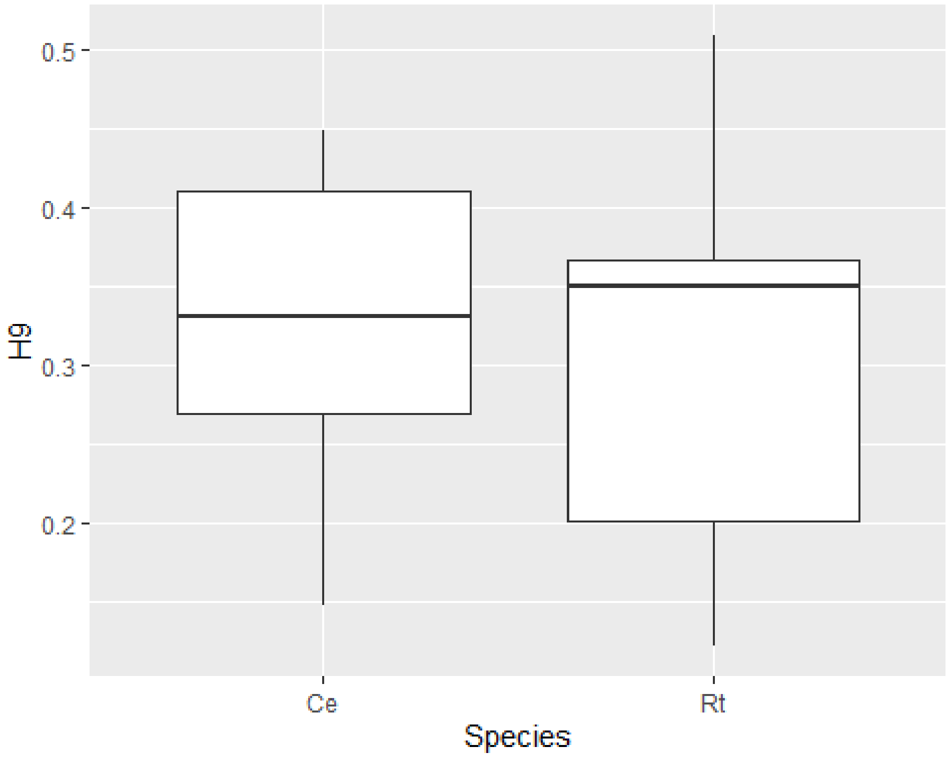


```
bf.test(I_H36 ~ I_Species, data = I)

##
##   Brown-Forsythe Test (alpha = 0.05)
## -------------------------------------------------------------
##   data : I_H36 and I_Species
##
##   statistic  : 0.004144098
##   num df     : 1
##   denom df   : 10.02772
##   p.value    : 0.9499371
##
##   Result     : Difference is not statistically significant.
## -------------------------------------------------------------

ggplot(I) +
  geom_boxplot(aes(x = I_Species, y = I_H36)) +
  labs(
    x = "Species",
    y = "H36"
  )
```

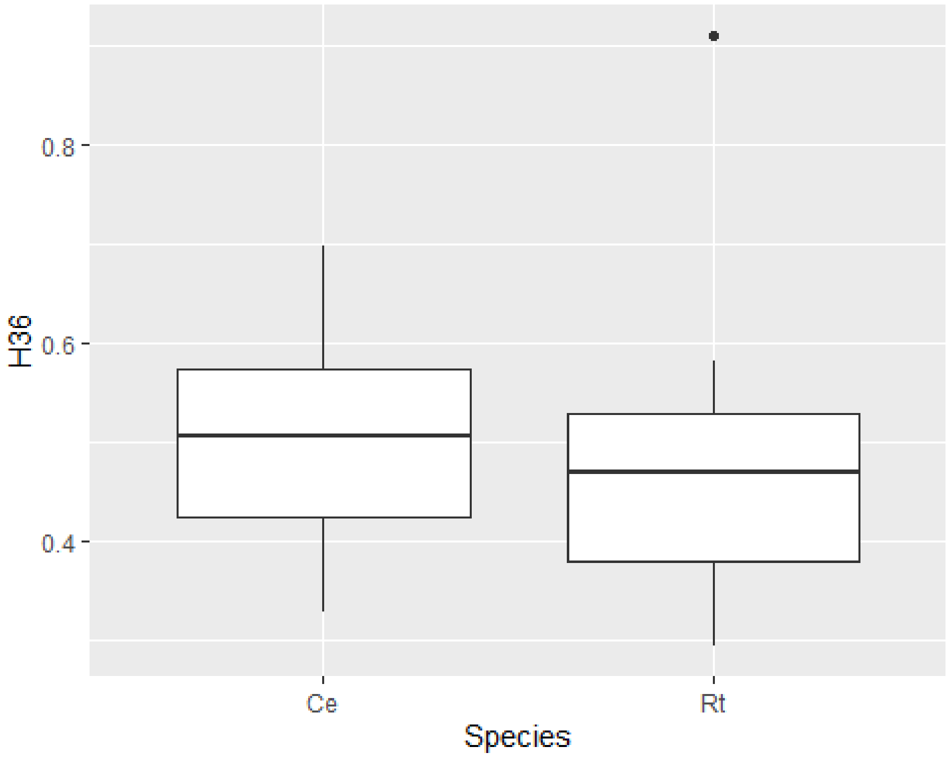


```
bf.test(I_H81 ~ I_Species, data = I)

##
##   Brown-Forsythe Test (alpha = 0.05)
## -------------------------------------------------------------
##   data : I_H81 and I_Species
##
##   statistic  : 0.2651944
##   num df     : 1
##   denom df   : 9.95608
##   p.value    : 0.6178126
##
##   Result     : Difference is not statistically significant.
## -------------------------------------------------------------

ggplot(I) +
  geom_boxplot(aes(x = I_Species, y = I_H81)) +
  labs(
    x = "Species",
    y = "H81"
  )
```

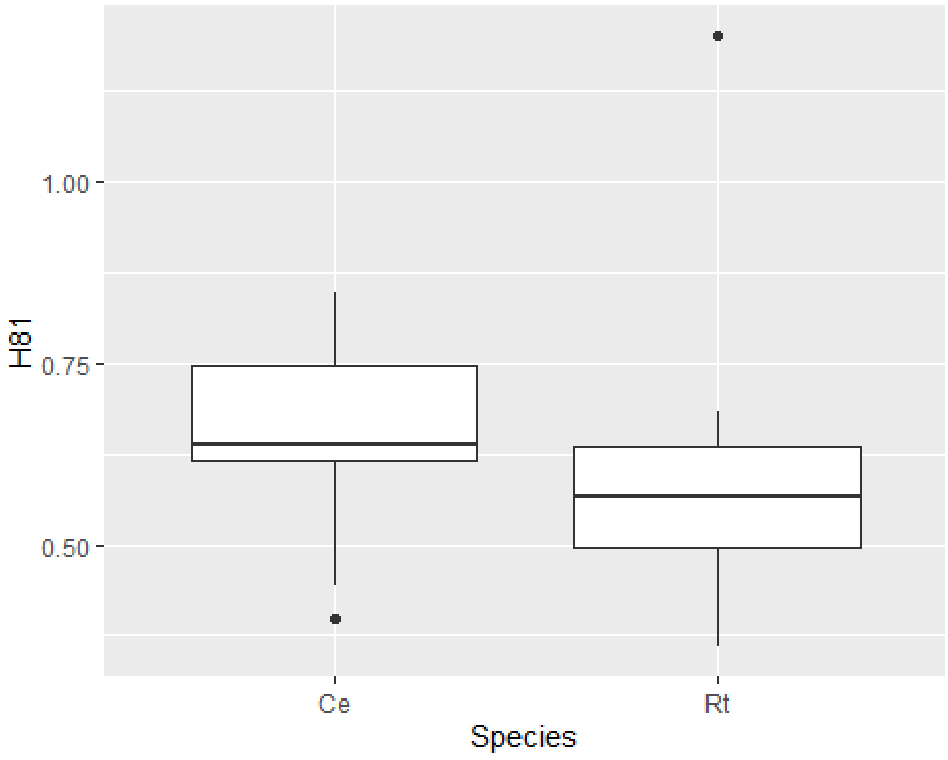

Glm: Impact of blocks over each DMTA parameter

```
glm_I_Asfc0 <- glm(I_Asfc ~ 1, data = I)
glm_I_Asfc1 <- glm(I_Asfc ~ I_Species, data = I)
Cand.models <- list()
Cand.models[[1]] <- glm_I_Asfc0
Cand.models[[2]] <- glm_I_Asfc1
Modnames <- lapply(Cand.models, "formula")
aictab(cand.set = Cand.models, modnames = paste0(Modnames), sort = TRUE)

##
## Model selection based on AICc:
##
##                       K  AICc Delta_AICc AICcWt Cum.Wt     LL
## I_Asfc ~ 1            2 72.91       0.00   0.54   0.54 -34.23
## I_Asfc ~ I_Species 3 73.20       0.29   0.46   1.00 -33.14

summary(glm_I_Asfc1)

##
## Call:
## glm(formula = I_Asfc ~ I_Species, data = I)
##
## Deviance Residuals:
##     Min       1Q   Median       3Q      Max
## -1.1072  -0.5887  -0.0800   0.4778   1.7738
##
## Coefficients:
```

```
##              Estimate Std. Error t value Pr(>|t|)
## (Intercept)   1.5640     0.1650   9.481 3.08e-10 ***
## I_SpeciesRt   0.4382     0.3012   1.455    0.157
## ---
## Signif. codes:  0 '***' 0.001 '**' 0.01 '*' 0.05 '.' 0.1 ' ' 1
##
## (Dispersion parameter for gaussian family taken to be 0.5714418)
##
##     Null deviance: 17.21  on 29  degrees of freedom
## Residual deviance: 16.00  on 28  degrees of freedom
## AIC: 72.279
##
## Number of Fisher Scoring iterations: 2
```

```
glm_I_epLsar0 <- glm(I_epLsar ~ 1, data = I)
glm_I_epLsar1 <- glm(I_epLsar ~ I_Species, data = I)
Cand.models <- list()
Cand.models[[1]] <- glm_I_epLsar0
Cand.models[[2]] <- glm_I_epLsar1
Modnames <- lapply(Cand.models, "formula")
aictab(cand.set = Cand.models, modnames = paste0(Modnames), sort = TRUE)
```

```
##
## Model selection based on AICc:
##
##                       K   AICc Delta_AICc AICcWt Cum.Wt     LL
## I_epLsar ~ 1          2 122.78       0.00   0.77   0.77 -59.17
## I_epLsar ~ I_Species 3 125.16       2.38   0.23   1.00 -59.12
```

```
glm_I_Smc0 <- glm(I_Smc ~ 1, data = I)
glm_I_Smc1 <- glm(I_Smc ~ I_Species, data = I)
Cand.models <- list()
Cand.models[[1]] <- glm_I_Smc0
Cand.models[[2]] <- glm_I_Smc1
Modnames <- lapply(Cand.models, "formula")
aictab(cand.set = Cand.models, modnames = paste0(Modnames), sort = TRUE)
```

```
##
## Model selection based on AICc:
##
##                   K   AICc Delta_AICc AICcWt Cum.Wt      LL
## I_Smc ~ 1         2 258.57       0.00   0.67   0.67 -127.06
## I_Smc ~ I_Species 3 260.02       1.45   0.33   1.00 -126.55
```

```
glm_I_H9_0 <- glm(I_H9 ~ 1, data = I)
glm_I_H9_1 <- glm(I_H9 ~ I_Species, data = I)
Cand.models <- list()
Cand.models[[1]] <- glm_I_H9_0
Cand.models[[2]] <- glm_I_H9_1
Modnames <- lapply(Cand.models, "formula")
aictab(cand.set = Cand.models, modnames = paste0(Modnames), sort = TRUE)
```

```
##
## Model selection based on AICc:
##
##                   K   AICc Delta_AICc AICcWt Cum.Wt    LL
## I_H9 ~ 1          2 -52.82       0.00   0.71   0.71 28.63
## I_H9 ~ I_Species 3 -51.03       1.79   0.29   1.00 28.98
```

```
glm_I_H36_0 <- glm(I_H36 ~ 1, data = I)
glm_I_H36_1 <- glm(I_H36 ~ I_Species, data = I)
Cand.models <- list()
Cand.models[[1]] <- glm_I_H36_0
Cand.models[[2]] <- glm_I_H36_1
Modnames <- lapply(Cand.models, "formula")
aictab(cand.set = Cand.models, modnames = paste0(Modnames), sort = TRUE)
```

```
##
## Model selection based on AICc:
##
##                    K   AICc Delta_AICc AICcWt Cum.Wt    LL
## I_H36 ~ 1          2 -36.47       0.00   0.77   0.77 20.46
## I_H36 ~ I_Species 3 -33.99       2.47   0.23   1.00 20.46
```

```
glm_I_H81_0 <- glm(I_H81 ~ 1, data = I)
glm_I_H81_1 <- glm(I_H81 ~ I_Species, data = I)
Cand.models <- list()
Cand.models[[1]] <- glm_I_H81_0
Cand.models[[2]] <- glm_I_H81_1
Modnames <- lapply(Cand.models, "formula")
aictab(cand.set = Cand.models, modnames = paste0(Modnames), sort = TRUE)
```

```
##
## Model selection based on AICc:
##
##                    K   AICc Delta_AICc AICcWt Cum.Wt    LL
## I_H81 ~ 1          2 -18.87       0.00   0.73   0.73 11.66
## I_H81 ~ I_Species 3 -16.85       2.02   0.27   1.00 11.89
```

Inter-specific differences between contemporaneous ungulates (per period)

```
AnteQ <- subset(db_FOSSILS, db_FOSSILS$Period == "AnteQ")
Quina <- subset(db_FOSSILS, db_FOSSILS$Period == "Quina")
PostQ <- subset(db_FOSSILS, db_FOSSILS$Period == "PostQ")

AnteQ_species <- AnteQ %>%
  dplyr::select(c(2)) %>%
  unlist(c(1))
AnteQ_Asfc <- AnteQ %>%
  dplyr::select(c(7)) %>%
  unlist(c(1))
AnteQ_epLsar <- AnteQ %>%
  dplyr::select(c(8)) %>%
  unlist(c(1))
AnteQ_Smc <- AnteQ %>%
  dplyr::select(c(9)) %>%
  unlist(c(1))
AnteQ_H9 <- AnteQ %>%
  dplyr::select(c(10)) %>%
  unlist(c(1))
AnteQ_H36 <- AnteQ %>%
  dplyr::select(c(12)) %>%
  unlist(c(1))
AnteQ_H81 <- AnteQ %>%
  dplyr::select(c(11)) %>%
  unlist(c(1))

Quina_species <- Quina %>%
  dplyr::select(c(2)) %>%
  unlist(c(1))
Quina_Asfc <- Quina %>%
  dplyr::select(c(7)) %>%
  unlist(c(1))
Quina_epLsar <- Quina %>%
  dplyr::select(c(8)) %>%
  unlist(c(1))
Quina_Smc <- Quina %>%
  dplyr::select(c(9)) %>%
  unlist(c(1))
Quina_H9 <- Quina %>%
  dplyr::select(c(10)) %>%
  unlist(c(1))
Quina_H36 <- Quina %>%
  dplyr::select(c(12)) %>%
  unlist(c(1))
Quina_H81 <- Quina %>%
  dplyr::select(c(11)) %>%
  unlist(c(1))
```

```r
PostQ_species <- PostQ %>%
  dplyr::select(c(2)) %>%
  unlist(c(1))
PostQ_Asfc <- PostQ %>%
  dplyr::select(c(7)) %>%
  unlist(c(1))
PostQ_epLsar <- PostQ %>%
  dplyr::select(c(8)) %>%
  unlist(c(1))
PostQ_Smc <- PostQ %>%
  dplyr::select(c(9)) %>%
  unlist(c(1))
PostQ_H9 <- PostQ %>%
  dplyr::select(c(10)) %>%
  unlist(c(1))
PostQ_H36 <- PostQ %>%
  dplyr::select(c(12)) %>%
  unlist(c(1))
PostQ_H81 <- PostQ %>%
  dplyr::select(c(11)) %>%
  unlist(c(1))

PostQ_Period <- PostQ %>%
  dplyr::select(c(3)) %>%
  unlist(c(1))
```

Homoscedasticity: Brown & Forsythe test (and data transformation whenever needed)

```r
bf.test(AnteQ_Asfc ~ AnteQ_species, data = AnteQ)

##
##   Brown-Forsythe Test (alpha = 0.05)
## -------------------------------------------------------------
##   data : AnteQ_Asfc and AnteQ_species
##
##   statistic  : 3.579516
##   num df     : 2
##   denom df   : 13.85899
##   p.value    : 0.05581227
##
##   Result     : Difference is not statistically significant.
## -------------------------------------------------------------

ggplot(AnteQ) +
  geom_boxplot(aes(x = AnteQ_species, y = AnteQ_Asfc)) +
  labs(
    x = "Species",
    y = "Asfc"
  )
```

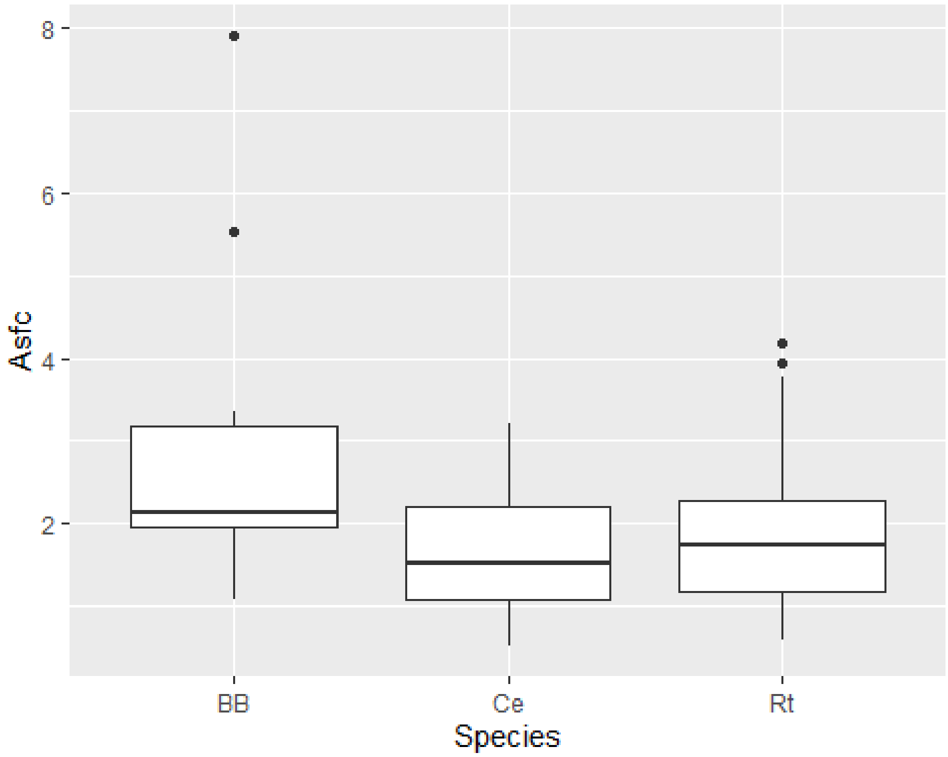


```
bf.test(AnteQ_epLsar ~ AnteQ_species, data = AnteQ)

## 
##   Brown-Forsythe Test (alpha = 0.05) 
## ------------------------------------------------------------- 
##   data : AnteQ_epLsar and AnteQ_species 
## 
##   statistic  : 0.8893147 
##   num df     : 2 
##   denom df   : 37.05611 
##   p.value    : 0.4195254 
## 
##   Result     : Difference is not statistically significant. 
## -------------------------------------------------------------

ggplot(AnteQ) +
  geom_boxplot(aes(x = AnteQ_species, y = AnteQ_epLsar)) +
  labs(
    x = "Species",
    y = "epLsar"
  )
```

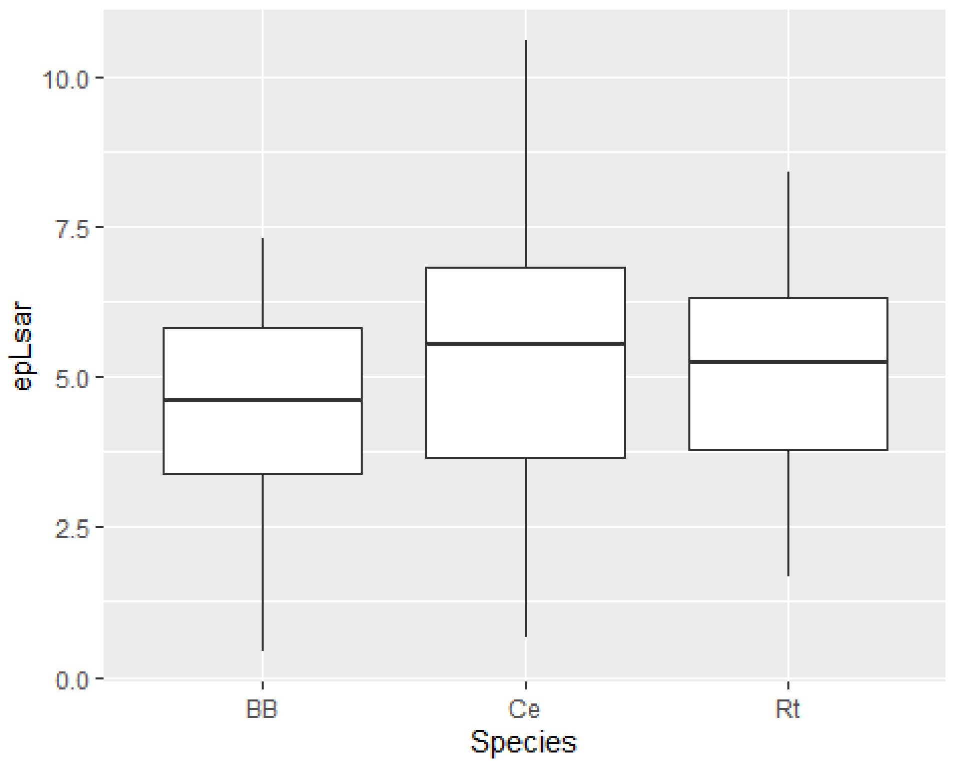


```
bf.test(AnteQ_Smc ~ AnteQ_species, data = AnteQ)

##
##   Brown-Forsythe Test (alpha = 0.05)
## -------------------------------------------------------------
##   data : AnteQ_Smc and AnteQ_species
##
##   statistic  : 3.123225
##   num df     : 2
##   denom df   : 50.43345
##   p.value    : 0.052632
##
##   Result     : Difference is not statistically significant.
## -------------------------------------------------------------

ggplot(AnteQ) +
  geom_boxplot(aes(x = AnteQ_species, y = AnteQ_Smc)) +
  labs(
    x = "Species",
    y = "Smc"
  )
```

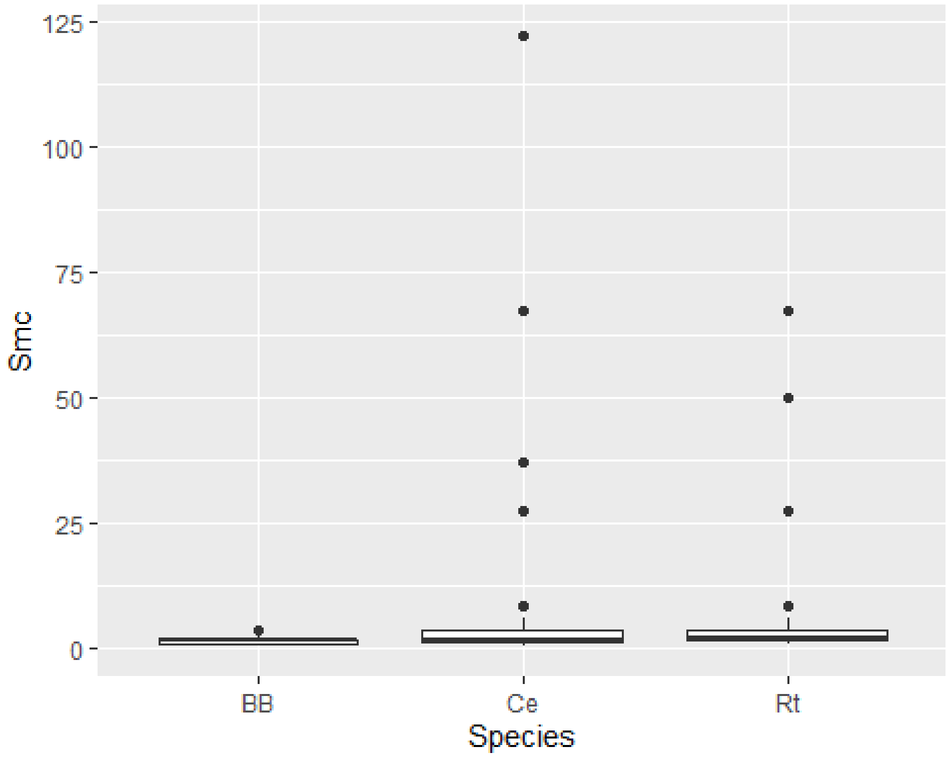


```
bf.test(AnteQ_H9 ~ AnteQ_species, data = AnteQ)

##
##   Brown-Forsythe Test (alpha = 0.05)
## -------------------------------------------------------------
##   data : AnteQ_H9 and AnteQ_species
##
##   statistic  : 1.538256
##   num df     : 2
##   denom df   : 57.75058
##   p.value    : 0.2234244
##
##   Result     : Difference is not statistically significant.
## -------------------------------------------------------------

ggplot(AnteQ) +
  geom_boxplot(aes(x = AnteQ_species, y = AnteQ_H9)) +
  labs(
    x = "Species",
    y = "H9"
  )
```

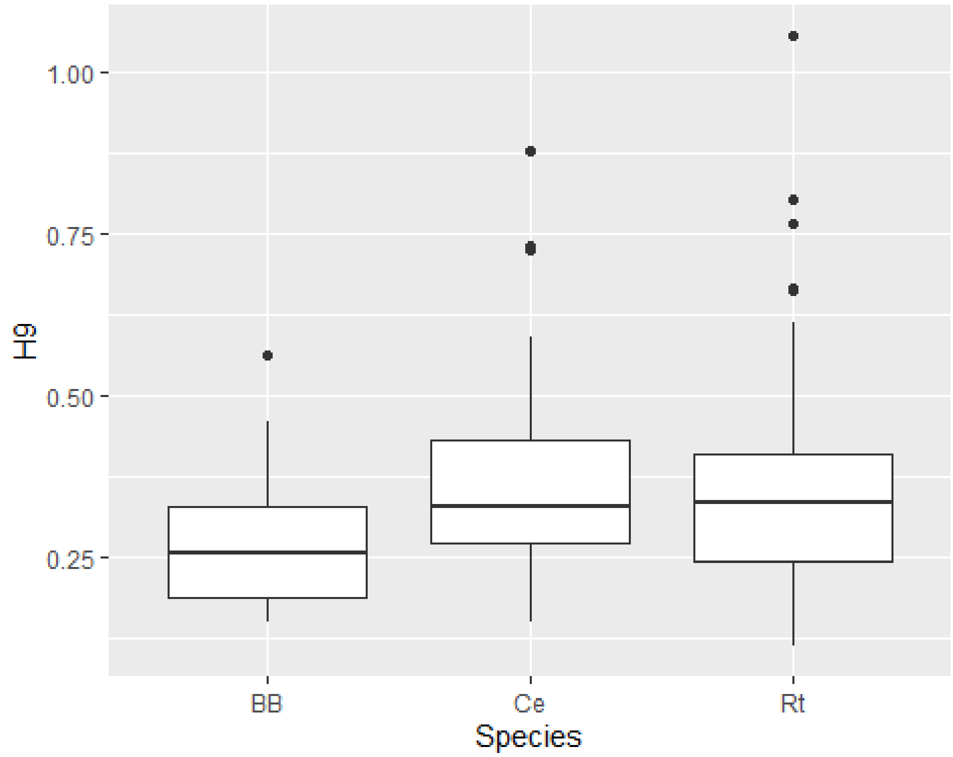


```
bf.test(AnteQ_H36 ~ AnteQ_species, data = AnteQ)

##
##   Brown-Forsythe Test (alpha = 0.05)
## -------------------------------------------------------------
##   data : AnteQ_H36 and AnteQ_species
##
##   statistic  : 1.197204
##   num df     : 2
##   denom df   : 98.96876
##   p.value    : 0.3063732
##
##   Result     : Difference is not statistically significant.
## -------------------------------------------------------------

ggplot(AnteQ) +
  geom_boxplot(aes(x = AnteQ_species, y = AnteQ_H36)) +
  labs(
    x = "Species",
    y = "H36"
  )
```

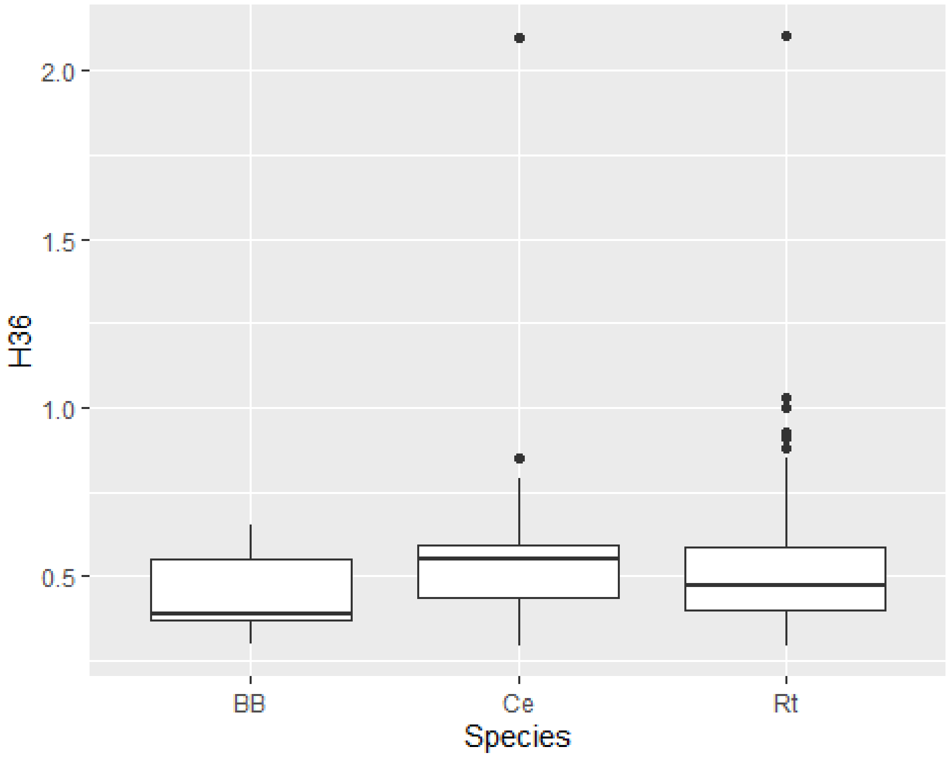


```
bf.test(AnteQ_H81 ~ AnteQ_species, data = AnteQ)

## 
##   Brown-Forsythe Test (alpha = 0.05) 
## ------------------------------------------------------------- 
##   data : AnteQ_H81 and AnteQ_species 
## 
##   statistic  : 1.590525 
##   num df     : 2 
##   denom df   : 77.88925 
##   p.value    : 0.2103665 
## 
##   Result     : Difference is not statistically significant. 
## -------------------------------------------------------------

ggplot(AnteQ) +
  geom_boxplot(aes(x = AnteQ_species, y = AnteQ_H81)) +
  labs(
    x = "Species",
    y = "H81"
  )
```

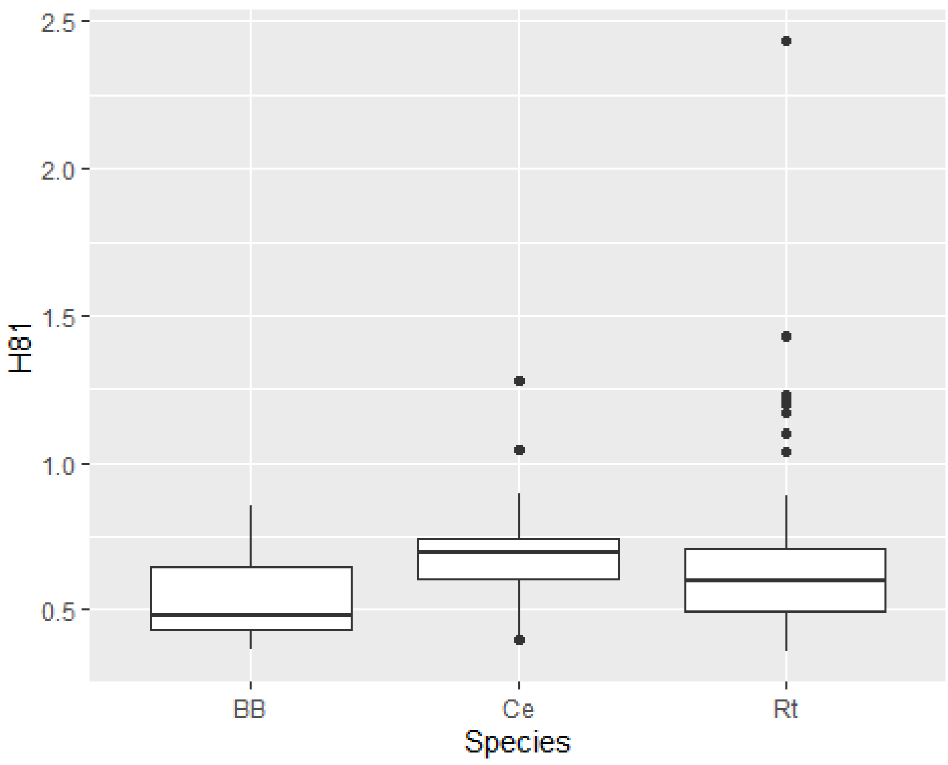


```
bf.test(PostQ_Asfc ~ PostQ_species, data = PostQ)

##
##   Brown-Forsythe Test (alpha = 0.05)
## -------------------------------------------------------------
##   data : PostQ_Asfc and PostQ_species
##
##   statistic  : 1.814291
##   num df     : 2
##   denom df   : 96.37847
##   p.value    : 0.1684744
##
##   Result     : Difference is not statistically significant.
## -------------------------------------------------------------

ggplot(PostQ) +
  geom_boxplot(aes(x = PostQ_species, y = PostQ_Asfc)) +
  labs(
    x = "Species",
    y = "Asfc"
  )
```

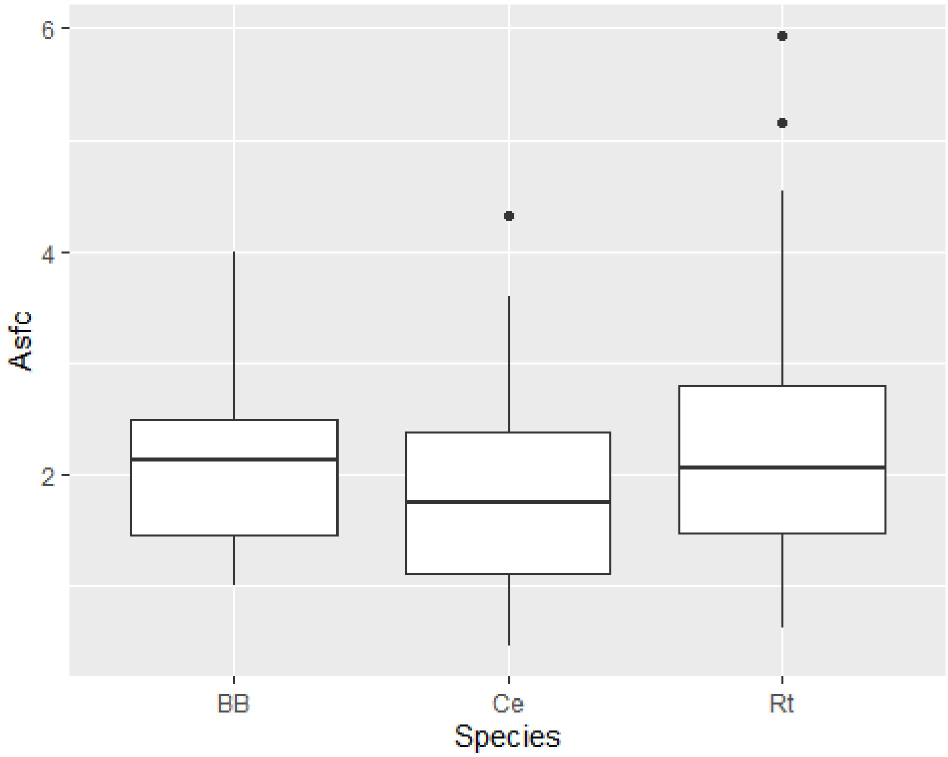


```
bf.test(PostQ_epLsar ~ PostQ_species, data = PostQ)

## 
##   Brown-Forsythe Test (alpha = 0.05) 
## ------------------------------------------------------------- 
##   data : PostQ_epLsar and PostQ_species 
## 
##   statistic  : 2.58016 
##   num df     : 2 
##   denom df   : 89.62576 
##   p.value    : 0.08138088 
## 
##   Result     : Difference is not statistically significant. 
## -------------------------------------------------------------

ggplot(PostQ) +
  geom_boxplot(aes(x = PostQ_species, y = PostQ_epLsar)) +
  labs(
    x = "Species",
    y = "epLsar"
  )
```

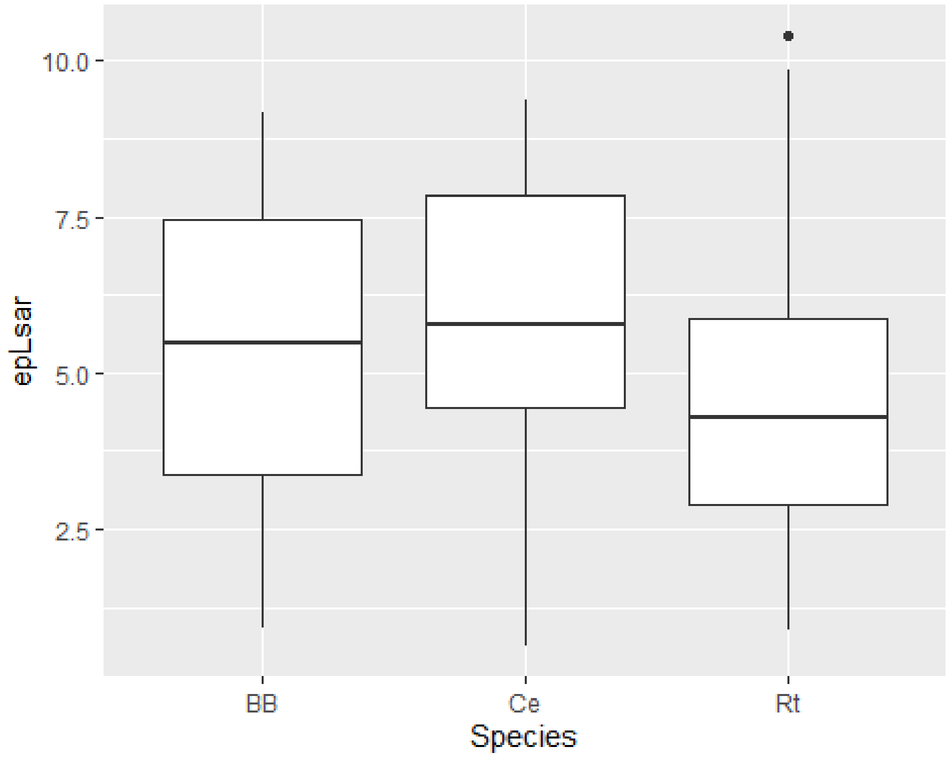


```
bf.test(PostQ_Smc ~ PostQ_species, data = PostQ)

##
##   Brown-Forsythe Test (alpha = 0.05)
## -------------------------------------------------------------
##   data : PostQ_Smc and PostQ_species
##
##   statistic  : 1.844441
##   num df     : 2
##   denom df   : 40.20559
##   p.value    : 0.1712432
##
##   Result     : Difference is not statistically significant.
## -------------------------------------------------------------

ggplot(PostQ) +
  geom_boxplot(aes(x = PostQ_species, y = PostQ_Smc)) +
  labs(
    x = "Species",
    y = "Smc"
  )
```

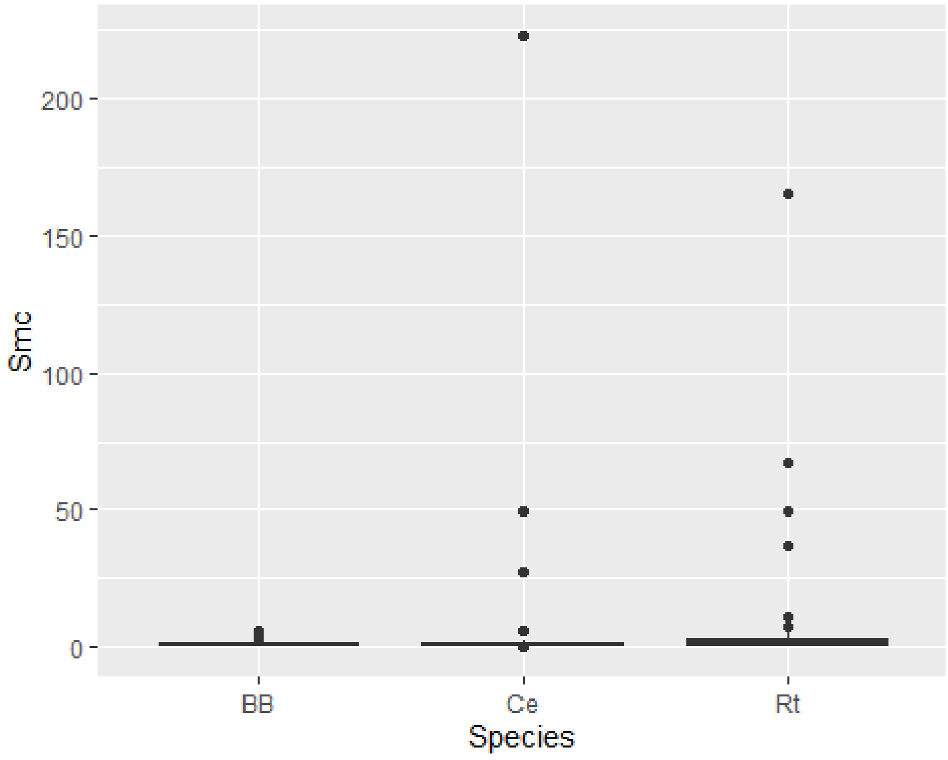


```
bf.test(PostQ_H9 ~ PostQ_species, data = PostQ)

##
##   Brown-Forsythe Test (alpha = 0.05)
## -------------------------------------------------------------
##   data : PostQ_H9 and PostQ_species
##
##   statistic  : 1.167621
##   num df     : 2
##   denom df   : 89.34894
##   p.value    : 0.3158071
##
##   Result     : Difference is not statistically significant.
## -------------------------------------------------------------

ggplot(PostQ) +
  geom_boxplot(aes(x = PostQ_species, y = PostQ_H9)) +
  labs(
    x = "Species",
    y = "H9"
  )
```

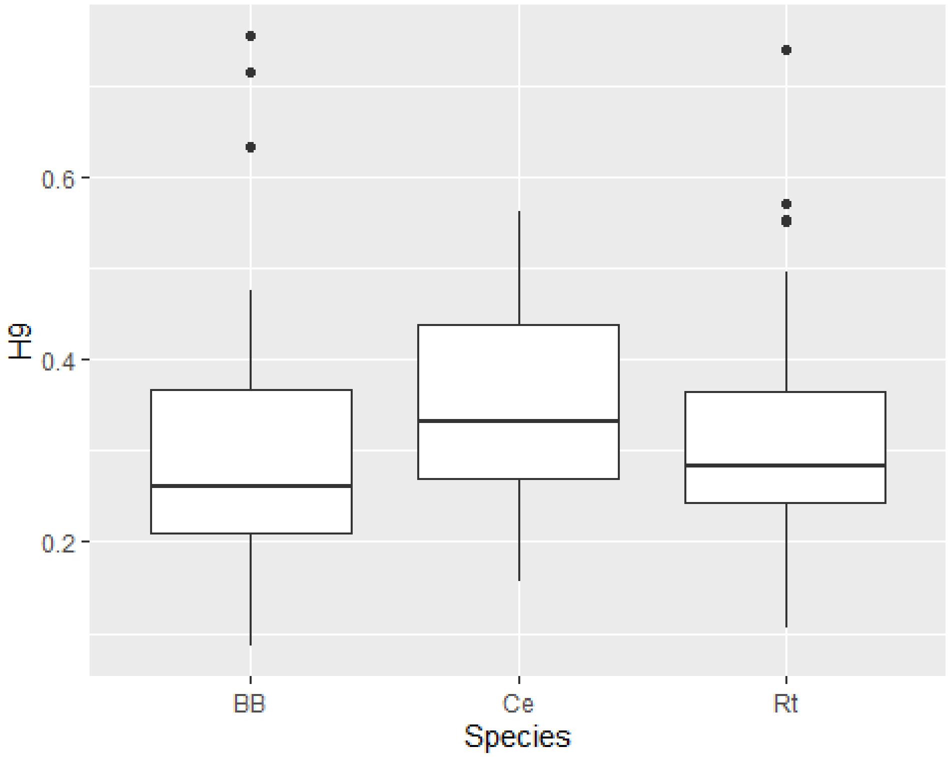


```
bf.test(PostQ_H36 ~ PostQ_species, data = PostQ)

##
##   Brown-Forsythe Test (alpha = 0.05)
## -------------------------------------------------------------
##   data : PostQ_H36 and PostQ_species
##
##   statistic  : 0.8569485
##   num df     : 2
##   denom df   : 102.1576
##   p.value    : 0.4274836
##
##   Result     : Difference is not statistically significant.
## -------------------------------------------------------------

ggplot(PostQ) +
  geom_boxplot(aes(x = PostQ_species, y = PostQ_H36)) +
  labs(
    x = "Species",
    y = "H36"
  )
```

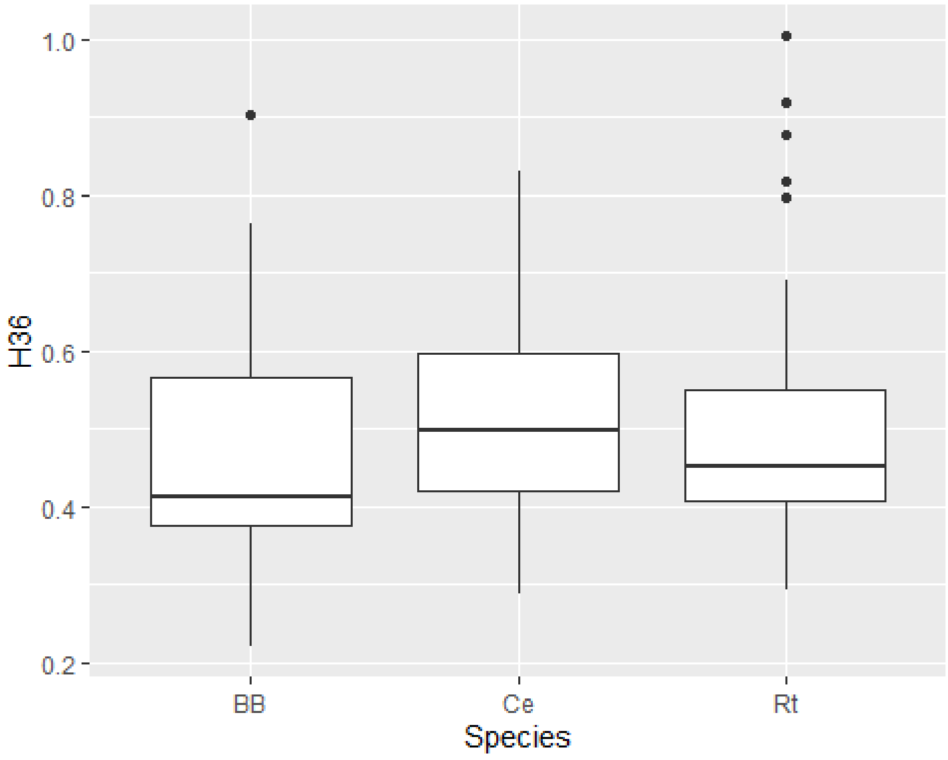


```
bf.test(PostQ_H81 ~ PostQ_species, data = PostQ)

##
##   Brown-Forsythe Test (alpha = 0.05)
## -------------------------------------------------------------
##   data : PostQ_H81 and PostQ_species
##
##   statistic  : 1.059646
##   num df     : 2
##   denom df   : 103.2374
##   p.value    : 0.3503173
##
##   Result     : Difference is not statistically significant.
## -------------------------------------------------------------

ggplot(PostQ) +
  geom_boxplot(aes(x = PostQ_species, y = PostQ_H36)) +
  labs(
    x = "Species",
    y = "H81"
  )
```

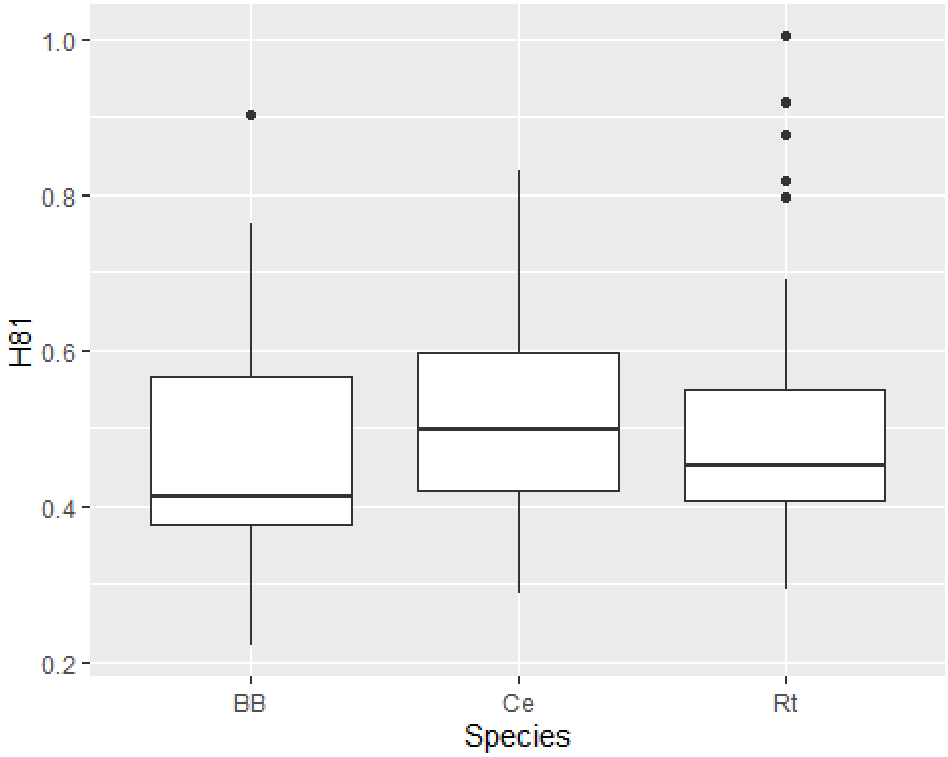


```
bf.test(Quina_Asfc ~ Quina_species, data = Quina)

##
##   Brown-Forsythe Test (alpha = 0.05)
## -------------------------------------------------------------
##   data : Quina_Asfc and Quina_species
##
##   statistic  : 2.335185
##   num df     : 2
##   denom df   : 5.366162
##   p.value    : 0.1863875
##
##   Result     : Difference is not statistically significant.
## -------------------------------------------------------------

ggplot(Quina) +
  geom_boxplot(aes(x = Quina_species, y = Quina_Asfc)) +
  labs(
    x = "Species",
    y = "Asfc"
  )
```

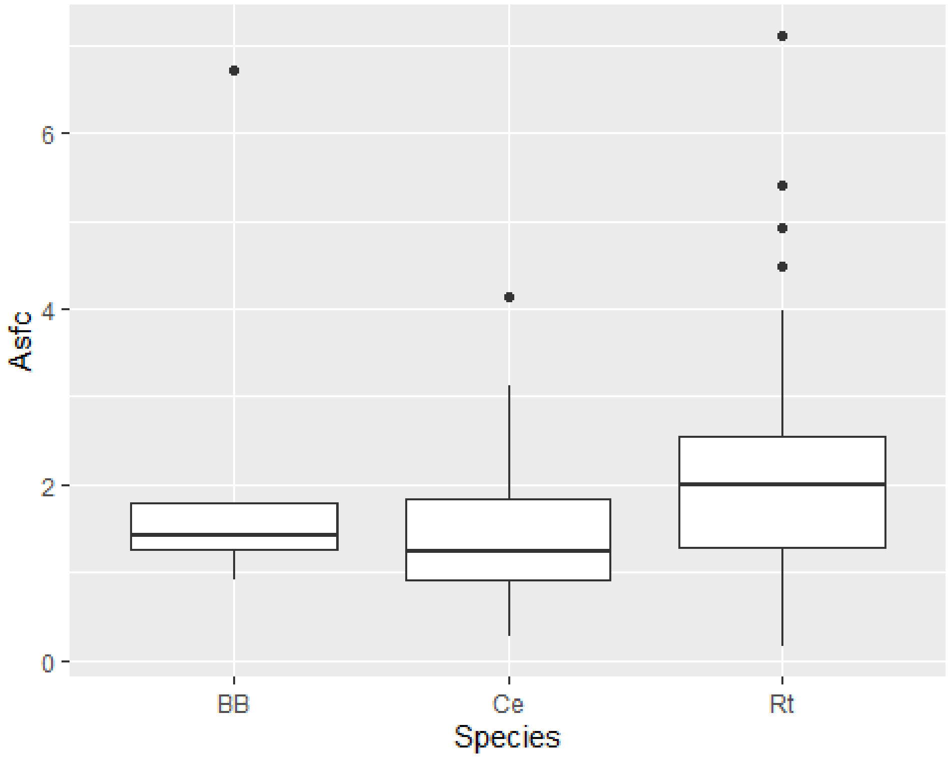


```
bf.test(Quina_epLsar ~ Quina_species, data = Quina)

##
##   Brown-Forsythe Test (alpha = 0.05)
## -------------------------------------------------------------
##   data : Quina_epLsar and Quina_species
##
##   statistic  : 2.337017
##   num df     : 2
##   denom df   : 8.643054
##   p.value    : 0.1544073
##
##   Result     : Difference is not statistically significant.
## -------------------------------------------------------------

ggplot(Quina) +
  geom_boxplot(aes(x = Quina_species, y = Quina_epLsar)) +
  labs(
    x = "Species",
    y = "epLsar"
  )
```

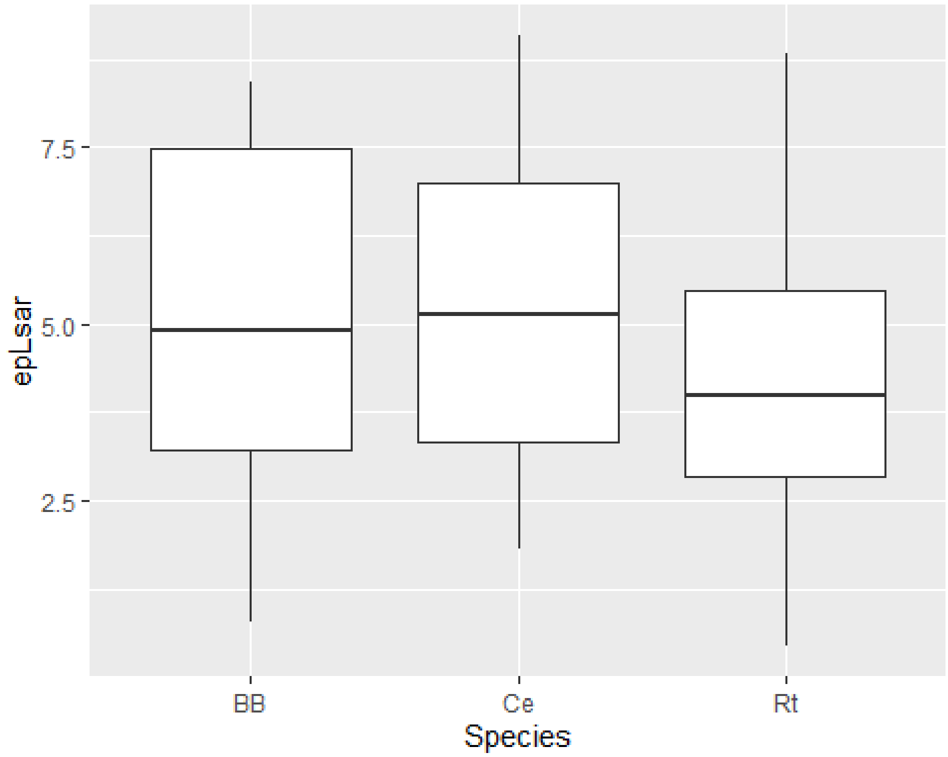


```
bf.test(Quina_Smc ~ Quina_species, data = Quina) # /!\ Value>0.05

##
##   Brown-Forsythe Test (alpha = 0.05)
## -------------------------------------------------------------
##   data : Quina_Smc and Quina_species
##
##   statistic  : 5.871552
##   num df     : 2
##   denom df   : 51.12821
##   p.value    : 0.005064647
##
##   Result     : Difference is statistically significant.
## -------------------------------------------------------------

logged <- log(Quina_Smc + 1)
bf.test(logged ~ Quina_species, data = Quina) # the variances remain signific
antly different

##
##   Brown-Forsythe Test (alpha = 0.05)
## -------------------------------------------------------------
##   data : logged and Quina_species
##
##   statistic  : 6.935182
##   num df     : 2
##   denom df   : 18.79625
```

```
##   p.value    : 0.005548056
##
##   Result     : Difference is statistically significant.
## -------------------------------------------------------------
```

```r
sqrted <- sqrt(Quina_Smc)
bf.test(sqrted ~ Quina_species, data = Quina) # the variances remain signific
antly different
```

```
##
##   Brown-Forsythe Test (alpha = 0.05)
## -------------------------------------------------------------
##   data : sqrted and Quina_species
##
##   statistic  : 6.983975
##   num df     : 2
##   denom df   : 26.3654
##   p.value    : 0.00368196
##
##   Result     : Difference is statistically significant.
## -------------------------------------------------------------
```

```r
logged10 <- log10(Quina_Smc)
bf.test(logged10 ~ Quina_species, data = Quina) # the variances remain signif
icantly different
```

```
##
##   Brown-Forsythe Test (alpha = 0.05)
## -------------------------------------------------------------
##   data : logged10 and Quina_species
##
##   statistic  : 6.863328
##   num df     : 2
##   denom df   : 19.95064
##   p.value    : 0.005392721
##
##   Result     : Difference is statistically significant.
## -------------------------------------------------------------
```

```r
GLM_Quina_Smc <- glm(formula = Quina_Smc ~ Quina_species, family = gaussian)
bc_Quina_Smc <- boxcox(GLM_Quina_Smc, lambda = seq(-3, 3))
```

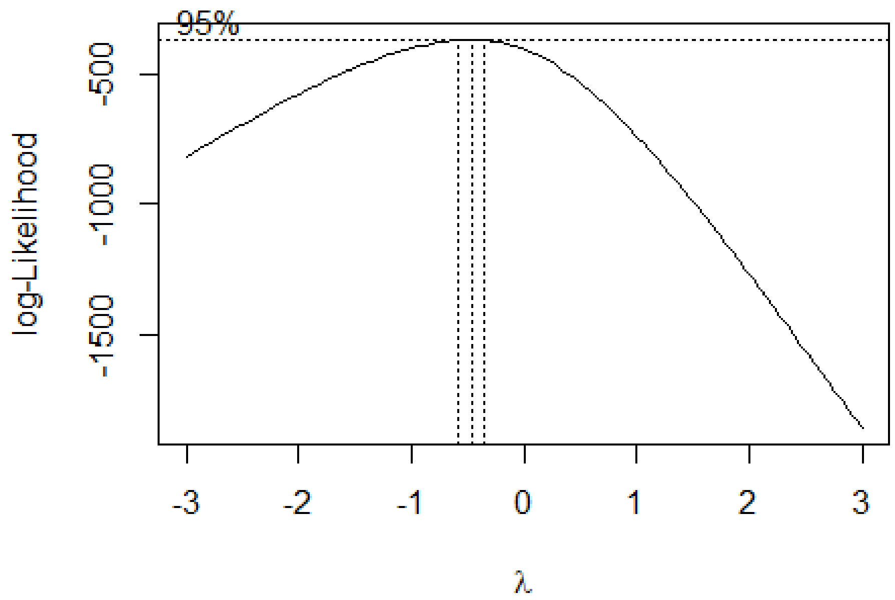


```
best.lam <- bc_Quina_Smc$x[which(bc_Quina_Smc$y == max(bc_Quina_Smc$y))]
best.lam # -0.4545455

## [1] -0.4545455

new_Quina_Smc <- (Quina_Smc)^-0.4545455
bf.test(new_Quina_Smc ~ Quina_species, data = Quina) # the variances remain s
ignificantly different

##
##   Brown-Forsythe Test (alpha = 0.05)
## -------------------------------------------------------------
##   data : new_Quina_Smc and Quina_species
##
##   statistic  : 5.990806
##   num df     : 2
##   denom df   : 26.38603
##   p.value    : 0.007160338
##
##   Result     : Difference is statistically significant.
## -------------------------------------------------------------

ggplot(Quina) +
  geom_boxplot(aes(x = Quina_species, y = new_Quina_Smc)) +
  labs(
    x = "Species",
    y = "Smc"
  )
```

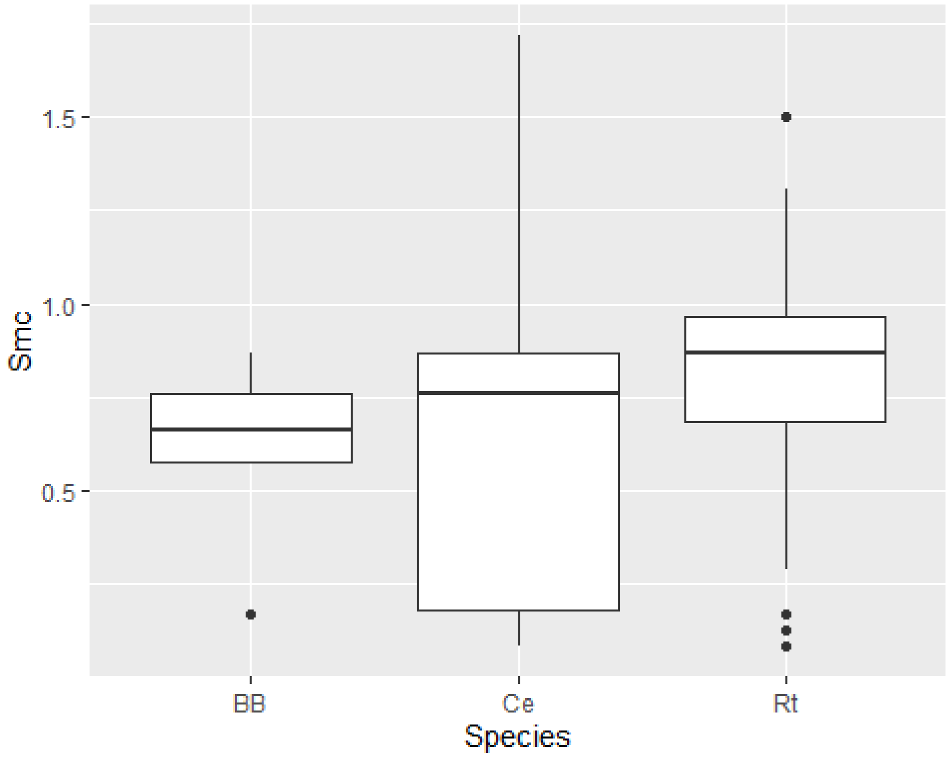


```
ranked <- rank(Quina_Smc)
bf.test(ranked ~ Quina_species, data = Quina) # the variances remain signific
antly differenT

##
##   Brown-Forsythe Test (alpha = 0.05)
## -------------------------------------------------------------
##   data : ranked and Quina_species
##
##   statistic  : 4.48156
##   num df     : 2
##   denom df   : 33.83737
##   p.value    : 0.01876774
##
##   Result     : Difference is statistically significant.
## -------------------------------------------------------------

# Here we have no choice but to use a non-parametric test to search for diffe
rences (see below)

bf.test(Quina_H9 ~ Quina_species, data = Quina)

##
##   Brown-Forsythe Test (alpha = 0.05)
## -------------------------------------------------------------
##   data : Quina_H9 and Quina_species
##
```

```
##   statistic  : 1.059788
##   num df     : 2
##   denom df   : 13.97307
##   p.value    : 0.3728005
##
##   Result     : Difference is not statistically significant.
## -------------------------------------------------------------

ggplot(Quina) +
  geom_boxplot(aes(x = Quina_species, y = Quina_H9)) +
  labs(
    x = "Species",
    y = "H9"
  )
```

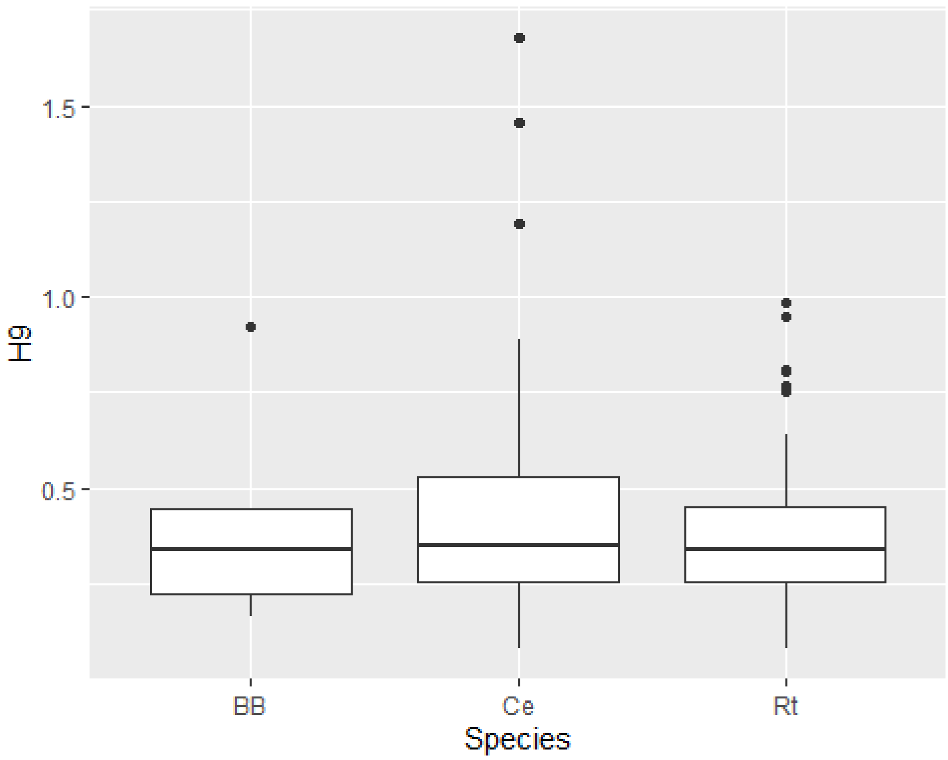


```
bf.test(Quina_H36 ~ Quina_species, data = Quina)

##
##   Brown-Forsythe Test (alpha = 0.05)
## -------------------------------------------------------------
##   data : Quina_H36 and Quina_species
##
##   statistic  : 1.18197
##   num df     : 2
##   denom df   : 22.29411
##   p.value    : 0.3251691
##
```

```
##   Result      : Difference is not statistically significant.
## -------------------------------------------------------------

ggplot(Quina) +
  geom_boxplot(aes(x = Quina_species, y = Quina_H36)) +
  labs(
    x = "Species",
    y = "H36"
  )
```

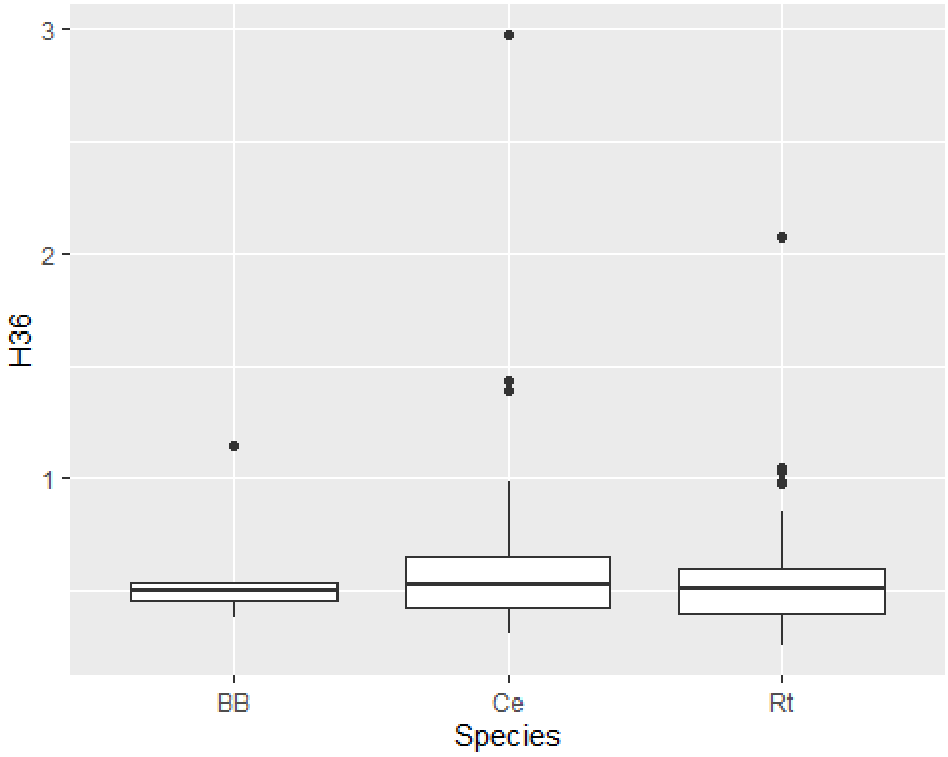


```
bf.test(Quina_H81 ~ Quina_species, data = Quina)

##
##   Brown-Forsythe Test (alpha = 0.05)
## -------------------------------------------------------------
##   data : Quina_H81 and Quina_species
##
##   statistic  : 1.481288
##   num df     : 2
##   denom df   : 14.51811
##   p.value    : 0.2597579
##
##   Result      : Difference is not statistically significant.
## -------------------------------------------------------------

ggplot(Quina) +
  geom_boxplot(aes(x = Quina_species, y = Quina_H81)) +
  labs(
```

```
    x = "Species",
    y = "H81"
  )
```

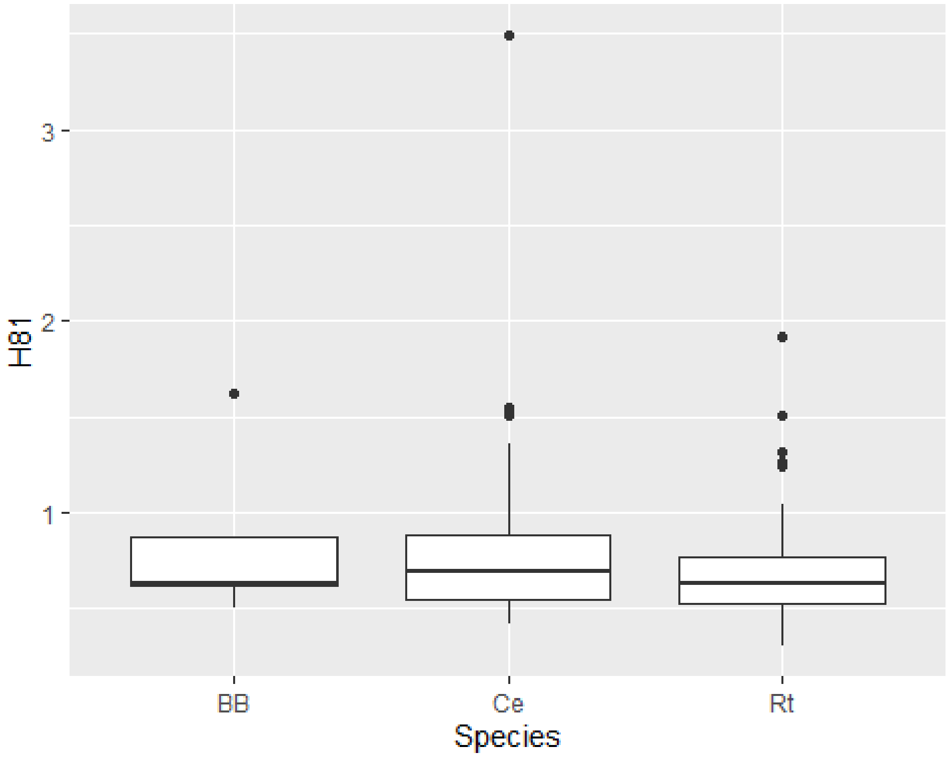

Glm: Impact of Period over each DMTA parameter

```
glm_AnteQ_Asfc0 <- glm(AnteQ_Asfc ~ 1, data = D)
glm_AnteQ_Asfc1 <- glm(AnteQ_Asfc ~ AnteQ_species, data = AnteQ)
Cand.models <- list()
Cand.models[[1]] <- glm_AnteQ_Asfc0
Cand.models[[2]] <- glm_AnteQ_Asfc1
Modnames <- lapply(Cand.models, "formula")
aictab(cand.set = Cand.models, modnames = paste0(Modnames), sort = TRUE)

##
## Model selection based on AICc:
##
##                               K   AICc Delta_AICc AICcWt Cum.Wt       LL
## AnteQ_Asfc ~ AnteQ_species 4 312.20       0.00      1      1 -151.91
## AnteQ_Asfc ~ 1             2 323.76      11.56      0      1 -159.82

marginal <- emmeans(glm_AnteQ_Asfc1, ~AnteQ_species)
pairs(marginal)

##  contrast estimate    SE  df t.ratio p.value
##  BB - Ce     1.333 0.331 107   4.032  0.0003
##  BB - Rt     1.159 0.321 107   3.605  0.0014
##  Ce - Rt    -0.174 0.199 107  -0.878  0.6552
```

```
##
## P value adjustment: tukey method for comparing a family of 3 estimates

glm_AnteQ_epLsar0 <- glm(AnteQ_epLsar ~ 1, data = D)
glm_AnteQ_epLsar1 <- glm(AnteQ_epLsar ~ AnteQ_species, data = AnteQ)
Cand.models <- list()
Cand.models[[1]] <- glm_AnteQ_epLsar0
Cand.models[[2]] <- glm_AnteQ_epLsar1
Modnames <- lapply(Cand.models, "formula")
aictab(cand.set = Cand.models, modnames = paste0(Modnames), sort = TRUE)

##
## Model selection based on AICc:
##
##                               K   AICc Delta_AICc AICcWt Cum.Wt      LL
## AnteQ_epLsar ~ 1              2 465.41       0.00   0.75   0.75 -230.65
## AnteQ_epLsar ~ AnteQ_species 4 467.65       2.24   0.25   1.00 -229.63

glm_AnteQ_Smc0 <- glm(AnteQ_Smc ~ 1, data = AnteQ)
glm_AnteQ_Smc1 <- glm(AnteQ_Smc ~ AnteQ_species, data = AnteQ)
Cand.models <- list()
Cand.models[[1]] <- glm_AnteQ_Smc0
Cand.models[[2]] <- glm_AnteQ_Smc1
Modnames <- lapply(Cand.models, "formula")
aictab(cand.set = Cand.models, modnames = paste0(Modnames), sort = TRUE)

##
## Model selection based on AICc:
##
##                            K   AICc Delta_AICc AICcWt Cum.Wt      LL
## AnteQ_Smc ~ AnteQ_species 4 974.10       0.00   0.56   0.56 -482.86
## AnteQ_Smc ~ 1              2 974.55       0.45   0.44   1.00 -485.22

marginal <- emmeans(glm_AnteQ_epLsar1, ~AnteQ_species)
pairs(marginal)

##  contrast estimate    SE  df t.ratio p.value
##  BB - Ce    -0.939 0.670 107  -1.401  0.3438
##  BB - Rt    -0.678 0.652 107  -1.041  0.5529
##  Ce - Rt     0.261 0.402 107   0.649  0.7935
##
## P value adjustment: tukey method for comparing a family of 3 estimates

glm_AnteQ_H9_0 <- glm(AnteQ_H9 ~ 1, data = AnteQ)
glm_AnteQ_H9_1 <- glm(AnteQ_H9 ~ AnteQ_species, data = AnteQ)
Cand.models <- list()
Cand.models[[1]] <- glm_AnteQ_H9_0
Cand.models[[2]] <- glm_AnteQ_H9_1
Modnames <- lapply(Cand.models, "formula")
aictab(cand.set = Cand.models, modnames = paste0(Modnames), sort = TRUE)
```

```
##
## Model selection based on AICc:
##
##                           K   AICc Delta_AICc AICcWt Cum.Wt    LL
## AnteQ_H9 ~ 1              2 -83.04       0.00   0.69   0.69 43.58
## AnteQ_H9 ~ AnteQ_species 4 -81.43       1.62   0.31   1.00 44.90

glm_AnteQ_H81_0 <- glm(AnteQ_H81 ~ 1, data = AnteQ)
glm_AnteQ_H81_1 <- glm(AnteQ_H81 ~ AnteQ_species, data = AnteQ)
Cand.models <- list()
Cand.models[[1]] <- glm_AnteQ_H81_0
Cand.models[[2]] <- glm_AnteQ_H81_1
Modnames <- lapply(Cand.models, "formula")
aictab(cand.set = Cand.models, modnames = paste0(Modnames), sort = TRUE)

##
## Model selection based on AICc:
##
##                            K  AICc Delta_AICc AICcWt Cum.Wt     LL
## AnteQ_H81 ~ 1              2 27.87       0.00   0.74   0.74 -11.88
## AnteQ_H81 ~ AnteQ_species 4 30.00       2.13   0.26   1.00 -10.81

glm_AnteQ_H36_0 <- glm(AnteQ_H36 ~ 1, data = AnteQ)
glm_AnteQ_H36_1 <- glm(AnteQ_H36 ~ AnteQ_species, data = AnteQ)
Cand.models <- list()
Cand.models[[1]] <- glm_AnteQ_H36_0
Cand.models[[2]] <- glm_AnteQ_H36_1
Modnames <- lapply(Cand.models, "formula")
aictab(cand.set = Cand.models, modnames = paste0(Modnames), sort = TRUE)

##
## Model selection based on AICc:
##
##                            K  AICc Delta_AICc AICcWt Cum.Wt    LL
## AnteQ_H36 ~ 1              2 22.55       0.00   0.78   0.78 -9.22
## AnteQ_H36 ~ AnteQ_species 4 25.14       2.59   0.22   1.00 -8.38

glm_Quina_Asfc0 <- glm(Quina_Asfc ~ 1, data = Quina)
glm_Quina_Asfc1 <- glm(Quina_Asfc ~ Quina_species, data = Quina)
Cand.models <- list()
Cand.models[[1]] <- glm_Quina_Asfc0
Cand.models[[2]] <- glm_Quina_Asfc1
Modnames <- lapply(Cand.models, "formula")
aictab(cand.set = Cand.models, modnames = paste0(Modnames), sort = TRUE)

##
## Model selection based on AICc:
##
##                             K   AICc Delta_AICc AICcWt Cum.Wt      LL
## Quina_Asfc ~ Quina_species 4 445.75       0.00   0.98   0.98 -218.74
## Quina_Asfc ~ 1              2 454.07       8.31   0.02   1.00 -224.99
```

```
marginal <- emmeans(glm_Quina_Asfc1, ~Quina_species)
pairs(marginal)

##  contrast estimate    SE  df t.ratio p.value
##  BB - Ce     1.001 0.515 143   1.946  0.1296
##  BB - Rt     0.338 0.502 143   0.673  0.7797
##  Ce - Rt    -0.664 0.195 143  -3.396  0.0025
##
## P value adjustment: tukey method for comparing a family of 3 estimates

glm_Quina_epLsar0 <- glm(Quina_epLsar ~ 1, data = Quina)
glm_Quina_epLsar1 <- glm(Quina_epLsar ~ Quina_species, data = Quina)
Cand.models <- list()
Cand.models[[1]] <- glm_Quina_epLsar0
Cand.models[[2]] <- glm_Quina_epLsar1
Modnames <- lapply(Cand.models, "formula")
aictab(cand.set = Cand.models, modnames = paste0(Modnames), sort = TRUE)

##
## Model selection based on AICc:
##
##                                K   AICc Delta_AICc AICcWt Cum.Wt       LL
## Quina_epLsar ~ Quina_species 4 622.05       0.00   0.87   0.87 -306.88
## Quina_epLsar ~ 1             2 625.89       3.84   0.13   1.00 -310.90

marginal <- emmeans(glm_Quina_epLsar1, ~Quina_species)
pairs(marginal)

##  contrast estimate    SE  df t.ratio p.value
##  BB - Ce    -0.262 0.941 143  -0.278  0.9583
##  BB - Rt     0.743 0.918 143   0.809  0.6981
##  Ce - Rt     1.004 0.357 143   2.810  0.0155
##
## P value adjustment: tukey method for comparing a family of 3 estimates

kruskal.test(Quina_Smc ~ Quina_species, data = Quina)

##
##  Kruskal-Wallis rank sum test
##
## data:  Quina_Smc by Quina_species
## Kruskal-Wallis chi-squared = 7.272, df = 2, p-value = 0.02636

dunnTest(Quina_Smc, Quina_species, method = "bonferroni")

##   Comparison        Z     P.unadj      P.adj
## 1    BB - Ce 0.591410 0.55424572 1.00000000
## 2    BB - Rt 1.538630 0.12389464 0.37168391
## 3    Ce - Rt 2.395557 0.01659512 0.04978537

glm_Quina_H9_0 <- glm(Quina_H9 ~ 1, data = Quina)
glm_Quina_H9_1 <- glm(Quina_H9 ~ Quina_species, data = Quina)
```

```
Cand.models <- list()
Cand.models[[1]] <- glm_Quina_H9_0
Cand.models[[2]] <- glm_Quina_H9_1
Modnames <- lapply(Cand.models, "formula")
aictab(cand.set = Cand.models, modnames = paste0(Modnames), sort = TRUE)
```

```
##
## Model selection based on AICc:
##
##                             K  AICc Delta_AICc AICcWt Cum.Wt   LL
## Quina_H9 ~ 1                2 -2.86       0.00   0.62   0.62 3.47
## Quina_H9 ~ Quina_species 4 -1.90       0.96   0.38   1.00 5.09
```

```
glm_Quina_H81_0 <- glm(Quina_H81 ~ 1, data = Quina)
glm_Quina_H81_1 <- glm(Quina_H81 ~ Quina_species, data = Quina)
Cand.models <- list()
Cand.models[[1]] <- glm_Quina_H81_0
Cand.models[[2]] <- glm_Quina_H81_1
Modnames <- lapply(Cand.models, "formula")
aictab(cand.set = Cand.models, modnames = paste0(Modnames), sort = TRUE)
```

```
##
## Model selection based on AICc:
##
##                              K   AICc Delta_AICc AICcWt Cum.Wt     LL
## Quina_H81 ~ Quina_species 4 116.41       0.00   0.55   0.55 -54.07
## Quina_H81 ~ 1                2 116.79       0.38   0.45   1.00 -56.35
```

```
marginal <- emmeans(glm_Quina_H81_1, ~Quina_species)
pairs(marginal)
```

```
##  contrast estimate     SE  df t.ratio p.value
##  BB - Ce    0.0411 0.1666 143   0.247  0.9669
##  BB - Rt    0.1669 0.1625 143   1.027  0.5611
##  Ce - Rt    0.1257 0.0633 143   1.988  0.1189
##
## P value adjustment: tukey method for comparing a family of 3 estimates
```

```
glm_Quina_H36_0 <- glm(Quina_H36 ~ 1, data = Quina)
glm_Quina_H36_1 <- glm(Quina_H36 ~ Quina_species, data = Quina)
Cand.models <- list()
Cand.models[[1]] <- glm_Quina_H36_0
Cand.models[[2]] <- glm_Quina_H36_1
Modnames <- lapply(Cand.models, "formula")
aictab(cand.set = Cand.models, modnames = paste0(Modnames), sort = TRUE)
```

```
##
## Model selection based on AICc:
##
##                              K  AICc Delta_AICc AICcWt Cum.Wt     LL
## Quina_H36 ~ 1                2 75.74       0.00   0.66   0.66 -35.83
## Quina_H36 ~ Quina_species 4 77.03       1.29   0.34   1.00 -34.37
```

```
glm_PostQ_Asfc0 <- glm(PostQ_Asfc ~ 1, data = PostQ)
glm_PostQ_Asfc1 <- glm(PostQ_Asfc ~ PostQ_species, data = PostQ)
Cand.models <- list()
Cand.models[[1]] <- glm_PostQ_Asfc0
Cand.models[[2]] <- glm_PostQ_Asfc1
Modnames <- lapply(Cand.models, "formula")
aictab(cand.set = Cand.models, modnames = paste0(Modnames), sort = TRUE)

##
## Model selection based on AICc:
##
##                              K   AICc Delta_AICc AICcWt Cum.Wt      LL
## PostQ_Asfc ~ 1               2 318.12       0.00    0.6    0.6 -157.01
## PostQ_Asfc ~ PostQ_species 4 318.96       0.84    0.4    1.0 -155.29

glm_PostQ_epLsar0 <- glm(PostQ_epLsar ~ 1, data = PostQ)
glm_PostQ_epLsar1 <- glm(PostQ_epLsar ~ PostQ_species, data = PostQ)
Cand.models <- list()
Cand.models[[1]] <- glm_PostQ_epLsar0
Cand.models[[2]] <- glm_PostQ_epLsar1
Modnames <- lapply(Cand.models, "formula")
aictab(cand.set = Cand.models, modnames = paste0(Modnames), sort = TRUE)

##
## Model selection based on AICc:
##
##                                K   AICc Delta_AICc AICcWt Cum.Wt      LL
## PostQ_epLsar ~ PostQ_species 4 512.72       0.00   0.63   0.63 -252.17
## PostQ_epLsar ~ 1               2 513.80       1.08   0.37   1.00 -254.85

marginal <- emmeans(glm_PostQ_epLsar1, ~PostQ_species)
pairs(marginal)

##  contrast estimate    SE  df t.ratio p.value
##  BB - Ce    -0.584 0.601 109  -0.972  0.5958
##  BB - Rt     0.667 0.516 109   1.293  0.4024
##  Ce - Rt     1.251 0.555 109   2.256  0.0666
##
## P value adjustment: tukey method for comparing a family of 3 estimates

glm_PostQ_Smc0 <- glm(PostQ_Smc ~ 1, data = PostQ)
glm_PostQ_Smc1 <- glm(PostQ_Smc ~ PostQ_species, data = PostQ)
Cand.models <- list()
Cand.models[[1]] <- glm_PostQ_Smc0
Cand.models[[2]] <- glm_PostQ_Smc1
Modnames <- lapply(Cand.models, "formula")
aictab(cand.set = Cand.models, modnames = paste0(Modnames), sort = TRUE)

##
## Model selection based on AICc:
##
##                             K    AICc Delta_AICc AICcWt Cum.Wt      LL
```

```
## PostQ_Smc ~ PostQ_species 4 1136.67       0.00   0.51   0.51 -564.15
## PostQ_Smc ~ 1              2 1136.78       0.11   0.49   1.00 -566.34
```

```
marginal <- emmeans(glm_PostQ_Smc1, ~PostQ_species)
pairs(marginal)
```

```
##  contrast estimate   SE  df t.ratio p.value
##  BB - Ce    -19.03 9.74 109  -1.954  0.1287
##  BB - Rt    -13.59 8.36 109  -1.625  0.2397
##  Ce - Rt      5.44 8.99 109   0.605  0.8175
##
## P value adjustment: tukey method for comparing a family of 3 estimates
```

```
glm_PostQ_H9_0 <- glm(PostQ_H9 ~ 1, data = PostQ)
glm_PostQ_H9_1 <- glm(PostQ_H9 ~ PostQ_species, data = PostQ)
Cand.models <- list()
Cand.models[[1]] <- glm_PostQ_H9_0
Cand.models[[2]] <- glm_PostQ_H9_1
Modnames <- lapply(Cand.models, "formula")
aictab(cand.set = Cand.models, modnames = paste0(Modnames), sort = TRUE)
```

```
##
## Model selection based on AICc:
##
##                           K    AICc Delta_AICc AICcWt Cum.Wt    LL
## PostQ_H9 ~ 1              2 -130.95       0.00   0.72   0.72 67.53
## PostQ_H9 ~ PostQ_species 4 -129.09       1.86   0.28   1.00 68.73
```

```
glm_PostQ_H81_0 <- glm(PostQ_H81 ~ 1, data = PostQ)
glm_PostQ_H81_1 <- glm(PostQ_H81 ~ PostQ_species, data = PostQ)
Cand.models <- list()
Cand.models[[1]] <- glm_PostQ_H81_0
Cand.models[[2]] <- glm_PostQ_H81_1
Modnames <- lapply(Cand.models, "formula")
aictab(cand.set = Cand.models, modnames = paste0(Modnames), sort = TRUE)
```

```
##
## Model selection based on AICc:
##
##                            K   AICc Delta_AICc AICcWt Cum.Wt    LL
## PostQ_H81 ~ 1              2 -52.35       0.00   0.76   0.76 28.23
## PostQ_H81 ~ PostQ_species 4 -50.09       2.26   0.24   1.00 29.23
```

```
glm_PostQ_H36_0 <- glm(PostQ_H36 ~ 1, data = PostQ)
glm_PostQ_H36_1 <- glm(PostQ_H36 ~ PostQ_species, data = PostQ)
Cand.models <- list()
Cand.models[[1]] <- glm_PostQ_H36_0
Cand.models[[2]] <- glm_PostQ_H36_1
Modnames <- lapply(Cand.models, "formula")
aictab(cand.set = Cand.models, modnames = paste0(Modnames), sort = TRUE)
```

```
##
## Model selection based on AICc:
##
##                            K    AICc Delta_AICc AICcWt Cum.Wt    LL
## PostQ_H36 ~ 1              2 -107.67       0.00   0.79   0.79 55.89
## PostQ_H36 ~ PostQ_species 4 -105.05       2.62   0.21   1.00 56.71
```
